# Global Profiling of Carbohydrate Active Enzymes in Human Gut Microbiome

**DOI:** 10.1371/journal.pone.0142038

**Published:** 2015-11-06

**Authors:** Tanudeep Bhattacharya, Tarini Shankar Ghosh, Sharmila S. Mande

**Affiliations:** 1 Teach for India, A 903, Tain Square, Fatima Nagar, Pune, Maharashtra, India; 2 TCS Innovation Labs, Tata Consultancy Services Ltd.,54-B, Hadapsar Industrial Estate, Pune, Maharashtra, India; Cairo University, EGYPT

## Abstract

**Motivation:**

Carbohydrate Active enzyme (CAZyme) families, encoded by human gut microflora, play a crucial role in breakdown of complex dietary carbohydrates into components that can be absorbed by our intestinal epithelium. Since nutritional wellbeing of an individual is dependent on the nutrient harvesting capability of the gut microbiome, it is important to understand how CAZyme repertoire in the gut is influenced by factors like age, geography and food habits.

**Results:**

This study reports a comprehensive *in-silico* analysis of CAZyme profiles in the gut microbiomes of 448 individuals belonging to different geographies, using similarity searches of the corresponding gut metagenomic contigs against the carbohydrate active enzymes database. The study identifies a core group of 89 CAZyme families that are present across 85% of the gut microbiomes. The study detects several geography/age-specific trends in gut CAZyme repertoires of the individuals. Notably, a group of CAZymes having a positive correlation with BMI has been identified. Further this group of BMI-associated CAZymes is observed to be specifically abundant in the Firmicutes phyla. One of the major findings from this study is identification of three distinct groups of individuals, referred to as 'CAZotypes', having similar CAZyme profiles. Distinct taxonomic drivers for these CAZotypes as well as the probable dietary basis for such trends have also been elucidated. The results of this study provide a global view of CAZyme profiles across individuals of various geographies and age-groups. These results re-iterate the need of a more precise understanding of the role of carbohydrate active enzymes in human nutrition.

## Introduction

Human gut harbours more than 1000 microbial species. These species, along with their approximately 4.2 million genes, constitute the gut microbiome [1]. A close symbiosis between our cells and the microbiome aids in carrying out several metabolic functions, many of which cannot be performed by the human genome [2]. These functions include modulation of immune response as well as nutrient absorption, protection against pathogens, and most importantly, efficient energy harvest from the diet. One of the key steps in the efficient harvest of energy involves degradation of carbohydrate components from our food.

Carbohydrates are the chief energy source and are also important ‘building blocks’ of life. Besides, they play an important role in host-microbe interactions by modulating various functions at the interface between host and the environment [3, 4]. Dietary carbohydrates can be broadly categorized as simple (eg., fructose, galactose and glucose) and complex (eg., cellulose, glycan, starch, glycogen) [5]. Breakdown of these carbohydrates into components that can be absorbed by the intestinal epithelium is typically performed by three broad classes of enzymes. Based on the mechanism of action, these enzyme classes are referred to as Glycoside Hydrolases (GHs), Carbohydrate Esterases (CEs) and Polysachharide Lyases (PLs). These three classes of enzymes are referred to as Carbohydrate active enzymes (CAZymes). Enzyme belonging to the CAZyme classes are further classified into families and sub-families based on their sequence and fold similarity in Carbohydrate active enzymes database (http://www.cazy.org). The enzymes belonging to each sub-family have been shown to have diverse substrate specificity [6, 7].

In spite of being a critical source of energy, the CAZyme repertoire encoded by the human genome is minimal (approximately 17 enzymes). Most of the carbohydrate degradation is performed by the resident gut microbiome. The capacity for carbohydrate degradation by the gut flora is immense. For example, the genome of *Bacteroides thetaiotaomicron* encodes as many as 260 GHs [8]. Since gut microbiome degrades complex dietary carbohydrates that cannot be degraded and digested by human enzymes into human digestable substrates like butyrate, it can be considered as ‘energy harvesting’ machines in the human body. The repertoire of CAZymes thus plays an important role in determining the nutritional status of the individuals. This role becomes particularly important given that diseases arising out of improper nutrition, like obesity [9–14] and malnutrition [15, 16], are fast emerging as global menaces.

Individuals with different CAZyme profiles of their gut microbiomes are likely to have different metabolic capacities with respect to carbohydrate degradation and absorption by the intestinal epithelium. It has been pointed out that while the microbiome of a healthy individual is like a ‘rainforest’, which is robust to external changes and helps in even channelization of input energy, a dysbiotic microbiome is similar to a ‘fertilizer run off’ having a predominance of a few species that may result in abnormal energy transductions [17]. A dysbiotic state may lead to unwanted circumstances that may be considered as analogous to ‘algal blooms’.

A few genome-level analyses have been performed to investigate the carbohydrate degrading potential of several microbes and their prospective roles in the human gut [13]. However, given that a large proportion of gut microbial species are hitherto uncharacterized, the purview of these studies is limited to the culturable/sequenced minority (whose genome sequences are already available). In this paper, we present an extensive analysis of CAZyme profiles in the guts of 448 individuals from diverse geographies and age groups.

## Results

### 'Core' group of CAZymes in the human gut

Extensive analyses of 448 publicly available human gut metagenomic data from nine geographies were performed. Results of the analyses revealed that 206 out of 260 documented CAZyme families are present in the human gut microbiome. Eighty nine out of the identified CAZyme families were observed to be ubiquitously present in at least 85% of the samples (S1 Fig, S1 Table).

### Correlation of Abundance, Diversity and Functional rarefaction of gut associated CAZymes with Age

**Fig 1:**
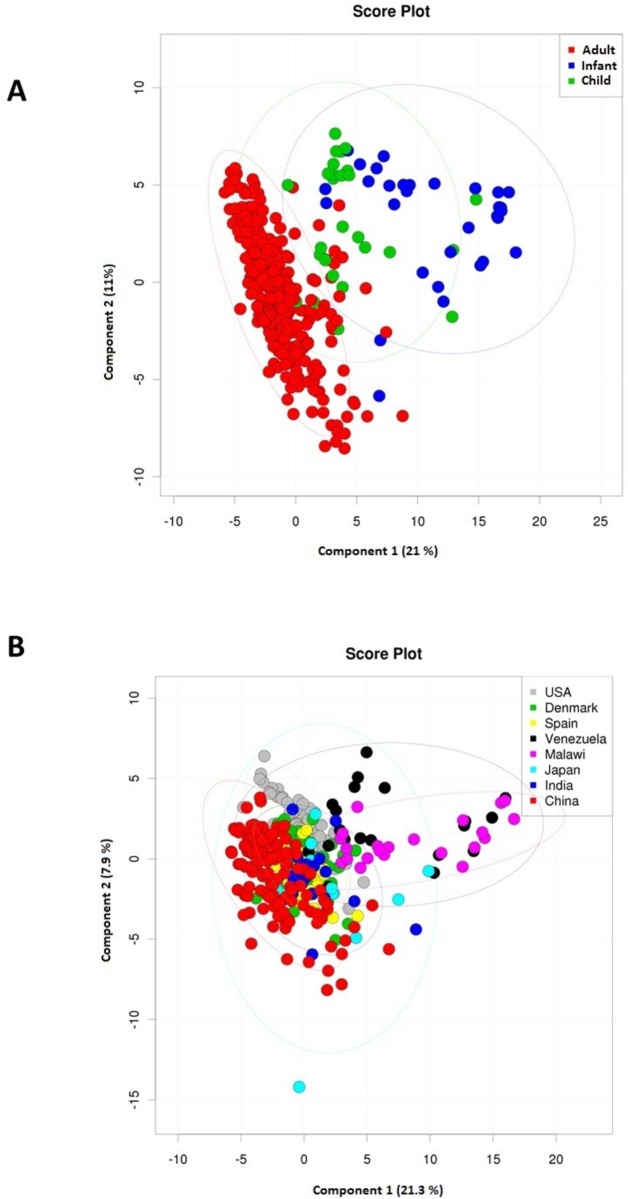
Differential CAZyme profiles across age and geographies. Partial Least Square Discriminant Analysis (PLS-DA) score plots showing global profiles of CAZymes across **(a)** Age and, **(b)** Geography. While there is a clear distinction in the CAZyme profiles of children/infants as compared to adult individuals, the differences among the adult individuals of various geographies are relatively subtle. However these differences are with respect to the overall CAZyme profiles.

Results of the multivariate Partial Least Square Discriminant Analysis (PLS-DA) indicated distinct composition of CAZymes constituting the guts of infants as well as children as compared to those of adults (Fig 1A). Further, the geography-wise PLS-DA revealed that the CAZyme profiles in the guts of the Malawi, Venezuelan, Japanese and Indian nationalities to be different (Fig 1B). This could be because of the distinctly low average age of these individuals. CAZyme families specifically involved in the degradation of simple carbohydrates (like lactose, sucrose) namely, GH1 and GH13, were observed to be specifically abundant in the infant gut microbiomes (S2 Table). On the other hand, apart from simple carbohydrate degrading CAZymes, the children/adult gut was found to contain several complex carbohydrate degrading enzymes (S3 Table). These observations suggest that, with intake of diet containing complex carbohydrates (in addition to simple sugars), the gut microbiome enriches itself with complex carbohydrate degrading enzymes.

**Fig 2:**
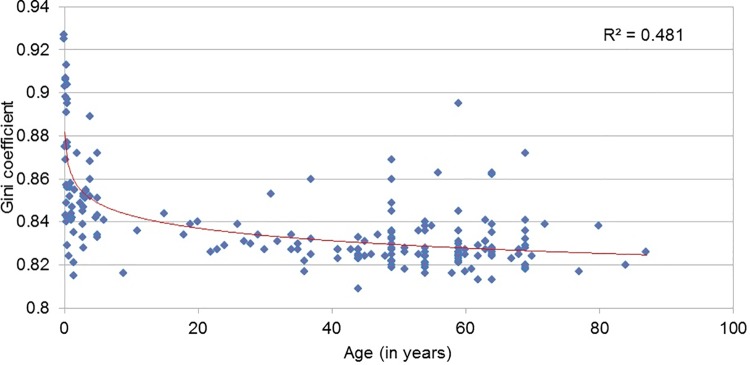
Variation of contribution index (expressed as GINI coefficient) of CAZyme families in the human gut with age. The GINI coefficient, an indicator of functional rarefaction, varies as a logarithmic function with age. Adult individuals have a lower GINI coefficient, indicating more equal distribution of CAZyme families, but with progressively younger age, fewer CAZyme families contribute to a greater proportion of CAZymes, thereby increasing the GINI coefficient. Also, high GINI coefficients, close to a maximum of 1, indicate frequency distribution of CAZyme families is highly non-uniform.

The abundance and diversity of CAZymes indicate the overall number of CAZymes and CAZyme families detected across various gut microbiomes, respectively. In order to evaluate the equality/inequality of contribution of CAZyme families in the human gut, GINI coefficient (described in the methods section, S1 Text) was used. In the present case, while a higher GINI coefficient indicates that only a few CAZymes account for a large proportion of overall functions, a lower GINI coefficient indicates that there are many CAZymes which contribute towards a particular function. Results of our analysis indicated a logarithmically decreasing trend of GINI coefficient with age (Fig 2). A high GINI coefficient was found in infants, indicating that a few CAZyme families contribute to a greater proportion of CAZyme repertoire. The observed decrease of GINI coefficient with age indicates an inequality of contributions of various CAZymes at younger age. These results indicate that CAZyme repertoire in the gut probably evolves with age. Beyond a certain age (during childhood), the GINI coefficient appears to be invariant (Fig 2). Thus, the abundance, diversity and functional rarefaction index of CAZymes in the gut probably change during childhood and get stabilized after a certain age.

In order to study the variation of CAZymes with age in adult individuals, Chinese, Danish and Spanish populations (for whom the age information was available) were analyzed. The overall abundance and diversity of CAZymes in the Chinese cohorts (S4 Table) did not show any correlation with age of the individuals (R^2 for abundance = 0.02; R^2 for diversity = 0.00) (S2 Fig). A similar observation was also noticed for the Danish and Spanish populations (R^2 for abundance: 0.01; R^2 for diversity: 0.01) (S4 Table; S3 Fig). Thus, no trend seems to exist between the diversity as well as abundances of CAZymes with age in case of adult individuals (S2 and S3 Figs). This trend is similar to that obtained using the GINI coefficient analysis, where in the functional diversity of the CAZyme repertoire remains invariant after a certain age.

### Correlations of abundances of CAZymes with BMI

**Fig 3:**
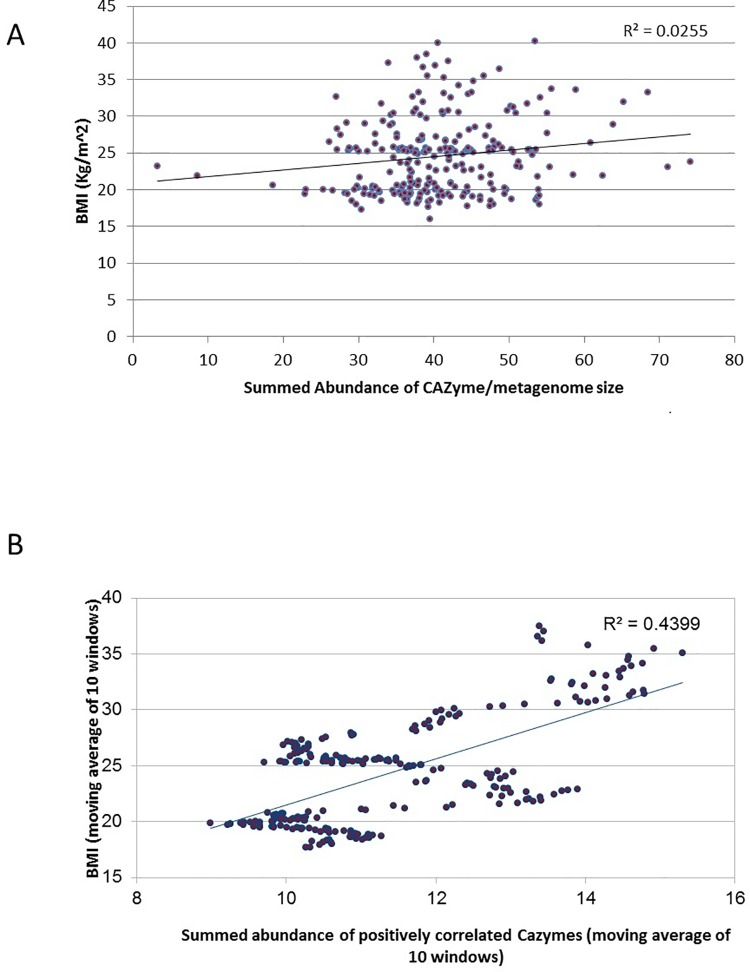
Variation of CAZyme abundances with BMI. **(a)** Correlation of BMI with summed abundances of all gut-associated CAZymes **(b)** Correlation of BMI with summed abundances of the 10 CAZyme families showing significant positive correlation with BMI. The overall summed abundance of all gut associated CAZyme families does not show any correlation with the BMI of the individuals. However, obtaining the correlation of the individual CAZyme families identified 10 such families (listed in Table 1) having a significantly positive correlation with BMI. The correlation of the summed abundances of these 10 families (computed using a sliding window based approach explained in the Methods section) with the BMI was observed to be even more significant (R^2 = 0.44, P < 0.01).

The overall abundances of CAZymes in individuals from Japan, France, Denmark, China, Spain, France, Italy (for whom BMI metadata was available) was observed to have a weak positive correlation (R^2 = 0.0255, Corrected P-value > 0.05) with BMI of the subjects (Fig 3A). However, analyzing the correlations of individual CAZymes with BMI indicated certain interesting trends. 10 CAZyme families were observed to have significant positive correlations, (P-value < 0.05, corrected using Bonferroni) (Table 1). Seven of the positively correlated CAZyme families were found to digest complex carbohydrates (Table 1). The cumulative abundance of these 10 CAZyme families (obtained using a sliding window based approach as described in Methods) was observed to show a much higher positive correlation with BMI (R^2 = 0.44) (Fig 3B). These results suggest that while the overall abundance of CAZymes does not show any relationship with an individual's BMI, bacteria specifically harboring these 10 CAZyme families (whose cumulated abundances have a statistically significant correlation with BMI) might act as one of the causative factors of obesity.

**Table 1:** List of CAZyme families having positive correlation with BMI along with their broad carbohydrate substrate classifications.

CAZyme Family	Correlation Coefficient (significant P-value < 0.05 using the Bonferroni method)	Type of Carbohydrate substrate
GH113	0.27	Simple
GH13	0.34	Starch
GH25	0.32	Complex
GH26	0.23	Complex
GH36	0.26	Complex
GH53	0.21	Complex
GH72	0.21	Complex
GH73	0.39	Complex
GH77	0.29	Starch
GH94	0.28	Complex

**Fig 4:**
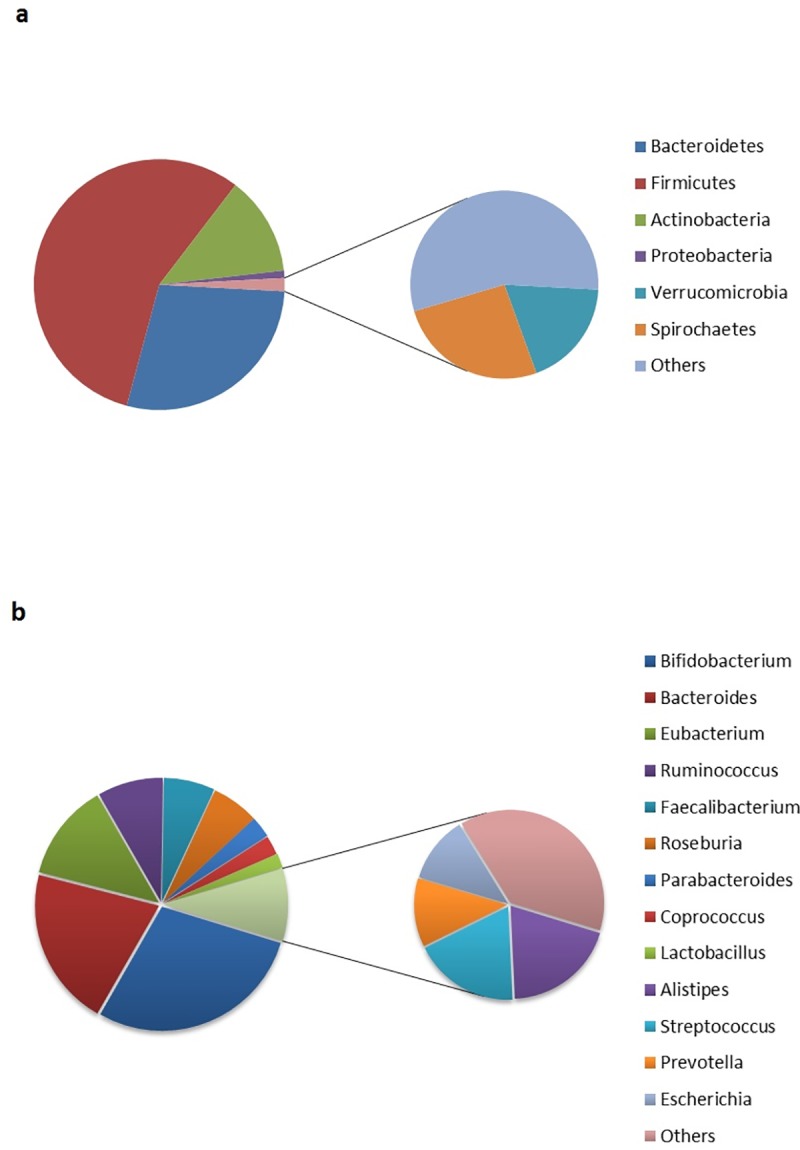
Taxonomic origins of the 10 CAZyme families showing significant positive correlation with BMI. Distribution of contributing clades at (**a)** Phylum level and **(b)** Genus level. Several genera belonging to Firmicutes phylum, like Roseburia, Faecalibacterium, Ruminococcus and Eubacterium, were observed to harbor these CAZyme families, two genera, namely Bifidobacterium (belonging to Actinobacteria) and Bacteroides (belonging to Bacteroidetes) accounted for almost half the proportion of such CAZymes.

Analysis of the taxa affiliations of the above mentioned positively correlated CAZyme markers revealed that majority of them belonged to the Firmicutes phyla (Fig 4A). This observation, along with earlier reports which indicated a link between higher Firmicutes to Bacteroidetes ratio in the gut of obese individuals [10,11,14], suggest possible role of the identified 10 CAZyme families in obesity. Interestingly, while (as expected) several genera belonging to Firmicutes phylum, like Roseburia, Faecalibacterium, Ruminococcus and Eubacterium, were observed to harbor these CAZyme families, two genera, namely Bifidobacterium (belonging to Actinobacteria) and Bacteroides (belonging to Bacteroidetes) accounted for almost half the proportion of these marker CAZymes (Fig 4B). The above results indicate that while the Firmicutes phyla (as a whole) have a higher prevalence of such efficient energy-harvesting enzymes, presence of specific genera belonging to the Bacteroidetes and Actinobacteria phyla may also enhance the energy-harvesting capabilities of the gut.

### Variation of CAZyme repertoire across geographies

#### Abundances and diversity of CAZymes across geographies

**Fig 5:**
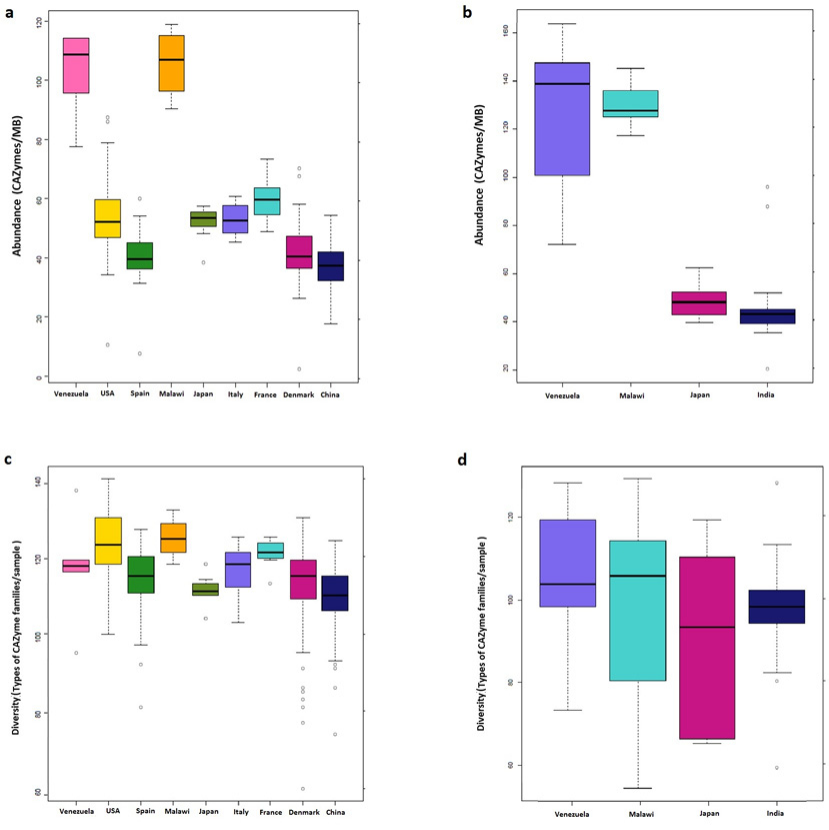
Variation of the abundance and diversity of CAZymes across geographies. Variation of the abundance of CAZymes across **(a)** Adult individuals belonging to various geographies and, **(b)** Children/infant individuals belonging to four geographies. Variation of the diversity of CAZymes across **(c)** Adult individuals belonging to various geographies and, **(d)** Children/infant individuals belonging to four geographies. For the adult individuals, while there is little variation in the diversity of CAZymes across countries, there are country-specific trends in the abundances of various CAZyme families. High CAZyme/MB content in individuals from rural Venezuela and Malawi may be attributable to their carbohydrate rich diet. It also shows that each adult individual, in general, have a consistent diversity across countries. However, in children and infants, in whom the gut flora is developing and unstable, diversity of CAZymes fluctuates greatly.

Certain interesting patterns were observed when the abundances and diversity of CAZymes were compared across the gut microbiomes of adults from different nationalities (Figs 5 and S3, S4 Table). Overall, for adult individuals, diversity of CAZymes was observed to be similar across all nationalities. In contrast, the abundances of CAZymes were observed to vary across different geographies (Fig 5). Adults from Malawi and Venezuela were observed to have an aberrantly high abundance of CAZymes (Figs 5 and S3) as compared to other groups (ANOVA, p < 0.01, corrected for multiple tests, post-hoc Tukey’s HSD). Besides the Malawian and Venezuelan populations, the abundances of CAZymes in adult samples from the remaining seven geographies were found to be relatively similar. However, comparing the abundances of CAZymes in the individuals belonging to these seven nationalities indicated that their CAZyme repertoires could be further divided into two groups. The first group, consisting of USA, Japan, Italy and France, had a higher abundance of CAZymes as compared to the second group, consisting of Denmark, Spain and China (ANOVA, P-value < 0.05 corrected using Bonferroni). This indicates certain geography-specific trends of the abundance of CAZymes. While the under-pinning of such differences will require extensive analyses, prima facie it might be suggested that such difference may be the result of dietary differences of the Malawian and Venezuelan populations with the rest.

The abundances of CAZymes in the gut microbiomes of children/infant belonging to Malawi and Venezuela were found to be higher than those in Japanese and Indian children/infants (Fig 5B). Thus, in addition to dietary and weaning habits, it appears that Japanese and Indian children/infants have a comparatively lower diversity of CAZymes, probably due to differences in dietary intake.

It was also observed that while the diversity of CAZymes in adults (Fig 5A) was consistent throughout with considerably smaller spread, it varied widely in infants and children (Fig 5B). Such huge inter-sample variances are a direct reflection of the developing microbiome, a reflection of the unstable microbiome at a younger age [17]. It was also observed that the CAZyme repertoire of Indian children had consistently less inter-sample variability as compared to other geographies, both in terms of abundance and diversity. This could be attributed to the fact that all Indian children have been sampled from the same locality while the Japanese, Malawi and Venezuelan samples have been collected from different geographical locations within the country [15, 18].

#### Geography-specific trends in CAZyme profiles

While abundances and diversities provide only an overall picture of the CAZyme compositions in a gut metagenome, it is important to understand how the CAZyme profile (the presence/abundance of the individual CAZyme families) varies with geographies. Fig 1B indicates that, besides the distinct CAZyme profiles of gut microbiomes of the Malawi, Venezuelan, Japanese and Indian nationalities (which may be primarily due to the lower age of the individuals), there is a little variation in the overall CAZyme profiles across the other nationality groups (which are dominated by adult individuals). The CAZyme profiles of the gut microbiomes from the Chinese individuals were observed to be marginally different from the Americans, albeit with considerable overlap (Fig 1B). The above results indicate that while the CAZyme profiles differ noticeably among adult, children and infants, the geography-specific variations are relatively subtle. This does not exclude the possibility of the presence of certain geography-specific CAZyme families (specifically present in the gut microbiota of individuals belonging to certain geographies).

#### Geography specific CAZymes

In order to investigate the subtle differences in the CAZyme profiles in the guts of adult individuals across different geographies (and identify geography specific CAZyme families), CAZyme families that were over-represented or under-represented in the four major continental cohorts (Asian, American, European and South America-Africa) were identified. CAZymes specific to the four geography-based cohorts, identified using Welch T-test (P< 0.05, corrected using Benjamini-Hochberg FDR method), are shown in S4 Fig. While four CAZyme families were found to be distinctly different in each of the Asian and North American cohorts, five were identified to be differentially abundant in European individuals. The South America-Africa cohort (primarily consisting of tribal individuals) had as many as 15 CAZyme families that were distinctly abundant as compared to the other cohorts (S4 Fig). The detailed functional annotation of digestive CAZymes which are specific for all pairs of countries is given in S3 Table. In summary, while certain CAZyme families were observed to be differentially abundant in specific cohorts, the higher number of differentially abundant digestive CAZymes identified in South America-Africa cohort may indicate an adaptation to indigenous cohort-specific diets. The higher number of differentially abundant CAZymes further indicates the consequence of diverse fiber-rich diet that is specific to the tribal individuals.

### ‘CAZotypes’: Gut microbial communities having similar CAZyme profiles

#### CAZotypes across geographies

**Fig 6:**
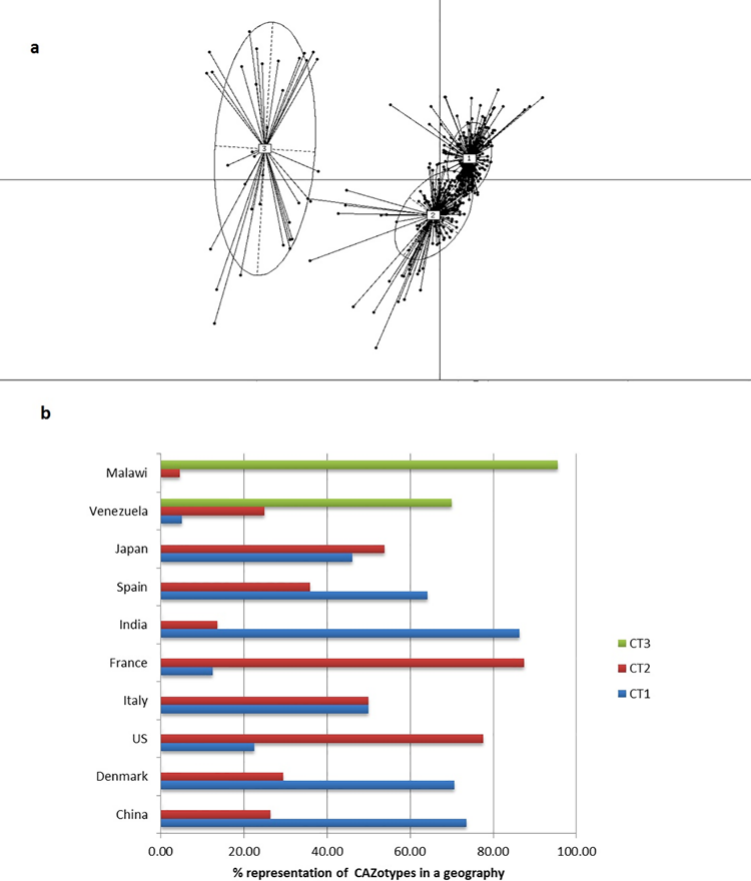
Identification of CAZotypes. **(a)** Distinct clustering of gut microbiomes based on similarities in the CAZyme profiles obtained using the BCA analysis. Each cluster represents a ‘CAZotype’. **(b)** CAZotype distributions across various nationalities. The CAZyme profiles of the 448 gut microbiomes could be grouped into three distinct clusters, referred to as CAZotypes. Further, the gut microbiomes from individuals of various geographies, were observed to have differential preferences to belong to one of the three CAZotypes.

Given the observed trends of diversity as well as abundance of CAZymes in the gut microbiomes of individuals belonging to the different geographies (mentioned in the above section), the subsequent investigation focussed on whether the individuals could be grouped (into clusters) based on their CAZyme profiles. The ‘Between Class Analysis’ (BCA) was performed for this purpose (described in methods section). Results of the BCA analysis indicated that the 448 individuals from nine different geographies could be grouped into three distinct clusters, based on the similarities in their CAZyme profiles (Fig 6A). These three clusters were referred to as ‘CAZotypes 1, 2 and 3’. Thus, each cluster represented groups of individuals having similar abundances of various CAZyme families in their gut microbiome.

Subsequently, the percentage representation of gut microbiomes in three clusters were analysed (Fig 6B; S5 Table). It was observed that while the gut microbiomes in the Chinese, Indian, Danish and Spanish individuals showed a clear preference towards CAZotype-1, those in the French and American populations preferred CAZotype-2. On the other hand, the CAZyme repertoire in the gut microbiomes of (predominantly children/infant) individuals from rural Africa (Malawi) and South America (Venezuela) belonged to an entirely different CAZotype-3. The CAZyme profiles in the guts of the Japanese and Italian populations were observed to show equal preference to CAZotypes 1 and 2. Thus, although the CAZotypes were observed to be cross-continental in nature, certain geography-specific preferences were observed. Similar geography-specific trends were reported recently for antibiotic resistance gene profiles, referred to as ‘Resistotypes’ [19].

#### Key genes and taxa specific to different CAZotypes

Each CAZotype is a reflection of the presence of distinct groups of CAZymes in the gut microbiomes of individuals belonging to that CAZotype. A detailed analysis identified 14, 9 and 17 digestive CAZyme families to be enriched in CAZotype-1, CAZotype-2 and CAZotype-3, respectively (S5 Fig). These three sets of CAZyme families could be regarded as ‘marker CAZyme families’ for the three different CAZotypes. Notably, a confirmatory analysis using Partial Least Square (PLS) regression also identified this set of CAZymes as among the top predictors for the three different CAZotypes (See S2 Text). The differential propensities of different CAZyme families associated with the three CAZotypes are likely to be a reflection of the inherent differences in the CAZyme profiles of the resident gut microbiota.

**Fig 7:**
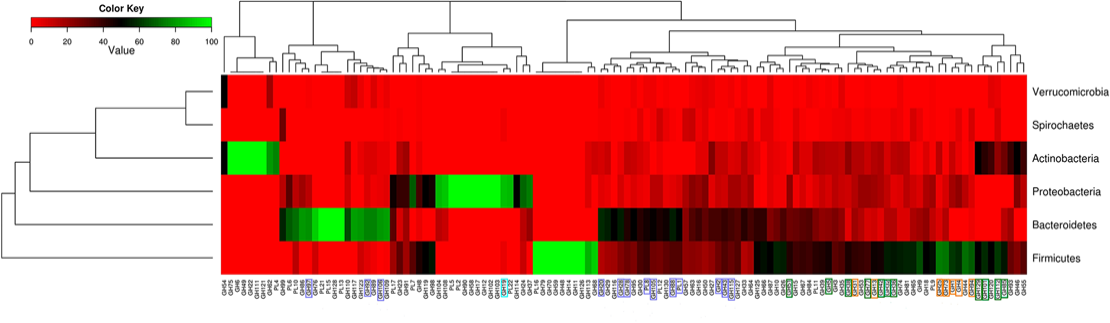
Phylum specific signatures of CAZyme family abundances. Heatmap of relative percentage abundance of the different digestive CAZyme families across top contributing phyla. The marker CAZyme families identified significantly abundant in the CAZotype-1, CAZotype-2 and CAZotype-3 are highlighted in Blue, Orange and Green text boxes, respectively. Distinct phylum specific signatures in the abundances of different CAZyme families are observed. Further, mapping of marker CAZyme families of the three CAZotypes on to the heatmap indicates that enzymes specific to each CAZotype show distinct phylum specific abundances. For example, a driver enzyme from CAZotype-1 maps perfectly well in the ‘Bacteroidetes specific’ region of the heatmap.

**Fig 8:**
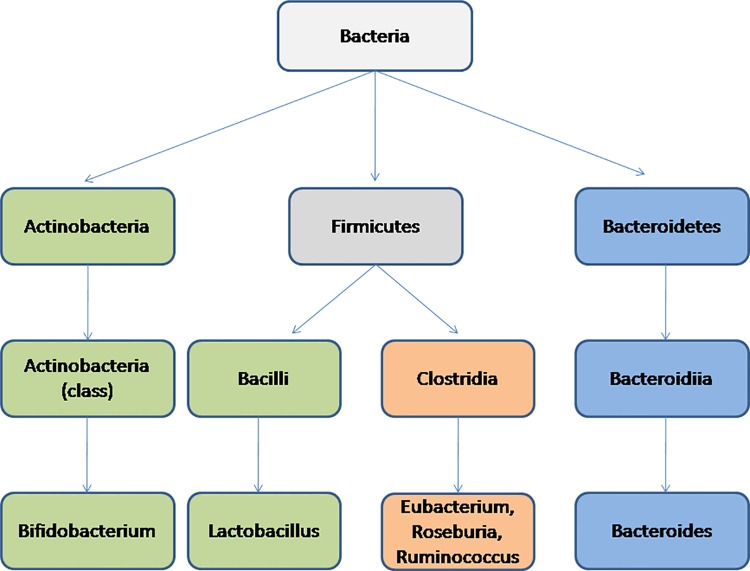
Taxonomic lineage of key drivers of the CAZotypes. Abundances of the different taxonomic lineages specific to the different CAZotypes. While CAZotype-1 is characterized by the over-representation of the genus Bacteroides lineage (phylum Bacteroidetes), CAZotype-2 has an over-representation of the genera Eubacterium, Ruminococcus and Roseburia (belonging to the Firmicutes phyla) and the genera Escherichia (belonging to Proteobacteria). The CAZotype-3, dominated by infant gut microbiomes, was observed to be dominated by two genera namely, Lactobacillus (phyla Firmicutes; class Bacilli) and Bifidobacterium (phyla Actinobacteria).

In order to identify whether any specific taxonomic groups(s) could be associated with one or more CAZotypes, the taxonomic origins of the CAZyme families were investigated (Figs 7 and 8). It was observed that different CAZyme families had distinct phylum-specific signatures. Further, it was also observed that the marker CAZyme families of the three CAZotypes had distinct phylum specific abundances. For example, most of the marker CAZyme families of CAZotype-1 were observed to be specifically abundant in the phylum ‘Bacteroidetes’ (the genus ‘Bacteroides’) (Fig 8). In addition, CAZymes specifically abundant in CAZotype-2 had an over-representation of various genera namely, Eubacterium, Ruminococcus, Roseburia (all belonging to the phylum Firmicutes) and the genus Escherichia (belonging to the phylum Proteobacteria). On the other hand, the CAZotype-3, having the highest number of infants, was observed to be dominated by CAZymes primarily belonging to the two genera namely, Lactobacillus (phyla Firmicutes; class Bacilli) and Bifidobacterium (phyla Actinobacteria). These results indicate that the CAZotype to which an individual belongs is dictated by the microbial composition in his/her gut. This is further confirmed by a separate confirmatory analysis using PLS regression, where in the taxonomic profile of a gut microbiome (at the level of genus) could account for around 70% variance in the CAZotypic affiliations of individuals (Refer to S2 Text). Thus, the CAZotype (and hence the CAZyme profile) is probably a reflection of the resident gut microflora.

## Discussion

Human gut associated microflora plays a key role in human health and nutrition [4, 11–12, 15–16]. Human genome encodes for only a few carbohydrate degrading enzymes. Consequently, we are reliant on our gut microbiome to achieve maximal efficiency from a carbohydrate-based diet [2, 4, 10–12, 14]. In spite of overwhelming scientific finding indicating an indispensable role of gut microbes in carbohydrate utilization [10–15], to the best of our knowledge, comprehensive analyses, providing a global picture of the CAZyme landscape in gut, are currently absent. The present study is based on a comprehensive analysis of 448 publicly available human gut metagenomes from ten different geographies. While the availability of such a large set of gut metagenomes from diverse geographies enables obtaining statistically meaningful insights, certain confounding factors still remain. A key factor is the average age of the individuals within the cohorts from different geographies. For example, while the Italian and French cohorts consisted of elderly individuals, the Malawian, Indian and Venezuelan cohorts were dominated by infants and children.

Although age and geographical location of individuals have been reported earlier to be important factors influencing the gut community structure [18, 20], diet plays the dominant role in modulating the gut microbiome. Individuals belonging to the age and (especially) different geographical locations are likely distinct dietary patterns. Keeping this is in mind, the current study has profiled trends in the CAZyme repertoire in the gut microbiomes across age and geography. Although metagenomic samples are collected from individuals from various continents with different lifestyles, the presence of 89 core CAZyme families in the gut microbiota indicates that one third of the CAZyme repertoire is common across all individuals, irrespective of age and geography. Further, although regional variations are observed, the abundance, diversity and GINI coefficient of the CAZyme repertoire show relatively subtle variations across the gut microbiome of adult individuals. In spite of the basic resemblance to each other, it appears that CAZyme profiles are probably shaped by diet. This is reflected in the extremely high abundance of CAZymes in the guts of individuals from remote rural Africa and Latin America, as compared to those from urban westernized populations. Since all adult samples, except those from Malawian and Venenzuela, belong to urban areas, it is probable that higher intake of fiber and starch in Malawian and Venezuelan individuals may be responsible for highly enriched CAZyme repertoire in the gut microbiome of individuals belonging to these two nationalities. Previous studies have indicated that, as compared to rural settings, the western diet is characterized by higher amount of simple sugar or fats (and lower proportion of complex carbohydrates) [20]. Thus, it is likely that individuals living in rural settings (thriving on a diet of complex carbohydrates) have adapted to a more saccharolytic mode. In this context, given that the current paper is based on a bioinformatics analysis of publicly available datasets (analyzed in previous studies) and considering the fact that majority of these studies (which were performed with varying objectives) did not perform a metabolite profiling of the gut microbiomes, the profiles of critical dietary metabolites like Short Chain Fatty Acids (SCFAs) are not available for the gut microbiomes considered herein. However the trends observed in the current study can form the basis/motivation of further studies which using a combination of metagenomic and metabolomic investigation, provide a much more holistic view of the structure, function and dietary specificities of the gut CAZyme repertoire.

The current study also revealed that the CAZyme repertoire of the gut microbiome of adults is different from that in infants and children, both in terms of diversity and richness. Previous studies have shown that gut microflora stabilizes after three years of age [17]. Our analysis, using the GINI coefficient, also indicates the stabilization of CAZyme repertoire in the early stage of childhood. Thus, unlike the variation of antibiotic resistance genes with age [19], the current study indicates that, after a certain age (early childhood), the variation in CAZyme profile is minimal. This inference is further bolstered by the fact, Bacteroidetes and Firmicutes, which forms the bulk of a matured gut microflora, also contributes to the bulk of CAZyme repertoire. However, a dysbiotic gut microbiome in an individual is likely to hamper his/her carbohydrate harnessing abilities. Further, the relatively homogeneity in the gut CAZyme profiles in the guts of the Indian children indicates that ‘young’ microbiome is more prone to ‘local’ effects because of exposure to a similar environment and diet, while such differences even out as the gut flora matures and acquires robust stability.

One key observation in our study pertains to the identification of certain digestive CAZymes that show high correlation with BMI. Further investigation revealed that such enzymes mostly belonged to Firmicutes phyla. Further, previous studies have indicated obesity to be linked to higher Firmicutes to Bacteroidetes ratio [10, 12]. In contrary, a recent study has shown presence of higher saccharolytic potential in Bacteroidetes as compared to Firmicutes [13]. The results of the present study provide an explanation to the above apparent paradox about obesity. The present analysis revealed that although Bacteroidetes phylum is indeed enriched with CAZymes (having a high overall abundance of CAZymes), the chief contributors of the obese-specific CAZymes (with significant positive correlation with BMI) are Firmicutes.

Identification of the three CAZotypes, based on similarity of the CAZyme profiles in the guts of individuals, is also one of the key findings of the current study. The nomenclature ‘CAZotype’ is analogous to ‘enterotype’ or ‘resistotype’ [19, 21]. The geography preferences for these CAZotypes were also observed. The present study also identified certain taxonomic lineages as the markers of the different CAZotypes (Fig 8). While CAZotype-1, predominant in Indian samples, is chiefly driven by Bacteroidetes phylum, CAZotype-3, predominant in rural Afro-Amazonian individuals is rich in Actinobacteria and Lactobacillus. CAZotype-2, on the other hand, predominantly found in westernized US and French population, is dominated by Clostridial clade of Firmicutes phyla.

The present study reveals the global picture of CAZyme profiles across various geographies and age. The current study can form a basis for further investigations into CAZyme profiles and the gut microbes harboring them, on much larger cohorts of individuals. At a time when the obesity endemic is affecting the developed world and malnutrition plagues the developing countries, the results of the present study re-iterate the need of more precise understanding of the role of carbohydrate active enzymes in human nutrition.

## Methods

### Datasets used

Assembled microbial genomic fragments or contigs corresponding to the human gut metagenomes from different geographies were downloaded from the following sources. While the metagenomes for four Danish, two American, four Spanish, eight French, six Italian and 13 Japanese samples were downloaded from http://www.bork.embl.de/Docu/Arumugam_et_al_2011/data/contigs/, those corresponding to 81 Danish and 35 Spanish individuals were downloaded from ftp://public.genomics.org.cn/BGI/gutmeta/Single_Sample_contig/. Further, 144 Chinese datasets were obtained from ftp://climb.genomics.cn/pub/10.5524/100001_101000/100036/AssemblyContigs/. Contigs corresponding to 22 Venezuela and 20 Malawi samples were downloaded from MG-RAST server (http://metagenomics.anl.gov). In addition, in-house assembled metagenomic datasets from 22 Indian children were also considered for this study. These data sets were previously analyzed in two studies by Ghosh *et al* and Gupta *et al* [15, 16]. Apart from this, contigs corresponding to 90 gut metagenomes obtained from American individuals, sequenced as part of the Human Microbiome Project, were downloaded HMP-DACC website (http://www.hmpdacc.org/HMASM/). The details of the various metagenomic contig datasets, along with their download links and the corresponding references have been provided in S4 Table.

### Detection of CAZymes in the gut metagenomes

Homologs of various carbohydrate active enzymes in the gut metagenome were detected by performing BLASTx searches of corresponding contigs against the carbohydrate active enzymes database [6, 7, 22]. In order to reduce the size of the database to suit the computational needs, the database sequences were pre-clustered using CD-HIT at 95% identity [23]. As the main motivation behind this study was to investigate the catalytic potential of human gut-flora associated CAZymes, only the classes of catalytic CAZyme families present in this database were considered. In other words, the analysis was performed specifically considering CAZyme families belonging to the classes of Glycosyl hydrolases (GH), Polysaccharide lyases (PL), Glycosyltransferases (GT) and Carbohydrate Esterases (CE). Thus the analysis did not include non-catalytic Carbohydrate Binding Modules (CBM) and other auxilliary enzymes (AA). Extensive parameter exploration was done to ensure that the maximum number of homologs of CAZymes is detected in hitherto uncharacterized gut microbiota and yet stringency is maintained to avoid false positives. Using a modified search criteria as per Cantarel *et al* [24], the following criteria was used for detecting homologs for each 'best' scoring alignment: i) E-value < 1e -05 ii) Bit-score per alignment length > 1.0 iii) Query coverage > = 75%.

### Abundance, diversity and GINI coefficient of CAZymes

Each BLAST hit was tagged to particular CAZyme family (GH1, GH2 etc.). The number of hits belonging to each CAZyme family for each metagenome was collated and this was subsequently represented as a matrix, termed as the 'abundance profile'. The abundance profile for each metagenome was subsequently normalized by the metagenomic size (i.e. total number of base-pairs in each metagenome). This was done to even out the heterogeneity arising for differential metagenomic sampling for individual dataset. Finally, abundance of each family in each metagenome was expressed as number of significant BLAST hits per million base pairs of the metagenome.

If the gut microbiome of an individual is enriched with a large number of CAZymes, as the case may be for a highly saccharolytic microbiome, this would be reflected in the 'overall abundance'. To obtain the 'abundance' of CAZymes in a metagenomic sample, the number of hits for all the families in a metagenomic sample were cumulated and subsequently divided by the metagenomic size. On the other hand, if the gut microbiome of an individual contains a wide variety of CAZymes, as the case may be for highly diverse microbiome capable to acting on a variety of substrates, this would be reflected in the 'overall diversity'. To obtain the 'diversity' of a metagenomic sample, the total number of CAZyme families to which a hit was tagged in a metagenome was calculated. However, if the number of hits belonging to one particular family was less than 0.01% of the total number of hits, that family was not considered while calculating 'diversity'.

For quantifying the functional specialization of a microbiome, GINI coefficient, a measure widely used in econometrics for quantifying inequality in a system, was used [25–27]. In the present scenario, the entire CAZyme repertoire in a microbiome can be considered equivalent to the ‘economy of a country’. Thus, the measure of how equally or unequally the CAZyme families are represented in a microbiome can be thought of as analogous to the ‘income distribution across the population in an economy’. GINI coefficient is obtained from Lorenz curve, a cumulative frequency curve that compares the distribution of a specific variable (in the present case, the abundance of the CAZyme families) with the uniform distribution that represents equality. Details on how the GINI coefficient was computed are described in detail along with an example in S1 Text. While a GINI coefficient of 0 will indicate perfect equality, a coefficient of 1 will indicates a complete inequality of representation of the CAZyme families in the gut microbiome. Thus, a high GINI coefficient will represent a dysbiotic or highly specialized microbiome, which chiefly performs only specific carbohydrate degrading activity. On the other hand, a gut microbiome having a low GINI coefficient is expected to perform several diverse and generic functions.

To calculate if the mean of abundances/diversity/GINI coefficients is significantly different between the age/geography cohorts, ANOVA was performed (with P-value, corrected using Benjamini Hochberg method < 0.05) and post-hoc test (Tukey's HSD).

### Correlation between CAZyme profiles with age/BMI of individuals

In order to identify CAZyme families having significant correlations with age and BMI of individuals, the abundance profiles for each metagenome (normalized with respect to metagenomic size) was first obtained. The abundances computed for each CAZy family were then ranked across all the gut metagenomes. Post-processing of the data was then performed to remove sparsely abundant families, as these families might give rise to spurious correlations. CAZyme families present in least 50% of the population were used for further analysis.

The correlation coefficients (Pearsons) for each CAZyme family was then obtained using a linear regression model in R statistical package and those relations with P-value < 0.05 were initially considered as significant. Further, to correct for multiple testing, we used Benjamini-Hochberg FDR correction to adjust for the P-value, and then only those relations which still had a corrected P-value of less than 0.05 were considered as significant.

### Obtaining overall CAZyme profiles and BCA analysis

In order to obtain an overall view of how the CAZyme profile of one metagenome varies from another, two variants of Principal Component analysis (PCA), namely Partial Least Square Discriminant Analysis (PLS-DA) [28] and Between- Class Analysis (BCA) [19, 21], were used. Partial least squares regression (PLS regression) is similar to PCA, as both of them provide a way of visualizing high-dimensional data without much loss of information. However, as opposed to finding hyperplanes of minimum variance between the response and independent variables (in the case of PCA), PLS finds a linear regression model by projecting the predicted variables and the observable variables to a new space. PLS-DA follows the same principle as PLS regression, the only difference being Y-axis is categorical. BCA is a special form of PCA [29] which first performs pre-clustering of data points. The center of gravity of the obtained clusters is then used for computing the principal components. BCA is more robust to noise as compared to PCA and has an additional advantage of ascribing data points to particular clusters, rather than trying to cluster the data points as in case of other conventional methods. A detailed tutorial of the BCA approach as well as its application for the detection of Enterotypes, as used by Arumugam *et al*. [21], is available at http://enterotype.embl.de/enterotypes.html.

As both PLS-DA and BCA methods are subjected to relative scaling, two-levels of normalization of the abundance profile were performed. First, each of the database normalized values were filtered to remove the sparse entities (as described earlier). Subsequently the obtained values were then converted to Z-scores using the formula below:

Z–score=[(Database sized normalized abundance)–Mean]/Standard deviation

Further these Z-scores were range -scaled so that the values ranged between 0 and 1 using:

Range−scaled Z−score=[(Z−score)–(Min.Z−score)]/[(Max Z−score)−(Min.Z−score)]

### Statistical analyses to detect discriminating geography/CAZotype specific CAZymes

In order to investigate the subtle differences in the CAZyme profiles of adult individuals across different geographies, the geography specific CAZymes that were over-represented in the different cohorts were identified. The various cohorts were: 1) Asian (144 Chinese and 7 Japanese individuals), 2) American (90 US individuals), 3) European (8 French, 6 Italian, 81 Danish and 35 Spanish individuals) and 4) SouthAmerica-Africa (22 Malawi and 20 Venezuelan individuals). Similarly, CAZotype specific CAZyme were obtained by grouping all gut microbiomes having the same CAZotype affiliations. Subsequently, significantly over-represented CAZymes in each CAZotype with respect to others were identified using statistical tests implemented in the STAMP pipeline [30]. Welch t-tests were performed using STAMP to test if the differences of means were significant for CAZymes in one cohort as compared with all the other cohorts taken together. Further, the following tests were performed in order to ensure adequate stringency to avoid Type I and Type II errors:

- Test: One sided Welch 's T-test (does not assume equal variance)
- Method of determining confidence interval: Welch's inverted (95%)
- P-value (corrected, using Benjamini Hochberg FDR method) < 0.05
- Minimum ratio of proportion to call significant >1.5

### Obtaining taxonomic affiliations for CAZymes and calculating enrichment index

For each gut metagenome, the probable microorganisms harbouring various CAZyme families were identified using a strategy similar to that adopted by previous studies for accurate estimation of taxonomy from metagenomic sequences [19]. The taxonomic assignment of each CAZyme sequence was obtained based on the taxonomic origin of the hit in carbohydrate active enzymes database and percentage identity between the hit and the CAZyme in the database. The following thresholds were applied for obtaining appropriate level of taxonomic affiliation:

- Percentage identity > 85% ==> Assign to the Genus of the organism corresponding to the best hit
- Percentage identity 70–85% ==> Assign to the Family of the organism corresponding to the best hit
- Percentage identity 55–70% ==> Assign to the Class of the organism corresponding to the best hit
- Percentage identity 40–55% ==> Assign to the Phylum of the organism corresponding to the best hit

To calculate enrichment index, the ratio of representation of a taxa affiliated to the CAZymes to the representation of the taxa in the entire metagenome, was calculated. This was referred to as the 'fold enrichment'. The logarithm (base 10) of the fold enrichment was calculated as the ‘enrichment index’.

### Identifying the key drivers of the CAZotype

Once the individual samples were tagged to a particular CAZotype, the abundances of CAZyme affiliated taxa for all the individual samples belonging to a particular CAZotype were collated. Subsequently, the contribution of the taxa in a CAZotype was expressed as percentages and all the taxa contributing to greater than 0.1% of the CAZyme repertoire in a particular CAZotype was filtered for downstream processing. Differences among each contributing taxon was compared across the three CAZotypes using Welch’s t-test (one-sided) and the parameters for determining significance threshold were same as those used for determining specific CAZymes in a CAZotype.

## Supporting Information

S1 Fig Identification of Core and Non-core CAZymes in the human gut microbiome. A core CAZyme group is present in the human gut, which consists of 89 CAZymes that are present in at least 85% of the individuals studied.
(TIF)

S2 Fig Variation of (a) Abundance and, (b) Diversity of the CAZyme profiles with age in the gut microbiomes of Chinese individuals. Neither the abundance nor the diversity of the CAZymes show any correlation with the age of the individuals.
(TIF)

S3 Fig Variation of (a) Abundance and, (b) Diversity of the CAZyme profiles with age in the gut microbiomes of Spanish and Danish individuals. Neither the abundance nor the diversity of the CAZymes show any correlation with the age of the individuals.
(TIF)

S4 Fig Geography-specific CAZymes. **a, b, c and d** refer to geography-specific CAZymes belonging to Asia, Europe, North America and Africa (along with North America), respectively. The significant groups were identified using Welch’s T-test. P < 0.05 was used as cutoffs for identification after applying Benjamini-Hochberg FDR method for multiple test corrections. Further stringency was established using minimum ratio of mean proportions to be 1.5. All statistical analyses were performed using the STAMP package.
(TIF)

S5 Fig CAZotype-specific CAZymes. **a, b, and c** refer to geography-specific CAZymes belonging to CAZotypes 1, 2 and 3, respectively. The significant groups were identified using Welch’s T-test. P < 0.05 was used as cutoffs for identification after applying Benjamini-Hochberg FDR method for multiple test corrections. Further stringency was established using minimum ratio of mean proportions to be 1.5. All statistical analyses were performed using the STAMP package.
(TIF)

S1 Table List of microbe associated Core CAZymes in the human gut, along with the percentages of samples in which they were present. (PDF)

S2 Table Details of the CAZymes in the gut metagenomic data sets obtained from 448 metagenomes from 10 geographies. The numbers shown for CAZyme families, indicate the number of contigs (in a given sample) that had the given CAZyme profile, normalized by the metagenomic size.
(PDF)

S3 Table List of all GH and PL CAZyme families that were found to over/underrepresentated in all pairwise comparisons of countries. (XLSX)

S4 Table Details of metagenomic datasets used. (XLS)

S5 Table CAZotype affiliation of 448 metagenomic sample. (XLS)

S1 Text Calculation of Gini coefficient from the CAZyme profile of a metagenome. (PDF)

S2 Text Investigation of the CAZotype predictive ability of the CAZyme and taxonomic profiles using Partial Least Square (PLS) Regression. (PDF)
