# Supplementary figures and images for: Global Profiling of Carbohydrate Active Enzymes in Human Gut Microbiome
Source: PLoS One. 2015 Nov 6;10(11):e0142038. doi: 10.1371/journal.pone.0142038 (PMC4636310; doi:10.1371/journal.pone.0142038)

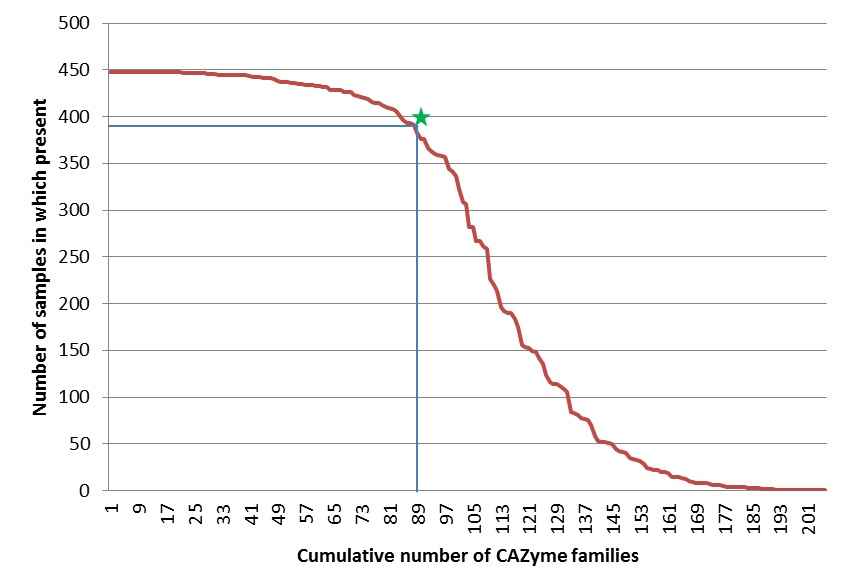

Supplement: S1 Fig — A core CAZyme group is present in the human gut, which consists of 89 CAZymes that are present in at least 85% of the individuals studied. (TIF) [file pone.0142038.s001.tif]

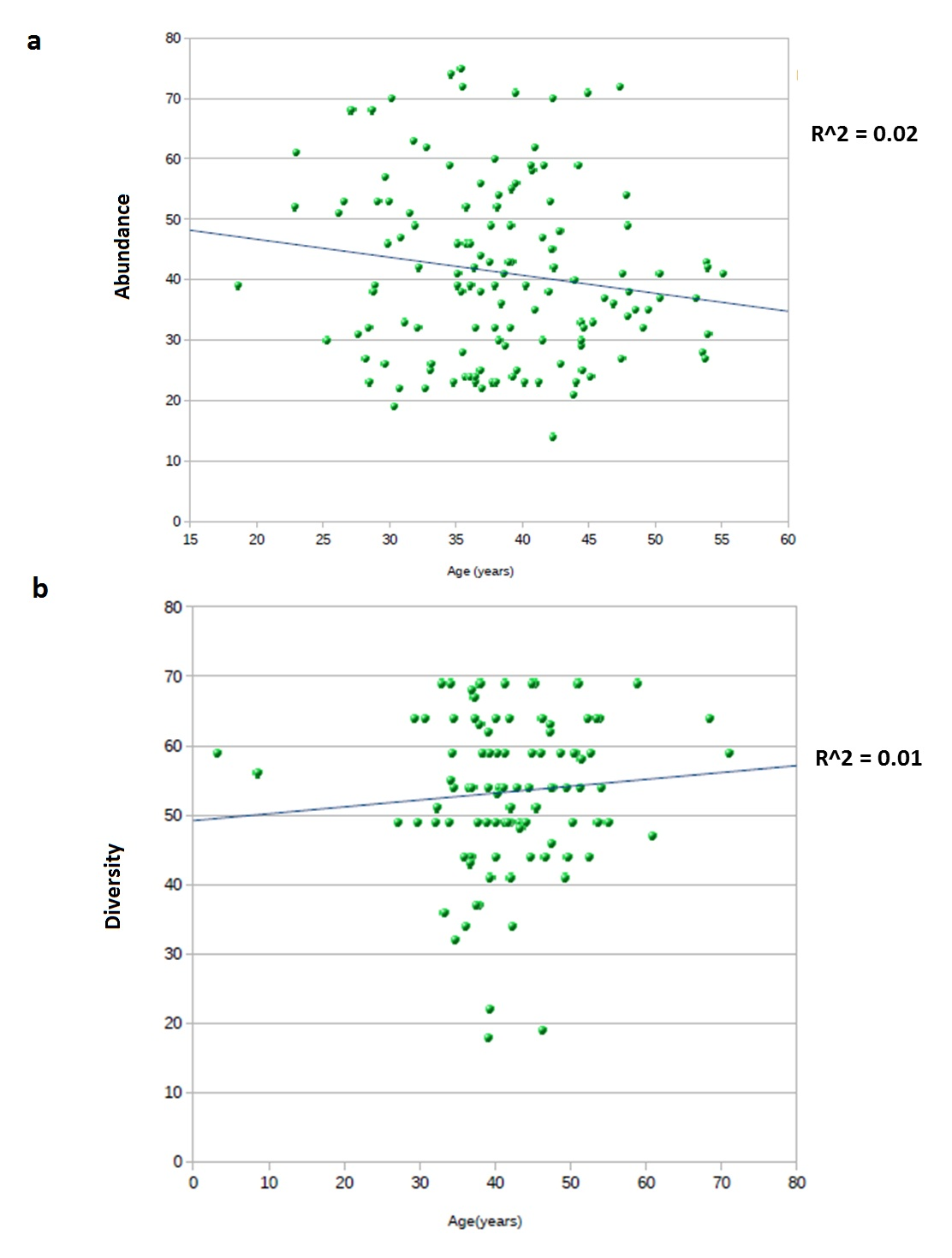

Supplement: S2 Fig — Neither the abundance nor the diversity of the CAZymes show any correlation with the age of the individuals. (TIF) [file pone.0142038.s002.tif]

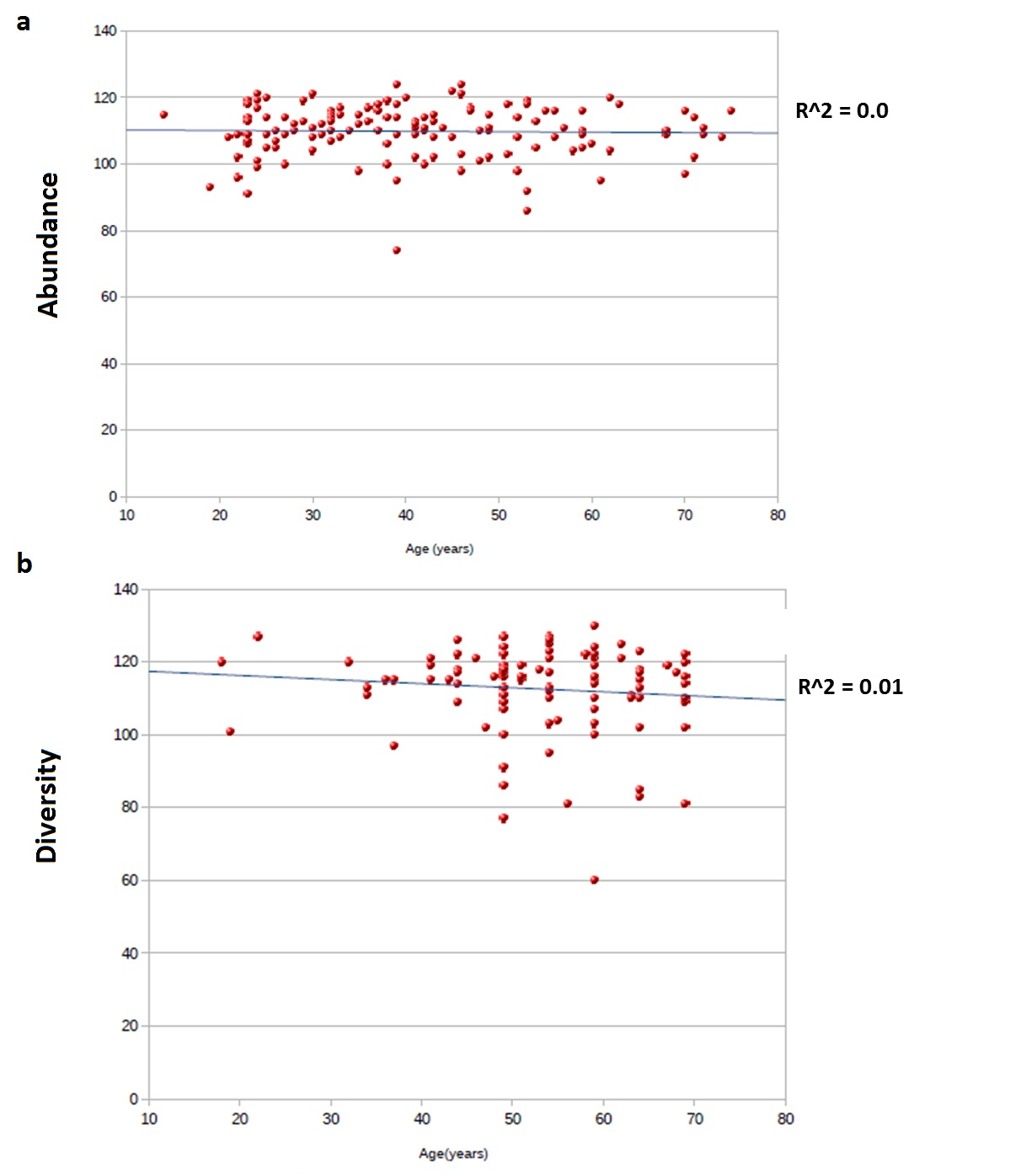

Supplement: S3 Fig — Neither the abundance nor the diversity of the CAZymes show any correlation with the age of the individuals. (TIF) [file pone.0142038.s003.tif]

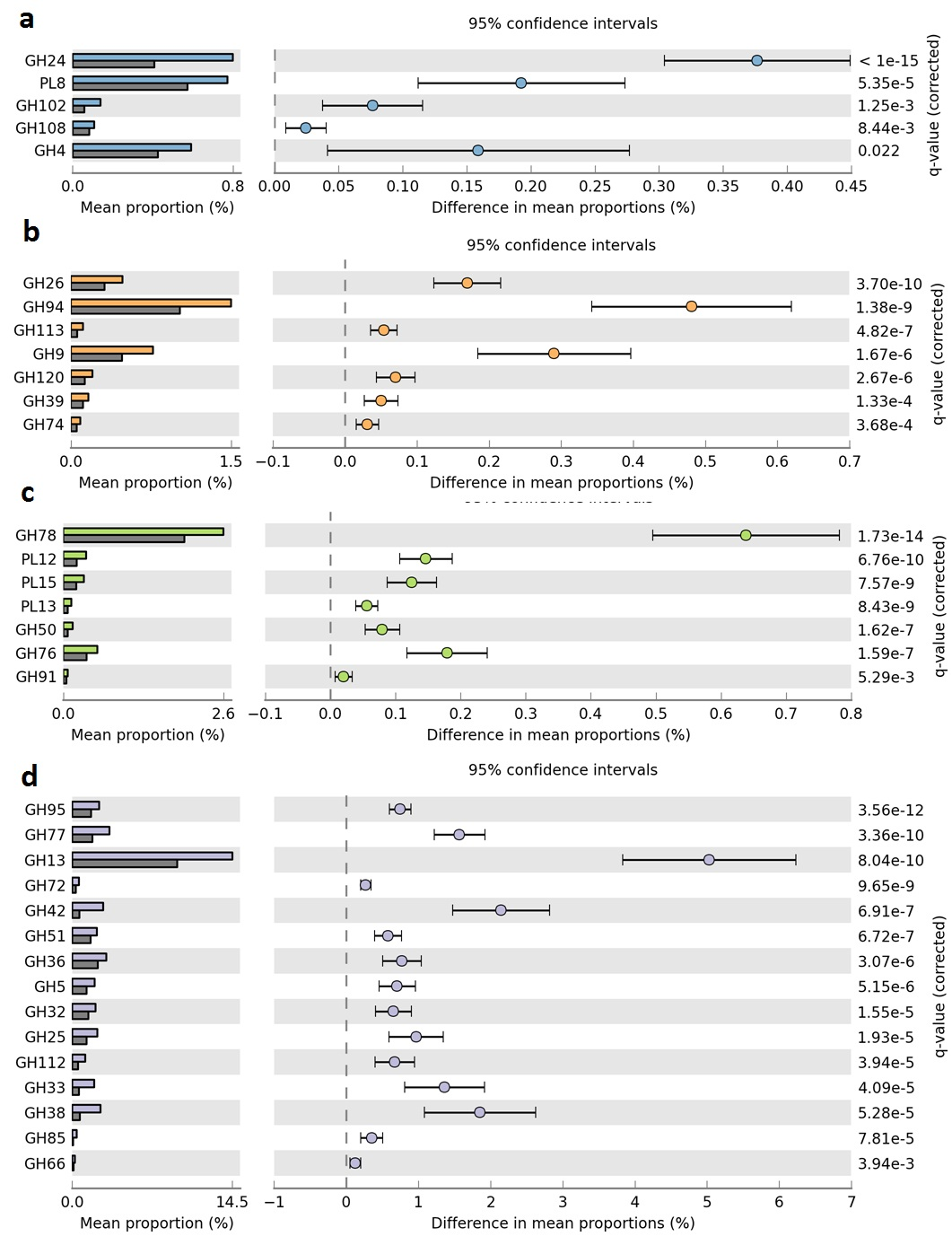

Supplement: S4 Fig — a, b, c and d refer to geography-specific CAZymes belonging to Asia, Europe, North America and Africa (along with North America), respectively. The significant groups were identified using Welch’s T-test. P < 0.05 was used as cutoffs for identification after applying Benjamini-Hochberg FDR method for multiple test corrections. Further stringency was established using minimum ratio of mean proportions to be 1.5. All statistical analyses were performed using the STAMP package. (TIF) [file pone.0142038.s004.tif]

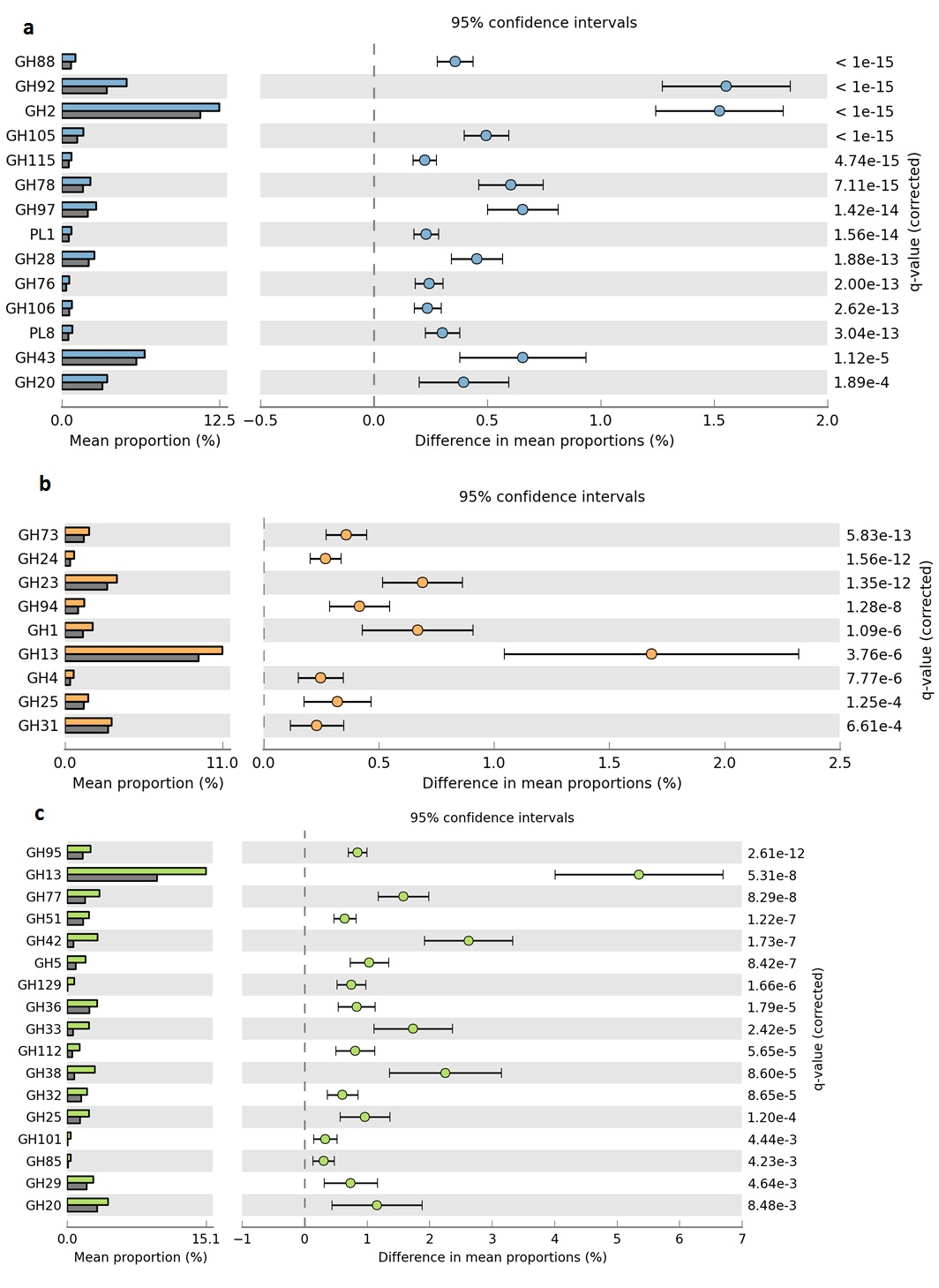

Supplement: S5 Fig — a, b, and c refer to geography-specific CAZymes belonging to CAZotypes 1, 2 and 3, respectively. The significant groups were identified using Welch’s T-test. P < 0.05 was used as cutoffs for identification after applying Benjamini-Hochberg FDR method for multiple test corrections. Further stringency was established using minimum ratio of mean proportions to be 1.5. All statistical analyses were performed using the STAMP package. (TIF) [file pone.0142038.s005.tif]
