# Supplementary material for: Global Profiling of Carbohydrate Active Enzymes in Human Gut Microbiome
Source: PLoS One. 2015 Nov 6;10(11):e0142038. doi: 10.1371/journal.pone.0142038 (PMC4636310; doi:10.1371/journal.pone.0142038)
Supplement: S1 Table — (PDF) [file pone.0142038.s006.pdf]

CAZyme_family	Presence_percentage_in samples_analyzed
GH29	100.00
GH23	100.00
GT4	100.00
GH36	100.00
GH2	100.00
GT28	100.00
GH28	100.00
GH73	100.00
GT5	100.00
GH18	100.00
GH3	100.00
GH31	100.00
GH77	100.00
GH43	100.00
GH51	100.00
GT35	100.00
GT2	100.00
GT51	100.00
GH32	100.00
GH13	100.00
CE4	100.00
GH5	99.78
CE1	99.78
CE9	99.78
GH20	99.78
GH25	99.78
GH35	99.78
GT30	99.78
GH92	99.55
GH95	99.55
CE10	99.55
GH1	99.33
GT26	99.33
GH127	99.33
GH112	99.33
CE11	99.33
GH72	99.11
GH24	99.11
GT9	99.11
CE8	99.11
GT19	98.88
GH78	98.66
GH33	98.66
GH125	98.66
GH38	98.44
GT8	98.44
GH30	98.44
GH42	98.21
GH16	97.77
GH105	97.54
GH63	97.54
GH65	97.54
GH94	97.32
GH84	97.32

GT83	97.10
GH89	97.10
GH109	96.88
GH27	96.88
GH4	96.88
GH97	96.65
GH88	96.65
GH53	96.43
CE7	96.43
GH106	95.76
PL1	95.76
GH115	95.76
GH130	95.54
GH57	95.09
CE12	95.09
GT32	95.09
GT3	94.42
GH110	94.20
GH10	93.97
GH9	93.75
PL10	93.53
GH26	92.86
PL11	92.63
GH67	92.63
PL9	91.96
GH123	91.52
GH8	91.29
PL8	91.07
GT11	90.63
CE2	89.51
GT1	88.39
GT47	87.72
GH117	87.72
GT20	87.28
GH66	85.27
