# Supplementary material for: Global Profiling of Carbohydrate Active Enzymes in Human Gut Microbiome
Source: PLoS One. 2015 Nov 6;10(11):e0142038. doi: 10.1371/journal.pone.0142038 (PMC4636310; doi:10.1371/journal.pone.0142038)
Supplement: S2 Table — The numbers shown for CAZyme families, indicate the number of contigs (in a given sample) that had the given CAZyme profile, normalized by the metagenomic size. (PDF) [file pone.0142038.s007.pdf]

CAZY_group	IN-CH-1	IN-CH-2	IN-CH-3	IN-CH-4	IN-CH-5	IN-CH-6	IN-CH-7	IN-CH-8	IN-CH-9	IN-IN-1	IN-CH-10	IN-CH-11	AM-AD-10	AM-AD-11	AM-AD-12	AM-AD-13	AM-AD-14	AM-AD-15	AM-AD-16	AM-AD-17
GH29	0.60	0.58	0.53	0.55	0.51	0.39	0.43	0.30	0.29	0.40	0.14	0.48	0.67	0.86	0.11	0.50	0.67	0.61	0.53	0.40
PL4	0.00	0.00	0.00	0.00	0.00	0.00	0.00	0.00	0.00	0.00	0.00	0.00	0.00	0.00	0.00	0.00	0.01	0.00	0.00	0.00
GH23	0.45	0.86	0.61	1.31	0.62	0.45	0.59	1.07	0.80	0.64	0.99	0.98	1.20	1.42	0.49	1.18	1.20	1.38	1.18	1.14
CE6	0.30	0.16	0.26	0.18	0.11	0.21	0.21	0.12	0.16	0.21	0.00	0.15	0.01	0.05	0.00	0.00	0.04	0.04	0.00	0.00
GH72	0.04	0.19	0.05	0.11	0.06	0.13	0.07	0.18	0.11	0.06	0.14	0.13	0.21	0.10	0.02	0.13	0.17	0.14	0.15	0.08
GH114	0.00	0.00	0.00	0.00	0.00	0.00	0.00	0.00	0.00	0.00	0.00	0.00	0.00	0.00	0.00	0.01	0.00	0.00	0.00	0.00
GH78	0.13	0.23	0.13	0.44	0.06	0.11	0.25	0.09	0.16	0.12	0.00	0.28	0.67	1.06	0.18	0.41	0.96	1.00	0.61	0.62
PL19	0.00	0.00	0.00	0.00	0.00	0.00	0.00	0.00	0.00	0.00	0.00	0.00	0.00	0.00	0.00	0.00	0.00	0.00	0.00	0.00
GT27	0.00	0.00	0.00	0.00	0.00	0.00	0.00	0.00	0.00	0.00	0.07	0.00	0.00	0.00	0.00	0.00	0.00	0.00	0.00	0.00
GH5	0.69	0.49	0.59	0.42	0.28	0.69	0.63	0.33	0.44	0.48	0.28	0.49	0.60	0.45	0.08	0.39	0.59	0.44	0.28	0.50
GT40	0.00	0.00	0.00	0.00	0.00	0.00	0.00	0.00	0.00	0.00	0.00	0.00	0.00	0.00	0.00	0.00	0.00	0.00	0.00	0.00
CE13	0.00	0.00	0.00	0.00	0.00	0.00	0.00	0.00	0.00	0.00	0.00	0.00	0.00	0.00	0.00	0.00	0.01	0.00	0.00	0.00
GT37	0.00	0.00	0.00	0.00	0.00	0.00	0.00	0.00	0.00	0.00	0.00	0.00	0.00	0.00	0.00	0.00	0.00	0.00	0.00	0.00
GH33	0.00	0.05	0.05	0.11	0.06	0.00	0.01	0.06	0.07	0.04	0.35	0.00	0.24	0.26	0.06	0.21	0.19	0.24	0.22	0.11
GT65	0.00	0.00	0.00	0.00	0.00	0.00	0.00	0.00	0.00	0.00	0.00	0.00	0.00	0.00	0.00	0.00	0.00	0.00	0.00	0.00
GH86	0.00	0.00	0.00	0.00	0.00	0.00	0.00	0.00	0.00	0.00	0.00	0.00	0.00	0.00	0.00	0.01	0.01	0.00	0.00	0.00
GH123	0.09	0.05	0.10	0.07	0.00	0.06	0.04	0.09	0.07	0.06	0.00	0.08	0.10	0.13	0.02	0.10	0.17	0.17	0.09	0.04
GH96	0.00	0.00	0.00	0.00	0.00	0.00	0.00	0.00	0.00	0.00	0.00	0.00	0.00	0.00	0.00	0.00	0.00	0.00	0.00	0.00
GH14	0.00	0.00	0.00	0.00	0.00	0.00	0.00	0.00	0.00	0.00	0.00	0.00	0.00	0.00	0.00	0.00	0.00	0.00	0.00	0.00
CE3	0.00	0.00	0.00	0.00	0.00	0.00	0.01	0.00	0.00	0.00	0.00	0.00	0.01	0.00	0.00	0.00	0.01	0.00	0.00	0.00
PL10	0.17	0.04	0.19	0.24	0.11	0.28	0.24	0.09	0.16	0.27	0.00	0.18	0.04	0.13	0.03	0.02	0.09	0.05	0.07	0.07
GT48	0.00	0.00	0.00	0.00	0.00	0.00	0.00	0.00	0.00	0.00	0.00	0.00	0.00	0.00	0.00	0.00	0.00	0.00	0.00	0.00
PL6	0.00	0.00	0.00	0.00	0.00	0.00	0.00	0.00	0.00	0.00	0.00	0.00	0.00	0.00	0.00	0.00	0.00	0.00	0.01	0.00
GT83	0.11	0.12	0.26	0.15	0.11	0.06	0.10	0.24	0.05	0.23	0.21	0.18	0.09	0.08	0.02	0.07	0.10	0.07	0.08	0.05
GH126	0.00	0.00	0.00	0.00	0.00	0.00	0.00	0.00	0.00	0.00	0.00	0.00	0.00	0.00	0.00	0.00	0.00	0.00	0.00	0.00
GH9	0.06	0.04	0.03	0.11	0.00	0.02	0.03	0.03	0.07	0.12	0.00	0.16	0.31	0.15	0.05	0.20	0.12	0.13	0.14	0.39
GH75	0.00	0.00	0.00	0.00	0.00	0.00	0.00	0.00	0.00	0.00	0.00	0.00	0.00	0.00	0.00	0.00	0.00	0.00	0.00	0.00
GT71	0.00	0.00	0.00	0.00	0.00	0.00	0.00	0.00	0.00	0.00	0.00	0.00	0.00	0.00	0.00	0.00	0.00	0.00	0.00	0.00
GH46	0.00	0.00	0.00	0.00	0.00	0.00	0.00	0.00	0.00	0.00	0.00	0.00	0.01	0.00	0.00	0.00	0.00	0.00	0.00	0.00
GT61	0.00	0.00	0.00	0.00	0.00	0.00	0.00	0.00	0.00	0.00	0.00	0.00	0.00	0.00	0.00	0.00	0.00	0.00	0.00	0.00
GT3	0.32	0.18	0.14	0.35	0.06	0.24	0.18	0.18	0.09	0.19	0.00	0.28	0.27	0.16	0.03	0.13	0.24	0.30	0.12	0.12
PL3	0.00	0.00	0.00	0.00	0.00	0.00	0.00	0.00	0.00	0.00	0.00	0.00	0.00	0.00	0.00	0.00	0.00	0.00	0.00	0.00
GH108	0.04	0.00	0.02	0.00	0.00	0.02	0.01	0.00	0.00	0.04	0.00	0.00	0.01	0.05	0.01	0.04	0.03	0.04	0.03	0.07
GT56	0.00	0.00	0.00	0.00	0.00	0.00	0.01	0.12	0.00	0.00	0.14	0.03	0.00	0.00	0.00	0.00	0.00	0.00	0.00	0.00
PL11	0.00	0.02	0.02	0.02	0.00	0.07	0.03	0.03	0.04	0.00	0.00	0.07	0.06	0.21	0.01	0.03	0.23	0.15	0.11	0.13
GH45	0.00	0.00	0.00	0.00	0.00	0.00	0.00	0.00	0.00	0.00	0.00	0.00	0.00	0.00	0.00	0.00	0.01	0.00	0.00	0.00
GT34	0.00	0.00	0.00	0.00	0.00	0.00	0.00	0.00	0.00	0.00	0.00	0.02	0.01	0.00	0.00	0.00	0.01	0.00	0.00	0.00
PL14	0.00	0.00	0.00	0.00	0.00	0.00	0.00	0.00	0.00	0.00	0.00	0.00	0.00	0.00	0.00	0.00	0.00	0.00	0.00	0.00
GH19	0.00	0.00	0.00	0.00	0.00	0.00	0.01	0.00	0.02	0.00	0.00	0.03	0.01	0.01	0.00	0.00	0.01	0.01	0.01	0.00
GH118	0.00	0.00	0.00	0.00	0.00	0.00	0.00	0.00	0.00	0.00	0.00	0.00	0.00	0.00	0.00	0.00	0.00	0.00	0.00	0.00
GH82	0.00	0.00	0.00	0.00	0.00	0.00	0.00	0.00	0.00	0.00	0.00	0.00	0.00	0.00	0.00	0.00	0.00	0.00	0.00	0.00
GH76	0.00	0.00	0.00	0.00	0.00	0.00	0.00	0.00	0.00	0.00	0.00	0.02	0.14	0.20	0.03	0.13	0.13	0.13	0.12	0.12
GT4	1.62	1.51	1.47	1.51	1.96	1.51	1.25	1.58	1.81	1.48	0.57	1.44	2.07	2.50	0.48	1.46	2.26	2.32	2.15	1.67
GT87	0.00	0.00	0.00	0.00	0.00	0.00	0.00	0.03	0.00	0.02	0.00	0.00	0.01	0.00	0.00	0.00	0.00	0.01	0.01	0.00
GH24	0.41	0.30	0.35	0.35	0.39	0.49	0.17	0.27	0.24	0.37	0.28	0.25	0.15	0.10	0.04	0.14	0.08	0.08	0.10	0.05
GH90	0.00	0.00	0.00	0.00	0.00	0.00	0.00	0.00	0.00	0.00	0.00	0.00	0.00	0.00	0.00	0.00	0.00	0.00	0.00	0.00
GH57	0.15	0.14	0.14	0.35	0.00	0.13	0.10	0.06	0.29	0.25	0.00	0.15	0.28	0.19	0.02	0.11	0.14	0.14	0.11	0.08
GH36	1.08	0.93	0.75	0.80	1.52	0.78	0.92	1.07	0.97	0.89	0.28	0.90	0.87	0.88	0.18	0.56	0.95	0.84	0.69	0.51
GT46	0.00	0.00	0.00	0.00	0.00	0.00	0.00	0.00	0.00	0.00	0.00	0.00	0.00	0.00	0.00	0.00	0.00	0.00	0.00	0.00
GH66	0.13	0.07	0.03	0.04	0.11	0.15	0.03	0.12	0.15	0.10	0.00	0.05	0.03	0.06	0.01	0.02	0.03	0.03	0.02	0.01

CAZY_group	IN-CH-1	IN-CH-2	IN-CH-3	IN-CH-4	IN-CH-5	IN-CH-6	IN-CH-7	IN-CH-8	IN-CH-9	IN-IN-1	IN-CH-10	IN-CH-11	AM-AD-10	AM-AD-11	AM-AD-12	AM-AD-13	AM-AD-14	AM-AD-15	AM-AD-16	AM-AD-17
**GH48**	0.00	0.00	0.00	0.00	0.00	0.00	0.00	0.00	0.00	0.00	0.00	0.02	0.00	0.01	0.00	0.02	0.01	0.01	0.00	0.03
**GT53**	0.00	0.00	0.00	0.00	0.00	0.00	0.00	0.00	0.00	0.00	0.00	0.00	0.00	0.00	0.00	0.00	0.00	0.00	0.00	0.00
**GH60**	0.00	0.00	0.00	0.00	0.00	0.00	0.00	0.00	0.00	0.00	0.00	0.00	0.00	0.00	0.00	0.00	0.00	0.00	0.00	0.00
**GT31**	0.00	0.00	0.00	0.00	0.00	0.00	0.00	0.03	0.00	0.00	0.00	0.02	0.00	0.00	0.00	0.00	0.00	0.00	0.00	0.00
**GH105**	0.22	0.26	0.16	0.18	0.17	0.24	0.28	0.06	0.24	0.10	0.00	0.31	0.48	0.87	0.09	0.20	0.48	0.48	0.51	0.29
**GT23**	0.00	0.00	0.00	0.00	0.00	0.00	0.00	0.00	0.00	0.00	0.00	0.00	0.00	0.02	0.00	0.00	0.00	0.00	0.01	0.01
**GH11**	0.00	0.00	0.00	0.00	0.00	0.00	0.00	0.00	0.00	0.00	0.00	0.00	0.01	0.01	0.00	0.00	0.01	0.00	0.01	0.01
**GT38**	0.00	0.00	0.00	0.00	0.00	0.00	0.00	0.00	0.00	0.00	0.00	0.00	0.00	0.00	0.00	0.00	0.00	0.00	0.00	0.00
**GT43**	0.00	0.00	0.00	0.00	0.00	0.00	0.00	0.00	0.00	0.00	0.00	0.00	0.00	0.00	0.00	0.00	0.00	0.00	0.00	0.00
**GH99**	0.00	0.00	0.00	0.00	0.00	0.04	0.00	0.00	0.00	0.02	0.00	0.00	0.01	0.02	0.01	0.00	0.01	0.00	0.01	0.01
**GH38**	0.00	0.05	0.02	0.11	0.00	0.04	0.04	0.00	0.04	0.00	0.43	0.11	0.24	0.31	0.08	0.22	0.24	0.31	0.25	0.19
**GH109**	0.32	0.26	0.26	0.22	0.11	0.11	0.17	0.09	0.20	0.19	0.00	0.21	0.24	0.34	0.03	0.17	0.23	0.24	0.27	0.23
**GH2**	2.74	2.80	2.97	2.90	3.20	2.88	2.65	2.56	2.28	2.58	0.71	3.11	4.42	5.28	1.04	2.97	4.25	4.22	3.55	2.62
**GT28**	0.50	0.44	0.37	0.60	0.67	0.28	0.27	0.45	0.49	0.37	0.50	0.48	0.41	0.52	0.15	0.38	0.51	0.50	0.42	0.29
**GH42**	0.06	0.09	0.03	0.09	0.06	0.02	0.06	0.06	0.07	0.00	0.50	0.08	0.21	0.32	0.06	0.17	0.40	0.32	0.10	0.20
**GT58**	0.00	0.00	0.00	0.00	0.00	0.00	0.00	0.00	0.00	0.00	0.00	0.00	0.00	0.00	0.00	0.00	0.00	0.00	0.00	0.01
**GH61**	0.00	0.00	0.00	0.00	0.00	0.00	0.00	0.00	0.00	0.00	0.00	0.00	0.00	0.00	0.00	0.00	0.00	0.00	0.00	0.00
**GH80**	0.00	0.00	0.00	0.00	0.00	0.00	0.00	0.00	0.00	0.00	0.00	0.00	0.00	0.00	0.00	0.00	0.00	0.00	0.00	0.00
**GH98**	0.00	0.00	0.00	0.02	0.00	0.00	0.00	0.00	0.00	0.00	0.00	0.00	0.02	0.00	0.01	0.00	0.00	0.00	0.00	0.02
**GH6**	0.00	0.00	0.00	0.00	0.00	0.00	0.00	0.00	0.00	0.00	0.00	0.00	0.00	0.00	0.00	0.00	0.01	0.01	0.00	0.00
**GT1**	0.06	0.07	0.00	0.02	0.00	0.02	0.01	0.24	0.00	0.04	0.07	0.07	0.02	0.05	0.02	0.02	0.03	0.04	0.05	0.05
**GH28**	0.71	0.65	0.72	0.64	0.51	0.88	0.80	0.48	0.71	0.75	0.07	0.84	0.74	1.17	0.21	0.20	0.84	0.75	0.66	0.38
**GT42**	0.00	0.00	0.00	0.00	0.00	0.02	0.00	0.00	0.00	0.00	0.00	0.00	0.00	0.00	0.00	0.00	0.00	0.00	0.00	0.00
**GH128**	0.00	0.00	0.00	0.00	0.00	0.00	0.00	0.00	0.00	0.00	0.00	0.00	0.00	0.00	0.00	0.00	0.00	0.00	0.00	0.00
**CE12**	0.24	0.30	0.16	0.20	0.39	0.22	0.28	0.15	0.20	0.23	0.00	0.20	0.13	0.19	0.03	0.05	0.13	0.17	0.09	0.11
**CE16**	0.00	0.00	0.00	0.00	0.00	0.00	0.00	0.00	0.00	0.00	0.00	0.00	0.00	0.00	0.00	0.00	0.00	0.00	0.00	0.00
**GT89**	0.00	0.00	0.00	0.00	0.00	0.00	0.00	0.00	0.00	0.00	0.00	0.00	0.00	0.00	0.00	0.00	0.00	0.00	0.00	0.00
**GT11**	0.04	0.11	0.02	0.07	0.06	0.04	0.04	0.00	0.02	0.02	0.00	0.05	0.03	0.06	0.01	0.04	0.05	0.03	0.05	0.05
**GT77**	0.02	0.00	0.00	0.00	0.06	0.02	1.08	0.15	0.04	0.00	0.64	0.16	0.01	0.00	0.00	0.00	0.00	0.00	0.00	0.00
**GT55**	0.00	0.00	0.00	0.00	0.00	0.00	0.00	0.00	0.00	0.00	0.00	0.00	0.00	0.00	0.00	0.00	0.00	0.00	0.00	0.00
**GT18**	0.00	0.00	0.00	0.00	0.00	0.00	0.00	0.00	0.00	0.00	0.00	0.00	0.00	0.00	0.00	0.00	0.00	0.00	0.00	0.00
**GH87**	0.04	0.00	0.00	0.00	0.00	0.02	0.00	0.00	0.07	0.02	0.00	0.00	0.01	0.00	0.00	0.00	0.00	0.01	0.01	0.00
**PL5**	0.00	0.00	0.00	0.00	0.00	0.00	0.00	0.00	0.00	0.00	0.00	0.00	0.00	0.00	0.00	0.00	0.00	0.00	0.00	0.00
**GH22**	0.00	0.00	0.00	0.00	0.00	0.00	0.00	0.00	0.00	0.00	0.00	0.00	0.00	0.00	0.00	0.00	0.00	0.00	0.00	0.00
**CE2**	0.09	0.07	0.05	0.04	0.00	0.06	0.04	0.03	0.09	0.04	0.00	0.05	0.09	0.04	0.02	0.04	0.13	0.07	0.07	0.04
**GH120**	0.00	0.00	0.00	0.00	0.00	0.06	0.00	0.00	0.02	0.00	0.00	0.03	0.10	0.03	0.01	0.05	0.05	0.09	0.01	0.02
**GH73**	0.56	0.56	0.38	0.31	0.62	0.47	0.49	0.83	0.77	0.79	0.07	0.41	0.55	0.66	0.14	0.39	0.63	0.69	0.56	0.53
**GT17**	0.00	0.00	0.00	0.00	0.00	0.00	0.00	0.00	0.00	0.00	0.00	0.00	0.02	0.00	0.01	0.00	0.01	0.00	0.00	0.00
**GH124**	0.00	0.00	0.00	0.00	0.00	0.00	0.00	0.00	0.00	0.00	0.00	0.00	0.00	0.00	0.00	0.00	0.00	0.00	0.00	0.00
**GH54**	0.00	0.00	0.00	0.00	0.00	0.00	0.00	0.00	0.00	0.00	0.00	0.00	0.00	0.00	0.00	0.00	0.01	0.00	0.00	0.00
**GH100**	0.00	0.00	0.00	0.00	0.00	0.00	0.00	0.00	0.00	0.00	0.00	0.00	0.00	0.00	0.00	0.00	0.00	0.00	0.00	0.00
**GH113**	0.00	0.07	0.00	0.00	0.00	0.02	0.01	0.00	0.00	0.00	0.00	0.03	0.05	0.02	0.01	0.04	0.08	0.02	0.03	0.00
**GT5**	0.35	0.48	0.37	0.49	0.28	0.32	0.32	0.21	0.36	0.25	0.21	0.38	0.53	0.54	0.15	0.47	0.59	0.63	0.49	0.41
**GH94**	0.47	0.39	0.40	0.31	0.22	0.32	0.38	0.33	0.36	0.27	0.00	0.41	0.67	0.38	0.19	0.54	0.75	0.44	0.49	0.27
**GT66**	0.02	0.00	0.00	0.00	0.00	0.00	0.00	0.00	0.02	0.02	0.00	0.02	0.00	0.00	0.00	0.00	0.01	0.01	0.01	0.00
**GT94**	0.00	0.00	0.00	0.00	0.00	0.00	0.00	0.00	0.00	0.00	0.00	0.00	0.00	0.00	0.00	0.00	0.00	0.00	0.00	0.00
**GH58**	0.00	0.00	0.00	0.00	0.00	0.00	0.00	0.06	0.00	0.00	0.00	0.00	0.00	0.00	0.00	0.00	0.00	0.00	0.00	0.00
**GH1**	0.09	0.28	0.06	0.09	0.39	0.17	0.15	2.27	1.70	0.02	0.28	0.28	0.40	0.72	0.18	0.50	0.66	0.46	0.58	0.30
**PL22**	0.00	0.00	0.00	0.00	0.00	0.00	0.00	0.00	0.00	0.00	0.00	0.00	0.00	0.00	0.00	0.00	0.00	0.00	0.00	0.00

CAZY_group	IN-CH-1	IN-CH-2	IN-CH-3	IN-CH-4	IN-CH-5	IN-CH-6	IN-CH-7	IN-CH-8	IN-CH-9	IN-IN-1	IN-CH-10	IN-CH-11	AM-AD-10	AM-AD-11	AM-AD-12	AM-AD-13	AM-AD-14	AM-AD-15	AM-AD-16	AM-AD-17
**GT76**	0.00	0.00	0.00	0.00	0.00	0.00	0.00	0.00	0.00	0.00	0.00	0.00	0.00	0.00	0.00	0.00	0.01	0.00	0.00	0.00
**GT32**	0.09	0.09	0.03	0.04	0.00	0.07	0.04	0.00	0.07	0.02	0.00	0.02	0.12	0.11	0.02	0.06	0.09	0.15	0.08	0.04
**GT47**	0.17	0.11	0.03	0.07	0.00	0.04	0.03	0.15	0.07	0.15	0.07	0.03	0.06	0.09	0.02	0.06	0.07	0.07	0.08	0.06
**GH84**	0.19	0.18	0.19	0.18	0.06	0.07	0.14	0.18	0.16	0.25	0.07	0.10	0.11	0.11	0.03	0.08	0.13	0.07	0.11	0.06
**GH83**	0.00	0.00	0.00	0.00	0.00	0.00	0.00	0.00	0.00	0.00	0.00	0.00	0.00	0.00	0.00	0.00	0.00	0.00	0.00	0.00
**GT59**	0.00	0.00	0.00	0.00	0.00	0.00	0.00	0.00	0.00	0.00	0.00	0.00	0.00	0.00	0.00	0.00	0.00	0.00	0.00	0.01
**GH34**	0.00	0.00	0.00	0.00	0.00	0.00	0.00	0.00	0.00	0.00	0.00	0.00	0.00	0.00	0.00	0.00	0.00	0.00	0.00	0.00
**GT14**	0.04	0.00	0.02	0.02	0.06	0.06	0.01	0.09	0.09	0.04	0.00	0.05	0.02	0.02	0.01	0.01	0.02	0.02	0.04	0.02
**GH47**	0.00	0.00	0.00	0.00	0.00	0.00	0.00	0.00	0.00	0.00	0.00	0.00	0.00	0.00	0.00	0.00	0.01	0.01	0.00	0.03
**GH97**	1.17	0.86	1.17	0.93	0.84	1.34	1.12	0.86	0.93	1.12	0.00	0.97	0.71	0.93	0.07	0.53	0.68	0.82	0.61	0.58
**GH50**	0.00	0.00	0.00	0.00	0.00	0.00	0.00	0.00	0.00	0.02	0.00	0.02	0.01	0.06	0.00	0.00	0.05	0.09	0.01	0.02
**GT26**	0.15	0.21	0.24	0.31	0.11	0.32	0.17	0.24	0.24	0.12	0.00	0.18	0.21	0.17	0.06	0.19	0.21	0.20	0.19	0.13
**GH18**	0.19	0.37	0.19	0.33	0.39	0.09	0.08	0.27	0.15	0.12	0.35	0.36	0.75	0.85	0.15	0.53	0.79	0.72	0.64	0.53
**GH37**	0.00	0.00	0.00	0.00	0.00	0.00	0.03	0.06	0.00	0.00	0.07	0.02	0.01	0.00	0.00	0.00	0.02	0.01	0.00	0.00
**PL13**	0.00	0.00	0.00	0.00	0.00	0.00	0.00	0.00	0.00	0.00	0.00	0.00	0.02	0.04	0.01	0.02	0.03	0.03	0.01	0.00
**GH27**	0.32	0.23	0.14	0.22	0.11	0.17	0.15	0.15	0.16	0.21	0.00	0.21	0.41	0.31	0.05	0.23	0.27	0.29	0.20	0.16
**GT54**	0.00	0.00	0.00	0.00	0.00	0.00	0.00	0.00	0.00	0.00	0.00	0.00	0.00	0.00	0.00	0.00	0.00	0.00	0.00	0.00
**GT91**	0.00	0.00	0.00	0.00	0.00	0.00	0.00	0.00	0.00	0.00	0.00	0.00	0.00	0.00	0.00	0.00	0.00	0.00	0.00	0.00
**GT72**	0.00	0.00	0.00	0.00	0.00	0.00	0.00	0.00	0.00	0.00	0.00	0.00	0.00	0.00	0.00	0.00	0.00	0.00	0.00	0.00
**GH67**	0.15	0.09	0.08	0.11	0.17	0.11	0.13	0.09	0.15	0.15	0.00	0.08	0.06	0.06	0.00	0.04	0.04	0.06	0.03	0.07
**GH12**	0.00	0.00	0.00	0.00	0.00	0.00	0.00	0.00	0.02	0.00	0.00	0.00	0.00	0.00	0.00	0.00	0.00	0.00	0.00	0.00
**GH91**	0.00	0.00	0.00	0.00	0.00	0.00	0.00	0.00	0.02	0.00	0.00	0.00	0.04	0.06	0.00	0.01	0.03	0.03	0.03	0.01
**GT69**	0.00	0.00	0.00	0.00	0.00	0.00	0.00	0.00	0.00	0.00	0.00	0.00	0.00	0.00	0.00	0.00	0.00	0.00	0.00	0.00
**GH106**	0.13	0.21	0.16	0.29	0.17	0.21	0.21	0.12	0.13	0.19	0.00	0.33	0.12	0.35	0.05	0.08	0.25	0.18	0.21	0.19
**GT62**	0.00	0.00	0.00	0.00	0.00	0.00	0.00	0.00	0.00	0.00	0.00	0.00	0.00	0.00	0.00	0.00	0.00	0.00	0.00	0.00
**GH17**	0.00	0.00	0.00	0.00	0.00	0.00	0.00	0.00	0.00	0.00	0.00	0.00	0.00	0.00	0.00	0.00	0.01	0.00	0.00	0.01
**GH88**	0.11	0.16	0.14	0.07	0.17	0.13	0.15	0.09	0.07	0.10	0.00	0.08	0.31	0.45	0.07	0.17	0.37	0.32	0.30	0.18
**CE5**	0.00	0.00	0.00	0.00	0.00	0.00	0.00	0.00	0.00	0.00	0.00	0.00	0.00	0.00	0.00	0.00	0.00	0.00	0.00	0.00
**GT8**	0.11	0.14	0.02	0.07	0.17	0.06	0.06	0.18	0.31	0.06	0.35	0.07	0.12	0.09	0.01	0.08	0.07	0.11	0.07	0.12
**GT50**	0.00	0.00	0.00	0.00	0.00	0.00	0.00	0.00	0.00	0.00	0.00	0.00	0.00	0.00	0.00	0.00	0.00	0.00	0.00	0.00
**GT20**	0.00	0.00	0.00	0.00	0.00	0.00	0.00	0.03	0.00	0.00	0.00	0.02	0.08	0.09	0.02	0.03	0.07	0.09	0.05	0.07
**PL9**	0.00	0.04	0.00	0.02	0.22	0.00	0.01	0.09	0.15	0.00	0.00	0.02	0.05	0.10	0.02	0.09	0.14	0.14	0.13	0.10
**GH55**	0.00	0.00	0.00	0.02	0.06	0.00	0.00	0.00	0.00	0.00	0.00	0.02	0.01	0.03	0.00	0.05	0.03	0.04	0.01	0.04
**GH64**	0.02	0.00	0.00	0.00	0.00	0.00	0.00	0.00	0.00	0.00	0.00	0.00	0.00	0.00	0.00	0.00	0.02	0.02	0.01	0.00
**GH127**	0.45	0.26	0.40	0.24	0.11	0.28	0.31	0.39	0.46	0.27	0.14	0.39	0.25	0.34	0.07	0.20	0.34	0.30	0.17	0.19
**GH103**	0.00	0.02	0.00	0.00	0.00	0.00	0.00	0.00	0.00	0.00	0.07	0.02	0.00	0.00	0.00	0.00	0.00	0.00	0.01	0.00
**GH3**	2.61	2.32	2.34	2.32	2.13	2.54	2.14	1.67	1.93	2.33	0.71	2.62	2.73	3.01	0.64	2.00	2.59	2.96	2.33	2.42
**GT45**	0.00	0.00	0.00	0.00	0.00	0.00	0.00	0.00	0.00	0.00	0.00	0.00	0.00	0.00	0.00	0.00	0.00	0.00	0.00	0.00
**GT74**	0.00	0.00	0.00	0.00	0.00	0.00	0.00	0.00	0.00	0.00	0.00	0.00	0.00	0.00	0.00	0.00	0.00	0.00	0.00	0.00
**GT90**	0.00	0.00	0.00	0.00	0.00	0.00	0.00	0.00	0.00	0.02	0.00	0.00	0.00	0.00	0.00	0.00	0.00	0.01	0.00	0.02
**GH116**	0.00	0.00	0.00	0.00	0.00	0.00	0.00	0.00	0.00	0.00	0.00	0.02	0.02	0.09	0.02	0.04	0.05	0.05	0.05	0.03
**GH31**	0.95	0.86	0.82	0.69	0.90	0.77	0.76	0.60	0.73	0.81	0.50	1.13	1.08	1.18	0.23	0.70	1.04	0.96	0.84	0.86
**GT12**	0.00	0.00	0.00	0.00	0.00	0.00	0.00	0.00	0.00	0.00	0.00	0.00	0.00	0.00	0.00	0.00	0.00	0.00	0.00	0.00
**CE1**	0.32	0.30	0.19	0.38	0.34	0.21	0.10	0.12	0.24	0.29	0.14	0.28	0.32	0.42	0.06	0.21	0.35	0.42	0.22	0.35
**GH121**	0.00	0.00	0.00	0.00	0.00	0.00	0.00	0.00	0.02	0.00	0.00	0.00	0.00	0.03	0.00	0.00	0.00	0.01	0.00	0.00
**GH79**	0.00	0.00	0.02	0.00	0.00	0.00	0.00	0.00	0.00	0.00	0.00	0.00	0.00	0.00	0.00	0.00	0.01	0.00	0.00	0.00
**GT73**	0.00	0.00	0.00	0.00	0.00	0.00	0.00	0.03	0.00	0.00	0.00	0.00	0.00	0.00	0.00	0.00	0.00	0.00	0.00	0.00
**GH77**	0.52	0.51	0.46	0.55	0.90	0.37	0.27	0.69	0.55	0.31	0.35	0.56	0.66	0.69	0.24	0.54	0.70	0.67	0.62	0.36
**PL17**	0.00	0.00	0.00	0.00	0.00	0.00	0.00	0.00	0.00	0.00	0.00	0.00	0.00	0.01	0.00	0.00	0.00	0.00	0.01	0.00
**PL8**	0.13	0.07	0.05	0.00	0.00	0.07	0.07	0.06	0.04	0.06	0.00	0.00	0.25	0.39	0.07	0.19	0.20	0.22	0.25	0.14

CAZY_group	IN-CH-1	IN-CH-2	IN-CH-3	IN-CH-4	IN-CH-5	IN-CH-6	IN-CH-7	IN-CH-8	IN-CH-9	IN-IN-1	IN-CH-10	IN-CH-11	AM-AD-10	AM-AD-11	AM-AD-12	AM-AD-13	AM-AD-14	AM-AD-15	AM-AD-16	AM-AD-17
CE9	0.26	0.28	0.16	0.20	0.45	0.07	0.15	0.33	0.31	0.08	0.21	0.26	0.30	0.44	0.14	0.26	0.38	0.36	0.34	0.29
CE15	0.00	0.00	0.00	0.00	0.00	0.00	0.00	0.00	0.00	0.00	0.00	0.00	0.01	0.02	0.01	0.00	0.02	0.02	0.01	0.02
GT79	0.00	0.00	0.00	0.00	0.00	0.00	0.00	0.00	0.00	0.00	0.00	0.00	0.00	0.00	0.00	0.00	0.00	0.00	0.00	0.00
GT10	0.02	0.00	0.00	0.04	0.00	0.00	0.00	0.00	0.02	0.00	0.00	0.02	0.01	0.00	0.00	0.01	0.01	0.01	0.02	0.00
GH119	0.00	0.00	0.00	0.00	0.00	0.00	0.00	0.00	0.00	0.00	0.00	0.00	0.00	0.00	0.00	0.00	0.00	0.00	0.00	0.00
GH16	0.19	0.09	0.16	0.15	0.11	0.19	0.18	0.27	0.18	0.15	0.00	0.16	0.27	0.29	0.03	0.15	0.19	0.26	0.20	0.11
GH43	2.81	2.06	2.47	1.39	1.80	2.52	1.98	1.58	2.01	2.27	0.14	1.92	2.07	2.68	0.32	1.00	1.83	2.14	1.40	1.37
GH51	0.95	0.60	0.91	0.69	0.62	0.99	0.88	0.60	0.71	0.67	0.21	0.67	0.48	0.70	0.08	0.32	0.56	0.59	0.43	0.26
GT35	0.60	1.14	0.55	0.77	0.90	0.67	0.50	0.75	0.57	0.52	0.50	0.74	1.35	1.14	0.36	0.96	1.33	1.02	0.99	0.86
GT25	0.00	0.00	0.00	0.00	0.00	0.00	0.00	0.03	0.00	0.00	0.00	0.00	0.00	0.02	0.00	0.00	0.00	0.00	0.01	0.00
GT2	4.77	5.19	4.46	4.80	4.94	4.35	3.88	5.55	4.72	4.43	3.12	5.46	5.11	5.28	1.08	3.65	4.83	5.06	4.60	3.26
GT51	1.14	1.09	1.01	1.37	0.95	1.01	1.11	1.73	1.51	1.10	0.78	1.18	1.02	1.06	0.25	0.83	0.99	0.81	0.83	0.69
GH107	0.00	0.00	0.00	0.00	0.00	0.00	0.00	0.00	0.00	0.00	0.00	0.00	0.00	0.00	0.00	0.00	0.00	0.00	0.00	0.00
GH7	0.00	0.00	0.00	0.00	0.00	0.00	0.00	0.00	0.00	0.00	0.00	0.00	0.00	0.00	0.00	0.00	0.00	0.00	0.00	0.00
GT7	0.00	0.00	0.00	0.00	0.00	0.00	0.00	0.00	0.00	0.00	0.00	0.00	0.00	0.00	0.00	0.00	0.00	0.00	0.00	0.00
GT36	0.00	0.00	0.00	0.00	0.00	0.00	0.00	0.00	0.00	0.00	0.00	0.00	0.00	0.00	0.00	0.00	0.00	0.00	0.00	0.00
PL7	0.00	0.00	0.00	0.00	0.00	0.00	0.00	0.00	0.00	0.00	0.00	0.00	0.00	0.00	0.00	0.00	0.00	0.00	0.00	0.00
GH110	0.04	0.02	0.10	0.07	0.00	0.04	0.04	0.03	0.05	0.08	0.00	0.05	0.15	0.12	0.03	0.10	0.06	0.10	0.12	0.04
GT21	0.00	0.00	0.00	0.00	0.00	0.00	0.00	0.00	0.00	0.00	0.00	0.00	0.00	0.00	0.00	0.00	0.00	0.00	0.00	0.00
GH62	0.00	0.00	0.00	0.00	0.00	0.00	0.00	0.00	0.00	0.00	0.00	0.00	0.00	0.00	0.00	0.00	0.00	0.00	0.00	0.00
GH125	0.09	0.14	0.16	0.11	0.06	0.09	0.07	0.12	0.11	0.10	0.07	0.21	0.17	0.14	0.03	0.17	0.13	0.17	0.14	0.12
GH117	0.02	0.05	0.02	0.02	0.00	0.02	0.00	0.00	0.04	0.02	0.00	0.00	0.12	0.10	0.02	0.02	0.10	0.09	0.08	0.03
GH68	0.00	0.00	0.00	0.00	0.00	0.00	0.00	0.00	0.05	0.00	0.00	0.00	0.00	0.00	0.00	0.00	0.00	0.00	0.00	0.00
GH85	0.15	0.19	0.11	0.09	0.11	0.06	0.03	0.12	0.05	0.08	0.14	0.02	0.03	0.01	0.01	0.01	0.04	0.01	0.02	0.01
GH92	0.93	1.02	0.98	1.26	0.62	0.71	0.60	0.48	0.69	0.98	0.00	0.98	1.45	2.01	0.28	0.68	1.52	1.59	1.33	0.96
GH40	0.00	0.00	0.00	0.00	0.00	0.00	0.00	0.00	0.00	0.00	0.00	0.00	0.00	0.00	0.00	0.00	0.00	0.00	0.00	0.00
GT49	0.00	0.00	0.00	0.00	0.00	0.00	0.00	0.00	0.00	0.00	0.00	0.00	0.00	0.00	0.00	0.00	0.00	0.00	0.00	0.01
GH112	0.02	0.16	0.05	0.07	0.17	0.07	0.04	0.06	0.04	0.02	0.07	0.15	0.11	0.26	0.09	0.21	0.26	0.17	0.24	0.16
GT67	0.00	0.00	0.00	0.00	0.00	0.00	0.00	0.00	0.00	0.00	0.00	0.00	0.00	0.00	0.00	0.00	0.00	0.00	0.00	0.00
CE8	0.28	0.25	0.18	0.15	0.17	0.26	0.25	0.15	0.16	0.10	0.07	0.20	0.19	0.27	0.03	0.15	0.23	0.17	0.24	0.17
GT86	0.00	0.00	0.00	0.00	0.00	0.00	0.00	0.00	0.00	0.00	0.00	0.00	0.00	0.00	0.00	0.00	0.00	0.00	0.00	0.00
GH101	0.00	0.00	0.00	0.00	0.00	0.00	0.00	0.00	0.00	0.00	0.00	0.00	0.02	0.07	0.00	0.03	0.02	0.01	0.00	0.00
GH81	0.00	0.00	0.00	0.00	0.00	0.00	0.00	0.00	0.00	0.00	0.00	0.02	0.00	0.01	0.00	0.02	0.01	0.01	0.01	0.00
GT93	0.00	0.00	0.00	0.00	0.00	0.00	0.00	0.00	0.00	0.00	0.00	0.00	0.00	0.00	0.00	0.00	0.00	0.00	0.00	0.00
GT63	0.00	0.00	0.00	0.00	0.00	0.00	0.00	0.00	0.00	0.00	0.00	0.00	0.00	0.00	0.00	0.00	0.00	0.00	0.00	0.00
CE11	0.35	0.44	0.37	0.24	0.17	0.35	0.36	0.33	0.44	0.44	0.07	0.43	0.29	0.27	0.09	0.19	0.17	0.19	0.18	0.14
GT19	0.32	0.21	0.21	0.18	0.17	0.24	0.22	0.24	0.31	0.35	0.07	0.34	0.14	0.12	0.03	0.10	0.11	0.11	0.10	0.08
GT33	0.00	0.00	0.00	0.00	0.00	0.00	0.00	0.00	0.00	0.00	0.00	0.00	0.00	0.00	0.00	0.00	0.00	0.00	0.00	0.00
GH21	0.00	0.00	0.00	0.00	0.00	0.00	0.00	0.00	0.00	0.00	0.00	0.00	0.00	0.00	0.00	0.00	0.00	0.00	0.00	0.00
GT68	0.00	0.00	0.00	0.00	0.00	0.00	0.00	0.00	0.00	0.00	0.00	0.00	0.00	0.00	0.00	0.00	0.00	0.00	0.00	0.00
GH89	0.24	0.19	0.22	0.13	0.06	0.17	0.14	0.21	0.16	0.25	0.00	0.26	0.24	0.30	0.07	0.11	0.25	0.19	0.21	0.11
GT82	0.00	0.00	0.00	0.00	0.00	0.00	0.00	0.00	0.00	0.00	0.00	0.00	0.01	0.00	0.00	0.00	0.00	0.00	0.00	0.00
GH30	0.13	0.16	0.05	0.07	0.06	0.15	0.11	0.15	0.11	0.10	0.00	0.15	0.18	0.27	0.04	0.07	0.19	0.35	0.14	0.14
GH20	1.27	0.95	1.19	0.93	0.95	0.86	0.91	0.72	0.71	1.02	0.35	0.77	1.07	1.56	0.20	0.85	1.08	1.01	0.96	0.58
GH52	0.00	0.00	0.00	0.00	0.00	0.00	0.00	0.00	0.00	0.00	0.00	0.00	0.00	0.00	0.00	0.00	0.00	0.00	0.00	0.00
GT84	0.00	0.00	0.00	0.00	0.00	0.00	0.00	0.18	0.00	0.00	0.00	0.00	0.14	0.05	0.00	0.02	0.00	0.06	0.02	0.03
GT15	0.00	0.00	0.00	0.00	0.00	0.00	0.00	0.00	0.00	0.00	0.00	0.00	0.00	0.00	0.00	0.00	0.00	0.00	0.00	0.00
GH93	0.00	0.00	0.00	0.00	0.00	0.00	0.00	0.00	0.00	0.00	0.00	0.00	0.00	0.01	0.01	0.00	0.00	0.00	0.01	0.00
PL16	0.00	0.00	0.00	0.00	0.00	0.00	0.00	0.00	0.00	0.00	0.00	0.00	0.00	0.00	0.00	0.00	0.00	0.00	0.00	0.00
GH26	0.11	0.12	0.06	0.11	0.00	0.09	0.07	0.06	0.15	0.02	0.00	0.13	0.15	0.14	0.02	0.14	0.18	0.17	0.09	0.15

CAZY_group	IN-CH-1	IN-CH-2	IN-CH-3	IN-CH-4	IN-CH-5	IN-CH-6	IN-CH-7	IN-CH-8	IN-CH-9	IN-IN-1	IN-CH-10	IN-CH-11	AM-AD-10	AM-AD-11	AM-AD-12	AM-AD-13	AM-AD-14	AM-AD-15	AM-AD-16	AM-AD-17
GT75	0.00	0.00	0.00	0.00	0.00	0.00	0.00	0.00	0.00	0.00	0.00	0.00	0.00	0.00	0.00	0.00	0.00	0.00	0.00	0.00
GH53	0.24	0.18	0.10	0.11	0.11	0.11	0.17	0.33	0.27	0.12	0.00	0.20	0.22	0.15	0.05	0.12	0.23	0.12	0.10	0.12
GT88	0.00	0.00	0.00	0.00	0.00	0.00	0.00	0.00	0.00	0.00	0.00	0.00	0.00	0.00	0.00	0.00	0.00	0.00	0.00	0.00
GT24	0.00	0.00	0.00	0.00	0.00	0.00	0.00	0.00	0.00	0.00	0.00	0.00	0.00	0.00	0.00	0.00	0.00	0.00	0.00	0.01
GT81	0.00	0.00	0.00	0.00	0.00	0.00	0.00	0.00	0.00	0.00	0.00	0.00	0.00	0.00	0.00	0.00	0.00	0.00	0.00	0.00
GH4	0.00	0.05	0.03	0.02	0.06	0.00	0.04	0.36	0.18	0.02	0.35	0.23	0.14	0.15	0.04	0.09	0.09	0.16	0.10	0.11
GT78	0.00	0.00	0.00	0.00	0.00	0.00	0.00	0.00	0.00	0.00	0.00	0.00	0.00	0.00	0.00	0.00	0.00	0.00	0.00	0.00
GH8	0.15	0.11	0.10	0.07	0.06	0.06	0.06	0.12	0.02	0.08	0.07	0.08	0.11	0.06	0.01	0.05	0.05	0.07	0.05	0.05
GH70	0.00	0.02	0.00	0.00	0.00	0.00	0.00	0.27	0.42	0.00	0.00	0.00	0.00	0.00	0.00	0.00	0.00	0.00	0.00	0.00
GH122	0.00	0.00	0.00	0.00	0.00	0.00	0.00	0.00	0.00	0.00	0.00	0.00	0.00	0.00	0.00	0.00	0.00	0.00	0.00	0.00
GH74	0.00	0.00	0.03	0.04	0.00	0.00	0.00	0.00	0.00	0.00	0.00	0.00	0.05	0.02	0.00	0.03	0.06	0.02	0.02	0.05
GT41	0.00	0.00	0.00	0.00	0.00	0.00	0.00	0.03	0.00	0.00	0.00	0.08	0.01	0.01	0.01	0.00	0.01	0.01	0.02	0.01
PL1	0.13	0.07	0.10	0.18	0.34	0.21	0.11	0.09	0.24	0.12	0.00	0.11	0.27	0.38	0.04	0.17	0.34	0.41	0.17	0.21
GH15	0.00	0.00	0.00	0.00	0.00	0.00	0.00	0.00	0.00	0.00	0.00	0.00	0.04	0.08	0.00	0.00	0.03	0.07	0.02	0.04
GH32	0.71	0.51	0.67	0.33	0.79	0.58	0.48	0.98	0.75	0.62	0.21	0.49	0.50	0.60	0.08	0.35	0.58	0.47	0.32	0.26
GH44	0.00	0.00	0.00	0.00	0.00	0.00	0.00	0.00	0.00	0.00	0.00	0.00	0.01	0.01	0.00	0.00	0.01	0.00	0.00	0.00
GT6	0.00	0.00	0.00	0.00	0.00	0.00	0.00	0.00	0.00	0.00	0.00	0.00	0.01	0.02	0.01	0.00	0.01	0.01	0.01	0.00
GH13	3.09	3.82	2.31	3.87	3.59	2.63	2.34	4.06	3.57	2.62	1.92	3.00	3.47	3.86	1.01	2.84	3.81	3.38	3.08	2.76
GT80	0.00	0.00	0.00	0.00	0.06	0.00	0.01	0.03	0.00	0.00	0.14	0.03	0.02	0.01	0.01	0.03	0.04	0.03	0.02	0.01
GH71	0.00	0.00	0.00	0.00	0.00	0.00	0.00	0.00	0.00	0.00	0.00	0.00	0.00	0.00	0.00	0.00	0.00	0.00	0.00	0.00
GH63	0.06	0.12	0.08	0.09	0.28	0.06	0.03	0.15	0.02	0.02	0.07	0.02	0.04	0.09	0.02	0.06	0.09	0.09	0.04	0.14
PL21	0.00	0.00	0.00	0.00	0.00	0.00	0.00	0.00	0.00	0.00	0.00	0.00	0.00	0.03	0.00	0.00	0.01	0.00	0.01	0.00
GH65	0.00	0.02	0.00	0.02	0.06	0.04	0.03	0.15	0.05	0.00	0.07	0.10	0.18	0.21	0.06	0.11	0.18	0.23	0.22	0.19
CE4	0.39	0.44	0.32	0.51	0.45	0.30	0.39	0.54	0.31	0.29	0.14	0.44	0.73	0.72	0.16	0.53	0.77	0.62	0.57	0.52
CE14	0.00	0.02	0.00	0.00	0.00	0.00	0.01	0.00	0.04	0.00	0.00	0.03	0.02	0.03	0.00	0.01	0.01	0.01	0.01	0.00
GH115	0.22	0.25	0.35	0.15	0.06	0.17	0.29	0.15	0.22	0.19	0.00	0.16	0.24	0.33	0.04	0.17	0.23	0.25	0.12	0.22
GH104	0.00	0.00	0.00	0.00	0.00	0.00	0.03	0.06	0.00	0.00	0.00	0.00	0.00	0.00	0.00	0.00	0.00	0.00	0.00	0.00
GH111	0.00	0.00	0.00	0.00	0.00	0.00	0.00	0.00	0.00	0.00	0.00	0.00	0.00	0.00	0.00	0.00	0.00	0.00	0.00	0.00
GH95	0.82	0.49	0.82	0.60	0.67	0.62	0.70	0.45	0.46	0.83	0.00	0.44	0.62	0.82	0.09	0.29	0.50	0.48	0.49	0.36
GT70	0.00	0.00	0.00	0.00	0.00	0.00	0.00	0.00	0.00	0.00	0.00	0.00	0.00	0.00	0.00	0.00	0.00	0.00	0.00	0.00
CE10	0.02	0.05	0.05	0.09	0.06	0.04	0.03	0.06	0.09	0.06	0.28	0.16	0.12	0.15	0.04	0.17	0.12	0.25	0.17	0.19
GH10	0.24	0.16	0.24	0.11	0.06	0.06	0.13	0.15	0.13	0.23	0.00	0.18	0.14	0.15	0.01	0.06	0.15	0.17	0.07	0.10
GH130	0.24	0.25	0.24	0.27	0.17	0.28	0.24	0.15	0.22	0.15	0.00	0.29	0.38	0.41	0.07	0.14	0.34	0.30	0.23	0.16
PL20	0.00	0.00	0.00	0.00	0.00	0.00	0.00	0.00	0.00	0.00	0.00	0.00	0.00	0.00	0.00	0.00	0.00	0.00	0.00	0.00
GH69	0.00	0.00	0.00	0.00	0.00	0.00	0.00	0.00	0.00	0.00	0.00	0.00	0.00	0.00	0.00	0.00	0.00	0.00	0.00	0.00
PL2	0.00	0.00	0.00	0.00	0.00	0.00	0.00	0.00	0.00	0.00	0.00	0.00	0.00	0.00	0.00	0.00	0.00	0.00	0.00	0.00
GH49	0.00	0.00	0.00	0.00	0.00	0.00	0.00	0.00	0.00	0.00	0.00	0.00	0.00	0.00	0.00	0.00	0.00	0.00	0.00	0.00
GH25	0.71	0.77	0.77	0.55	0.84	0.77	0.77	1.07	1.00	0.75	0.00	0.66	0.43	0.48	0.14	0.32	0.45	0.44	0.46	0.42
GH59	0.00	0.00	0.00	0.00	0.00	0.00	0.00	0.00	0.00	0.00	0.00	0.00	0.06	0.02	0.00	0.02	0.02	0.02	0.02	0.03
GH35	0.35	0.25	0.38	0.22	0.28	0.32	0.36	0.21	0.20	0.25	0.21	0.29	0.37	0.41	0.05	0.23	0.35	0.29	0.30	0.19
GT39	0.00	0.02	0.00	0.00	0.00	0.00	0.00	0.00	0.00	0.00	0.00	0.00	0.01	0.01	0.01	0.02	0.01	0.00	0.02	0.01
GT57	0.00	0.00	0.00	0.00	0.00	0.00	0.00	0.00	0.00	0.00	0.00	0.00	0.00	0.00	0.00	0.00	0.00	0.00	0.00	0.00
GH129	0.00	0.00	0.00	0.00	0.00	0.00	0.00	0.00	0.00	0.00	0.07	0.00	0.00	0.00	0.00	0.00	0.02	0.01	0.00	0.00
GT22	0.00	0.00	0.00	0.00	0.00	0.00	0.00	0.00	0.00	0.00	0.00	0.00	0.00	0.00	0.00	0.00	0.00	0.00	0.00	0.03
PL12	0.02	0.02	0.02	0.02	0.00	0.02	0.00	0.00	0.00	0.02	0.00	0.02	0.09	0.19	0.02	0.03	0.11	0.09	0.07	0.00
PL18	0.00	0.00	0.00	0.00	0.00	0.00	0.00	0.00	0.00	0.00	0.00	0.00	0.00	0.00	0.00	0.00	0.00	0.00	0.00	0.00
GT92	0.00	0.00	0.00	0.00	0.00	0.00	0.00	0.00	0.00	0.00	0.00	0.00	0.00	0.00	0.00	0.00	0.00	0.00	0.00	0.00
GT44	0.00	0.00	0.00	0.00	0.00	0.00	0.00	0.00	0.00	0.00	0.00	0.00	0.01	0.00	0.00	0.00	0.00	0.00	0.00	0.00
GT29	0.00	0.00	0.00	0.00	0.00	0.00	0.00	0.00	0.00	0.00	0.00	0.00	0.00	0.00	0.00	0.00	0.00	0.00	0.00	0.00
GT13	0.00	0.00	0.00	0.00	0.00	0.00	0.00	0.00	0.00	0.00	0.00	0.00	0.00	0.00	0.00	0.00	0.00	0.01	0.00	0.01

CAZY_group	IN-CH-1	IN-CH-2	IN-CH-3	IN-CH-4	IN-CH-5	IN-CH-6	IN-CH-7	IN-CH-8	IN-CH-9	IN-IN-1	IN-CH-10	IN-CH-11	AM-AD-10	AM-AD-11	AM-AD-12	AM-AD-13	AM-AD-14	AM-AD-15	AM-AD-16	AM-AD-17
**GT52**	0.00	0.00	0.00	0.00	0.00	0.00	0.00	0.00	0.00	0.00	0.00	0.00	0.00	0.00	0.00	0.00	0.00	0.00	0.00	0.00
**GT64**	0.00	0.00	0.00	0.00	0.00	0.00	0.00	0.00	0.00	0.00	0.00	0.00	0.00	0.00	0.00	0.00	0.00	0.00	0.00	0.00
**GT85**	0.00	0.00	0.00	0.00	0.00	0.00	0.00	0.00	0.00	0.00	0.00	0.00	0.00	0.00	0.00	0.00	0.00	0.00	0.00	0.00
**GT16**	0.00	0.00	0.00	0.00	0.00	0.00	0.00	0.00	0.00	0.00	0.00	0.00	0.00	0.00	0.00	0.00	0.00	0.00	0.00	0.00
**GH56**	0.00	0.00	0.00	0.00	0.00	0.00	0.00	0.00	0.00	0.00	0.00	0.00	0.00	0.00	0.00	0.00	0.00	0.00	0.00	0.00
**GT60**	0.00	0.00	0.00	0.00	0.00	0.00	0.00	0.00	0.00	0.00	0.00	0.00	0.00	0.00	0.00	0.00	0.00	0.00	0.00	0.00
**GH41**	0.00	0.00	0.00	0.00	0.00	0.00	0.00	0.00	0.00	0.00	0.00	0.00	0.00	0.00	0.00	0.00	0.00	0.00	0.00	0.00
**CE7**	0.15	0.12	0.24	0.13	0.34	0.21	0.22	0.30	0.22	0.12	0.00	0.13	0.05	0.13	0.02	0.04	0.10	0.09	0.05	0.07
**GH39**	0.02	0.02	0.00	0.00	0.00	0.00	0.00	0.00	0.02	0.00	0.00	0.05	0.07	0.06	0.01	0.01	0.05	0.05	0.03	0.04
**GT30**	0.37	0.28	0.42	0.31	0.28	0.26	0.25	0.36	0.33	0.48	0.14	0.43	0.29	0.24	0.05	0.19	0.19	0.21	0.19	0.19
**PL15**	0.02	0.09	0.02	0.00	0.00	0.04	0.00	0.00	0.00	0.04	0.00	0.03	0.05	0.12	0.02	0.03	0.13	0.06	0.08	0.02
**GH102**	0.00	0.02	0.02	0.00	0.00	0.02	0.03	0.06	0.04	0.02	0.14	0.02	0.00	0.00	0.01	0.00	0.00	0.00	0.02	0.00

CAZY_group	AM-AD-18	AM-AD-19	AM-AD-20	AM-AD-21	AM-AD-22	AM-AD-23	AM-AD-24	AM-AD-25	AM-AD-26	AM-AD-27	AM-AD-28	AM-AD-29	AM-AD-3	AM-AD-30	AM-AD-31	AM-AD-32	AM-AD-33
**GH29**	0.67	0.74	0.36	0.64	0.72	0.83	0.85	1.15	0.62	0.89	0.75	0.97	0.95	1.07	1.03	0.58	0.64
**PL4**	0.00	0.00	0.00	0.01	0.00	0.01	0.00	0.00	0.00	0.02	0.00	0.00	0.00	0.01	0.00	0.01	0.00
**GH23**	1.22	1.23	1.30	1.10	1.16	1.24	1.45	1.46	1.34	1.20	1.45	1.90	1.16	1.20	1.31	1.41	1.33
**CE6**	0.00	0.06	0.03	0.02	0.04	0.04	0.04	0.06	0.01	0.03	0.06	0.06	0.05	0.07	0.03	0.03	0.04
**GH72**	0.14	0.14	0.22	0.13	0.15	0.16	0.20	0.09	0.12	0.16	0.21	0.25	0.15	0.15	0.24	0.04	0.11
**GH114**	0.00	0.00	0.00	0.00	0.00	0.00	0.00	0.00	0.01	0.00	0.00	0.00	0.00	0.00	0.00	0.00	0.00
**GH78**	0.77	0.94	0.50	1.01	1.23	0.93	1.14	1.59	0.62	1.39	0.91	1.04	1.10	1.48	1.63	0.89	0.94
**PL19**	0.00	0.00	0.00	0.00	0.00	0.00	0.00	0.00	0.00	0.00	0.00	0.00	0.00	0.00	0.00	0.00	0.00
**GT27**	0.00	0.00	0.01	0.00	0.00	0.00	0.00	0.00	0.00	0.00	0.00	0.00	0.00	0.00	0.00	0.00	0.00
**GH5**	0.28	0.32	0.41	0.39	0.60	0.74	0.33	0.23	0.17	0.76	0.58	0.60	0.59	0.98	0.74	0.63	0.50
**GT40**	0.00	0.00	0.00	0.00	0.00	0.00	0.00	0.00	0.00	0.00	0.00	0.00	0.00	0.00	0.00	0.00	0.00
**CE13**	0.01	0.00	0.01	0.00	0.00	0.00	0.00	0.00	0.00	0.00	0.00	0.00	0.00	0.00	0.01	0.00	0.00
**GT37**	0.00	0.00	0.00	0.00	0.00	0.00	0.00	0.00	0.00	0.00	0.00	0.00	0.00	0.00	0.00	0.00	0.00
**GH33**	0.15	0.28	0.11	0.23	0.23	0.31	0.30	0.27	0.20	0.28	0.30	0.42	0.25	0.40	0.30	0.22	0.24
**GT65**	0.00	0.00	0.00	0.00	0.00	0.00	0.00	0.00	0.00	0.00	0.00	0.00	0.00	0.00	0.00	0.00	0.00
**GH86**	0.00	0.01	0.00	0.01	0.01	0.00	0.00	0.00	0.00	0.00	0.00	0.03	0.00	0.00	0.01	0.00	0.00
**GH123**	0.11	0.15	0.04	0.11	0.15	0.11	0.23	0.22	0.17	0.16	0.09	0.14	0.15	0.23	0.16	0.16	0.12
**GH96**	0.00	0.00	0.00	0.00	0.00	0.00	0.00	0.00	0.00	0.00	0.00	0.00	0.00	0.00	0.00	0.00	0.00
**GH14**	0.00	0.00	0.00	0.00	0.00	0.00	0.00	0.00	0.00	0.00	0.00	0.00	0.00	0.00	0.00	0.00	0.00
**CE3**	0.02	0.00	0.00	0.00	0.00	0.00	0.00	0.00	0.00	0.00	0.00	0.00	0.00	0.00	0.01	0.00	0.00
**PL10**	0.03	0.13	0.06	0.07	0.12	0.14	0.18	0.10	0.11	0.12	0.08	0.11	0.09	0.15	0.11	0.10	0.06
**GT48**	0.00	0.00	0.00	0.00	0.00	0.00	0.00	0.00	0.00	0.00	0.00	0.00	0.00	0.00	0.00	0.00	0.00
**PL6**	0.00	0.01	0.00	0.00	0.02	0.00	0.01	0.00	0.00	0.01	0.00	0.00	0.00	0.00	0.00	0.00	0.00
**GT83**	0.08	0.15	0.06	0.07	0.11	0.04	0.21	0.11	0.04	0.11	0.15	0.17	0.12	0.14	0.09	0.12	0.10
**GH126**	0.00	0.00	0.00	0.00	0.00	0.00	0.00	0.00	0.00	0.00	0.00	0.00	0.00	0.00	0.00	0.00	0.00
**GH9**	0.19	0.07	0.17	0.15	0.20	0.14	0.14	0.14	0.15	0.37	0.28	0.33	0.33	0.28	0.23	0.14	0.24
**GH75**	0.00	0.00	0.00	0.00	0.00	0.00	0.00	0.00	0.00	0.00	0.00	0.00	0.00	0.00	0.00	0.00	0.00
**GT71**	0.00	0.00	0.00	0.00	0.00	0.00	0.00	0.00	0.00	0.00	0.00	0.00	0.00	0.00	0.00	0.00	0.00
**GH46**	0.00	0.00	0.00	0.00	0.00	0.00	0.00	0.00	0.00	0.01	0.00	0.00	0.00	0.00	0.00	0.00	0.00
**GT61**	0.00	0.00	0.00	0.00	0.00	0.00	0.00	0.00	0.00	0.00	0.00	0.00	0.00	0.00	0.00	0.00	0.00
**GT3**	0.11	0.22	0.03	0.20	0.24	0.13	0.44	0.19	0.36	0.30	0.29	0.36	0.28	0.17	0.31	0.24	0.35
**PL3**	0.00	0.00	0.00	0.00	0.00	0.00	0.00	0.00	0.00	0.00	0.00	0.01	0.00	0.00	0.00	0.01	0.00
**GH108**	0.04	0.06	0.02	0.02	0.04	0.03	0.04	0.07	0.01	0.03	0.01	0.03	0.02	0.06	0.04	0.02	0.03
**GT56**	0.00	0.00	0.00	0.00	0.00	0.00	0.00	0.00	0.00	0.00	0.00	0.01	0.00	0.00	0.00	0.02	0.00
**PL11**	0.10	0.22	0.09	0.14	0.10	0.25	0.21	0.22	0.12	0.22	0.09	0.18	0.11	0.30	0.21	0.29	0.13
**GH45**	0.00	0.00	0.00	0.01	0.00	0.00	0.01	0.00	0.00	0.00	0.00	0.00	0.00	0.00	0.00	0.00	0.00
**GT34**	0.00	0.00	0.00	0.00	0.00	0.00	0.00	0.00	0.00	0.00	0.00	0.03	0.00	0.00	0.00	0.00	0.01
**PL14**	0.00	0.00	0.00	0.00	0.00	0.00	0.00	0.00	0.00	0.00	0.00	0.00	0.00	0.00	0.00	0.00	0.00
**GH19**	0.01	0.00	0.01	0.01	0.01	0.02	0.00	0.01	0.01	0.01	0.01	0.00	0.01	0.01	0.01	0.04	0.00
**GH118**	0.00	0.00	0.00	0.00	0.00	0.00	0.00	0.00	0.00	0.00	0.00	0.00	0.00	0.00	0.00	0.00	0.00
**GH82**	0.00	0.00	0.00	0.00	0.00	0.00	0.00	0.00	0.00	0.00	0.00	0.00	0.00	0.00	0.00	0.00	0.00
**GH76**	0.09	0.13	0.07	0.19	0.28	0.29	0.26	0.55	0.06	0.27	0.26	0.34	0.26	0.28	0.20	0.17	0.24
**GT4**	1.63	2.09	2.01	1.95	2.48	2.38	2.37	2.28	2.22	2.58	2.50	2.97	2.60	2.28	2.74	2.17	2.10
**GT87**	0.01	0.01	0.00	0.01	0.01	0.02	0.01	0.00	0.00	0.00	0.01	0.00	0.01	0.01	0.01	0.00	0.00
**GH24**	0.10	0.13	0.09	0.13	0.13	0.07	0.18	0.30	0.20	0.14	0.12	0.13	0.10	0.23	0.12	0.21	0.15
**GH90**	0.00	0.00	0.00	0.00	0.00	0.00	0.00	0.00	0.00	0.00	0.00	0.00	0.00	0.00	0.00	0.00	0.00
**GH57**	0.06	0.11	0.03	0.23	0.15	0.16	0.34	0.22	0.25	0.19	0.13	0.25	0.26	0.18	0.17	0.10	0.17
**GH36**	0.73	0.67	1.02	0.66	0.99	0.96	0.86	0.80	0.69	1.10	1.04	1.26	1.00	1.01	1.26	0.97	0.76
**GT46**	0.00	0.00	0.00	0.00	0.00	0.00	0.00	0.00	0.00	0.00	0.00	0.00	0.00	0.00	0.00	0.00	0.00
**GH66**	0.02	0.03	0.01	0.03	0.08	0.05	0.02	0.11	0.02	0.08	0.00	0.05	0.07	0.05	0.04	0.01	0.04

CAZY_group	AM-AD-18	AM-AD-19	AM-AD-20	AM-AD-21	AM-AD-22	AM-AD-23	AM-AD-24	AM-AD-25	AM-AD-26	AM-AD-27	AM-AD-28	AM-AD-29	AM-AD-3	AM-AD-30	AM-AD-31	AM-AD-32	AM-AD-33
**GH48**	0.01	0.00	0.01	0.00	0.00	0.00	0.00	0.00	0.00	0.00	0.00	0.00	0.00	0.00	0.00	0.00	0.00
**GT53**	0.00	0.00	0.00	0.00	0.00	0.00	0.00	0.00	0.00	0.00	0.00	0.00	0.00	0.00	0.00	0.00	0.00
**GH60**	0.00	0.00	0.00	0.00	0.00	0.00	0.00	0.00	0.00	0.00	0.00	0.00	0.00	0.00	0.00	0.00	0.00
**GT31**	0.00	0.00	0.00	0.00	0.00	0.00	0.00	0.00	0.00	0.00	0.00	0.00	0.00	0.00	0.00	0.01	0.00
**GH105**	0.20	0.53	0.36	0.51	0.78	0.61	0.63	1.28	0.53	0.80	0.36	0.46	0.52	1.04	0.87	0.54	0.47
**GT23**	0.00	0.01	0.01	0.01	0.00	0.02	0.02	0.00	0.00	0.01	0.00	0.00	0.00	0.03	0.01	0.00	0.00
**GH11**	0.00	0.00	0.01	0.01	0.01	0.01	0.00	0.00	0.00	0.01	0.00	0.00	0.00	0.01	0.00	0.00	0.00
**GT38**	0.00	0.00	0.00	0.00	0.00	0.00	0.00	0.00	0.00	0.00	0.00	0.00	0.00	0.00	0.00	0.00	0.00
**GT43**	0.00	0.00	0.00	0.00	0.00	0.00	0.00	0.00	0.00	0.00	0.00	0.00	0.00	0.00	0.00	0.00	0.00
**GH99**	0.02	0.00	0.00	0.01	0.02	0.01	0.01	0.06	0.00	0.02	0.02	0.03	0.00	0.00	0.01	0.02	0.01
**GH38**	0.21	0.14	0.20	0.22	0.33	0.28	0.23	0.33	0.20	0.35	0.48	0.76	0.25	0.23	0.55	0.21	0.20
**GH109**	0.16	0.22	0.08	0.27	0.35	0.23	0.45	0.52	0.41	0.39	0.24	0.27	0.33	0.35	0.39	0.29	0.24
**GH2**	2.86	3.94	3.49	3.67	5.22	5.09	4.95	6.49	3.85	5.92	4.27	5.48	5.23	6.89	6.46	4.17	4.44
**GT28**	0.47	0.39	0.74	0.49	0.48	0.53	0.38	0.36	0.45	0.48	0.39	0.46	0.46	0.45	0.56	0.51	0.43
**GH42**	0.30	0.19	0.58	0.15	0.25	0.32	0.12	0.11	0.19	0.27	0.51	0.61	0.18	0.35	0.38	0.28	0.20
**GT58**	0.00	0.00	0.00	0.00	0.00	0.00	0.00	0.00	0.00	0.00	0.00	0.00	0.00	0.00	0.00	0.00	0.00
**GH61**	0.00	0.00	0.00	0.00	0.00	0.00	0.00	0.00	0.00	0.00	0.00	0.00	0.00	0.00	0.00	0.00	0.00
**GH80**	0.00	0.00	0.00	0.00	0.00	0.00	0.00	0.00	0.00	0.00	0.00	0.00	0.00	0.00	0.00	0.00	0.00
**GH98**	0.02	0.00	0.00	0.02	0.01	0.00	0.00	0.03	0.00	0.01	0.00	0.00	0.00	0.01	0.00	0.02	0.00
**GH6**	0.00	0.00	0.00	0.00	0.00	0.00	0.00	0.00	0.00	0.00	0.00	0.00	0.00	0.00	0.00	0.00	0.00
**GT1**	0.04	0.03	0.09	0.07	0.05	0.10	0.09	0.07	0.05	0.05	0.05	0.04	0.04	0.03	0.07	0.07	0.04
**GH28**	0.42	0.92	0.51	0.76	0.96	0.79	1.15	1.39	1.03	1.10	0.63	0.99	0.99	1.33	1.22	0.91	0.76
**GT42**	0.00	0.00	0.00	0.00	0.00	0.00	0.00	0.00	0.00	0.00	0.00	0.00	0.00	0.00	0.00	0.00	0.00
**GH128**	0.00	0.00	0.00	0.00	0.00	0.00	0.00	0.00	0.00	0.00	0.00	0.00	0.00	0.00	0.00	0.00	0.00
**CE12**	0.07	0.13	0.11	0.14	0.14	0.18	0.14	0.26	0.17	0.22	0.07	0.08	0.14	0.29	0.15	0.16	0.18
**CE16**	0.00	0.00	0.00	0.00	0.00	0.00	0.00	0.00	0.00	0.00	0.00	0.00	0.00	0.00	0.00	0.00	0.00
**GT89**	0.00	0.00	0.00	0.00	0.00	0.00	0.00	0.00	0.00	0.00	0.00	0.00	0.00	0.00	0.00	0.00	0.00
**GT11**	0.02	0.04	0.05	0.04	0.06	0.07	0.07	0.06	0.07	0.07	0.02	0.10	0.10	0.06	0.09	0.08	0.05
**GT77**	0.00	0.00	0.01	0.00	0.00	0.00	0.00	0.00	0.00	0.00	0.03	0.00	0.00	0.00	0.00	0.03	0.00
**GT55**	0.00	0.00	0.00	0.00	0.00	0.00	0.00	0.00	0.00	0.00	0.00	0.00	0.00	0.00	0.00	0.00	0.00
**GT18**	0.00	0.00	0.00	0.00	0.00	0.00	0.00	0.00	0.00	0.00	0.00	0.00	0.00	0.00	0.00	0.00	0.00
**GH87**	0.00	0.00	0.00	0.01	0.00	0.00	0.00	0.00	0.00	0.01	0.00	0.00	0.00	0.00	0.01	0.01	0.00
**PL5**	0.00	0.00	0.00	0.00	0.00	0.00	0.00	0.00	0.00	0.00	0.00	0.00	0.00	0.00	0.00	0.00	0.00
**GH22**	0.00	0.00	0.00	0.00	0.00	0.00	0.01	0.00	0.00	0.00	0.00	0.00	0.00	0.00	0.00	0.00	0.00
**CE2**	0.04	0.04	0.07	0.03	0.08	0.17	0.08	0.09	0.10	0.13	0.07	0.09	0.07	0.13	0.11	0.10	0.09
**GH120**	0.06	0.00	0.09	0.05	0.02	0.06	0.02	0.00	0.00	0.02	0.10	0.11	0.04	0.01	0.15	0.05	0.03
**GH73**	0.60	0.62	0.74	0.50	0.47	0.80	0.50	0.65	0.59	0.57	0.72	1.03	0.57	0.66	0.62	0.54	0.57
**GT17**	0.00	0.00	0.00	0.00	0.01	0.01	0.01	0.00	0.00	0.00	0.00	0.00	0.00	0.00	0.00	0.00	0.00
**GH124**	0.00	0.00	0.00	0.00	0.00	0.00	0.00	0.00	0.00	0.00	0.00	0.00	0.00	0.00	0.00	0.00	0.00
**GH54**	0.00	0.00	0.00	0.00	0.00	0.00	0.00	0.00	0.00	0.00	0.00	0.00	0.00	0.00	0.00	0.00	0.00
**GH100**	0.00	0.00	0.00	0.00	0.00	0.00	0.00	0.00	0.00	0.00	0.00	0.00	0.00	0.00	0.00	0.00	0.00
**GH113**	0.02	0.01	0.09	0.04	0.03	0.05	0.02	0.00	0.01	0.03	0.06	0.05	0.03	0.07	0.02	0.04	0.02
**GT5**	0.58	0.45	0.69	0.49	0.48	0.67	0.31	0.34	0.46	0.46	0.52	0.64	0.52	0.55	0.56	0.55	0.47
**GH94**	0.41	0.24	0.94	0.37	0.47	0.84	0.13	0.23	0.22	0.85	0.60	0.52	0.40	0.69	0.54	0.71	0.43
**GT66**	0.00	0.00	0.00	0.00	0.01	0.00	0.00	0.00	0.00	0.00	0.00	0.00	0.00	0.00	0.00	0.00	0.00
**GT94**	0.00	0.00	0.00	0.00	0.00	0.00	0.00	0.00	0.00	0.00	0.00	0.00	0.00	0.00	0.00	0.00	0.00
**GH58**	0.00	0.00	0.00	0.01	0.00	0.00	0.00	0.00	0.00	0.00	0.00	0.00	0.00	0.00	0.00	0.02	0.00
**GH1**	0.87	0.33	1.00	0.58	0.57	0.97	0.09	0.20	0.81	0.49	0.39	0.43	0.51	0.25	0.61	0.65	0.42
**PL22**	0.00	0.00	0.00	0.00	0.00	0.00	0.00	0.00	0.00	0.00	0.00	0.00	0.00	0.00	0.00	0.01	0.00

CAZY_group	AM-AD-18	AM-AD-19	AM-AD-20	AM-AD-21	AM-AD-22	AM-AD-23	AM-AD-24	AM-AD-25	AM-AD-26	AM-AD-27	AM-AD-28	AM-AD-29	AM-AD-3	AM-AD-30	AM-AD-31	AM-AD-32	AM-AD-33
**GT76**	0.00	0.00	0.01	0.00	0.00	0.00	0.00	0.00	0.00	0.00	0.00	0.00	0.00	0.00	0.00	0.00	0.00
**GT32**	0.04	0.06	0.07	0.08	0.14	0.11	0.08	0.11	0.06	0.12	0.15	0.13	0.21	0.12	0.11	0.06	0.11
**GT47**	0.06	0.07	0.07	0.08	0.09	0.04	0.04	0.01	0.07	0.08	0.09	0.10	0.08	0.12	0.11	0.10	0.07
**GH84**	0.09	0.09	0.05	0.09	0.11	0.19	0.11	0.14	0.06	0.11	0.26	0.25	0.09	0.13	0.13	0.09	0.10
**GH83**	0.00	0.00	0.00	0.00	0.00	0.00	0.00	0.00	0.00	0.00	0.00	0.00	0.00	0.00	0.00	0.00	0.00
**GT59**	0.00	0.00	0.00	0.00	0.00	0.00	0.00	0.00	0.00	0.00	0.00	0.00	0.00	0.00	0.00	0.00	0.00
**GH34**	0.00	0.00	0.00	0.00	0.00	0.00	0.00	0.00	0.00	0.00	0.00	0.00	0.00	0.00	0.00	0.00	0.00
**GT14**	0.00	0.00	0.02	0.02	0.03	0.04	0.03	0.04	0.01	0.05	0.07	0.06	0.04	0.05	0.04	0.02	0.03
**GH47**	0.00	0.01	0.01	0.00	0.00	0.01	0.00	0.00	0.00	0.00	0.00	0.00	0.00	0.00	0.00	0.00	0.00
**GH97**	0.31	0.88	0.32	0.76	1.12	0.83	1.03	1.87	0.99	1.22	0.79	1.21	1.30	1.24	1.33	0.64	0.81
**GH50**	0.02	0.06	0.00	0.03	0.06	0.06	0.04	0.11	0.01	0.13	0.01	0.06	0.06	0.09	0.11	0.01	0.08
**GT26**	0.16	0.16	0.30	0.18	0.19	0.27	0.19	0.16	0.20	0.24	0.19	0.32	0.18	0.25	0.33	0.21	0.22
**GH18**	0.67	0.51	0.59	0.65	0.80	0.77	0.51	1.18	0.41	0.89	0.85	0.96	0.70	0.79	0.75	0.58	0.61
**GH37**	0.00	0.03	0.00	0.01	0.01	0.01	0.02	0.00	0.01	0.02	0.00	0.00	0.01	0.00	0.05	0.05	0.01
**PL13**	0.01	0.05	0.01	0.03	0.06	0.05	0.03	0.11	0.07	0.06	0.03	0.05	0.07	0.08	0.09	0.02	0.03
**GH27**	0.13	0.16	0.13	0.21	0.27	0.27	0.28	0.27	0.28	0.41	0.28	0.43	0.35	0.35	0.47	0.25	0.23
**GT54**	0.00	0.00	0.00	0.00	0.00	0.00	0.00	0.00	0.00	0.00	0.00	0.00	0.00	0.00	0.00	0.00	0.00
**GT91**	0.00	0.00	0.00	0.00	0.00	0.00	0.00	0.00	0.00	0.00	0.00	0.00	0.00	0.00	0.00	0.00	0.00
**GT72**	0.00	0.00	0.00	0.00	0.00	0.00	0.00	0.00	0.00	0.00	0.00	0.00	0.00	0.00	0.00	0.00	0.00
**GH67**	0.01	0.07	0.05	0.09	0.05	0.07	0.11	0.11	0.12	0.15	0.06	0.05	0.09	0.14	0.10	0.07	0.06
**GH12**	0.00	0.00	0.00	0.00	0.00	0.00	0.00	0.00	0.00	0.01	0.00	0.00	0.00	0.00	0.00	0.00	0.00
**GH91**	0.01	0.01	0.01	0.03	0.04	0.01	0.02	0.09	0.01	0.05	0.02	0.01	0.01	0.09	0.05	0.01	0.01
**GT69**	0.00	0.00	0.00	0.00	0.00	0.00	0.00	0.00	0.00	0.00	0.00	0.00	0.00	0.00	0.00	0.00	0.00
**GH106**	0.12	0.25	0.06	0.19	0.29	0.28	0.38	0.59	0.28	0.37	0.14	0.24	0.33	0.45	0.52	0.26	0.29
**GT62**	0.00	0.00	0.00	0.00	0.00	0.00	0.00	0.00	0.00	0.00	0.00	0.00	0.00	0.00	0.00	0.00	0.00
**GH17**	0.00	0.00	0.00	0.00	0.01	0.00	0.00	0.00	0.00	0.00	0.00	0.00	0.00	0.00	0.00	0.00	0.00
**GH88**	0.22	0.41	0.17	0.37	0.45	0.33	0.47	0.63	0.41	0.46	0.23	0.46	0.41	0.62	0.65	0.29	0.37
**CE5**	0.00	0.00	0.00	0.00	0.00	0.00	0.00	0.00	0.00	0.00	0.00	0.00	0.00	0.00	0.00	0.00	0.00
**GT8**	0.06	0.09	0.12	0.06	0.07	0.16	0.09	0.04	0.17	0.16	0.17	0.29	0.12	0.07	0.18	0.13	0.10
**GT50**	0.00	0.00	0.00	0.00	0.00	0.00	0.00	0.00	0.00	0.00	0.00	0.00	0.00	0.00	0.00	0.00	0.00
**GT20**	0.05	0.10	0.02	0.10	0.11	0.06	0.11	0.20	0.12	0.10	0.02	0.03	0.08	0.15	0.12	0.10	0.12
**PL9**	0.05	0.08	0.10	0.15	0.10	0.05	0.02	0.17	0.12	0.12	0.06	0.06	0.10	0.21	0.11	0.07	0.08
**GH55**	0.04	0.03	0.01	0.02	0.05	0.04	0.02	0.01	0.01	0.08	0.06	0.08	0.02	0.05	0.01	0.05	0.02
**GH64**	0.00	0.00	0.01	0.00	0.00	0.00	0.00	0.00	0.00	0.00	0.00	0.00	0.00	0.00	0.00	0.00	0.00
**GH127**	0.27	0.27	0.29	0.19	0.39	0.38	0.42	0.47	0.19	0.41	0.39	0.47	0.39	0.47	0.49	0.41	0.33
**GH103**	0.00	0.00	0.01	0.00	0.00	0.00	0.00	0.00	0.00	0.00	0.00	0.00	0.00	0.00	0.01	0.05	0.00
**GH3**	2.11	2.34	2.72	2.79	3.54	3.39	2.78	3.50	2.73	3.80	3.48	4.75	2.91	4.40	4.16	2.74	3.02
**GT45**	0.00	0.00	0.00	0.00	0.00	0.00	0.00	0.00	0.00	0.00	0.00	0.00	0.00	0.00	0.00	0.00	0.00
**GT74**	0.00	0.00	0.00	0.00	0.00	0.00	0.00	0.00	0.00	0.00	0.00	0.00	0.00	0.00	0.00	0.00	0.00
**GT90**	0.00	0.00	0.00	0.00	0.00	0.00	0.01	0.00	0.00	0.00	0.00	0.00	0.00	0.01	0.00	0.00	0.00
**GH116**	0.04	0.04	0.02	0.09	0.08	0.02	0.07	0.06	0.12	0.06	0.14	0.15	0.07	0.12	0.09	0.04	0.06
**GH31**	0.83	0.79	0.99	0.96	1.28	1.30	0.95	1.56	0.90	1.37	1.24	1.59	1.07	1.38	1.46	1.03	1.01
**GT12**	0.00	0.00	0.00	0.00	0.00	0.00	0.00	0.00	0.00	0.00	0.00	0.00	0.00	0.00	0.00	0.00	0.00
**CE1**	0.24	0.30	0.20	0.43	0.42	0.33	0.43	0.46	0.35	0.43	0.48	0.60	0.45	0.51	0.53	0.26	0.39
**GH121**	0.00	0.00	0.01	0.00	0.01	0.00	0.00	0.00	0.00	0.00	0.05	0.10	0.00	0.00	0.02	0.00	0.00
**GH79**	0.01	0.00	0.01	0.01	0.00	0.04	0.00	0.00	0.00	0.02	0.01	0.03	0.00	0.00	0.00	0.01	0.01
**GT73**	0.00	0.00	0.00	0.00	0.00	0.00	0.00	0.00	0.00	0.00	0.00	0.00	0.00	0.00	0.00	0.01	0.00
**GH77**	0.65	0.59	0.98	0.63	0.66	0.93	0.46	0.53	0.58	0.71	0.79	0.87	0.70	0.67	0.73	0.62	0.65
**PL17**	0.01	0.01	0.00	0.01	0.01	0.00	0.00	0.00	0.00	0.01	0.00	0.00	0.01	0.01	0.01	0.01	0.00
**PL8**	0.14	0.30	0.06	0.27	0.40	0.19	0.33	0.75	0.40	0.45	0.15	0.28	0.26	0.37	0.43	0.14	0.28

CAZY_group	AM-AD-18	AM-AD-19	AM-AD-20	AM-AD-21	AM-AD-22	AM-AD-23	AM-AD-24	AM-AD-25	AM-AD-26	AM-AD-27	AM-AD-28	AM-AD-29	AM-AD-3	AM-AD-30	AM-AD-31	AM-AD-32	AM-AD-33
**CE9**	0.41	0.42	0.45	0.36	0.42	0.54	0.25	0.53	0.28	0.41	0.43	0.50	0.39	0.56	0.43	0.33	0.25
**CE15**	0.03	0.03	0.01	0.01	0.01	0.03	0.01	0.01	0.02	0.02	0.00	0.04	0.02	0.01	0.02	0.00	0.03
**GT79**	0.00	0.00	0.00	0.00	0.00	0.00	0.00	0.00	0.00	0.00	0.00	0.00	0.00	0.00	0.00	0.00	0.00
**GT10**	0.00	0.01	0.00	0.00	0.01	0.00	0.03	0.00	0.01	0.02	0.01	0.01	0.00	0.00	0.01	0.02	0.01
**GH119**	0.00	0.00	0.00	0.00	0.00	0.00	0.00	0.00	0.00	0.00	0.00	0.00	0.00	0.00	0.00	0.00	0.00
**GH16**	0.09	0.18	0.15	0.15	0.30	0.22	0.23	0.43	0.16	0.32	0.15	0.25	0.28	0.32	0.34	0.26	0.14
**GH43**	1.04	1.93	1.36	1.68	2.53	2.24	2.35	3.69	1.50	2.96	2.63	3.64	2.83	3.72	3.22	2.06	2.14
**GH51**	0.42	0.48	0.46	0.46	0.67	0.76	0.55	0.72	0.27	0.61	0.63	0.89	0.65	0.94	0.91	0.73	0.55
**GT35**	1.11	0.80	1.67	1.08	1.05	1.36	0.62	0.62	0.64	1.10	1.21	1.29	1.15	1.19	1.26	1.23	0.96
**GT25**	0.00	0.00	0.03	0.01	0.00	0.01	0.00	0.01	0.00	0.00	0.02	0.04	0.00	0.00	0.00	0.02	0.00
**GT2**	3.93	4.34	4.70	4.79	5.25	5.76	4.58	4.48	5.07	5.61	5.48	6.38	5.47	5.40	6.01	4.98	4.94
**GT51**	0.70	1.01	1.00	0.97	1.08	1.13	1.11	1.09	1.21	1.17	0.96	1.39	1.01	1.21	1.23	1.13	1.08
**GH107**	0.00	0.00	0.00	0.00	0.00	0.00	0.00	0.00	0.00	0.00	0.00	0.00	0.00	0.00	0.00	0.00	0.00
**GH7**	0.00	0.00	0.00	0.00	0.00	0.00	0.00	0.00	0.00	0.00	0.00	0.00	0.00	0.00	0.00	0.00	0.00
**GT7**	0.00	0.00	0.00	0.00	0.00	0.00	0.00	0.00	0.00	0.00	0.00	0.00	0.00	0.00	0.00	0.00	0.00
**GT36**	0.00	0.00	0.00	0.00	0.00	0.00	0.00	0.00	0.00	0.00	0.00	0.00	0.00	0.00	0.00	0.00	0.00
**PL7**	0.00	0.00	0.01	0.00	0.00	0.00	0.00	0.00	0.00	0.00	0.00	0.00	0.00	0.00	0.00	0.01	0.00
**GH110**	0.05	0.12	0.03	0.13	0.11	0.12	0.11	0.23	0.09	0.15	0.16	0.18	0.16	0.18	0.13	0.13	0.07
**GT21**	0.00	0.00	0.00	0.00	0.00	0.00	0.00	0.00	0.00	0.00	0.00	0.00	0.00	0.00	0.00	0.00	0.00
**GH62**	0.00	0.00	0.00	0.00	0.00	0.00	0.00	0.00	0.00	0.00	0.00	0.00	0.00	0.00	0.00	0.00	0.00
**GH125**	0.10	0.18	0.07	0.19	0.27	0.16	0.20	0.32	0.19	0.25	0.26	0.41	0.18	0.29	0.27	0.10	0.28
**GH117**	0.03	0.04	0.01	0.08	0.07	0.10	0.13	0.19	0.12	0.14	0.05	0.13	0.07	0.17	0.15	0.10	0.13
**GH68**	0.00	0.01	0.00	0.00	0.00	0.00	0.00	0.00	0.00	0.00	0.00	0.00	0.00	0.00	0.00	0.00	0.00
**GH85**	0.01	0.01	0.02	0.00	0.03	0.02	0.02	0.00	0.00	0.02	0.07	0.04	0.06	0.03	0.02	0.00	0.01
**GH92**	0.80	1.67	0.46	1.66	2.12	1.50	2.32	3.82	1.80	2.34	1.76	2.68	2.20	2.66	2.50	1.38	2.00
**GH40**	0.00	0.00	0.00	0.00	0.00	0.00	0.00	0.00	0.00	0.00	0.00	0.00	0.00	0.00	0.00	0.00	0.00
**GT49**	0.00	0.00	0.00	0.00	0.00	0.00	0.00	0.00	0.00	0.00	0.00	0.00	0.00	0.00	0.00	0.00	0.00
**GH112**	0.29	0.13	0.24	0.15	0.19	0.30	0.05	0.09	0.07	0.25	0.22	0.40	0.12	0.15	0.20	0.32	0.12
**GT67**	0.00	0.00	0.00	0.00	0.00	0.00	0.00	0.00	0.00	0.00	0.00	0.00	0.00	0.00	0.00	0.00	0.00
**CE8**	0.06	0.28	0.20	0.22	0.24	0.32	0.25	0.24	0.26	0.32	0.12	0.15	0.25	0.38	0.26	0.23	0.19
**GT86**	0.00	0.00	0.00	0.00	0.00	0.00	0.00	0.00	0.00	0.00	0.00	0.00	0.00	0.00	0.00	0.00	0.00
**GH101**	0.01	0.00	0.06	0.00	0.01	0.03	0.00	0.00	0.00	0.00	0.02	0.14	0.00	0.00	0.01	0.00	0.00
**GH81**	0.01	0.00	0.01	0.00	0.02	0.01	0.00	0.00	0.00	0.01	0.01	0.03	0.00	0.00	0.00	0.01	0.00
**GT93**	0.00	0.00	0.00	0.00	0.00	0.00	0.00	0.00	0.00	0.00	0.00	0.00	0.00	0.00	0.00	0.00	0.00
**GT63**	0.00	0.00	0.00	0.00	0.00	0.00	0.00	0.00	0.00	0.00	0.00	0.00	0.00	0.00	0.00	0.00	0.00
**CE11**	0.16	0.26	0.17	0.20	0.24	0.27	0.34	0.23	0.36	0.24	0.27	0.38	0.26	0.26	0.30	0.28	0.24
**GT19**	0.06	0.13	0.07	0.10	0.13	0.13	0.23	0.24	0.17	0.15	0.12	0.15	0.18	0.17	0.17	0.12	0.15
**GT33**	0.00	0.00	0.00	0.00	0.00	0.00	0.00	0.00	0.00	0.00	0.00	0.00	0.00	0.00	0.00	0.00	0.00
**GH21**	0.00	0.00	0.00	0.00	0.00	0.00	0.00	0.00	0.00	0.00	0.00	0.00	0.00	0.00	0.00	0.00	0.00
**GT68**	0.00	0.00	0.00	0.00	0.00	0.00	0.00	0.00	0.00	0.00	0.00	0.00	0.00	0.00	0.00	0.00	0.00
**GH89**	0.16	0.22	0.09	0.20	0.23	0.30	0.33	0.46	0.28	0.25	0.20	0.23	0.23	0.43	0.35	0.20	0.15
**GT82**	0.00	0.00	0.00	0.00	0.00	0.00	0.00	0.00	0.00	0.00	0.00	0.00	0.00	0.00	0.00	0.00	0.00
**GH30**	0.16	0.26	0.17	0.19	0.35	0.23	0.23	0.33	0.20	0.29	0.35	0.46	0.23	0.31	0.37	0.19	0.18
**GH20**	0.84	1.28	0.44	0.99	1.20	1.31	1.55	2.77	1.30	1.87	1.16	1.64	1.51	2.06	1.86	0.94	1.48
**GH52**	0.00	0.00	0.00	0.00	0.00	0.00	0.00	0.00	0.00	0.00	0.00	0.00	0.00	0.00	0.00	0.00	0.00
**GT84**	0.02	0.00	0.01	0.03	0.04	0.04	0.00	0.00	0.02	0.00	0.01	0.03	0.08	0.00	0.02	0.00	0.02
**GT15**	0.00	0.00	0.00	0.00	0.00	0.00	0.00	0.00	0.00	0.00	0.00	0.00	0.00	0.00	0.00	0.00	0.00
**GH93**	0.00	0.00	0.00	0.00	0.01	0.02	0.01	0.00	0.00	0.01	0.02	0.00	0.01	0.02	0.01	0.00	0.00
**PL16**	0.00	0.00	0.00	0.00	0.00	0.00	0.00	0.00	0.00	0.00	0.00	0.00	0.00	0.00	0.00	0.00	0.00
**GH26**	0.08	0.08	0.12	0.13	0.15	0.18	0.12	0.03	0.05	0.23	0.20	0.23	0.21	0.14	0.22	0.27	0.12

CAZY_group	AM-AD-18	AM-AD-19	AM-AD-20	AM-AD-21	AM-AD-22	AM-AD-23	AM-AD-24	AM-AD-25	AM-AD-26	AM-AD-27	AM-AD-28	AM-AD-29	AM-AD-3	AM-AD-30	AM-AD-31	AM-AD-32	AM-AD-33
GT75	0.00	0.00	0.00	0.00	0.00	0.00	0.00	0.00	0.00	0.00	0.00	0.00	0.00	0.00	0.00	0.00	0.00
GH53	0.06	0.05	0.20	0.10	0.14	0.23	0.03	0.11	0.15	0.20	0.15	0.27	0.12	0.25	0.20	0.29	0.19
GT88	0.00	0.00	0.00	0.00	0.00	0.00	0.00	0.00	0.00	0.00	0.00	0.00	0.00	0.00	0.00	0.00	0.00
GT24	0.00	0.00	0.00	0.00	0.00	0.00	0.00	0.00	0.00	0.00	0.00	0.00	0.00	0.00	0.00	0.00	0.00
GT81	0.00	0.00	0.00	0.00	0.00	0.00	0.00	0.00	0.00	0.00	0.00	0.00	0.00	0.00	0.00	0.00	0.00
GH4	0.27	0.09	0.17	0.09	0.13	0.11	0.02	0.09	0.22	0.16	0.21	0.28	0.13	0.08	0.18	0.28	0.11
GT78	0.00	0.00	0.00	0.00	0.00	0.00	0.00	0.00	0.00	0.00	0.00	0.00	0.00	0.00	0.00	0.00	0.00
GH8	0.02	0.06	0.05	0.01	0.05	0.08	0.03	0.01	0.04	0.11	0.08	0.09	0.07	0.13	0.09	0.07	0.06
GH70	0.01	0.00	0.00	0.00	0.00	0.00	0.00	0.00	0.00	0.00	0.00	0.01	0.00	0.00	0.00	0.00	0.00
GH122	0.00	0.00	0.00	0.00	0.00	0.00	0.00	0.00	0.00	0.00	0.00	0.00	0.00	0.00	0.00	0.00	0.00
GH74	0.02	0.01	0.03	0.01	0.01	0.04	0.03	0.00	0.02	0.06	0.05	0.00	0.04	0.07	0.03	0.05	0.01
GT41	0.01	0.01	0.05	0.02	0.01	0.04	0.01	0.01	0.02	0.01	0.00	0.00	0.00	0.00	0.00	0.01	0.00
PL1	0.18	0.25	0.28	0.27	0.31	0.32	0.23	0.76	0.25	0.33	0.17	0.19	0.34	0.43	0.42	0.29	0.26
GH15	0.03	0.07	0.00	0.05	0.06	0.03	0.09	0.07	0.10	0.08	0.00	0.01	0.05	0.07	0.08	0.03	0.08
GH32	0.55	0.35	0.75	0.44	0.61	0.49	0.38	0.60	0.31	0.47	0.64	0.69	0.48	0.56	0.75	0.43	0.43
GH44	0.01	0.01	0.01	0.01	0.01	0.00	0.00	0.00	0.00	0.00	0.01	0.00	0.00	0.01	0.01	0.00	0.00
GT6	0.00	0.01	0.01	0.01	0.00	0.01	0.00	0.00	0.01	0.01	0.00	0.00	0.00	0.00	0.02	0.00	0.00
GH13	3.19	2.74	5.05	3.06	3.48	4.31	2.21	2.27	3.10	3.53	4.20	5.57	3.39	3.45	4.35	3.21	3.20
GT80	0.02	0.01	0.00	0.02	0.02	0.03	0.05	0.01	0.06	0.03	0.00	0.04	0.02	0.02	0.04	0.05	0.04
GH71	0.00	0.00	0.00	0.00	0.00	0.00	0.00	0.00	0.00	0.00	0.00	0.00	0.00	0.00	0.00	0.00	0.00
GH63	0.06	0.10	0.03	0.07	0.12	0.13	0.13	0.11	0.12	0.11	0.20	0.24	0.17	0.18	0.21	0.10	0.10
PL21	0.00	0.00	0.00	0.04	0.03	0.04	0.04	0.09	0.07	0.02	0.00	0.00	0.03	0.00	0.01	0.00	0.00
GH65	0.11	0.25	0.25	0.13	0.19	0.17	0.18	0.09	0.14	0.27	0.14	0.36	0.19	0.20	0.19	0.09	0.19
CE4	0.80	0.59	0.85	0.64	0.71	0.91	0.54	0.52	0.62	0.65	0.60	0.71	0.67	0.61	0.77	0.73	0.52
CE14	0.02	0.01	0.00	0.01	0.03	0.01	0.02	0.00	0.00	0.03	0.05	0.04	0.02	0.01	0.01	0.03	0.01
GH115	0.08	0.27	0.15	0.24	0.30	0.35	0.34	0.49	0.27	0.46	0.17	0.31	0.36	0.55	0.42	0.33	0.28
GH104	0.00	0.00	0.00	0.00	0.00	0.01	0.00	0.00	0.00	0.00	0.01	0.00	0.00	0.00	0.00	0.02	0.00
GH111	0.00	0.00	0.00	0.00	0.00	0.00	0.00	0.00	0.00	0.00	0.00	0.00	0.00	0.00	0.00	0.00	0.00
GH95	0.33	0.49	0.36	0.45	0.79	0.73	0.82	1.21	0.51	0.71	0.66	0.76	0.78	1.01	0.78	0.55	0.57
GT70	0.00	0.00	0.00	0.00	0.00	0.00	0.00	0.00	0.00	0.00	0.00	0.00	0.00	0.00	0.00	0.00	0.00
CE10	0.14	0.18	0.09	0.17	0.17	0.20	0.23	0.29	0.24	0.24	0.24	0.34	0.20	0.23	0.24	0.16	0.20
GH10	0.04	0.13	0.11	0.08	0.10	0.11	0.13	0.07	0.06	0.22	0.15	0.25	0.17	0.26	0.13	0.10	0.12
GH130	0.18	0.25	0.21	0.34	0.48	0.36	0.34	0.59	0.31	0.49	0.45	0.51	0.56	0.47	0.61	0.36	0.38
PL20	0.00	0.00	0.00	0.00	0.00	0.00	0.00	0.00	0.00	0.00	0.00	0.00	0.00	0.00	0.00	0.00	0.00
GH69	0.00	0.00	0.00	0.00	0.00	0.00	0.00	0.00	0.00	0.00	0.00	0.00	0.00	0.00	0.00	0.00	0.00
PL2	0.00	0.00	0.00	0.00	0.00	0.00	0.00	0.00	0.00	0.00	0.00	0.00	0.00	0.00	0.00	0.00	0.00
GH49	0.00	0.00	0.00	0.00	0.00	0.00	0.00	0.00	0.00	0.00	0.00	0.00	0.00	0.00	0.00	0.00	0.00
GH25	0.35	0.44	0.67	0.47	0.45	0.51	0.24	0.34	0.42	0.45	0.50	0.60	0.40	0.47	0.42	0.42	0.38
GH59	0.01	0.01	0.02	0.02	0.03	0.06	0.03	0.03	0.00	0.05	0.03	0.00	0.06	0.09	0.03	0.03	0.04
GH35	0.15	0.34	0.12	0.36	0.41	0.43	0.44	0.86	0.28	0.60	0.31	0.46	0.50	0.63	0.48	0.42	0.32
GT39	0.01	0.01	0.01	0.00	0.01	0.00	0.00	0.00	0.00	0.02	0.00	0.03	0.00	0.01	0.01	0.01	0.01
GT57	0.00	0.00	0.00	0.00	0.00	0.00	0.00	0.00	0.00	0.00	0.00	0.00	0.00	0.00	0.00	0.00	0.00
GH129	0.03	0.00	0.01	0.00	0.02	0.00	0.00	0.00	0.00	0.01	0.02	0.03	0.00	0.00	0.03	0.00	0.00
GT22	0.00	0.00	0.00	0.00	0.00	0.00	0.00	0.00	0.00	0.00	0.00	0.00	0.00	0.00	0.01	0.00	0.00
PL12	0.10	0.09	0.03	0.14	0.18	0.10	0.12	0.29	0.19	0.14	0.09	0.11	0.14	0.16	0.21	0.07	0.17
PL18	0.00	0.00	0.00	0.00	0.00	0.00	0.00	0.00	0.00	0.00	0.00	0.00	0.00	0.00	0.00	0.00	0.00
GT92	0.00	0.00	0.00	0.00	0.00	0.00	0.00	0.00	0.00	0.00	0.00	0.00	0.00	0.00	0.00	0.00	0.00
GT44	0.00	0.00	0.00	0.00	0.00	0.00	0.00	0.00	0.00	0.00	0.00	0.00	0.00	0.00	0.00	0.00	0.00
GT29	0.00	0.00	0.00	0.00	0.00	0.00	0.00	0.00	0.00	0.00	0.00	0.00	0.00	0.00	0.00	0.00	0.00
GT13	0.00	0.00	0.00	0.00	0.00	0.00	0.00	0.00	0.00	0.00	0.00	0.00	0.00	0.00	0.00	0.00	0.00

CAZY_group	AM-AD-18	AM-AD-19	AM-AD-20	AM-AD-21	AM-AD-22	AM-AD-23	AM-AD-24	AM-AD-25	AM-AD-26	AM-AD-27	AM-AD-28	AM-AD-29	AM-AD-3	AM-AD-30	AM-AD-31	AM-AD-32	AM-AD-33
**GT52**	0.00	0.00	0.00	0.00	0.00	0.00	0.00	0.00	0.00	0.00	0.00	0.00	0.00	0.00	0.00	0.00	0.00
**GT64**	0.00	0.00	0.00	0.00	0.00	0.00	0.00	0.00	0.00	0.00	0.00	0.00	0.00	0.00	0.00	0.00	0.00
**GT85**	0.00	0.00	0.00	0.00	0.00	0.00	0.00	0.00	0.00	0.00	0.00	0.00	0.00	0.00	0.00	0.00	0.00
**GT16**	0.00	0.00	0.00	0.00	0.00	0.00	0.00	0.00	0.00	0.00	0.00	0.00	0.00	0.00	0.00	0.00	0.00
**GH56**	0.00	0.00	0.00	0.00	0.00	0.00	0.00	0.00	0.00	0.00	0.00	0.00	0.00	0.00	0.00	0.00	0.00
**GT60**	0.00	0.00	0.00	0.00	0.00	0.00	0.00	0.00	0.00	0.00	0.00	0.00	0.00	0.00	0.00	0.00	0.00
**GH41**	0.00	0.00	0.00	0.00	0.00	0.00	0.00	0.00	0.00	0.00	0.00	0.00	0.00	0.00	0.00	0.00	0.00
**CE7**	0.09	0.17	0.05	0.07	0.14	0.08	0.10	0.16	0.07	0.10	0.05	0.11	0.09	0.24	0.21	0.08	0.13
**GH39**	0.04	0.05	0.03	0.06	0.02	0.06	0.03	0.06	0.01	0.05	0.09	0.09	0.02	0.10	0.04	0.08	0.06
**GT30**	0.16	0.21	0.17	0.23	0.29	0.25	0.41	0.42	0.42	0.31	0.28	0.28	0.29	0.26	0.30	0.27	0.30
**PL15**	0.06	0.19	0.03	0.08	0.17	0.11	0.13	0.32	0.06	0.15	0.08	0.13	0.15	0.21	0.17	0.10	0.11
**GH102**	0.01	0.01	0.03	0.01	0.01	0.04	0.01	0.00	0.01	0.01	0.01	0.01	0.00	0.00	0.00	0.01	0.00

CAZY_group	AM-AD-34	AM-AD-35	AM-AD-37	AM-AD-38	AM-AD-39	AM-AD-4	AM-AD-40	AM-AD-41	AM-AD-42	AM-AD-43	AM-AD-44	AM-AD-45	AM-AD-46	AM-AD-47	AM-AD-48	AM-AD-49	AM-AD-5
**GH29**	0.87	0.93	0.64	1.00	0.87	0.73	0.78	0.64	0.95	0.79	0.81	0.72	0.85	1.12	0.89	0.66	0.90
**PL4**	0.00	0.00	0.00	0.02	0.00	0.00	0.00	0.00	0.00	0.00	0.00	0.00	0.00	0.00	0.01	0.00	0.00
**GH23**	1.27	1.19	1.14	1.54	1.85	1.39	1.54	1.04	1.46	1.33	1.52	1.13	1.34	1.61	1.27	1.22	1.35
**CE6**	0.06	0.03	0.05	0.01	0.04	0.02	0.01	0.01	0.00	0.05	0.02	0.05	0.07	0.07	0.07	0.03	0.08
**GH72**	0.16	0.18	0.15	0.27	0.19	0.12	0.16	0.13	0.24	0.17	0.11	0.20	0.14	0.19	0.14	0.15	0.15
**GH114**	0.00	0.00	0.00	0.00	0.00	0.00	0.00	0.00	0.00	0.00	0.00	0.00	0.00	0.00	0.00	0.00	0.00
**GH78**	0.98	1.31	0.78	0.96	1.50	1.10	0.88	0.96	0.86	0.86	0.86	0.90	1.03	1.23	1.28	0.77	0.92
**PL19**	0.00	0.00	0.00	0.00	0.00	0.00	0.00	0.00	0.00	0.00	0.00	0.00	0.00	0.00	0.00	0.00	0.00
**GT27**	0.00	0.00	0.00	0.00	0.00	0.00	0.00	0.00	0.00	0.00	0.00	0.00	0.00	0.00	0.00	0.00	0.00
**GH5**	0.31	0.40	0.33	0.56	0.56	0.42	0.40	0.70	0.46	0.60	0.21	0.42	0.57	0.59	0.63	0.45	0.72
**GT40**	0.00	0.00	0.00	0.00	0.00	0.00	0.00	0.00	0.00	0.00	0.00	0.00	0.00	0.00	0.00	0.00	0.00
**CE13**	0.00	0.00	0.00	0.01	0.00	0.00	0.00	0.00	0.00	0.00	0.00	0.00	0.00	0.00	0.01	0.00	0.00
**GT37**	0.00	0.00	0.00	0.00	0.00	0.00	0.00	0.00	0.00	0.00	0.00	0.00	0.00	0.00	0.00	0.00	0.00
**GH33**	0.33	0.28	0.23	0.26	0.43	0.34	0.30	0.18	0.24	0.22	0.33	0.19	0.20	0.39	0.24	0.16	0.37
**GT65**	0.00	0.00	0.00	0.00	0.00	0.00	0.00	0.00	0.00	0.00	0.00	0.00	0.00	0.00	0.00	0.00	0.00
**GH86**	0.00	0.00	0.01	0.01	0.02	0.01	0.00	0.00	0.00	0.00	0.00	0.00	0.01	0.00	0.00	0.01	0.00
**GH123**	0.10	0.18	0.17	0.11	0.21	0.10	0.17	0.14	0.19	0.15	0.12	0.16	0.12	0.18	0.11	0.07	0.17
**GH96**	0.00	0.00	0.00	0.00	0.00	0.00	0.00	0.00	0.00	0.00	0.00	0.00	0.00	0.00	0.00	0.00	0.00
**GH14**	0.00	0.00	0.00	0.00	0.00	0.01	0.00	0.00	0.00	0.00	0.00	0.00	0.00	0.00	0.00	0.00	0.00
**CE3**	0.00	0.00	0.00	0.00	0.00	0.00	0.00	0.00	0.00	0.00	0.00	0.00	0.00	0.00	0.00	0.00	0.00
**PL10**	0.05	0.15	0.02	0.12	0.10	0.08	0.19	0.09	0.10	0.06	0.11	0.10	0.06	0.13	0.12	0.04	0.14
**GT48**	0.00	0.00	0.00	0.00	0.00	0.00	0.00	0.00	0.00	0.00	0.00	0.00	0.00	0.00	0.00	0.00	0.00
**PL6**	0.00	0.01	0.02	0.00	0.00	0.00	0.00	0.00	0.00	0.00	0.00	0.00	0.01	0.00	0.01	0.00	0.02
**GT83**	0.17	0.09	0.05	0.13	0.19	0.16	0.14	0.07	0.10	0.10	0.12	0.25	0.13	0.12	0.13	0.06	0.17
**GH126**	0.00	0.00	0.00	0.00	0.00	0.00	0.00	0.00	0.00	0.00	0.00	0.00	0.00	0.00	0.00	0.00	0.00
**GH9**	0.12	0.18	0.10	0.16	0.35	0.32	0.09	0.09	0.24	0.21	0.09	0.09	0.12	0.17	0.24	0.20	0.12
**GH75**	0.00	0.00	0.00	0.00	0.00	0.00	0.00	0.00	0.00	0.00	0.00	0.00	0.00	0.00	0.00	0.00	0.00
**GT71**	0.00	0.00	0.00	0.00	0.00	0.00	0.00	0.00	0.00	0.00	0.00	0.00	0.00	0.00	0.00	0.00	0.00
**GH46**	0.00	0.00	0.00	0.01	0.00	0.00	0.00	0.00	0.00	0.00	0.00	0.00	0.00	0.00	0.00	0.00	0.00
**GT61**	0.00	0.00	0.00	0.00	0.00	0.00	0.00	0.00	0.00	0.00	0.00	0.00	0.00	0.00	0.00	0.00	0.00
**GT3**	0.19	0.38	0.21	0.40	0.31	0.38	0.52	0.22	0.49	0.30	0.20	0.29	0.28	0.38	0.30	0.17	0.42
**PL3**	0.00	0.00	0.00	0.00	0.00	0.00	0.00	0.00	0.00	0.00	0.00	0.00	0.00	0.00	0.00	0.00	0.00
**GH108**	0.03	0.06	0.03	0.03	0.04	0.04	0.01	0.05	0.03	0.03	0.02	0.00	0.02	0.04	0.02	0.04	0.03
**GT56**	0.00	0.00	0.00	0.00	0.00	0.00	0.00	0.00	0.00	0.00	0.01	0.00	0.00	0.00	0.00	0.00	0.00
**PL11**	0.17	0.24	0.17	0.24	0.17	0.10	0.19	0.14	0.10	0.10	0.09	0.14	0.13	0.29	0.15	0.11	0.16
**GH45**	0.00	0.00	0.01	0.01	0.00	0.00	0.00	0.00	0.00	0.00	0.00	0.00	0.00	0.01	0.00	0.00	0.01
**GT34**	0.00	0.00	0.00	0.00	0.00	0.00	0.00	0.00	0.00	0.00	0.00	0.00	0.00	0.00	0.00	0.00	0.00
**PL14**	0.00	0.00	0.00	0.00	0.00	0.00	0.00	0.00	0.00	0.00	0.00	0.00	0.00	0.00	0.00	0.00	0.00
**GH19**	0.01	0.00	0.01	0.02	0.02	0.01	0.03	0.01	0.00	0.01	0.01	0.00	0.00	0.01	0.00	0.02	0.01
**GH118**	0.00	0.00	0.00	0.00	0.00	0.00	0.00	0.00	0.00	0.00	0.00	0.00	0.00	0.00	0.00	0.00	0.00
**GH82**	0.00	0.00	0.00	0.00	0.00	0.00	0.00	0.00	0.00	0.00	0.00	0.00	0.00	0.00	0.00	0.00	0.00
**GH76**	0.17	0.23	0.30	0.19	0.60	0.22	0.17	0.23	0.15	0.25	0.15	0.23	0.22	0.36	0.24	0.11	0.20
**GT4**	2.28	2.12	2.22	2.44	3.40	1.95	2.77	2.16	2.31	2.09	1.76	2.63	2.21	3.11	2.90	1.61	2.77
**GT87**	0.01	0.00	0.01	0.01	0.00	0.01	0.00	0.01	0.00	0.01	0.01	0.00	0.02	0.00	0.00	0.01	0.00
**GH24**	0.13	0.11	0.14	0.13	0.19	0.12	0.13	0.10	0.12	0.10	0.18	0.10	0.20	0.16	0.12	0.08	0.12
**GH90**	0.00	0.00	0.00	0.00	0.00	0.00	0.00	0.00	0.00	0.00	0.00	0.00	0.00	0.00	0.00	0.00	0.00
**GH57**	0.13	0.26	0.12	0.24	0.23	0.21	0.16	0.15	0.27	0.17	0.11	0.14	0.24	0.18	0.26	0.10	0.22
**GH36**	0.90	0.78	0.72	1.19	1.05	0.83	1.05	0.97	0.77	0.73	0.59	0.88	1.00	1.02	1.09	0.64	0.78
**GT46**	0.00	0.00	0.00	0.00	0.00	0.00	0.00	0.00	0.00	0.00	0.00	0.00	0.00	0.00	0.00	0.00	0.00
**GH66**	0.05	0.05	0.04	0.07	0.04	0.02	0.00	0.03	0.03	0.05	0.04	0.01	0.04	0.03	0.05	0.03	0.03

CAZY_group	AM-AD-34	AM-AD-35	AM-AD-37	AM-AD-38	AM-AD-39	AM-AD-4	AM-AD-40	AM-AD-41	AM-AD-42	AM-AD-43	AM-AD-44	AM-AD-45	AM-AD-46	AM-AD-47	AM-AD-48	AM-AD-49	AM-AD-5
GH48	0.00	0.00	0.00	0.01	0.00	0.00	0.00	0.00	0.00	0.00	0.00	0.00	0.00	0.00	0.00	0.00	0.00
GT53	0.00	0.00	0.00	0.00	0.00	0.00	0.00	0.00	0.00	0.00	0.00	0.00	0.00	0.00	0.00	0.00	0.00
GH60	0.00	0.00	0.00	0.00	0.00	0.00	0.00	0.00	0.00	0.00	0.00	0.00	0.00	0.00	0.00	0.00	0.00
GT31	0.00	0.00	0.00	0.00	0.00	0.00	0.00	0.00	0.00	0.00	0.00	0.00	0.00	0.00	0.00	0.00	0.00
GH105	0.46	0.71	0.53	0.66	0.80	0.45	0.70	0.52	0.53	0.49	0.60	0.62	0.57	0.79	0.67	0.44	0.59
GT23	0.00	0.03	0.00	0.02	0.00	0.01	0.03	0.01	0.03	0.00	0.00	0.01	0.00	0.01	0.01	0.00	0.01
GH11	0.00	0.01	0.00	0.01	0.00	0.00	0.00	0.01	0.03	0.00	0.01	0.00	0.00	0.00	0.01	0.00	0.01
GT38	0.00	0.00	0.00	0.00	0.00	0.00	0.00	0.00	0.00	0.00	0.00	0.00	0.00	0.00	0.00	0.00	0.00
GT43	0.00	0.00	0.00	0.00	0.00	0.00	0.00	0.00	0.00	0.00	0.00	0.00	0.00	0.00	0.00	0.00	0.00
GH99	0.03	0.01	0.02	0.00	0.04	0.00	0.01	0.01	0.00	0.00	0.00	0.00	0.00	0.03	0.02	0.01	0.00
GH38	0.16	0.17	0.25	0.51	0.60	0.25	0.22	0.32	0.47	0.31	0.33	0.34	0.24	0.41	0.45	0.25	0.25
GH109	0.28	0.41	0.25	0.34	0.47	0.35	0.46	0.25	0.36	0.28	0.23	0.28	0.29	0.40	0.34	0.25	0.30
GH2	4.64	5.14	3.85	5.32	8.08	4.09	4.34	4.53	4.41	4.39	3.76	4.40	4.40	5.70	5.30	3.50	4.75
GT28	0.46	0.39	0.44	0.51	0.49	0.43	0.52	0.50	0.40	0.46	0.37	0.44	0.55	0.55	0.54	0.44	0.47
GH42	0.27	0.16	0.24	0.52	0.31	0.34	0.26	0.22	0.27	0.19	0.33	0.25	0.33	0.17	0.32	0.26	0.24
GT58	0.00	0.00	0.00	0.00	0.00	0.00	0.00	0.00	0.00	0.00	0.00	0.00	0.00	0.00	0.00	0.00	0.00
GH61	0.00	0.00	0.00	0.00	0.00	0.00	0.00	0.00	0.00	0.00	0.00	0.00	0.00	0.00	0.00	0.00	0.00
GH80	0.00	0.00	0.00	0.00	0.00	0.00	0.00	0.00	0.00	0.00	0.00	0.00	0.00	0.00	0.00	0.00	0.00
GH98	0.00	0.00	0.02	0.01	0.02	0.01	0.00	0.00	0.00	0.01	0.01	0.00	0.03	0.01	0.02	0.02	0.01
GH6	0.00	0.00	0.00	0.00	0.00	0.00	0.00	0.00	0.00	0.00	0.00	0.00	0.00	0.00	0.00	0.00	0.00
GT1	0.03	0.04	0.07	0.03	0.12	0.05	0.09	0.02	0.03	0.04	0.05	0.05	0.05	0.07	0.06	0.05	0.04
GH28	0.74	1.04	0.83	1.03	1.19	0.83	0.96	0.89	0.65	0.85	0.76	0.88	0.86	1.19	1.04	0.67	0.77
GT42	0.00	0.00	0.00	0.00	0.00	0.00	0.00	0.00	0.00	0.00	0.00	0.00	0.00	0.00	0.00	0.00	0.00
GH128	0.00	0.00	0.00	0.00	0.00	0.00	0.00	0.00	0.00	0.00	0.00	0.00	0.00	0.00	0.01	0.00	0.00
CE12	0.16	0.17	0.05	0.13	0.14	0.13	0.16	0.16	0.13	0.17	0.12	0.27	0.19	0.21	0.08	0.11	0.15
CE16	0.00	0.00	0.00	0.00	0.00	0.00	0.00	0.00	0.00	0.00	0.00	0.00	0.00	0.00	0.00	0.00	0.00
GT89	0.00	0.00	0.00	0.00	0.00	0.00	0.00	0.00	0.00	0.00	0.00	0.00	0.00	0.00	0.00	0.00	0.00
GT11	0.08	0.08	0.05	0.08	0.06	0.04	0.06	0.04	0.12	0.08	0.07	0.05	0.06	0.12	0.07	0.05	0.05
GT77	0.00	0.00	0.00	0.00	0.00	0.01	0.00	0.00	0.00	0.00	0.01	0.00	0.00	0.01	0.00	0.00	0.00
GT55	0.00	0.00	0.00	0.00	0.00	0.00	0.00	0.00	0.00	0.00	0.00	0.00	0.00	0.00	0.00	0.00	0.00
GT18	0.00	0.00	0.00	0.00	0.00	0.00	0.00	0.00	0.00	0.00	0.00	0.00	0.00	0.00	0.00	0.00	0.00
GH87	0.00	0.00	0.01	0.05	0.00	0.00	0.00	0.02	0.00	0.00	0.00	0.00	0.00	0.00	0.02	0.01	0.00
PL5	0.00	0.00	0.00	0.00	0.00	0.00	0.00	0.00	0.00	0.00	0.00	0.00	0.00	0.00	0.00	0.00	0.00
GH22	0.00	0.00	0.00	0.00	0.00	0.00	0.00	0.00	0.00	0.00	0.00	0.00	0.00	0.00	0.00	0.00	0.00
CE2	0.03	0.10	0.08	0.09	0.10	0.05	0.04	0.06	0.18	0.09	0.04	0.09	0.08	0.11	0.10	0.06	0.13
GH120	0.10	0.01	0.09	0.09	0.02	0.04	0.07	0.06	0.06	0.04	0.07	0.08	0.04	0.00	0.07	0.07	0.04
GH73	0.53	0.59	0.52	0.65	0.72	0.63	0.52	0.59	0.77	0.55	0.57	0.66	0.78	0.66	0.52	0.50	0.54
GT17	0.00	0.00	0.00	0.00	0.00	0.01	0.00	0.01	0.00	0.01	0.00	0.00	0.00	0.00	0.00	0.00	0.00
GH124	0.00	0.00	0.00	0.00	0.00	0.00	0.00	0.00	0.00	0.00	0.00	0.00	0.00	0.00	0.00	0.00	0.00
GH54	0.00	0.00	0.00	0.00	0.00	0.00	0.00	0.00	0.00	0.00	0.00	0.00	0.00	0.00	0.00	0.00	0.00
GH100	0.00	0.00	0.00	0.00	0.00	0.00	0.00	0.00	0.00	0.00	0.00	0.00	0.00	0.00	0.00	0.00	0.00
GH113	0.03	0.00	0.03	0.02	0.04	0.04	0.01	0.03	0.01	0.02	0.00	0.05	0.03	0.03	0.06	0.01	0.04
GT5	0.48	0.32	0.48	0.75	0.51	0.42	0.49	0.54	0.38	0.57	0.37	0.40	0.61	0.45	0.64	0.42	0.39
GH94	0.47	0.22	0.56	0.67	0.58	0.41	0.43	0.47	0.37	0.57	0.19	0.37	0.31	0.45	0.56	0.44	0.40
GT66	0.01	0.00	0.00	0.00	0.00	0.00	0.00	0.00	0.00	0.00	0.00	0.00	0.01	0.00	0.00	0.00	0.00
GT94	0.00	0.00	0.00	0.00	0.00	0.00	0.00	0.00	0.00	0.00	0.00	0.00	0.00	0.00	0.00	0.00	0.00
GH58	0.00	0.00	0.00	0.00	0.00	0.00	0.00	0.00	0.00	0.00	0.00	0.00	0.00	0.00	0.00	0.00	0.00
GH1	0.37	0.10	0.48	0.50	0.56	0.35	0.55	0.47	0.30	0.54	0.62	0.19	0.24	0.48	0.67	0.35	0.33
PL22	0.00	0.00	0.00	0.00	0.00	0.00	0.00	0.00	0.00	0.00	0.00	0.00	0.00	0.00	0.00	0.00	0.00

CAZY_group	AM-AD-34	AM-AD-35	AM-AD-37	AM-AD-38	AM-AD-39	AM-AD-4	AM-AD-40	AM-AD-41	AM-AD-42	AM-AD-43	AM-AD-44	AM-AD-45	AM-AD-46	AM-AD-47	AM-AD-48	AM-AD-49	AM-AD-5
GT76	0.00	0.00	0.00	0.00	0.00	0.00	0.00	0.00	0.00	0.00	0.00	0.00	0.00	0.00	0.00	0.00	0.00
GT32	0.10	0.09	0.12	0.10	0.19	0.10	0.13	0.09	0.07	0.08	0.05	0.10	0.16	0.12	0.13	0.10	0.10
GT47	0.08	0.10	0.11	0.09	0.10	0.10	0.09	0.05	0.09	0.06	0.08	0.05	0.09	0.11	0.11	0.07	0.05
GH84	0.16	0.16	0.11	0.08	0.16	0.10	0.17	0.09	0.21	0.13	0.07	0.10	0.12	0.11	0.13	0.08	0.09
GH83	0.00	0.00	0.00	0.00	0.00	0.00	0.00	0.00	0.00	0.00	0.00	0.00	0.00	0.00	0.00	0.00	0.00
GT59	0.00	0.00	0.00	0.00	0.00	0.00	0.00	0.00	0.00	0.00	0.00	0.00	0.00	0.00	0.00	0.00	0.00
GH34	0.00	0.00	0.00	0.00	0.00	0.00	0.00	0.00	0.00	0.00	0.00	0.00	0.00	0.00	0.00	0.00	0.00
GT14	0.02	0.03	0.04	0.02	0.12	0.04	0.01	0.02	0.07	0.01	0.01	0.03	0.00	0.06	0.03	0.03	0.08
GH47	0.00	0.00	0.00	0.00	0.00	0.00	0.00	0.00	0.00	0.00	0.00	0.00	0.00	0.00	0.00	0.00	0.00
GH97	0.98	1.40	0.78	1.26	1.79	0.75	1.12	0.74	0.96	0.85	0.68	1.04	1.08	1.15	1.31	0.57	1.19
GH50	0.08	0.06	0.04	0.03	0.02	0.12	0.00	0.09	0.00	0.07	0.07	0.06	0.10	0.10	0.07	0.06	0.09
GT26	0.23	0.22	0.22	0.27	0.27	0.16	0.24	0.13	0.24	0.21	0.21	0.20	0.23	0.27	0.24	0.18	0.18
GH18	0.63	0.87	0.71	0.87	1.22	0.70	0.56	0.63	0.55	0.73	0.57	0.59	0.75	0.83	0.72	0.51	0.85
GH37	0.00	0.03	0.01	0.03	0.04	0.02	0.00	0.02	0.00	0.00	0.03	0.00	0.00	0.00	0.01	0.00	0.00
PL13	0.04	0.08	0.04	0.07	0.14	0.04	0.06	0.03	0.04	0.03	0.02	0.08	0.02	0.08	0.06	0.02	0.09
GH27	0.27	0.29	0.27	0.41	0.43	0.28	0.46	0.24	0.27	0.33	0.23	0.27	0.19	0.40	0.35	0.18	0.24
GT54	0.00	0.00	0.00	0.00	0.00	0.00	0.00	0.00	0.00	0.00	0.00	0.00	0.00	0.00	0.00	0.00	0.00
GT91	0.00	0.00	0.00	0.00	0.00	0.00	0.00	0.00	0.00	0.00	0.00	0.00	0.00	0.00	0.00	0.00	0.00
GT72	0.00	0.00	0.00	0.00	0.00	0.00	0.00	0.00	0.00	0.00	0.00	0.00	0.00	0.00	0.00	0.00	0.00
GH67	0.08	0.12	0.11	0.06	0.08	0.04	0.07	0.07	0.07	0.07	0.07	0.08	0.07	0.16	0.12	0.07	0.12
GH12	0.00	0.00	0.00	0.00	0.00	0.00	0.00	0.00	0.00	0.01	0.00	0.00	0.00	0.00	0.00	0.00	0.00
GH91	0.03	0.03	0.04	0.07	0.00	0.01	0.01	0.02	0.03	0.04	0.05	0.03	0.01	0.06	0.02	0.01	0.02
GT69	0.00	0.00	0.00	0.00	0.00	0.00	0.00	0.00	0.00	0.00	0.00	0.00	0.00	0.00	0.00	0.00	0.00
GH106	0.21	0.37	0.28	0.37	0.41	0.40	0.24	0.30	0.18	0.24	0.29	0.24	0.52	0.38	0.31	0.18	0.37
GT62	0.00	0.00	0.00	0.00	0.00	0.00	0.00	0.00	0.00	0.00	0.00	0.00	0.00	0.00	0.00	0.00	0.00
GH17	0.00	0.00	0.00	0.00	0.00	0.00	0.01	0.00	0.00	0.00	0.00	0.00	0.00	0.00	0.00	0.00	0.00
GH88	0.37	0.54	0.35	0.51	0.68	0.31	0.52	0.31	0.34	0.28	0.37	0.48	0.35	0.59	0.49	0.21	0.44
CE5	0.00	0.00	0.00	0.00	0.00	0.00	0.00	0.00	0.00	0.00	0.00	0.00	0.00	0.00	0.00	0.00	0.00
GT8	0.12	0.09	0.13	0.08	0.21	0.10	0.07	0.12	0.24	0.13	0.08	0.16	0.07	0.13	0.15	0.05	0.21
GT50	0.00	0.00	0.00	0.00	0.00	0.00	0.00	0.00	0.00	0.00	0.00	0.00	0.00	0.00	0.00	0.00	0.00
GT20	0.10	0.13	0.07	0.13	0.16	0.06	0.01	0.08	0.09	0.10	0.06	0.10	0.11	0.10	0.07	0.06	0.11
PL9	0.05	0.07	0.12	0.10	0.10	0.10	0.01	0.13	0.04	0.06	0.05	0.05	0.05	0.13	0.06	0.07	0.04
GH55	0.01	0.01	0.01	0.06	0.06	0.01	0.09	0.03	0.01	0.01	0.01	0.04	0.03	0.02	0.03	0.02	0.04
GH64	0.00	0.00	0.00	0.00	0.00	0.00	0.01	0.00	0.00	0.00	0.00	0.00	0.00	0.00	0.01	0.00	0.00
GH127	0.30	0.33	0.24	0.37	0.54	0.34	0.37	0.29	0.36	0.30	0.28	0.46	0.37	0.47	0.46	0.24	0.36
GH103	0.01	0.00	0.00	0.00	0.00	0.00	0.00	0.00	0.00	0.00	0.01	0.01	0.00	0.00	0.00	0.01	0.01
GH3	2.90	3.17	2.46	4.01	4.41	2.74	2.93	2.47	3.23	2.68	2.36	3.29	3.14	3.30	3.57	2.55	3.09
GT45	0.00	0.00	0.00	0.00	0.00	0.00	0.00	0.00	0.00	0.00	0.00	0.00	0.00	0.00	0.00	0.00	0.00
GT74	0.00	0.00	0.00	0.00	0.00	0.00	0.00	0.00	0.00	0.00	0.00	0.00	0.00	0.00	0.00	0.00	0.00
GT90	0.00	0.00	0.00	0.01	0.00	0.00	0.00	0.00	0.01	0.01	0.00	0.00	0.00	0.01	0.00	0.00	0.00
GH116	0.08	0.11	0.03	0.08	0.21	0.05	0.04	0.03	0.18	0.04	0.04	0.00	0.05	0.10	0.11	0.08	0.10
GH31	1.35	1.11	1.09	1.50	1.79	0.94	1.25	1.00	0.96	1.01	0.96	0.87	0.97	1.35	1.15	0.80	1.16
GT12	0.00	0.00	0.00	0.00	0.00	0.00	0.00	0.00	0.00	0.00	0.00	0.00	0.00	0.00	0.00	0.00	0.00
CE1	0.40	0.38	0.34	0.43	0.74	0.26	0.35	0.34	0.67	0.41	0.29	0.57	0.52	0.44	0.51	0.34	0.42
GH121	0.00	0.00	0.00	0.02	0.00	0.00	0.09	0.00	0.00	0.00	0.00	0.00	0.01	0.00	0.02	0.05	0.00
GH79	0.00	0.00	0.01	0.00	0.00	0.01	0.00	0.00	0.00	0.00	0.00	0.00	0.00	0.00	0.00	0.00	0.00
GT73	0.00	0.00	0.00	0.00	0.00	0.00	0.00	0.00	0.00	0.00	0.00	0.00	0.00	0.00	0.00	0.00	0.00
GH77	0.65	0.42	0.69	0.82	0.99	0.51	0.91	0.66	0.52	0.58	0.47	0.56	0.60	0.86	0.82	0.52	0.51
PL17	0.00	0.00	0.00	0.00	0.00	0.03	0.06	0.01	0.00	0.01	0.00	0.01	0.03	0.00	0.00	0.00	0.00
PL8	0.30	0.49	0.16	0.35	0.56	0.13	0.36	0.22	0.38	0.27	0.32	0.40	0.18	0.32	0.31	0.19	0.28

CAZY_group	AM-AD-34	AM-AD-35	AM-AD-37	AM-AD-38	AM-AD-39	AM-AD-4	AM-AD-40	AM-AD-41	AM-AD-42	AM-AD-43	AM-AD-44	AM-AD-45	AM-AD-46	AM-AD-47	AM-AD-48	AM-AD-49	AM-AD-5
CE9	0.44	0.36	0.40	0.44	0.64	0.28	0.42	0.41	0.31	0.33	0.40	0.33	0.30	0.53	0.32	0.34	0.31
CE15	0.01	0.03	0.00	0.03	0.06	0.01	0.01	0.02	0.01	0.02	0.03	0.01	0.02	0.07	0.02	0.02	0.04
GT79	0.00	0.00	0.00	0.00	0.00	0.00	0.00	0.00	0.00	0.00	0.00	0.00	0.00	0.00	0.00	0.00	0.00
GT10	0.01	0.00	0.01	0.00	0.04	0.01	0.04	0.01	0.00	0.01	0.00	0.00	0.00	0.01	0.01	0.01	0.02
GH119	0.00	0.00	0.00	0.00	0.00	0.00	0.00	0.00	0.00	0.00	0.00	0.00	0.00	0.00	0.00	0.00	0.00
GH16	0.26	0.27	0.13	0.20	0.39	0.22	0.24	0.22	0.24	0.21	0.18	0.28	0.21	0.21	0.31	0.16	0.18
GH43	2.21	2.76	1.85	2.65	3.73	1.76	2.10	1.93	2.32	2.02	1.75	2.23	2.12	2.45	3.01	1.74	2.52
GH51	0.60	0.74	0.51	0.77	1.07	0.56	0.72	0.52	0.70	0.58	0.59	0.58	0.78	0.78	0.67	0.45	0.65
GT35	0.87	0.78	1.12	1.42	1.13	0.97	1.08	1.04	0.75	1.12	0.86	0.80	1.00	1.12	1.37	1.08	0.81
GT25	0.01	0.00	0.00	0.00	0.02	0.01	0.00	0.01	0.00	0.00	0.00	0.01	0.00	0.00	0.00	0.00	0.02
GT2	4.49	4.26	4.47	6.04	6.76	4.55	5.97	4.65	5.08	4.71	3.80	5.56	5.33	6.07	6.06	3.93	5.61
GT51	1.22	1.06	1.04	1.10	1.53	1.13	1.18	1.04	1.12	0.99	0.82	1.09	1.25	1.17	1.21	0.71	1.21
GH107	0.00	0.00	0.00	0.00	0.00	0.00	0.00	0.00	0.00	0.00	0.00	0.00	0.00	0.00	0.00	0.00	0.00
GH7	0.00	0.00	0.00	0.00	0.00	0.00	0.00	0.00	0.00	0.00	0.00	0.00	0.00	0.00	0.00	0.00	0.00
GT7	0.00	0.00	0.00	0.00	0.00	0.00	0.00	0.00	0.00	0.00	0.00	0.00	0.00	0.00	0.00	0.00	0.00
GT36	0.00	0.00	0.00	0.00	0.00	0.00	0.00	0.00	0.00	0.00	0.00	0.00	0.00	0.00	0.00	0.00	0.00
PL7	0.00	0.00	0.00	0.00	0.00	0.00	0.01	0.00	0.00	0.00	0.00	0.00	0.00	0.00	0.00	0.00	0.00
GH110	0.14	0.14	0.16	0.11	0.21	0.13	0.13	0.10	0.12	0.12	0.13	0.15	0.08	0.16	0.15	0.05	0.13
GT21	0.00	0.00	0.00	0.00	0.00	0.00	0.00	0.00	0.00	0.00	0.00	0.00	0.00	0.00	0.00	0.00	0.00
GH62	0.00	0.00	0.00	0.00	0.00	0.00	0.00	0.00	0.00	0.00	0.00	0.00	0.00	0.00	0.00	0.00	0.00
GH125	0.20	0.29	0.19	0.27	0.39	0.16	0.22	0.18	0.19	0.14	0.23	0.25	0.23	0.25	0.30	0.17	0.21
GH117	0.14	0.16	0.04	0.07	0.19	0.07	0.12	0.09	0.07	0.07	0.06	0.09	0.10	0.11	0.11	0.08	0.10
GH68	0.00	0.00	0.00	0.00	0.00	0.00	0.00	0.00	0.00	0.00	0.00	0.00	0.00	0.00	0.00	0.00	0.00
GH85	0.00	0.00	0.02	0.03	0.00	0.00	0.09	0.02	0.01	0.01	0.00	0.06	0.04	0.00	0.01	0.00	0.00
GH92	1.94	2.66	1.59	2.04	3.48	1.48	2.20	1.71	1.69	1.77	1.53	2.12	1.68	2.47	2.27	1.33	2.21
GH40	0.00	0.00	0.00	0.00	0.00	0.00	0.00	0.00	0.00	0.00	0.00	0.00	0.00	0.00	0.00	0.00	0.00
GT49	0.00	0.00	0.00	0.00	0.00	0.00	0.00	0.00	0.00	0.00	0.00	0.00	0.00	0.00	0.00	0.00	0.00
GH112	0.17	0.03	0.15	0.30	0.19	0.16	0.32	0.15	0.25	0.13	0.23	0.24	0.20	0.22	0.21	0.14	0.14
GT67	0.00	0.00	0.00	0.00	0.00	0.00	0.00	0.00	0.00	0.00	0.00	0.00	0.00	0.00	0.00	0.00	0.00
CE8	0.20	0.19	0.16	0.32	0.21	0.16	0.22	0.18	0.18	0.17	0.18	0.25	0.17	0.23	0.29	0.15	0.26
GT86	0.00	0.00	0.00	0.00	0.00	0.00	0.00	0.00	0.00	0.00	0.00	0.00	0.00	0.00	0.00	0.00	0.00
GH101	0.01	0.00	0.01	0.06	0.00	0.02	0.14	0.01	0.00	0.00	0.02	0.00	0.04	0.00	0.02	0.02	0.00
GH81	0.00	0.00	0.00	0.01	0.00	0.00	0.04	0.01	0.00	0.00	0.01	0.00	0.00	0.00	0.02	0.00	0.00
GT93	0.00	0.00	0.00	0.00	0.00	0.00	0.00	0.00	0.00	0.00	0.00	0.00	0.00	0.00	0.00	0.00	0.00
GT63	0.00	0.00	0.00	0.00	0.00	0.00	0.00	0.00	0.00	0.00	0.00	0.00	0.00	0.00	0.00	0.00	0.00
CE11	0.34	0.24	0.21	0.29	0.41	0.24	0.35	0.19	0.36	0.28	0.24	0.38	0.33	0.30	0.33	0.20	0.30
GT19	0.24	0.24	0.10	0.10	0.21	0.13	0.22	0.14	0.16	0.13	0.12	0.19	0.21	0.20	0.19	0.12	0.16
GT33	0.00	0.00	0.00	0.00	0.00	0.00	0.00	0.00	0.00	0.00	0.00	0.00	0.00	0.00	0.00	0.00	0.00
GH21	0.00	0.00	0.00	0.00	0.00	0.00	0.00	0.00	0.00	0.00	0.00	0.00	0.00	0.00	0.00	0.00	0.00
GT68	0.00	0.00	0.00	0.00	0.00	0.00	0.00	0.00	0.00	0.00	0.00	0.00	0.00	0.00	0.00	0.00	0.00
GH89	0.25	0.39	0.19	0.24	0.21	0.18	0.32	0.27	0.16	0.33	0.15	0.28	0.21	0.41	0.18	0.18	0.28
GT82	0.00	0.00	0.00	0.00	0.00	0.00	0.00	0.00	0.00	0.00	0.00	0.00	0.01	0.00	0.00	0.00	0.00
GH30	0.29	0.33	0.21	0.31	0.33	0.17	0.30	0.23	0.31	0.18	0.27	0.23	0.25	0.27	0.34	0.16	0.24
GH20	1.43	1.45	1.15	1.45	2.66	1.16	1.47	1.26	1.38	1.31	1.21	1.58	1.44	2.09	1.48	0.95	1.64
GH52	0.00	0.00	0.00	0.00	0.00	0.00	0.00	0.00	0.00	0.00	0.00	0.00	0.00	0.00	0.00	0.00	0.00
GT84	0.00	0.04	0.01	0.00	0.00	0.09	0.00	0.01	0.09	0.06	0.01	0.00	0.00	0.10	0.06	0.11	0.01
GT15	0.00	0.00	0.00	0.00	0.00	0.00	0.00	0.00	0.00	0.00	0.00	0.00	0.00	0.00	0.00	0.00	0.00
GH93	0.00	0.00	0.01	0.00	0.06	0.01	0.00	0.01	0.00	0.00	0.01	0.00	0.00	0.01	0.01	0.01	0.00
PL16	0.00	0.00	0.00	0.00	0.00	0.00	0.00	0.00	0.00	0.00	0.00	0.00	0.00	0.00	0.00	0.00	0.00
GH26	0.09	0.10	0.09	0.21	0.27	0.16	0.07	0.20	0.13	0.16	0.12	0.18	0.16	0.13	0.23	0.07	0.27

CAZY_group	AM-AD-34	AM-AD-35	AM-AD-37	AM-AD-38	AM-AD-39	AM-AD-4	AM-AD-40	AM-AD-41	AM-AD-42	AM-AD-43	AM-AD-44	AM-AD-45	AM-AD-46	AM-AD-47	AM-AD-48	AM-AD-49	AM-AD-5
**GT75**	0.00	0.00	0.00	0.00	0.00	0.00	0.00	0.00	0.00	0.00	0.00	0.00	0.00	0.00	0.00	0.00	0.00
**GH53**	0.12	0.14	0.13	0.09	0.43	0.13	0.19	0.15	0.15	0.11	0.01	0.16	0.16	0.12	0.22	0.06	0.20
**GT88**	0.00	0.00	0.00	0.00	0.00	0.00	0.00	0.00	0.00	0.00	0.00	0.00	0.00	0.00	0.00	0.00	0.00
**GT24**	0.00	0.01	0.00	0.00	0.00	0.00	0.00	0.00	0.00	0.00	0.00	0.00	0.00	0.00	0.00	0.00	0.00
**GT81**	0.00	0.00	0.00	0.00	0.00	0.00	0.00	0.00	0.00	0.00	0.00	0.00	0.00	0.00	0.00	0.00	0.00
**GH4**	0.11	0.06	0.14	0.17	0.17	0.11	0.14	0.16	0.06	0.10	0.36	0.03	0.23	0.08	0.17	0.24	0.05
**GT78**	0.00	0.00	0.00	0.00	0.00	0.00	0.00	0.00	0.00	0.00	0.00	0.00	0.00	0.00	0.00	0.00	0.00
**GH8**	0.05	0.03	0.05	0.10	0.12	0.07	0.06	0.04	0.09	0.04	0.04	0.09	0.06	0.07	0.10	0.02	0.07
**GH70**	0.00	0.00	0.00	0.00	0.00	0.00	0.00	0.00	0.00	0.00	0.00	0.00	0.00	0.00	0.00	0.00	0.00
**GH122**	0.00	0.00	0.00	0.00	0.00	0.00	0.00	0.00	0.00	0.00	0.00	0.00	0.00	0.00	0.00	0.00	0.00
**GH74**	0.01	0.03	0.04	0.03	0.00	0.01	0.00	0.07	0.06	0.05	0.01	0.03	0.05	0.06	0.03	0.03	0.03
**GT41**	0.00	0.02	0.02	0.01	0.02	0.01	0.00	0.00	0.00	0.01	0.01	0.03	0.00	0.01	0.01	0.01	0.01
**PL1**	0.24	0.34	0.24	0.38	0.29	0.27	0.20	0.24	0.25	0.19	0.18	0.44	0.29	0.34	0.42	0.25	0.43
**GH15**	0.06	0.10	0.04	0.06	0.08	0.03	0.00	0.05	0.09	0.05	0.01	0.09	0.06	0.07	0.04	0.03	0.07
**GH32**	0.60	0.45	0.46	0.72	0.78	0.43	0.55	0.52	0.34	0.41	0.37	0.44	0.56	0.47	0.67	0.41	0.28
**GH44**	0.00	0.00	0.00	0.01	0.00	0.00	0.00	0.01	0.00	0.00	0.00	0.00	0.00	0.00	0.00	0.00	0.00
**GT6**	0.00	0.01	0.00	0.00	0.02	0.02	0.00	0.00	0.00	0.00	0.00	0.00	0.00	0.01	0.00	0.00	0.00
**GH13**	3.08	2.56	3.38	4.71	4.49	3.38	3.98	3.54	3.23	3.41	2.53	2.86	3.49	3.77	4.41	3.09	3.84
**GT80**	0.01	0.03	0.04	0.06	0.02	0.01	0.04	0.04	0.06	0.03	0.05	0.04	0.02	0.03	0.03	0.01	0.05
**GH71**	0.00	0.00	0.00	0.00	0.00	0.00	0.00	0.00	0.00	0.00	0.00	0.00	0.00	0.00	0.00	0.00	0.00
**GH63**	0.08	0.13	0.08	0.13	0.29	0.13	0.12	0.09	0.18	0.14	0.07	0.10	0.09	0.16	0.10	0.12	0.11
**PL21**	0.02	0.03	0.00	0.00	0.06	0.00	0.00	0.01	0.00	0.02	0.00	0.03	0.00	0.08	0.08	0.04	0.03
**GH65**	0.18	0.24	0.15	0.20	0.29	0.13	0.37	0.16	0.22	0.21	0.19	0.27	0.17	0.12	0.24	0.21	0.16
**CE4**	0.58	0.41	0.72	0.87	0.64	0.57	0.62	0.72	0.65	0.60	0.57	0.58	0.56	0.63	0.80	0.60	0.54
**CE14**	0.04	0.01	0.02	0.01	0.08	0.02	0.01	0.02	0.00	0.02	0.01	0.05	0.03	0.03	0.01	0.01	0.02
**GH115**	0.28	0.41	0.23	0.40	0.39	0.22	0.33	0.23	0.27	0.24	0.27	0.38	0.27	0.34	0.39	0.10	0.38
**GH104**	0.00	0.00	0.00	0.00	0.00	0.00	0.00	0.00	0.00	0.00	0.01	0.00	0.01	0.00	0.00	0.00	0.00
**GH111**	0.00	0.00	0.00	0.00	0.00	0.00	0.00	0.00	0.00	0.00	0.00	0.00	0.00	0.00	0.00	0.01	0.00
**GH95**	0.64	0.80	0.57	0.59	0.85	0.52	0.88	0.57	0.59	0.66	0.54	0.54	0.54	0.82	0.75	0.47	0.68
**GT70**	0.00	0.00	0.00	0.00	0.00	0.00	0.00	0.00	0.00	0.00	0.00	0.00	0.00	0.00	0.00	0.00	0.00
**CE10**	0.19	0.22	0.19	0.24	0.35	0.18	0.23	0.22	0.19	0.19	0.17	0.21	0.23	0.17	0.24	0.19	0.13
**GH10**	0.11	0.10	0.13	0.20	0.17	0.16	0.09	0.08	0.10	0.10	0.14	0.24	0.17	0.13	0.14	0.06	0.16
**GH130**	0.37	0.51	0.44	0.55	0.68	0.28	0.33	0.39	0.31	0.36	0.25	0.37	0.37	0.39	0.44	0.30	0.59
**PL20**	0.00	0.00	0.00	0.00	0.00	0.00	0.00	0.00	0.00	0.00	0.00	0.00	0.00	0.00	0.00	0.00	0.00
**GH69**	0.00	0.00	0.00	0.00	0.00	0.00	0.00	0.00	0.00	0.00	0.00	0.00	0.00	0.00	0.00	0.00	0.00
**PL2**	0.00	0.00	0.00	0.00	0.00	0.00	0.00	0.00	0.00	0.00	0.00	0.00	0.00	0.00	0.00	0.00	0.00
**GH49**	0.00	0.00	0.00	0.00	0.00	0.00	0.00	0.00	0.00	0.00	0.00	0.00	0.00	0.00	0.00	0.00	0.00
**GH25**	0.30	0.39	0.46	0.48	0.43	0.34	0.53	0.52	0.40	0.46	0.37	0.47	0.51	0.47	0.49	0.35	0.39
**GH59**	0.00	0.04	0.04	0.02	0.00	0.01	0.00	0.03	0.00	0.04	0.01	0.00	0.03	0.03	0.03	0.01	0.02
**GH35**	0.57	0.59	0.33	0.45	0.76	0.42	0.43	0.41	0.44	0.39	0.40	0.47	0.39	0.41	0.57	0.38	0.35
**GT39**	0.01	0.00	0.02	0.01	0.00	0.01	0.01	0.01	0.00	0.01	0.01	0.00	0.01	0.00	0.00	0.01	0.01
**GT57**	0.00	0.00	0.00	0.00	0.00	0.00	0.00	0.00	0.00	0.00	0.00	0.00	0.00	0.00	0.00	0.00	0.00
**GH129**	0.03	0.00	0.00	0.03	0.00	0.01	0.00	0.01	0.00	0.00	0.03	0.00	0.00	0.00	0.02	0.01	0.01
**GT22**	0.00	0.01	0.00	0.00	0.00	0.00	0.00	0.00	0.00	0.00	0.00	0.00	0.00	0.00	0.00	0.00	0.00
**PL12**	0.09	0.24	0.12	0.13	0.25	0.16	0.09	0.14	0.22	0.13	0.10	0.18	0.11	0.22	0.17	0.06	0.20
**PL18**	0.00	0.00	0.00	0.00	0.00	0.00	0.00	0.00	0.00	0.00	0.00	0.00	0.00	0.00	0.00	0.00	0.00
**GT92**	0.00	0.00	0.00	0.00	0.00	0.00	0.00	0.00	0.00	0.00	0.00	0.00	0.00	0.00	0.00	0.00	0.00
**GT44**	0.00	0.00	0.00	0.01	0.00	0.00	0.01	0.00	0.00	0.00	0.00	0.00	0.00	0.00	0.00	0.00	0.00
**GT29**	0.00	0.00	0.00	0.00	0.00	0.00	0.00	0.00	0.00	0.00	0.00	0.00	0.00	0.00	0.00	0.00	0.00
**GT13**	0.00	0.00	0.00	0.00	0.00	0.00	0.00	0.00	0.00	0.00	0.00	0.00	0.00	0.00	0.00	0.00	0.00

CAZY_group	AM-AD-34	AM-AD-35	AM-AD-37	AM-AD-38	AM-AD-39	AM-AD-4	AM-AD-40	AM-AD-41	AM-AD-42	AM-AD-43	AM-AD-44	AM-AD-45	AM-AD-46	AM-AD-47	AM-AD-48	AM-AD-49	AM-AD-5
**GT52**	0.00	0.00	0.00	0.00	0.00	0.00	0.00	0.00	0.00	0.00	0.00	0.00	0.00	0.00	0.00	0.00	0.00
**GT64**	0.00	0.00	0.00	0.00	0.00	0.00	0.00	0.00	0.00	0.00	0.00	0.00	0.00	0.00	0.00	0.00	0.00
**GT85**	0.00	0.00	0.00	0.00	0.00	0.00	0.00	0.00	0.00	0.00	0.00	0.00	0.00	0.00	0.00	0.00	0.00
**GT16**	0.00	0.00	0.00	0.00	0.00	0.00	0.00	0.00	0.00	0.00	0.00	0.00	0.00	0.00	0.00	0.00	0.00
**GH56**	0.00	0.00	0.00	0.00	0.00	0.00	0.00	0.00	0.00	0.00	0.00	0.00	0.00	0.00	0.00	0.00	0.00
**GT60**	0.00	0.00	0.00	0.00	0.00	0.00	0.00	0.00	0.00	0.00	0.00	0.00	0.00	0.00	0.00	0.00	0.00
**GH41**	0.00	0.00	0.00	0.00	0.00	0.00	0.00	0.00	0.00	0.00	0.00	0.00	0.00	0.00	0.00	0.00	0.00
**CE7**	0.12	0.13	0.04	0.16	0.21	0.06	0.13	0.10	0.06	0.14	0.12	0.10	0.14	0.18	0.14	0.07	0.11
**GH39**	0.03	0.03	0.04	0.05	0.16	0.06	0.09	0.08	0.12	0.05	0.04	0.08	0.11	0.00	0.09	0.01	0.03
**GT30**	0.45	0.45	0.19	0.32	0.47	0.24	0.32	0.24	0.28	0.33	0.25	0.43	0.40	0.39	0.31	0.21	0.31
**PL15**	0.16	0.11	0.17	0.16	0.21	0.16	0.16	0.09	0.07	0.10	0.11	0.19	0.07	0.23	0.13	0.08	0.23
**GH102**	0.01	0.00	0.02	0.00	0.02	0.01	0.00	0.01	0.01	0.00	0.02	0.01	0.01	0.01	0.01	0.01	0.02

CAZY_group	AM-AD-50	AM-AD-51	AM-AD-52	AM-AD-53	AM-AD-54	AM-AD-55	AM-AD-56	AM-AD-57	AM-AD-58	AM-AD-59	AM-AD-6	AM-AD-60	AM-AD-61	AM-AD-62
**GH29**	0.63	1.02	0.85	0.71	0.76	0.97	0.75	1.28	0.43	0.85	1.50	0.45	1.31	0.77
**PL4**	0.00	0.00	0.00	0.00	0.00	0.00	0.00	0.00	0.00	0.00	0.00	0.00	0.00	0.00
**GH23**	1.09	1.37	1.40	1.24	1.05	1.38	1.32	1.54	1.26	1.45	1.57	1.15	1.69	1.15
**CE6**	0.01	0.04	0.01	0.02	0.02	0.06	0.04	0.05	0.06	0.11	0.14	0.00	0.03	0.03
**GH72**	0.19	0.13	0.16	0.13	0.13	0.15	0.13	0.12	0.11	0.09	0.14	0.14	0.10	0.13
**GH114**	0.00	0.00	0.00	0.01	0.00	0.00	0.00	0.00	0.00	0.00	0.00	0.00	0.00	0.01
**GH78**	0.66	1.23	0.72	0.81	0.93	1.81	0.96	1.69	0.68	1.35	2.09	0.62	1.34	1.09
**PL19**	0.00	0.00	0.00	0.00	0.00	0.00	0.00	0.00	0.00	0.00	0.00	0.00	0.00	0.00
**GT27**	0.00	0.00	0.00	0.00	0.00	0.00	0.00	0.00	0.00	0.00	0.00	0.00	0.00	0.00
**GH5**	0.41	0.45	0.59	0.41	0.53	0.18	0.45	0.42	0.09	0.47	1.12	0.39	0.39	0.59
**GT40**	0.00	0.00	0.00	0.00	0.00	0.00	0.00	0.00	0.00	0.00	0.00	0.00	0.00	0.00
**CE13**	0.01	0.01	0.00	0.00	0.00	0.00	0.01	0.00	0.00	0.00	0.00	0.00	0.00	0.01
**GT37**	0.00	0.00	0.00	0.00	0.00	0.00	0.00	0.00	0.00	0.00	0.00	0.00	0.00	0.00
**GH33**	0.31	0.46	0.20	0.17	0.15	0.36	0.29	0.50	0.26	0.27	0.41	0.16	0.44	0.22
**GT65**	0.00	0.00	0.00	0.00	0.00	0.00	0.00	0.00	0.00	0.00	0.00	0.00	0.00	0.00
**GH86**	0.01	0.01	0.00	0.01	0.00	0.00	0.01	0.00	0.00	0.01	0.00	0.00	0.00	0.00
**GH123**	0.06	0.21	0.11	0.12	0.08	0.17	0.13	0.28	0.09	0.14	0.25	0.10	0.24	0.17
**GH96**	0.00	0.00	0.00	0.00	0.00	0.00	0.00	0.00	0.00	0.00	0.00	0.00	0.00	0.00
**GH14**	0.01	0.00	0.00	0.00	0.00	0.00	0.00	0.00	0.00	0.00	0.00	0.00	0.00	0.00
**CE3**	0.01	0.00	0.00	0.00	0.00	0.00	0.00	0.00	0.00	0.00	0.00	0.00	0.00	0.00
**PL10**	0.06	0.16	0.14	0.13	0.09	0.15	0.11	0.24	0.15	0.16	0.20	0.03	0.13	0.12
**GT48**	0.00	0.00	0.00	0.00	0.00	0.00	0.00	0.00	0.00	0.00	0.00	0.00	0.00	0.00
**PL6**	0.00	0.00	0.00	0.00	0.00	0.03	0.00	0.00	0.00	0.01	0.02	0.00	0.00	0.00
**GT83**	0.04	0.13	0.03	0.08	0.09	0.17	0.11	0.27	0.09	0.05	0.23	0.05	0.29	0.12
**GH126**	0.00	0.00	0.00	0.00	0.00	0.00	0.00	0.00	0.00	0.00	0.00	0.00	0.00	0.00
**GH9**	0.24	0.30	0.28	0.16	0.28	0.08	0.17	0.11	0.09	0.19	0.32	0.20	0.29	0.15
**GH75**	0.00	0.00	0.00	0.00	0.00	0.00	0.00	0.00	0.00	0.00	0.00	0.00	0.00	0.00
**GT71**	0.00	0.00	0.00	0.00	0.00	0.00	0.00	0.00	0.00	0.00	0.00	0.00	0.00	0.00
**GH46**	0.01	0.01	0.00	0.00	0.00	0.00	0.00	0.00	0.00	0.00	0.00	0.00	0.00	0.00
**GT61**	0.00	0.00	0.00	0.00	0.00	0.00	0.00	0.00	0.00	0.00	0.00	0.00	0.00	0.00
**GT3**	0.12	0.33	0.24	0.14	0.25	0.33	0.27	0.50	0.15	0.22	0.23	0.18	0.51	0.27
**PL3**	0.00	0.00	0.00	0.01	0.00	0.00	0.00	0.00	0.00	0.01	0.00	0.00	0.00	0.00
**GH108**	0.02	0.04	0.02	0.04	0.02	0.03	0.03	0.03	0.02	0.07	0.07	0.01	0.06	0.03
**GT56**	0.00	0.00	0.00	0.00	0.00	0.00	0.00	0.00	0.00	0.00	0.00	0.00	0.00	0.00
**PL11**	0.11	0.15	0.25	0.13	0.15	0.29	0.17	0.36	0.11	0.16	0.41	0.07	0.21	0.23
**GH45**	0.00	0.00	0.00	0.01	0.00	0.00	0.00	0.00	0.00	0.00	0.00	0.00	0.00	0.00
**GT34**	0.01	0.00	0.01	0.00	0.00	0.00	0.00	0.00	0.00	0.00	0.00	0.00	0.00	0.00
**PL14**	0.00	0.00	0.00	0.00	0.00	0.00	0.00	0.00	0.00	0.00	0.00	0.00	0.00	0.00
**GH19**	0.00	0.00	0.00	0.01	0.00	0.02	0.04	0.00	0.02	0.01	0.00	0.00	0.01	0.01
**GH118**	0.00	0.00	0.00	0.00	0.00	0.00	0.00	0.00	0.00	0.00	0.00	0.00	0.00	0.00
**GH82**	0.00	0.00	0.00	0.00	0.00	0.00	0.00	0.00	0.00	0.00	0.00	0.00	0.00	0.00
**GH76**	0.06	0.27	0.15	0.20	0.18	0.52	0.16	0.45	0.15	0.32	0.75	0.13	0.38	0.20
**GT4**	1.86	3.11	2.36	1.84	2.07	2.61	2.33	2.47	1.96	1.99	3.19	1.78	3.13	2.16
**GT87**	0.00	0.00	0.01	0.00	0.00	0.00	0.00	0.00	0.02	0.00	0.00	0.00	0.00	0.00
**GH24**	0.09	0.10	0.06	0.08	0.12	0.17	0.14	0.18	0.19	0.12	0.20	0.09	0.06	0.11
**GH90**	0.00	0.00	0.00	0.00	0.00	0.00	0.00	0.00	0.00	0.00	0.00	0.00	0.00	0.00
**GH57**	0.09	0.22	0.18	0.17	0.23	0.18	0.22	0.35	0.07	0.21	0.25	0.16	0.24	0.16
**GH36**	0.94	1.14	1.06	0.62	0.71	1.09	1.02	1.11	0.48	0.69	1.39	0.72	0.99	0.81
**GT46**	0.00	0.00	0.00	0.00	0.00	0.00	0.00	0.00	0.00	0.00	0.00	0.00	0.00	0.00
**GH66**	0.01	0.05	0.03	0.04	0.03	0.08	0.04	0.09	0.00	0.06	0.14	0.01	0.04	0.03

CAZY_group	AM-AD-50	AM-AD-51	AM-AD-52	AM-AD-53	AM-AD-54	AM-AD-55	AM-AD-56	AM-AD-57	AM-AD-58	AM-AD-59	AM-AD-6	AM-AD-60	AM-AD-61	AM-AD-62	
GH48	0.01	0.00	0.02	0.01	0.00	0.00	0.01	0.00	0.00	0.00	0.00	0.01	0.00	0.00	
GT53	0.00	0.00	0.00	0.00	0.00	0.00	0.00	0.00	0.00	0.00	0.00	0.00	0.00	0.00	
GH60	0.00	0.00	0.00	0.00	0.00	0.00	0.00	0.00	0.00	0.00	0.00	0.00	0.00	0.00	
GT31	0.00	0.00	0.00	0.00	0.00	0.00	0.00	0.00	0.00	0.00	0.00	0.00	0.00	0.00	
GH105	0.29	0.75	0.65	0.54	0.56	0.96	0.57	1.01	0.22	0.86	1.55	0.31	0.76	0.71	
GT23	0.02	0.02	0.01	0.01	0.00	0.02	0.00	0.01	0.00	0.00	0.00	0.00	0.01	0.01	
GH11	0.01	0.00	0.04	0.00	0.00	0.00	0.00	0.00	0.00	0.00	0.00	0.00	0.00	0.01	
GT38	0.00	0.00	0.00	0.00	0.00	0.00	0.00	0.00	0.00	0.00	0.00	0.00	0.00	0.00	
GT43	0.00	0.00	0.00	0.00	0.00	0.00	0.00	0.00	0.00	0.00	0.00	0.00	0.00	0.00	
GH99	0.00	0.01	0.01	0.00	0.00	0.03	0.00	0.02	0.04	0.01	0.05	0.00	0.00	0.01	
GH38	0.31	0.33	0.28	0.16	0.25	0.33	0.26	0.32	0.22	0.37	0.27	0.25	0.52	0.27	
GH109	0.15	0.42	0.31	0.21	0.28	0.39	0.29	0.43	0.26	0.30	0.46	0.15	0.58	0.27	
GH2	3.44	5.97	4.55	3.65	4.72	6.68	4.35	7.06	2.98	5.21	8.85	3.02	6.76	4.86	
GT28	0.57	0.61	0.54	0.41	0.36	0.36	0.55	0.39	0.30	0.35	0.43	0.46	0.62	0.45	
GH42	0.48	0.36	0.41	0.19	0.22	0.18	0.25	0.12	0.15	0.23	0.25	0.21	0.20	0.29	
GT58	0.00	0.00	0.00	0.00	0.00	0.00	0.00	0.00	0.00	0.00	0.00	0.00	0.00	0.00	
GH61	0.00	0.00	0.00	0.00	0.00	0.00	0.00	0.00	0.00	0.00	0.00	0.00	0.00	0.00	
GH80	0.00	0.00	0.00	0.00	0.00	0.00	0.00	0.00	0.00	0.00	0.00	0.00	0.00	0.00	
GH98	0.01	0.03	0.02	0.03	0.01	0.02	0.01	0.03	0.00	0.00	0.02	0.00	0.03	0.04	
GH6	0.00	0.00	0.00	0.00	0.00	0.00	0.00	0.00	0.00	0.00	0.00	0.00	0.00	0.00	
GT1	0.05	0.04	0.09	0.05	0.05	0.02	0.04	0.04	0.00	0.01	0.05	0.08	0.08	0.06	
GH28	0.60	1.17	1.17	0.93	0.88	1.50	0.86	1.50	0.61	1.16	1.66	0.53	1.28	1.03	
GT42	0.00	0.00	0.00	0.00	0.00	0.00	0.00	0.00	0.00	0.00	0.00	0.00	0.00	0.00	
GH128	0.00	0.00	0.00	0.00	0.00	0.00	0.00	0.00	0.00	0.01	0.00	0.00	0.00	0.00	
CE12	0.13	0.16	0.22	0.13	0.10	0.23	0.14	0.32	0.13	0.19	0.25	0.10	0.10	0.13	
CE16	0.00	0.00	0.00	0.00	0.00	0.00	0.00	0.00	0.00	0.00	0.00	0.00	0.00	0.00	
GT89	0.00	0.00	0.00	0.00	0.00	0.00	0.00	0.00	0.00	0.00	0.00	0.00	0.00	0.00	
GT11	0.05	0.12	0.06	0.07	0.06	0.06	0.04	0.07	0.07	0.02	0.11	0.04	0.10	0.05	
GT77	0.01	0.00	0.00	0.00	0.00	0.00	0.00	0.01	0.00	0.00	0.00	0.00	0.00	0.00	
GT55	0.00	0.00	0.00	0.00	0.00	0.00	0.00	0.00	0.00	0.00	0.00	0.00	0.00	0.00	
GT18	0.00	0.00	0.00	0.00	0.00	0.00	0.00	0.00	0.00	0.00	0.00	0.00	0.00	0.00	
GH87	0.01	0.01	0.00	0.02	0.00	0.00	0.01	0.00	0.00	0.00	0.00	0.00	0.01	0.01	
PL5	0.00	0.00	0.00	0.00	0.00	0.00	0.00	0.00	0.00	0.00	0.00	0.00	0.00	0.00	
GH22	0.00	0.00	0.00	0.00	0.00	0.00	0.00	0.00	0.00	0.00	0.00	0.00	0.00	0.00	
CE2	0.08	0.10	0.14	0.03	0.09	0.05	0.07	0.10	0.00	0.06	0.09	0.11	0.08	0.07	
GH120	0.09	0.07	0.12	0.04	0.09	0.00	0.04	0.00	0.04	0.00	0.02	0.09	0.00	0.07	
GH73	0.55	0.67	0.62	0.46	0.49	0.58	0.59	0.51	0.46	0.52	0.77	0.54	0.87	0.54	
GT17	0.00	0.00	0.01	0.01	0.01	0.00	0.00	0.00	0.00	0.00	0.00	0.01	0.00	0.00	
GH124	0.00	0.00	0.00	0.00	0.00	0.00	0.00	0.00	0.00	0.00	0.00	0.00	0.00	0.00	
GH54	0.00	0.00	0.00	0.00	0.00	0.00	0.00	0.00	0.00	0.00	0.00	0.00	0.00	0.00	
GH100	0.00	0.00	0.00	0.00	0.00	0.00	0.00	0.00	0.00	0.00	0.00	0.00	0.00	0.00	
GH113	0.05	0.04	0.04	0.02	0.03	0.02	0.03	0.01	0.02	0.02	0.02	0.03	0.01	0.03	
GT5	0.64	0.54	0.51	0.39	0.39	0.27	0.53	0.34	0.22	0.36	0.30	0.61	0.53	0.43	
GH94	0.72	0.52	0.65	0.33	0.45	0.05	0.49	0.09	0.26	0.27	0.30	0.53	0.51	0.47	
GT66	0.00	0.00	0.01	0.00	0.00	0.00	0.00	0.00	0.00	0.00	0.00	0.00	0.00	0.00	
GT94	0.00	0.00	0.00	0.00	0.00	0.00	0.00	0.00	0.00	0.00	0.00	0.00	0.00	0.00	
GH58	0.00	0.00	0.00	0.00	0.00	0.02	0.00	0.00	0.02	0.00	0.00	0.00	0.00	0.00	
GH1	0.61	0.41	0.70	0.45	0.44	0.26	0.49	0.13	0.28	0.27	0.02	0.47	0.52	0.30	
PL22	0.00	0.01	0.00	0.00	0.00	0.00	0.00	0.00	0.00	0.00	0.00	0.00	0.00	0.00	

CAZY_group	AM-AD-50	AM-AD-51	AM-AD-52	AM-AD-53	AM-AD-54	AM-AD-55	AM-AD-56	AM-AD-57	AM-AD-58	AM-AD-59	AM-AD-6	AM-AD-60	AM-AD-61	AM-AD-62
**GT76**	0.00	0.00	0.00	0.00	0.00	0.00	0.00	0.00	0.00	0.00	0.00	0.00	0.00	0.00
**GT32**	0.08	0.13	0.13	0.06	0.07	0.20	0.12	0.10	0.09	0.04	0.09	0.07	0.18	0.11
**GT47**	0.12	0.13	0.09	0.07	0.06	0.03	0.08	0.08	0.00	0.07	0.05	0.05	0.04	0.08
**GH84**	0.09	0.11	0.09	0.13	0.11	0.18	0.16	0.21	0.11	0.05	0.14	0.08	0.15	0.13
**GH83**	0.00	0.00	0.00	0.00	0.00	0.00	0.00	0.00	0.00	0.00	0.00	0.00	0.00	0.00
**GT59**	0.00	0.00	0.00	0.00	0.00	0.00	0.00	0.00	0.00	0.00	0.00	0.00	0.00	0.00
**GH34**	0.00	0.00	0.00	0.00	0.00	0.00	0.00	0.00	0.00	0.00	0.00	0.00	0.00	0.00
**GT14**	0.03	0.04	0.02	0.01	0.03	0.03	0.01	0.08	0.00	0.06	0.07	0.02	0.04	0.03
**GH47**	0.00	0.00	0.00	0.00	0.00	0.00	0.00	0.00	0.00	0.00	0.00	0.00	0.00	0.00
**GH97**	0.29	1.36	0.88	0.67	1.12	1.84	0.81	1.65	0.56	1.41	2.21	0.44	1.43	1.04
**GH50**	0.02	0.11	0.00	0.05	0.05	0.15	0.08	0.12	0.00	0.11	0.32	0.00	0.06	0.07
**GT26**	0.19	0.25	0.18	0.15	0.21	0.18	0.18	0.16	0.24	0.14	0.27	0.15	0.23	0.20
**GH18**	0.56	0.83	0.79	0.54	0.54	0.85	0.64	0.98	0.52	0.85	1.25	0.74	0.81	0.67
**GH37**	0.00	0.02	0.01	0.00	0.01	0.00	0.03	0.02	0.00	0.00	0.00	0.01	0.01	0.04
**PL13**	0.01	0.10	0.03	0.02	0.02	0.09	0.05	0.13	0.02	0.14	0.20	0.02	0.08	0.08
**GH27**	0.26	0.46	0.31	0.25	0.27	0.39	0.28	0.47	0.20	0.33	0.43	0.24	0.56	0.29
**GT54**	0.00	0.00	0.00	0.00	0.00	0.00	0.00	0.00	0.00	0.00	0.00	0.00	0.00	0.00
**GT91**	0.00	0.00	0.00	0.00	0.00	0.00	0.00	0.00	0.00	0.00	0.00	0.00	0.00	0.00
**GT72**	0.00	0.00	0.00	0.00	0.00	0.00	0.00	0.00	0.00	0.00	0.00	0.00	0.00	0.00
**GH67**	0.06	0.11	0.09	0.07	0.09	0.06	0.07	0.11	0.04	0.15	0.16	0.06	0.13	0.08
**GH12**	0.00	0.00	0.00	0.01	0.00	0.00	0.00	0.00	0.00	0.00	0.00	0.00	0.00	0.00
**GH91**	0.02	0.04	0.02	0.03	0.02	0.03	0.07	0.01	0.02	0.01	0.02	0.02	0.06	0.06
**GT69**	0.00	0.00	0.00	0.00	0.00	0.00	0.00	0.00	0.00	0.00	0.00	0.00	0.00	0.00
**GH106**	0.18	0.33	0.39	0.27	0.24	0.73	0.45	0.52	0.09	0.46	0.71	0.10	0.39	0.29
**GT62**	0.00	0.00	0.00	0.00	0.00	0.00	0.00	0.00	0.00	0.00	0.00	0.00	0.00	0.00
**GH17**	0.00	0.00	0.00	0.00	0.00	0.00	0.00	0.00	0.00	0.00	0.00	0.00	0.00	0.00
**GH88**	0.17	0.63	0.36	0.37	0.41	0.70	0.45	0.83	0.20	0.47	0.89	0.22	0.77	0.52
**CE5**	0.00	0.00	0.00	0.00	0.00	0.00	0.00	0.00	0.00	0.00	0.00	0.00	0.00	0.00
**GT8**	0.16	0.21	0.20	0.06	0.10	0.05	0.14	0.10	0.06	0.09	0.16	0.12	0.11	0.13
**GT50**	0.00	0.00	0.00	0.00	0.00	0.00	0.00	0.00	0.00	0.00	0.00	0.00	0.00	0.00
**GT20**	0.05	0.13	0.07	0.08	0.06	0.11	0.08	0.23	0.07	0.05	0.16	0.06	0.19	0.10
**PL9**	0.11	0.10	0.12	0.08	0.07	0.09	0.13	0.12	0.13	0.06	0.11	0.04	0.09	0.14
**GH55**	0.02	0.05	0.02	0.04	0.05	0.02	0.01	0.01	0.02	0.05	0.07	0.07	0.19	0.03
**GH64**	0.00	0.00	0.00	0.01	0.00	0.00	0.00	0.00	0.00	0.00	0.00	0.01	0.01	0.00
**GH127**	0.33	0.41	0.43	0.20	0.40	0.32	0.27	0.61	0.13	0.36	0.55	0.19	0.42	0.37
**GH103**	0.00	0.00	0.01	0.01	0.00	0.02	0.01	0.00	0.00	0.00	0.00	0.00	0.00	0.00
**GH3**	2.61	3.61	2.90	2.90	2.90	3.26	2.76	4.17	2.13	3.68	5.28	2.28	4.32	2.92
**GT45**	0.00	0.00	0.00	0.00	0.00	0.00	0.00	0.00	0.00	0.00	0.00	0.00	0.00	0.00
**GT74**	0.00	0.00	0.00	0.00	0.00	0.00	0.00	0.00	0.00	0.00	0.00	0.00	0.00	0.00
**GT90**	0.00	0.01	0.00	0.00	0.00	0.00	0.00	0.00	0.00	0.00	0.02	0.00	0.00	0.00
**GH116**	0.02	0.04	0.06	0.04	0.09	0.06	0.07	0.09	0.07	0.07	0.11	0.02	0.20	0.06
**GH31**	1.10	1.22	1.23	0.73	1.10	1.24	1.05	1.20	0.81	1.33	1.59	0.81	1.42	1.09
**GT12**	0.00	0.00	0.00	0.00	0.00	0.00	0.00	0.00	0.00	0.00	0.00	0.00	0.00	0.00
**CE1**	0.22	0.47	0.37	0.29	0.41	0.43	0.32	0.50	0.26	0.37	0.82	0.26	0.62	0.38
**GH121**	0.04	0.00	0.05	0.00	0.00	0.00	0.00	0.00	0.00	0.00	0.00	0.00	0.00	0.00
**GH79**	0.00	0.02	0.00	0.00	0.00	0.00	0.00	0.00	0.00	0.00	0.00	0.00	0.00	0.00
**GT73**	0.00	0.00	0.00	0.00	0.00	0.00	0.00	0.00	0.00	0.00	0.00	0.00	0.00	0.00
**GH77**	0.92	0.71	0.71	0.54	0.57	0.49	0.75	0.59	0.52	0.69	0.86	0.67	0.85	0.58
**PL17**	0.00	0.01	0.00	0.00	0.01	0.02	0.00	0.00	0.00	0.00	0.02	0.00	0.00	0.00
**PL8**	0.11	0.38	0.36	0.24	0.22	0.43	0.28	0.60	0.17	0.36	0.55	0.13	0.42	0.35

CAZY_group	AM-AD-50	AM-AD-51	AM-AD-52	AM-AD-53	AM-AD-54	AM-AD-55	AM-AD-56	AM-AD-57	AM-AD-58	AM-AD-59	AM-AD-6	AM-AD-60	AM-AD-61	AM-AD-62
**CE9**	0.42	0.46	0.38	0.30	0.26	0.59	0.38	0.45	0.41	0.38	0.52	0.24	0.48	0.38
**CE15**	0.00	0.01	0.04	0.00	0.01	0.02	0.01	0.04	0.02	0.06	0.00	0.01	0.01	0.01
**GT79**	0.00	0.00	0.00	0.00	0.00	0.00	0.00	0.00	0.00	0.00	0.00	0.00	0.00	0.00
**GT10**	0.01	0.01	0.01	0.01	0.01	0.02	0.02	0.02	0.02	0.00	0.02	0.01	0.00	0.01
**GH119**	0.00	0.00	0.00	0.00	0.00	0.00	0.00	0.00	0.00	0.00	0.00	0.00	0.00	0.00
**GH16**	0.12	0.40	0.18	0.25	0.24	0.27	0.18	0.37	0.09	0.23	0.23	0.21	0.43	0.26
**GH43**	1.71	2.47	2.74	1.57	2.26	3.07	1.64	3.57	1.13	3.32	4.96	1.25	2.79	2.44
**GH51**	0.71	0.65	0.81	0.43	0.68	0.70	0.53	0.79	0.35	0.59	1.12	0.40	0.81	0.68
**GT35**	1.50	1.09	1.39	0.84	0.96	0.64	0.95	0.59	0.80	0.74	0.68	1.28	1.11	0.85
**GT25**	0.00	0.00	0.00	0.00	0.00	0.02	0.01	0.00	0.00	0.00	0.02	0.00	0.00	0.01
**GT2**	4.95	6.00	5.29	3.94	4.44	4.96	4.78	6.04	3.66	4.63	6.46	4.31	6.15	4.99
**GT51**	0.82	1.16	1.13	0.93	0.91	1.09	1.25	1.43	0.74	0.95	1.12	0.81	1.11	1.16
**GH107**	0.00	0.00	0.00	0.00	0.00	0.00	0.00	0.00	0.00	0.00	0.00	0.00	0.00	0.00
**GH7**	0.00	0.00	0.00	0.00	0.00	0.00	0.00	0.00	0.00	0.00	0.00	0.00	0.00	0.00
**GT7**	0.00	0.00	0.00	0.00	0.00	0.00	0.00	0.00	0.00	0.00	0.00	0.00	0.00	0.00
**GT36**	0.00	0.00	0.00	0.00	0.00	0.00	0.00	0.00	0.00	0.00	0.00	0.00	0.00	0.00
**PL7**	0.00	0.00	0.00	0.00	0.00	0.00	0.00	0.00	0.00	0.00	0.00	0.00	0.00	0.00
**GH110**	0.09	0.16	0.14	0.11	0.11	0.26	0.14	0.28	0.07	0.10	0.34	0.08	0.18	0.15
**GT21**	0.00	0.00	0.00	0.00	0.00	0.00	0.00	0.00	0.00	0.00	0.00	0.00	0.00	0.00
**GH62**	0.00	0.00	0.00	0.00	0.00	0.00	0.00	0.00	0.00	0.00	0.00	0.00	0.00	0.00
**GH125**	0.09	0.32	0.22	0.16	0.17	0.41	0.24	0.41	0.09	0.28	0.57	0.10	0.32	0.20
**GH117**	0.05	0.13	0.09	0.12	0.08	0.18	0.11	0.21	0.09	0.14	0.16	0.06	0.19	0.12
**GH68**	0.00	0.00	0.00	0.00	0.00	0.00	0.00	0.00	0.00	0.00	0.00	0.00	0.00	0.00
**GH85**	0.05	0.03	0.03	0.01	0.05	0.00	0.01	0.00	0.00	0.00	0.00	0.00	0.03	0.03
**GH92**	0.80	2.50	1.99	1.28	1.80	3.86	1.74	3.88	1.37	2.55	5.28	0.88	3.07	2.14
**GH40**	0.00	0.00	0.00	0.00	0.00	0.00	0.00	0.00	0.00	0.00	0.00	0.00	0.00	0.00
**GT49**	0.00	0.01	0.00	0.00	0.00	0.00	0.00	0.00	0.00	0.00	0.00	0.00	0.00	0.00
**GH112**	0.24	0.19	0.14	0.18	0.11	0.11	0.17	0.11	0.19	0.20	0.11	0.17	0.11	0.15
**GT67**	0.00	0.00	0.00	0.00	0.00	0.00	0.00	0.00	0.00	0.00	0.00	0.00	0.00	0.00
**CE8**	0.18	0.33	0.30	0.23	0.18	0.27	0.23	0.41	0.19	0.35	0.41	0.09	0.23	0.26
**GT86**	0.00	0.00	0.00	0.00	0.00	0.00	0.00	0.00	0.00	0.00	0.00	0.00	0.00	0.00
**GH101**	0.05	0.00	0.05	0.00	0.00	0.02	0.00	0.00	0.00	0.00	0.00	0.01	0.00	0.00
**GH81**	0.00	0.00	0.01	0.02	0.01	0.00	0.00	0.00	0.00	0.00	0.00	0.02	0.04	0.00
**GT93**	0.00	0.00	0.00	0.00	0.00	0.00	0.00	0.00	0.00	0.00	0.00	0.00	0.00	0.00
**GT63**	0.00	0.00	0.00	0.00	0.00	0.00	0.00	0.00	0.00	0.00	0.00	0.00	0.00	0.00
**CE11**	0.16	0.32	0.25	0.21	0.30	0.35	0.26	0.51	0.26	0.26	0.34	0.20	0.38	0.24
**GT19**	0.10	0.17	0.14	0.13	0.14	0.24	0.21	0.28	0.15	0.21	0.27	0.08	0.25	0.19
**GT33**	0.00	0.00	0.00	0.00	0.00	0.00	0.00	0.00	0.00	0.00	0.00	0.00	0.00	0.00
**GH21**	0.00	0.00	0.00	0.00	0.00	0.00	0.00	0.00	0.00	0.00	0.00	0.00	0.00	0.00
**GT68**	0.00	0.00	0.00	0.00	0.00	0.00	0.00	0.00	0.00	0.00	0.00	0.00	0.00	0.00
**GH89**	0.09	0.31	0.27	0.28	0.27	0.39	0.29	0.42	0.17	0.37	0.61	0.17	0.42	0.31
**GT82**	0.00	0.00	0.00	0.00	0.00	0.00	0.00	0.01	0.00	0.00	0.00	0.00	0.00	0.00
**GH30**	0.19	0.22	0.30	0.23	0.18	0.43	0.22	0.40	0.19	0.35	0.57	0.13	0.33	0.31
**GH20**	0.76	1.98	1.28	1.21	1.14	2.23	1.37	2.67	0.83	1.40	2.98	0.73	2.49	1.40
**GH52**	0.00	0.00	0.00	0.00	0.00	0.00	0.00	0.00	0.00	0.00	0.00	0.00	0.00	0.00
**GT84**	0.12	0.06	0.10	0.03	0.08	0.00	0.09	0.02	0.00	0.02	0.00	0.07	0.01	0.03
**GT15**	0.00	0.00	0.00	0.00	0.00	0.00	0.00	0.00	0.00	0.00	0.00	0.00	0.00	0.00
**GH93**	0.00	0.00	0.00	0.00	0.00	0.02	0.01	0.01	0.00	0.00	0.00	0.00	0.00	0.01
**PL16**	0.00	0.00	0.00	0.00	0.00	0.00	0.00	0.00	0.00	0.00	0.00	0.00	0.00	0.00
**GH26**	0.12	0.16	0.20	0.11	0.20	0.08	0.18	0.11	0.04	0.09	0.25	0.11	0.08	0.20

CAZY_group	AM-AD-50	AM-AD-51	AM-AD-52	AM-AD-53	AM-AD-54	AM-AD-55	AM-AD-56	AM-AD-57	AM-AD-58	AM-AD-59	AM-AD-6	AM-AD-60	AM-AD-61	AM-AD-62
**GT75**	0.00	0.00	0.00	0.00	0.00	0.00	0.00	0.00	0.00	0.00	0.00	0.00	0.00	0.00
**GH53**	0.16	0.17	0.18	0.13	0.15	0.02	0.15	0.07	0.06	0.09	0.11	0.13	0.13	0.20
**GT88**	0.00	0.00	0.00	0.00	0.00	0.00	0.00	0.00	0.00	0.00	0.00	0.00	0.00	0.00
**GT24**	0.00	0.00	0.00	0.00	0.00	0.00	0.00	0.00	0.00	0.00	0.00	0.00	0.00	0.00
**GT81**	0.00	0.00	0.00	0.00	0.00	0.00	0.00	0.00	0.00	0.00	0.00	0.00	0.00	0.00
**GH4**	0.16	0.07	0.19	0.11	0.09	0.06	0.16	0.05	0.09	0.11	0.02	0.13	0.18	0.11
**GT78**	0.00	0.00	0.00	0.00	0.00	0.00	0.00	0.00	0.00	0.00	0.00	0.00	0.00	0.00
**GH8**	0.05	0.07	0.07	0.05	0.07	0.00	0.04	0.04	0.09	0.05	0.05	0.06	0.05	0.06
**GH70**	0.00	0.00	0.00	0.00	0.00	0.00	0.00	0.00	0.00	0.00	0.00	0.00	0.00	0.00
**GH122**	0.00	0.00	0.00	0.00	0.00	0.00	0.00	0.00	0.00	0.00	0.00	0.00	0.00	0.00
**GH74**	0.06	0.04	0.07	0.04	0.04	0.00	0.02	0.00	0.00	0.00	0.07	0.05	0.00	0.02
**GT41**	0.00	0.01	0.01	0.01	0.01	0.02	0.02	0.01	0.00	0.00	0.00	0.00	0.01	0.02
**PL1**	0.21	0.34	0.39	0.33	0.19	0.77	0.29	0.66	0.24	0.25	0.91	0.13	0.34	0.38
**GH15**	0.01	0.07	0.06	0.07	0.03	0.11	0.05	0.11	0.02	0.02	0.09	0.03	0.11	0.07
**GH32**	0.68	0.56	0.55	0.36	0.45	0.73	0.54	0.45	0.52	0.54	0.77	0.45	0.53	0.44
**GH44**	0.01	0.00	0.02	0.00	0.00	0.00	0.00	0.00	0.00	0.00	0.00	0.01	0.01	0.01
**GT6**	0.01	0.00	0.01	0.01	0.00	0.00	0.00	0.00	0.00	0.00	0.02	0.00	0.00	0.00
**GH13**	4.72	3.99	4.08	2.46	2.79	2.14	3.50	2.38	2.42	3.04	3.60	3.50	4.16	3.38
**GT80**	0.00	0.03	0.05	0.01	0.01	0.03	0.03	0.07	0.00	0.02	0.00	0.02	0.03	0.04
**GH71**	0.00	0.00	0.00	0.00	0.00	0.00	0.00	0.00	0.00	0.00	0.00	0.00	0.00	0.00
**GH63**	0.11	0.17	0.16	0.12	0.15	0.14	0.09	0.27	0.04	0.04	0.16	0.05	0.16	0.13
**PL21**	0.01	0.07	0.02	0.01	0.01	0.03	0.03	0.02	0.06	0.04	0.07	0.00	0.04	0.04
**GH65**	0.22	0.16	0.17	0.19	0.16	0.27	0.13	0.27	0.13	0.23	0.16	0.19	0.16	0.11
**CE4**	0.78	0.79	0.78	0.58	0.61	0.50	0.61	0.52	0.28	0.59	0.73	0.63	0.77	0.63
**CE14**	0.01	0.01	0.02	0.01	0.02	0.06	0.01	0.01	0.00	0.00	0.05	0.01	0.04	0.01
**GH115**	0.19	0.38	0.39	0.25	0.31	0.50	0.23	0.61	0.13	0.46	0.77	0.15	0.43	0.35
**GH104**	0.00	0.00	0.00	0.00	0.00	0.02	0.00	0.00	0.00	0.00	0.00	0.00	0.00	0.00
**GH111**	0.00	0.00	0.00	0.00	0.00	0.00	0.00	0.00	0.00	0.00	0.00	0.00	0.00	0.00
**GH95**	0.38	0.82	0.65	0.58	0.74	1.34	0.63	1.12	0.22	0.79	1.64	0.46	0.81	0.82
**GT70**	0.00	0.00	0.00	0.00	0.00	0.00	0.00	0.00	0.00	0.00	0.00	0.00	0.00	0.00
**CE10**	0.12	0.33	0.21	0.17	0.12	0.36	0.22	0.33	0.11	0.16	0.57	0.10	0.30	0.21
**GH10**	0.16	0.16	0.20	0.15	0.13	0.02	0.08	0.12	0.06	0.19	0.16	0.07	0.14	0.14
**GH130**	0.28	0.46	0.39	0.27	0.42	0.56	0.42	0.50	0.19	0.51	0.98	0.28	0.53	0.45
**PL20**	0.00	0.00	0.00	0.00	0.00	0.00	0.00	0.00	0.00	0.00	0.00	0.00	0.00	0.00
**GH69**	0.00	0.00	0.00	0.00	0.00	0.00	0.00	0.00	0.00	0.00	0.00	0.00	0.00	0.00
**PL2**	0.00	0.00	0.00	0.00	0.00	0.00	0.00	0.00	0.00	0.00	0.00	0.00	0.00	0.00
**GH49**	0.00	0.00	0.00	0.00	0.00	0.00	0.00	0.00	0.00	0.00	0.00	0.00	0.00	0.00
**GH25**	0.52	0.52	0.51	0.46	0.38	0.29	0.43	0.28	0.41	0.27	0.32	0.42	0.30	0.41
**GH59**	0.01	0.02	0.03	0.01	0.01	0.00	0.01	0.00	0.00	0.01	0.05	0.02	0.00	0.01
**GH35**	0.28	0.57	0.33	0.38	0.38	0.61	0.39	0.66	0.30	0.48	0.86	0.24	0.42	0.41
**GT39**	0.01	0.00	0.01	0.00	0.00	0.00	0.01	0.02	0.02	0.00	0.00	0.02	0.03	0.01
**GT57**	0.00	0.00	0.00	0.00	0.00	0.00	0.00	0.00	0.00	0.00	0.00	0.00	0.00	0.00
**GH129**	0.04	0.00	0.04	0.00	0.00	0.05	0.00	0.00	0.00	0.00	0.00	0.01	0.00	0.01
**GT22**	0.00	0.00	0.00	0.00	0.00	0.00	0.00	0.00	0.00	0.00	0.00	0.00	0.00	0.00
**PL12**	0.04	0.22	0.14	0.10	0.09	0.15	0.18	0.30	0.19	0.25	0.39	0.06	0.15	0.19
**PL18**	0.00	0.00	0.00	0.00	0.00	0.00	0.00	0.00	0.00	0.00	0.00	0.00	0.00	0.00
**GT92**	0.00	0.00	0.00	0.00	0.00	0.00	0.00	0.00	0.00	0.00	0.00	0.00	0.00	0.00
**GT44**	0.00	0.00	0.00	0.00	0.00	0.00	0.00	0.00	0.00	0.00	0.00	0.00	0.00	0.00
**GT29**	0.00	0.00	0.00	0.00	0.00	0.00	0.00	0.00	0.00	0.00	0.00	0.00	0.00	0.00
**GT13**	0.00	0.00	0.00	0.00	0.00	0.00	0.00	0.00	0.00	0.00	0.00	0.00	0.00	0.00

CAZY_group	AM-AD-50	AM-AD-51	AM-AD-52	AM-AD-53	AM-AD-54	AM-AD-55	AM-AD-56	AM-AD-57	AM-AD-58	AM-AD-59	AM-AD-6	AM-AD-60	AM-AD-61	AM-AD-62
**GT52**	0.00	0.00	0.00	0.00	0.00	0.00	0.00	0.00	0.00	0.00	0.00	0.00	0.00	0.00
**GT64**	0.00	0.00	0.00	0.00	0.00	0.00	0.00	0.00	0.00	0.00	0.00	0.00	0.00	0.00
**GT85**	0.00	0.00	0.00	0.00	0.00	0.00	0.00	0.00	0.00	0.00	0.00	0.00	0.00	0.00
**GT16**	0.00	0.00	0.00	0.00	0.00	0.00	0.00	0.00	0.00	0.00	0.00	0.00	0.00	0.00
**GH56**	0.00	0.00	0.00	0.00	0.00	0.00	0.00	0.00	0.00	0.00	0.00	0.00	0.00	0.00
**GT60**	0.00	0.00	0.00	0.00	0.00	0.00	0.00	0.00	0.00	0.00	0.00	0.00	0.00	0.00
**GH41**	0.00	0.00	0.00	0.00	0.00	0.00	0.00	0.00	0.00	0.00	0.00	0.00	0.00	0.00
**CE7**	0.06	0.17	0.10	0.10	0.08	0.21	0.13	0.15	0.06	0.15	0.23	0.04	0.15	0.11
**GH39**	0.09	0.10	0.05	0.05	0.02	0.00	0.04	0.00	0.02	0.01	0.02	0.03	0.08	0.04
**GT30**	0.14	0.39	0.31	0.19	0.22	0.43	0.32	0.51	0.33	0.30	0.55	0.19	0.43	0.34
**PL15**	0.03	0.14	0.07	0.16	0.07	0.26	0.11	0.22	0.07	0.22	0.50	0.04	0.13	0.13
**GH102**	0.01	0.01	0.00	0.04	0.00	0.00	0.02	0.00	0.00	0.01	0.00	0.00	0.00	0.01

CAZY_group	AM-AD-63	AM-AD-64	AM-AD-65	AM-AD-66	AM-AD-67	AM-AD-68	AM-AD-69	AM-AD-7	AM-AD-70	AM-AD-71	AM-AD-72	AM-AD-73	AM-AD-74
**GH29**	0.81	1.03	1.48	1.19	1.08	0.95	0.85	0.87	0.58	0.94	0.74	0.55	1.18
**PL4**	0.00	0.00	0.00	0.00	0.00	0.00	0.00	0.01	0.00	0.00	0.00	0.00	0.00
**GH23**	1.21	1.53	1.51	1.92	1.69	1.35	1.29	1.31	1.03	1.55	1.18	1.20	1.34
**CE6**	0.04	0.08	0.08	0.07	0.05	0.09	0.03	0.04	0.02	0.03	0.03	0.05	0.07
**GH72**	0.14	0.23	0.18	0.22	0.17	0.16	0.12	0.25	0.16	0.21	0.15	0.13	0.11
**GH114**	0.01	0.00	0.00	0.00	0.00	0.00	0.00	0.00	0.00	0.00	0.00	0.00	0.01
**GH78**	0.80	0.78	1.77	1.77	1.23	1.66	1.14	0.89	1.02	0.78	1.12	1.06	1.32
**PL19**	0.00	0.00	0.00	0.00	0.00	0.00	0.00	0.00	0.00	0.00	0.00	0.00	0.00
**GT27**	0.00	0.00	0.00	0.00	0.00	0.00	0.00	0.00	0.00	0.00	0.00	0.00	0.00
**GH5**	0.40	0.29	0.78	0.86	0.72	0.67	0.71	0.53	0.27	0.31	0.71	0.38	0.22
**GT40**	0.00	0.00	0.00	0.00	0.00	0.00	0.00	0.00	0.00	0.00	0.00	0.00	0.00
**CE13**	0.00	0.00	0.00	0.00	0.00	0.00	0.00	0.00	0.00	0.00	0.00	0.00	0.00
**GT37**	0.00	0.00	0.00	0.00	0.00	0.00	0.00	0.00	0.00	0.00	0.00	0.00	0.00
**GH33**	0.15	0.52	0.45	0.47	0.34	0.38	0.23	0.23	0.13	0.38	0.28	0.16	0.46
**GT65**	0.00	0.00	0.00	0.00	0.00	0.00	0.00	0.00	0.00	0.00	0.00	0.00	0.00
**GH86**	0.00	0.00	0.01	0.00	0.00	0.00	0.00	0.00	0.00	0.00	0.00	0.01	0.01
**GH123**	0.09	0.27	0.37	0.15	0.22	0.15	0.16	0.14	0.06	0.08	0.13	0.15	0.25
**GH96**	0.00	0.00	0.00	0.00	0.00	0.00	0.00	0.00	0.00	0.00	0.00	0.00	0.00
**GH14**	0.00	0.00	0.00	0.00	0.00	0.00	0.00	0.00	0.00	0.00	0.00	0.00	0.00
**CE3**	0.00	0.00	0.00	0.00	0.00	0.00	0.00	0.00	0.02	0.00	0.00	0.00	0.00
**PL10**	0.11	0.08	0.14	0.17	0.19	0.15	0.19	0.12	0.09	0.16	0.14	0.11	0.16
**GT48**	0.00	0.00	0.00	0.00	0.00	0.00	0.00	0.00	0.00	0.00	0.00	0.00	0.00
**PL6**	0.00	0.00	0.00	0.07	0.01	0.00	0.00	0.00	0.01	0.00	0.00	0.00	0.01
**GT83**	0.12	0.17	0.25	0.18	0.14	0.22	0.20	0.10	0.11	0.18	0.08	0.07	0.34
**GH126**	0.00	0.00	0.00	0.00	0.00	0.00	0.00	0.00	0.00	0.00	0.00	0.00	0.00
**GH9**	0.17	0.02	0.16	0.25	0.32	0.19	0.38	0.09	0.08	0.13	0.23	0.47	0.08
**GH75**	0.00	0.00	0.00	0.00	0.00	0.00	0.00	0.00	0.00	0.00	0.00	0.01	0.00
**GT71**	0.00	0.00	0.00	0.00	0.00	0.00	0.00	0.00	0.00	0.00	0.00	0.00	0.00
**GH46**	0.01	0.00	0.00	0.00	0.00	0.00	0.00	0.00	0.00	0.00	0.00	0.00	0.00
**GT61**	0.00	0.00	0.00	0.00	0.00	0.00	0.00	0.00	0.00	0.00	0.00	0.00	0.00
**GT3**	0.21	0.21	0.38	0.22	0.47	0.38	0.24	0.33	0.20	0.13	0.20	0.40	0.48
**PL3**	0.00	0.00	0.00	0.00	0.00	0.00	0.00	0.00	0.00	0.00	0.00	0.00	0.00
**GH108**	0.04	0.00	0.07	0.03	0.04	0.02	0.05	0.01	0.02	0.12	0.02	0.04	0.04
**GT56**	0.00	0.00	0.00	0.00	0.00	0.00	0.00	0.00	0.00	0.00	0.00	0.00	0.00
**PL11**	0.12	0.21	0.24	0.30	0.30	0.33	0.16	0.18	0.17	0.13	0.18	0.11	0.18
**GH45**	0.01	0.00	0.00	0.00	0.00	0.00	0.00	0.00	0.00	0.00	0.00	0.00	0.01
**GT34**	0.00	0.02	0.00	0.00	0.00	0.00	0.00	0.00	0.00	0.00	0.00	0.00	0.00
**PL14**	0.00	0.00	0.00	0.00	0.00	0.00	0.00	0.00	0.00	0.00	0.00	0.00	0.00
**GH19**	0.00	0.04	0.00	0.02	0.02	0.00	0.00	0.01	0.00	0.00	0.00	0.00	0.00
**GH118**	0.00	0.00	0.00	0.00	0.00	0.00	0.00	0.00	0.00	0.00	0.00	0.00	0.00
**GH82**	0.00	0.00	0.00	0.00	0.00	0.00	0.00	0.00	0.00	0.00	0.00	0.00	0.00
**GH76**	0.17	0.33	0.43	0.42	0.39	0.30	0.35	0.16	0.11	0.16	0.17	0.15	0.33
**GT4**	2.04	2.66	3.15	3.75	2.94	2.92	2.62	2.33	1.98	2.49	2.47	1.86	2.41
**GT87**	0.01	0.02	0.00	0.02	0.02	0.01	0.01	0.00	0.00	0.00	0.01	0.00	0.01
**GH24**	0.13	0.23	0.18	0.12	0.12	0.18	0.22	0.15	0.15	0.10	0.12	0.13	0.18
**GH90**	0.00	0.00	0.00	0.00	0.00	0.00	0.00	0.00	0.00	0.00	0.00	0.00	0.00
**GH57**	0.19	0.12	0.28	0.20	0.29	0.23	0.29	0.18	0.12	0.16	0.17	0.18	0.21
**GH36**	0.75	0.99	0.96	1.14	1.04	1.09	0.91	0.71	0.64	0.96	0.92	0.75	0.82
**GT46**	0.00	0.00	0.00	0.00	0.00	0.00	0.00	0.00	0.00	0.00	0.00	0.00	0.00
**GH66**	0.05	0.04	0.04	0.05	0.10	0.07	0.09	0.04	0.03	0.03	0.07	0.04	0.02

CAZY_group	AM-AD-63	AM-AD-64	AM-AD-65	AM-AD-66	AM-AD-67	AM-AD-68	AM-AD-69	AM-AD-7	AM-AD-70	AM-AD-71	AM-AD-72	AM-AD-73	AM-AD-74
GH48	0.00	0.00	0.00	0.00	0.00	0.01	0.00	0.00	0.00	0.00	0.01	0.02	0.00
GT53	0.00	0.00	0.00	0.00	0.00	0.00	0.00	0.00	0.00	0.00	0.00	0.00	0.00
GH60	0.00	0.00	0.00	0.00	0.00	0.00	0.00	0.00	0.00	0.00	0.00	0.00	0.00
GT31	0.00	0.00	0.00	0.00	0.00	0.00	0.00	0.00	0.00	0.00	0.00	0.00	0.00
GH105	0.54	0.79	1.29	1.43	1.03	0.75	0.85	0.49	0.42	0.68	0.60	0.49	0.57
GT23	0.00	0.02	0.01	0.05	0.02	0.02	0.00	0.01	0.02	0.03	0.01	0.00	0.01
GH11	0.01	0.00	0.00	0.00	0.02	0.00	0.00	0.00	0.01	0.03	0.00	0.00	0.00
GT38	0.00	0.00	0.00	0.00	0.00	0.00	0.00	0.00	0.00	0.00	0.00	0.00	0.00
GT43	0.00	0.00	0.00	0.00	0.00	0.00	0.00	0.00	0.00	0.00	0.00	0.00	0.00
GH99	0.02	0.00	0.01	0.07	0.03	0.02	0.00	0.01	0.01	0.02	0.00	0.00	0.00
GH38	0.26	0.37	0.33	0.47	0.42	0.35	0.24	0.19	0.19	0.20	0.31	0.20	0.29
GH109	0.32	0.25	0.57	0.44	0.47	0.35	0.24	0.23	0.21	0.33	0.30	0.29	0.40
GH2	4.12	4.65	7.73	7.73	6.76	6.15	5.77	3.83	3.74	4.70	4.89	3.89	5.13
GT28	0.43	0.48	0.41	0.50	0.52	0.39	0.49	0.48	0.40	0.59	0.45	0.43	0.42
GH42	0.21	0.10	0.17	0.52	0.26	0.26	0.11	0.16	0.17	0.51	0.22	0.11	0.14
GT58	0.00	0.00	0.00	0.00	0.00	0.00	0.00	0.00	0.00	0.00	0.00	0.00	0.00
GH61	0.00	0.00	0.00	0.00	0.00	0.00	0.00	0.00	0.00	0.00	0.00	0.00	0.00
GH80	0.00	0.00	0.00	0.00	0.00	0.00	0.00	0.00	0.00	0.00	0.00	0.00	0.00
GH98	0.01	0.00	0.01	0.02	0.01	0.02	0.01	0.01	0.02	0.00	0.02	0.01	0.01
GH6	0.00	0.00	0.00	0.00	0.00	0.00	0.00	0.00	0.00	0.00	0.00	0.00	0.00
GT1	0.04	0.06	0.06	0.10	0.11	0.05	0.07	0.04	0.05	0.05	0.03	0.03	0.02
GH28	0.73	0.97	1.45	1.87	1.31	1.49	1.00	0.90	0.71	0.94	0.95	0.84	0.98
GT42	0.00	0.00	0.00	0.00	0.00	0.00	0.00	0.00	0.00	0.00	0.00	0.00	0.00
GH128	0.00	0.00	0.00	0.00	0.00	0.00	0.00	0.00	0.00	0.00	0.00	0.00	0.00
CE12	0.10	0.10	0.17	0.29	0.20	0.18	0.18	0.24	0.11	0.20	0.18	0.16	0.20
CE16	0.00	0.00	0.00	0.00	0.00	0.00	0.00	0.00	0.00	0.00	0.00	0.00	0.00
GT89	0.00	0.00	0.00	0.00	0.00	0.00	0.00	0.00	0.00	0.00	0.00	0.00	0.00
GT11	0.07	0.12	0.08	0.10	0.10	0.04	0.06	0.05	0.02	0.07	0.04	0.04	0.09
GT77	0.00	0.00	0.00	0.00	0.00	0.00	0.00	0.00	0.00	0.00	0.00	0.00	0.00
GT55	0.00	0.00	0.00	0.00	0.00	0.00	0.00	0.00	0.00	0.00	0.00	0.00	0.00
GT18	0.00	0.00	0.00	0.00	0.00	0.00	0.00	0.00	0.00	0.00	0.00	0.00	0.00
GH87	0.00	0.00	0.00	0.00	0.00	0.01	0.00	0.01	0.00	0.00	0.00	0.00	0.00
PL5	0.00	0.00	0.00	0.00	0.00	0.00	0.00	0.00	0.00	0.00	0.00	0.00	0.00
GH22	0.01	0.00	0.00	0.00	0.00	0.00	0.00	0.00	0.00	0.00	0.00	0.00	0.00
CE2	0.09	0.04	0.13	0.07	0.14	0.12	0.10	0.05	0.03	0.07	0.06	0.06	0.02
GH120	0.06	0.04	0.01	0.08	0.04	0.02	0.00	0.04	0.00	0.12	0.08	0.01	0.00
GH73	0.57	0.60	0.80	0.87	0.63	0.78	0.53	0.60	0.62	0.66	0.62	0.48	0.53
GT17	0.01	0.00	0.00	0.00	0.00	0.00	0.00	0.00	0.00	0.00	0.01	0.00	0.00
GH124	0.00	0.00	0.00	0.00	0.00	0.00	0.00	0.00	0.00	0.00	0.00	0.00	0.00
GH54	0.00	0.00	0.00	0.00	0.00	0.00	0.00	0.00	0.00	0.00	0.00	0.00	0.00
GH100	0.00	0.00	0.00	0.00	0.00	0.00	0.00	0.00	0.00	0.00	0.00	0.00	0.00
GH113	0.03	0.02	0.02	0.07	0.01	0.03	0.05	0.04	0.01	0.05	0.02	0.02	0.01
GT5	0.41	0.48	0.37	0.50	0.41	0.46	0.43	0.57	0.35	0.78	0.54	0.42	0.29
GH94	0.45	0.19	0.29	0.74	0.35	0.21	0.43	0.44	0.56	0.86	0.38	0.41	0.15
GT66	0.00	0.00	0.00	0.00	0.00	0.00	0.00	0.00	0.00	0.00	0.00	0.00	0.00
GT94	0.00	0.00	0.00	0.00	0.00	0.00	0.00	0.00	0.00	0.00	0.00	0.00	0.00
GH58	0.00	0.00	0.00	0.00	0.00	0.00	0.00	0.00	0.00	0.00	0.00	0.00	0.00
GH1	0.34	0.21	0.36	0.64	0.38	0.36	0.29	0.28	0.57	0.49	0.40	0.49	0.12
PL22	0.00	0.04	0.00	0.00	0.00	0.00	0.00	0.00	0.00	0.00	0.00	0.00	0.00

CAZY_group	AM-AD-63	AM-AD-64	AM-AD-65	AM-AD-66	AM-AD-67	AM-AD-68	AM-AD-69	AM-AD-7	AM-AD-70	AM-AD-71	AM-AD-72	AM-AD-73	AM-AD-74
**GT76**	0.00	0.00	0.00	0.00	0.00	0.00	0.00	0.00	0.00	0.00	0.00	0.00	0.00
**GT32**	0.14	0.08	0.16	0.10	0.10	0.14	0.16	0.12	0.09	0.08	0.09	0.08	0.04
**GT47**	0.09	0.08	0.07	0.12	0.07	0.09	0.05	0.10	0.08	0.05	0.07	0.06	0.09
**GH84**	0.14	0.19	0.12	0.15	0.11	0.14	0.11	0.08	0.08	0.16	0.12	0.04	0.20
**GH83**	0.00	0.00	0.00	0.00	0.00	0.00	0.00	0.00	0.00	0.00	0.00	0.00	0.00
**GT59**	0.00	0.00	0.00	0.00	0.00	0.00	0.00	0.00	0.00	0.00	0.00	0.00	0.00
**GH34**	0.00	0.00	0.00	0.00	0.00	0.00	0.00	0.00	0.00	0.00	0.00	0.00	0.00
**GT14**	0.03	0.02	0.06	0.07	0.04	0.03	0.04	0.03	0.01	0.00	0.04	0.01	0.01
**GH47**	0.00	0.02	0.00	0.00	0.00	0.00	0.00	0.01	0.00	0.00	0.00	0.00	0.00
**GH97**	0.81	0.97	2.14	1.78	1.86	1.72	1.43	0.86	0.61	0.68	1.05	0.94	1.30
**GH50**	0.05	0.14	0.13	0.12	0.13	0.13	0.07	0.08	0.03	0.00	0.07	0.08	0.07
**GT26**	0.16	0.23	0.28	0.45	0.32	0.25	0.24	0.28	0.16	0.25	0.22	0.15	0.22
**GH18**	0.60	0.89	0.77	1.21	0.84	0.83	0.75	0.73	0.42	0.87	0.63	0.53	0.62
**GH37**	0.00	0.00	0.03	0.00	0.00	0.02	0.00	0.03	0.00	0.00	0.00	0.02	0.05
**PL13**	0.05	0.06	0.07	0.12	0.08	0.08	0.05	0.04	0.02	0.05	0.06	0.08	0.07
**GH27**	0.22	0.31	0.57	0.39	0.41	0.25	0.26	0.27	0.21	0.36	0.26	0.19	0.33
**GT54**	0.00	0.00	0.00	0.00	0.00	0.00	0.00	0.00	0.00	0.00	0.00	0.00	0.00
**GT91**	0.00	0.00	0.00	0.00	0.00	0.00	0.00	0.00	0.00	0.00	0.00	0.00	0.00
**GT72**	0.00	0.00	0.00	0.00	0.00	0.00	0.00	0.00	0.00	0.00	0.00	0.00	0.00
**GH67**	0.12	0.06	0.12	0.24	0.13	0.09	0.06	0.09	0.05	0.05	0.10	0.06	0.15
**GH12**	0.00	0.00	0.00	0.00	0.00	0.00	0.00	0.00	0.00	0.00	0.00	0.00	0.00
**GH91**	0.01	0.00	0.07	0.17	0.03	0.04	0.05	0.02	0.02	0.05	0.03	0.02	0.06
**GT69**	0.00	0.00	0.00	0.00	0.00	0.00	0.00	0.00	0.00	0.00	0.00	0.00	0.00
**GH106**	0.21	0.25	0.56	0.76	0.42	0.92	0.29	0.30	0.30	0.23	0.29	0.29	0.46
**GT62**	0.00	0.00	0.00	0.00	0.00	0.00	0.00	0.00	0.00	0.00	0.00	0.00	0.00
**GH17**	0.00	0.00	0.00	0.00	0.00	0.00	0.00	0.01	0.00	0.00	0.00	0.00	0.00
**GH88**	0.36	0.54	0.74	0.81	0.65	0.57	0.33	0.28	0.20	0.31	0.50	0.36	0.56
**CE5**	0.00	0.00	0.00	0.00	0.00	0.00	0.00	0.00	0.00	0.00	0.00	0.00	0.00
**GT8**	0.11	0.14	0.08	0.34	0.14	0.05	0.15	0.20	0.08	0.16	0.05	0.10	0.15
**GT50**	0.00	0.00	0.00	0.00	0.00	0.00	0.00	0.00	0.00	0.00	0.00	0.00	0.00
**GT20**	0.06	0.06	0.16	0.18	0.15	0.09	0.11	0.14	0.08	0.08	0.06	0.12	0.14
**PL9**	0.04	0.04	0.17	0.20	0.06	0.07	0.15	0.18	0.05	0.20	0.10	0.09	0.05
**GH55**	0.03	0.02	0.05	0.05	0.04	0.05	0.03	0.03	0.03	0.07	0.03	0.02	0.01
**GH64**	0.00	0.00	0.00	0.00	0.00	0.00	0.01	0.00	0.00	0.00	0.00	0.01	0.00
**GH127**	0.27	0.37	0.52	0.52	0.47	0.46	0.52	0.41	0.30	0.49	0.35	0.25	0.41
**GH103**	0.00	0.00	0.01	0.02	0.01	0.00	0.00	0.00	0.00	0.00	0.00	0.00	0.00
**GH3**	2.75	2.97	4.40	5.04	4.53	3.57	3.31	2.79	2.09	3.99	3.00	2.39	2.68
**GT45**	0.00	0.00	0.00	0.00	0.00	0.00	0.00	0.00	0.00	0.00	0.00	0.00	0.00
**GT74**	0.00	0.00	0.00	0.00	0.00	0.00	0.00	0.00	0.00	0.00	0.00	0.00	0.00
**GT90**	0.00	0.00	0.01	0.02	0.00	0.00	0.00	0.00	0.00	0.00	0.00	0.00	0.00
**GH116**	0.09	0.00	0.18	0.18	0.13	0.14	0.10	0.06	0.03	0.08	0.06	0.04	0.09
**GH31**	1.12	1.01	1.49	1.87	1.38	1.26	1.17	0.90	0.91	1.25	1.13	0.69	0.95
**GT12**	0.00	0.00	0.00	0.00	0.00	0.00	0.00	0.00	0.00	0.00	0.00	0.00	0.00
**CE1**	0.37	0.33	0.55	0.79	0.54	0.57	0.48	0.41	0.37	0.51	0.45	0.34	0.41
**GH121**	0.00	0.00	0.00	0.00	0.00	0.00	0.00	0.00	0.00	0.00	0.00	0.00	0.00
**GH79**	0.00	0.00	0.00	0.00	0.00	0.00	0.00	0.01	0.01	0.00	0.00	0.00	0.00
**GT73**	0.00	0.00	0.00	0.00	0.00	0.00	0.00	0.00	0.00	0.00	0.00	0.00	0.00
**GH77**	0.57	0.54	0.70	0.87	0.73	0.73	0.53	0.62	0.62	0.74	0.56	0.59	0.40
**PL17**	0.00	0.00	0.02	0.02	0.00	0.00	0.00	0.00	0.00	0.00	0.00	0.00	0.00
**PL8**	0.28	0.21	0.60	0.57	0.56	0.43	0.52	0.15	0.12	0.28	0.25	0.26	0.47

CAZY_group	AM-AD-63	AM-AD-64	AM-AD-65	AM-AD-66	AM-AD-67	AM-AD-68	AM-AD-69	AM-AD-7	AM-AD-70	AM-AD-71	AM-AD-72	AM-AD-73	AM-AD-74
**CE9**	0.34	0.39	0.48	0.71	0.38	0.37	0.30	0.32	0.27	0.40	0.34	0.27	0.28
**CE15**	0.04	0.00	0.03	0.02	0.04	0.05	0.02	0.01	0.00	0.02	0.03	0.05	0.02
**GT79**	0.00	0.00	0.00	0.00	0.00	0.00	0.00	0.00	0.00	0.00	0.00	0.00	0.00
**GT10**	0.00	0.02	0.00	0.00	0.00	0.00	0.01	0.00	0.00	0.00	0.00	0.01	0.02
**GH119**	0.00	0.00	0.00	0.00	0.00	0.00	0.00	0.00	0.00	0.00	0.00	0.00	0.00
**GH16**	0.18	0.39	0.38	0.44	0.32	0.26	0.26	0.23	0.12	0.26	0.21	0.10	0.34
**GH43**	1.98	2.17	3.86	4.76	3.99	3.36	3.23	2.10	1.76	2.36	2.38	1.64	2.24
**GH51**	0.43	0.68	0.92	1.18	0.82	0.72	0.73	0.66	0.47	0.69	0.62	0.47	0.69
**GT35**	0.98	1.14	0.79	1.29	1.02	0.90	0.90	1.27	0.82	1.55	1.07	0.81	0.55
**GT25**	0.00	0.00	0.00	0.03	0.00	0.00	0.00	0.00	0.00	0.00	0.00	0.00	0.02
**GT2**	4.67	4.86	6.48	7.63	6.30	5.55	5.46	5.26	3.96	5.76	4.91	4.07	4.82
**GT51**	0.91	1.22	1.49	1.29	1.38	1.34	1.13	1.26	0.81	1.44	1.08	0.91	1.46
**GH107**	0.00	0.00	0.00	0.00	0.00	0.00	0.00	0.00	0.00	0.00	0.00	0.00	0.00
**GH7**	0.00	0.00	0.00	0.00	0.00	0.00	0.00	0.00	0.00	0.00	0.00	0.00	0.00
**GT7**	0.00	0.00	0.00	0.00	0.00	0.00	0.00	0.00	0.00	0.00	0.00	0.00	0.00
**GT36**	0.00	0.00	0.00	0.00	0.00	0.00	0.00	0.00	0.00	0.00	0.00	0.00	0.00
**PL7**	0.00	0.00	0.00	0.00	0.00	0.00	0.00	0.00	0.00	0.00	0.00	0.00	0.00
**GH110**	0.15	0.16	0.14	0.25	0.15	0.10	0.15	0.09	0.06	0.08	0.13	0.06	0.29
**GT21**	0.00	0.00	0.00	0.00	0.00	0.00	0.00	0.00	0.00	0.00	0.00	0.00	0.00
**GH62**	0.00	0.00	0.00	0.00	0.00	0.01	0.00	0.00	0.00	0.00	0.00	0.00	0.00
**GH125**	0.18	0.23	0.38	0.22	0.34	0.34	0.21	0.17	0.09	0.20	0.23	0.18	0.27
**GH117**	0.08	0.14	0.21	0.17	0.16	0.15	0.10	0.10	0.06	0.12	0.11	0.09	0.16
**GH68**	0.00	0.00	0.00	0.00	0.00	0.00	0.00	0.00	0.00	0.00	0.00	0.00	0.00
**GH85**	0.01	0.00	0.02	0.03	0.01	0.04	0.00	0.09	0.01	0.03	0.05	0.01	0.01
**GH92**	1.81	2.31	3.72	2.96	3.47	3.10	3.05	1.74	1.55	1.45	2.05	1.75	3.42
**GH40**	0.00	0.00	0.00	0.00	0.00	0.00	0.00	0.00	0.00	0.00	0.00	0.00	0.00
**GT49**	0.00	0.00	0.00	0.00	0.00	0.00	0.00	0.00	0.00	0.00	0.00	0.00	0.00
**GH112**	0.16	0.14	0.10	0.42	0.11	0.09	0.15	0.12	0.16	0.35	0.15	0.12	0.05
**GT67**	0.00	0.00	0.00	0.00	0.00	0.00	0.00	0.00	0.00	0.00	0.00	0.00	0.00
**CE8**	0.15	0.25	0.34	0.57	0.32	0.20	0.34	0.24	0.13	0.30	0.22	0.26	0.15
**GT86**	0.00	0.00	0.00	0.00	0.00	0.00	0.00	0.00	0.00	0.00	0.00	0.00	0.00
**GH101**	0.00	0.02	0.00	0.00	0.00	0.02	0.00	0.00	0.02	0.03	0.00	0.01	0.00
**GH81**	0.01	0.00	0.00	0.00	0.01	0.00	0.00	0.05	0.02	0.02	0.00	0.01	0.00
**GT93**	0.00	0.00	0.00	0.00	0.00	0.00	0.00	0.00	0.00	0.00	0.00	0.00	0.00
**GT63**	0.00	0.00	0.00	0.00	0.00	0.00	0.00	0.00	0.00	0.00	0.00	0.00	0.00
**CE11**	0.26	0.25	0.45	0.22	0.39	0.45	0.31	0.28	0.24	0.30	0.28	0.27	0.40
**GT19**	0.15	0.06	0.26	0.17	0.20	0.27	0.21	0.20	0.16	0.15	0.19	0.18	0.28
**GT33**	0.00	0.00	0.00	0.00	0.00	0.00	0.00	0.00	0.00	0.00	0.00	0.00	0.00
**GH21**	0.00	0.00	0.00	0.00	0.00	0.00	0.00	0.00	0.00	0.00	0.00	0.00	0.00
**GT68**	0.00	0.00	0.00	0.00	0.00	0.00	0.00	0.00	0.00	0.00	0.00	0.00	0.00
**GH89**	0.22	0.43	0.45	0.45	0.29	0.31	0.33	0.21	0.20	0.15	0.22	0.25	0.34
**GT82**	0.01	0.00	0.00	0.00	0.00	0.00	0.00	0.00	0.00	0.00	0.01	0.01	0.00
**GH30**	0.19	0.12	0.45	0.29	0.42	0.37	0.32	0.20	0.14	0.26	0.34	0.24	0.26
**GH20**	1.14	1.84	2.80	2.22	2.17	2.06	1.46	1.40	0.91	1.45	1.16	1.17	2.48
**GH52**	0.00	0.00	0.00	0.00	0.00	0.00	0.00	0.00	0.00	0.00	0.00	0.00	0.00
**GT84**	0.12	0.00	0.04	0.02	0.06	0.00	0.08	0.15	0.00	0.00	0.02	0.02	0.00
**GT15**	0.00	0.00	0.00	0.00	0.00	0.00	0.00	0.00	0.00	0.00	0.00	0.00	0.00
**GH93**	0.00	0.00	0.01	0.05	0.01	0.04	0.02	0.00	0.00	0.02	0.01	0.00	0.00
**PL16**	0.00	0.00	0.00	0.00	0.00	0.00	0.00	0.00	0.00	0.00	0.00	0.00	0.00
**GH26**	0.12	0.10	0.19	0.22	0.27	0.21	0.16	0.13	0.02	0.13	0.20	0.09	0.13

CAZY_group	AM-AD-63	AM-AD-64	AM-AD-65	AM-AD-66	AM-AD-67	AM-AD-68	AM-AD-69	AM-AD-7	AM-AD-70	AM-AD-71	AM-AD-72	AM-AD-73	AM-AD-74
GT75	0.00	0.00	0.00	0.00	0.00	0.00	0.00	0.00	0.00	0.00	0.00	0.00	0.00
GH53	0.18	0.08	0.11	0.44	0.11	0.21	0.16	0.17	0.13	0.25	0.11	0.12	0.08
GT88	0.00	0.00	0.00	0.00	0.00	0.00	0.00	0.00	0.00	0.00	0.00	0.00	0.00
GT24	0.00	0.00	0.00	0.00	0.00	0.00	0.00	0.00	0.00	0.00	0.00	0.00	0.00
GT81	0.00	0.00	0.00	0.00	0.00	0.00	0.00	0.00	0.00	0.00	0.00	0.00	0.00
GH4	0.10	0.39	0.09	0.17	0.08	0.11	0.05	0.09	0.11	0.15	0.16	0.15	0.04
GT78	0.00	0.00	0.00	0.00	0.00	0.00	0.00	0.00	0.00	0.00	0.00	0.00	0.00
GH8	0.08	0.04	0.08	0.24	0.12	0.05	0.06	0.07	0.06	0.21	0.09	0.06	0.04
GH70	0.00	0.00	0.00	0.00	0.00	0.00	0.00	0.01	0.00	0.02	0.00	0.00	0.00
GH122	0.00	0.00	0.00	0.00	0.00	0.00	0.00	0.00	0.00	0.00	0.00	0.00	0.00
GH74	0.02	0.04	0.02	0.08	0.02	0.03	0.03	0.01	0.01	0.05	0.05	0.01	0.00
GT41	0.02	0.02	0.01	0.03	0.01	0.01	0.01	0.01	0.00	0.03	0.01	0.00	0.00
PL1	0.20	0.31	0.48	0.72	0.43	0.47	0.48	0.35	0.27	0.26	0.39	0.22	0.30
GH15	0.05	0.02	0.13	0.12	0.06	0.13	0.08	0.07	0.04	0.05	0.05	0.08	0.07
GH32	0.34	0.56	0.58	0.71	0.56	0.55	0.42	0.33	0.37	0.78	0.47	0.33	0.42
GH44	0.00	0.00	0.00	0.00	0.00	0.00	0.00	0.00	0.01	0.00	0.00	0.00	0.00
GT6	0.02	0.00	0.00	0.00	0.00	0.00	0.01	0.00	0.02	0.00	0.00	0.00	0.00
GH13	2.87	2.95	3.08	4.05	3.92	3.38	3.02	3.51	2.76	4.34	3.08	2.49	2.26
GT80	0.04	0.00	0.02	0.00	0.04	0.02	0.01	0.00	0.01	0.03	0.03	0.03	0.05
GH71	0.00	0.00	0.00	0.00	0.00	0.00	0.00	0.00	0.00	0.00	0.00	0.00	0.00
GH63	0.11	0.08	0.23	0.13	0.22	0.14	0.19	0.07	0.09	0.05	0.13	0.13	0.18
PL21	0.07	0.00	0.06	0.05	0.04	0.00	0.08	0.01	0.00	0.10	0.00	0.04	0.00
GH65	0.20	0.06	0.24	0.20	0.20	0.27	0.13	0.23	0.16	0.41	0.20	0.12	0.16
CE4	0.62	0.79	0.57	1.03	0.67	0.62	0.63	0.76	0.53	0.91	0.65	0.53	0.60
CE14	0.03	0.00	0.03	0.02	0.00	0.01	0.03	0.02	0.01	0.02	0.01	0.02	0.00
GH115	0.27	0.43	0.50	0.82	0.49	0.33	0.30	0.28	0.16	0.41	0.36	0.22	0.36
GH104	0.00	0.00	0.00	0.00	0.00	0.00	0.00	0.00	0.00	0.00	0.00	0.00	0.00
GH111	0.00	0.00	0.00	0.00	0.00	0.00	0.00	0.00	0.00	0.00	0.00	0.00	0.00
GH95	0.57	0.85	1.10	1.19	1.17	0.91	0.91	0.57	0.48	0.76	0.72	0.43	0.88
GT70	0.00	0.00	0.00	0.00	0.00	0.00	0.00	0.00	0.00	0.00	0.00	0.00	0.00
CE10	0.17	0.37	0.28	0.34	0.32	0.26	0.23	0.16	0.14	0.15	0.12	0.16	0.27
GH10	0.15	0.14	0.16	0.24	0.25	0.15	0.11	0.17	0.07	0.25	0.17	0.08	0.15
GH130	0.40	0.27	0.69	0.62	0.64	0.50	0.53	0.36	0.28	0.40	0.41	0.33	0.37
PL20	0.00	0.00	0.00	0.00	0.00	0.00	0.00	0.00	0.00	0.00	0.00	0.00	0.00
GH69	0.00	0.00	0.00	0.00	0.00	0.00	0.00	0.00	0.00	0.00	0.00	0.00	0.00
PL2	0.00	0.00	0.00	0.00	0.00	0.00	0.00	0.00	0.00	0.00	0.00	0.00	0.00
GH49	0.00	0.00	0.00	0.00	0.00	0.00	0.00	0.00	0.00	0.00	0.00	0.00	0.00
GH25	0.31	0.62	0.41	0.67	0.53	0.57	0.51	0.52	0.45	0.54	0.49	0.32	0.23
GH59	0.02	0.00	0.01	0.02	0.02	0.00	0.02	0.01	0.02	0.00	0.04	0.00	0.00
GH35	0.42	0.64	0.68	0.72	0.63	0.59	0.46	0.40	0.26	0.43	0.41	0.30	0.49
GT39	0.03	0.00	0.00	0.00	0.02	0.00	0.01	0.01	0.00	0.00	0.01	0.00	0.00
GT57	0.00	0.00	0.00	0.00	0.00	0.00	0.00	0.00	0.00	0.00	0.00	0.00	0.00
GH129	0.00	0.02	0.00	0.00	0.00	0.00	0.00	0.00	0.00	0.03	0.01	0.00	0.00
GT22	0.00	0.00	0.00	0.00	0.00	0.00	0.00	0.00	0.00	0.00	0.00	0.00	0.00
PL12	0.16	0.23	0.21	0.25	0.24	0.15	0.18	0.10	0.09	0.20	0.13	0.13	0.11
PL18	0.00	0.00	0.00	0.00	0.00	0.00	0.00	0.00	0.00	0.00	0.00	0.00	0.00
GT92	0.00	0.00	0.00	0.00	0.00	0.00	0.00	0.00	0.00	0.00	0.00	0.00	0.00
GT44	0.00	0.00	0.00	0.00	0.00	0.00	0.00	0.00	0.00	0.00	0.00	0.00	0.00
GT29	0.00	0.00	0.00	0.00	0.00	0.00	0.00	0.00	0.00	0.00	0.00	0.00	0.00
GT13	0.00	0.00	0.00	0.00	0.00	0.00	0.00	0.00	0.00	0.00	0.00	0.00	0.00

CAZY_group	AM-AD-63	AM-AD-64	AM-AD-65	AM-AD-66	AM-AD-67	AM-AD-68	AM-AD-69	AM-AD-7	AM-AD-70	AM-AD-71	AM-AD-72	AM-AD-73	AM-AD-74
**GT52**	0.00	0.00	0.00	0.00	0.00	0.00	0.00	0.00	0.00	0.00	0.00	0.00	0.00
**GT64**	0.00	0.00	0.00	0.00	0.00	0.00	0.00	0.00	0.00	0.00	0.00	0.00	0.00
**GT85**	0.00	0.00	0.00	0.00	0.00	0.00	0.00	0.00	0.00	0.00	0.00	0.00	0.00
**GT16**	0.00	0.00	0.00	0.00	0.00	0.00	0.00	0.00	0.00	0.00	0.00	0.00	0.00
**GH56**	0.00	0.00	0.00	0.00	0.00	0.00	0.00	0.00	0.00	0.00	0.00	0.00	0.00
**GT60**	0.00	0.00	0.00	0.00	0.00	0.00	0.00	0.00	0.00	0.00	0.00	0.00	0.00
**GH41**	0.00	0.00	0.00	0.00	0.00	0.00	0.00	0.00	0.00	0.00	0.00	0.00	0.00
**CE7**	0.12	0.08	0.17	0.20	0.17	0.27	0.13	0.10	0.11	0.08	0.15	0.11	0.19
**GH39**	0.06	0.02	0.13	0.17	0.08	0.12	0.01	0.06	0.01	0.21	0.04	0.06	0.02
**GT30**	0.26	0.33	0.55	0.32	0.41	0.50	0.36	0.26	0.29	0.38	0.28	0.35	0.54
**PL15**	0.18	0.10	0.24	0.25	0.18	0.19	0.13	0.17	0.16	0.10	0.08	0.09	0.22
**GH102**	0.01	0.00	0.00	0.00	0.00	0.01	0.00	0.01	0.01	0.00	0.02	0.00	0.02

CAZY_group	AM-AD-75	AM-AD-76	AM-AD-77	AM-AD-78	AM-AD-79	AM-AD-8	AM-AD-80	AM-AD-81	AM-AD-82	AM-AD-83	AM-AD-84	AM-AD-85	AM-AD-86
GH29	0.61	0.62	0.63	0.63	1.13	0.92	0.52	0.54	0.34	0.74	0.70	0.56	0.82
PL4	0.00	0.00	0.00	0.00	0.01	0.00	0.00	0.00	0.00	0.00	0.00	0.00	0.00
GH23	1.20	1.25	1.01	1.06	1.08	1.35	1.15	1.32	1.72	1.10	1.20	0.97	1.51
CE6	0.02	0.04	0.02	0.02	0.06	0.09	0.00	0.03	0.00	0.04	0.03	0.11	0.05
GH72	0.16	0.15	0.12	0.15	0.15	0.12	0.14	0.17	0.18	0.11	0.10	0.06	0.13
GH114	0.00	0.00	0.00	0.00	0.00	0.00	0.00	0.00	0.00	0.00	0.00	0.00	0.00
GH78	0.87	0.92	0.93	0.70	1.20	1.11	0.77	0.78	0.29	0.80	0.80	1.06	0.55
PL19	0.00	0.00	0.00	0.00	0.00	0.00	0.00	0.00	0.00	0.00	0.00	0.00	0.00
GT27	0.00	0.00	0.00	0.01	0.00	0.00	0.00	0.00	0.00	0.00	0.00	0.00	0.00
GH5	0.34	0.58	0.57	0.32	0.60	0.18	0.39	0.35	0.26	0.30	0.39	0.32	0.30
GT40	0.00	0.00	0.00	0.00	0.00	0.00	0.00	0.00	0.00	0.00	0.00	0.00	0.00
CE13	0.00	0.00	0.00	0.00	0.00	0.00	0.01	0.00	0.00	0.00	0.00	0.00	0.00
GT37	0.00	0.00	0.00	0.00	0.00	0.00	0.00	0.00	0.00	0.00	0.00	0.00	0.00
GH33	0.17	0.24	0.22	0.19	0.33	0.22	0.14	0.25	0.09	0.19	0.28	0.24	0.33
GT65	0.00	0.00	0.00	0.00	0.00	0.00	0.00	0.00	0.00	0.00	0.00	0.00	0.00
GH86	0.00	0.00	0.02	0.00	0.03	0.00	0.00	0.01	0.00	0.00	0.00	0.00	0.00
GH123	0.09	0.09	0.13	0.12	0.14	0.15	0.12	0.08	0.02	0.13	0.14	0.09	0.13
GH96	0.00	0.00	0.00	0.00	0.00	0.00	0.00	0.00	0.00	0.00	0.00	0.00	0.00
GH14	0.00	0.00	0.00	0.00	0.00	0.00	0.00	0.00	0.00	0.00	0.00	0.00	0.00
CE3	0.00	0.01	0.00	0.00	0.00	0.00	0.00	0.00	0.00	0.00	0.00	0.00	0.00
PL10	0.09	0.07	0.10	0.11	0.11	0.34	0.04	0.03	0.01	0.09	0.08	0.13	0.13
GT48	0.00	0.00	0.00	0.00	0.00	0.00	0.00	0.00	0.00	0.00	0.00	0.00	0.00
PL6	0.00	0.00	0.01	0.00	0.00	0.00	0.00	0.00	0.00	0.00	0.00	0.00	0.00
GT83	0.08	0.07	0.05	0.12	0.08	0.22	0.08	0.09	0.03	0.09	0.17	0.06	0.13
GH126	0.00	0.00	0.00	0.00	0.00	0.00	0.00	0.00	0.00	0.00	0.00	0.00	0.00
GH9	0.10	0.35	0.14	0.14	0.23	0.03	0.09	0.19	0.10	0.11	0.08	0.02	0.13
GH75	0.00	0.00	0.00	0.00	0.00	0.00	0.00	0.00	0.00	0.00	0.00	0.00	0.00
GT71	0.00	0.00	0.00	0.00	0.00	0.00	0.00	0.00	0.00	0.00	0.00	0.00	0.00
GH46	0.00	0.00	0.00	0.00	0.00	0.00	0.01	0.00	0.00	0.00	0.00	0.00	0.00
GT61	0.01	0.00	0.00	0.00	0.00	0.00	0.00	0.00	0.00	0.00	0.00	0.00	0.00
GT3	0.15	0.08	0.28	0.14	0.17	0.03	0.22	0.11	0.11	0.17	0.26	0.06	0.22
PL3	0.00	0.00	0.00	0.00	0.00	0.00	0.00	0.00	0.00	0.00	0.00	0.00	0.00
GH108	0.02	0.03	0.03	0.04	0.03	0.00	0.03	0.02	0.01	0.06	0.03	0.04	0.03
GT56	0.01	0.01	0.00	0.00	0.00	0.09	0.00	0.00	0.02	0.00	0.00	0.00	0.00
PL11	0.14	0.20	0.23	0.11	0.27	0.31	0.09	0.12	0.02	0.08	0.19	0.24	0.18
GH45	0.00	0.01	0.00	0.01	0.00	0.00	0.01	0.00	0.00	0.00	0.00	0.00	0.00
GT34	0.01	0.01	0.00	0.01	0.00	0.00	0.00	0.00	0.00	0.00	0.00	0.00	0.00
PL14	0.00	0.00	0.00	0.00	0.00	0.00	0.00	0.00	0.00	0.00	0.00	0.00	0.00
GH19	0.02	0.01	0.01	0.01	0.02	0.09	0.01	0.02	0.01	0.01	0.00	0.00	0.00
GH118	0.00	0.00	0.00	0.00	0.00	0.00	0.00	0.00	0.00	0.00	0.00	0.00	0.00
GH82	0.00	0.00	0.00	0.00	0.00	0.00	0.00	0.00	0.00	0.00	0.00	0.00	0.00
GH76	0.13	0.15	0.21	0.19	0.42	0.25	0.09	0.21	0.09	0.15	0.20	0.37	0.08
GT4	2.15	1.89	1.96	1.96	2.22	1.78	1.71	1.93	1.48	1.91	1.86	1.94	2.30
GT87	0.00	0.00	0.00	0.01	0.01	0.00	0.01	0.00	0.00	0.01	0.01	0.00	0.02
GH24	0.14	0.22	0.10	0.09	0.22	0.28	0.10	0.12	0.08	0.11	0.16	0.13	0.17
GH90	0.01	0.00	0.00	0.00	0.00	0.00	0.00	0.00	0.00	0.00	0.00	0.00	0.00
GH57	0.07	0.07	0.15	0.15	0.15	0.15	0.11	0.08	0.06	0.11	0.18	0.09	0.15
GH36	0.97	0.94	1.00	0.71	1.01	0.95	0.70	0.66	0.57	0.69	0.76	0.80	0.88
GT46	0.00	0.00	0.00	0.00	0.00	0.00	0.00	0.00	0.00	0.00	0.00	0.00	0.00
GH66	0.04	0.03	0.02	0.02	0.09	0.06	0.03	0.07	0.00	0.03	0.02	0.09	0.05

CAZY_group	AM-AD-75	AM-AD-76	AM-AD-77	AM-AD-78	AM-AD-79	AM-AD-8	AM-AD-80	AM-AD-81	AM-AD-82	AM-AD-83	AM-AD-84	AM-AD-85	AM-AD-86
GH48	0.00	0.01	0.00	0.00	0.00	0.00	0.00	0.00	0.00	0.00	0.00	0.00	0.00
GT53	0.00	0.00	0.00	0.00	0.00	0.00	0.00	0.00	0.00	0.00	0.00	0.00	0.00
GH60	0.00	0.00	0.00	0.00	0.00	0.00	0.00	0.00	0.00	0.00	0.00	0.00	0.00
GT31	0.00	0.00	0.00	0.00	0.00	0.00	0.00	0.00	0.00	0.00	0.00	0.00	0.00
GH105	0.48	0.51	0.63	0.53	1.03	0.92	0.20	0.51	0.19	0.46	0.50	0.82	0.72
GT23	0.01	0.01	0.01	0.00	0.01	0.00	0.01	0.00	0.01	0.02	0.01	0.00	0.00
GH11	0.00	0.00	0.01	0.01	0.00	0.00	0.01	0.00	0.00	0.00	0.00	0.00	0.00
GT38	0.00	0.01	0.00	0.00	0.00	0.00	0.00	0.00	0.00	0.00	0.00	0.00	0.00
GT43	0.00	0.00	0.00	0.00	0.00	0.00	0.00	0.00	0.00	0.00	0.00	0.00	0.00
GH99	0.00	0.02	0.01	0.01	0.02	0.00	0.01	0.04	0.00	0.00	0.00	0.00	0.02
GH38	0.33	0.23	0.26	0.16	0.29	0.15	0.26	0.26	0.16	0.17	0.23	0.39	0.33
GH109	0.23	0.20	0.26	0.28	0.26	0.25	0.14	0.17	0.02	0.26	0.33	0.22	0.28
GH2	4.07	4.38	4.25	3.50	6.68	5.10	2.98	4.45	2.51	3.80	3.97	4.87	5.19
GT28	0.52	0.57	0.45	0.41	0.39	0.31	0.44	0.48	0.54	0.45	0.35	0.43	0.45
GH42	0.49	0.36	0.22	0.17	0.24	0.12	0.30	0.21	0.41	0.22	0.16	0.32	0.13
GT58	0.00	0.00	0.00	0.00	0.00	0.00	0.00	0.00	0.00	0.00	0.00	0.00	0.00
GH61	0.00	0.00	0.00	0.00	0.00	0.00	0.00	0.00	0.00	0.00	0.00	0.00	0.00
GH80	0.00	0.00	0.00	0.00	0.00	0.00	0.00	0.00	0.00	0.00	0.00	0.00	0.00
GH98	0.01	0.00	0.00	0.01	0.02	0.00	0.00	0.00	0.01	0.00	0.00	0.00	0.03
GH6	0.00	0.00	0.00	0.00	0.00	0.00	0.00	0.00	0.00	0.00	0.00	0.00	0.00
GT1	0.05	0.04	0.07	0.05	0.06	0.00	0.03	0.01	0.07	0.05	0.05	0.04	0.03
GH28	0.97	0.88	0.94	0.73	1.36	1.32	0.48	0.85	0.52	0.85	0.84	1.16	0.97
GT42	0.00	0.00	0.00	0.00	0.00	0.00	0.00	0.00	0.00	0.00	0.00	0.00	0.00
GH128	0.00	0.00	0.00	0.00	0.01	0.00	0.00	0.00	0.00	0.00	0.00	0.00	0.02
CE12	0.09	0.12	0.17	0.12	0.32	0.18	0.08	0.09	0.05	0.10	0.11	0.17	0.17
CE16	0.00	0.00	0.00	0.00	0.00	0.00	0.00	0.00	0.00	0.00	0.00	0.00	0.00
GT89	0.00	0.00	0.00	0.00	0.00	0.00	0.00	0.00	0.00	0.00	0.00	0.00	0.00
GT11	0.06	0.07	0.05	0.08	0.05	0.06	0.04	0.03	0.03	0.08	0.06	0.06	0.05
GT77	0.01	0.01	0.00	0.00	0.00	0.03	0.00	0.01	0.01	0.00	0.00	0.00	0.02
GT55	0.00	0.00	0.00	0.00	0.00	0.00	0.00	0.00	0.00	0.00	0.00	0.00	0.00
GT18	0.00	0.00	0.00	0.00	0.00	0.00	0.00	0.00	0.00	0.00	0.00	0.00	0.00
GH87	0.01	0.00	0.01	0.00	0.00	0.03	0.01	0.02	0.02	0.00	0.00	0.00	0.00
PL5	0.00	0.00	0.00	0.00	0.00	0.00	0.00	0.00	0.00	0.00	0.00	0.00	0.00
GH22	0.00	0.00	0.00	0.00	0.00	0.00	0.01	0.00	0.00	0.00	0.00	0.00	0.00
CE2	0.06	0.12	0.05	0.04	0.06	0.03	0.11	0.09	0.06	0.05	0.06	0.00	0.05
GH120	0.11	0.10	0.01	0.07	0.06	0.00	0.07	0.03	0.06	0.08	0.05	0.00	0.02
GH73	0.68	0.73	0.46	0.45	0.44	0.52	0.51	0.62	0.67	0.53	0.50	0.58	0.65
GT17	0.01	0.01	0.00	0.00	0.00	0.00	0.01	0.00	0.00	0.00	0.00	0.00	0.00
GH124	0.00	0.00	0.00	0.00	0.00	0.00	0.00	0.00	0.00	0.00	0.00	0.00	0.00
GH54	0.00	0.00	0.00	0.00	0.00	0.00	0.00	0.00	0.00	0.00	0.00	0.00	0.00
GH100	0.00	0.00	0.00	0.00	0.00	0.00	0.00	0.00	0.00	0.00	0.00	0.00	0.00
GH113	0.02	0.05	0.03	0.02	0.03	0.00	0.04	0.04	0.05	0.04	0.04	0.02	0.02
GT5	0.57	0.55	0.50	0.38	0.38	0.34	0.43	0.53	0.58	0.37	0.40	0.41	0.47
GH94	0.36	0.71	0.56	0.34	0.46	0.12	0.48	0.39	0.53	0.39	0.23	0.28	0.38
GT66	0.00	0.00	0.00	0.01	0.00	0.00	0.00	0.00	0.00	0.00	0.00	0.00	0.00
GT94	0.00	0.00	0.00	0.00	0.00	0.00	0.00	0.00	0.00	0.00	0.00	0.00	0.00
GH58	0.00	0.01	0.00	0.00	0.00	0.00	0.00	0.00	0.00	0.00	0.00	0.00	0.00
GH1	0.99	0.71	0.42	0.47	0.49	0.43	0.35	0.40	0.53	0.53	0.41	1.08	0.92
PL22	0.01	0.00	0.00	0.00	0.00	0.00	0.00	0.00	0.00	0.00	0.00	0.00	0.00

CAZY_group	AM-AD-75	AM-AD-76	AM-AD-77	AM-AD-78	AM-AD-79	AM-AD-8	AM-AD-80	AM-AD-81	AM-AD-82	AM-AD-83	AM-AD-84	AM-AD-85	AM-AD-86
GT76	0.00	0.00	0.01	0.00	0.00	0.00	0.00	0.00	0.00	0.00	0.00	0.00	0.00
GT32	0.05	0.10	0.07	0.09	0.08	0.03	0.07	0.05	0.07	0.03	0.03	0.06	0.13
GT47	0.08	0.08	0.07	0.07	0.08	0.06	0.10	0.07	0.15	0.09	0.06	0.04	0.10
GH84	0.13	0.07	0.10	0.14	0.14	0.18	0.06	0.15	0.00	0.12	0.07	0.19	0.15
GH83	0.00	0.00	0.00	0.00	0.00	0.00	0.00	0.00	0.00	0.00	0.00	0.00	0.00
GT59	0.00	0.00	0.00	0.00	0.00	0.00	0.00	0.00	0.00	0.00	0.00	0.00	0.00
GH34	0.00	0.00	0.00	0.00	0.00	0.00	0.00	0.00	0.00	0.00	0.00	0.00	0.00
GT14	0.02	0.03	0.02	0.03	0.09	0.00	0.03	0.03	0.00	0.01	0.03	0.02	0.02
GH47	0.00	0.00	0.00	0.02	0.00	0.00	0.00	0.00	0.00	0.00	0.01	0.00	0.00
GH97	0.70	0.49	0.87	0.68	1.42	0.98	0.39	0.80	0.32	0.71	0.78	0.97	1.07
GH50	0.06	0.04	0.07	0.03	0.09	0.09	0.02	0.02	0.00	0.07	0.09	0.19	0.00
GT26	0.22	0.29	0.21	0.17	0.18	0.12	0.17	0.27	0.15	0.20	0.16	0.11	0.15
GH18	0.61	0.61	0.59	0.51	0.91	0.83	0.57	0.71	0.54	0.45	0.47	0.95	0.77
GH37	0.02	0.02	0.03	0.02	0.00	0.09	0.01	0.00	0.00	0.01	0.00	0.00	0.00
PL13	0.03	0.02	0.05	0.04	0.05	0.06	0.02	0.03	0.01	0.01	0.03	0.09	0.00
GH27	0.21	0.23	0.26	0.23	0.28	0.18	0.24	0.25	0.12	0.26	0.22	0.26	0.22
GT54	0.00	0.00	0.00	0.00	0.00	0.00	0.00	0.00	0.00	0.00	0.00	0.00	0.00
GT91	0.00	0.00	0.00	0.00	0.00	0.00	0.00	0.00	0.00	0.00	0.00	0.00	0.00
GT72	0.00	0.00	0.00	0.00	0.00	0.00	0.00	0.00	0.00	0.00	0.00	0.00	0.00
GH67	0.07	0.10	0.05	0.08	0.09	0.18	0.04	0.03	0.01	0.09	0.10	0.06	0.05
GH12	0.00	0.00	0.00	0.00	0.00	0.00	0.00	0.00	0.00	0.00	0.00	0.00	0.00
GH91	0.03	0.01	0.01	0.02	0.06	0.03	0.01	0.03	0.00	0.08	0.03	0.04	0.05
GT69	0.00	0.00	0.00	0.00	0.00	0.00	0.00	0.00	0.00	0.00	0.00	0.00	0.00
GH106	0.18	0.27	0.26	0.18	0.36	0.34	0.16	0.24	0.03	0.20	0.30	0.43	0.12
GT62	0.00	0.00	0.00	0.00	0.00	0.00	0.00	0.00	0.00	0.00	0.00	0.00	0.00
GH17	0.01	0.00	0.00	0.02	0.00	0.00	0.01	0.00	0.00	0.00	0.00	0.00	0.00
GH88	0.33	0.39	0.36	0.38	0.56	0.37	0.20	0.21	0.10	0.30	0.39	0.50	0.53
CE5	0.00	0.00	0.00	0.00	0.00	0.00	0.00	0.00	0.00	0.00	0.00	0.00	0.00
GT8	0.09	0.12	0.09	0.13	0.07	0.09	0.12	0.11	0.14	0.09	0.10	0.22	0.28
GT50	0.00	0.00	0.00	0.00	0.00	0.00	0.00	0.00	0.00	0.00	0.00	0.00	0.00
GT20	0.08	0.07	0.06	0.11	0.13	0.09	0.05	0.03	0.03	0.10	0.10	0.04	0.10
PL9	0.05	0.06	0.15	0.10	0.20	0.03	0.05	0.09	0.07	0.01	0.10	0.15	0.08
GH55	0.01	0.01	0.05	0.01	0.06	0.00	0.01	0.10	0.02	0.01	0.01	0.00	0.05
GH64	0.01	0.00	0.01	0.00	0.00	0.00	0.01	0.01	0.00	0.00	0.00	0.00	0.02
GH127	0.34	0.34	0.31	0.27	0.42	0.52	0.28	0.23	0.20	0.30	0.37	0.15	0.27
GH103	0.01	0.00	0.00	0.00	0.00	0.06	0.01	0.00	0.01	0.00	0.00	0.00	0.02
GH3	3.01	3.43	2.65	2.55	4.36	2.58	2.24	2.63	2.00	2.42	2.57	2.91	2.95
GT45	0.00	0.00	0.00	0.00	0.00	0.00	0.00	0.00	0.00	0.00	0.00	0.00	0.00
GT74	0.00	0.00	0.00	0.00	0.00	0.00	0.00	0.00	0.00	0.00	0.00	0.00	0.00
GT90	0.00	0.01	0.01	0.01	0.00	0.00	0.00	0.00	0.00	0.00	0.00	0.00	0.00
GH116	0.04	0.01	0.03	0.07	0.11	0.00	0.05	0.06	0.02	0.08	0.03	0.04	0.05
GH31	1.14	1.20	0.84	1.04	1.60	0.95	0.81	1.16	1.01	0.98	0.84	1.27	1.10
GT12	0.00	0.00	0.00	0.00	0.00	0.00	0.00	0.00	0.00	0.00	0.00	0.00	0.00
CE1	0.29	0.29	0.36	0.37	0.59	0.22	0.31	0.31	0.27	0.29	0.36	0.37	0.72
GH121	0.04	0.01	0.00	0.03	0.00	0.06	0.05	0.00	0.00	0.00	0.00	0.00	0.00
GH79	0.00	0.02	0.00	0.02	0.00	0.00	0.00	0.00	0.00	0.01	0.00	0.00	0.00
GT73	0.01	0.00	0.00	0.00	0.00	0.00	0.00	0.00	0.00	0.00	0.00	0.00	0.00
GH77	0.80	0.79	0.71	0.51	0.65	0.37	0.66	0.77	0.83	0.54	0.46	0.60	0.53
PL17	0.01	0.00	0.00	0.03	0.00	0.00	0.00	0.00	0.02	0.00	0.00	0.02	0.00
PL8	0.17	0.16	0.29	0.32	0.59	0.15	0.13	0.33	0.07	0.28	0.27	0.28	0.57

CAZY_group	AM-AD-75	AM-AD-76	AM-AD-77	AM-AD-78	AM-AD-79	AM-AD-8	AM-AD-80	AM-AD-81	AM-AD-82	AM-AD-83	AM-AD-84	AM-AD-85	AM-AD-86
**CE9**	0.40	0.37	0.38	0.33	0.47	0.68	0.27	0.50	0.41	0.30	0.28	0.56	0.38
**CE15**	0.02	0.00	0.00	0.03	0.02	0.00	0.01	0.01	0.01	0.00	0.01	0.00	0.00
**GT79**	0.00	0.00	0.00	0.00	0.00	0.00	0.00	0.00	0.00	0.00	0.00	0.00	0.00
**GT10**	0.01	0.00	0.02	0.01	0.00	0.00	0.01	0.01	0.00	0.00	0.03	0.02	0.00
**GH119**	0.00	0.00	0.00	0.00	0.00	0.00	0.00	0.00	0.00	0.00	0.00	0.00	0.00
**GH16**	0.13	0.19	0.18	0.19	0.28	0.22	0.13	0.17	0.05	0.26	0.16	0.24	0.37
**GH43**	1.83	1.87	1.73	2.05	3.35	2.03	1.42	1.80	0.88	1.56	2.01	2.45	2.46
**GH51**	0.64	0.58	0.41	0.65	0.64	0.58	0.46	0.43	0.33	0.59	0.58	0.47	0.42
**GT35**	1.17	1.13	0.96	0.93	1.03	1.14	1.07	1.10	1.11	0.84	0.81	1.01	0.88
**GT25**	0.00	0.00	0.00	0.00	0.00	0.00	0.01	0.00	0.02	0.00	0.00	0.00	0.05
**GT2**	4.98	4.81	4.31	4.22	4.79	3.72	4.11	4.62	3.95	4.30	3.89	3.81	5.24
**GT51**	1.03	0.95	0.89	0.92	1.00	0.95	0.76	0.97	0.92	1.00	0.89	0.93	1.11
**GH107**	0.00	0.00	0.00	0.00	0.00	0.00	0.00	0.00	0.00	0.00	0.00	0.00	0.00
**GH7**	0.00	0.00	0.00	0.00	0.00	0.00	0.00	0.00	0.00	0.00	0.00	0.00	0.00
**GT7**	0.00	0.00	0.00	0.00	0.00	0.00	0.00	0.00	0.00	0.00	0.00	0.00	0.00
**GT36**	0.00	0.00	0.00	0.00	0.00	0.00	0.00	0.00	0.00	0.00	0.00	0.00	0.00
**PL7**	0.00	0.00	0.00	0.00	0.00	0.00	0.00	0.00	0.00	0.00	0.00	0.00	0.00
**GH110**	0.09	0.07	0.07	0.13	0.27	0.09	0.05	0.12	0.01	0.10	0.12	0.06	0.13
**GT21**	0.00	0.00	0.00	0.00	0.00	0.00	0.00	0.00	0.00	0.00	0.00	0.00	0.00
**GH62**	0.00	0.00	0.00	0.00	0.00	0.00	0.01	0.01	0.00	0.00	0.00	0.00	0.00
**GH125**	0.14	0.15	0.13	0.17	0.22	0.12	0.12	0.18	0.10	0.11	0.18	0.19	0.17
**GH117**	0.08	0.07	0.08	0.08	0.13	0.09	0.05	0.07	0.02	0.15	0.12	0.06	0.10
**GH68**	0.00	0.00	0.00	0.00	0.00	0.00	0.00	0.00	0.00	0.00	0.00	0.00	0.00
**GH85**	0.02	0.03	0.05	0.01	0.01	0.00	0.03	0.00	0.02	0.00	0.01	0.06	0.05
**GH92**	1.18	1.24	1.63	1.53	2.49	1.45	0.94	1.70	0.76	1.14	1.90	2.15	1.83
**GH40**	0.00	0.00	0.00	0.00	0.00	0.00	0.00	0.00	0.00	0.00	0.00	0.00	0.00
**GT49**	0.00	0.00	0.00	0.01	0.00	0.00	0.00	0.00	0.00	0.00	0.00	0.00	0.00
**GH112**	0.30	0.26	0.20	0.16	0.20	0.12	0.20	0.21	0.28	0.20	0.10	0.41	0.22
**GT67**	0.00	0.00	0.00	0.00	0.00	0.00	0.00	0.00	0.00	0.00	0.00	0.00	0.00
**CE8**	0.16	0.21	0.28	0.21	0.38	0.34	0.15	0.16	0.14	0.14	0.16	0.13	0.27
**GT86**	0.00	0.00	0.00	0.00	0.00	0.00	0.00	0.00	0.00	0.00	0.00	0.00	0.00
**GH101**	0.11	0.03	0.01	0.00	0.02	0.00	0.02	0.02	0.00	0.02	0.01	0.15	0.05
**GH81**	0.01	0.01	0.01	0.00	0.01	0.00	0.02	0.01	0.01	0.00	0.00	0.00	0.00
**GT93**	0.00	0.00	0.00	0.00	0.00	0.00	0.00	0.00	0.00	0.00	0.00	0.00	0.00
**GT63**	0.00	0.00	0.00	0.00	0.00	0.00	0.00	0.00	0.00	0.00	0.00	0.00	0.00
**CE11**	0.20	0.20	0.20	0.25	0.22	0.31	0.22	0.19	0.19	0.28	0.28	0.13	0.33
**GT19**	0.10	0.09	0.13	0.13	0.10	0.22	0.13	0.11	0.08	0.17	0.20	0.09	0.22
**GT33**	0.00	0.00	0.00	0.00	0.00	0.00	0.00	0.00	0.00	0.00	0.00	0.00	0.00
**GH21**	0.00	0.00	0.00	0.00	0.00	0.00	0.00	0.00	0.00	0.00	0.00	0.00	0.00
**GT68**	0.00	0.00	0.00	0.00	0.00	0.00	0.00	0.00	0.00	0.00	0.00	0.00	0.00
**GH89**	0.20	0.15	0.31	0.26	0.47	0.34	0.11	0.20	0.08	0.25	0.24	0.45	0.23
**GT82**	0.00	0.00	0.00	0.00	0.00	0.00	0.00	0.00	0.00	0.00	0.00	0.00	0.00
**GH30**	0.19	0.23	0.18	0.20	0.44	0.46	0.17	0.21	0.06	0.13	0.15	0.30	0.42
**GH20**	1.03	0.92	1.16	1.16	1.71	1.29	0.85	1.24	0.42	1.11	1.26	1.08	1.10
**GH52**	0.00	0.00	0.00	0.00	0.00	0.00	0.00	0.00	0.00	0.00	0.00	0.00	0.00
**GT84**	0.01	0.04	0.01	0.03	0.00	0.00	0.01	0.00	0.00	0.00	0.01	0.00	0.00
**GT15**	0.00	0.00	0.00	0.00	0.00	0.00	0.00	0.00	0.00	0.00	0.00	0.00	0.00
**GH93**	0.01	0.00	0.01	0.00	0.01	0.00	0.02	0.01	0.01	0.00	0.00	0.00	0.00
**PL16**	0.00	0.00	0.00	0.00	0.00	0.00	0.00	0.00	0.00	0.00	0.00	0.00	0.00
**GH26**	0.08	0.20	0.15	0.08	0.15	0.00	0.10	0.09	0.05	0.08	0.12	0.04	0.18

CAZY_group	AM-AD-75	AM-AD-76	AM-AD-77	AM-AD-78	AM-AD-79	AM-AD-8	AM-AD-80	AM-AD-81	AM-AD-82	AM-AD-83	AM-AD-84	AM-AD-85	AM-AD-86
**GT75**	0.00	0.00	0.00	0.00	0.00	0.00	0.00	0.00	0.00	0.00	0.00	0.00	0.00
**GH53**	0.13	0.24	0.12	0.17	0.16	0.03	0.12	0.21	0.21	0.10	0.06	0.15	0.13
**GT88**	0.00	0.00	0.00	0.00	0.00	0.00	0.00	0.00	0.00	0.00	0.00	0.00	0.00
**GT24**	0.00	0.00	0.00	0.00	0.00	0.00	0.00	0.00	0.00	0.00	0.00	0.00	0.00
**GT81**	0.00	0.00	0.00	0.00	0.00	0.00	0.00	0.00	0.00	0.00	0.00	0.00	0.00
**GH4**	0.26	0.22	0.09	0.07	0.10	0.49	0.10	0.19	0.26	0.06	0.11	0.28	0.15
**GT78**	0.00	0.00	0.00	0.00	0.00	0.00	0.00	0.00	0.00	0.00	0.00	0.00	0.00
**GH8**	0.07	0.11	0.05	0.07	0.03	0.03	0.04	0.03	0.02	0.04	0.07	0.04	0.13
**GH70**	0.00	0.00	0.00	0.00	0.00	0.00	0.00	0.00	0.00	0.00	0.00	0.00	0.00
**GH122**	0.00	0.00	0.00	0.00	0.00	0.00	0.00	0.00	0.00	0.00	0.00	0.00	0.00
**GH74**	0.02	0.07	0.05	0.02	0.02	0.00	0.01	0.02	0.02	0.02	0.05	0.00	0.00
**GT41**	0.02	0.01	0.02	0.01	0.02	0.03	0.00	0.03	0.06	0.00	0.01	0.00	0.05
**PL1**	0.16	0.30	0.36	0.29	0.58	0.37	0.21	0.37	0.07	0.17	0.17	0.45	0.30
**GH15**	0.02	0.05	0.04	0.04	0.05	0.06	0.03	0.01	0.01	0.03	0.06	0.04	0.02
**GH32**	0.83	0.58	0.39	0.43	0.69	0.68	0.42	0.45	0.47	0.47	0.40	0.67	0.40
**GH44**	0.00	0.01	0.00	0.01	0.01	0.00	0.00	0.00	0.00	0.00	0.00	0.00	0.00
**GT6**	0.03	0.00	0.01	0.01	0.02	0.00	0.01	0.01	0.00	0.01	0.00	0.00	0.02
**GH13**	4.18	3.66	3.16	2.82	2.99	1.88	3.24	3.41	3.86	2.76	2.42	3.38	3.00
**GT80**	0.02	0.01	0.02	0.01	0.03	0.03	0.02	0.02	0.02	0.01	0.03	0.00	0.03
**GH71**	0.00	0.00	0.00	0.00	0.00	0.00	0.00	0.00	0.00	0.00	0.00	0.00	0.00
**GH63**	0.09	0.13	0.10	0.05	0.08	0.22	0.06	0.09	0.07	0.08	0.04	0.04	0.10
**PL21**	0.01	0.01	0.01	0.04	0.00	0.00	0.03	0.00	0.00	0.03	0.02	0.00	0.00
**GH65**	0.12	0.20	0.10	0.16	0.20	0.09	0.14	0.22	0.11	0.09	0.12	0.09	0.32
**CE4**	0.80	0.82	0.62	0.55	0.61	0.52	0.56	0.52	0.63	0.54	0.50	0.62	0.58
**CE14**	0.02	0.01	0.02	0.03	0.01	0.03	0.01	0.01	0.01	0.00	0.01	0.02	0.02
**GH115**	0.29	0.31	0.31	0.28	0.50	0.52	0.14	0.26	0.10	0.26	0.24	0.43	0.33
**GH104**	0.00	0.01	0.00	0.00	0.00	0.00	0.00	0.00	0.00	0.00	0.00	0.00	0.00
**GH111**	0.01	0.00	0.00	0.00	0.00	0.00	0.00	0.00	0.00	0.00	0.00	0.00	0.00
**GH95**	0.52	0.57	0.58	0.57	1.03	0.49	0.35	0.51	0.18	0.62	0.63	1.01	0.60
**GT70**	0.00	0.00	0.00	0.00	0.00	0.00	0.00	0.00	0.00	0.00	0.00	0.00	0.00
**CE10**	0.15	0.15	0.15	0.18	0.18	0.18	0.10	0.21	0.14	0.18	0.16	0.19	0.18
**GH10**	0.11	0.23	0.09	0.16	0.12	0.12	0.04	0.08	0.06	0.08	0.16	0.15	0.10
**GH130**	0.21	0.44	0.35	0.28	0.43	0.25	0.27	0.33	0.15	0.25	0.31	0.30	0.23
**PL20**	0.00	0.00	0.00	0.00	0.00	0.00	0.00	0.00	0.00	0.00	0.00	0.00	0.00
**GH69**	0.00	0.00	0.00	0.00	0.00	0.00	0.00	0.00	0.00	0.00	0.00	0.00	0.00
**PL2**	0.00	0.00	0.00	0.00	0.00	0.00	0.00	0.00	0.00	0.00	0.00	0.00	0.00
**GH49**	0.00	0.00	0.00	0.00	0.00	0.00	0.00	0.00	0.00	0.00	0.00	0.00	0.00
**GH25**	0.45	0.61	0.46	0.27	0.38	0.34	0.32	0.42	0.70	0.34	0.27	0.54	0.47
**GH59**	0.01	0.05	0.03	0.02	0.01	0.00	0.04	0.08	0.06	0.01	0.02	0.00	0.00
**GH35**	0.29	0.28	0.33	0.38	0.49	0.49	0.28	0.39	0.17	0.41	0.39	0.54	0.45
**GT39**	0.01	0.03	0.00	0.01	0.00	0.00	0.02	0.02	0.00	0.01	0.01	0.00	0.00
**GT57**	0.00	0.00	0.00	0.00	0.00	0.00	0.00	0.00	0.00	0.00	0.00	0.00	0.00
**GH129**	0.04	0.01	0.00	0.00	0.00	0.00	0.02	0.00	0.00	0.02	0.01	0.02	0.00
**GT22**	0.00	0.00	0.00	0.00	0.00	0.00	0.00	0.00	0.00	0.00	0.00	0.00	0.00
**PL12**	0.07	0.12	0.16	0.19	0.16	0.28	0.08	0.12	0.06	0.14	0.11	0.06	0.08
**PL18**	0.00	0.00	0.00	0.00	0.00	0.00	0.00	0.00	0.00	0.00	0.00	0.00	0.00
**GT92**	0.00	0.00	0.00	0.00	0.00	0.00	0.00	0.00	0.00	0.00	0.00	0.00	0.00
**GT44**	0.00	0.00	0.00	0.00	0.01	0.00	0.00	0.00	0.00	0.00	0.00	0.00	0.00
**GT29**	0.00	0.00	0.00	0.00	0.00	0.00	0.00	0.00	0.00	0.00	0.00	0.00	0.00
**GT13**	0.00	0.00	0.00	0.01	0.00	0.00	0.00	0.00	0.00	0.00	0.00	0.00	0.00

CAZY_group	AM-AD-75	AM-AD-76	AM-AD-77	AM-AD-78	AM-AD-79	AM-AD-8	AM-AD-80	AM-AD-81	AM-AD-82	AM-AD-83	AM-AD-84	AM-AD-85	AM-AD-86
**GT52**	0.00	0.00	0.00	0.00	0.00	0.00	0.00	0.00	0.02	0.00	0.00	0.00	0.00
**GT64**	0.00	0.00	0.00	0.00	0.00	0.00	0.00	0.00	0.00	0.00	0.00	0.00	0.00
**GT85**	0.00	0.00	0.00	0.00	0.00	0.00	0.00	0.00	0.00	0.00	0.00	0.00	0.00
**GT16**	0.00	0.00	0.00	0.00	0.00	0.00	0.00	0.00	0.00	0.00	0.00	0.00	0.00
**GH56**	0.00	0.00	0.00	0.00	0.00	0.00	0.00	0.00	0.00	0.00	0.00	0.00	0.00
**GT60**	0.00	0.00	0.00	0.00	0.00	0.00	0.00	0.00	0.00	0.00	0.00	0.00	0.00
**GH41**	0.00	0.00	0.00	0.00	0.00	0.00	0.00	0.00	0.00	0.00	0.00	0.00	0.00
**CE7**	0.12	0.12	0.09	0.09	0.14	0.12	0.04	0.10	0.10	0.04	0.09	0.06	0.13
**GH39**	0.06	0.06	0.06	0.06	0.05	0.03	0.04	0.02	0.02	0.02	0.02	0.02	0.02
**GT30**	0.19	0.16	0.24	0.19	0.28	0.40	0.23	0.22	0.15	0.24	0.31	0.22	0.28
**PL15**	0.06	0.16	0.14	0.09	0.20	0.22	0.06	0.07	0.03	0.10	0.15	0.26	0.08
**GH102**	0.02	0.01	0.01	0.02	0.00	0.00	0.00	0.00	0.06	0.00	0.01	0.00	0.00

CAZY_group	AM-AD-87	AM-AD-88	AM-AD-89	AM-AD-9	AM-AD-90	AM-AD-91	AM-AD-92	DA-AD-1	DA-AD-10	DA-AD-11	DA-AD-12	DA-AD-13	DA-AD-14
**GH29**	0.66	0.69	0.61	0.93	1.12	0.85	0.68	0.70	0.61	0.58	0.57	1.58	0.64
**PL4**	0.00	0.00	0.00	0.00	0.00	0.00	0.00	0.00	0.00	0.00	0.00	0.00	0.00
**GH23**	1.25	1.40	1.30	1.32	1.40	1.20	1.34	1.29	1.28	1.24	0.78	1.52	0.99
**CE6**	0.02	0.02	0.02	0.09	0.08	0.02	0.04	0.07	0.00	0.03	0.05	0.03	0.02
**GH72**	0.14	0.17	0.15	0.15	0.17	0.12	0.17	0.22	0.26	0.26	0.10	0.06	0.08
**GH114**	0.00	0.00	0.00	0.00	0.00	0.00	0.00	0.00	0.00	0.00	0.00	0.00	0.00
**GH78**	1.04	1.29	0.78	1.44	1.28	0.85	1.13	0.73	0.38	0.76	0.38	1.18	0.51
**PL19**	0.00	0.00	0.00	0.00	0.00	0.00	0.00	0.00	0.00	0.00	0.00	0.00	0.00
**GT27**	0.00	0.00	0.00	0.00	0.00	0.00	0.00	0.00	0.00	0.00	0.00	0.00	0.00
**GH5**	0.46	0.59	0.37	0.21	0.37	0.18	0.50	0.57	0.93	0.39	0.47	0.39	0.40
**GT40**	0.00	0.00	0.00	0.00	0.00	0.00	0.00	0.00	0.00	0.00	0.00	0.00	0.00
**CE13**	0.00	0.00	0.00	0.00	0.00	0.00	0.00	0.00	0.00	0.00	0.00	0.00	0.00
**GT37**	0.00	0.00	0.00	0.00	0.00	0.00	0.00	0.00	0.00	0.00	0.00	0.00	0.00
**GH33**	0.17	0.15	0.20	0.39	0.52	0.51	0.28	0.13	0.13	0.26	0.21	0.20	0.24
**GT65**	0.00	0.00	0.00	0.00	0.00	0.00	0.00	0.00	0.00	0.00	0.00	0.00	0.00
**GH86**	0.00	0.00	0.00	0.00	0.00	0.00	0.00	0.01	0.00	0.00	0.00	0.00	0.00
**GH123**	0.09	0.17	0.05	0.30	0.19	0.12	0.15	0.13	0.06	0.08	0.11	0.17	0.05
**GH96**	0.00	0.00	0.00	0.00	0.00	0.00	0.00	0.00	0.00	0.00	0.00	0.00	0.00
**GH14**	0.00	0.00	0.00	0.00	0.00	0.00	0.00	0.00	0.00	0.00	0.00	0.00	0.00
**CE3**	0.00	0.00	0.00	0.00	0.00	0.00	0.00	0.00	0.00	0.00	0.00	0.00	0.00
**PL10**	0.07	0.18	0.11	0.09	0.08	0.30	0.11	0.10	0.13	0.05	0.07	0.11	0.05
**GT48**	0.00	0.00	0.00	0.00	0.00	0.00	0.00	0.00	0.00	0.00	0.00	0.00	0.00
**PL6**	0.00	0.00	0.01	0.00	0.00	0.00	0.00	0.00	0.00	0.00	0.00	0.00	0.00
**GT83**	0.09	0.12	0.06	0.12	0.19	0.25	0.08	0.11	0.00	0.05	0.05	0.08	0.07
**GH126**	0.00	0.00	0.00	0.00	0.00	0.00	0.00	0.00	0.00	0.00	0.00	0.00	0.00
**GH9**	0.11	0.21	0.05	0.00	0.22	0.18	0.17	0.10	1.06	0.29	0.12	0.20	0.20
**GH75**	0.00	0.00	0.00	0.00	0.00	0.00	0.00	0.00	0.00	0.00	0.00	0.00	0.00
**GT71**	0.00	0.00	0.00	0.00	0.00	0.00	0.00	0.00	0.00	0.00	0.00	0.00	0.00
**GH46**	0.00	0.00	0.00	0.00	0.00	0.00	0.01	0.00	0.00	0.00	0.00	0.00	0.00
**GT61**	0.00	0.00	0.00	0.00	0.00	0.00	0.00	0.00	0.00	0.00	0.00	0.00	0.00
**GT3**	0.26	0.29	0.19	0.24	0.45	0.14	0.21	0.20	0.16	0.29	0.16	0.31	0.17
**PL3**	0.00	0.00	0.00	0.00	0.00	0.00	0.00	0.00	0.00	0.00	0.00	0.00	0.00
**GH108**	0.04	0.06	0.02	0.00	0.06	0.09	0.06	0.02	0.06	0.03	0.00	0.00	0.01
**GT56**	0.00	0.00	0.00	0.00	0.00	0.00	0.00	0.00	0.00	0.03	0.00	0.00	0.00
**PL11**	0.18	0.18	0.20	0.66	0.18	0.16	0.11	0.10	0.06	0.11	0.07	0.17	0.06
**GH45**	0.00	0.00	0.00	0.00	0.00	0.00	0.00	0.00	0.00	0.00	0.00	0.00	0.00
**GT34**	0.00	0.00	0.01	0.00	0.00	0.00	0.00	0.00	0.03	0.00	0.00	0.00	0.00
**PL14**	0.00	0.00	0.00	0.00	0.00	0.00	0.00	0.00	0.00	0.00	0.00	0.00	0.00
**GH19**	0.00	0.00	0.00	0.00	0.01	0.00	0.00	0.00	0.00	0.03	0.00	0.00	0.00
**GH118**	0.00	0.00	0.00	0.00	0.00	0.00	0.00	0.00	0.00	0.00	0.00	0.00	0.00
**GH82**	0.00	0.00	0.00	0.00	0.00	0.00	0.00	0.00	0.00	0.00	0.00	0.00	0.00
**GH76**	0.23	0.09	0.09	0.72	0.35	0.23	0.14	0.14	0.10	0.11	0.03	0.06	0.07
**GT4**	1.69	2.40	2.27	2.16	2.65	1.45	2.28	2.65	1.73	1.95	1.41	2.28	1.61
**GT87**	0.00	0.00	0.00	0.03	0.01	0.00	0.01	0.03	0.00	0.00	0.00	0.00	0.00
**GH24**	0.13	0.20	0.09	0.18	0.15	0.30	0.09	0.09	0.03	0.08	0.05	0.08	0.09
**GH90**	0.00	0.00	0.00	0.00	0.00	0.00	0.00	0.00	0.00	0.00	0.00	0.00	0.00
**GH57**	0.14	0.21	0.14	0.15	0.25	0.16	0.15	0.15	0.06	0.18	0.17	0.17	0.15
**GH36**	0.66	0.95	0.58	0.90	1.01	0.90	0.92	0.74	1.18	0.84	0.45	0.73	0.59
**GT46**	0.00	0.00	0.00	0.00	0.00	0.00	0.00	0.00	0.00	0.00	0.00	0.00	0.00
**GH66**	0.06	0.02	0.02	0.12	0.05	0.00	0.05	0.03	0.03	0.03	0.04	0.06	0.00

CAZY_group	AM-AD-87	AM-AD-88	AM-AD-89	AM-AD-9	AM-AD-90	AM-AD-91	AM-AD-92	DA-AD-1	DA-AD-10	DA-AD-11	DA-AD-12	DA-AD-13	DA-AD-14
GH48	0.00	0.00	0.00	0.00	0.00	0.00	0.00	0.00	0.00	0.00	0.00	0.00	0.00
GT53	0.00	0.00	0.00	0.00	0.00	0.00	0.00	0.00	0.00	0.00	0.00	0.00	0.00
GH60	0.00	0.00	0.00	0.00	0.00	0.00	0.00	0.00	0.00	0.00	0.00	0.00	0.00
GT31	0.00	0.00	0.00	0.00	0.00	0.00	0.00	0.00	0.00	0.00	0.00	0.00	0.00
GH105	0.45	0.66	0.49	1.14	0.85	0.64	0.60	0.49	0.58	0.55	0.28	1.15	0.26
GT23	0.00	0.02	0.02	0.00	0.02	0.00	0.01	0.01	0.06	0.00	0.01	0.03	0.00
GH11	0.00	0.00	0.00	0.00	0.00	0.00	0.01	0.00	0.06	0.00	0.00	0.03	0.01
GT38	0.00	0.00	0.00	0.00	0.00	0.00	0.00	0.00	0.00	0.00	0.00	0.00	0.00
GT43	0.00	0.00	0.00	0.00	0.00	0.00	0.00	0.00	0.00	0.00	0.00	0.00	0.00
GH99	0.01	0.00	0.00	0.03	0.02	0.00	0.00	0.00	0.00	0.00	0.00	0.00	0.00
GH38	0.33	0.62	0.22	0.39	0.33	0.12	0.35	0.20	0.26	0.21	0.05	0.45	0.20
GH109	0.26	0.26	0.12	0.42	0.41	0.30	0.24	0.25	0.13	0.29	0.19	0.54	0.20
GH2	4.41	5.61	3.81	6.10	5.68	4.28	4.56	4.44	3.87	4.50	2.33	6.87	3.16
GT28	0.36	0.47	0.61	0.42	0.50	0.35	0.56	0.32	0.77	0.53	0.39	0.37	0.40
GH42	0.14	0.29	0.14	0.21	0.36	0.16	0.28	0.20	0.58	0.45	0.07	0.25	0.14
GT58	0.00	0.00	0.00	0.00	0.00	0.00	0.00	0.00	0.00	0.00	0.00	0.00	0.00
GH61	0.00	0.00	0.00	0.00	0.00	0.00	0.00	0.00	0.00	0.00	0.00	0.00	0.00
GH80	0.00	0.00	0.00	0.00	0.00	0.00	0.00	0.00	0.00	0.00	0.00	0.00	0.00
GH98	0.00	0.00	0.00	0.00	0.00	0.00	0.02	0.00	0.03	0.00	0.00	0.00	0.00
GH6	0.00	0.00	0.00	0.00	0.00	0.00	0.00	0.00	0.00	0.00	0.00	0.00	0.00
GT1	0.02	0.03	0.07	0.06	0.03	0.07	0.02	0.06	0.13	0.05	0.06	0.03	0.00
GH28	0.75	0.78	0.79	1.26	1.12	0.78	0.99	0.92	0.96	0.84	0.48	1.30	0.44
GT42	0.00	0.00	0.00	0.00	0.00	0.00	0.00	0.00	0.00	0.00	0.00	0.00	0.00
GH128	0.00	0.00	0.00	0.00	0.00	0.00	0.00	0.00	0.00	0.00	0.00	0.00	0.00
CE12	0.09	0.14	0.24	0.15	0.19	0.00	0.13	0.12	0.26	0.16	0.05	0.06	0.05
CE16	0.00	0.00	0.00	0.00	0.00	0.00	0.00	0.00	0.00	0.00	0.00	0.00	0.00
GT89	0.00	0.00	0.00	0.00	0.00	0.00	0.00	0.00	0.00	0.00	0.00	0.00	0.00
GT11	0.07	0.03	0.08	0.06	0.11	0.07	0.09	0.15	0.10	0.05	0.04	0.11	0.03
GT77	0.00	0.00	0.00	0.00	0.00	0.00	0.00	0.00	0.03	0.00	0.00	0.00	0.00
GT55	0.00	0.00	0.00	0.00	0.00	0.00	0.00	0.00	0.00	0.00	0.00	0.00	0.00
GT18	0.00	0.00	0.00	0.00	0.00	0.00	0.00	0.00	0.00	0.00	0.00	0.00	0.00
GH87	0.00	0.02	0.04	0.00	0.01	0.00	0.00	0.00	0.00	0.00	0.00	0.00	0.00
PL5	0.00	0.00	0.00	0.00	0.00	0.00	0.00	0.00	0.00	0.00	0.00	0.00	0.00
GH22	0.00	0.00	0.01	0.00	0.00	0.00	0.00	0.00	0.00	0.00	0.00	0.00	0.00
CE2	0.14	0.15	0.08	0.03	0.10	0.05	0.09	0.03	0.22	0.08	0.05	0.06	0.08
GH120	0.08	0.12	0.08	0.00	0.00	0.07	0.05	0.11	0.10	0.08	0.06	0.00	0.05
GH73	0.48	0.78	0.59	0.48	0.77	0.48	0.72	0.55	1.09	0.58	0.44	0.62	0.54
GT17	0.01	0.00	0.00	0.00	0.00	0.00	0.03	0.03	0.00	0.00	0.01	0.00	0.00
GH124	0.00	0.00	0.00	0.00	0.00	0.00	0.00	0.00	0.00	0.00	0.00	0.00	0.00
GH54	0.00	0.00	0.00	0.00	0.00	0.00	0.00	0.00	0.00	0.00	0.01	0.00	0.00
GH100	0.00	0.00	0.00	0.00	0.00	0.00	0.00	0.00	0.00	0.00	0.00	0.00	0.00
GH113	0.02	0.03	0.01	0.00	0.03	0.02	0.06	0.03	0.10	0.08	0.02	0.08	0.04
GT5	0.52	0.42	0.46	0.18	0.55	0.41	0.63	0.53	0.80	0.42	0.41	0.42	0.45
GH94	0.50	0.54	0.43	0.18	0.55	0.35	0.61	0.41	1.41	0.76	0.41	0.62	0.43
GT66	0.00	0.00	0.00	0.00	0.00	0.00	0.00	0.01	0.00	0.00	0.00	0.00	0.02
GT94	0.00	0.00	0.00	0.00	0.00	0.00	0.00	0.00	0.00	0.00	0.00	0.00	0.00
GH58	0.00	0.00	0.00	0.00	0.00	0.00	0.00	0.00	0.00	0.00	0.00	0.00	0.00
GH1	0.46	0.35	0.72	0.06	0.58	0.35	0.54	0.65	0.67	1.16	0.33	0.25	0.31
PL22	0.00	0.00	0.00	0.00	0.00	0.00	0.00	0.00	0.00	0.03	0.00	0.00	0.00

CAZY_group	AM-AD-87	AM-AD-88	AM-AD-89	AM-AD-9	AM-AD-90	AM-AD-91	AM-AD-92	DA-AD-1	DA-AD-10	DA-AD-11	DA-AD-12	DA-AD-13	DA-AD-14
GT76	0.00	0.00	0.00	0.00	0.00	0.00	0.00	0.00	0.00	0.00	0.00	0.00	0.00
GT32	0.06	0.12	0.04	0.00	0.11	0.05	0.09	0.11	0.03	0.05	0.10	0.08	0.06
GT47	0.03	0.06	0.09	0.06	0.09	0.05	0.07	0.08	0.03	0.03	0.07	0.00	0.04
GH84	0.08	0.08	0.11	0.09	0.20	0.09	0.07	0.09	0.10	0.13	0.10	0.23	0.10
GH83	0.00	0.00	0.00	0.00	0.00	0.00	0.00	0.00	0.00	0.00	0.00	0.00	0.00
GT59	0.00	0.00	0.00	0.00	0.00	0.00	0.00	0.00	0.00	0.00	0.00	0.00	0.00
GH34	0.00	0.00	0.00	0.00	0.00	0.00	0.00	0.00	0.00	0.00	0.00	0.00	0.00
GT14	0.01	0.00	0.02	0.03	0.03	0.00	0.02	0.01	0.06	0.05	0.03	0.08	0.01
GH47	0.00	0.00	0.00	0.00	0.00	0.00	0.00	0.00	0.00	0.00	0.00	0.00	0.00
GH97	0.69	1.08	0.56	1.86	1.54	1.11	0.96	0.82	0.77	1.00	0.37	1.72	0.61
GH50	0.02	0.05	0.04	0.27	0.07	0.00	0.08	0.03	0.00	0.03	0.00	0.03	0.00
GT26	0.17	0.20	0.21	0.27	0.15	0.09	0.19	0.20	0.32	0.32	0.16	0.23	0.21
GH18	0.52	0.71	0.69	1.11	0.98	0.55	0.66	0.65	0.51	0.42	0.36	0.68	0.44
GH37	0.00	0.00	0.02	0.00	0.01	0.00	0.01	0.00	0.00	0.05	0.00	0.00	0.00
PL13	0.06	0.05	0.04	0.09	0.08	0.09	0.05	0.01	0.03	0.00	0.01	0.11	0.01
GH27	0.19	0.26	0.29	0.45	0.40	0.25	0.25	0.20	0.29	0.18	0.18	0.25	0.18
GT54	0.00	0.00	0.00	0.00	0.00	0.00	0.00	0.00	0.00	0.00	0.00	0.00	0.00
GT91	0.00	0.00	0.00	0.00	0.00	0.00	0.00	0.00	0.00	0.00	0.00	0.00	0.00
GT72	0.00	0.00	0.00	0.00	0.00	0.00	0.00	0.00	0.00	0.00	0.00	0.00	0.00
GH67	0.06	0.06	0.08	0.09	0.05	0.05	0.12	0.09	0.03	0.08	0.08	0.11	0.05
GH12	0.00	0.00	0.00	0.00	0.00	0.00	0.00	0.00	0.00	0.00	0.00	0.00	0.00
GH91	0.02	0.06	0.02	0.00	0.01	0.00	0.05	0.02	0.03	0.00	0.02	0.06	0.02
GT69	0.00	0.00	0.00	0.00	0.00	0.00	0.00	0.00	0.00	0.00	0.00	0.00	0.00
GH106	0.18	0.24	0.24	0.42	0.28	0.30	0.24	0.26	0.22	0.26	0.11	0.37	0.09
GT62	0.00	0.00	0.00	0.00	0.00	0.00	0.00	0.00	0.00	0.00	0.00	0.00	0.00
GH17	0.00	0.00	0.02	0.00	0.00	0.00	0.00	0.01	0.00	0.00	0.00	0.00	0.00
GH88	0.42	0.41	0.14	0.78	0.66	0.37	0.48	0.21	0.35	0.39	0.10	0.82	0.22
CE5	0.00	0.00	0.00	0.00	0.00	0.00	0.00	0.00	0.00	0.00	0.00	0.00	0.00
GT8	0.03	0.15	0.18	0.06	0.15	0.02	0.09	0.14	0.10	0.11	0.08	0.11	0.05
GT50	0.00	0.00	0.00	0.00	0.00	0.00	0.00	0.00	0.00	0.00	0.00	0.00	0.00
GT20	0.11	0.12	0.08	0.09	0.17	0.16	0.08	0.08	0.06	0.08	0.06	0.17	0.05
PL9	0.04	0.09	0.14	0.12	0.14	0.02	0.06	0.13	0.22	0.13	0.01	0.08	0.00
GH55	0.02	0.02	0.04	0.00	0.07	0.12	0.08	0.01	0.10	0.05	0.00	0.06	0.01
GH64	0.00	0.00	0.02	0.00	0.00	0.00	0.01	0.00	0.00	0.00	0.01	0.00	0.00
GH127	0.26	0.35	0.21	0.42	0.48	0.35	0.34	0.42	0.22	0.34	0.17	0.56	0.32
GH103	0.00	0.00	0.02	0.00	0.00	0.00	0.00	0.00	0.00	0.03	0.01	0.00	0.01
GH3	2.88	4.58	2.21	3.34	3.58	3.34	3.21	2.77	3.26	2.92	1.52	4.42	2.12
GT45	0.00	0.00	0.00	0.00	0.00	0.00	0.00	0.00	0.00	0.00	0.00	0.00	0.00
GT74	0.00	0.00	0.00	0.00	0.00	0.00	0.00	0.00	0.00	0.00	0.00	0.00	0.00
GT90	0.00	0.00	0.00	0.00	0.00	0.00	0.01	0.00	0.00	0.00	0.00	0.00	0.00
GH116	0.06	0.05	0.01	0.00	0.05	0.07	0.03	0.03	0.13	0.16	0.05	0.20	0.07
GH31	0.90	1.47	0.77	1.26	1.44	0.69	0.98	0.87	1.50	1.40	0.58	1.46	0.84
GT12	0.00	0.00	0.00	0.00	0.00	0.00	0.00	0.00	0.00	0.00	0.00	0.00	0.00
CE1	0.46	0.57	0.30	0.36	0.65	0.32	0.43	0.43	0.70	0.53	0.28	0.45	0.35
GH121	0.02	0.00	0.00	0.00	0.00	0.00	0.00	0.00	0.00	0.05	0.00	0.00	0.00
GH79	0.00	0.00	0.00	0.00	0.00	0.00	0.00	0.01	0.00	0.00	0.00	0.00	0.00
GT73	0.00	0.00	0.00	0.00	0.00	0.00	0.00	0.00	0.00	0.00	0.01	0.00	0.00
GH77	0.55	0.65	0.65	0.48	0.67	0.62	0.71	0.91	1.15	0.79	0.43	0.68	0.61
PL17	0.00	0.00	0.01	0.00	0.00	0.00	0.01	0.00	0.00	0.00	0.00	0.00	0.01
PL8	0.24	0.41	0.12	0.30	0.43	0.51	0.24	0.13	0.06	0.18	0.06	0.59	0.18

CAZY_group	AM-AD-87	AM-AD-88	AM-AD-89	AM-AD-9	AM-AD-90	AM-AD-91	AM-AD-92	DA-AD-1	DA-AD-10	DA-AD-11	DA-AD-12	DA-AD-13	DA-AD-14
**CE9**	0.36	0.27	0.31	0.45	0.42	0.30	0.38	0.26	0.22	0.34	0.21	0.54	0.23
**CE15**	0.01	0.05	0.00	0.00	0.03	0.00	0.02	0.00	0.00	0.03	0.02	0.00	0.01
**GT79**	0.00	0.00	0.00	0.00	0.00	0.00	0.00	0.00	0.00	0.00	0.00	0.00	0.00
**GT10**	0.00	0.00	0.00	0.00	0.01	0.00	0.01	0.00	0.00	0.00	0.01	0.00	0.01
**GH119**	0.00	0.00	0.00	0.00	0.00	0.00	0.00	0.00	0.00	0.00	0.00	0.00	0.00
**GH16**	0.18	0.20	0.21	0.33	0.22	0.23	0.24	0.15	0.19	0.18	0.10	0.28	0.18
**GH43**	1.70	2.46	1.74	3.55	2.85	1.59	2.28	1.83	2.85	2.58	1.36	3.94	1.33
**GH51**	0.56	0.81	0.47	0.84	0.80	0.71	0.65	0.63	0.80	0.66	0.29	0.87	0.48
**GT35**	0.96	1.01	0.98	0.69	0.95	0.67	1.12	1.35	1.28	0.82	0.80	0.73	0.84
**GT25**	0.00	0.00	0.00	0.00	0.00	0.00	0.00	0.00	0.03	0.00	0.00	0.03	0.00
**GT2**	4.22	5.07	4.66	3.85	5.36	4.12	4.86	6.74	5.66	4.32	3.12	5.46	3.94
**GT51**	1.08	1.17	0.93	1.05	1.18	0.97	1.05	1.16	1.06	1.24	0.72	1.15	0.84
**GH107**	0.00	0.00	0.00	0.00	0.00	0.00	0.00	0.00	0.00	0.00	0.00	0.00	0.00
**GH7**	0.00	0.00	0.00	0.00	0.00	0.00	0.00	0.00	0.00	0.00	0.00	0.00	0.00
**GT7**	0.00	0.00	0.00	0.00	0.00	0.00	0.00	0.00	0.00	0.00	0.00	0.00	0.00
**GT36**	0.00	0.00	0.00	0.00	0.00	0.00	0.00	0.00	0.00	0.00	0.00	0.00	0.00
**PL7**	0.00	0.00	0.00	0.00	0.00	0.00	0.00	0.00	0.00	0.00	0.00	0.00	0.00
**GH110**	0.06	0.12	0.11	0.12	0.22	0.16	0.08	0.15	0.10	0.11	0.09	0.31	0.15
**GT21**	0.00	0.00	0.00	0.00	0.00	0.00	0.00	0.00	0.00	0.00	0.00	0.00	0.00
**GH62**	0.00	0.00	0.00	0.00	0.01	0.00	0.00	0.00	0.00	0.00	0.00	0.00	0.00
**GH125**	0.21	0.23	0.13	0.39	0.37	0.21	0.22	0.11	0.19	0.18	0.08	0.34	0.08
**GH117**	0.08	0.08	0.02	0.09	0.19	0.16	0.14	0.06	0.13	0.08	0.02	0.14	0.05
**GH68**	0.00	0.00	0.00	0.00	0.00	0.00	0.00	0.00	0.00	0.00	0.00	0.00	0.00
**GH85**	0.02	0.11	0.02	0.03	0.06	0.00	0.01	0.03	0.03	0.03	0.00	0.03	0.00
**GH92**	1.77	1.77	1.20	4.39	3.08	1.84	1.64	1.40	1.02	1.71	0.65	2.20	1.07
**GH40**	0.00	0.00	0.00	0.00	0.00	0.00	0.00	0.00	0.00	0.00	0.00	0.00	0.00
**GT49**	0.00	0.00	0.00	0.00	0.00	0.00	0.00	0.00	0.00	0.00	0.00	0.00	0.00
**GH112**	0.26	0.23	0.24	0.12	0.18	0.28	0.19	0.19	0.26	0.13	0.12	0.25	0.09
**GT67**	0.00	0.00	0.00	0.00	0.00	0.00	0.00	0.00	0.00	0.00	0.00	0.00	0.00
**CE8**	0.15	0.33	0.28	0.33	0.28	0.23	0.20	0.22	0.19	0.26	0.13	0.39	0.15
**GT86**	0.00	0.00	0.00	0.00	0.00	0.00	0.00	0.00	0.00	0.00	0.00	0.00	0.00
**GH101**	0.02	0.00	0.02	0.00	0.05	0.05	0.00	0.00	0.00	0.00	0.00	0.00	0.00
**GH81**	0.01	0.00	0.03	0.00	0.00	0.00	0.01	0.01	0.00	0.00	0.00	0.00	0.00
**GT93**	0.00	0.00	0.00	0.00	0.00	0.00	0.00	0.00	0.00	0.00	0.00	0.00	0.00
**GT63**	0.00	0.00	0.00	0.00	0.00	0.00	0.00	0.00	0.00	0.00	0.00	0.00	0.00
**CE11**	0.26	0.29	0.16	0.33	0.36	0.30	0.25	0.23	0.16	0.29	0.18	0.14	0.21
**GT19**	0.18	0.20	0.11	0.24	0.24	0.12	0.12	0.19	0.06	0.13	0.09	0.20	0.11
**GT33**	0.00	0.00	0.00	0.00	0.00	0.00	0.00	0.00	0.00	0.00	0.00	0.00	0.00
**GH21**	0.00	0.00	0.00	0.00	0.00	0.00	0.00	0.00	0.00	0.00	0.00	0.00	0.00
**GT68**	0.00	0.00	0.00	0.00	0.00	0.00	0.00	0.00	0.00	0.00	0.00	0.00	0.00
**GH89**	0.21	0.21	0.18	0.48	0.42	0.21	0.23	0.26	0.13	0.21	0.13	0.31	0.15
**GT82**	0.00	0.00	0.00	0.00	0.00	0.00	0.00	0.00	0.00	0.00	0.00	0.00	0.01
**GH30**	0.26	0.24	0.17	0.36	0.40	0.41	0.27	0.14	0.38	0.26	0.08	0.34	0.10
**GH20**	1.16	1.20	0.91	1.92	2.06	1.38	1.25	1.07	0.64	0.97	0.67	2.20	0.82
**GH52**	0.00	0.00	0.00	0.00	0.00	0.00	0.00	0.00	0.00	0.00	0.00	0.00	0.00
**GT84**	0.01	0.00	0.08	0.00	0.00	0.00	0.04	0.08	0.00	0.05	0.03	0.00	0.07
**GT15**	0.00	0.00	0.00	0.00	0.00	0.00	0.00	0.00	0.00	0.00	0.00	0.00	0.00
**GH93**	0.02	0.00	0.00	0.03	0.00	0.00	0.01	0.00	0.00	0.00	0.00	0.00	0.00
**PL16**	0.00	0.00	0.00	0.00	0.00	0.00	0.00	0.00	0.00	0.00	0.00	0.00	0.00
**GH26**	0.14	0.24	0.11	0.06	0.12	0.09	0.15	0.03	0.35	0.03	0.10	0.17	0.10

CAZY_group	AM-AD-87	AM-AD-88	AM-AD-89	AM-AD-9	AM-AD-90	AM-AD-91	AM-AD-92	DA-AD-1	DA-AD-10	DA-AD-11	DA-AD-12	DA-AD-13	DA-AD-14
**GT75**	0.00	0.00	0.00	0.00	0.00	0.00	0.00	0.00	0.00	0.00	0.00	0.00	0.00
**GH53**	0.12	0.18	0.06	0.06	0.29	0.18	0.14	0.13	0.61	0.26	0.13	0.25	0.11
**GT88**	0.00	0.00	0.00	0.00	0.00	0.00	0.00	0.00	0.00	0.00	0.00	0.00	0.00
**GT24**	0.00	0.00	0.00	0.00	0.00	0.00	0.00	0.00	0.00	0.00	0.00	0.00	0.00
**GT81**	0.00	0.00	0.00	0.00	0.00	0.00	0.00	0.00	0.00	0.00	0.00	0.00	0.00
**GH4**	0.20	0.08	0.18	0.03	0.11	0.05	0.11	0.23	0.03	0.32	0.11	0.11	0.08
**GT78**	0.00	0.00	0.00	0.00	0.00	0.00	0.00	0.00	0.00	0.00	0.00	0.00	0.00
**GH8**	0.05	0.08	0.06	0.00	0.07	0.02	0.05	0.04	0.06	0.13	0.05	0.17	0.06
**GH70**	0.00	0.00	0.00	0.00	0.01	0.00	0.00	0.00	0.00	0.00	0.00	0.00	0.00
**GH122**	0.00	0.00	0.00	0.00	0.00	0.00	0.00	0.00	0.00	0.00	0.00	0.00	0.00
**GH74**	0.05	0.08	0.02	0.00	0.06	0.00	0.04	0.02	0.06	0.00	0.04	0.06	0.02
**GT41**	0.01	0.02	0.01	0.06	0.01	0.00	0.01	0.00	0.00	0.00	0.01	0.06	0.01
**PL1**	0.17	0.24	0.35	0.60	0.45	0.16	0.28	0.21	0.22	0.18	0.09	0.51	0.08
**GH15**	0.06	0.02	0.05	0.06	0.06	0.09	0.05	0.07	0.06	0.00	0.04	0.06	0.02
**GH32**	0.47	0.45	0.29	0.81	0.75	0.25	0.50	0.38	0.83	0.42	0.34	0.51	0.31
**GH44**	0.01	0.00	0.00	0.00	0.00	0.00	0.00	0.00	0.00	0.00	0.00	0.00	0.00
**GT6**	0.00	0.02	0.02	0.00	0.01	0.00	0.01	0.00	0.00	0.00	0.00	0.00	0.00
**GH13**	2.89	3.03	3.47	2.10	3.89	2.67	3.53	3.64	5.69	4.29	2.52	3.83	3.04
**GT80**	0.02	0.02	0.02	0.03	0.05	0.02	0.03	0.00	0.00	0.00	0.01	0.03	0.02
**GH71**	0.00	0.00	0.00	0.00	0.00	0.00	0.00	0.00	0.00	0.00	0.00	0.00	0.00
**GH63**	0.06	0.12	0.09	0.09	0.15	0.07	0.16	0.18	0.19	0.18	0.09	0.20	0.07
**PL21**	0.02	0.00	0.00	0.00	0.00	0.21	0.00	0.04	0.00	0.00	0.01	0.17	0.00
**GH65**	0.27	0.23	0.20	0.09	0.19	0.30	0.24	0.13	0.03	0.16	0.09	0.14	0.06
**CE4**	0.58	0.59	0.66	0.45	0.53	0.46	0.66	0.57	0.96	0.84	0.55	0.73	0.58
**CE14**	0.02	0.03	0.00	0.00	0.06	0.00	0.01	0.06	0.00	0.03	0.03	0.00	0.01
**GH115**	0.26	0.33	0.21	0.60	0.46	0.25	0.36	0.25	0.19	0.21	0.13	0.59	0.19
**GH104**	0.00	0.00	0.00	0.00	0.00	0.00	0.00	0.00	0.00	0.00	0.00	0.00	0.00
**GH111**	0.00	0.00	0.00	0.00	0.00	0.00	0.00	0.00	0.00	0.00	0.00	0.00	0.00
**GH95**	0.46	0.57	0.56	1.08	0.87	0.64	0.53	0.59	0.51	0.42	0.33	1.21	0.48
**GT70**	0.00	0.00	0.00	0.00	0.00	0.00	0.00	0.00	0.00	0.00	0.00	0.00	0.00
**CE10**	0.22	0.20	0.18	0.42	0.28	0.21	0.19	0.13	0.22	0.18	0.07	0.31	0.12
**GH10**	0.08	0.18	0.08	0.06	0.23	0.09	0.21	0.18	0.42	0.11	0.05	0.31	0.12
**GH130**	0.41	0.47	0.24	0.39	0.53	0.25	0.41	0.35	0.38	0.26	0.18	0.59	0.22
**PL20**	0.00	0.00	0.00	0.00	0.00	0.00	0.00	0.00	0.00	0.00	0.00	0.00	0.00
**GH69**	0.00	0.00	0.00	0.00	0.00	0.00	0.00	0.00	0.00	0.00	0.00	0.00	0.00
**PL2**	0.00	0.00	0.00	0.00	0.00	0.00	0.00	0.00	0.00	0.00	0.00	0.00	0.00
**GH49**	0.00	0.00	0.00	0.00	0.00	0.00	0.00	0.00	0.00	0.00	0.00	0.00	0.00
**GH25**	0.36	0.36	0.50	0.18	0.33	0.32	0.47	0.43	1.09	0.50	0.36	0.54	0.28
**GH59**	0.04	0.06	0.01	0.00	0.00	0.00	0.02	0.04	0.03	0.00	0.01	0.03	0.02
**GH35**	0.44	0.41	0.30	0.69	0.48	0.41	0.38	0.29	0.26	0.29	0.18	0.54	0.25
**GT39**	0.04	0.02	0.01	0.00	0.00	0.00	0.01	0.00	0.00	0.00	0.01	0.00	0.00
**GT57**	0.00	0.00	0.00	0.00	0.00	0.00	0.00	0.00	0.00	0.00	0.00	0.00	0.00
**GH129**	0.00	0.00	0.00	0.00	0.00	0.00	0.00	0.00	0.00	0.00	0.00	0.00	0.01
**GT22**	0.00	0.00	0.00	0.00	0.00	0.00	0.01	0.00	0.00	0.00	0.00	0.00	0.00
**PL12**	0.14	0.02	0.05	0.30	0.14	0.30	0.12	0.09	0.06	0.00	0.04	0.34	0.02
**PL18**	0.00	0.00	0.00	0.00	0.00	0.00	0.00	0.00	0.00	0.00	0.00	0.00	0.00
**GT92**	0.00	0.00	0.00	0.00	0.00	0.00	0.00	0.00	0.00	0.00	0.00	0.00	0.00
**GT44**	0.00	0.00	0.00	0.00	0.00	0.00	0.00	0.00	0.00	0.00	0.00	0.00	0.00
**GT29**	0.00	0.00	0.00	0.00	0.00	0.00	0.00	0.01	0.00	0.00	0.00	0.00	0.00
**GT13**	0.00	0.00	0.00	0.00	0.00	0.00	0.00	0.00	0.00	0.00	0.00	0.00	0.00

CAZY_group	AM-AD-87	AM-AD-88	AM-AD-89	AM-AD-9	AM-AD-90	AM-AD-91	AM-AD-92	DA-AD-1	DA-AD-10	DA-AD-11	DA-AD-12	DA-AD-13	DA-AD-14
**GT52**	0.00	0.00	0.00	0.00	0.00	0.00	0.00	0.00	0.00	0.00	0.00	0.00	0.00
**GT64**	0.00	0.00	0.00	0.00	0.00	0.00	0.00	0.00	0.00	0.00	0.00	0.00	0.00
**GT85**	0.00	0.00	0.00	0.00	0.00	0.00	0.00	0.00	0.00	0.00	0.00	0.00	0.00
**GT16**	0.00	0.00	0.00	0.00	0.00	0.00	0.00	0.00	0.00	0.00	0.00	0.00	0.00
**GH56**	0.00	0.00	0.00	0.00	0.00	0.00	0.00	0.00	0.00	0.00	0.00	0.00	0.00
**GT60**	0.00	0.00	0.00	0.00	0.00	0.00	0.00	0.00	0.00	0.00	0.00	0.00	0.00
**GH41**	0.00	0.00	0.00	0.00	0.00	0.00	0.00	0.00	0.00	0.00	0.00	0.00	0.00
**CE7**	0.15	0.11	0.07	0.18	0.17	0.09	0.14	0.12	0.10	0.08	0.05	0.11	0.06
**GH39**	0.02	0.05	0.05	0.00	0.12	0.12	0.04	0.06	0.06	0.11	0.02	0.14	0.04
**GT30**	0.32	0.36	0.19	0.51	0.34	0.37	0.28	0.30	0.19	0.26	0.18	0.34	0.21
**PL15**	0.15	0.11	0.05	0.21	0.29	0.14	0.12	0.08	0.10	0.00	0.03	0.28	0.02
**GH102**	0.00	0.00	0.02	0.00	0.01	0.00	0.00	0.07	0.00	0.03	0.01	0.00	0.01

CAZY_group	DA-AD-15	DA-AD-16	DA-AD-17	DA-AD-18	DA-AD-19	DA-AD-2	DA-AD-20	DA-AD-21	DA-AD-22	DA-AD-23	DA-AD-24	DA-AD-25	DA-AD-26
**GH29**	0.58	0.63	0.39	0.69	1.56	0.80	0.64	0.50	0.74	0.55	0.28	0.51	0.85
**PL4**	0.00	0.00	0.00	0.00	0.00	0.01	0.00	0.00	0.00	0.00	0.00	0.00	0.00
**GH23**	0.79	1.04	0.99	1.27	0.87	1.55	1.52	1.06	1.24	0.48	0.97	0.89	1.09
**CE6**	0.02	0.00	0.01	0.00	0.12	0.03	0.02	0.01	0.01	0.00	0.08	0.00	0.04
**GH72**	0.05	0.09	0.09	0.09	0.17	0.15	0.07	0.05	0.16	0.24	0.04	0.13	0.19
**GH114**	0.00	0.00	0.00	0.00	0.00	0.00	0.00	0.00	0.00	0.00	0.00	0.00	0.00
**GH78**	0.59	0.77	0.71	0.90	0.46	0.86	1.05	0.62	1.26	0.27	0.00	0.79	0.55
**PL19**	0.00	0.00	0.00	0.00	0.00	0.00	0.00	0.00	0.00	0.00	0.00	0.00	0.00
**GT27**	0.00	0.00	0.00	0.00	0.00	0.00	0.00	0.00	0.00	0.00	0.00	0.00	0.00
**GH5**	0.51	0.63	0.39	0.93	0.58	0.38	0.52	0.25	0.55	0.58	0.77	0.87	0.48
**GT40**	0.00	0.00	0.00	0.00	0.00	0.00	0.00	0.00	0.00	0.00	0.00	0.00	0.00
**CE13**	0.00	0.00	0.00	0.00	0.00	0.00	0.00	0.00	0.00	0.00	0.00	0.00	0.00
**GT37**	0.00	0.00	0.00	0.00	0.00	0.00	0.00	0.00	0.00	0.00	0.00	0.00	0.00
**GH33**	0.17	0.09	0.20	0.18	0.12	0.33	0.29	0.09	0.23	0.14	0.04	0.11	0.19
**GT65**	0.00	0.00	0.00	0.00	0.00	0.00	0.00	0.00	0.00	0.00	0.00	0.00	0.00
**GH86**	0.00	0.00	0.00	0.00	0.00	0.00	0.00	0.00	0.00	0.00	0.00	0.00	0.01
**GH123**	0.08	0.06	0.09	0.12	0.12	0.23	0.20	0.09	0.12	0.03	0.04	0.08	0.11
**GH96**	0.00	0.00	0.00	0.00	0.00	0.00	0.00	0.00	0.00	0.00	0.00	0.00	0.00
**GH14**	0.00	0.00	0.00	0.00	0.00	0.00	0.00	0.00	0.00	0.00	0.00	0.00	0.00
**CE3**	0.00	0.00	0.00	0.00	0.00	0.00	0.00	0.00	0.00	0.00	0.00	0.00	0.00
**PL10**	0.04	0.12	0.11	0.09	0.29	0.11	0.07	0.08	0.25	0.10	0.04	0.11	0.09
**GT48**	0.00	0.00	0.00	0.00	0.00	0.00	0.00	0.00	0.00	0.00	0.00	0.00	0.00
**PL6**	0.00	0.00	0.00	0.00	0.00	0.00	0.00	0.00	0.00	0.00	0.00	0.00	0.00
**GT83**	0.03	0.15	0.11	0.00	0.12	0.07	0.05	0.13	0.17	0.00	0.00	0.06	0.10
**GH126**	0.00	0.00	0.00	0.00	0.00	0.00	0.00	0.00	0.00	0.00	0.00	0.00	0.00
**GH9**	0.22	0.33	0.10	0.45	0.00	0.29	0.25	0.12	0.22	0.17	0.40	0.40	0.12
**GH75**	0.00	0.00	0.00	0.00	0.00	0.00	0.00	0.00	0.00	0.00	0.00	0.00	0.00
**GT71**	0.00	0.00	0.00	0.00	0.00	0.00	0.00	0.00	0.00	0.00	0.00	0.00	0.00
**GH46**	0.01	0.00	0.00	0.00	0.00	0.00	0.00	0.00	0.00	0.00	0.00	0.00	0.00
**GT61**	0.00	0.00	0.00	0.00	0.00	0.00	0.00	0.00	0.00	0.00	0.00	0.00	0.00
**GT3**	0.11	0.42	0.15	0.60	0.17	0.21	0.22	0.15	0.23	0.21	0.00	0.21	0.28
**PL3**	0.00	0.00	0.00	0.00	0.00	0.00	0.00	0.00	0.00	0.00	0.00	0.00	0.00
**GH108**	0.01	0.06	0.04	0.00	0.12	0.01	0.07	0.03	0.01	0.00	0.00	0.06	0.05
**GT56**	0.01	0.00	0.00	0.00	0.00	0.00	0.00	0.00	0.00	0.00	0.00	0.00	0.01
**PL11**	0.09	0.12	0.15	0.06	0.12	0.06	0.22	0.06	0.25	0.00	0.04	0.04	0.09
**GH45**	0.00	0.00	0.00	0.00	0.06	0.00	0.00	0.00	0.00	0.00	0.00	0.00	0.00
**GT34**	0.00	0.00	0.00	0.00	0.00	0.01	0.00	0.00	0.00	0.00	0.00	0.00	0.00
**PL14**	0.00	0.00	0.00	0.00	0.00	0.00	0.00	0.00	0.00	0.00	0.00	0.00	0.00
**GH19**	0.01	0.00	0.00	0.00	0.06	0.00	0.00	0.00	0.00	0.00	0.04	0.00	0.00
**GH118**	0.00	0.00	0.00	0.00	0.00	0.00	0.00	0.00	0.00	0.00	0.00	0.00	0.00
**GH82**	0.00	0.00	0.00	0.00	0.00	0.00	0.00	0.00	0.00	0.00	0.00	0.00	0.00
**GH76**	0.12	0.18	0.18	0.06	0.06	0.16	0.27	0.14	0.31	0.00	0.00	0.08	0.09
**GT4**	1.69	1.97	1.59	1.66	2.90	2.28	1.89	1.61	2.34	1.51	1.01	2.03	2.02
**GT87**	0.00	0.00	0.01	0.00	0.00	0.00	0.00	0.00	0.00	0.00	0.00	0.00	0.01
**GH24**	0.09	0.09	0.09	0.18	0.35	0.13	0.05	0.08	0.12	0.10	0.04	0.04	0.23
**GH90**	0.00	0.00	0.00	0.00	0.00	0.00	0.00	0.00	0.00	0.00	0.00	0.00	0.00
**GH57**	0.10	0.09	0.07	0.18	0.23	0.18	0.25	0.06	0.19	0.10	0.16	0.09	0.17
**GH36**	0.67	1.07	0.80	1.03	0.81	0.76	0.93	0.56	0.76	1.06	0.60	0.62	0.70
**GT46**	0.00	0.00	0.00	0.00	0.00	0.00	0.00	0.00	0.00	0.00	0.00	0.00	0.00
**GH66**	0.01	0.03	0.05	0.03	0.00	0.05	0.02	0.00	0.06	0.00	0.04	0.04	0.03

CAZY_group	DA-AD-15	DA-AD-16	DA-AD-17	DA-AD-18	DA-AD-19	DA-AD-2	DA-AD-20	DA-AD-21	DA-AD-22	DA-AD-23	DA-AD-24	DA-AD-25	DA-AD-26
**GH48**	0.00	0.00	0.00	0.00	0.00	0.00	0.00	0.00	0.00	0.00	0.00	0.00	0.00
**GT53**	0.00	0.00	0.00	0.00	0.00	0.00	0.00	0.00	0.00	0.00	0.00	0.00	0.00
**GH60**	0.00	0.00	0.00	0.00	0.00	0.00	0.00	0.00	0.00	0.00	0.00	0.00	0.00
**GT31**	0.01	0.00	0.00	0.00	0.00	0.00	0.00	0.00	0.00	0.00	0.00	0.00	0.01
**GH105**	0.39	0.54	0.46	0.51	0.58	0.47	0.52	0.35	0.64	0.14	0.12	0.55	0.47
**GT23**	0.00	0.03	0.02	0.00	0.00	0.00	0.02	0.01	0.01	0.00	0.00	0.00	0.01
**GH11**	0.02	0.00	0.00	0.00	0.00	0.00	0.00	0.00	0.00	0.00	0.00	0.00	0.00
**GT38**	0.02	0.00	0.00	0.00	0.00	0.00	0.00	0.00	0.00	0.00	0.00	0.00	0.00
**GT43**	0.00	0.00	0.00	0.00	0.00	0.00	0.00	0.00	0.00	0.00	0.00	0.00	0.00
**GH99**	0.01	0.00	0.02	0.00	0.06	0.00	0.02	0.00	0.01	0.00	0.00	0.00	0.00
**GH38**	0.27	0.42	0.25	0.12	0.06	0.33	0.27	0.29	0.36	0.10	0.00	0.21	0.17
**GH109**	0.24	0.30	0.19	0.27	0.35	0.40	0.25	0.14	0.25	0.21	0.20	0.23	0.34
**GH2**	3.18	5.03	3.46	5.01	4.63	4.21	4.07	3.25	4.07	2.53	1.81	3.73	3.61
**GT28**	0.40	0.54	0.45	0.45	0.35	0.53	0.49	0.38	0.41	0.51	0.56	0.55	0.49
**GH42**	0.16	0.21	0.23	0.21	0.00	0.22	0.27	0.25	0.28	0.14	0.28	0.26	0.18
**GT58**	0.00	0.00	0.00	0.00	0.00	0.00	0.00	0.00	0.00	0.00	0.00	0.00	0.00
**GH61**	0.00	0.00	0.00	0.00	0.00	0.00	0.00	0.00	0.00	0.00	0.00	0.00	0.00
**GH80**	0.00	0.00	0.00	0.00	0.00	0.00	0.00	0.00	0.00	0.00	0.00	0.00	0.00
**GH98**	0.00	0.00	0.02	0.00	0.00	0.00	0.00	0.00	0.00	0.00	0.00	0.00	0.00
**GH6**	0.00	0.00	0.00	0.00	0.00	0.00	0.00	0.00	0.00	0.00	0.00	0.00	0.00
**GT1**	0.05	0.09	0.03	0.00	0.00	0.02	0.10	0.04	0.04	0.03	0.00	0.04	0.05
**GH28**	0.51	1.13	0.67	0.90	1.16	0.79	0.81	0.58	0.90	0.51	0.48	0.58	0.57
**GT42**	0.00	0.00	0.00	0.00	0.00	0.00	0.00	0.00	0.00	0.00	0.00	0.00	0.00
**GH128**	0.00	0.00	0.00	0.00	0.00	0.00	0.00	0.00	0.00	0.00	0.00	0.00	0.00
**CE12**	0.12	0.12	0.13	0.06	0.23	0.02	0.10	0.06	0.23	0.00	0.04	0.23	0.04
**CE16**	0.00	0.00	0.00	0.00	0.00	0.00	0.00	0.00	0.00	0.00	0.00	0.00	0.00
**GT89**	0.00	0.00	0.00	0.00	0.00	0.00	0.00	0.00	0.00	0.00	0.00	0.00	0.00
**GT11**	0.04	0.03	0.05	0.06	0.17	0.08	0.07	0.01	0.09	0.03	0.00	0.02	0.06
**GT77**	0.00	0.00	0.00	0.00	0.00	0.00	0.00	0.00	0.00	0.00	0.00	0.00	0.00
**GT55**	0.00	0.00	0.00	0.00	0.00	0.00	0.00	0.00	0.00	0.00	0.00	0.00	0.00
**GT18**	0.00	0.00	0.00	0.00	0.00	0.00	0.00	0.00	0.00	0.00	0.00	0.00	0.00
**GH87**	0.00	0.03	0.00	0.00	0.00	0.00	0.00	0.01	0.00	0.00	0.00	0.00	0.01
**PL5**	0.00	0.00	0.00	0.00	0.00	0.00	0.00	0.00	0.00	0.00	0.00	0.00	0.00
**GH22**	0.00	0.00	0.00	0.00	0.00	0.00	0.00	0.00	0.00	0.00	0.00	0.00	0.00
**CE2**	0.09	0.06	0.05	0.15	0.12	0.05	0.10	0.04	0.07	0.10	0.04	0.08	0.03
**GH120**	0.05	0.12	0.12	0.09	0.00	0.00	0.05	0.09	0.07	0.00	0.04	0.06	0.03
**GH73**	0.50	0.54	0.43	0.93	0.41	0.63	0.56	0.49	0.57	0.62	0.77	0.57	0.56
**GT17**	0.01	0.00	0.01	0.00	0.00	0.00	0.00	0.00	0.00	0.00	0.00	0.00	0.00
**GH124**	0.00	0.00	0.00	0.00	0.00	0.00	0.00	0.00	0.00	0.00	0.00	0.00	0.00
**GH54**	0.00	0.00	0.00	0.00	0.00	0.00	0.00	0.00	0.00	0.00	0.00	0.00	0.00
**GH100**	0.00	0.00	0.00	0.00	0.00	0.00	0.00	0.00	0.00	0.00	0.00	0.00	0.00
**GH113**	0.04	0.06	0.04	0.09	0.00	0.01	0.02	0.00	0.03	0.03	0.04	0.04	0.02
**GT5**	0.33	0.57	0.50	0.57	0.29	0.54	0.47	0.41	0.38	0.55	0.69	0.38	0.53
**GH94**	0.60	0.69	0.39	0.60	0.06	0.20	0.69	0.26	0.32	0.41	0.77	0.55	0.35
**GT66**	0.00	0.00	0.00	0.00	0.00	0.01	0.00	0.01	0.00	0.00	0.00	0.00	0.01
**GT94**	0.00	0.00	0.00	0.00	0.00	0.00	0.00	0.00	0.00	0.00	0.00	0.00	0.00
**GH58**	0.00	0.00	0.00	0.00	0.00	0.00	0.00	0.00	0.00	0.00	0.00	0.00	0.01
**GH1**	0.44	0.71	0.49	0.60	0.00	0.43	0.29	0.38	0.39	0.17	0.28	0.43	0.40
**PL22**	0.00	0.00	0.00	0.00	0.00	0.00	0.00	0.00	0.00	0.00	0.00	0.00	0.00

CAZY_group	DA-AD-15	DA-AD-16	DA-AD-17	DA-AD-18	DA-AD-19	DA-AD-2	DA-AD-20	DA-AD-21	DA-AD-22	DA-AD-23	DA-AD-24	DA-AD-25	DA-AD-26	
GT76	0.00	0.00	0.00	0.00	0.00	0.00	0.00	0.00	0.00	0.00	0.00	0.00	0.00	0.00
GT32	0.09	0.09	0.09	0.00	0.06	0.16	0.07	0.02	0.09	0.17	0.04	0.08	0.11	
GT47	0.00	0.06	0.04	0.06	0.12	0.07	0.07	0.02	0.06	0.00	0.00	0.00	0.04	
GH84	0.08	0.18	0.11	0.12	0.17	0.05	0.10	0.05	0.12	0.10	0.00	0.13	0.18	
GH83	0.00	0.00	0.00	0.00	0.00	0.00	0.00	0.00	0.00	0.00	0.00	0.00	0.00	
GT59	0.00	0.00	0.00	0.00	0.00	0.00	0.00	0.00	0.00	0.00	0.00	0.00	0.00	
GH34	0.00	0.00	0.00	0.00	0.00	0.00	0.00	0.00	0.00	0.00	0.00	0.00	0.00	
GT14	0.04	0.06	0.03	0.03	0.00	0.08	0.10	0.01	0.01	0.00	0.04	0.02	0.07	
GH47	0.00	0.00	0.00	0.00	0.00	0.00	0.00	0.00	0.00	0.00	0.00	0.00	0.00	
GH97	0.56	1.04	0.79	1.54	0.87	0.92	1.01	0.82	0.97	0.48	0.24	0.87	0.96	
GH50	0.02	0.00	0.02	0.00	0.00	0.02	0.10	0.01	0.06	0.00	0.00	0.06	0.00	
GT26	0.23	0.30	0.16	0.15	0.46	0.09	0.07	0.17	0.25	0.14	0.44	0.15	0.19	
GH18	0.60	0.45	0.62	0.48	0.41	0.56	0.74	0.42	0.58	0.51	0.24	0.60	0.51	
GH37	0.04	0.00	0.00	0.00	0.00	0.02	0.00	0.00	0.00	0.00	0.00	0.00	0.02	
PL13	0.03	0.06	0.03	0.09	0.00	0.00	0.05	0.03	0.06	0.00	0.00	0.04	0.02	
GH27	0.24	0.24	0.22	0.27	0.23	0.22	0.17	0.20	0.26	0.24	0.20	0.24	0.17	
GT54	0.00	0.00	0.00	0.00	0.00	0.00	0.00	0.00	0.00	0.00	0.00	0.00	0.00	
GT91	0.00	0.00	0.00	0.00	0.00	0.00	0.00	0.00	0.00	0.00	0.00	0.00	0.00	
GT72	0.00	0.00	0.00	0.00	0.00	0.00	0.00	0.00	0.00	0.00	0.00	0.00	0.00	
GH67	0.07	0.03	0.04	0.12	0.12	0.02	0.07	0.03	0.06	0.03	0.00	0.04	0.02	
GH12	0.00	0.00	0.00	0.00	0.00	0.00	0.00	0.00	0.00	0.00	0.00	0.00	0.00	
GH91	0.03	0.00	0.00	0.00	0.00	0.01	0.07	0.00	0.03	0.00	0.00	0.00	0.05	
GT69	0.00	0.00	0.00	0.00	0.00	0.00	0.00	0.00	0.00	0.00	0.00	0.00	0.00	
GH106	0.15	0.36	0.23	0.24	0.35	0.34	0.25	0.17	0.17	0.21	0.00	0.06	0.16	
GT62	0.00	0.00	0.00	0.00	0.00	0.00	0.00	0.00	0.00	0.00	0.00	0.00	0.00	
GH17	0.00	0.00	0.00	0.00	0.00	0.00	0.00	0.00	0.00	0.00	0.00	0.00	0.00	
GH88	0.21	0.27	0.37	0.48	0.17	0.37	0.27	0.23	0.36	0.00	0.04	0.32	0.29	
CE5	0.00	0.00	0.00	0.00	0.00	0.00	0.00	0.00	0.00	0.00	0.00	0.00	0.00	
GT8	0.13	0.03	0.11	0.15	0.06	0.16	0.05	0.02	0.15	0.03	0.16	0.04	0.14	
GT50	0.00	0.00	0.00	0.00	0.00	0.00	0.00	0.00	0.00	0.00	0.00	0.00	0.00	
GT20	0.09	0.18	0.05	0.06	0.06	0.17	0.07	0.07	0.10	0.00	0.00	0.06	0.06	
PL9	0.09	0.06	0.09	0.12	0.06	0.02	0.15	0.06	0.12	0.03	0.00	0.13	0.04	
GH55	0.03	0.09	0.04	0.09	0.06	0.02	0.02	0.01	0.04	0.00	0.00	0.04	0.03	
GH64	0.00	0.00	0.00	0.00	0.00	0.00	0.00	0.00	0.00	0.00	0.00	0.00	0.00	
GH127	0.30	0.30	0.35	0.42	0.29	0.26	0.27	0.39	0.47	0.10	0.24	0.15	0.32	
GH103	0.00	0.00	0.00	0.03	0.00	0.01	0.00	0.01	0.00	0.00	0.00	0.00	0.02	
GH3	2.12	2.95	2.74	3.47	2.32	2.26	2.70	2.15	3.30	1.85	1.85	2.99	1.92	
GT45	0.00	0.00	0.00	0.00	0.00	0.00	0.00	0.00	0.00	0.00	0.00	0.00	0.00	
GT74	0.00	0.00	0.00	0.00	0.00	0.00	0.00	0.00	0.00	0.00	0.00	0.00	0.00	
GT90	0.00	0.00	0.00	0.00	0.00	0.02	0.00	0.00	0.00	0.00	0.00	0.00	0.00	
GH116	0.07	0.12	0.09	0.09	0.12	0.14	0.02	0.04	0.09	0.00	0.00	0.02	0.10	
GH31	0.92	1.25	0.99	1.27	0.87	0.98	1.15	0.88	1.12	0.51	0.69	1.37	0.94	
GT12	0.00	0.00	0.00	0.00	0.00	0.00	0.00	0.00	0.00	0.00	0.00	0.00	0.00	
CE1	0.44	0.57	0.42	0.54	0.35	0.30	0.47	0.31	0.52	0.27	0.16	0.51	0.29	
GH121	0.00	0.00	0.00	0.00	0.00	0.01	0.00	0.00	0.00	0.00	0.00	0.00	0.00	
GH79	0.01	0.06	0.00	0.03	0.00	0.01	0.00	0.01	0.01	0.00	0.00	0.02	0.01	
GT73	0.00	0.00	0.00	0.00	0.00	0.00	0.00	0.00	0.00	0.00	0.00	0.00	0.00	
GH77	0.50	0.83	0.58	0.84	0.64	0.69	0.69	0.51	0.60	0.75	0.64	0.62	0.57	
PL17	0.00	0.00	0.00	0.00	0.06	0.00	0.00	0.00	0.00	0.00	0.00	0.00	0.00	
PL8	0.20	0.18	0.21	0.24	0.12	0.32	0.17	0.15	0.17	0.00	0.00	0.19	0.29	

CAZY_group	DA-AD-15	DA-AD-16	DA-AD-17	DA-AD-18	DA-AD-19	DA-AD-2	DA-AD-20	DA-AD-21	DA-AD-22	DA-AD-23	DA-AD-24	DA-AD-25	DA-AD-26
**CE9**	0.30	0.30	0.33	0.24	0.12	0.40	0.44	0.21	0.42	0.24	0.24	0.17	0.33
**CE15**	0.03	0.06	0.00	0.00	0.00	0.03	0.00	0.01	0.00	0.00	0.00	0.00	0.01
**GT79**	0.00	0.00	0.00	0.00	0.00	0.00	0.00	0.00	0.00	0.00	0.00	0.00	0.00
**GT10**	0.00	0.00	0.01	0.00	0.00	0.01	0.00	0.02	0.00	0.00	0.00	0.00	0.03
**GH119**	0.00	0.00	0.00	0.00	0.00	0.00	0.00	0.00	0.00	0.00	0.00	0.00	0.00
**GH16**	0.15	0.18	0.11	0.21	0.12	0.24	0.15	0.17	0.28	0.17	0.04	0.17	0.25
**GH43**	1.51	2.29	1.59	2.23	2.84	2.08	1.69	1.79	2.54	0.92	0.69	1.85	1.67
**GH51**	0.48	0.57	0.56	0.57	0.64	0.46	0.59	0.53	0.73	0.21	0.48	0.45	0.46
**GT35**	0.79	0.86	0.66	0.93	0.41	0.99	0.96	0.64	0.96	1.16	0.97	1.05	0.72
**GT25**	0.00	0.00	0.01	0.00	0.00	0.01	0.00	0.00	0.00	0.00	0.00	0.00	0.01
**GT2**	3.75	4.85	4.33	4.80	5.27	5.23	4.56	3.87	5.03	3.66	3.79	4.78	4.08
**GT51**	0.73	0.80	0.83	0.81	1.16	0.93	0.76	0.90	1.06	0.92	0.77	0.70	0.95
**GH107**	0.00	0.00	0.00	0.00	0.00	0.00	0.00	0.00	0.00	0.00	0.00	0.00	0.00
**GH7**	0.00	0.00	0.00	0.00	0.00	0.00	0.00	0.00	0.00	0.00	0.00	0.00	0.00
**GT7**	0.00	0.00	0.00	0.00	0.00	0.00	0.00	0.00	0.00	0.00	0.00	0.00	0.00
**GT36**	0.00	0.00	0.00	0.00	0.00	0.00	0.00	0.00	0.00	0.00	0.00	0.00	0.00
**PL7**	0.00	0.00	0.00	0.00	0.00	0.00	0.00	0.00	0.01	0.00	0.00	0.00	0.00
**GH110**	0.12	0.09	0.11	0.06	0.23	0.15	0.10	0.07	0.12	0.10	0.04	0.08	0.11
**GT21**	0.00	0.00	0.00	0.00	0.00	0.00	0.00	0.00	0.00	0.00	0.00	0.00	0.00
**GH62**	0.00	0.00	0.00	0.00	0.00	0.00	0.00	0.00	0.00	0.00	0.00	0.00	0.00
**GH125**	0.11	0.21	0.18	0.18	0.00	0.24	0.37	0.15	0.22	0.03	0.08	0.11	0.14
**GH117**	0.06	0.12	0.09	0.03	0.12	0.07	0.17	0.03	0.12	0.00	0.00	0.08	0.06
**GH68**	0.00	0.00	0.00	0.00	0.00	0.00	0.00	0.00	0.00	0.00	0.00	0.00	0.00
**GH85**	0.01	0.03	0.02	0.00	0.00	0.03	0.00	0.00	0.00	0.00	0.04	0.00	0.01
**GH92**	1.27	1.64	1.48	1.51	1.68	1.63	1.74	1.69	2.15	0.58	0.48	1.49	1.49
**GH40**	0.00	0.00	0.00	0.00	0.00	0.00	0.00	0.00	0.00	0.00	0.00	0.00	0.00
**GT49**	0.00	0.00	0.00	0.00	0.00	0.00	0.00	0.00	0.00	0.00	0.00	0.00	0.00
**GH112**	0.15	0.09	0.22	0.09	0.00	0.21	0.20	0.07	0.23	0.24	0.24	0.19	0.08
**GT67**	0.00	0.00	0.00	0.00	0.00	0.00	0.00	0.00	0.00	0.00	0.00	0.00	0.00
**CE8**	0.19	0.24	0.15	0.30	0.52	0.13	0.10	0.15	0.35	0.14	0.08	0.21	0.18
**GT86**	0.00	0.00	0.00	0.00	0.00	0.00	0.00	0.00	0.00	0.00	0.00	0.00	0.00
**GH101**	0.00	0.00	0.03	0.00	0.00	0.00	0.00	0.00	0.00	0.00	0.00	0.02	0.00
**GH81**	0.00	0.00	0.00	0.00	0.00	0.00	0.00	0.00	0.01	0.00	0.00	0.00	0.00
**GT93**	0.00	0.00	0.00	0.00	0.00	0.00	0.00	0.00	0.00	0.00	0.00	0.00	0.00
**GT63**	0.00	0.00	0.00	0.00	0.00	0.00	0.00	0.00	0.00	0.00	0.00	0.00	0.00
**CE11**	0.19	0.24	0.19	0.24	0.46	0.20	0.32	0.21	0.26	0.17	0.20	0.19	0.26
**GT19**	0.09	0.21	0.14	0.18	0.52	0.24	0.20	0.10	0.09	0.10	0.16	0.11	0.18
**GT33**	0.00	0.00	0.00	0.00	0.00	0.00	0.00	0.00	0.00	0.00	0.00	0.00	0.00
**GH21**	0.00	0.00	0.00	0.00	0.00	0.00	0.00	0.00	0.00	0.00	0.00	0.00	0.00
**GT68**	0.00	0.00	0.00	0.00	0.00	0.00	0.00	0.00	0.00	0.00	0.00	0.00	0.00
**GH89**	0.16	0.21	0.21	0.12	0.35	0.24	0.15	0.12	0.23	0.21	0.08	0.11	0.18
**GT82**	0.00	0.00	0.00	0.00	0.00	0.00	0.00	0.00	0.00	0.00	0.00	0.00	0.00
**GH30**	0.17	0.27	0.20	0.18	0.23	0.22	0.27	0.22	0.29	0.07	0.08	0.23	0.25
**GH20**	0.74	1.31	1.02	0.75	1.33	1.36	1.08	0.87	1.21	0.82	0.60	0.62	1.15
**GH52**	0.00	0.00	0.00	0.00	0.00	0.00	0.00	0.00	0.00	0.00	0.00	0.00	0.00
**GT84**	0.02	0.00	0.04	0.00	0.00	0.08	0.00	0.00	0.03	0.00	0.00	0.00	0.00
**GT15**	0.00	0.00	0.00	0.00	0.00	0.00	0.00	0.00	0.00	0.00	0.00	0.00	0.00
**GH93**	0.02	0.00	0.01	0.00	0.00	0.01	0.00	0.00	0.00	0.00	0.00	0.00	0.00
**PL16**	0.00	0.00	0.00	0.00	0.00	0.00	0.00	0.00	0.00	0.00	0.00	0.00	0.00
**GH26**	0.18	0.33	0.12	0.15	0.00	0.11	0.20	0.10	0.15	0.17	0.16	0.28	0.16

CAZY_group	DA-AD-15	DA-AD-16	DA-AD-17	DA-AD-18	DA-AD-19	DA-AD-2	DA-AD-20	DA-AD-21	DA-AD-22	DA-AD-23	DA-AD-24	DA-AD-25	DA-AD-26
**GT75**	0.00	0.00	0.00	0.00	0.00	0.00	0.00	0.00	0.00	0.00	0.00	0.00	0.00
**GH53**	0.19	0.27	0.19	0.33	0.06	0.11	0.25	0.09	0.12	0.38	0.48	0.21	0.13
**GT88**	0.00	0.00	0.00	0.00	0.00	0.00	0.00	0.00	0.00	0.00	0.00	0.00	0.00
**GT24**	0.00	0.00	0.00	0.00	0.00	0.00	0.00	0.00	0.00	0.00	0.00	0.00	0.00
**GT81**	0.00	0.00	0.00	0.00	0.00	0.00	0.00	0.00	0.00	0.00	0.00	0.00	0.00
**GH4**	0.12	0.15	0.10	0.12	0.06	0.37	0.05	0.12	0.09	0.10	0.12	0.09	0.18
**GT78**	0.00	0.00	0.00	0.00	0.00	0.00	0.00	0.00	0.00	0.00	0.00	0.00	0.00
**GH8**	0.07	0.06	0.03	0.06	0.12	0.06	0.07	0.04	0.10	0.07	0.12	0.11	0.05
**GH70**	0.00	0.03	0.00	0.00	0.00	0.00	0.00	0.00	0.00	0.00	0.00	0.02	0.00
**GH122**	0.00	0.00	0.00	0.00	0.00	0.00	0.00	0.00	0.00	0.00	0.00	0.00	0.00
**GH74**	0.03	0.06	0.04	0.03	0.00	0.00	0.00	0.00	0.01	0.03	0.00	0.02	0.02
**GT41**	0.01	0.06	0.01	0.00	0.06	0.00	0.02	0.00	0.01	0.00	0.00	0.00	0.01
**PL1**	0.21	0.06	0.25	0.36	0.52	0.16	0.34	0.13	0.47	0.21	0.04	0.26	0.15
**GH15**	0.03	0.06	0.03	0.00	0.00	0.03	0.00	0.02	0.07	0.00	0.00	0.06	0.05
**GH32**	0.35	0.33	0.43	0.36	0.58	0.36	0.52	0.53	0.42	0.31	0.36	0.38	0.50
**GH44**	0.01	0.00	0.01	0.00	0.00	0.00	0.00	0.00	0.00	0.00	0.00	0.00	0.00
**GT6**	0.01	0.00	0.01	0.00	0.00	0.00	0.00	0.00	0.00	0.00	0.00	0.00	0.00
**GH13**	2.96	4.08	3.29	4.16	2.32	3.12	3.75	2.75	3.21	3.73	4.39	3.39	3.03
**GT80**	0.03	0.03	0.02	0.00	0.06	0.05	0.02	0.01	0.01	0.00	0.00	0.00	0.01
**GH71**	0.00	0.00	0.00	0.00	0.00	0.00	0.00	0.00	0.00	0.00	0.00	0.00	0.00
**GH63**	0.08	0.18	0.09	0.18	0.12	0.14	0.15	0.07	0.17	0.07	0.00	0.19	0.12
**PL21**	0.02	0.00	0.03	0.12	0.00	0.00	0.00	0.00	0.00	0.00	0.00	0.00	0.01
**GH65**	0.12	0.06	0.12	0.15	0.00	0.21	0.07	0.10	0.13	0.00	0.04	0.15	0.08
**CE4**	0.61	0.63	0.61	0.60	0.46	0.56	0.52	0.42	0.64	0.68	0.64	0.58	0.56
**CE14**	0.01	0.00	0.04	0.03	0.00	0.02	0.05	0.01	0.01	0.07	0.00	0.04	0.01
**GH115**	0.15	0.39	0.24	0.24	0.23	0.16	0.12	0.19	0.33	0.10	0.16	0.11	0.15
**GH104**	0.01	0.00	0.00	0.00	0.00	0.00	0.00	0.00	0.00	0.00	0.00	0.00	0.01
**GH111**	0.00	0.00	0.00	0.00	0.00	0.00	0.00	0.00	0.00	0.00	0.00	0.00	0.00
**GH95**	0.41	0.45	0.44	0.75	1.10	0.62	0.44	0.45	0.64	0.38	0.20	0.43	0.49
**GT70**	0.00	0.00	0.00	0.00	0.00	0.00	0.00	0.00	0.00	0.00	0.00	0.00	0.00
**CE10**	0.15	0.27	0.18	0.18	0.17	0.14	0.20	0.19	0.28	0.14	0.12	0.09	0.14
**GH10**	0.12	0.03	0.09	0.18	0.00	0.09	0.15	0.04	0.16	0.03	0.08	0.13	0.06
**GH130**	0.35	0.48	0.32	0.54	0.23	0.26	0.42	0.32	0.45	0.31	0.32	0.36	0.24
**PL20**	0.00	0.00	0.00	0.00	0.00	0.00	0.00	0.00	0.00	0.00	0.00	0.00	0.00
**GH69**	0.00	0.00	0.00	0.00	0.00	0.00	0.00	0.00	0.00	0.00	0.00	0.00	0.00
**PL2**	0.00	0.00	0.00	0.00	0.00	0.00	0.00	0.00	0.00	0.00	0.00	0.00	0.00
**GH49**	0.00	0.00	0.00	0.00	0.00	0.00	0.00	0.00	0.00	0.00	0.00	0.00	0.00
**GH25**	0.23	0.42	0.47	0.30	0.75	0.21	0.42	0.29	0.44	0.55	0.60	0.41	0.40
**GH59**	0.04	0.00	0.01	0.09	0.00	0.02	0.00	0.00	0.03	0.03	0.00	0.02	0.03
**GH35**	0.30	0.15	0.31	0.36	0.46	0.40	0.20	0.31	0.38	0.21	0.08	0.17	0.38
**GT39**	0.00	0.00	0.00	0.00	0.00	0.00	0.00	0.00	0.00	0.00	0.00	0.00	0.00
**GT57**	0.00	0.00	0.00	0.00	0.00	0.00	0.00	0.00	0.00	0.00	0.00	0.00	0.00
**GH129**	0.00	0.00	0.01	0.00	0.00	0.00	0.00	0.00	0.00	0.00	0.00	0.00	0.04
**GT22**	0.00	0.00	0.00	0.00	0.00	0.00	0.00	0.00	0.00	0.00	0.00	0.00	0.00
**PL12**	0.07	0.06	0.11	0.15	0.00	0.07	0.17	0.05	0.09	0.00	0.00	0.06	0.05
**PL18**	0.00	0.00	0.00	0.00	0.00	0.00	0.00	0.00	0.00	0.00	0.00	0.00	0.00
**GT92**	0.00	0.00	0.00	0.00	0.00	0.00	0.00	0.00	0.00	0.00	0.00	0.00	0.00
**GT44**	0.00	0.00	0.00	0.00	0.00	0.00	0.00	0.00	0.00	0.00	0.00	0.00	0.00
**GT29**	0.00	0.00	0.00	0.00	0.00	0.00	0.00	0.00	0.00	0.00	0.00	0.00	0.00
**GT13**	0.00	0.00	0.00	0.00	0.00	0.00	0.00	0.00	0.00	0.00	0.00	0.00	0.00

CAZY_group	DA-AD-15	DA-AD-16	DA-AD-17	DA-AD-18	DA-AD-19	DA-AD-2	DA-AD-20	DA-AD-21	DA-AD-22	DA-AD-23	DA-AD-24	DA-AD-25	DA-AD-26
**GT52**	0.00	0.00	0.00	0.00	0.00	0.00	0.00	0.00	0.00	0.00	0.00	0.00	0.00
**GT64**	0.00	0.00	0.00	0.00	0.00	0.00	0.00	0.00	0.00	0.00	0.00	0.00	0.00
**GT85**	0.00	0.00	0.00	0.00	0.00	0.00	0.00	0.00	0.00	0.00	0.00	0.00	0.00
**GT16**	0.00	0.00	0.00	0.00	0.00	0.00	0.00	0.00	0.00	0.00	0.00	0.00	0.00
**GH56**	0.00	0.00	0.00	0.00	0.00	0.00	0.00	0.00	0.00	0.00	0.00	0.00	0.00
**GT60**	0.00	0.00	0.00	0.00	0.00	0.00	0.00	0.00	0.00	0.00	0.00	0.00	0.00
**GH41**	0.00	0.00	0.00	0.00	0.00	0.00	0.00	0.00	0.00	0.00	0.00	0.00	0.00
**CE7**	0.04	0.15	0.05	0.06	0.35	0.13	0.20	0.03	0.25	0.03	0.08	0.11	0.11
**GH39**	0.03	0.09	0.04	0.09	0.00	0.01	0.10	0.02	0.04	0.00	0.08	0.08	0.01
**GT30**	0.23	0.24	0.23	0.30	0.41	0.26	0.34	0.22	0.22	0.14	0.04	0.23	0.26
**PL15**	0.06	0.09	0.05	0.30	0.00	0.05	0.17	0.05	0.04	0.00	0.00	0.04	0.10
**GH102**	0.03	0.00	0.03	0.00	0.06	0.03	0.02	0.01	0.03	0.00	0.00	0.00	0.05

CAZY_group	DA-AD-27	DA-AD-28	DA-AD-29	DA-AD-3	DA-AD-30	DA-AD-31	DA-AD-32	DA-AD-33	DA-AD-34	DA-AD-35	DA-AD-36	DA-AD-37	DA-AD-38
**GH29**	0.42	1.03	0.70	0.60	0.47	0.45	0.55	0.49	0.61	0.50	0.64	0.48	0.66
**PL4**	0.00	0.00	0.00	0.00	0.00	0.00	0.00	0.00	0.00	0.00	0.00	0.00	0.00
**GH23**	1.00	1.39	0.92	1.40	0.66	1.06	0.96	0.96	0.86	0.83	1.01	0.90	0.88
**CE6**	0.03	0.00	0.00	0.02	0.04	0.00	0.01	0.00	0.01	0.01	0.01	0.01	0.05
**GH72**	0.09	0.15	0.12	0.18	0.18	0.15	0.07	0.14	0.14	0.07	0.10	0.12	0.12
**GH114**	0.00	0.00	0.00	0.00	0.00	0.00	0.00	0.00	0.00	0.00	0.00	0.00	0.00
**GH78**	0.39	1.03	0.57	0.79	0.26	0.59	0.54	0.46	0.72	0.55	0.69	0.60	0.97
**PL19**	0.00	0.00	0.00	0.00	0.00	0.00	0.00	0.00	0.00	0.00	0.00	0.00	0.00
**GT27**	0.00	0.00	0.00	0.00	0.00	0.00	0.00	0.00	0.00	0.00	0.00	0.00	0.00
**GH5**	0.48	0.83	0.37	0.30	0.56	0.59	0.49	0.25	0.62	0.64	0.18	0.35	0.56
**GT40**	0.00	0.00	0.00	0.00	0.00	0.00	0.00	0.00	0.00	0.00	0.00	0.00	0.00
**CE13**	0.00	0.00	0.00	0.00	0.00	0.00	0.00	0.00	0.00	0.00	0.00	0.00	0.00
**GT37**	0.00	0.00	0.00	0.00	0.00	0.00	0.00	0.00	0.00	0.00	0.00	0.00	0.00
**GH33**	0.10	0.31	0.09	0.18	0.09	0.21	0.11	0.11	0.15	0.15	0.23	0.16	0.24
**GT65**	0.00	0.00	0.00	0.00	0.00	0.00	0.00	0.00	0.00	0.00	0.00	0.00	0.00
**GH86**	0.00	0.00	0.01	0.00	0.00	0.00	0.00	0.00	0.01	0.00	0.00	0.00	0.00
**GH123**	0.04	0.15	0.07	0.10	0.09	0.09	0.04	0.05	0.11	0.09	0.08	0.11	0.14
**GH96**	0.00	0.00	0.00	0.00	0.00	0.00	0.00	0.00	0.00	0.00	0.00	0.00	0.00
**GH14**	0.00	0.00	0.00	0.00	0.00	0.00	0.00	0.00	0.00	0.01	0.00	0.00	0.00
**CE3**	0.00	0.00	0.00	0.00	0.01	0.00	0.00	0.00	0.00	0.00	0.00	0.00	0.00
**PL10**	0.04	0.15	0.11	0.07	0.07	0.02	0.07	0.08	0.07	0.09	0.05	0.10	0.12
**GT48**	0.00	0.00	0.00	0.00	0.00	0.00	0.00	0.00	0.00	0.00	0.00	0.00	0.00
**PL6**	0.00	0.00	0.00	0.00	0.00	0.00	0.00	0.00	0.00	0.01	0.00	0.00	0.00
**GT83**	0.06	0.05	0.07	0.09	0.03	0.04	0.04	0.08	0.08	0.09	0.12	0.07	0.12
**GH126**	0.00	0.00	0.00	0.00	0.00	0.00	0.00	0.00	0.00	0.00	0.00	0.00	0.00
**GH9**	0.31	0.52	0.24	0.11	0.16	0.19	0.33	0.11	0.29	0.20	0.09	0.29	0.12
**GH75**	0.00	0.00	0.00	0.00	0.00	0.00	0.00	0.00	0.00	0.00	0.00	0.00	0.00
**GT71**	0.00	0.00	0.00	0.00	0.00	0.00	0.00	0.00	0.00	0.00	0.00	0.00	0.00
**GH46**	0.00	0.00	0.00	0.00	0.00	0.00	0.00	0.00	0.00	0.00	0.00	0.00	0.00
**GT61**	0.00	0.00	0.00	0.00	0.00	0.00	0.00	0.00	0.00	0.00	0.00	0.00	0.00
**GT3**	0.16	0.31	0.24	0.22	0.14	0.21	0.13	0.11	0.11	0.23	0.20	0.14	0.19
**PL3**	0.00	0.00	0.00	0.00	0.00	0.00	0.00	0.00	0.00	0.01	0.00	0.00	0.00
**GH108**	0.09	0.10	0.05	0.01	0.00	0.02	0.05	0.08	0.03	0.01	0.04	0.05	0.04
**GT56**	0.00	0.00	0.01	0.01	0.00	0.00	0.00	0.00	0.00	0.00	0.00	0.00	0.00
**PL11**	0.04	0.21	0.07	0.04	0.00	0.13	0.05	0.11	0.15	0.13	0.15	0.07	0.24
**GH45**	0.00	0.00	0.00	0.00	0.00	0.00	0.00	0.00	0.01	0.00	0.00	0.01	0.00
**GT34**	0.00	0.00	0.00	0.00	0.00	0.00	0.00	0.00	0.00	0.01	0.00	0.00	0.00
**PL14**	0.00	0.00	0.00	0.00	0.00	0.00	0.00	0.00	0.00	0.00	0.00	0.00	0.00
**GH19**	0.00	0.00	0.01	0.00	0.00	0.00	0.00	0.00	0.00	0.00	0.01	0.00	0.00
**GH118**	0.00	0.00	0.00	0.00	0.00	0.00	0.00	0.00	0.00	0.00	0.00	0.00	0.00
**GH82**	0.00	0.00	0.00	0.00	0.00	0.00	0.00	0.00	0.00	0.00	0.00	0.00	0.00
**GH76**	0.12	0.41	0.07	0.00	0.03	0.06	0.12	0.00	0.17	0.22	0.12	0.20	0.31
**GT4**	1.60	2.74	1.52	1.51	1.23	1.87	1.73	1.89	2.09	1.62	1.40	1.52	2.20
**GT87**	0.01	0.00	0.00	0.01	0.00	0.00	0.00	0.00	0.00	0.00	0.00	0.00	0.00
**GH24**	0.06	0.10	0.09	0.06	0.09	0.11	0.07	0.14	0.06	0.03	0.06	0.10	0.04
**GH90**	0.00	0.00	0.00	0.00	0.00	0.00	0.00	0.00	0.00	0.00	0.00	0.00	0.00
**GH57**	0.10	0.05	0.16	0.10	0.09	0.11	0.09	0.25	0.07	0.08	0.13	0.10	0.10
**GH36**	0.61	1.14	0.57	0.72	0.77	0.72	0.65	1.07	0.70	0.74	0.72	0.53	0.56
**GT46**	0.00	0.00	0.00	0.00	0.00	0.00	0.00	0.00	0.00	0.00	0.00	0.00	0.00
**GH66**	0.00	0.05	0.02	0.02	0.00	0.00	0.01	0.00	0.02	0.05	0.04	0.05	0.06

CAZY_group	DA-AD-27	DA-AD-28	DA-AD-29	DA-AD-3	DA-AD-30	DA-AD-31	DA-AD-32	DA-AD-33	DA-AD-34	DA-AD-35	DA-AD-36	DA-AD-37	DA-AD-38
**GH48**	0.00	0.00	0.00	0.00	0.00	0.00	0.00	0.00	0.00	0.00	0.00	0.00	0.00
**GT53**	0.00	0.00	0.00	0.00	0.00	0.00	0.00	0.00	0.00	0.00	0.00	0.00	0.00
**GH60**	0.00	0.00	0.00	0.00	0.00	0.00	0.00	0.00	0.00	0.00	0.00	0.00	0.00
**GT31**	0.00	0.00	0.00	0.01	0.00	0.00	0.00	0.00	0.00	0.00	0.00	0.00	0.00
**GH105**	0.25	0.83	0.42	0.23	0.23	0.38	0.41	0.36	0.46	0.39	0.32	0.45	0.76
**GT23**	0.00	0.00	0.00	0.00	0.00	0.00	0.00	0.00	0.01	0.00	0.00	0.00	0.00
**GH11**	0.00	0.00	0.01	0.00	0.00	0.00	0.01	0.00	0.01	0.00	0.01	0.00	0.01
**GT38**	0.00	0.00	0.00	0.00	0.00	0.00	0.00	0.00	0.00	0.00	0.00	0.00	0.00
**GT43**	0.00	0.00	0.00	0.00	0.00	0.00	0.00	0.00	0.00	0.00	0.00	0.00	0.00
**GH99**	0.00	0.00	0.00	0.01	0.00	0.00	0.00	0.00	0.00	0.01	0.00	0.01	0.01
**GH38**	0.16	0.52	0.21	0.30	0.04	0.11	0.17	0.03	0.14	0.22	0.32	0.26	0.39
**GH109**	0.19	0.57	0.23	0.25	0.16	0.15	0.25	0.33	0.28	0.15	0.23	0.25	0.32
**GH2**	2.67	8.01	2.98	3.03	2.48	3.47	2.99	2.68	3.51	3.38	2.92	3.17	4.36
**GT28**	0.33	0.46	0.33	0.56	0.45	0.45	0.37	0.49	0.35	0.39	0.41	0.38	0.39
**GH42**	0.12	0.41	0.14	0.25	0.09	0.15	0.13	0.11	0.27	0.16	0.29	0.13	0.18
**GT58**	0.00	0.00	0.00	0.00	0.00	0.00	0.00	0.00	0.00	0.00	0.00	0.00	0.00
**GH61**	0.00	0.00	0.00	0.00	0.00	0.00	0.00	0.00	0.00	0.00	0.00	0.00	0.00
**GH80**	0.00	0.00	0.00	0.00	0.00	0.00	0.00	0.00	0.00	0.00	0.00	0.00	0.00
**GH98**	0.00	0.00	0.00	0.00	0.00	0.02	0.00	0.00	0.01	0.00	0.00	0.00	0.00
**GH6**	0.00	0.00	0.00	0.00	0.00	0.00	0.00	0.00	0.00	0.00	0.01	0.00	0.00
**GT1**	0.06	0.00	0.02	0.02	0.03	0.09	0.02	0.05	0.05	0.02	0.01	0.02	0.04
**GH28**	0.42	1.08	0.65	0.52	0.53	0.66	0.66	0.74	0.79	0.82	0.47	0.66	1.15
**GT42**	0.00	0.00	0.00	0.00	0.01	0.00	0.00	0.00	0.00	0.00	0.00	0.00	0.00
**GH128**	0.00	0.00	0.00	0.00	0.00	0.00	0.00	0.00	0.00	0.00	0.00	0.00	0.00
**CE12**	0.04	0.10	0.08	0.11	0.11	0.17	0.14	0.25	0.23	0.12	0.09	0.20	0.24
**CE16**	0.00	0.00	0.00	0.00	0.00	0.00	0.00	0.00	0.00	0.00	0.00	0.00	0.00
**GT89**	0.00	0.00	0.00	0.00	0.00	0.00	0.00	0.00	0.00	0.00	0.00	0.00	0.00
**GT11**	0.06	0.10	0.06	0.03	0.04	0.08	0.01	0.08	0.05	0.05	0.06	0.05	0.07
**GT77**	0.00	0.00	0.00	0.00	0.00	0.00	0.00	0.00	0.00	0.00	0.00	0.00	0.00
**GT55**	0.00	0.00	0.00	0.00	0.00	0.00	0.00	0.00	0.00	0.00	0.00	0.00	0.00
**GT18**	0.00	0.00	0.00	0.00	0.00	0.00	0.00	0.00	0.00	0.00	0.00	0.00	0.00
**GH87**	0.00	0.00	0.00	0.00	0.00	0.00	0.00	0.00	0.00	0.00	0.00	0.00	0.00
**PL5**	0.00	0.00	0.00	0.00	0.00	0.00	0.00	0.00	0.00	0.00	0.00	0.00	0.00
**GH22**	0.00	0.00	0.00	0.00	0.00	0.00	0.00	0.00	0.00	0.00	0.00	0.00	0.00
**CE2**	0.04	0.31	0.04	0.07	0.12	0.08	0.07	0.05	0.04	0.10	0.08	0.05	0.12
**GH120**	0.04	0.05	0.08	0.11	0.07	0.04	0.08	0.00	0.09	0.04	0.04	0.05	0.06
**GH73**	0.54	0.57	0.37	0.50	0.61	0.57	0.49	0.55	0.37	0.43	0.38	0.44	0.52
**GT17**	0.00	0.00	0.00	0.00	0.01	0.00	0.00	0.00	0.02	0.00	0.00	0.00	0.01
**GH124**	0.00	0.00	0.00	0.00	0.00	0.00	0.00	0.00	0.00	0.00	0.00	0.00	0.00
**GH54**	0.00	0.00	0.00	0.00	0.00	0.00	0.00	0.00	0.00	0.00	0.00	0.00	0.00
**GH100**	0.00	0.00	0.00	0.00	0.00	0.00	0.00	0.00	0.00	0.00	0.00	0.00	0.00
**GH113**	0.03	0.05	0.03	0.00	0.07	0.11	0.07	0.14	0.03	0.03	0.04	0.01	0.04
**GT5**	0.39	0.72	0.48	0.68	0.69	0.59	0.42	0.46	0.34	0.48	0.40	0.26	0.47
**GH94**	0.43	1.08	0.32	0.25	0.39	0.45	0.56	0.55	0.62	0.57	0.31	0.29	0.48
**GT66**	0.00	0.00	0.00	0.02	0.00	0.02	0.00	0.00	0.00	0.00	0.00	0.00	0.00
**GT94**	0.00	0.00	0.00	0.00	0.00	0.00	0.00	0.00	0.00	0.00	0.00	0.00	0.00
**GH58**	0.00	0.00	0.00	0.00	0.00	0.00	0.00	0.00	0.00	0.00	0.00	0.00	0.00
**GH1**	0.49	0.77	0.36	0.47	0.37	0.47	0.49	0.71	0.25	0.47	0.28	0.11	0.27
**PL22**	0.00	0.00	0.02	0.00	0.00	0.00	0.00	0.00	0.00	0.00	0.00	0.00	0.00

CAZY_group	DA-AD-27	DA-AD-28	DA-AD-29	DA-AD-3	DA-AD-30	DA-AD-31	DA-AD-32	DA-AD-33	DA-AD-34	DA-AD-35	DA-AD-36	DA-AD-37	DA-AD-38
GT76	0.00	0.00	0.00	0.00	0.00	0.00	0.00	0.00	0.00	0.00	0.00	0.00	0.00
GT32	0.09	0.05	0.05	0.05	0.05	0.09	0.12	0.08	0.13	0.07	0.06	0.05	0.09
GT47	0.01	0.00	0.01	0.12	0.04	0.04	0.03	0.05	0.04	0.05	0.05	0.05	0.05
GH84	0.09	0.21	0.11	0.10	0.07	0.08	0.05	0.08	0.11	0.02	0.08	0.07	0.08
GH83	0.00	0.00	0.00	0.00	0.00	0.00	0.00	0.00	0.00	0.00	0.00	0.00	0.00
GT59	0.00	0.00	0.00	0.00	0.00	0.00	0.00	0.00	0.00	0.00	0.00	0.00	0.00
GH34	0.00	0.00	0.00	0.00	0.00	0.00	0.00	0.00	0.00	0.00	0.00	0.00	0.00
GT14	0.01	0.15	0.02	0.00	0.04	0.02	0.06	0.00	0.02	0.02	0.01	0.02	0.01
GH47	0.00	0.00	0.00	0.00	0.00	0.00	0.00	0.00	0.00	0.00	0.00	0.00	0.00
GH97	0.46	1.14	0.52	0.72	0.50	0.43	0.54	0.38	0.69	0.74	0.58	0.68	0.96
GH50	0.00	0.00	0.00	0.00	0.00	0.00	0.04	0.00	0.04	0.05	0.08	0.02	0.06
GT26	0.16	0.46	0.21	0.15	0.24	0.19	0.19	0.30	0.23	0.22	0.18	0.19	0.20
GH18	0.46	0.46	0.39	0.57	0.37	0.42	0.40	0.52	0.51	0.61	0.42	0.45	0.79
GH37	0.00	0.00	0.03	0.01	0.00	0.00	0.01	0.00	0.00	0.00	0.03	0.00	0.00
PL13	0.00	0.05	0.02	0.00	0.01	0.00	0.00	0.00	0.02	0.02	0.05	0.04	0.06
GH27	0.19	0.52	0.14	0.26	0.19	0.08	0.20	0.08	0.24	0.23	0.24	0.19	0.34
GT54	0.00	0.00	0.00	0.00	0.00	0.00	0.00	0.00	0.00	0.00	0.00	0.00	0.00
GT91	0.00	0.00	0.00	0.00	0.00	0.00	0.00	0.00	0.00	0.00	0.00	0.00	0.00
GT72	0.00	0.00	0.00	0.00	0.00	0.00	0.00	0.00	0.00	0.00	0.00	0.00	0.00
GH67	0.03	0.05	0.06	0.07	0.11	0.04	0.04	0.16	0.12	0.05	0.01	0.04	0.09
GH12	0.00	0.00	0.00	0.00	0.00	0.00	0.00	0.00	0.00	0.00	0.00	0.00	0.00
GH91	0.01	0.05	0.01	0.00	0.00	0.02	0.01	0.00	0.01	0.04	0.01	0.02	0.04
GT69	0.00	0.00	0.00	0.00	0.00	0.00	0.00	0.00	0.00	0.00	0.00	0.00	0.00
GH106	0.10	0.41	0.16	0.08	0.11	0.28	0.16	0.19	0.17	0.23	0.23	0.17	0.25
GT62	0.00	0.00	0.00	0.00	0.00	0.00	0.00	0.00	0.00	0.00	0.00	0.00	0.00
GH17	0.00	0.00	0.00	0.00	0.00	0.00	0.00	0.00	0.00	0.00	0.00	0.00	0.00
GH88	0.16	0.72	0.25	0.12	0.07	0.26	0.25	0.22	0.27	0.25	0.23	0.32	0.52
CE5	0.00	0.00	0.00	0.00	0.00	0.00	0.00	0.00	0.00	0.00	0.00	0.00	0.00
GT8	0.04	0.00	0.04	0.12	0.08	0.19	0.10	0.16	0.15	0.12	0.14	0.05	0.13
GT50	0.00	0.00	0.00	0.00	0.00	0.00	0.00	0.00	0.00	0.00	0.00	0.00	0.00
GT20	0.07	0.15	0.08	0.01	0.03	0.08	0.05	0.05	0.08	0.05	0.06	0.07	0.04
PL9	0.07	0.05	0.03	0.00	0.04	0.09	0.11	0.22	0.15	0.12	0.00	0.15	0.16
GH55	0.03	0.10	0.01	0.01	0.00	0.02	0.02	0.00	0.03	0.00	0.03	0.02	0.04
GH64	0.00	0.00	0.00	0.00	0.00	0.00	0.00	0.00	0.00	0.00	0.00	0.00	0.00
GH127	0.12	0.36	0.22	0.24	0.22	0.19	0.24	0.33	0.31	0.32	0.19	0.24	0.25
GH103	0.00	0.00	0.01	0.01	0.00	0.00	0.00	0.00	0.00	0.00	0.00	0.00	0.00
GH3	1.71	4.49	2.14	2.46	1.80	1.79	2.00	1.01	2.54	2.16	2.15	2.11	2.92
GT45	0.00	0.00	0.00	0.00	0.00	0.00	0.00	0.00	0.00	0.00	0.00	0.00	0.00
GT74	0.00	0.00	0.00	0.00	0.00	0.00	0.00	0.00	0.00	0.00	0.00	0.00	0.00
GT90	0.00	0.00	0.00	0.00	0.00	0.00	0.00	0.00	0.00	0.00	0.01	0.00	0.00
GH116	0.10	0.31	0.02	0.02	0.00	0.11	0.07	0.00	0.10	0.00	0.01	0.07	0.08
GH31	0.70	2.01	0.73	0.84	0.79	0.51	1.01	0.66	1.18	0.88	0.82	0.87	1.03
GT12	0.00	0.00	0.00	0.00	0.00	0.00	0.00	0.00	0.00	0.00	0.00	0.00	0.00
CE1	0.27	0.67	0.40	0.26	0.30	0.40	0.36	0.41	0.35	0.34	0.35	0.30	0.40
GH121	0.00	0.00	0.00	0.08	0.00	0.00	0.00	0.00	0.00	0.00	0.00	0.00	0.00
GH79	0.01	0.10	0.00	0.00	0.01	0.02	0.03	0.00	0.02	0.00	0.00	0.00	0.04
GT73	0.00	0.00	0.01	0.00	0.00	0.00	0.00	0.00	0.00	0.00	0.00	0.00	0.00
GH77	0.46	0.88	0.56	1.00	0.60	0.70	0.55	0.60	0.50	0.55	0.51	0.44	0.59
PL17	0.00	0.00	0.00	0.03	0.00	0.00	0.01	0.00	0.01	0.00	0.00	0.00	0.00
PL8	0.21	0.26	0.17	0.09	0.12	0.09	0.16	0.08	0.11	0.14	0.13	0.36	0.37

CAZY_group	DA-AD-27	DA-AD-28	DA-AD-29	DA-AD-3	DA-AD-30	DA-AD-31	DA-AD-32	DA-AD-33	DA-AD-34	DA-AD-35	DA-AD-36	DA-AD-37	DA-AD-38
**CE9**	0.16	0.41	0.27	0.41	0.19	0.38	0.14	0.22	0.22	0.29	0.23	0.20	0.39
**CE15**	0.00	0.05	0.01	0.03	0.00	0.02	0.01	0.03	0.01	0.03	0.01	0.02	0.05
**GT79**	0.00	0.00	0.00	0.00	0.00	0.00	0.00	0.00	0.00	0.00	0.00	0.00	0.00
**GT10**	0.00	0.00	0.00	0.00	0.00	0.00	0.01	0.00	0.02	0.00	0.00	0.00	0.01
**GH119**	0.00	0.00	0.00	0.00	0.00	0.00	0.00	0.00	0.00	0.00	0.00	0.00	0.00
**GH16**	0.15	0.41	0.18	0.11	0.08	0.09	0.15	0.11	0.18	0.20	0.14	0.20	0.22
**GH43**	1.33	2.84	1.46	1.46	1.41	1.51	1.75	1.91	2.53	1.78	1.35	1.70	2.54
**GH51**	0.33	0.77	0.32	0.35	0.42	0.26	0.36	0.36	0.47	0.52	0.42	0.44	0.56
**GT35**	0.91	0.93	0.75	1.19	0.98	1.00	0.95	0.98	0.94	0.84	0.94	0.60	0.77
**GT25**	0.00	0.00	0.00	0.00	0.00	0.00	0.00	0.00	0.00	0.00	0.00	0.00	0.00
**GT2**	3.26	6.30	3.76	4.09	3.94	4.61	3.89	4.57	4.42	4.06	3.10	3.42	4.92
**GT51**	0.75	0.57	0.77	0.94	0.77	1.00	0.80	0.90	0.83	0.87	0.73	0.64	0.86
**GH107**	0.00	0.00	0.00	0.00	0.00	0.00	0.00	0.00	0.00	0.00	0.00	0.00	0.00
**GH7**	0.00	0.00	0.00	0.00	0.00	0.00	0.00	0.00	0.00	0.00	0.00	0.00	0.00
**GT7**	0.00	0.00	0.00	0.00	0.00	0.00	0.00	0.00	0.00	0.00	0.00	0.00	0.00
**GT36**	0.00	0.00	0.00	0.00	0.00	0.00	0.00	0.00	0.00	0.00	0.00	0.00	0.00
**PL7**	0.00	0.00	0.00	0.00	0.01	0.00	0.00	0.00	0.00	0.00	0.00	0.00	0.00
**GH110**	0.07	0.26	0.08	0.06	0.04	0.11	0.06	0.11	0.10	0.05	0.05	0.11	0.10
**GT21**	0.00	0.00	0.00	0.00	0.00	0.00	0.00	0.00	0.00	0.00	0.00	0.00	0.00
**GH62**	0.00	0.00	0.00	0.00	0.00	0.00	0.00	0.00	0.00	0.00	0.00	0.00	0.00
**GH125**	0.18	0.26	0.11	0.15	0.09	0.13	0.09	0.00	0.09	0.13	0.14	0.17	0.22
**GH117**	0.03	0.31	0.08	0.06	0.03	0.06	0.06	0.05	0.05	0.04	0.06	0.11	0.09
**GH68**	0.00	0.00	0.00	0.00	0.00	0.00	0.00	0.00	0.00	0.00	0.00	0.00	0.00
**GH85**	0.01	0.00	0.00	0.07	0.03	0.00	0.00	0.00	0.00	0.02	0.00	0.01	0.01
**GH92**	1.22	2.12	1.15	1.33	0.70	1.42	0.94	0.88	1.33	1.40	1.18	1.31	2.00
**GH40**	0.00	0.00	0.00	0.00	0.00	0.00	0.00	0.00	0.00	0.00	0.00	0.00	0.00
**GT49**	0.00	0.00	0.00	0.00	0.00	0.00	0.00	0.00	0.00	0.00	0.00	0.00	0.00
**GH112**	0.13	0.10	0.20	0.30	0.19	0.17	0.11	0.19	0.15	0.17	0.22	0.08	0.14
**GT67**	0.00	0.00	0.00	0.00	0.00	0.00	0.00	0.00	0.00	0.00	0.00	0.00	0.00
**CE8**	0.16	0.21	0.16	0.07	0.12	0.28	0.21	0.41	0.20	0.23	0.09	0.28	0.28
**GT86**	0.00	0.00	0.00	0.00	0.00	0.00	0.00	0.00	0.00	0.00	0.00	0.00	0.00
**GH101**	0.00	0.00	0.00	0.07	0.00	0.00	0.00	0.00	0.00	0.00	0.00	0.00	0.01
**GH81**	0.00	0.00	0.00	0.00	0.00	0.00	0.00	0.00	0.00	0.00	0.00	0.00	0.00
**GT93**	0.00	0.00	0.00	0.00	0.00	0.00	0.00	0.00	0.00	0.00	0.00	0.00	0.00
**GT63**	0.00	0.00	0.00	0.00	0.00	0.00	0.00	0.00	0.00	0.00	0.00	0.00	0.00
**CE11**	0.18	0.15	0.19	0.17	0.19	0.25	0.23	0.25	0.22	0.17	0.24	0.20	0.22
**GT19**	0.09	0.10	0.13	0.12	0.09	0.11	0.09	0.08	0.13	0.10	0.15	0.12	0.12
**GT33**	0.00	0.00	0.00	0.00	0.00	0.00	0.00	0.00	0.00	0.00	0.00	0.00	0.00
**GH21**	0.00	0.00	0.00	0.00	0.00	0.00	0.00	0.00	0.00	0.00	0.00	0.00	0.00
**GT68**	0.00	0.00	0.00	0.00	0.00	0.00	0.00	0.00	0.00	0.00	0.00	0.00	0.00
**GH89**	0.12	0.21	0.17	0.11	0.12	0.08	0.14	0.08	0.19	0.23	0.17	0.20	0.30
**GT82**	0.00	0.00	0.00	0.00	0.00	0.00	0.00	0.00	0.00	0.00	0.00	0.00	0.00
**GH30**	0.22	0.41	0.13	0.24	0.14	0.11	0.16	0.25	0.22	0.15	0.13	0.24	0.27
**GH20**	0.58	2.07	0.87	0.81	0.39	0.68	0.62	0.66	0.92	0.84	0.96	0.90	1.18
**GH52**	0.00	0.00	0.00	0.00	0.00	0.00	0.00	0.00	0.00	0.00	0.00	0.00	0.00
**GT84**	0.01	0.00	0.03	0.12	0.04	0.04	0.00	0.03	0.02	0.01	0.00	0.03	0.01
**GT15**	0.00	0.00	0.00	0.00	0.00	0.00	0.00	0.00	0.00	0.00	0.00	0.00	0.00
**GH93**	0.00	0.00	0.00	0.00	0.00	0.00	0.00	0.00	0.00	0.00	0.00	0.00	0.00
**PL16**	0.00	0.00	0.00	0.00	0.00	0.00	0.00	0.00	0.00	0.00	0.00	0.00	0.00
**GH26**	0.22	0.41	0.12	0.09	0.12	0.15	0.17	0.19	0.09	0.20	0.04	0.10	0.18

CAZY_group	DA-AD-27	DA-AD-28	DA-AD-29	DA-AD-3	DA-AD-30	DA-AD-31	DA-AD-32	DA-AD-33	DA-AD-34	DA-AD-35	DA-AD-36	DA-AD-37	DA-AD-38
GT75	0.00	0.00	0.00	0.00	0.00	0.00	0.00	0.00	0.00	0.00	0.00	0.00	0.00
GH53	0.09	0.21	0.08	0.08	0.15	0.17	0.19	0.22	0.20	0.21	0.17	0.03	0.14
GT88	0.00	0.00	0.00	0.00	0.00	0.00	0.00	0.00	0.00	0.00	0.00	0.00	0.00
GT24	0.00	0.00	0.00	0.00	0.00	0.00	0.00	0.00	0.00	0.00	0.00	0.00	0.00
GT81	0.00	0.00	0.00	0.00	0.00	0.00	0.00	0.00	0.00	0.00	0.00	0.00	0.00
GH4	0.10	0.10	0.12	0.29	0.08	0.09	0.06	0.03	0.08	0.11	0.09	0.05	0.09
GT78	0.00	0.00	0.00	0.00	0.00	0.00	0.00	0.00	0.00	0.00	0.00	0.00	0.00
GH8	0.01	0.15	0.08	0.09	0.08	0.02	0.08	0.05	0.10	0.07	0.04	0.05	0.11
GH70	0.00	0.00	0.01	0.00	0.00	0.00	0.00	0.00	0.00	0.00	0.00	0.00	0.00
GH122	0.00	0.00	0.00	0.00	0.00	0.00	0.00	0.00	0.00	0.00	0.00	0.00	0.00
GH74	0.03	0.10	0.02	0.00	0.05	0.02	0.04	0.00	0.01	0.02	0.01	0.02	0.05
GT41	0.00	0.00	0.01	0.01	0.01	0.00	0.00	0.00	0.01	0.00	0.00	0.01	0.01
PL1	0.13	0.26	0.20	0.09	0.12	0.26	0.21	0.30	0.26	0.35	0.17	0.32	0.34
GH15	0.03	0.15	0.05	0.02	0.04	0.02	0.05	0.08	0.06	0.03	0.05	0.03	0.03
GH32	0.31	0.52	0.36	0.27	0.23	0.34	0.38	0.57	0.30	0.35	0.37	0.29	0.36
GH44	0.00	0.00	0.00	0.00	0.00	0.00	0.00	0.00	0.01	0.00	0.00	0.00	0.00
GT6	0.00	0.00	0.00	0.00	0.00	0.00	0.01	0.00	0.00	0.00	0.00	0.00	0.00
GH13	2.68	4.86	2.74	3.05	3.06	3.78	3.14	4.05	3.25	3.37	2.87	2.46	3.09
GT80	0.03	0.00	0.01	0.02	0.00	0.02	0.00	0.00	0.00	0.01	0.00	0.00	0.01
GH71	0.00	0.00	0.00	0.00	0.00	0.00	0.00	0.00	0.00	0.00	0.00	0.00	0.00
GH63	0.09	0.31	0.11	0.11	0.04	0.11	0.07	0.11	0.11	0.08	0.10	0.03	0.12
PL21	0.00	0.00	0.00	0.00	0.01	0.09	0.00	0.00	0.00	0.00	0.01	0.02	0.05
GH65	0.06	0.41	0.08	0.15	0.03	0.11	0.07	0.08	0.09	0.11	0.19	0.11	0.23
CE4	0.57	0.83	0.52	0.64	0.69	0.77	0.73	0.79	0.59	0.57	0.53	0.50	0.59
CE14	0.00	0.00	0.03	0.01	0.00	0.08	0.02	0.00	0.01	0.01	0.03	0.01	0.03
GH115	0.13	0.21	0.17	0.14	0.15	0.09	0.19	0.25	0.25	0.31	0.18	0.19	0.25
GH104	0.00	0.00	0.01	0.00	0.00	0.00	0.00	0.00	0.00	0.00	0.00	0.00	0.00
GH111	0.00	0.00	0.00	0.00	0.00	0.00	0.00	0.00	0.00	0.00	0.00	0.00	0.00
GH95	0.34	0.62	0.41	0.31	0.42	0.43	0.35	0.38	0.42	0.46	0.53	0.44	0.71
GT70	0.00	0.00	0.00	0.00	0.00	0.00	0.00	0.00	0.00	0.00	0.00	0.00	0.00
CE10	0.12	0.36	0.17	0.10	0.07	0.08	0.09	0.11	0.14	0.15	0.10	0.09	0.18
GH10	0.03	0.15	0.10	0.10	0.12	0.06	0.12	0.19	0.17	0.16	0.08	0.09	0.22
GH130	0.22	0.72	0.26	0.30	0.28	0.19	0.26	0.25	0.29	0.37	0.24	0.22	0.50
PL20	0.00	0.00	0.00	0.00	0.00	0.00	0.00	0.00	0.00	0.00	0.00	0.00	0.00
GH69	0.00	0.00	0.00	0.00	0.00	0.00	0.00	0.00	0.00	0.00	0.00	0.00	0.00
PL2	0.00	0.00	0.00	0.00	0.00	0.00	0.00	0.00	0.00	0.00	0.00	0.00	0.00
GH49	0.00	0.00	0.00	0.00	0.00	0.00	0.00	0.00	0.00	0.00	0.00	0.00	0.00
GH25	0.46	0.57	0.27	0.29	0.42	0.60	0.39	0.63	0.34	0.32	0.29	0.28	0.29
GH59	0.03	0.05	0.02	0.01	0.04	0.04	0.04	0.00	0.04	0.07	0.00	0.02	0.04
GH35	0.13	0.31	0.19	0.19	0.18	0.09	0.25	0.22	0.34	0.31	0.28	0.28	0.43
GT39	0.00	0.00	0.01	0.03	0.00	0.00	0.01	0.00	0.00	0.00	0.00	0.00	0.01
GT57	0.00	0.00	0.00	0.00	0.00	0.00	0.00	0.00	0.00	0.00	0.00	0.00	0.00
GH129	0.00	0.00	0.01	0.02	0.00	0.00	0.00	0.00	0.00	0.01	0.01	0.03	0.01
GT22	0.00	0.00	0.00	0.00	0.00	0.00	0.00	0.00	0.01	0.00	0.00	0.00	0.00
PL12	0.06	0.10	0.04	0.04	0.07	0.08	0.05	0.03	0.04	0.05	0.08	0.11	0.15
PL18	0.00	0.00	0.00	0.00	0.00	0.00	0.00	0.00	0.00	0.00	0.00	0.00	0.00
GT92	0.00	0.00	0.00	0.00	0.00	0.00	0.00	0.00	0.00	0.00	0.00	0.00	0.00
GT44	0.00	0.00	0.00	0.00	0.00	0.00	0.00	0.00	0.00	0.00	0.00	0.00	0.00
GT29	0.00	0.00	0.00	0.00	0.00	0.00	0.00	0.00	0.00	0.00	0.00	0.00	0.00
GT13	0.00	0.00	0.00	0.00	0.00	0.00	0.00	0.00	0.00	0.00	0.00	0.00	0.00

CAZY_group	DA-AD-27	DA-AD-28	DA-AD-29	DA-AD-3	DA-AD-30	DA-AD-31	DA-AD-32	DA-AD-33	DA-AD-34	DA-AD-35	DA-AD-36	DA-AD-37	DA-AD-38
**GT52**	0.00	0.00	0.00	0.00	0.00	0.00	0.00	0.00	0.00	0.00	0.00	0.00	0.00
**GT64**	0.00	0.00	0.00	0.00	0.00	0.00	0.00	0.00	0.00	0.00	0.00	0.00	0.00
**GT85**	0.00	0.00	0.00	0.00	0.00	0.00	0.00	0.00	0.00	0.00	0.00	0.00	0.00
**GT16**	0.00	0.00	0.00	0.00	0.00	0.00	0.00	0.00	0.00	0.00	0.00	0.00	0.00
**GH56**	0.00	0.00	0.00	0.00	0.00	0.00	0.00	0.00	0.00	0.00	0.00	0.00	0.00
**GT60**	0.00	0.00	0.00	0.00	0.00	0.00	0.00	0.00	0.00	0.00	0.00	0.00	0.00
**GH41**	0.00	0.00	0.00	0.00	0.00	0.00	0.00	0.00	0.00	0.00	0.00	0.00	0.00
**CE7**	0.10	0.31	0.10	0.05	0.04	0.08	0.04	0.08	0.08	0.05	0.05	0.10	0.19
**GH39**	0.00	0.05	0.05	0.01	0.09	0.02	0.09	0.11	0.04	0.04	0.03	0.01	0.08
**GT30**	0.25	0.31	0.25	0.27	0.12	0.23	0.20	0.14	0.17	0.24	0.20	0.21	0.31
**PL15**	0.01	0.21	0.05	0.00	0.05	0.00	0.03	0.00	0.06	0.10	0.15	0.05	0.13
**GH102**	0.00	0.00	0.01	0.09	0.01	0.06	0.02	0.03	0.02	0.00	0.01	0.02	0.04

CAZY_group	DA-AD-39	DA-AD-4	DA-AD-40	DA-AD-41	DA-AD-42	DA-AD-43	DA-AD-44	DA-AD-45	DA-AD-46	DA-AD-47	DA-AD-48	DA-AD-49	DA-AD-5
**GH29**	0.61	0.36	0.06	0.50	0.53	0.51	0.61	0.97	0.91	0.66	0.65	0.60	0.31
**PL4**	0.00	0.00	0.00	0.00	0.00	0.00	0.01	0.00	0.00	0.00	0.00	0.00	0.00
**GH23**	0.88	1.57	0.01	0.96	0.85	0.74	1.00	1.18	1.23	1.07	1.06	0.99	0.94
**CE6**	0.02	0.03	0.00	0.01	0.03	0.02	0.07	0.00	0.00	0.00	0.06	0.02	0.10
**GH72**	0.16	0.14	0.00	0.09	0.08	0.10	0.17	0.10	0.12	0.17	0.11	0.15	0.26
**GH114**	0.00	0.00	0.00	0.00	0.00	0.00	0.00	0.00	0.00	0.00	0.00	0.00	0.00
**GH78**	0.50	0.54	0.07	0.52	0.65	0.56	0.66	0.63	1.21	0.75	0.56	0.56	0.68
**PL19**	0.00	0.00	0.00	0.00	0.00	0.00	0.00	0.00	0.00	0.00	0.00	0.00	0.00
**GT27**	0.00	0.00	0.00	0.00	0.00	0.00	0.00	0.00	0.00	0.00	0.00	0.00	0.00
**GH5**	0.35	0.42	0.00	0.42	0.37	0.53	0.74	0.65	0.58	0.70	0.47	0.41	0.63
**GT40**	0.00	0.00	0.00	0.00	0.00	0.00	0.00	0.00	0.00	0.00	0.00	0.00	0.00
**CE13**	0.00	0.00	0.00	0.00	0.00	0.00	0.00	0.00	0.00	0.00	0.00	0.00	0.00
**GT37**	0.00	0.00	0.00	0.00	0.00	0.00	0.00	0.00	0.00	0.00	0.00	0.00	0.00
**GH33**	0.14	0.09	0.01	0.14	0.24	0.12	0.15	0.23	0.30	0.24	0.22	0.18	0.00
**GT65**	0.00	0.00	0.00	0.00	0.00	0.00	0.00	0.00	0.00	0.00	0.00	0.00	0.00
**GH86**	0.02	0.00	0.00	0.00	0.00	0.00	0.00	0.00	0.00	0.00	0.00	0.01	0.00
**GH123**	0.07	0.07	0.00	0.10	0.09	0.09	0.15	0.15	0.12	0.09	0.06	0.05	0.00
**GH96**	0.00	0.00	0.00	0.00	0.00	0.00	0.00	0.00	0.00	0.00	0.00	0.00	0.00
**GH14**	0.00	0.00	0.00	0.00	0.00	0.00	0.00	0.00	0.00	0.00	0.00	0.00	0.00
**CE3**	0.00	0.00	0.00	0.00	0.00	0.00	0.00	0.00	0.00	0.00	0.00	0.00	0.00
**PL10**	0.07	0.05	0.01	0.03	0.10	0.06	0.10	0.09	0.16	0.22	0.09	0.08	0.10
**GT48**	0.00	0.00	0.00	0.00	0.00	0.00	0.00	0.00	0.00	0.00	0.00	0.00	0.00
**PL6**	0.00	0.00	0.00	0.01	0.00	0.00	0.01	0.00	0.00	0.00	0.00	0.00	0.00
**GT83**	0.04	0.04	0.01	0.08	0.06	0.05	0.09	0.03	0.19	0.05	0.09	0.10	0.05
**GH126**	0.00	0.00	0.00	0.00	0.00	0.00	0.00	0.00	0.00	0.00	0.00	0.00	0.00
**GH9**	0.26	0.16	0.06	0.15	0.16	0.18	0.33	0.21	0.07	0.55	0.52	0.25	0.78
**GH75**	0.00	0.00	0.00	0.00	0.00	0.00	0.00	0.00	0.00	0.00	0.00	0.00	0.00
**GT71**	0.00	0.00	0.00	0.00	0.00	0.00	0.00	0.00	0.00	0.00	0.00	0.00	0.00
**GH46**	0.00	0.01	0.00	0.00	0.00	0.01	0.00	0.00	0.00	0.00	0.00	0.00	0.00
**GT61**	0.00	0.00	0.00	0.00	0.00	0.00	0.00	0.00	0.00	0.00	0.00	0.00	0.00
**GT3**	0.19	0.15	0.02	0.17	0.15	0.16	0.07	0.28	0.28	0.17	0.13	0.20	0.31
**PL3**	0.00	0.00	0.00	0.00	0.00	0.00	0.00	0.00	0.00	0.00	0.00	0.00	0.00
**GH108**	0.02	0.01	0.00	0.02	0.06	0.03	0.01	0.01	0.05	0.02	0.06	0.03	0.00
**GT56**	0.00	0.00	0.00	0.00	0.00	0.00	0.00	0.00	0.00	0.00	0.00	0.00	0.00
**PL11**	0.12	0.08	0.00	0.08	0.10	0.13	0.19	0.23	0.19	0.20	0.06	0.08	0.00
**GH45**	0.00	0.00	0.00	0.00	0.00	0.00	0.00	0.00	0.00	0.00	0.00	0.00	0.00
**GT34**	0.00	0.00	0.00	0.00	0.00	0.00	0.00	0.00	0.00	0.00	0.00	0.00	0.00
**PL14**	0.00	0.00	0.00	0.00	0.00	0.00	0.00	0.00	0.00	0.00	0.00	0.00	0.00
**GH19**	0.00	0.00	0.00	0.01	0.01	0.01	0.00	0.00	0.02	0.00	0.00	0.00	0.00
**GH118**	0.00	0.00	0.00	0.00	0.00	0.00	0.00	0.00	0.00	0.00	0.00	0.00	0.00
**GH82**	0.00	0.00	0.00	0.00	0.00	0.00	0.00	0.00	0.00	0.00	0.00	0.00	0.00
**GH76**	0.13	0.02	0.02	0.10	0.15	0.15	0.22	0.10	0.09	0.12	0.11	0.08	0.05
**GT4**	1.62	1.99	0.08	1.52	1.74	1.80	2.16	1.73	1.90	2.32	1.55	1.75	1.46
**GT87**	0.00	0.00	0.00	0.00	0.00	0.00	0.00	0.00	0.00	0.02	0.02	0.01	0.00
**GH24**	0.06	0.12	0.01	0.10	0.08	0.09	0.05	0.09	0.14	0.12	0.06	0.10	0.00
**GH90**	0.00	0.00	0.00	0.00	0.00	0.00	0.00	0.00	0.00	0.00	0.00	0.00	0.00
**GH57**	0.18	0.20	0.01	0.11	0.13	0.05	0.07	0.15	0.35	0.17	0.15	0.16	0.21
**GH36**	0.66	0.71	0.01	0.61	0.54	0.60	0.82	0.82	0.72	0.68	0.93	0.67	0.84
**GT46**	0.00	0.00	0.00	0.00	0.00	0.00	0.00	0.00	0.00	0.00	0.00	0.00	0.00
**GH66**	0.02	0.01	0.00	0.01	0.04	0.01	0.02	0.06	0.05	0.03	0.04	0.03	0.00

CAZY_group	DA-AD-39	DA-AD-4	DA-AD-40	DA-AD-41	DA-AD-42	DA-AD-43	DA-AD-44	DA-AD-45	DA-AD-46	DA-AD-47	DA-AD-48	DA-AD-49	DA-AD-5
**GH48**	0.00	0.00	0.00	0.00	0.01	0.00	0.01	0.00	0.00	0.00	0.00	0.00	0.00
**GT53**	0.00	0.00	0.00	0.00	0.00	0.00	0.00	0.00	0.00	0.00	0.00	0.00	0.00
**GH60**	0.00	0.00	0.00	0.00	0.00	0.00	0.00	0.00	0.00	0.00	0.00	0.00	0.00
**GT31**	0.00	0.00	0.00	0.00	0.00	0.00	0.00	0.00	0.00	0.00	0.00	0.00	0.00
**GH105**	0.39	0.24	0.00	0.28	0.39	0.53	0.58	0.53	0.49	0.63	0.45	0.31	0.37
**GT23**	0.00	0.00	0.00	0.01	0.01	0.00	0.02	0.01	0.02	0.00	0.00	0.00	0.00
**GH11**	0.00	0.00	0.00	0.00	0.00	0.00	0.01	0.00	0.00	0.03	0.00	0.00	0.00
**GT38**	0.00	0.00	0.00	0.00	0.00	0.00	0.00	0.00	0.00	0.00	0.00	0.00	0.00
**GT43**	0.00	0.00	0.00	0.00	0.00	0.00	0.00	0.00	0.00	0.00	0.00	0.00	0.00
**GH99**	0.01	0.00	0.00	0.00	0.01	0.00	0.01	0.00	0.00	0.00	0.02	0.00	0.00
**GH38**	0.14	0.16	0.02	0.19	0.20	0.13	0.25	0.21	0.26	0.19	0.15	0.17	0.05
**GH109**	0.27	0.11	0.06	0.22	0.21	0.21	0.15	0.30	0.33	0.31	0.30	0.18	0.10
**GH2**	3.15	2.89	0.26	2.79	3.02	3.24	3.76	4.68	4.04	4.24	3.84	3.05	2.82
**GT28**	0.37	0.48	0.03	0.43	0.31	0.41	0.49	0.42	0.56	0.46	0.39	0.40	0.47
**GH42**	0.12	0.18	0.01	0.31	0.26	0.21	0.29	0.09	0.19	0.17	0.22	0.20	0.05
**GT58**	0.00	0.00	0.00	0.00	0.01	0.00	0.00	0.00	0.00	0.00	0.00	0.00	0.00
**GH61**	0.00	0.00	0.00	0.00	0.00	0.00	0.00	0.00	0.00	0.00	0.00	0.00	0.00
**GH80**	0.00	0.00	0.00	0.00	0.00	0.00	0.00	0.00	0.00	0.00	0.00	0.00	0.00
**GH98**	0.00	0.00	0.00	0.00	0.00	0.00	0.02	0.00	0.00	0.10	0.02	0.01	0.00
**GH6**	0.00	0.00	0.00	0.00	0.00	0.00	0.00	0.00	0.00	0.00	0.00	0.00	0.00
**GT1**	0.04	0.05	0.00	0.05	0.06	0.06	0.05	0.09	0.00	0.05	0.06	0.02	0.00
**GH28**	0.55	0.52	0.04	0.48	0.67	0.75	0.88	1.07	1.07	0.95	0.71	0.65	0.47
**GT42**	0.00	0.00	0.00	0.00	0.00	0.00	0.00	0.00	0.00	0.00	0.00	0.00	0.00
**GH128**	0.00	0.00	0.00	0.00	0.00	0.00	0.00	0.00	0.02	0.00	0.00	0.00	0.00
**CE12**	0.06	0.07	0.00	0.08	0.10	0.13	0.21	0.18	0.16	0.07	0.24	0.06	0.31
**CE16**	0.00	0.00	0.00	0.00	0.00	0.00	0.00	0.00	0.00	0.00	0.00	0.00	0.00
**GT89**	0.00	0.00	0.00	0.00	0.00	0.00	0.00	0.00	0.00	0.00	0.00	0.00	0.00
**GT11**	0.02	0.00	0.00	0.03	0.04	0.04	0.08	0.08	0.09	0.07	0.13	0.07	0.00
**GT77**	0.00	0.00	0.00	0.00	0.00	0.00	0.00	0.00	0.00	0.00	0.00	0.00	0.00
**GT55**	0.00	0.00	0.00	0.00	0.00	0.00	0.00	0.00	0.00	0.00	0.00	0.00	0.00
**GT18**	0.00	0.00	0.00	0.00	0.00	0.00	0.00	0.00	0.00	0.00	0.00	0.00	0.00
**GH87**	0.00	0.00	0.00	0.00	0.00	0.00	0.00	0.00	0.00	0.00	0.00	0.00	0.00
**PL5**	0.00	0.00	0.00	0.00	0.00	0.00	0.00	0.00	0.00	0.00	0.00	0.00	0.00
**GH22**	0.00	0.00	0.00	0.00	0.00	0.00	0.00	0.00	0.00	0.00	0.00	0.00	0.00
**CE2**	0.05	0.05	0.00	0.06	0.08	0.05	0.05	0.14	0.09	0.17	0.04	0.08	0.10
**GH120**	0.05	0.03	0.01	0.08	0.09	0.05	0.10	0.01	0.05	0.05	0.06	0.08	0.00
**GH73**	0.46	0.49	0.10	0.41	0.36	0.42	0.57	0.54	0.88	0.48	0.52	0.50	0.52
**GT17**	0.00	0.00	0.00	0.00	0.00	0.00	0.00	0.00	0.00	0.00	0.00	0.00	0.00
**GH124**	0.00	0.00	0.00	0.00	0.00	0.00	0.00	0.00	0.00	0.00	0.00	0.00	0.00
**GH54**	0.00	0.00	0.00	0.00	0.00	0.02	0.00	0.00	0.00	0.00	0.00	0.00	0.00
**GH100**	0.00	0.00	0.00	0.00	0.00	0.00	0.00	0.00	0.00	0.00	0.00	0.00	0.00
**GH113**	0.02	0.05	0.00	0.03	0.02	0.03	0.04	0.06	0.00	0.00	0.09	0.02	0.05
**GT5**	0.41	0.69	0.02	0.49	0.39	0.44	0.53	0.42	0.42	0.44	0.47	0.45	0.52
**GH94**	0.33	0.48	0.01	0.34	0.30	0.56	0.68	0.38	0.16	0.37	0.69	0.37	0.68
**GT66**	0.00	0.01	0.00	0.00	0.00	0.00	0.00	0.00	0.00	0.00	0.00	0.00	0.00
**GT94**	0.00	0.00	0.00	0.00	0.00	0.00	0.00	0.00	0.00	0.00	0.00	0.00	0.00
**GH58**	0.00	0.00	0.00	0.00	0.00	0.00	0.00	0.00	0.00	0.00	0.00	0.00	0.00
**GH1**	0.21	0.53	0.00	0.30	0.36	0.44	0.47	0.35	0.30	0.10	0.39	0.32	0.42
**PL22**	0.00	0.00	0.00	0.00	0.00	0.00	0.00	0.00	0.00	0.00	0.00	0.00	0.00

CAZY_group	DA-AD-39	DA-AD-4	DA-AD-40	DA-AD-41	DA-AD-42	DA-AD-43	DA-AD-44	DA-AD-45	DA-AD-46	DA-AD-47	DA-AD-48	DA-AD-49	DA-AD-5
GT76	0.00	0.00	0.00	0.00	0.00	0.00	0.00	0.00	0.00	0.00	0.00	0.00	0.00
GT32	0.08	0.08	0.00	0.08	0.13	0.11	0.08	0.10	0.09	0.12	0.15	0.09	0.00
GT47	0.03	0.09	0.00	0.03	0.10	0.02	0.04	0.03	0.02	0.12	0.00	0.02	0.00
GH84	0.08	0.09	0.00	0.08	0.08	0.03	0.10	0.18	0.14	0.10	0.11	0.09	0.05
GH83	0.00	0.00	0.00	0.00	0.00	0.00	0.00	0.00	0.00	0.00	0.00	0.00	0.00
GT59	0.00	0.00	0.00	0.00	0.00	0.00	0.00	0.00	0.00	0.00	0.00	0.00	0.00
GH34	0.00	0.00	0.00	0.00	0.00	0.00	0.00	0.00	0.00	0.00	0.00	0.00	0.00
GT14	0.02	0.01	0.00	0.02	0.03	0.02	0.03	0.05	0.00	0.02	0.02	0.01	0.00
GH47	0.00	0.00	0.00	0.00	0.03	0.00	0.00	0.00	0.00	0.00	0.00	0.00	0.00
GH97	0.66	0.48	0.08	0.54	0.63	0.60	0.89	1.10	1.28	0.97	0.78	0.63	0.42
GH50	0.01	0.00	0.00	0.02	0.03	0.03	0.02	0.00	0.00	0.02	0.00	0.01	0.00
GT26	0.20	0.20	0.00	0.18	0.20	0.21	0.25	0.20	0.23	0.24	0.17	0.23	0.21
GH18	0.55	0.40	0.03	0.53	0.58	0.32	0.74	0.50	0.51	0.51	0.60	0.36	0.37
GH37	0.01	0.00	0.00	0.00	0.00	0.00	0.00	0.00	0.00	0.00	0.00	0.00	0.00
PL13	0.06	0.00	0.00	0.00	0.05	0.02	0.04	0.00	0.00	0.05	0.04	0.01	0.00
GH27	0.26	0.17	0.00	0.12	0.25	0.12	0.27	0.19	0.19	0.24	0.15	0.21	0.26
GT54	0.00	0.00	0.00	0.00	0.00	0.00	0.00	0.00	0.00	0.00	0.00	0.00	0.00
GT91	0.00	0.00	0.00	0.00	0.00	0.00	0.00	0.00	0.00	0.00	0.00	0.00	0.00
GT72	0.00	0.00	0.00	0.00	0.00	0.00	0.00	0.00	0.00	0.00	0.00	0.00	0.00
GH67	0.04	0.05	0.00	0.08	0.06	0.05	0.05	0.09	0.26	0.14	0.04	0.10	0.05
GH12	0.00	0.00	0.00	0.00	0.00	0.00	0.00	0.00	0.00	0.00	0.00	0.00	0.00
GH91	0.01	0.00	0.00	0.02	0.01	0.04	0.02	0.00	0.02	0.00	0.06	0.02	0.00
GT69	0.00	0.00	0.00	0.00	0.00	0.00	0.00	0.00	0.00	0.00	0.00	0.00	0.00
GH106	0.20	0.12	0.03	0.14	0.19	0.27	0.23	0.29	0.30	0.26	0.22	0.25	0.57
GT62	0.00	0.00	0.00	0.00	0.00	0.00	0.00	0.00	0.00	0.00	0.00	0.00	0.00
GH17	0.00	0.00	0.00	0.00	0.00	0.00	0.00	0.00	0.00	0.00	0.00	0.00	0.00
GH88	0.33	0.09	0.01	0.14	0.22	0.30	0.27	0.29	0.21	0.37	0.37	0.21	0.05
CE5	0.00	0.00	0.00	0.00	0.00	0.00	0.00	0.00	0.00	0.00	0.00	0.00	0.00
GT8	0.09	0.09	0.00	0.10	0.04	0.05	0.08	0.06	0.14	0.07	0.04	0.14	0.16
GT50	0.00	0.00	0.00	0.00	0.00	0.00	0.00	0.00	0.00	0.00	0.00	0.00	0.00
GT20	0.07	0.00	0.01	0.08	0.05	0.09	0.07	0.05	0.09	0.09	0.09	0.08	0.00
PL9	0.04	0.03	0.00	0.04	0.02	0.06	0.24	0.08	0.05	0.07	0.06	0.02	0.16
GH55	0.04	0.01	0.00	0.01	0.02	0.02	0.02	0.05	0.00	0.03	0.04	0.02	0.00
GH64	0.00	0.00	0.00	0.00	0.00	0.00	0.00	0.00	0.02	0.00	0.00	0.00	0.00
GH127	0.24	0.35	0.02	0.24	0.27	0.29	0.30	0.33	0.56	0.26	0.41	0.35	0.21
GH103	0.00	0.01	0.00	0.00	0.00	0.00	0.00	0.00	0.00	0.00	0.00	0.00	0.00
GH3	1.96	1.85	0.21	2.22	2.19	2.35	2.91	3.01	2.28	3.05	2.81	1.97	2.09
GT45	0.00	0.00	0.00	0.00	0.00	0.00	0.00	0.00	0.00	0.00	0.00	0.00	0.00
GT74	0.00	0.00	0.00	0.00	0.00	0.00	0.00	0.00	0.00	0.00	0.00	0.00	0.00
GT90	0.00	0.00	0.00	0.00	0.00	0.00	0.00	0.00	0.00	0.00	0.00	0.00	0.00
GH116	0.11	0.03	0.00	0.05	0.08	0.05	0.07	0.08	0.00	0.02	0.09	0.06	0.00
GH31	0.78	0.66	0.06	0.85	0.90	0.89	1.19	1.22	0.84	1.02	0.93	0.76	0.84
GT12	0.00	0.00	0.00	0.00	0.00	0.00	0.00	0.00	0.00	0.00	0.00	0.00	0.00
CE1	0.28	0.30	0.01	0.26	0.34	0.32	0.40	0.74	0.74	0.41	0.37	0.30	0.47
GH121	0.00	0.00	0.00	0.00	0.00	0.00	0.00	0.00	0.00	0.00	0.00	0.00	0.00
GH79	0.02	0.00	0.00	0.00	0.00	0.01	0.00	0.03	0.00	0.00	0.00	0.00	0.00
GT73	0.00	0.00	0.00	0.00	0.00	0.00	0.00	0.00	0.00	0.00	0.00	0.00	0.00
GH77	0.50	0.80	0.07	0.56	0.52	0.57	0.75	0.71	0.74	0.55	0.60	0.59	0.68
PL17	0.01	0.01	0.00	0.01	0.00	0.01	0.00	0.00	0.00	0.00	0.00	0.00	0.00
PL8	0.37	0.02	0.01	0.08	0.22	0.19	0.15	0.26	0.00	0.29	0.22	0.13	0.00

CAZY_group	DA-AD-39	DA-AD-4	DA-AD-40	DA-AD-41	DA-AD-42	DA-AD-43	DA-AD-44	DA-AD-45	DA-AD-46	DA-AD-47	DA-AD-48	DA-AD-49	DA-AD-5
**CE9**	0.21	0.26	0.00	0.19	0.22	0.31	0.38	0.40	0.30	0.15	0.26	0.22	0.21
**CE15**	0.02	0.01	0.00	0.00	0.01	0.00	0.00	0.00	0.00	0.00	0.00	0.00	0.00
**GT79**	0.00	0.00	0.00	0.00	0.00	0.00	0.00	0.00	0.00	0.00	0.00	0.00	0.00
**GT10**	0.01	0.00	0.00	0.00	0.00	0.00	0.03	0.00	0.00	0.00	0.06	0.03	0.00
**GH119**	0.00	0.00	0.00	0.00	0.00	0.00	0.00	0.00	0.00	0.00	0.00	0.00	0.00
**GH16**	0.23	0.13	0.01	0.13	0.16	0.13	0.22	0.28	0.12	0.20	0.35	0.18	0.05
**GH43**	1.53	1.16	0.24	1.31	1.58	2.06	2.31	2.71	3.37	2.37	2.01	2.08	0.99
**GH51**	0.38	0.50	0.08	0.35	0.41	0.46	0.60	0.53	0.79	0.53	0.80	0.59	0.47
**GT35**	0.73	1.31	0.16	0.88	0.87	0.75	1.08	0.68	0.98	0.87	0.89	0.96	1.36
**GT25**	0.00	0.00	0.00	0.00	0.00	0.00	0.00	0.00	0.00	0.00	0.00	0.00	0.00
**GT2**	3.52	5.59	0.29	3.25	3.43	4.19	5.12	4.67	4.37	4.04	4.34	3.67	5.27
**GT51**	0.72	0.83	0.10	0.62	0.65	0.81	1.06	1.17	1.32	0.75	1.01	0.74	1.46
**GH107**	0.00	0.00	0.00	0.00	0.00	0.00	0.00	0.00	0.00	0.00	0.00	0.00	0.00
**GH7**	0.00	0.00	0.00	0.00	0.00	0.00	0.00	0.00	0.00	0.00	0.00	0.00	0.00
**GT7**	0.00	0.00	0.00	0.00	0.00	0.00	0.00	0.00	0.00	0.00	0.00	0.00	0.00
**GT36**	0.00	0.00	0.00	0.00	0.00	0.00	0.00	0.00	0.00	0.00	0.00	0.00	0.00
**PL7**	0.00	0.00	0.00	0.00	0.00	0.00	0.00	0.00	0.00	0.00	0.00	0.00	0.00
**GH110**	0.08	0.03	0.01	0.04	0.07	0.04	0.12	0.09	0.07	0.09	0.17	0.08	0.00
**GT21**	0.00	0.00	0.00	0.00	0.00	0.00	0.00	0.00	0.00	0.00	0.00	0.00	0.00
**GH62**	0.00	0.00	0.00	0.00	0.00	0.00	0.00	0.00	0.00	0.00	0.00	0.00	0.00
**GH125**	0.20	0.16	0.01	0.15	0.10	0.12	0.19	0.23	0.14	0.19	0.11	0.12	0.05
**GH117**	0.08	0.03	0.01	0.04	0.03	0.04	0.07	0.08	0.05	0.20	0.06	0.07	0.00
**GH68**	0.00	0.00	0.00	0.00	0.00	0.01	0.00	0.00	0.00	0.00	0.00	0.00	0.00
**GH85**	0.00	0.06	0.00	0.00	0.01	0.00	0.05	0.00	0.00	0.02	0.02	0.01	0.00
**GH92**	1.23	0.73	0.20	1.06	1.27	1.16	1.67	1.76	1.81	2.10	1.23	0.98	1.20
**GH40**	0.00	0.00	0.00	0.00	0.00	0.00	0.00	0.00	0.00	0.00	0.00	0.00	0.00
**GT49**	0.00	0.00	0.00	0.00	0.01	0.00	0.00	0.00	0.00	0.00	0.00	0.00	0.00
**GH112**	0.09	0.19	0.01	0.15	0.12	0.19	0.13	0.11	0.09	0.10	0.15	0.07	0.47
**GT67**	0.00	0.00	0.00	0.00	0.00	0.00	0.00	0.00	0.00	0.00	0.00	0.00	0.00
**CE8**	0.16	0.14	0.00	0.11	0.11	0.26	0.32	0.28	0.21	0.19	0.28	0.16	0.10
**GT86**	0.00	0.00	0.00	0.00	0.00	0.00	0.00	0.00	0.00	0.00	0.00	0.00	0.00
**GH101**	0.00	0.00	0.00	0.00	0.00	0.00	0.02	0.00	0.00	0.00	0.00	0.00	0.00
**GH81**	0.00	0.00	0.00	0.00	0.00	0.00	0.00	0.00	0.00	0.00	0.00	0.00	0.00
**GT93**	0.00	0.00	0.00	0.00	0.00	0.00	0.00	0.00	0.00	0.00	0.00	0.00	0.00
**GT63**	0.00	0.00	0.00	0.00	0.00	0.00	0.00	0.00	0.00	0.00	0.00	0.00	0.00
**CE11**	0.21	0.23	0.00	0.17	0.27	0.18	0.15	0.31	0.42	0.20	0.24	0.26	0.37
**GT19**	0.09	0.16	0.03	0.10	0.08	0.09	0.11	0.28	0.33	0.14	0.11	0.11	0.16
**GT33**	0.00	0.00	0.00	0.00	0.00	0.00	0.00	0.00	0.00	0.00	0.00	0.00	0.00
**GH21**	0.00	0.00	0.00	0.00	0.00	0.00	0.00	0.00	0.00	0.00	0.00	0.00	0.00
**GT68**	0.00	0.00	0.00	0.00	0.00	0.00	0.00	0.00	0.00	0.00	0.00	0.00	0.00
**GH89**	0.21	0.08	0.02	0.17	0.19	0.15	0.28	0.24	0.16	0.32	0.24	0.10	0.00
**GT82**	0.00	0.00	0.00	0.00	0.00	0.00	0.00	0.00	0.02	0.02	0.00	0.00	0.00
**GH30**	0.13	0.08	0.01	0.15	0.16	0.23	0.15	0.31	0.26	0.39	0.22	0.19	0.10
**GH20**	0.91	0.63	0.07	0.85	0.96	0.77	1.08	1.18	0.98	1.07	1.36	0.96	0.31
**GH52**	0.00	0.00	0.00	0.00	0.00	0.00	0.00	0.00	0.00	0.00	0.00	0.00	0.00
**GT84**	0.03	0.11	0.00	0.00	0.03	0.03	0.00	0.00	0.02	0.00	0.00	0.02	0.00
**GT15**	0.00	0.00	0.00	0.00	0.00	0.00	0.00	0.00	0.00	0.00	0.00	0.00	0.00
**GH93**	0.00	0.00	0.00	0.00	0.00	0.00	0.03	0.00	0.00	0.00	0.00	0.00	0.00
**PL16**	0.00	0.00	0.00	0.00	0.00	0.00	0.00	0.00	0.00	0.00	0.00	0.00	0.00
**GH26**	0.13	0.14	0.00	0.11	0.13	0.09	0.20	0.18	0.30	0.17	0.15	0.15	0.16

CAZY_group	DA-AD-39	DA-AD-4	DA-AD-40	DA-AD-41	DA-AD-42	DA-AD-43	DA-AD-44	DA-AD-45	DA-AD-46	DA-AD-47	DA-AD-48	DA-AD-49	DA-AD-5
GT75	0.00	0.00	0.00	0.00	0.00	0.00	0.00	0.00	0.00	0.00	0.00	0.00	0.00
GH53	0.09	0.17	0.00	0.12	0.08	0.16	0.21	0.23	0.07	0.14	0.22	0.15	0.47
GT88	0.00	0.00	0.00	0.00	0.00	0.00	0.00	0.00	0.00	0.00	0.00	0.00	0.00
GT24	0.00	0.00	0.00	0.00	0.00	0.00	0.00	0.00	0.00	0.00	0.00	0.00	0.00
GT81	0.00	0.00	0.00	0.00	0.00	0.00	0.00	0.00	0.00	0.00	0.00	0.00	0.00
GH4	0.13	0.19	0.00	0.12	0.16	0.12	0.08	0.10	0.00	0.02	0.00	0.08	0.00
GT78	0.00	0.00	0.00	0.00	0.00	0.00	0.00	0.00	0.00	0.00	0.00	0.00	0.00
GH8	0.01	0.02	0.00	0.08	0.06	0.06	0.09	0.04	0.09	0.17	0.02	0.06	0.05
GH70	0.00	0.00	0.00	0.00	0.00	0.00	0.00	0.00	0.00	0.00	0.00	0.00	0.00
GH122	0.00	0.00	0.00	0.00	0.00	0.00	0.00	0.00	0.00	0.00	0.00	0.00	0.00
GH74	0.02	0.01	0.00	0.01	0.00	0.03	0.05	0.00	0.00	0.05	0.06	0.05	0.00
GT41	0.00	0.01	0.00	0.02	0.00	0.00	0.00	0.00	0.00	0.00	0.00	0.01	0.00
PL1	0.21	0.12	0.02	0.15	0.14	0.20	0.49	0.26	0.21	0.41	0.35	0.18	0.37
GH15	0.03	0.00	0.00	0.04	0.07	0.05	0.08	0.05	0.14	0.09	0.02	0.02	0.00
GH32	0.32	0.44	0.03	0.48	0.34	0.32	0.57	0.57	0.49	0.34	0.45	0.39	0.42
GH44	0.00	0.00	0.00	0.00	0.00	0.00	0.01	0.00	0.00	0.02	0.00	0.00	0.00
GT6	0.00	0.01	0.00	0.00	0.00	0.00	0.00	0.00	0.00	0.00	0.00	0.00	0.00
GH13	2.75	3.73	0.26	3.20	2.65	2.93	3.28	3.35	3.23	3.56	2.98	2.82	4.91
GT80	0.03	0.01	0.01	0.04	0.03	0.00	0.00	0.00	0.02	0.02	0.02	0.01	0.00
GH71	0.00	0.00	0.00	0.00	0.00	0.00	0.00	0.00	0.00	0.00	0.00	0.00	0.00
GH63	0.11	0.07	0.01	0.05	0.12	0.08	0.08	0.15	0.12	0.17	0.04	0.08	0.16
PL21	0.05	0.00	0.00	0.00	0.00	0.00	0.03	0.00	0.00	0.02	0.00	0.00	0.00
GH65	0.09	0.10	0.00	0.13	0.11	0.10	0.14	0.19	0.07	0.17	0.22	0.10	0.05
CE4	0.53	0.64	0.06	0.54	0.47	0.57	0.76	0.64	0.60	0.70	0.54	0.56	0.42
CE14	0.01	0.01	0.00	0.02	0.03	0.03	0.00	0.00	0.00	0.02	0.00	0.01	0.00
GH115	0.22	0.11	0.00	0.15	0.31	0.29	0.29	0.24	0.42	0.26	0.22	0.16	0.16
GH104	0.00	0.00	0.00	0.00	0.00	0.00	0.00	0.00	0.00	0.00	0.00	0.00	0.00
GH111	0.00	0.00	0.00	0.00	0.00	0.00	0.00	0.00	0.00	0.00	0.00	0.00	0.00
GH95	0.36	0.37	0.01	0.31	0.39	0.55	0.64	0.76	0.86	0.72	0.58	0.59	0.47
GT70	0.00	0.00	0.00	0.00	0.00	0.00	0.00	0.00	0.00	0.00	0.00	0.00	0.00
CE10	0.13	0.04	0.01	0.09	0.14	0.09	0.13	0.13	0.23	0.14	0.19	0.16	0.00
GH10	0.04	0.15	0.00	0.11	0.11	0.11	0.32	0.10	0.16	0.17	0.17	0.06	0.16
GH130	0.22	0.31	0.01	0.34	0.30	0.31	0.40	0.26	0.28	0.39	0.41	0.23	0.26
PL20	0.00	0.00	0.00	0.00	0.00	0.00	0.00	0.00	0.00	0.00	0.00	0.00	0.00
GH69	0.00	0.00	0.00	0.00	0.00	0.00	0.00	0.00	0.00	0.00	0.00	0.00	0.00
PL2	0.00	0.00	0.00	0.00	0.00	0.00	0.00	0.00	0.00	0.00	0.00	0.00	0.00
GH49	0.00	0.00	0.00	0.00	0.00	0.00	0.00	0.00	0.00	0.00	0.00	0.00	0.00
GH25	0.26	0.35	0.03	0.35	0.34	0.42	0.45	0.67	0.28	0.43	0.58	0.41	0.47
GH59	0.02	0.00	0.00	0.02	0.01	0.04	0.03	0.00	0.00	0.00	0.02	0.02	0.00
GH35	0.25	0.19	0.00	0.21	0.28	0.29	0.28	0.37	0.28	0.26	0.45	0.23	0.10
GT39	0.01	0.01	0.00	0.00	0.00	0.00	0.00	0.00	0.00	0.00	0.00	0.00	0.00
GT57	0.00	0.00	0.00	0.00	0.01	0.00	0.00	0.00	0.00	0.00	0.00	0.00	0.00
GH129	0.00	0.00	0.00	0.01	0.00	0.00	0.02	0.00	0.00	0.00	0.00	0.01	0.00
GT22	0.00	0.01	0.00	0.00	0.01	0.00	0.00	0.00	0.00	0.00	0.00	0.00	0.00
PL12	0.16	0.04	0.00	0.02	0.09	0.05	0.11	0.06	0.05	0.15	0.06	0.05	0.00
PL18	0.00	0.00	0.00	0.00	0.00	0.00	0.00	0.00	0.00	0.00	0.00	0.00	0.00
GT92	0.00	0.00	0.00	0.00	0.00	0.00	0.00	0.00	0.00	0.00	0.00	0.00	0.00
GT44	0.00	0.00	0.00	0.00	0.00	0.00	0.00	0.00	0.00	0.00	0.00	0.00	0.00
GT29	0.00	0.00	0.00	0.00	0.00	0.00	0.00	0.00	0.00	0.00	0.00	0.00	0.00
GT13	0.00	0.00	0.00	0.00	0.00	0.00	0.00	0.00	0.00	0.00	0.00	0.00	0.00

CAZY_group	DA-AD-39	DA-AD-4	DA-AD-40	DA-AD-41	DA-AD-42	DA-AD-43	DA-AD-44	DA-AD-45	DA-AD-46	DA-AD-47	DA-AD-48	DA-AD-49	DA-AD-5
**GT52**	0.00	0.00	0.00	0.00	0.00	0.00	0.00	0.00	0.00	0.00	0.00	0.00	0.00
**GT64**	0.00	0.00	0.00	0.00	0.00	0.00	0.00	0.00	0.00	0.00	0.00	0.00	0.00
**GT85**	0.00	0.00	0.00	0.00	0.00	0.00	0.00	0.00	0.00	0.00	0.00	0.00	0.00
**GT16**	0.00	0.00	0.00	0.00	0.00	0.00	0.00	0.00	0.00	0.00	0.00	0.00	0.00
**GH56**	0.00	0.00	0.00	0.00	0.00	0.00	0.00	0.00	0.00	0.00	0.00	0.00	0.00
**GT60**	0.00	0.00	0.00	0.00	0.00	0.00	0.00	0.00	0.00	0.00	0.00	0.00	0.00
**GH41**	0.00	0.00	0.00	0.00	0.00	0.00	0.00	0.00	0.00	0.00	0.00	0.00	0.00
**CE7**	0.08	0.05	0.00	0.09	0.11	0.08	0.09	0.21	0.09	0.17	0.22	0.11	0.05
**GH39**	0.06	0.05	0.00	0.04	0.03	0.06	0.03	0.03	0.07	0.03	0.09	0.06	0.10
**GT30**	0.21	0.14	0.04	0.16	0.18	0.14	0.17	0.26	0.37	0.29	0.26	0.26	0.21
**PL15**	0.07	0.00	0.00	0.04	0.09	0.06	0.16	0.03	0.02	0.10	0.09	0.01	0.00
**GH102**	0.04	0.01	0.00	0.03	0.03	0.01	0.01	0.01	0.02	0.02	0.00	0.01	0.00

CAZY_group	DA-AD-50	DA-AD-51	DA-AD-52	DA-AD-53	DA-AD-54	DA-AD-55	DA-AD-56	DA-AD-57	DA-AD-58	DA-AD-59	DA-AD-6	DA-AD-60	DA-AD-61
GH29	0.66	0.62	0.33	0.36	0.59	0.82	0.61	0.72	0.56	0.56	0.53	0.83	0.78
PL4	0.00	0.00	0.00	0.00	0.00	0.00	0.00	0.00	0.00	0.00	0.00	0.00	0.02
GH23	1.04	0.94	0.81	0.80	1.00	1.09	1.13	0.89	0.75	0.92	0.96	0.95	0.99
CE6	0.08	0.01	0.04	0.00	0.03	0.01	0.00	0.04	0.02	0.02	0.00	0.03	0.03
GH72	0.13	0.07	0.10	0.10	0.15	0.19	0.09	0.18	0.13	0.13	0.12	0.08	0.14
GH114	0.00	0.00	0.00	0.00	0.00	0.00	0.00	0.00	0.00	0.00	0.00	0.00	0.00
GH78	0.74	0.36	0.20	0.47	0.55	0.96	0.89	0.72	0.77	0.53	0.43	0.85	0.68
PL19	0.00	0.00	0.00	0.00	0.00	0.00	0.00	0.00	0.00	0.00	0.00	0.00	0.00
GT27	0.02	0.00	0.00	0.00	0.00	0.00	0.00	0.00	0.00	0.00	0.00	0.00	0.00
GH5	0.85	0.88	0.49	0.38	0.60	0.53	0.43	0.49	0.50	0.51	0.43	0.34	0.25
GT40	0.00	0.00	0.00	0.00	0.00	0.00	0.00	0.00	0.00	0.00	0.00	0.00	0.00
CE13	0.00	0.00	0.00	0.00	0.00	0.00	0.00	0.00	0.01	0.00	0.00	0.00	0.00
GT37	0.00	0.00	0.00	0.00	0.00	0.00	0.00	0.00	0.00	0.00	0.00	0.00	0.00
GH33	0.20	0.19	0.02	0.14	0.14	0.29	0.17	0.21	0.17	0.18	0.19	0.24	0.20
GT65	0.00	0.00	0.00	0.00	0.00	0.00	0.00	0.00	0.00	0.00	0.00	0.00	0.00
GH86	0.00	0.00	0.00	0.00	0.00	0.01	0.00	0.00	0.00	0.00	0.00	0.00	0.00
GH123	0.13	0.06	0.06	0.06	0.09	0.16	0.16	0.10	0.13	0.08	0.10	0.14	0.07
GH96	0.00	0.00	0.00	0.00	0.00	0.00	0.00	0.00	0.00	0.00	0.00	0.00	0.00
GH14	0.00	0.00	0.00	0.00	0.00	0.00	0.00	0.00	0.00	0.00	0.00	0.00	0.00
CE3	0.00	0.00	0.00	0.00	0.00	0.00	0.00	0.00	0.00	0.00	0.00	0.00	0.00
PL10	0.16	0.12	0.00	0.06	0.15	0.10	0.11	0.09	0.09	0.08	0.05	0.08	0.07
GT48	0.00	0.00	0.00	0.00	0.00	0.00	0.00	0.00	0.00	0.00	0.00	0.00	0.00
PL6	0.02	0.00	0.00	0.00	0.00	0.00	0.00	0.00	0.00	0.00	0.00	0.00	0.00
GT83	0.06	0.01	0.05	0.06	0.03	0.09	0.10	0.09	0.09	0.04	0.06	0.06	0.05
GH126	0.00	0.00	0.00	0.00	0.00	0.00	0.00	0.00	0.00	0.00	0.00	0.00	0.00
GH9	0.49	0.25	0.29	0.08	0.10	0.48	0.19	0.21	0.30	0.30	0.30	0.07	0.05
GH75	0.00	0.00	0.00	0.00	0.00	0.00	0.00	0.00	0.00	0.00	0.00	0.00	0.00
GT71	0.00	0.00	0.00	0.00	0.00	0.00	0.00	0.00	0.00	0.00	0.00	0.00	0.00
GH46	0.00	0.00	0.00	0.00	0.00	0.00	0.00	0.00	0.00	0.00	0.00	0.00	0.00
GT61	0.00	0.00	0.00	0.00	0.00	0.00	0.00	0.00	0.00	0.00	0.00	0.00	0.00
GT3	0.22	0.22	0.17	0.12	0.18	0.27	0.13	0.13	0.13	0.12	0.21	0.18	0.15
PL3	0.00	0.00	0.00	0.00	0.00	0.00	0.00	0.00	0.00	0.00	0.00	0.00	0.00
GH108	0.03	0.04	0.01	0.01	0.03	0.02	0.03	0.03	0.05	0.02	0.09	0.03	0.05
GT56	0.00	0.00	0.00	0.00	0.00	0.01	0.00	0.00	0.00	0.00	0.00	0.00	0.00
PL11	0.20	0.16	0.02	0.13	0.08	0.10	0.14	0.12	0.11	0.09	0.06	0.13	0.06
GH45	0.00	0.00	0.00	0.00	0.00	0.00	0.00	0.00	0.01	0.00	0.00	0.00	0.00
GT34	0.00	0.00	0.00	0.00	0.00	0.00	0.00	0.00	0.00	0.00	0.00	0.00	0.00
PL14	0.00	0.00	0.00	0.00	0.00	0.00	0.00	0.00	0.00	0.00	0.00	0.00	0.00
GH19	0.00	0.00	0.00	0.02	0.01	0.00	0.00	0.00	0.01	0.02	0.00	0.00	0.00
GH118	0.00	0.00	0.00	0.00	0.00	0.00	0.00	0.00	0.00	0.00	0.00	0.00	0.00
GH82	0.00	0.00	0.00	0.00	0.00	0.00	0.00	0.00	0.00	0.00	0.00	0.00	0.00
GH76	0.20	0.03	0.00	0.11	0.03	0.02	0.19	0.19	0.13	0.18	0.07	0.20	0.16
GT4	1.95	1.59	1.06	1.40	1.80	1.93	1.52	2.20	1.61	1.42	1.85	1.45	1.57
GT87	0.02	0.00	0.00	0.01	0.01	0.00	0.03	0.01	0.00	0.00	0.00	0.00	0.01
GH24	0.13	0.10	0.05	0.05	0.15	0.12	0.11	0.06	0.06	0.12	0.07	0.10	0.10
GH90	0.00	0.00	0.00	0.00	0.00	0.00	0.00	0.00	0.00	0.00	0.00	0.00	0.00
GH57	0.13	0.22	0.14	0.06	0.12	0.15	0.11	0.11	0.12	0.09	0.09	0.11	0.09
GH36	0.82	0.59	0.50	0.49	0.82	0.70	0.49	0.65	0.65	0.60	0.66	0.58	0.85
GT46	0.00	0.00	0.00	0.00	0.00	0.00	0.00	0.00	0.00	0.00	0.00	0.00	0.00
GH66	0.05	0.03	0.02	0.03	0.04	0.03	0.04	0.04	0.03	0.03	0.00	0.03	0.02

CAZY_group	DA-AD-50	DA-AD-51	DA-AD-52	DA-AD-53	DA-AD-54	DA-AD-55	DA-AD-56	DA-AD-57	DA-AD-58	DA-AD-59	DA-AD-6	DA-AD-60	DA-AD-61
**GH48**	0.00	0.00	0.00	0.00	0.00	0.00	0.00	0.00	0.00	0.00	0.00	0.00	0.00
**GT53**	0.00	0.00	0.00	0.00	0.00	0.00	0.00	0.00	0.00	0.00	0.00	0.00	0.00
**GH60**	0.00	0.00	0.00	0.00	0.00	0.00	0.00	0.00	0.00	0.00	0.00	0.00	0.00
**GT31**	0.00	0.00	0.00	0.00	0.00	0.00	0.00	0.00	0.00	0.00	0.00	0.00	0.00
**GH105**	0.64	0.38	0.10	0.24	0.34	0.47	0.47	0.50	0.45	0.43	0.23	0.47	0.45
**GT23**	0.00	0.01	0.00	0.01	0.01	0.01	0.01	0.02	0.00	0.00	0.01	0.01	0.00
**GH11**	0.00	0.00	0.01	0.00	0.01	0.02	0.00	0.00	0.00	0.01	0.02	0.00	0.00
**GT38**	0.00	0.00	0.00	0.00	0.00	0.00	0.00	0.00	0.00	0.00	0.00	0.00	0.00
**GT43**	0.00	0.00	0.00	0.00	0.00	0.00	0.00	0.00	0.00	0.00	0.00	0.00	0.00
**GH99**	0.00	0.00	0.00	0.00	0.00	0.00	0.00	0.01	0.00	0.00	0.00	0.00	0.00
**GH38**	0.28	0.12	0.07	0.19	0.16	0.19	0.14	0.28	0.22	0.28	0.21	0.16	0.20
**GH109**	0.36	0.28	0.12	0.15	0.20	0.26	0.29	0.28	0.25	0.20	0.25	0.23	0.20
**GH2**	4.39	3.56	1.75	2.71	3.17	4.12	3.83	3.78	3.42	3.03	2.94	3.02	3.74
**GT28**	0.49	0.46	0.37	0.43	0.47	0.40	0.40	0.45	0.38	0.41	0.51	0.41	0.45
**GH42**	0.22	0.22	0.06	0.19	0.14	0.15	0.09	0.23	0.12	0.16	0.19	0.04	0.20
**GT58**	0.00	0.00	0.00	0.00	0.00	0.00	0.00	0.00	0.00	0.00	0.00	0.00	0.00
**GH61**	0.00	0.00	0.00	0.00	0.00	0.00	0.00	0.00	0.00	0.00	0.00	0.00	0.00
**GH80**	0.00	0.00	0.00	0.00	0.00	0.00	0.00	0.00	0.00	0.00	0.00	0.00	0.00
**GH98**	0.06	0.00	0.00	0.00	0.01	0.00	0.00	0.01	0.01	0.01	0.00	0.00	0.00
**GH6**	0.00	0.00	0.00	0.00	0.00	0.00	0.00	0.00	0.00	0.00	0.00	0.00	0.00
**GT1**	0.03	0.04	0.04	0.04	0.04	0.04	0.03	0.04	0.04	0.03	0.03	0.07	0.05
**GH28**	1.13	0.56	0.33	0.41	0.72	0.65	1.02	0.82	0.83	0.61	0.45	0.75	0.64
**GT42**	0.00	0.00	0.00	0.00	0.00	0.00	0.00	0.00	0.00	0.00	0.00	0.00	0.00
**GH128**	0.00	0.00	0.00	0.00	0.00	0.00	0.00	0.00	0.00	0.00	0.00	0.00	0.00
**CE12**	0.24	0.12	0.14	0.07	0.11	0.12	0.17	0.17	0.13	0.09	0.06	0.13	0.10
**CE16**	0.00	0.00	0.00	0.00	0.00	0.00	0.00	0.00	0.00	0.00	0.00	0.00	0.00
**GT89**	0.00	0.00	0.00	0.00	0.00	0.00	0.00	0.00	0.00	0.00	0.00	0.00	0.00
**GT11**	0.05	0.07	0.04	0.01	0.04	0.05	0.06	0.13	0.10	0.06	0.06	0.10	0.01
**GT77**	0.00	0.00	0.00	0.01	0.00	0.01	0.00	0.00	0.00	0.00	0.01	0.00	0.00
**GT55**	0.00	0.00	0.00	0.00	0.00	0.00	0.00	0.00	0.00	0.00	0.00	0.00	0.00
**GT18**	0.00	0.00	0.00	0.00	0.00	0.00	0.00	0.00	0.00	0.00	0.00	0.00	0.00
**GH87**	0.00	0.00	0.00	0.00	0.01	0.00	0.00	0.00	0.00	0.00	0.02	0.00	0.00
**PL5**	0.00	0.00	0.00	0.00	0.00	0.00	0.00	0.00	0.00	0.00	0.00	0.00	0.00
**GH22**	0.00	0.00	0.00	0.00	0.00	0.00	0.00	0.00	0.00	0.00	0.00	0.00	0.00
**CE2**	0.14	0.07	0.08	0.06	0.07	0.09	0.06	0.06	0.09	0.10	0.10	0.06	0.05
**GH120**	0.02	0.01	0.05	0.06	0.07	0.10	0.00	0.06	0.12	0.06	0.07	0.03	0.08
**GH73**	0.42	0.42	0.42	0.37	0.58	0.58	0.50	0.47	0.55	0.43	0.56	0.28	0.52
**GT17**	0.00	0.00	0.00	0.00	0.00	0.00	0.00	0.00	0.00	0.00	0.00	0.01	0.00
**GH124**	0.00	0.00	0.00	0.00	0.00	0.00	0.00	0.00	0.00	0.00	0.00	0.00	0.00
**GH54**	0.00	0.00	0.00	0.00	0.00	0.00	0.00	0.00	0.00	0.00	0.00	0.00	0.00
**GH100**	0.00	0.00	0.00	0.00	0.00	0.00	0.00	0.00	0.00	0.00	0.00	0.00	0.00
**GH113**	0.03	0.10	0.04	0.03	0.06	0.04	0.03	0.04	0.02	0.02	0.02	0.03	0.01
**GT5**	0.36	0.42	0.53	0.37	0.44	0.40	0.36	0.45	0.47	0.40	0.57	0.30	0.52
**GH94**	0.52	0.41	0.36	0.37	0.52	0.42	0.20	0.45	0.40	0.40	0.43	0.35	0.30
**GT66**	0.00	0.00	0.00	0.00	0.01	0.01	0.00	0.00	0.00	0.00	0.00	0.00	0.00
**GT94**	0.00	0.00	0.00	0.00	0.00	0.00	0.00	0.00	0.00	0.00	0.00	0.00	0.00
**GH58**	0.00	0.00	0.00	0.00	0.00	0.00	0.00	0.00	0.00	0.00	0.00	0.00	0.00
**GH1**	0.31	0.36	0.65	0.40	0.31	0.42	0.36	0.42	0.36	0.22	0.51	0.34	0.76
**PL22**	0.00	0.00	0.00	0.00	0.00	0.00	0.00	0.00	0.00	0.00	0.00	0.00	0.00

CAZY_group	DA-AD-50	DA-AD-51	DA-AD-52	DA-AD-53	DA-AD-54	DA-AD-55	DA-AD-56	DA-AD-57	DA-AD-58	DA-AD-59	DA-AD-6	DA-AD-60	DA-AD-61
GT76	0.00	0.00	0.00	0.00	0.00	0.00	0.00	0.00	0.00	0.00	0.00	0.00	0.00
GT32	0.17	0.01	0.11	0.09	0.11	0.13	0.07	0.11	0.11	0.07	0.12	0.07	0.06
GT47	0.06	0.06	0.05	0.02	0.02	0.06	0.04	0.04	0.02	0.04	0.02	0.04	0.06
GH84	0.11	0.13	0.04	0.04	0.08	0.14	0.13	0.12	0.06	0.04	0.10	0.08	0.09
GH83	0.00	0.00	0.00	0.00	0.00	0.00	0.00	0.00	0.00	0.00	0.00	0.00	0.00
GT59	0.00	0.00	0.00	0.00	0.00	0.00	0.00	0.00	0.00	0.00	0.00	0.00	0.00
GH34	0.00	0.00	0.00	0.00	0.00	0.00	0.00	0.00	0.00	0.00	0.00	0.00	0.00
GT14	0.05	0.04	0.00	0.00	0.01	0.02	0.04	0.04	0.02	0.01	0.02	0.03	0.00
GH47	0.00	0.00	0.00	0.00	0.00	0.00	0.00	0.00	0.00	0.00	0.00	0.00	0.00
GH97	0.99	0.80	0.25	0.44	0.84	0.83	0.86	0.78	0.81	0.65	0.64	0.54	0.69
GH50	0.05	0.00	0.00	0.00	0.00	0.02	0.04	0.02	0.02	0.06	0.00	0.07	0.03
GT26	0.13	0.28	0.21	0.13	0.20	0.22	0.16	0.29	0.21	0.17	0.15	0.23	0.17
GH18	0.75	0.32	0.38	0.51	0.39	0.49	0.53	0.63	0.42	0.47	0.64	0.61	0.50
GH37	0.00	0.00	0.00	0.02	0.00	0.01	0.00	0.00	0.00	0.00	0.01	0.01	0.01
PL13	0.06	0.01	0.00	0.02	0.01	0.02	0.03	0.04	0.05	0.03	0.03	0.04	0.03
GH27	0.16	0.23	0.21	0.19	0.13	0.16	0.16	0.19	0.24	0.21	0.22	0.13	0.27
GT54	0.00	0.00	0.00	0.00	0.00	0.00	0.00	0.00	0.00	0.00	0.00	0.00	0.00
GT91	0.00	0.00	0.00	0.00	0.00	0.00	0.00	0.00	0.00	0.00	0.00	0.00	0.00
GT72	0.00	0.00	0.00	0.00	0.00	0.00	0.00	0.00	0.00	0.00	0.00	0.00	0.00
GH67	0.08	0.01	0.04	0.06	0.12	0.12	0.11	0.05	0.07	0.07	0.08	0.04	0.02
GH12	0.00	0.00	0.00	0.00	0.00	0.00	0.00	0.00	0.00	0.00	0.00	0.00	0.00
GH91	0.03	0.00	0.02	0.01	0.00	0.02	0.07	0.02	0.03	0.02	0.01	0.06	0.03
GT69	0.00	0.00	0.00	0.00	0.00	0.00	0.00	0.00	0.00	0.00	0.00	0.00	0.00
GH106	0.19	0.14	0.13	0.15	0.21	0.21	0.24	0.20	0.20	0.22	0.13	0.18	0.17
GT62	0.00	0.00	0.00	0.00	0.00	0.00	0.00	0.00	0.00	0.00	0.00	0.00	0.00
GH17	0.00	0.00	0.00	0.00	0.00	0.00	0.00	0.00	0.00	0.00	0.00	0.00	0.00
GH88	0.33	0.17	0.11	0.13	0.18	0.32	0.39	0.30	0.34	0.28	0.21	0.44	0.34
CE5	0.00	0.00	0.00	0.00	0.00	0.00	0.00	0.00	0.00	0.00	0.00	0.00	0.00
GT8	0.08	0.10	0.12	0.08	0.07	0.13	0.09	0.10	0.08	0.04	0.10	0.08	0.09
GT50	0.00	0.00	0.00	0.00	0.00	0.00	0.00	0.00	0.00	0.00	0.00	0.00	0.00
GT20	0.06	0.03	0.00	0.01	0.04	0.09	0.09	0.07	0.09	0.09	0.08	0.13	0.08
PL9	0.17	0.16	0.07	0.01	0.07	0.09	0.14	0.10	0.08	0.13	0.03	0.04	0.08
GH55	0.03	0.06	0.01	0.00	0.02	0.04	0.01	0.04	0.02	0.01	0.02	0.00	0.03
GH64	0.00	0.00	0.00	0.00	0.00	0.00	0.00	0.00	0.00	0.00	0.01	0.00	0.00
GH127	0.35	0.23	0.06	0.22	0.32	0.35	0.31	0.41	0.26	0.19	0.34	0.21	0.20
GH103	0.00	0.00	0.00	0.00	0.00	0.00	0.00	0.00	0.00	0.00	0.00	0.00	0.00
GH3	2.82	2.07	1.14	1.90	2.63	2.92	2.36	2.60	2.40	1.97	2.05	2.05	2.32
GT45	0.00	0.00	0.00	0.00	0.00	0.00	0.00	0.00	0.00	0.00	0.00	0.00	0.00
GT74	0.00	0.00	0.00	0.00	0.00	0.00	0.00	0.00	0.00	0.00	0.00	0.00	0.00
GT90	0.00	0.00	0.00	0.00	0.00	0.00	0.00	0.00	0.00	0.00	0.00	0.00	0.00
GH116	0.06	0.09	0.01	0.01	0.04	0.07	0.06	0.04	0.08	0.09	0.05	0.03	0.02
GH31	0.93	0.80	0.45	0.62	0.89	1.03	0.89	1.14	1.02	0.78	0.64	0.79	0.94
GT12	0.00	0.00	0.00	0.00	0.00	0.00	0.00	0.00	0.00	0.00	0.00	0.00	0.00
CE1	0.61	0.33	0.24	0.25	0.41	0.44	0.57	0.41	0.47	0.27	0.31	0.37	0.39
GH121	0.00	0.00	0.00	0.00	0.00	0.01	0.00	0.00	0.00	0.00	0.00	0.00	0.00
GH79	0.00	0.00	0.00	0.03	0.00	0.01	0.00	0.02	0.00	0.00	0.00	0.00	0.00
GT73	0.00	0.00	0.00	0.01	0.00	0.00	0.00	0.00	0.00	0.00	0.00	0.00	0.00
GH77	0.72	0.62	0.51	0.70	0.63	0.61	0.53	0.61	0.59	0.49	0.64	0.61	0.72
PL17	0.00	0.00	0.00	0.00	0.00	0.00	0.00	0.00	0.00	0.02	0.00	0.00	0.00
PL8	0.38	0.16	0.04	0.08	0.13	0.26	0.16	0.19	0.28	0.14	0.12	0.21	0.24

CAZY_group	DA-AD-50	DA-AD-51	DA-AD-52	DA-AD-53	DA-AD-54	DA-AD-55	DA-AD-56	DA-AD-57	DA-AD-58	DA-AD-59	DA-AD-6	DA-AD-60	DA-AD-61
CE9	0.30	0.23	0.19	0.20	0.26	0.35	0.31	0.37	0.30	0.21	0.22	0.23	0.41
CE15	0.03	0.01	0.00	0.01	0.00	0.02	0.03	0.04	0.01	0.01	0.03	0.00	0.01
GT79	0.00	0.00	0.00	0.00	0.00	0.00	0.00	0.00	0.00	0.00	0.00	0.00	0.00
GT10	0.00	0.00	0.00	0.00	0.00	0.00	0.03	0.02	0.01	0.00	0.00	0.03	0.01
GH119	0.00	0.00	0.00	0.00	0.00	0.00	0.00	0.00	0.00	0.00	0.00	0.00	0.00
GH16	0.20	0.13	0.10	0.06	0.16	0.21	0.10	0.22	0.22	0.20	0.13	0.28	0.15
GH43	2.53	1.53	0.91	1.01	2.06	2.36	2.24	2.61	2.21	1.43	1.45	1.30	1.33
GH51	0.49	0.38	0.33	0.37	0.49	0.56	0.57	0.60	0.47	0.35	0.44	0.37	0.49
GT35	0.99	0.80	0.74	0.94	0.99	0.78	0.73	0.73	0.75	0.73	0.87	0.73	0.82
GT25	0.00	0.00	0.00	0.00	0.00	0.00	0.00	0.00	0.00	0.00	0.00	0.03	0.00
GT2	5.05	4.78	3.03	2.98	3.92	4.26	4.53	4.52	3.99	3.36	3.60	3.40	3.90
GT51	0.74	1.01	0.63	0.69	0.88	1.05	0.84	0.89	0.73	0.58	0.86	0.68	0.73
GH107	0.00	0.00	0.00	0.00	0.00	0.00	0.00	0.00	0.00	0.00	0.00	0.00	0.00
GH7	0.00	0.00	0.00	0.00	0.00	0.00	0.00	0.00	0.00	0.00	0.00	0.00	0.00
GT7	0.00	0.00	0.00	0.00	0.00	0.00	0.00	0.00	0.00	0.00	0.00	0.00	0.00
GT36	0.00	0.00	0.00	0.00	0.00	0.00	0.00	0.00	0.00	0.00	0.00	0.00	0.00
PL7	0.00	0.00	0.00	0.00	0.00	0.00	0.00	0.01	0.00	0.00	0.00	0.00	0.00
GH110	0.09	0.07	0.00	0.03	0.08	0.12	0.13	0.13	0.10	0.06	0.09	0.14	0.10
GT21	0.00	0.00	0.00	0.00	0.00	0.00	0.00	0.00	0.00	0.00	0.00	0.00	0.00
GH62	0.00	0.00	0.00	0.00	0.00	0.00	0.00	0.00	0.00	0.00	0.00	0.00	0.00
GH125	0.19	0.14	0.07	0.11	0.10	0.17	0.21	0.17	0.17	0.21	0.10	0.13	0.11
GH117	0.09	0.07	0.01	0.03	0.04	0.06	0.13	0.09	0.08	0.03	0.07	0.11	0.06
GH68	0.00	0.00	0.00	0.00	0.00	0.00	0.00	0.00	0.00	0.00	0.00	0.00	0.00
GH85	0.00	0.00	0.00	0.00	0.00	0.03	0.03	0.03	0.00	0.00	0.00	0.01	0.00
GH92	1.79	0.81	0.44	0.94	1.10	1.82	1.76	1.51	1.53	1.21	1.08	1.44	1.28
GH40	0.00	0.00	0.00	0.00	0.00	0.00	0.00	0.00	0.00	0.00	0.00	0.00	0.00
GT49	0.00	0.00	0.00	0.00	0.00	0.00	0.00	0.00	0.00	0.00	0.00	0.00	0.00
GH112	0.09	0.16	0.13	0.17	0.12	0.07	0.11	0.15	0.10	0.16	0.15	0.13	0.17
GT67	0.00	0.00	0.00	0.00	0.00	0.00	0.00	0.00	0.00	0.00	0.00	0.00	0.00
CE8	0.25	0.12	0.11	0.15	0.26	0.18	0.31	0.20	0.21	0.18	0.13	0.21	0.18
GT86	0.00	0.00	0.00	0.00	0.00	0.00	0.00	0.00	0.00	0.00	0.00	0.00	0.00
GH101	0.00	0.00	0.00	0.00	0.00	0.01	0.00	0.00	0.00	0.00	0.01	0.01	0.01
GH81	0.00	0.00	0.00	0.00	0.00	0.00	0.00	0.00	0.00	0.00	0.01	0.00	0.01
GT93	0.00	0.00	0.00	0.00	0.00	0.00	0.00	0.00	0.00	0.00	0.00	0.00	0.00
GT63	0.00	0.00	0.00	0.00	0.00	0.00	0.00	0.00	0.00	0.00	0.00	0.00	0.00
CE11	0.22	0.26	0.19	0.15	0.30	0.29	0.39	0.27	0.23	0.22	0.23	0.17	0.17
GT19	0.11	0.19	0.08	0.07	0.17	0.14	0.20	0.13	0.13	0.12	0.10	0.10	0.19
GT33	0.00	0.00	0.00	0.00	0.00	0.00	0.00	0.00	0.00	0.00	0.00	0.00	0.00
GH21	0.00	0.00	0.00	0.00	0.00	0.00	0.00	0.00	0.00	0.00	0.00	0.00	0.00
GT68	0.00	0.00	0.00	0.00	0.00	0.00	0.00	0.00	0.00	0.00	0.00	0.00	0.00
GH89	0.20	0.20	0.08	0.04	0.14	0.17	0.24	0.25	0.21	0.27	0.13	0.24	0.20
GT82	0.00	0.00	0.00	0.00	0.00	0.00	0.00	0.00	0.00	0.00	0.00	0.00	0.00
GH30	0.16	0.25	0.08	0.11	0.15	0.19	0.23	0.22	0.23	0.17	0.21	0.16	0.20
GH20	1.11	0.80	0.29	0.62	0.61	1.12	1.23	1.45	0.94	0.73	0.93	1.26	0.81
GH52	0.00	0.00	0.00	0.00	0.00	0.00	0.00	0.00	0.00	0.00	0.00	0.00	0.00
GT84	0.00	0.00	0.05	0.02	0.00	0.01	0.00	0.02	0.05	0.00	0.07	0.04	0.01
GT15	0.00	0.00	0.00	0.00	0.00	0.00	0.00	0.00	0.00	0.00	0.00	0.00	0.00
GH93	0.02	0.00	0.00	0.00	0.00	0.00	0.00	0.00	0.00	0.01	0.00	0.00	0.00
PL16	0.00	0.00	0.00	0.00	0.00	0.00	0.00	0.00	0.00	0.00	0.00	0.00	0.00
GH26	0.27	0.22	0.08	0.14	0.22	0.19	0.11	0.14	0.18	0.12	0.19	0.10	0.03

CAZY_group	DA-AD-50	DA-AD-51	DA-AD-52	DA-AD-53	DA-AD-54	DA-AD-55	DA-AD-56	DA-AD-57	DA-AD-58	DA-AD-59	DA-AD-6	DA-AD-60	DA-AD-61
**GT75**	0.00	0.00	0.00	0.00	0.00	0.00	0.00	0.00	0.00	0.00	0.00	0.00	0.00
**GH53**	0.20	0.33	0.06	0.18	0.09	0.17	0.09	0.15	0.17	0.11	0.08	0.10	0.15
**GT88**	0.00	0.00	0.00	0.00	0.00	0.00	0.00	0.00	0.00	0.00	0.00	0.00	0.00
**GT24**	0.00	0.00	0.00	0.00	0.00	0.00	0.00	0.00	0.00	0.00	0.00	0.00	0.00
**GT81**	0.00	0.00	0.00	0.00	0.00	0.00	0.00	0.00	0.00	0.00	0.00	0.00	0.00
**GH4**	0.08	0.06	0.11	0.09	0.09	0.16	0.06	0.11	0.08	0.10	0.13	0.04	0.23
**GT78**	0.00	0.00	0.00	0.00	0.00	0.00	0.00	0.00	0.00	0.00	0.00	0.00	0.00
**GH8**	0.08	0.06	0.05	0.09	0.07	0.09	0.06	0.08	0.10	0.11	0.08	0.01	0.01
**GH70**	0.00	0.00	0.00	0.00	0.00	0.00	0.00	0.00	0.00	0.00	0.00	0.00	0.00
**GH122**	0.00	0.00	0.00	0.00	0.00	0.00	0.00	0.00	0.00	0.00	0.00	0.00	0.00
**GH74**	0.02	0.01	0.01	0.01	0.04	0.02	0.01	0.04	0.02	0.04	0.02	0.03	0.00
**GT41**	0.02	0.03	0.02	0.00	0.00	0.01	0.01	0.01	0.01	0.00	0.00	0.03	0.00
**PL1**	0.41	0.16	0.08	0.21	0.17	0.19	0.21	0.20	0.17	0.11	0.10	0.25	0.23
**GH15**	0.05	0.00	0.00	0.00	0.05	0.04	0.06	0.06	0.07	0.01	0.03	0.04	0.08
**GH32**	0.50	0.48	0.23	0.22	0.40	0.47	0.39	0.43	0.32	0.31	0.45	0.27	0.59
**GH44**	0.00	0.00	0.00	0.00	0.00	0.01	0.00	0.00	0.00	0.00	0.00	0.00	0.00
**GT6**	0.00	0.00	0.00	0.00	0.00	0.00	0.00	0.00	0.00	0.00	0.01	0.00	0.01
**GH13**	3.18	3.75	2.84	2.93	3.06	3.25	2.82	3.23	3.00	2.57	3.16	2.39	3.44
**GT80**	0.00	0.00	0.00	0.03	0.02	0.04	0.01	0.04	0.01	0.01	0.05	0.01	0.03
**GH71**	0.00	0.00	0.00	0.00	0.00	0.00	0.00	0.00	0.00	0.00	0.00	0.00	0.00
**GH63**	0.17	0.07	0.04	0.06	0.11	0.12	0.14	0.13	0.11	0.08	0.12	0.07	0.09
**PL21**	0.05	0.01	0.00	0.00	0.03	0.01	0.03	0.02	0.03	0.00	0.00	0.04	0.00
**GH65**	0.14	0.10	0.02	0.11	0.11	0.24	0.10	0.15	0.12	0.09	0.22	0.10	0.18
**CE4**	0.63	0.71	0.70	0.57	0.69	0.59	0.64	0.66	0.65	0.62	0.64	0.45	0.64
**CE14**	0.03	0.01	0.00	0.01	0.02	0.05	0.00	0.05	0.02	0.02	0.01	0.00	0.02
**GH115**	0.24	0.22	0.11	0.21	0.16	0.18	0.21	0.27	0.20	0.22	0.20	0.25	0.15
**GH104**	0.00	0.00	0.00	0.00	0.00	0.00	0.00	0.00	0.00	0.00	0.00	0.00	0.00
**GH111**	0.00	0.00	0.00	0.00	0.00	0.00	0.00	0.00	0.00	0.00	0.00	0.00	0.00
**GH95**	0.52	0.41	0.27	0.30	0.45	0.44	0.57	0.49	0.57	0.41	0.36	0.56	0.48
**GT70**	0.00	0.00	0.00	0.00	0.00	0.00	0.00	0.00	0.00	0.00	0.00	0.00	0.00
**CE10**	0.17	0.10	0.05	0.12	0.13	0.10	0.14	0.14	0.15	0.11	0.15	0.13	0.15
**GH10**	0.16	0.09	0.07	0.06	0.18	0.19	0.07	0.16	0.13	0.13	0.08	0.06	0.05
**GH130**	0.35	0.26	0.13	0.27	0.31	0.35	0.33	0.41	0.36	0.35	0.19	0.28	0.37
**PL20**	0.00	0.00	0.00	0.00	0.00	0.00	0.00	0.00	0.00	0.00	0.00	0.00	0.00
**GH69**	0.00	0.00	0.00	0.00	0.00	0.00	0.00	0.00	0.00	0.00	0.00	0.00	0.00
**PL2**	0.00	0.00	0.00	0.00	0.00	0.00	0.00	0.00	0.00	0.00	0.00	0.00	0.00
**GH49**	0.00	0.00	0.00	0.00	0.00	0.00	0.00	0.00	0.00	0.00	0.00	0.00	0.00
**GH25**	0.61	0.52	0.50	0.41	0.50	0.44	0.36	0.34	0.32	0.31	0.34	0.23	0.40
**GH59**	0.03	0.04	0.01	0.01	0.02	0.01	0.00	0.04	0.03	0.02	0.01	0.01	0.00
**GH35**	0.28	0.30	0.17	0.20	0.36	0.34	0.24	0.31	0.35	0.27	0.10	0.35	0.28
**GT39**	0.00	0.00	0.01	0.00	0.00	0.00	0.00	0.01	0.00	0.01	0.00	0.00	0.01
**GT57**	0.00	0.00	0.00	0.00	0.00	0.00	0.00	0.00	0.00	0.00	0.00	0.00	0.00
**GH129**	0.00	0.00	0.00	0.00	0.02	0.00	0.00	0.01	0.00	0.01	0.00	0.00	0.05
**GT22**	0.00	0.00	0.00	0.00	0.00	0.00	0.00	0.00	0.00	0.00	0.00	0.00	0.00
**PL12**	0.17	0.03	0.00	0.02	0.03	0.04	0.06	0.11	0.11	0.11	0.08	0.13	0.07
**PL18**	0.00	0.00	0.00	0.00	0.00	0.00	0.00	0.00	0.00	0.00	0.00	0.00	0.00
**GT92**	0.00	0.00	0.00	0.00	0.00	0.00	0.00	0.00	0.00	0.00	0.00	0.00	0.00
**GT44**	0.00	0.00	0.00	0.00	0.00	0.00	0.00	0.00	0.00	0.00	0.00	0.00	0.00
**GT29**	0.00	0.00	0.00	0.00	0.00	0.00	0.00	0.00	0.00	0.00	0.00	0.00	0.00
**GT13**	0.00	0.00	0.00	0.00	0.00	0.00	0.00	0.00	0.00	0.00	0.00	0.00	0.00

CAZY_group	DA-AD-50	DA-AD-51	DA-AD-52	DA-AD-53	DA-AD-54	DA-AD-55	DA-AD-56	DA-AD-57	DA-AD-58	DA-AD-59	DA-AD-6	DA-AD-60	DA-AD-61
**GT52**	0.00	0.00	0.00	0.00	0.00	0.00	0.00	0.00	0.00	0.00	0.00	0.00	0.00
**GT64**	0.00	0.00	0.00	0.00	0.00	0.00	0.00	0.00	0.00	0.00	0.00	0.00	0.00
**GT85**	0.00	0.00	0.00	0.00	0.00	0.00	0.00	0.00	0.00	0.00	0.00	0.00	0.00
**GT16**	0.00	0.00	0.00	0.00	0.00	0.00	0.00	0.00	0.00	0.00	0.00	0.00	0.00
**GH56**	0.00	0.00	0.00	0.00	0.00	0.00	0.00	0.00	0.00	0.00	0.00	0.00	0.00
**GT60**	0.00	0.00	0.00	0.00	0.00	0.00	0.00	0.00	0.00	0.00	0.00	0.00	0.00
**GH41**	0.00	0.00	0.00	0.00	0.00	0.00	0.00	0.00	0.00	0.00	0.00	0.00	0.00
**CE7**	0.13	0.06	0.05	0.02	0.06	0.05	0.14	0.12	0.06	0.06	0.05	0.08	0.14
**GH39**	0.11	0.04	0.04	0.03	0.05	0.05	0.00	0.05	0.02	0.03	0.05	0.07	0.01
**GT30**	0.28	0.23	0.17	0.20	0.17	0.27	0.34	0.21	0.21	0.22	0.17	0.21	0.19
**PL15**	0.11	0.03	0.00	0.04	0.03	0.05	0.10	0.10	0.09	0.06	0.03	0.18	0.08
**GH102**	0.02	0.03	0.00	0.01	0.03	0.01	0.00	0.03	0.01	0.01	0.07	0.00	0.02

CAZY_group	DA-AD-62	DA-AD-63	DA-AD-64	DA-AD-65	DA-AD-66	DA-AD-67	DA-AD-68	DA-AD-69	DA-AD-7	DA-AD-70	DA-AD-71	DA-AD-72	DA-AD-73
GH29	0.51	0.59	0.45	0.71	0.72	0.40	0.58	0.66	0.66	0.74	0.57	0.97	0.25
PL4	0.00	0.00	0.00	0.00	0.00	0.00	0.00	0.00	0.01	0.00	0.00	0.00	0.00
GH23	0.74	1.03	0.83	0.94	0.94	1.07	0.91	0.90	0.91	0.89	0.83	1.17	0.77
CE6	0.01	0.01	0.03	0.02	0.01	0.00	0.00	0.01	0.02	0.03	0.07	0.05	0.01
GH72	0.05	0.06	0.11	0.07	0.05	0.10	0.13	0.15	0.09	0.08	0.15	0.14	0.08
GH114	0.00	0.00	0.00	0.00	0.00	0.00	0.00	0.00	0.00	0.00	0.00	0.00	0.00
GH78	0.50	0.81	0.38	0.63	0.59	0.35	0.44	0.56	0.86	0.58	0.78	0.83	0.40
PL19	0.00	0.00	0.00	0.00	0.00	0.00	0.00	0.00	0.00	0.00	0.00	0.00	0.00
GT27	0.00	0.00	0.00	0.00	0.00	0.00	0.00	0.00	0.00	0.00	0.00	0.00	0.00
GH5	0.52	0.63	0.39	0.37	0.26	0.30	0.54	0.53	0.49	0.56	0.61	0.34	0.25
GT40	0.00	0.00	0.00	0.00	0.00	0.00	0.00	0.00	0.00	0.00	0.00	0.00	0.00
CE13	0.00	0.00	0.00	0.00	0.00	0.00	0.00	0.00	0.00	0.00	0.00	0.00	0.00
GT37	0.00	0.00	0.00	0.00	0.00	0.00	0.00	0.00	0.00	0.00	0.00	0.00	0.00
GH33	0.10	0.22	0.09	0.20	0.15	0.09	0.08	0.13	0.18	0.21	0.28	0.27	0.11
GT65	0.00	0.00	0.00	0.00	0.00	0.00	0.00	0.00	0.00	0.00	0.00	0.00	0.00
GH86	0.00	0.02	0.00	0.01	0.00	0.00	0.00	0.00	0.00	0.01	0.00	0.00	0.00
GH123	0.07	0.12	0.03	0.11	0.07	0.07	0.06	0.06	0.10	0.08	0.17	0.12	0.06
GH96	0.00	0.00	0.00	0.00	0.00	0.00	0.00	0.00	0.00	0.00	0.00	0.00	0.00
GH14	0.00	0.00	0.00	0.00	0.00	0.00	0.00	0.00	0.00	0.00	0.00	0.00	0.00
CE3	0.00	0.00	0.00	0.00	0.00	0.00	0.00	0.00	0.00	0.00	0.00	0.00	0.00
PL10	0.06	0.04	0.01	0.11	0.10	0.01	0.03	0.13	0.14	0.02	0.07	0.11	0.08
GT48	0.00	0.00	0.00	0.00	0.00	0.00	0.00	0.00	0.00	0.00	0.00	0.00	0.00
PL6	0.00	0.00	0.00	0.01	0.00	0.00	0.00	0.00	0.00	0.00	0.01	0.02	0.00
GT83	0.05	0.07	0.03	0.09	0.11	0.07	0.06	0.06	0.12	0.06	0.15	0.08	0.10
GH126	0.00	0.00	0.00	0.00	0.00	0.00	0.00	0.00	0.00	0.00	0.00	0.00	0.00
GH9	0.13	0.36	0.10	0.18	0.14	0.05	0.36	0.32	0.15	0.12	0.07	0.09	0.06
GH75	0.00	0.00	0.00	0.00	0.00	0.00	0.00	0.00	0.00	0.00	0.00	0.00	0.00
GT71	0.00	0.00	0.00	0.00	0.00	0.00	0.00	0.00	0.00	0.00	0.00	0.00	0.00
GH46	0.00	0.00	0.00	0.00	0.00	0.00	0.00	0.00	0.00	0.00	0.00	0.00	0.00
GT61	0.00	0.00	0.00	0.00	0.00	0.00	0.00	0.00	0.00	0.00	0.00	0.00	0.00
GT3	0.07	0.11	0.10	0.19	0.16	0.17	0.17	0.11	0.22	0.09	0.12	0.14	0.12
PL3	0.00	0.00	0.00	0.00	0.00	0.00	0.00	0.01	0.00	0.00	0.00	0.01	0.00
GH108	0.01	0.02	0.01	0.02	0.05	0.02	0.00	0.05	0.03	0.00	0.08	0.02	0.02
GT56	0.00	0.00	0.00	0.00	0.00	0.00	0.00	0.00	0.00	0.00	0.00	0.00	0.00
PL11	0.02	0.12	0.08	0.09	0.03	0.01	0.09	0.20	0.10	0.07	0.21	0.14	0.04
GH45	0.00	0.00	0.00	0.00	0.00	0.00	0.01	0.00	0.00	0.00	0.00	0.00	0.00
GT34	0.00	0.00	0.00	0.00	0.00	0.00	0.01	0.00	0.00	0.00	0.00	0.00	0.00
PL14	0.00	0.00	0.00	0.00	0.00	0.00	0.00	0.00	0.00	0.00	0.00	0.00	0.00
GH19	0.00	0.01	0.00	0.00	0.00	0.00	0.01	0.00	0.01	0.00	0.00	0.02	0.01
GH118	0.00	0.00	0.00	0.00	0.00	0.00	0.00	0.00	0.00	0.00	0.00	0.00	0.00
GH82	0.00	0.00	0.00	0.00	0.00	0.00	0.00	0.00	0.00	0.00	0.00	0.00	0.00
GH76	0.04	0.20	0.03	0.17	0.09	0.09	0.08	0.13	0.21	0.03	0.28	0.28	0.04
GT4	1.13	1.88	1.25	1.56	1.39	1.77	1.65	1.70	1.83	1.59	2.36	2.03	1.12
GT87	0.01	0.00	0.00	0.00	0.00	0.00	0.01	0.01	0.00	0.01	0.01	0.01	0.00
GH24	0.05	0.08	0.06	0.11	0.08	0.14	0.03	0.09	0.12	0.09	0.11	0.06	0.09
GH90	0.00	0.00	0.00	0.00	0.00	0.00	0.00	0.00	0.00	0.00	0.00	0.00	0.00
GH57	0.11	0.14	0.17	0.11	0.13	0.07	0.11	0.17	0.11	0.10	0.07	0.09	0.08
GH36	0.60	0.65	0.63	0.59	0.49	0.60	0.71	0.74	0.83	0.74	0.96	1.06	0.43
GT46	0.00	0.00	0.00	0.00	0.00	0.00	0.00	0.00	0.00	0.00	0.00	0.00	0.00
GH66	0.00	0.04	0.02	0.03	0.02	0.00	0.04	0.03	0.04	0.01	0.07	0.04	0.00

CAZY_group	DA-AD-62	DA-AD-63	DA-AD-64	DA-AD-65	DA-AD-66	DA-AD-67	DA-AD-68	DA-AD-69	DA-AD-7	DA-AD-70	DA-AD-71	DA-AD-72	DA-AD-73
**GH48**	0.00	0.00	0.00	0.00	0.00	0.00	0.01	0.00	0.00	0.02	0.00	0.00	0.00
**GT53**	0.00	0.00	0.00	0.00	0.00	0.00	0.00	0.00	0.00	0.00	0.00	0.00	0.00
**GH60**	0.00	0.00	0.00	0.00	0.00	0.00	0.00	0.00	0.00	0.00	0.00	0.00	0.00
**GT31**	0.00	0.00	0.00	0.00	0.00	0.00	0.00	0.00	0.00	0.00	0.00	0.00	0.00
**GH105**	0.26	0.46	0.27	0.48	0.44	0.17	0.26	0.52	0.45	0.23	0.60	0.74	0.38
**GT23**	0.02	0.04	0.00	0.00	0.01	0.01	0.00	0.00	0.01	0.00	0.01	0.02	0.00
**GH11**	0.00	0.01	0.00	0.00	0.00	0.00	0.03	0.00	0.00	0.00	0.01	0.00	0.00
**GT38**	0.00	0.00	0.00	0.00	0.00	0.00	0.00	0.00	0.00	0.00	0.00	0.00	0.00
**GT43**	0.00	0.00	0.00	0.00	0.00	0.00	0.00	0.00	0.00	0.00	0.00	0.00	0.00
**GH99**	0.02	0.00	0.00	0.01	0.00	0.00	0.00	0.00	0.00	0.00	0.03	0.01	0.00
**GH38**	0.27	0.37	0.06	0.21	0.23	0.21	0.23	0.18	0.36	0.27	0.28	0.21	0.12
**GH109**	0.10	0.28	0.16	0.24	0.27	0.11	0.11	0.24	0.22	0.15	0.24	0.38	0.12
**GH2**	2.79	3.27	2.33	3.68	3.29	2.68	3.02	3.48	3.72	2.79	5.06	4.53	2.28
**GT28**	0.41	0.40	0.32	0.37	0.30	0.47	0.39	0.40	0.41	0.41	0.33	0.47	0.35
**GH42**	0.26	0.19	0.13	0.16	0.14	0.21	0.24	0.11	0.21	0.26	0.24	0.24	0.15
**GT58**	0.00	0.00	0.00	0.00	0.00	0.00	0.00	0.00	0.00	0.00	0.00	0.00	0.00
**GH61**	0.00	0.00	0.00	0.00	0.00	0.00	0.00	0.00	0.00	0.00	0.00	0.00	0.00
**GH80**	0.00	0.00	0.00	0.00	0.00	0.00	0.00	0.00	0.00	0.00	0.00	0.00	0.00
**GH98**	0.00	0.02	0.00	0.00	0.00	0.00	0.01	0.02	0.00	0.02	0.06	0.01	0.00
**GH6**	0.00	0.00	0.00	0.01	0.00	0.00	0.00	0.00	0.00	0.00	0.00	0.00	0.00
**GT1**	0.04	0.06	0.03	0.01	0.03	0.02	0.05	0.08	0.03	0.02	0.08	0.04	0.04
**GH28**	0.41	0.79	0.34	0.62	0.52	0.38	0.43	0.76	0.69	0.27	1.05	0.97	0.50
**GT42**	0.00	0.00	0.00	0.00	0.00	0.00	0.00	0.00	0.01	0.00	0.00	0.00	0.00
**GH128**	0.00	0.00	0.00	0.00	0.00	0.00	0.00	0.00	0.00	0.00	0.00	0.00	0.00
**CE12**	0.15	0.17	0.07	0.09	0.07	0.01	0.10	0.21	0.10	0.06	0.21	0.25	0.04
**CE16**	0.00	0.00	0.00	0.00	0.00	0.00	0.00	0.00	0.00	0.00	0.00	0.00	0.00
**GT89**	0.00	0.00	0.00	0.00	0.00	0.00	0.00	0.00	0.00	0.00	0.00	0.00	0.00
**GT11**	0.05	0.06	0.05	0.03	0.05	0.05	0.04	0.08	0.06	0.04	0.08	0.02	0.00
**GT77**	0.00	0.00	0.00	0.00	0.00	0.00	0.00	0.01	0.00	0.01	0.00	0.00	0.00
**GT55**	0.00	0.00	0.00	0.00	0.00	0.00	0.00	0.00	0.00	0.00	0.00	0.00	0.00
**GT18**	0.00	0.00	0.00	0.00	0.00	0.00	0.00	0.00	0.00	0.00	0.00	0.00	0.00
**GH87**	0.00	0.00	0.00	0.00	0.00	0.00	0.00	0.00	0.04	0.00	0.00	0.00	0.00
**PL5**	0.00	0.00	0.00	0.00	0.00	0.00	0.00	0.00	0.00	0.00	0.00	0.00	0.00
**GH22**	0.00	0.00	0.01	0.00	0.00	0.00	0.00	0.00	0.00	0.00	0.00	0.00	0.00
**CE2**	0.06	0.10	0.09	0.09	0.04	0.04	0.13	0.05	0.10	0.08	0.07	0.04	0.04
**GH120**	0.05	0.06	0.06	0.03	0.03	0.07	0.08	0.05	0.04	0.04	0.08	0.05	0.05
**GH73**	0.37	0.49	0.46	0.37	0.53	0.53	0.43	0.46	0.60	0.50	0.57	0.63	0.44
**GT17**	0.00	0.00	0.00	0.00	0.00	0.01	0.00	0.02	0.00	0.00	0.00	0.00	0.00
**GH124**	0.00	0.00	0.00	0.00	0.00	0.00	0.00	0.00	0.00	0.00	0.00	0.00	0.00
**GH54**	0.00	0.00	0.00	0.00	0.00	0.00	0.00	0.00	0.00	0.00	0.00	0.00	0.00
**GH100**	0.00	0.00	0.00	0.00	0.00	0.00	0.00	0.00	0.00	0.00	0.00	0.00	0.00
**GH113**	0.04	0.02	0.08	0.00	0.05	0.00	0.04	0.05	0.03	0.02	0.06	0.04	0.04
**GT5**	0.49	0.46	0.44	0.34	0.42	0.50	0.39	0.41	0.34	0.37	0.42	0.43	0.40
**GH94**	0.46	0.60	0.46	0.23	0.41	0.33	0.63	0.56	0.35	0.41	0.48	0.43	0.30
**GT66**	0.00	0.00	0.02	0.00	0.00	0.01	0.02	0.00	0.00	0.00	0.00	0.00	0.00
**GT94**	0.00	0.00	0.00	0.00	0.00	0.00	0.00	0.00	0.00	0.00	0.00	0.00	0.00
**GH58**	0.00	0.00	0.00	0.00	0.00	0.00	0.00	0.00	0.00	0.00	0.00	0.00	0.00
**GH1**	0.39	0.53	0.56	0.12	0.31	0.66	0.39	0.35	0.51	0.41	0.79	0.85	0.49
**PL22**	0.00	0.00	0.00	0.00	0.00	0.00	0.00	0.00	0.00	0.00	0.00	0.00	0.00

CAZY_group	DA-AD-62	DA-AD-63	DA-AD-64	DA-AD-65	DA-AD-66	DA-AD-67	DA-AD-68	DA-AD-69	DA-AD-7	DA-AD-70	DA-AD-71	DA-AD-72	DA-AD-73
**GT76**	0.00	0.00	0.00	0.00	0.00	0.00	0.00	0.00	0.00	0.00	0.00	0.00	0.00
**GT32**	0.02	0.12	0.08	0.04	0.08	0.02	0.10	0.13	0.09	0.10	0.14	0.13	0.05
**GT47**	0.02	0.06	0.03	0.02	0.01	0.01	0.04	0.02	0.03	0.07	0.07	0.02	0.01
**GH84**	0.10	0.06	0.08	0.11	0.10	0.09	0.11	0.14	0.09	0.10	0.11	0.19	0.05
**GH83**	0.00	0.00	0.00	0.00	0.00	0.00	0.00	0.00	0.00	0.00	0.00	0.00	0.00
**GT59**	0.00	0.00	0.00	0.00	0.00	0.00	0.00	0.00	0.00	0.00	0.00	0.00	0.00
**GH34**	0.00	0.00	0.00	0.00	0.00	0.00	0.00	0.00	0.00	0.00	0.00	0.00	0.00
**GT14**	0.05	0.02	0.00	0.03	0.03	0.01	0.02	0.04	0.02	0.00	0.01	0.07	0.00
**GH47**	0.00	0.00	0.00	0.00	0.00	0.00	0.00	0.00	0.00	0.00	0.00	0.00	0.00
**GH97**	0.49	0.54	0.34	0.94	0.60	0.43	0.59	0.67	0.78	0.30	0.80	0.98	0.32
**GH50**	0.00	0.05	0.00	0.03	0.00	0.00	0.00	0.03	0.03	0.00	0.08	0.08	0.01
**GT26**	0.13	0.19	0.16	0.10	0.16	0.17	0.16	0.15	0.16	0.18	0.21	0.22	0.09
**GH18**	0.34	0.42	0.38	0.57	0.34	0.52	0.54	0.59	0.49	0.49	0.86	0.69	0.53
**GH37**	0.00	0.00	0.00	0.02	0.00	0.00	0.00	0.00	0.00	0.00	0.00	0.00	0.00
**PL13**	0.02	0.02	0.00	0.03	0.03	0.02	0.01	0.03	0.03	0.00	0.07	0.05	0.02
**GH27**	0.16	0.18	0.18	0.26	0.18	0.21	0.22	0.24	0.24	0.19	0.21	0.26	0.14
**GT54**	0.00	0.00	0.00	0.00	0.00	0.00	0.00	0.00	0.00	0.00	0.00	0.00	0.00
**GT91**	0.00	0.00	0.00	0.00	0.00	0.00	0.00	0.00	0.00	0.00	0.00	0.00	0.00
**GT72**	0.00	0.00	0.00	0.00	0.00	0.00	0.00	0.00	0.00	0.00	0.00	0.00	0.00
**GH67**	0.02	0.05	0.05	0.04	0.03	0.00	0.06	0.06	0.07	0.04	0.10	0.08	0.02
**GH12**	0.00	0.00	0.00	0.00	0.00	0.00	0.00	0.00	0.00	0.00	0.00	0.00	0.00
**GH91**	0.02	0.00	0.01	0.03	0.05	0.04	0.02	0.02	0.02	0.01	0.03	0.01	0.01
**GT69**	0.00	0.00	0.00	0.00	0.00	0.00	0.00	0.00	0.00	0.00	0.00	0.00	0.00
**GH106**	0.17	0.25	0.08	0.21	0.13	0.04	0.09	0.26	0.14	0.08	0.30	0.26	0.04
**GT62**	0.00	0.00	0.00	0.00	0.00	0.00	0.00	0.00	0.00	0.00	0.00	0.00	0.00
**GH17**	0.00	0.00	0.00	0.00	0.00	0.00	0.00	0.00	0.00	0.00	0.00	0.00	0.00
**GH88**	0.18	0.19	0.08	0.35	0.31	0.12	0.19	0.28	0.31	0.21	0.32	0.47	0.21
**CE5**	0.00	0.00	0.00	0.00	0.00	0.00	0.00	0.00	0.00	0.00	0.00	0.00	0.00
**GT8**	0.22	0.06	0.05	0.16	0.01	0.06	0.04	0.09	0.24	0.12	0.06	0.14	0.05
**GT50**	0.00	0.00	0.00	0.00	0.00	0.00	0.00	0.00	0.00	0.00	0.00	0.00	0.00
**GT20**	0.04	0.05	0.05	0.10	0.07	0.04	0.04	0.11	0.04	0.07	0.06	0.08	0.03
**PL9**	0.02	0.11	0.04	0.06	0.01	0.02	0.10	0.07	0.06	0.08	0.08	0.06	0.00
**GH55**	0.01	0.02	0.02	0.01	0.05	0.04	0.01	0.02	0.02	0.01	0.06	0.02	0.03
**GH64**	0.00	0.00	0.00	0.00	0.00	0.00	0.00	0.00	0.00	0.00	0.00	0.00	0.00
**GH127**	0.17	0.26	0.16	0.34	0.29	0.27	0.33	0.18	0.45	0.20	0.28	0.37	0.19
**GH103**	0.01	0.00	0.00	0.00	0.00	0.00	0.00	0.00	0.00	0.01	0.01	0.01	0.00
**GH3**	1.87	2.45	1.54	2.10	2.34	2.02	2.16	2.04	2.61	2.17	3.05	3.16	1.94
**GT45**	0.00	0.00	0.00	0.00	0.00	0.00	0.00	0.00	0.00	0.00	0.00	0.00	0.00
**GT74**	0.00	0.00	0.00	0.00	0.00	0.00	0.00	0.00	0.00	0.00	0.00	0.00	0.00
**GT90**	0.00	0.00	0.00	0.00	0.00	0.00	0.00	0.00	0.00	0.00	0.00	0.01	0.00
**GH116**	0.07	0.05	0.02	0.10	0.08	0.04	0.06	0.03	0.04	0.00	0.07	0.07	0.03
**GH31**	0.89	0.78	0.73	0.80	0.77	0.79	0.99	0.96	1.04	0.92	1.16	1.10	0.75
**GT12**	0.00	0.00	0.00	0.00	0.00	0.00	0.00	0.00	0.00	0.00	0.00	0.00	0.00
**CE1**	0.26	0.38	0.23	0.38	0.26	0.27	0.32	0.39	0.48	0.31	0.44	0.47	0.28
**GH121**	0.00	0.00	0.00	0.06	0.00	0.01	0.00	0.00	0.00	0.00	0.00	0.00	0.00
**GH79**	0.00	0.01	0.00	0.00	0.00	0.00	0.00	0.00	0.00	0.01	0.00	0.01	0.00
**GT73**	0.00	0.00	0.00	0.00	0.00	0.00	0.00	0.00	0.00	0.00	0.00	0.00	0.00
**GH77**	0.60	0.65	0.55	0.47	0.52	0.63	0.55	0.53	0.55	0.55	0.65	0.72	0.45
**PL17**	0.00	0.00	0.01	0.03	0.00	0.00	0.00	0.03	0.01	0.00	0.00	0.02	0.02
**PL8**	0.06	0.13	0.10	0.42	0.21	0.07	0.18	0.19	0.22	0.21	0.32	0.45	0.16

CAZY_group	DA-AD-62	DA-AD-63	DA-AD-64	DA-AD-65	DA-AD-66	DA-AD-67	DA-AD-68	DA-AD-69	DA-AD-7	DA-AD-70	DA-AD-71	DA-AD-72	DA-AD-73
**CE9**	0.22	0.30	0.20	0.19	0.27	0.33	0.25	0.30	0.32	0.27	0.46	0.43	0.23
**CE15**	0.00	0.00	0.00	0.02	0.00	0.01	0.03	0.01	0.03	0.01	0.01	0.01	0.03
**GT79**	0.00	0.00	0.00	0.00	0.00	0.00	0.00	0.00	0.00	0.00	0.00	0.00	0.00
**GT10**	0.00	0.00	0.02	0.00	0.01	0.00	0.00	0.00	0.00	0.02	0.03	0.00	0.00
**GH119**	0.00	0.00	0.00	0.00	0.00	0.00	0.00	0.00	0.00	0.00	0.00	0.00	0.00
**GH16**	0.09	0.16	0.13	0.21	0.20	0.14	0.17	0.19	0.20	0.19	0.26	0.19	0.08
**GH43**	1.46	1.62	1.09	2.04	1.54	0.93	1.75	2.00	2.23	1.21	2.29	2.77	0.99
**GH51**	0.37	0.52	0.45	0.48	0.54	0.47	0.65	0.44	0.58	0.56	0.51	0.58	0.33
**GT35**	0.94	0.96	0.93	0.79	0.67	0.74	0.86	0.86	0.74	0.86	0.85	0.84	0.79
**GT25**	0.00	0.00	0.00	0.00	0.00	0.00	0.00	0.00	0.00	0.00	0.06	0.04	0.00
**GT2**	3.70	4.49	3.55	3.38	3.42	4.11	3.60	3.85	4.09	3.42	5.22	4.80	3.53
**GT51**	0.71	0.76	0.59	0.82	0.70	0.83	0.76	0.79	0.95	0.71	0.86	1.11	0.72
**GH107**	0.00	0.00	0.00	0.00	0.00	0.00	0.00	0.00	0.00	0.00	0.00	0.00	0.00
**GH7**	0.00	0.00	0.00	0.00	0.00	0.00	0.00	0.00	0.00	0.00	0.00	0.00	0.00
**GT7**	0.00	0.00	0.00	0.00	0.00	0.00	0.00	0.00	0.00	0.00	0.00	0.00	0.00
**GT36**	0.00	0.00	0.00	0.00	0.00	0.00	0.00	0.00	0.00	0.00	0.00	0.00	0.00
**PL7**	0.00	0.00	0.00	0.00	0.00	0.00	0.00	0.00	0.00	0.00	0.00	0.00	0.00
**GH110**	0.04	0.07	0.08	0.12	0.11	0.02	0.10	0.11	0.09	0.15	0.10	0.12	0.03
**GT21**	0.00	0.00	0.00	0.00	0.00	0.00	0.00	0.00	0.00	0.00	0.00	0.00	0.00
**GH62**	0.00	0.00	0.00	0.00	0.00	0.00	0.00	0.00	0.00	0.00	0.00	0.00	0.00
**GH125**	0.15	0.11	0.09	0.13	0.17	0.16	0.14	0.11	0.17	0.15	0.22	0.18	0.10
**GH117**	0.01	0.07	0.01	0.06	0.08	0.01	0.04	0.05	0.11	0.02	0.07	0.09	0.06
**GH68**	0.00	0.00	0.00	0.00	0.00	0.00	0.00	0.00	0.02	0.00	0.00	0.00	0.00
**GH85**	0.02	0.07	0.00	0.00	0.01	0.02	0.01	0.01	0.06	0.00	0.07	0.02	0.00
**GH92**	0.93	1.15	0.64	1.44	1.36	0.79	1.11	1.55	1.55	0.74	2.04	1.94	0.65
**GH40**	0.00	0.00	0.00	0.00	0.00	0.00	0.00	0.00	0.00	0.00	0.00	0.00	0.00
**GT49**	0.00	0.00	0.00	0.00	0.00	0.00	0.00	0.00	0.00	0.00	0.00	0.00	0.00
**GH112**	0.12	0.18	0.08	0.04	0.15	0.20	0.10	0.13	0.17	0.18	0.19	0.22	0.21
**GT67**	0.00	0.00	0.00	0.00	0.00	0.00	0.00	0.00	0.00	0.00	0.00	0.00	0.00
**CE8**	0.13	0.18	0.08	0.21	0.18	0.12	0.14	0.26	0.22	0.09	0.21	0.32	0.12
**GT86**	0.00	0.00	0.00	0.00	0.00	0.00	0.00	0.00	0.00	0.00	0.00	0.00	0.00
**GH101**	0.00	0.00	0.00	0.00	0.00	0.01	0.01	0.00	0.00	0.00	0.00	0.02	0.01
**GH81**	0.00	0.00	0.00	0.00	0.02	0.00	0.00	0.00	0.00	0.00	0.01	0.01	0.00
**GT93**	0.00	0.00	0.00	0.00	0.00	0.00	0.00	0.00	0.00	0.00	0.00	0.00	0.00
**GT63**	0.00	0.00	0.00	0.00	0.00	0.00	0.00	0.00	0.00	0.00	0.00	0.00	0.00
**CE11**	0.18	0.18	0.16	0.17	0.20	0.21	0.16	0.21	0.22	0.22	0.22	0.26	0.16
**GT19**	0.11	0.12	0.10	0.12	0.16	0.10	0.11	0.14	0.16	0.10	0.15	0.19	0.06
**GT33**	0.00	0.00	0.00	0.00	0.00	0.00	0.00	0.00	0.00	0.00	0.00	0.00	0.00
**GH21**	0.00	0.00	0.00	0.00	0.00	0.00	0.00	0.00	0.00	0.00	0.00	0.00	0.00
**GT68**	0.00	0.00	0.00	0.00	0.00	0.00	0.00	0.00	0.00	0.00	0.00	0.00	0.00
**GH89**	0.13	0.26	0.13	0.27	0.16	0.05	0.13	0.23	0.16	0.16	0.28	0.26	0.08
**GT82**	0.00	0.00	0.00	0.00	0.00	0.00	0.00	0.00	0.00	0.01	0.00	0.00	0.00
**GH30**	0.10	0.10	0.08	0.22	0.13	0.14	0.18	0.17	0.22	0.13	0.29	0.20	0.13
**GH20**	0.52	1.00	0.55	1.29	0.92	0.61	0.73	0.97	1.10	0.83	1.29	1.25	0.46
**GH52**	0.00	0.00	0.00	0.00	0.00	0.00	0.00	0.00	0.00	0.00	0.00	0.00	0.00
**GT84**	0.04	0.06	0.04	0.00	0.01	0.02	0.04	0.05	0.01	0.01	0.00	0.00	0.00
**GT15**	0.00	0.00	0.00	0.00	0.00	0.00	0.00	0.00	0.00	0.00	0.00	0.00	0.00
**GH93**	0.00	0.00	0.01	0.01	0.00	0.00	0.00	0.00	0.00	0.01	0.01	0.00	0.00
**PL16**	0.00	0.00	0.00	0.00	0.00	0.00	0.00	0.00	0.00	0.00	0.00	0.00	0.00
**GH26**	0.12	0.18	0.12	0.19	0.10	0.15	0.19	0.13	0.22	0.23	0.21	0.05	0.04

CAZY_group	DA-AD-62	DA-AD-63	DA-AD-64	DA-AD-65	DA-AD-66	DA-AD-67	DA-AD-68	DA-AD-69	DA-AD-7	DA-AD-70	DA-AD-71	DA-AD-72	DA-AD-73	
GT75	0.00	0.00	0.00	0.00	0.00	0.00	0.00	0.00	0.00	0.00	0.00	0.00	0.00	
GH53	0.19	0.29	0.12	0.08	0.10	0.09	0.17	0.09	0.11	0.04	0.17	0.09	0.06	
GT88	0.00	0.00	0.00	0.00	0.00	0.00	0.00	0.00	0.00	0.00	0.00	0.00	0.00	
GT24	0.00	0.00	0.01	0.00	0.00	0.00	0.00	0.00	0.00	0.00	0.00	0.00	0.00	
GT81	0.00	0.00	0.00	0.00	0.00	0.00	0.00	0.00	0.00	0.00	0.00	0.00	0.00	
GH4	0.07	0.19	0.13	0.02	0.07	0.19	0.13	0.05	0.09	0.18	0.17	0.22	0.23	
GT78	0.00	0.00	0.00	0.00	0.00	0.00	0.00	0.00	0.00	0.00	0.00	0.00	0.00	
GH8	0.06	0.08	0.06	0.07	0.09	0.05	0.08	0.06	0.11	0.02	0.10	0.07	0.08	
GH70	0.00	0.00	0.00	0.00	0.00	0.00	0.01	0.00	0.22	0.00	0.01	0.00	0.00	
GH122	0.00	0.00	0.00	0.00	0.00	0.00	0.00	0.00	0.00	0.00	0.00	0.00	0.00	
GH74	0.01	0.04	0.05	0.02	0.02	0.05	0.06	0.03	0.03	0.08	0.06	0.02	0.02	
GT41	0.01	0.00	0.02	0.00	0.00	0.02	0.00	0.01	0.00	0.00	0.00	0.02	0.00	
PL1	0.21	0.23	0.07	0.27	0.16	0.14	0.18	0.22	0.18	0.13	0.43	0.24	0.10	
GH15	0.05	0.07	0.07	0.08	0.01	0.01	0.04	0.04	0.04	0.03	0.08	0.05	0.01	
GH32	0.41	0.29	0.35	0.20	0.33	0.33	0.44	0.36	0.46	0.42	0.64	0.60	0.33	
GH44	0.00	0.00	0.01	0.00	0.00	0.00	0.01	0.00	0.00	0.00	0.00	0.00	0.00	
GT6	0.00	0.01	0.00	0.00	0.00	0.00	0.00	0.00	0.00	0.00	0.00	0.00	0.00	
GH13	2.63	3.12	2.52	2.51	2.48	3.10	3.06	2.72	2.87	3.19	3.01	3.38	2.53	
GT80	0.00	0.01	0.01	0.02	0.03	0.05	0.04	0.00	0.03	0.01	0.00	0.00	0.01	
GH71	0.00	0.00	0.00	0.00	0.00	0.00	0.00	0.00	0.00	0.00	0.00	0.00	0.00	
GH63	0.10	0.08	0.04	0.08	0.09	0.07	0.09	0.09	0.07	0.01	0.06	0.09	0.03	
PL21	0.00	0.01	0.00	0.02	0.00	0.00	0.00	0.02	0.01	0.02	0.01	0.04	0.00	
GH65	0.15	0.11	0.07	0.14	0.14	0.07	0.14	0.11	0.13	0.19	0.12	0.12	0.13	
CE4	0.54	0.67	0.45	0.51	0.51	0.50	0.56	0.65	0.56	0.59	0.69	0.64	0.44	
CE14	0.04	0.02	0.02	0.02	0.01	0.02	0.02	0.01	0.00	0.01	0.00	0.00	0.00	
GH115	0.15	0.22	0.11	0.21	0.22	0.16	0.20	0.22	0.27	0.08	0.40	0.38	0.21	
GH104	0.00	0.00	0.00	0.00	0.00	0.00	0.00	0.00	0.00	0.00	0.00	0.00	0.00	
GH111	0.00	0.00	0.00	0.00	0.00	0.00	0.00	0.00	0.00	0.00	0.00	0.00	0.00	
GH95	0.34	0.40	0.23	0.42	0.44	0.19	0.36	0.56	0.51	0.39	0.60	0.76	0.21	
GT70	0.00	0.00	0.00	0.00	0.00	0.00	0.00	0.00	0.00	0.00	0.00	0.00	0.00	
CE10	0.07	0.14	0.07	0.17	0.17	0.14	0.14	0.15	0.15	0.06	0.21	0.11	0.12	
GH10	0.13	0.13	0.10	0.10	0.07	0.05	0.11	0.12	0.09	0.07	0.21	0.17	0.04	
GH130	0.30	0.41	0.29	0.32	0.26	0.20	0.31	0.33	0.37	0.22	0.53	0.34	0.15	
PL20	0.00	0.00	0.00	0.00	0.00	0.00	0.00	0.00	0.00	0.00	0.00	0.00	0.00	
GH69	0.00	0.00	0.00	0.00	0.00	0.00	0.00	0.00	0.00	0.00	0.00	0.00	0.00	
PL2	0.00	0.00	0.00	0.00	0.00	0.00	0.00	0.00	0.00	0.00	0.00	0.00	0.00	
GH49	0.00	0.00	0.00	0.00	0.00	0.00	0.00	0.00	0.00	0.00	0.00	0.00	0.00	
GH25	0.58	0.48	0.41	0.35	0.36	0.38	0.48	0.31	0.41	0.40	0.43	0.70	0.35	
GH59	0.02	0.01	0.03	0.02	0.00	0.02	0.04	0.03	0.03	0.04	0.03	0.01	0.02	
GH35	0.22	0.28	0.14	0.34	0.34	0.19	0.25	0.30	0.28	0.20	0.40	0.45	0.16	
GT39	0.01	0.00	0.00	0.00	0.00	0.00	0.01	0.00	0.01	0.00	0.01	0.00	0.00	
GT57	0.00	0.00	0.00	0.00	0.00	0.00	0.00	0.00	0.00	0.00	0.00	0.00	0.00	
GH129	0.00	0.00	0.00	0.00	0.00	0.05	0.01	0.00	0.03	0.02	0.00	0.01	0.03	
GT22	0.00	0.00	0.00	0.00	0.00	0.00	0.00	0.00	0.00	0.00	0.00	0.00	0.00	
PL12	0.02	0.06	0.00	0.12	0.08	0.04	0.04	0.08	0.13	0.08	0.25	0.12	0.06	
PL18	0.00	0.00	0.00	0.00	0.00	0.00	0.00	0.00	0.00	0.00	0.00	0.00	0.00	
GT92	0.00	0.00	0.00	0.00	0.00	0.00	0.00	0.00	0.00	0.00	0.00	0.00	0.00	
GT44	0.00	0.00	0.00	0.00	0.00	0.00	0.00	0.00	0.00	0.00	0.00	0.00	0.00	
GT29	0.00	0.00	0.00	0.00	0.00	0.00	0.00	0.00	0.00	0.00	0.00	0.00	0.00	
GT13	0.00	0.00	0.00	0.00	0.00	0.00	0.00	0.00	0.00	0.00	0.00	0.00	0.00	

CAZY_group	DA-AD-62	DA-AD-63	DA-AD-64	DA-AD-65	DA-AD-66	DA-AD-67	DA-AD-68	DA-AD-69	DA-AD-7	DA-AD-70	DA-AD-71	DA-AD-72	DA-AD-73
**GT52**	0.00	0.00	0.00	0.00	0.00	0.00	0.00	0.00	0.00	0.00	0.00	0.00	0.00
**GT64**	0.00	0.00	0.00	0.00	0.00	0.00	0.00	0.00	0.00	0.00	0.00	0.00	0.00
**GT85**	0.00	0.00	0.00	0.00	0.00	0.00	0.00	0.00	0.00	0.00	0.00	0.00	0.00
**GT16**	0.00	0.00	0.00	0.00	0.00	0.00	0.00	0.00	0.00	0.00	0.00	0.00	0.00
**GH56**	0.00	0.00	0.00	0.00	0.00	0.00	0.00	0.00	0.00	0.00	0.00	0.00	0.00
**GT60**	0.00	0.00	0.00	0.00	0.00	0.00	0.00	0.00	0.00	0.00	0.00	0.00	0.00
**GH41**	0.00	0.00	0.00	0.00	0.00	0.00	0.00	0.00	0.00	0.00	0.00	0.00	0.00
**CE7**	0.11	0.07	0.03	0.04	0.07	0.02	0.02	0.07	0.08	0.02	0.17	0.14	0.03
**GH39**	0.02	0.04	0.02	0.02	0.05	0.05	0.07	0.04	0.03	0.03	0.07	0.05	0.02
**GT30**	0.18	0.12	0.14	0.21	0.23	0.15	0.18	0.25	0.22	0.19	0.12	0.27	0.11
**PL15**	0.05	0.04	0.02	0.16	0.07	0.01	0.04	0.08	0.05	0.03	0.14	0.17	0.04
**GH102**	0.01	0.00	0.02	0.01	0.01	0.00	0.02	0.02	0.01	0.00	0.03	0.02	0.01

CAZY_group	DA-AD-74	DA-AD-75	DA-AD-76	DA-AD-77	DA-AD-78	DA-AD-79	DA-AD-8	DA-AD-80	DA-AD-81	DA-AD-82	DA-AD-83	DA-AD-84	DA-AD-85
**GH29**	0.53	0.80	0.54	0.41	0.57	0.66	1.09	0.47	0.46	0.47	1.19	0.67	0.44
**PL4**	0.00	0.00	0.00	0.00	0.00	0.00	0.00	0.00	0.00	0.00	0.00	0.00	0.00
**GH23**	0.80	1.03	0.83	1.06	1.21	1.00	1.38	0.82	1.00	1.00	1.15	0.91	0.94
**CE6**	0.01	0.00	0.04	0.00	0.02	0.01	0.00	0.00	0.00	0.01	0.04	0.01	0.01
**GH72**	0.11	0.10	0.20	0.10	0.18	0.06	0.13	0.08	0.17	0.10	0.16	0.11	0.11
**GH114**	0.00	0.00	0.00	0.00	0.00	0.00	0.00	0.00	0.00	0.00	0.00	0.00	0.00
**GH78**	0.57	0.59	0.43	0.34	0.22	0.68	0.51	0.52	0.63	0.77	1.01	1.09	0.40
**PL19**	0.00	0.00	0.00	0.00	0.00	0.00	0.00	0.00	0.00	0.00	0.00	0.00	0.00
**GT27**	0.00	0.00	0.00	0.00	0.00	0.00	0.00	0.00	0.00	0.00	0.00	0.00	0.00
**GH5**	0.40	0.63	0.44	0.75	0.25	0.52	0.77	0.43	0.57	0.45	0.27	0.47	0.58
**GT40**	0.00	0.00	0.00	0.00	0.00	0.00	0.00	0.00	0.00	0.00	0.00	0.00	0.00
**CE13**	0.00	0.00	0.00	0.00	0.00	0.00	0.00	0.00	0.00	0.00	0.00	0.00	0.00
**GT37**	0.00	0.00	0.00	0.00	0.00	0.00	0.00	0.00	0.00	0.00	0.00	0.00	0.00
**GH33**	0.19	0.14	0.15	0.03	0.11	0.21	0.16	0.12	0.11	0.18	0.29	0.18	0.12
**GT65**	0.00	0.00	0.00	0.00	0.00	0.00	0.00	0.00	0.00	0.00	0.00	0.00	0.00
**GH86**	0.00	0.00	0.00	0.03	0.00	0.00	0.00	0.00	0.00	0.00	0.00	0.01	0.00
**GH123**	0.06	0.08	0.08	0.00	0.03	0.05	0.10	0.08	0.06	0.14	0.14	0.14	0.05
**GH96**	0.00	0.00	0.00	0.00	0.00	0.00	0.00	0.00	0.00	0.00	0.00	0.00	0.00
**GH14**	0.00	0.00	0.00	0.00	0.00	0.00	0.00	0.00	0.00	0.00	0.00	0.00	0.00
**CE3**	0.00	0.00	0.00	0.00	0.00	0.00	0.00	0.00	0.00	0.00	0.00	0.00	0.00
**PL10**	0.06	0.04	0.03	0.00	0.03	0.06	0.42	0.08	0.09	0.07	0.08	0.07	0.04
**GT48**	0.00	0.00	0.00	0.00	0.00	0.00	0.00	0.00	0.00	0.00	0.00	0.00	0.00
**PL6**	0.00	0.00	0.00	0.00	0.00	0.00	0.00	0.00	0.00	0.00	0.02	0.02	0.00
**GT83**	0.08	0.05	0.07	0.03	0.00	0.11	0.03	0.10	0.04	0.09	0.14	0.11	0.03
**GH126**	0.00	0.00	0.00	0.00	0.00	0.00	0.00	0.00	0.00	0.00	0.00	0.00	0.00
**GH9**	0.14	0.31	0.11	0.75	0.06	0.17	0.23	0.08	0.14	0.23	0.16	0.18	0.26
**GH75**	0.00	0.00	0.00	0.00	0.00	0.00	0.00	0.00	0.00	0.00	0.00	0.00	0.00
**GT71**	0.00	0.00	0.00	0.00	0.00	0.00	0.00	0.00	0.00	0.00	0.00	0.00	0.00
**GH46**	0.00	0.00	0.00	0.00	0.00	0.00	0.00	0.00	0.00	0.00	0.00	0.00	0.00
**GT61**	0.00	0.00	0.00	0.00	0.00	0.00	0.00	0.00	0.00	0.00	0.00	0.00	0.00
**GT3**	0.15	0.14	0.14	0.07	0.11	0.17	0.32	0.16	0.12	0.15	0.16	0.22	0.10
**PL3**	0.00	0.00	0.00	0.00	0.00	0.00	0.00	0.00	0.00	0.00	0.00	0.00	0.00
**GH108**	0.02	0.00	0.03	0.00	0.02	0.02	0.06	0.03	0.04	0.01	0.06	0.04	0.02
**GT56**	0.00	0.00	0.00	0.00	0.02	0.00	0.00	0.00	0.00	0.00	0.00	0.00	0.01
**PL11**	0.11	0.04	0.09	0.03	0.05	0.10	0.16	0.10	0.09	0.14	0.04	0.10	0.11
**GH45**	0.01	0.00	0.00	0.00	0.00	0.00	0.00	0.00	0.01	0.00	0.02	0.00	0.00
**GT34**	0.00	0.00	0.00	0.00	0.00	0.00	0.00	0.00	0.00	0.00	0.00	0.00	0.00
**PL14**	0.00	0.00	0.00	0.00	0.00	0.00	0.00	0.00	0.00	0.00	0.00	0.00	0.00
**GH19**	0.00	0.00	0.00	0.00	0.03	0.00	0.00	0.00	0.01	0.01	0.00	0.01	0.01
**GH118**	0.00	0.00	0.00	0.00	0.00	0.00	0.00	0.00	0.00	0.00	0.00	0.00	0.00
**GH82**	0.00	0.00	0.00	0.00	0.00	0.00	0.00	0.00	0.00	0.00	0.00	0.00	0.00
**GH76**	0.09	0.08	0.11	0.00	0.05	0.24	0.00	0.08	0.05	0.18	0.23	0.41	0.10
**GT4**	1.45	1.84	1.91	1.13	1.47	2.07	1.45	1.68	1.54	1.69	2.07	2.11	1.49
**GT87**	0.01	0.01	0.00	0.00	0.02	0.00	0.00	0.01	0.01	0.00	0.02	0.01	0.00
**GH24**	0.15	0.01	0.06	0.21	0.13	0.09	0.23	0.08	0.05	0.10	0.14	0.13	0.06
**GH90**	0.00	0.00	0.00	0.00	0.00	0.00	0.00	0.00	0.00	0.00	0.00	0.00	0.00
**GH57**	0.12	0.20	0.16	0.14	0.18	0.15	0.19	0.08	0.20	0.07	0.12	0.12	0.08
**GH36**	0.66	0.77	0.62	1.03	0.27	0.90	0.90	0.65	0.44	0.63	0.82	0.79	0.75
**GT46**	0.00	0.00	0.00	0.00	0.00	0.00	0.00	0.00	0.00	0.00	0.00	0.00	0.00
**GH66**	0.01	0.03	0.02	0.03	0.02	0.02	0.03	0.02	0.04	0.02	0.06	0.10	0.03

CAZY_group	DA-AD-74	DA-AD-75	DA-AD-76	DA-AD-77	DA-AD-78	DA-AD-79	DA-AD-8	DA-AD-80	DA-AD-81	DA-AD-82	DA-AD-83	DA-AD-84	DA-AD-85
GH48	0.00	0.00	0.00	0.00	0.00	0.00	0.00	0.00	0.00	0.00	0.00	0.00	0.01
GT53	0.00	0.00	0.00	0.00	0.00	0.00	0.00	0.00	0.00	0.00	0.00	0.00	0.00
GH60	0.00	0.00	0.00	0.00	0.00	0.00	0.00	0.00	0.00	0.00	0.00	0.00	0.00
GT31	0.00	0.00	0.00	0.00	0.00	0.00	0.00	0.00	0.00	0.00	0.00	0.00	0.00
GH105	0.44	0.41	0.30	0.03	0.22	0.42	0.51	0.38	0.56	0.29	0.51	0.56	0.24
GT23	0.00	0.03	0.00	0.00	0.00	0.02	0.03	0.02	0.00	0.00	0.02	0.01	0.01
GH11	0.02	0.00	0.00	0.00	0.00	0.03	0.00	0.00	0.01	0.01	0.00	0.00	0.01
GT38	0.00	0.00	0.00	0.00	0.00	0.00	0.00	0.00	0.00	0.00	0.00	0.00	0.00
GT43	0.00	0.00	0.00	0.00	0.00	0.00	0.00	0.00	0.00	0.00	0.00	0.00	0.00
GH99	0.00	0.00	0.00	0.00	0.00	0.02	0.00	0.00	0.00	0.00	0.00	0.02	0.00
GH38	0.13	0.22	0.15	0.17	0.22	0.23	0.23	0.30	0.18	0.17	0.27	0.36	0.25
GH109	0.25	0.27	0.11	0.24	0.18	0.27	0.39	0.26	0.21	0.19	0.41	0.24	0.09
GH2	2.88	3.25	3.20	2.43	1.75	3.57	4.47	2.90	3.37	3.33	4.72	4.82	2.78
GT28	0.36	0.43	0.49	0.65	0.24	0.41	0.55	0.42	0.38	0.50	0.41	0.41	0.47
GH42	0.12	0.19	0.23	0.17	0.10	0.24	0.26	0.19	0.12	0.19	0.10	0.14	0.37
GT58	0.00	0.00	0.00	0.00	0.00	0.00	0.00	0.00	0.00	0.00	0.00	0.00	0.00
GH61	0.00	0.00	0.00	0.00	0.00	0.00	0.00	0.00	0.00	0.00	0.00	0.00	0.00
GH80	0.00	0.00	0.00	0.00	0.00	0.00	0.00	0.00	0.00	0.00	0.00	0.00	0.00
GH98	0.01	0.00	0.01	0.00	0.00	0.00	0.00	0.00	0.01	0.00	0.00	0.01	0.01
GH6	0.00	0.01	0.00	0.00	0.00	0.00	0.00	0.00	0.00	0.00	0.00	0.00	0.00
GT1	0.03	0.05	0.03	0.00	0.03	0.03	0.00	0.05	0.02	0.06	0.02	0.05	0.04
GH28	0.63	0.82	0.56	0.38	0.57	0.51	1.09	0.60	0.74	0.73	0.78	0.77	0.47
GT42	0.00	0.00	0.00	0.00	0.00	0.00	0.00	0.00	0.00	0.00	0.00	0.00	0.00
GH128	0.00	0.00	0.00	0.00	0.00	0.00	0.00	0.00	0.00	0.00	0.00	0.00	0.01
CE12	0.06	0.14	0.11	0.10	0.11	0.10	0.10	0.10	0.19	0.12	0.10	0.10	0.07
CE16	0.00	0.00	0.00	0.00	0.00	0.00	0.00	0.00	0.00	0.00	0.00	0.00	0.00
GT89	0.00	0.00	0.00	0.00	0.00	0.00	0.00	0.00	0.00	0.00	0.00	0.00	0.00
GT11	0.05	0.04	0.07	0.03	0.00	0.08	0.06	0.09	0.05	0.07	0.16	0.05	0.05
GT77	0.00	0.00	0.00	0.00	0.00	0.00	0.00	0.00	0.00	0.00	0.00	0.00	0.00
GT55	0.00	0.00	0.00	0.00	0.00	0.00	0.00	0.00	0.00	0.00	0.00	0.00	0.00
GT18	0.00	0.00	0.00	0.00	0.00	0.00	0.00	0.00	0.00	0.00	0.00	0.00	0.00
GH87	0.00	0.00	0.00	0.00	0.00	0.00	0.00	0.00	0.01	0.01	0.00	0.00	0.02
PL5	0.00	0.00	0.00	0.00	0.00	0.00	0.00	0.00	0.00	0.00	0.00	0.00	0.00
GH22	0.00	0.00	0.00	0.00	0.00	0.00	0.00	0.00	0.00	0.00	0.00	0.00	0.00
CE2	0.07	0.09	0.05	0.10	0.05	0.06	0.19	0.08	0.06	0.08	0.02	0.10	0.08
GH120	0.01	0.11	0.10	0.03	0.00	0.09	0.03	0.04	0.09	0.08	0.10	0.00	0.05
GH73	0.53	0.66	0.57	0.79	0.45	0.51	0.58	0.40	0.52	0.49	0.70	0.44	0.40
GT17	0.00	0.00	0.02	0.00	0.00	0.01	0.00	0.00	0.00	0.01	0.00	0.00	0.00
GH124	0.00	0.00	0.00	0.00	0.00	0.00	0.00	0.00	0.00	0.00	0.00	0.00	0.00
GH54	0.00	0.00	0.00	0.00	0.00	0.00	0.00	0.00	0.00	0.00	0.00	0.00	0.00
GH100	0.00	0.00	0.00	0.00	0.00	0.00	0.00	0.00	0.00	0.00	0.00	0.00	0.00
GH113	0.02	0.04	0.08	0.14	0.00	0.02	0.03	0.05	0.04	0.04	0.02	0.05	0.03
GT5	0.33	0.49	0.51	0.68	0.51	0.49	0.48	0.47	0.47	0.43	0.49	0.33	0.51
GH94	0.33	0.66	0.64	0.99	0.10	0.49	0.74	0.31	0.43	0.47	0.31	0.30	0.65
GT66	0.00	0.00	0.00	0.00	0.00	0.00	0.00	0.00	0.00	0.00	0.00	0.00	0.00
GT94	0.00	0.00	0.00	0.00	0.00	0.00	0.00	0.00	0.00	0.00	0.00	0.00	0.00
GH58	0.00	0.00	0.00	0.00	0.02	0.00	0.00	0.00	0.00	0.00	0.00	0.00	0.00
GH1	0.45	0.40	0.49	0.75	0.29	0.42	0.23	0.35	0.55	0.55	0.43	0.37	0.65
PL22	0.00	0.00	0.00	0.00	0.00	0.00	0.00	0.00	0.00	0.00	0.00	0.00	0.00

CAZY_group	DA-AD-74	DA-AD-75	DA-AD-76	DA-AD-77	DA-AD-78	DA-AD-79	DA-AD-8	DA-AD-80	DA-AD-81	DA-AD-82	DA-AD-83	DA-AD-84	DA-AD-85
**GT76**	0.00	0.00	0.00	0.00	0.00	0.00	0.00	0.00	0.00	0.00	0.00	0.00	0.00
**GT32**	0.10	0.09	0.10	0.10	0.08	0.09	0.06	0.10	0.10	0.09	0.04	0.10	0.07
**GT47**	0.08	0.04	0.05	0.03	0.08	0.07	0.06	0.07	0.05	0.06	0.02	0.05	0.01
**GH84**	0.08	0.13	0.14	0.07	0.13	0.11	0.10	0.05	0.07	0.12	0.16	0.13	0.03
**GH83**	0.00	0.00	0.00	0.00	0.00	0.00	0.00	0.00	0.00	0.00	0.00	0.00	0.00
**GT59**	0.00	0.00	0.00	0.00	0.00	0.00	0.00	0.00	0.00	0.00	0.00	0.00	0.00
**GH34**	0.00	0.00	0.00	0.00	0.00	0.00	0.00	0.00	0.00	0.00	0.00	0.00	0.00
**GT14**	0.02	0.04	0.02	0.00	0.03	0.04	0.03	0.03	0.02	0.05	0.02	0.04	0.01
**GH47**	0.00	0.00	0.00	0.00	0.00	0.00	0.00	0.00	0.00	0.00	0.00	0.00	0.00
**GH97**	0.46	0.92	0.52	0.27	0.57	0.86	0.80	0.63	0.76	0.55	0.86	0.97	0.47
**GH50**	0.03	0.01	0.02	0.00	0.02	0.02	0.00	0.06	0.00	0.05	0.04	0.08	0.01
**GT26**	0.17	0.24	0.22	0.21	0.11	0.12	0.06	0.14	0.18	0.19	0.25	0.13	0.22
**GH18**	0.40	0.43	0.49	0.24	0.40	0.64	0.51	0.55	0.53	0.71	0.72	0.68	0.41
**GH37**	0.00	0.00	0.01	0.00	0.08	0.00	0.00	0.02	0.00	0.01	0.00	0.01	0.01
**PL13**	0.01	0.04	0.01	0.00	0.02	0.02	0.03	0.03	0.01	0.04	0.04	0.02	0.01
**GH27**	0.18	0.26	0.16	0.10	0.11	0.32	0.26	0.22	0.27	0.18	0.31	0.24	0.24
**GT54**	0.00	0.00	0.00	0.00	0.00	0.00	0.00	0.00	0.00	0.00	0.00	0.00	0.00
**GT91**	0.00	0.00	0.00	0.00	0.00	0.00	0.00	0.00	0.00	0.00	0.00	0.00	0.00
**GT72**	0.00	0.00	0.00	0.00	0.00	0.00	0.00	0.00	0.00	0.00	0.00	0.00	0.00
**GH67**	0.08	0.09	0.06	0.00	0.08	0.08	0.13	0.05	0.12	0.07	0.04	0.04	0.07
**GH12**	0.00	0.00	0.00	0.00	0.00	0.00	0.00	0.00	0.00	0.00	0.00	0.00	0.00
**GH91**	0.02	0.01	0.00	0.00	0.00	0.06	0.00	0.03	0.00	0.02	0.00	0.06	0.03
**GT69**	0.00	0.00	0.00	0.00	0.00	0.00	0.00	0.00	0.00	0.00	0.00	0.00	0.00
**GH106**	0.16	0.15	0.13	0.07	0.03	0.14	0.19	0.13	0.14	0.18	0.31	0.33	0.08
**GT62**	0.00	0.00	0.00	0.00	0.00	0.00	0.00	0.00	0.00	0.00	0.00	0.00	0.00
**GH17**	0.00	0.00	0.00	0.00	0.00	0.00	0.00	0.00	0.00	0.00	0.00	0.00	0.00
**GH88**	0.27	0.14	0.16	0.10	0.13	0.31	0.29	0.22	0.36	0.24	0.35	0.39	0.17
**CE5**	0.00	0.00	0.00	0.00	0.00	0.00	0.00	0.00	0.00	0.00	0.00	0.00	0.00
**GT8**	0.06	0.11	0.13	0.10	0.14	0.12	0.03	0.08	0.04	0.09	0.18	0.06	0.16
**GT50**	0.00	0.00	0.00	0.00	0.00	0.00	0.00	0.00	0.00	0.00	0.00	0.00	0.00
**GT20**	0.09	0.05	0.04	0.00	0.10	0.09	0.00	0.06	0.05	0.07	0.08	0.11	0.03
**PL9**	0.03	0.09	0.12	0.14	0.08	0.07	0.06	0.08	0.08	0.05	0.10	0.02	0.06
**GH55**	0.01	0.04	0.06	0.00	0.05	0.06	0.03	0.02	0.02	0.02	0.04	0.01	0.01
**GH64**	0.00	0.00	0.00	0.00	0.00	0.00	0.00	0.00	0.00	0.00	0.00	0.00	0.00
**GH127**	0.22	0.41	0.33	0.17	0.18	0.45	0.23	0.21	0.35	0.24	0.31	0.37	0.29
**GH103**	0.00	0.00	0.00	0.00	0.02	0.00	0.00	0.01	0.00	0.00	0.00	0.00	0.01
**GH3**	1.87	3.00	2.12	1.47	1.27	2.98	2.96	2.21	2.32	2.31	3.22	2.85	2.49
**GT45**	0.00	0.00	0.00	0.00	0.00	0.00	0.00	0.00	0.00	0.00	0.00	0.00	0.00
**GT74**	0.00	0.00	0.00	0.00	0.00	0.00	0.00	0.00	0.00	0.00	0.00	0.00	0.00
**GT90**	0.00	0.00	0.00	0.00	0.00	0.00	0.00	0.00	0.00	0.00	0.00	0.00	0.00
**GH116**	0.05	0.09	0.04	0.00	0.02	0.12	0.00	0.05	0.08	0.10	0.21	0.12	0.04
**GH31**	0.81	1.10	1.08	0.86	0.64	1.06	1.13	0.78	0.82	1.01	1.27	1.04	0.95
**GT12**	0.00	0.00	0.00	0.00	0.00	0.00	0.00	0.00	0.00	0.00	0.00	0.00	0.00
**CE1**	0.39	0.37	0.37	0.24	0.37	0.40	0.48	0.30	0.37	0.37	0.43	0.35	0.29
**GH121**	0.00	0.00	0.00	0.00	0.00	0.00	0.00	0.00	0.00	0.00	0.00	0.00	0.03
**GH79**	0.01	0.00	0.00	0.00	0.00	0.02	0.03	0.02	0.01	0.01	0.00	0.01	0.01
**GT73**	0.00	0.00	0.00	0.00	0.00	0.00	0.00	0.00	0.00	0.00	0.00	0.00	0.00
**GH77**	0.55	0.54	0.66	0.72	0.51	0.66	0.87	0.51	0.49	0.71	0.62	0.56	0.84
**PL17**	0.01	0.04	0.00	0.00	0.00	0.00	0.00	0.02	0.00	0.00	0.00	0.01	0.00
**PL8**	0.21	0.24	0.09	0.00	0.13	0.21	0.29	0.24	0.24	0.14	0.27	0.32	0.09

CAZY_group	DA-AD-74	DA-AD-75	DA-AD-76	DA-AD-77	DA-AD-78	DA-AD-79	DA-AD-8	DA-AD-80	DA-AD-81	DA-AD-82	DA-AD-83	DA-AD-84	DA-AD-85
**CE9**	0.32	0.34	0.28	0.14	0.24	0.30	0.42	0.25	0.21	0.28	0.39	0.33	0.40
**CE15**	0.01	0.00	0.02	0.00	0.00	0.00	0.00	0.00	0.02	0.04	0.04	0.01	0.01
**GT79**	0.00	0.00	0.00	0.00	0.00	0.00	0.00	0.00	0.00	0.00	0.00	0.00	0.00
**GT10**	0.00	0.00	0.02	0.00	0.00	0.01	0.00	0.01	0.01	0.00	0.00	0.02	0.01
**GH119**	0.00	0.00	0.00	0.00	0.00	0.00	0.00	0.00	0.00	0.00	0.00	0.00	0.00
**GH16**	0.14	0.14	0.22	0.24	0.08	0.19	0.13	0.20	0.08	0.15	0.18	0.32	0.14
**GH43**	1.49	2.13	1.98	1.10	0.76	2.01	1.74	1.72	2.30	1.54	1.85	2.12	1.47
**GH51**	0.51	0.46	0.53	0.34	0.30	0.66	0.64	0.46	0.49	0.38	0.60	0.61	0.51
**GT35**	0.97	0.93	0.91	1.23	0.80	0.93	1.16	0.83	0.88	0.83	0.96	0.57	0.96
**GT25**	0.00	0.00	0.00	0.00	0.00	0.01	0.00	0.00	0.00	0.01	0.00	0.00	0.00
**GT2**	4.15	4.66	4.96	4.52	3.36	4.49	4.18	3.81	3.74	3.93	4.95	4.77	4.10
**GT51**	0.77	1.05	0.81	0.99	0.72	0.91	1.03	0.72	0.78	0.84	0.94	0.86	0.81
**GH107**	0.00	0.00	0.00	0.00	0.00	0.00	0.00	0.00	0.00	0.00	0.00	0.00	0.00
**GH7**	0.00	0.00	0.00	0.00	0.00	0.00	0.00	0.00	0.00	0.00	0.00	0.00	0.00
**GT7**	0.00	0.00	0.00	0.00	0.00	0.00	0.00	0.00	0.00	0.00	0.00	0.00	0.00
**GT36**	0.00	0.00	0.00	0.00	0.00	0.00	0.00	0.00	0.00	0.00	0.00	0.00	0.00
**PL7**	0.00	0.00	0.00	0.00	0.00	0.00	0.00	0.00	0.00	0.00	0.00	0.00	0.00
**GH110**	0.11	0.11	0.12	0.00	0.03	0.11	0.00	0.07	0.07	0.06	0.18	0.12	0.05
**GT21**	0.00	0.00	0.00	0.00	0.00	0.00	0.00	0.00	0.00	0.00	0.00	0.00	0.00
**GH62**	0.00	0.00	0.00	0.00	0.00	0.00	0.00	0.00	0.00	0.00	0.00	0.00	0.00
**GH125**	0.11	0.10	0.16	0.03	0.14	0.17	0.10	0.11	0.16	0.15	0.25	0.18	0.11
**GH117**	0.04	0.05	0.07	0.00	0.05	0.02	0.10	0.05	0.06	0.09	0.14	0.08	0.02
**GH68**	0.00	0.00	0.00	0.00	0.00	0.00	0.00	0.00	0.00	0.00	0.02	0.00	0.01
**GH85**	0.00	0.01	0.01	0.00	0.02	0.02	0.03	0.02	0.00	0.00	0.02	0.01	0.03
**GH92**	1.08	1.14	1.22	0.72	0.81	1.68	1.61	0.91	1.05	1.43	2.11	2.12	0.78
**GH40**	0.00	0.00	0.00	0.00	0.00	0.00	0.00	0.00	0.00	0.00	0.00	0.00	0.00
**GT49**	0.00	0.00	0.00	0.00	0.00	0.00	0.00	0.00	0.00	0.00	0.00	0.00	0.00
**GH112**	0.13	0.08	0.14	0.24	0.03	0.14	0.19	0.13	0.11	0.06	0.10	0.11	0.22
**GT67**	0.00	0.00	0.00	0.00	0.00	0.00	0.00	0.00	0.00	0.00	0.00	0.00	0.00
**CE8**	0.22	0.22	0.12	0.10	0.14	0.19	0.13	0.16	0.18	0.16	0.14	0.22	0.14
**GT86**	0.00	0.00	0.00	0.00	0.00	0.00	0.00	0.00	0.00	0.00	0.00	0.00	0.00
**GH101**	0.00	0.00	0.02	0.00	0.00	0.01	0.00	0.00	0.00	0.00	0.00	0.00	0.01
**GH81**	0.00	0.00	0.00	0.00	0.00	0.01	0.00	0.00	0.00	0.00	0.00	0.01	0.00
**GT93**	0.00	0.00	0.00	0.00	0.00	0.00	0.00	0.00	0.00	0.00	0.00	0.00	0.00
**GT63**	0.00	0.00	0.00	0.00	0.00	0.00	0.00	0.00	0.00	0.00	0.00	0.00	0.00
**CE11**	0.22	0.15	0.24	0.17	0.18	0.19	0.32	0.18	0.23	0.24	0.31	0.23	0.15
**GT19**	0.15	0.08	0.09	0.21	0.11	0.13	0.26	0.11	0.11	0.11	0.16	0.11	0.05
**GT33**	0.00	0.00	0.00	0.00	0.00	0.00	0.00	0.00	0.00	0.00	0.00	0.00	0.00
**GH21**	0.00	0.00	0.00	0.00	0.00	0.00	0.00	0.00	0.00	0.00	0.00	0.00	0.00
**GT68**	0.00	0.00	0.00	0.00	0.00	0.00	0.00	0.00	0.00	0.00	0.00	0.00	0.00
**GH89**	0.15	0.10	0.09	0.14	0.16	0.27	0.10	0.17	0.08	0.23	0.37	0.24	0.06
**GT82**	0.00	0.00	0.00	0.00	0.00	0.00	0.00	0.00	0.00	0.00	0.00	0.00	0.00
**GH30**	0.11	0.23	0.16	0.17	0.08	0.21	0.03	0.13	0.27	0.20	0.31	0.23	0.16
**GH20**	0.90	0.71	0.69	0.44	0.43	0.97	1.16	0.77	0.62	0.97	1.62	1.35	0.56
**GH52**	0.00	0.00	0.00	0.00	0.00	0.00	0.00	0.00	0.00	0.00	0.00	0.00	0.00
**GT84**	0.05	0.06	0.01	0.00	0.06	0.06	0.00	0.03	0.04	0.01	0.00	0.00	0.02
**GT15**	0.00	0.00	0.00	0.00	0.00	0.00	0.00	0.00	0.00	0.00	0.00	0.00	0.00
**GH93**	0.00	0.00	0.00	0.00	0.00	0.00	0.00	0.00	0.00	0.01	0.00	0.00	0.00
**PL16**	0.00	0.00	0.00	0.00	0.00	0.00	0.00	0.00	0.00	0.00	0.00	0.00	0.01
**GH26**	0.14	0.26	0.16	0.27	0.05	0.25	0.23	0.21	0.16	0.08	0.08	0.12	0.15

CAZY_group	DA-AD-74	DA-AD-75	DA-AD-76	DA-AD-77	DA-AD-78	DA-AD-79	DA-AD-8	DA-AD-80	DA-AD-81	DA-AD-82	DA-AD-83	DA-AD-84	DA-AD-85
**GT75**	0.00	0.00	0.00	0.00	0.00	0.00	0.00	0.00	0.00	0.00	0.00	0.00	0.00
**GH53**	0.08	0.15	0.18	0.34	0.02	0.07	0.29	0.24	0.13	0.11	0.06	0.12	0.10
**GT88**	0.00	0.00	0.00	0.00	0.00	0.00	0.00	0.00	0.00	0.00	0.00	0.00	0.00
**GT24**	0.00	0.00	0.00	0.00	0.00	0.00	0.00	0.00	0.00	0.00	0.00	0.00	0.00
**GT81**	0.00	0.00	0.00	0.00	0.00	0.00	0.00	0.00	0.00	0.00	0.00	0.00	0.00
**GH4**	0.11	0.08	0.03	0.10	0.14	0.05	0.06	0.06	0.10	0.11	0.12	0.17	0.17
**GT78**	0.00	0.00	0.00	0.00	0.00	0.00	0.00	0.00	0.00	0.00	0.00	0.00	0.00
**GH8**	0.06	0.13	0.12	0.10	0.06	0.11	0.00	0.08	0.09	0.06	0.08	0.05	0.05
**GH70**	0.00	0.00	0.00	0.00	0.00	0.00	0.00	0.00	0.00	0.00	0.00	0.00	0.18
**GH122**	0.00	0.00	0.00	0.00	0.00	0.00	0.00	0.00	0.00	0.00	0.00	0.00	0.00
**GH74**	0.02	0.06	0.05	0.00	0.02	0.04	0.03	0.06	0.01	0.01	0.00	0.01	0.05
**GT41**	0.00	0.01	0.01	0.03	0.02	0.02	0.00	0.00	0.01	0.01	0.00	0.01	0.02
**PL1**	0.21	0.28	0.17	0.17	0.18	0.18	0.32	0.19	0.23	0.24	0.21	0.26	0.15
**GH15**	0.05	0.05	0.07	0.00	0.06	0.08	0.00	0.05	0.04	0.08	0.06	0.05	0.01
**GH32**	0.31	0.37	0.40	0.44	0.22	0.54	0.42	0.26	0.30	0.41	0.57	0.41	0.58
**GH44**	0.00	0.00	0.00	0.00	0.00	0.00	0.00	0.00	0.01	0.00	0.00	0.01	0.02
**GT6**	0.00	0.00	0.00	0.00	0.00	0.00	0.00	0.00	0.00	0.00	0.00	0.01	0.00
**GH13**	3.05	3.60	3.41	5.51	2.49	3.08	4.86	3.09	3.02	3.10	3.47	2.63	3.85
**GT80**	0.00	0.00	0.02	0.00	0.02	0.01	0.00	0.04	0.03	0.02	0.02	0.01	0.00
**GH71**	0.00	0.00	0.00	0.00	0.00	0.00	0.00	0.00	0.00	0.00	0.00	0.00	0.00
**GH63**	0.06	0.17	0.10	0.03	0.10	0.12	0.13	0.04	0.10	0.10	0.10	0.13	0.06
**PL21**	0.03	0.03	0.00	0.03	0.00	0.00	0.06	0.02	0.00	0.00	0.04	0.00	0.00
**GH65**	0.11	0.08	0.16	0.21	0.16	0.22	0.29	0.09	0.14	0.13	0.14	0.14	0.15
**CE4**	0.56	0.71	0.61	0.82	0.53	0.69	0.68	0.67	0.62	0.57	0.62	0.49	0.69
**CE14**	0.01	0.03	0.04	0.00	0.03	0.00	0.00	0.02	0.04	0.02	0.00	0.02	0.00
**GH115**	0.22	0.13	0.23	0.14	0.13	0.22	0.35	0.20	0.21	0.14	0.16	0.25	0.19
**GH104**	0.00	0.00	0.00	0.00	0.02	0.00	0.00	0.00	0.00	0.00	0.00	0.00	0.00
**GH111**	0.00	0.00	0.00	0.00	0.00	0.00	0.00	0.00	0.00	0.00	0.00	0.00	0.00
**GH95**	0.43	0.51	0.49	0.38	0.14	0.51	0.74	0.34	0.44	0.34	0.53	0.54	0.35
**GT70**	0.00	0.00	0.00	0.00	0.00	0.00	0.00	0.00	0.00	0.00	0.00	0.00	0.00
**CE10**	0.08	0.08	0.12	0.07	0.10	0.10	0.13	0.08	0.15	0.15	0.23	0.22	0.11
**GH10**	0.06	0.11	0.18	0.14	0.05	0.15	0.10	0.09	0.17	0.13	0.04	0.06	0.11
**GH130**	0.30	0.34	0.29	0.24	0.18	0.32	0.35	0.36	0.28	0.32	0.37	0.50	0.25
**PL20**	0.00	0.00	0.00	0.00	0.00	0.00	0.00	0.00	0.00	0.00	0.00	0.00	0.00
**GH69**	0.00	0.00	0.00	0.00	0.00	0.00	0.00	0.00	0.00	0.00	0.00	0.00	0.00
**PL2**	0.00	0.00	0.00	0.00	0.00	0.00	0.00	0.00	0.00	0.00	0.00	0.00	0.00
**GH49**	0.00	0.00	0.00	0.00	0.00	0.00	0.00	0.00	0.00	0.00	0.00	0.00	0.00
**GH25**	0.31	0.45	0.42	0.92	0.38	0.37	0.45	0.33	0.31	0.30	0.45	0.35	0.39
**GH59**	0.01	0.05	0.01	0.00	0.00	0.04	0.03	0.02	0.03	0.02	0.00	0.02	0.03
**GH35**	0.26	0.29	0.24	0.21	0.14	0.32	0.29	0.19	0.22	0.27	0.45	0.42	0.19
**GT39**	0.00	0.00	0.00	0.00	0.00	0.03	0.00	0.00	0.00	0.00	0.00	0.00	0.00
**GT57**	0.00	0.00	0.00	0.00	0.00	0.00	0.00	0.00	0.00	0.00	0.00	0.00	0.00
**GH129**	0.00	0.00	0.00	0.00	0.00	0.01	0.00	0.00	0.01	0.02	0.00	0.00	0.03
**GT22**	0.00	0.00	0.00	0.00	0.00	0.00	0.00	0.00	0.00	0.00	0.00	0.00	0.00
**PL12**	0.06	0.08	0.05	0.00	0.02	0.10	0.06	0.05	0.03	0.07	0.12	0.17	0.05
**PL18**	0.00	0.00	0.00	0.00	0.00	0.00	0.00	0.00	0.00	0.00	0.00	0.00	0.00
**GT92**	0.00	0.00	0.00	0.00	0.00	0.00	0.00	0.00	0.00	0.00	0.00	0.00	0.00
**GT44**	0.00	0.00	0.00	0.00	0.00	0.00	0.00	0.00	0.00	0.00	0.00	0.00	0.00
**GT29**	0.00	0.00	0.00	0.00	0.00	0.00	0.00	0.00	0.00	0.00	0.00	0.00	0.00
**GT13**	0.00	0.00	0.00	0.00	0.00	0.00	0.00	0.00	0.00	0.00	0.00	0.00	0.00

CAZY_group	DA-AD-74	DA-AD-75	DA-AD-76	DA-AD-77	DA-AD-78	DA-AD-79	DA-AD-8	DA-AD-80	DA-AD-81	DA-AD-82	DA-AD-83	DA-AD-84	DA-AD-85
**GT52**	0.00	0.00	0.00	0.00	0.00	0.00	0.00	0.00	0.00	0.00	0.00	0.00	0.00
**GT64**	0.00	0.00	0.00	0.00	0.00	0.00	0.00	0.00	0.00	0.00	0.00	0.00	0.00
**GT85**	0.00	0.00	0.00	0.00	0.00	0.00	0.00	0.00	0.00	0.00	0.00	0.00	0.00
**GT16**	0.00	0.00	0.00	0.00	0.00	0.00	0.00	0.00	0.00	0.00	0.00	0.00	0.00
**GH56**	0.00	0.00	0.00	0.00	0.00	0.00	0.00	0.00	0.00	0.00	0.00	0.00	0.00
**GT60**	0.00	0.00	0.00	0.00	0.00	0.00	0.00	0.00	0.00	0.00	0.00	0.00	0.00
**GH41**	0.00	0.00	0.00	0.00	0.00	0.00	0.00	0.00	0.00	0.00	0.00	0.00	0.00
**CE7**	0.04	0.05	0.08	0.00	0.06	0.04	0.03	0.05	0.09	0.08	0.21	0.16	0.06
**GH39**	0.04	0.10	0.04	0.17	0.02	0.05	0.13	0.04	0.04	0.04	0.04	0.04	0.07
**GT30**	0.23	0.18	0.20	0.17	0.19	0.19	0.26	0.23	0.17	0.24	0.29	0.26	0.13
**PL15**	0.04	0.10	0.03	0.00	0.03	0.09	0.03	0.11	0.04	0.09	0.14	0.13	0.06
**GH102**	0.02	0.01	0.02	0.00	0.06	0.03	0.00	0.01	0.02	0.04	0.02	0.00	0.01

CAZY_group	DA-AD-9	IN-CH-12
GH29	1.15	0.65
PL4	0.00	0.00
GH23	1.10	0.72
CE6	0.00	0.26
GH72	0.16	0.21
GH114	0.00	0.00
GH78	0.31	0.19
PL19	0.00	0.00
GT27	0.00	0.00
GH5	0.94	0.77
GT40	0.00	0.00
CE13	0.00	0.00
GT37	0.00	0.00
GH33	0.10	0.00
GT65	0.00	0.00
GH86	0.00	0.00
GH123	0.05	0.05
GH96	0.00	0.00
GH14	0.00	0.00
CE3	0.00	0.00
PL10	0.21	0.21
GT48	0.00	0.00
PL6	0.00	0.00
GT83	0.05	0.05
GH126	0.00	0.00
GH9	0.21	0.04
GH75	0.00	0.00
GT71	0.00	0.00
GH46	0.00	0.00
GT61	0.00	0.00
GT3	0.26	0.32
PL3	0.00	0.00
GH108	0.05	0.02
GT56	0.00	0.02
PL11	0.00	0.04
GH45	0.00	0.00
GT34	0.00	0.00
PL14	0.00	0.00
GH19	0.00	0.02
GH118	0.00	0.00
GH82	0.00	0.00
GH76	0.00	0.00
GT4	1.52	1.34
GT87	0.00	0.00
GH24	0.05	0.39
GH90	0.00	0.00
GH57	0.00	0.12
GH36	1.10	1.04
GT46	0.00	0.00
GH66	0.00	0.02

CAZY_group	DA-AD-9	IN-CH-12
GH48	0.00	0.00
GT53	0.00	0.00
GH60	0.00	0.00
GT31	0.00	0.00
GH105	0.10	0.28
GT23	0.00	0.00
GH11	0.00	0.00
GT38	0.00	0.00
GT43	0.00	0.00
GH99	0.00	0.00
GH38	0.16	0.02
GH109	0.16	0.25
GH2	3.77	2.90
GT28	0.52	0.39
GH42	0.42	0.05
GT58	0.00	0.00
GH61	0.00	0.00
GH80	0.00	0.00
GH98	0.00	0.00
GH6	0.00	0.00
GT1	0.10	0.00
GH28	0.79	0.88
GT42	0.00	0.00
GH128	0.00	0.00
CE12	0.10	0.33
CE16	0.00	0.00
GT89	0.00	0.00
GT11	0.00	0.05
GT77	0.00	0.07
GT55	0.00	0.00
GT18	0.00	0.00
GH87	0.00	0.00
PL5	0.00	0.00
GH22	0.00	0.00
CE2	0.05	0.05
GH120	0.05	0.00
GH73	0.73	0.65
GT17	0.00	0.00
GH124	0.00	0.00
GH54	0.00	0.00
GH100	0.00	0.00
GH113	0.05	0.00
GT5	0.84	0.30
GH94	0.73	0.35
GT66	0.00	0.00
GT94	0.00	0.00
GH58	0.00	0.00
GH1	0.16	0.07
PL22	0.00	0.00

CAZY_group	DA-AD-9	IN-CH-12
**GT76**	0.00	0.00
**GT32**	0.16	0.09
**GT47**	0.00	0.00
**GH84**	0.10	0.30
**GH83**	0.00	0.00
**GT59**	0.00	0.00
**GH34**	0.00	0.00
**GT14**	0.00	0.02
**GH47**	0.00	0.00
**GH97**	0.73	1.26
**GH50**	0.00	0.00
**GT26**	0.16	0.28
**GH18**	0.37	0.11
**GH37**	0.00	0.02
**PL13**	0.00	0.00
**GH27**	0.16	0.18
**GT54**	0.00	0.00
**GT91**	0.00	0.00
**GT72**	0.00	0.00
**GH67**	0.00	0.21
**GH12**	0.00	0.00
**GH91**	0.00	0.00
**GT69**	0.00	0.00
**GH106**	0.21	0.21
**GT62**	0.00	0.00
**GH17**	0.00	0.00
**GH88**	0.00	0.12
**CE5**	0.00	0.00
**GT8**	0.05	0.07
**GT50**	0.00	0.00
**GT20**	0.00	0.00
**PL9**	0.00	0.00
**GH55**	0.00	0.00
**GH64**	0.00	0.00
**GH127**	0.31	0.37
**GH103**	0.00	0.00
**GH3**	2.25	2.76
**GT45**	0.00	0.00
**GT74**	0.00	0.00
**GT90**	0.00	0.00
**GH116**	0.05	0.00
**GH31**	1.05	0.98
**GT12**	0.00	0.00
**CE1**	0.26	0.30
**GH121**	0.00	0.00
**GH79**	0.00	0.00
**GT73**	0.00	0.00
**GH77**	0.63	0.49
**PL17**	0.00	0.00
**PL8**	0.00	0.05

CAZY_group	DA-AD-9	IN-CH-12
CE9	0.42	0.19
CE15	0.00	0.00
GT79	0.00	0.00
GT10	0.00	0.00
GH119	0.00	0.00
GH16	0.26	0.25
GH43	1.78	2.55
GH51	0.63	1.21
GT35	0.94	0.58
GT25	0.00	0.00
GT2	3.93	4.97
GT51	1.26	1.30
GH107	0.00	0.00
GH7	0.00	0.00
GT7	0.00	0.00
GT36	0.00	0.00
PL7	0.00	0.00
GH110	0.10	0.11
GT21	0.00	0.00
GH62	0.00	0.00
GH125	0.10	0.21
GH117	0.05	0.00
GH68	0.00	0.00
GH85	0.10	0.16
GH92	0.79	1.02
GH40	0.00	0.00
GT49	0.00	0.00
GH112	0.16	0.02
GT67	0.00	0.00
CE8	0.16	0.35
GT86	0.00	0.00
GH101	0.00	0.00
GH81	0.00	0.00
GT93	0.00	0.00
GT63	0.00	0.00
CE11	0.42	0.40
GT19	0.26	0.26
GT33	0.00	0.00
GH21	0.00	0.00
GT68	0.00	0.00
GH89	0.26	0.14
GT82	0.00	0.00
GH30	0.05	0.19
GH20	1.15	1.05
GH52	0.00	0.00
GT84	0.00	0.00
GT15	0.00	0.00
GH93	0.00	0.00
PL16	0.00	0.00
GH26	0.21	0.00

CAZY_group	DA-AD-9	IN-CH-12
GT75	0.00	0.00
GH53	0.73	0.14
GT88	0.00	0.00
GT24	0.00	0.00
GT81	0.00	0.00
GH4	0.00	0.05
GT78	0.00	0.00
GH8	0.05	0.12
GH70	0.00	0.02
GH122	0.00	0.00
GH74	0.00	0.00
GT41	0.00	0.00
PL1	0.21	0.12
GH15	0.00	0.00
GH32	0.94	0.69
GH44	0.00	0.00
GT6	0.00	0.00
GH13	4.61	3.39
GT80	0.00	0.00
GH71	0.00	0.00
GH63	0.05	0.05
PL21	0.00	0.00
GH65	0.10	0.02
CE4	0.73	0.35
CE14	0.05	0.00
GH115	0.10	0.26
GH104	0.00	0.02
GH111	0.00	0.00
GH95	0.89	0.72
GT70	0.00	0.00
CE10	0.05	0.09
GH10	0.10	0.19
GH130	0.21	0.30
PL20	0.00	0.00
GH69	0.00	0.00
PL2	0.00	0.00
GH49	0.00	0.00
GH25	0.58	0.86
GH59	0.00	0.00
GH35	0.16	0.35
GT39	0.00	0.00
GT57	0.00	0.00
GH129	0.00	0.00
GT22	0.00	0.00
PL12	0.00	0.02
PL18	0.00	0.00
GT92	0.00	0.00
GT44	0.00	0.00
GT29	0.00	0.00
GT13	0.00	0.00

CAZY_group	DA-AD-9	IN-CH-12
**GT52**	0.00	0.00
**GT64**	0.00	0.00
**GT85**	0.00	0.00
**GT16**	0.00	0.00
**GH56**	0.00	0.00
**GT60**	0.00	0.00
**GH41**	0.00	0.00
**CE7**	0.21	0.16
**GH39**	0.16	0.02
**GT30**	0.37	0.28
**PL15**	0.00	0.05
**GH102**	0.00	0.04

CAZY_group	CN-AD-67	CN-AD-68	CN-AD-69	CN-AD-70	ES-AD-1	ES-AD-10	ES-AD-11	ES-AD-12	ES-AD-13	ES-AD-14	ES-AD-15	ES-AD-16	ES-AD-17	ES-AD-18	ES-AD-19	ES-AD-2
GH29	0.48	0.64	0.37	0.62	1.04	1.16	0.08	0.86	0.58	0.45	0.65	0.76	0.62	0.86	0.47	0.92
PL4	0.00	0.00	0.00	0.00	0.00	0.00	0.00	0.00	0.00	0.00	0.00	0.00	0.00	0.00	0.00	0.00
GH23	0.69	1.24	1.08	0.90	1.50	1.34	0.29	1.08	1.09	1.03	1.29	1.05	1.48	0.88	0.87	1.59
CE6	0.03	0.02	0.02	0.07	0.05	0.03	0.01	0.01	0.01	0.02	0.02	0.03	0.02	0.00	0.00	0.03
GH72	0.03	0.09	0.04	0.08	0.17	0.15	0.01	0.08	0.06	0.12	0.04	0.14	0.15	0.09	0.07	0.19
GH114	0.00	0.00	0.00	0.00	0.00	0.00	0.00	0.00	0.00	0.00	0.00	0.00	0.00	0.00	0.00	0.00
GH78	0.42	0.53	0.90	1.08	1.35	0.96	0.06	0.81	0.38	0.67	0.91	0.98	0.82	0.24	0.66	0.72
PL19	0.00	0.00	0.00	0.00	0.00	0.00	0.00	0.00	0.00	0.00	0.00	0.00	0.00	0.00	0.00	0.00
GT27	0.00	0.00	0.00	0.00	0.00	0.00	0.00	0.00	0.00	0.00	0.00	0.00	0.00	0.00	0.00	0.00
GH5	0.24	0.46	0.19	0.38	0.20	0.30	0.07	0.36	0.47	0.38	0.84	0.44	0.34	0.57	0.22	0.45
GT40	0.00	0.00	0.00	0.00	0.00	0.00	0.00	0.00	0.00	0.00	0.00	0.00	0.00	0.00	0.00	0.00
CE13	0.00	0.00	0.00	0.00	0.00	0.00	0.00	0.00	0.00	0.00	0.00	0.00	0.00	0.00	0.00	0.00
GT37	0.00	0.00	0.00	0.00	0.00	0.00	0.00	0.00	0.00	0.00	0.00	0.00	0.00	0.00	0.00	0.00
GH33	0.18	0.25	0.16	0.34	0.39	0.35	0.04	0.14	0.21	0.19	0.28	0.27	0.23	0.16	0.16	0.22
GT65	0.00	0.00	0.00	0.00	0.00	0.00	0.00	0.00	0.00	0.00	0.00	0.00	0.00	0.00	0.00	0.00
GH86	0.00	0.00	0.02	0.00	0.00	0.00	0.00	0.01	0.00	0.00	0.00	0.00	0.02	0.00	0.00	0.00
GH123	0.00	0.07	0.18	0.11	0.17	0.13	0.03	0.13	0.04	0.09	0.11	0.17	0.14	0.12	0.06	0.07
GH96	0.00	0.00	0.00	0.00	0.00	0.00	0.00	0.00	0.00	0.00	0.00	0.00	0.00	0.00	0.00	0.00
GH14	0.00	0.00	0.00	0.00	0.00	0.00	0.00	0.00	0.00	0.00	0.00	0.00	0.00	0.00	0.00	0.00
CE3	0.00	0.00	0.00	0.00	0.00	0.00	0.00	0.00	0.00	0.00	0.00	0.00	0.00	0.00	0.00	0.00
PL10	0.12	0.16	0.11	0.14	0.06	0.20	0.01	0.17	0.17	0.05	0.11	0.05	0.12	0.04	0.12	0.14
GT48	0.00	0.00	0.00	0.00	0.00	0.00	0.00	0.00	0.00	0.00	0.00	0.00	0.00	0.00	0.00	0.00
PL6	0.00	0.00	0.00	0.01	0.00	0.00	0.00	0.00	0.00	0.00	0.00	0.01	0.00	0.00	0.00	0.00
GT83	0.03	0.05	0.07	0.07	0.21	0.20	0.03	0.02	0.06	0.05	0.11	0.10	0.03	0.09	0.09	0.08
GH126	0.00	0.00	0.00	0.00	0.00	0.00	0.00	0.00	0.00	0.00	0.00	0.00	0.00	0.00	0.00	0.00
GH9	0.12	0.14	0.18	0.12	0.12	0.28	0.01	0.08	0.04	0.11	0.30	0.04	0.26	0.32	0.07	0.24
GH75	0.00	0.00	0.00	0.00	0.00	0.00	0.00	0.00	0.00	0.00	0.00	0.00	0.00	0.00	0.00	0.00
GT71	0.00	0.00	0.00	0.00	0.00	0.00	0.00	0.00	0.00	0.00	0.00	0.00	0.00	0.00	0.00	0.00
GH46	0.00	0.00	0.00	0.00	0.00	0.00	0.00	0.00	0.00	0.00	0.00	0.00	0.02	0.00	0.00	0.00
GT61	0.00	0.00	0.00	0.00	0.00	0.00	0.00	0.00	0.00	0.00	0.00	0.00	0.00	0.00	0.00	0.00
GT3	0.15	0.07	0.12	0.07	0.24	0.20	0.00	0.17	0.17	0.07	0.09	0.20	0.20	0.27	0.14	0.26
PL3	0.00	0.00	0.00	0.00	0.00	0.00	0.00	0.00	0.00	0.00	0.00	0.00	0.00	0.00	0.00	0.00
GH108	0.03	0.07	0.07	0.01	0.02	0.05	0.00	0.04	0.01	0.00	0.02	0.03	0.03	0.01	0.07	0.01
GT56	0.00	0.00	0.00	0.00	0.00	0.00	0.00	0.00	0.00	0.00	0.00	0.00	0.02	0.00	0.00	0.00
PL11	0.15	0.07	0.07	0.22	0.21	0.15	0.03	0.07	0.08	0.10	0.22	0.09	0.17	0.05	0.09	0.15
GH45	0.03	0.00	0.02	0.00	0.00	0.03	0.00	0.01	0.00	0.01	0.00	0.00	0.02	0.00	0.00	0.00
GT34	0.00	0.00	0.00	0.00	0.00	0.00	0.00	0.00	0.00	0.00	0.00	0.00	0.00	0.00	0.01	0.00
PL14	0.00	0.00	0.00	0.00	0.00	0.00	0.00	0.00	0.00	0.00	0.00	0.00	0.00	0.00	0.00	0.00
GH19	0.00	0.00	0.04	0.01	0.02	0.00	0.00	0.03	0.00	0.00	0.00	0.01	0.00	0.03	0.00	0.03
GH118	0.00	0.00	0.00	0.00	0.00	0.00	0.00	0.00	0.00	0.00	0.00	0.00	0.00	0.00	0.00	0.00
GH82	0.00	0.00	0.00	0.00	0.00	0.00	0.00	0.00	0.00	0.00	0.00	0.00	0.00	0.00	0.00	0.00
GH76	0.09	0.16	0.18	0.22	0.20	0.38	0.03	0.16	0.03	0.11	0.24	0.24	0.26	0.04	0.14	0.04
GT4	1.09	1.91	2.05	1.52	2.45	2.51	0.18	1.45	1.56	1.66	1.75	2.10	1.91	1.26	1.95	2.13
GT87	0.00	0.00	0.00	0.00	0.00	0.03	0.00	0.01	0.00	0.00	0.02	0.00	0.00	0.01	0.00	0.00
GH24	0.36	0.14	0.16	0.18	0.21	0.25	0.01	0.14	0.10	0.07	0.13	0.08	0.12	0.14	0.15	0.19
GH90	0.00	0.00	0.00	0.00	0.00	0.00	0.00	0.00	0.00	0.00	0.00	0.00	0.00	0.00	0.00	0.00
GH57	0.12	0.14	0.14	0.11	0.14	0.25	0.00	0.21	0.13	0.07	0.11	0.07	0.15	0.16	0.11	0.14
GH36	0.51	0.44	0.49	0.66	0.86	0.68	0.13	0.65	0.48	0.67	1.03	0.91	0.57	0.59	0.63	0.88
GT46	0.00	0.00	0.00	0.00	0.00	0.00	0.00	0.00	0.00	0.00	0.00	0.00	0.00	0.00	0.00	0.00
GH66	0.00	0.00	0.02	0.03	0.08	0.03	0.00	0.03	0.04	0.01	0.06	0.03	0.05	0.03	0.05	0.07

CAZY_group	CN-AD-67	CN-AD-68	CN-AD-69	CN-AD-70	ES-AD-1	ES-AD-10	ES-AD-11	ES-AD-12	ES-AD-13	ES-AD-14	ES-AD-15	ES-AD-16	ES-AD-17	ES-AD-18	ES-AD-19	ES-AD-2
GH48	0.00	0.00	0.00	0.00	0.00	0.00	0.00	0.00	0.01	0.00	0.00	0.01	0.00	0.00	0.00	0.00
GT53	0.00	0.00	0.00	0.00	0.00	0.00	0.00	0.00	0.00	0.00	0.00	0.00	0.00	0.00	0.00	0.00
GH60	0.00	0.00	0.00	0.00	0.00	0.00	0.00	0.00	0.00	0.00	0.00	0.00	0.00	0.00	0.00	0.00
GT31	0.00	0.00	0.00	0.00	0.00	0.00	0.01	0.00	0.00	0.00	0.00	0.00	0.02	0.00	0.00	0.00
GH105	0.36	0.51	0.49	0.63	0.82	0.86	0.13	0.45	0.38	0.38	0.69	0.50	0.54	0.18	0.37	0.38
GT23	0.00	0.02	0.00	0.00	0.00	0.00	0.00	0.00	0.00	0.02	0.02	0.02	0.02	0.00	0.01	0.00
GH11	0.00	0.00	0.00	0.00	0.00	0.00	0.00	0.00	0.00	0.00	0.00	0.00	0.00	0.00	0.00	0.00
GT38	0.00	0.00	0.00	0.00	0.00	0.00	0.00	0.00	0.00	0.00	0.00	0.00	0.00	0.00	0.00	0.00
GT43	0.00	0.00	0.00	0.00	0.00	0.00	0.00	0.00	0.00	0.00	0.00	0.00	0.00	0.00	0.00	0.00
GH99	0.00	0.00	0.02	0.01	0.03	0.00	0.00	0.00	0.00	0.00	0.00	0.02	0.05	0.00	0.00	0.00
GH38	0.06	0.12	0.34	0.27	0.35	0.10	0.04	0.14	0.04	0.25	0.52	0.51	0.26	0.08	0.38	0.16
GH109	0.30	0.35	0.21	0.22	0.38	0.48	0.03	0.31	0.21	0.16	0.22	0.26	0.35	0.24	0.15	0.24
GH2	2.50	3.11	3.78	4.03	5.31	5.80	0.63	3.42	2.55	2.97	4.70	4.34	3.99	2.78	2.63	4.29
GT28	0.18	0.21	0.39	0.29	0.42	0.53	0.06	0.37	0.42	0.43	0.54	0.48	0.45	0.54	0.47	0.57
GH42	0.09	0.09	0.19	0.37	0.24	0.10	0.07	0.10	0.17	0.36	0.30	0.35	0.14	0.14	0.31	0.24
GT58	0.00	0.00	0.00	0.00	0.00	0.00	0.00	0.00	0.00	0.00	0.00	0.00	0.00	0.00	0.00	0.00
GH61	0.00	0.00	0.00	0.00	0.00	0.00	0.00	0.00	0.00	0.00	0.00	0.00	0.00	0.00	0.00	0.00
GH80	0.00	0.00	0.00	0.00	0.00	0.00	0.00	0.00	0.00	0.00	0.00	0.00	0.00	0.00	0.00	0.00
GH98	0.00	0.00	0.00	0.01	0.00	0.05	0.00	0.01	0.00	0.00	0.00	0.02	0.02	0.00	0.00	0.00
GH6	0.00	0.00	0.00	0.00	0.00	0.00	0.00	0.00	0.00	0.00	0.00	0.00	0.00	0.00	0.00	0.00
GT1	0.00	0.02	0.05	0.03	0.05	0.00	0.00	0.05	0.01	0.09	0.09	0.06	0.00	0.07	0.12	0.04
GH28	0.81	0.71	0.62	0.85	1.14	1.11	0.08	0.80	0.69	0.69	1.16	0.73	0.74	0.27	0.64	0.79
GT42	0.00	0.00	0.00	0.00	0.00	0.00	0.00	0.00	0.00	0.00	0.00	0.00	0.00	0.00	0.00	0.00
GH128	0.00	0.00	0.00	0.00	0.00	0.00	0.00	0.00	0.00	0.00	0.00	0.00	0.00	0.00	0.00	0.00
CE12	0.18	0.09	0.09	0.18	0.20	0.28	0.00	0.17	0.10	0.15	0.19	0.17	0.11	0.11	0.07	0.12
CE16	0.00	0.00	0.00	0.00	0.00	0.00	0.00	0.00	0.00	0.00	0.00	0.00	0.00	0.00	0.00	0.00
GT89	0.00	0.00	0.00	0.00	0.00	0.00	0.00	0.00	0.00	0.00	0.00	0.00	0.00	0.00	0.00	0.00
GT11	0.06	0.07	0.04	0.03	0.06	0.10	0.00	0.06	0.03	0.06	0.06	0.06	0.08	0.00	0.02	0.01
GT77	0.00	0.02	0.00	0.00	0.00	0.00	0.00	0.00	0.00	0.00	0.00	0.00	0.00	0.00	0.00	0.00
GT55	0.00	0.00	0.00	0.00	0.00	0.00	0.00	0.00	0.00	0.00	0.00	0.00	0.00	0.00	0.00	0.00
GT18	0.00	0.00	0.00	0.00	0.00	0.00	0.00	0.00	0.00	0.00	0.00	0.00	0.00	0.00	0.00	0.00
GH87	0.00	0.00	0.00	0.00	0.00	0.00	0.00	0.00	0.00	0.00	0.00	0.00	0.00	0.00	0.04	0.01
PL5	0.00	0.00	0.00	0.00	0.00	0.00	0.00	0.00	0.00	0.00	0.00	0.00	0.00	0.00	0.00	0.00
GH22	0.00	0.00	0.00	0.00	0.00	0.00	0.00	0.00	0.00	0.00	0.00	0.00	0.00	0.00	0.00	0.00
CE2	0.03	0.02	0.04	0.03	0.03	0.05	0.00	0.05	0.10	0.07	0.11	0.15	0.08	0.07	0.01	0.04
GH120	0.00	0.02	0.04	0.10	0.08	0.00	0.00	0.04	0.00	0.11	0.11	0.10	0.03	0.04	0.06	0.08
GH73	0.24	0.39	0.32	0.34	0.39	0.68	0.11	0.49	0.52	0.43	0.54	0.48	0.32	0.57	0.68	0.45
GT17	0.00	0.00	0.00	0.00	0.00	0.00	0.00	0.00	0.00	0.00	0.00	0.00	0.00	0.00	0.00	0.00
GH124	0.00	0.00	0.00	0.00	0.00	0.00	0.00	0.00	0.00	0.00	0.00	0.00	0.00	0.00	0.00	0.00
GH54	0.00	0.00	0.00	0.00	0.00	0.00	0.00	0.00	0.00	0.00	0.00	0.00	0.00	0.00	0.00	0.00
GH100	0.00	0.00	0.00	0.00	0.00	0.00	0.00	0.00	0.00	0.00	0.00	0.00	0.00	0.00	0.00	0.00
GH113	0.00	0.00	0.02	0.01	0.00	0.00	0.00	0.02	0.03	0.02	0.11	0.03	0.02	0.04	0.00	0.01
GT5	0.42	0.21	0.35	0.44	0.30	0.46	0.04	0.42	0.38	0.40	0.45	0.39	0.34	0.47	0.44	0.54
GH94	0.12	0.23	0.18	0.31	0.24	0.15	0.01	0.23	0.25	0.37	0.67	0.52	0.31	0.38	0.21	0.53
GT66	0.00	0.00	0.00	0.00	0.02	0.00	0.00	0.00	0.03	0.00	0.00	0.00	0.00	0.00	0.01	0.00
GT94	0.00	0.00	0.00	0.00	0.00	0.00	0.00	0.00	0.00	0.00	0.00	0.00	0.00	0.00	0.00	0.00
GH58	0.00	0.00	0.00	0.00	0.00	0.00	0.00	0.00	0.00	0.00	0.00	0.00	0.02	0.00	0.00	0.00
GH1	0.33	0.21	0.35	1.04	0.85	0.68	0.22	0.39	0.52	0.71	0.67	0.50	0.25	0.45	0.63	0.57
PL22	0.00	0.00	0.04	0.00	0.00	0.00	0.00	0.00	0.00	0.00	0.00	0.00	0.00	0.00	0.00	0.00
GT9	0.12	0.14	0.18	0.10	0.14	0.35	0.00	0.08	0.13	0.04	0.06	0.12	0.14	0.08	0.30	0.16

CAZY_group	CN-AD-67	CN-AD-68	CN-AD-69	CN-AD-70	ES-AD-1	ES-AD-10	ES-AD-11	ES-AD-12	ES-AD-13	ES-AD-14	ES-AD-15	ES-AD-16	ES-AD-17	ES-AD-18	ES-AD-19	ES-AD-2
GT76	0.00	0.00	0.00	0.00	0.00	0.00	0.00	0.00	0.00	0.00	0.00	0.00	0.00	0.00	0.00	0.00
GT32	0.03	0.09	0.04	0.11	0.11	0.15	0.00	0.03	0.01	0.09	0.11	0.08	0.11	0.05	0.04	0.09
GT47	0.03	0.05	0.02	0.04	0.08	0.00	0.00	0.01	0.03	0.02	0.06	0.05	0.05	0.03	0.05	0.05
GH84	0.09	0.09	0.09	0.16	0.21	0.18	0.01	0.11	0.08	0.07	0.06	0.14	0.15	0.15	0.05	0.14
GH83	0.00	0.00	0.00	0.00	0.00	0.00	0.00	0.00	0.00	0.00	0.00	0.00	0.00	0.00	0.00	0.00
GT59	0.00	0.00	0.00	0.00	0.00	0.00	0.00	0.00	0.00	0.00	0.00	0.00	0.00	0.00	0.00	0.00
GH34	0.00	0.00	0.00	0.00	0.00	0.00	0.00	0.00	0.00	0.00	0.00	0.00	0.00	0.00	0.00	0.00
GT14	0.00	0.02	0.02	0.03	0.09	0.05	0.00	0.02	0.03	0.01	0.09	0.01	0.00	0.00	0.01	0.01
GH47	0.00	0.00	0.00	0.00	0.00	0.00	0.00	0.00	0.00	0.00	0.00	0.00	0.00	0.00	0.00	0.00
GH97	0.51	0.53	0.72	0.84	1.24	1.32	0.13	0.75	0.48	0.47	0.75	0.87	0.66	0.54	0.57	0.90
GH50	0.06	0.02	0.02	0.05	0.05	0.05	0.00	0.00	0.00	0.02	0.04	0.04	0.00	0.00	0.05	0.03
GT26	0.12	0.12	0.14	0.14	0.21	0.38	0.03	0.21	0.11	0.21	0.30	0.25	0.18	0.14	0.14	0.19
GH18	0.18	0.28	0.56	0.62	0.86	0.61	0.20	0.47	0.48	0.55	0.54	0.79	0.51	0.39	0.59	0.45
GH37	0.00	0.00	0.09	0.00	0.00	0.03	0.01	0.01	0.00	0.00	0.00	0.02	0.06	0.00	0.04	0.00
PL13	0.03	0.02	0.07	0.04	0.05	0.00	0.00	0.04	0.01	0.02	0.00	0.03	0.05	0.00	0.01	0.00
GH27	0.06	0.21	0.26	0.23	0.27	0.35	0.06	0.17	0.20	0.15	0.30	0.26	0.40	0.16	0.15	0.14
GT54	0.00	0.00	0.00	0.00	0.00	0.00	0.00	0.00	0.00	0.00	0.00	0.00	0.00	0.00	0.00	0.00
GT91	0.00	0.00	0.00	0.00	0.00	0.00	0.00	0.00	0.00	0.00	0.00	0.00	0.00	0.00	0.00	0.00
GT72	0.00	0.00	0.00	0.00	0.00	0.00	0.00	0.00	0.00	0.00	0.00	0.00	0.00	0.00	0.00	0.00
GH67	0.12	0.05	0.05	0.11	0.05	0.13	0.04	0.08	0.04	0.04	0.06	0.10	0.09	0.01	0.02	0.07
GH12	0.00	0.00	0.00	0.00	0.00	0.00	0.00	0.00	0.00	0.00	0.00	0.00	0.00	0.00	0.00	0.00
GH91	0.00	0.05	0.00	0.01	0.06	0.00	0.00	0.04	0.00	0.00	0.00	0.03	0.00	0.00	0.01	0.01
GT69	0.00	0.00	0.00	0.00	0.00	0.00	0.00	0.00	0.00	0.00	0.00	0.00	0.00	0.00	0.00	0.00
GH106	0.12	0.23	0.30	0.30	0.47	0.35	0.01	0.31	0.17	0.11	0.28	0.21	0.28	0.08	0.16	0.24
GT62	0.00	0.00	0.00	0.00	0.00	0.00	0.00	0.00	0.00	0.00	0.00	0.00	0.00	0.00	0.00	0.00
GH17	0.00	0.00	0.00	0.00	0.00	0.00	0.00	0.00	0.00	0.00	0.00	0.00	0.00	0.00	0.00	0.01
GH88	0.21	0.21	0.35	0.34	0.55	0.51	0.15	0.37	0.21	0.26	0.28	0.32	0.23	0.04	0.14	0.26
CE5	0.00	0.00	0.00	0.00	0.00	0.00	0.00	0.00	0.00	0.00	0.00	0.00	0.00	0.00	0.00	0.00
GT8	0.03	0.02	0.09	0.07	0.12	0.08	0.00	0.08	0.10	0.19	0.09	0.18	0.15	0.04	0.31	0.18
GT50	0.00	0.00	0.00	0.00	0.00	0.00	0.00	0.00	0.00	0.00	0.00	0.00	0.00	0.00	0.00	0.00
GT20	0.06	0.09	0.11	0.12	0.20	0.18	0.03	0.07	0.06	0.10	0.06	0.10	0.09	0.00	0.04	0.07
PL9	0.06	0.02	0.02	0.10	0.14	0.08	0.00	0.08	0.04	0.11	0.24	0.12	0.11	0.03	0.02	0.23
GH55	0.00	0.02	0.04	0.01	0.03	0.05	0.00	0.01	0.01	0.01	0.02	0.02	0.06	0.00	0.04	0.01
GH64	0.00	0.00	0.00	0.00	0.00	0.00	0.00	0.00	0.00	0.00	0.00	0.00	0.00	0.00	0.00	0.00
GH127	0.12	0.09	0.26	0.31	0.36	0.30	0.03	0.19	0.28	0.26	0.32	0.41	0.28	0.12	0.22	0.27
GH103	0.00	0.00	0.02	0.01	0.00	0.00	0.00	0.00	0.00	0.00	0.00	0.00	0.06	0.00	0.01	0.01
GH3	1.45	1.93	1.99	3.08	3.00	3.24	0.75	2.18	1.67	2.33	3.79	3.14	2.31	1.99	2.05	3.04
GT45	0.00	0.00	0.00	0.00	0.00	0.00	0.00	0.00	0.00	0.00	0.00	0.00	0.00	0.00	0.00	0.00
GT74	0.00	0.00	0.00	0.00	0.00	0.00	0.00	0.00	0.00	0.00	0.00	0.00	0.00	0.00	0.00	0.00
GT90	0.00	0.00	0.00	0.00	0.00	0.00	0.00	0.00	0.00	0.00	0.00	0.00	0.00	0.01	0.00	0.00
GH116	0.06	0.05	0.04	0.07	0.09	0.05	0.00	0.06	0.06	0.06	0.09	0.04	0.12	0.03	0.02	0.07
GH31	0.63	0.69	0.71	1.16	1.47	1.59	0.27	0.92	0.61	1.02	1.31	1.26	0.97	0.66	0.89	0.94
GT12	0.00	0.00	0.00	0.00	0.00	0.00	0.00	0.00	0.00	0.00	0.00	0.00	0.00	0.00	0.00	0.00
CE1	0.21	0.23	0.49	0.31	0.53	0.73	0.10	0.43	0.34	0.32	0.32	0.55	0.26	0.42	0.40	0.46
GH121	0.00	0.00	0.00	0.00	0.03	0.00	0.00	0.00	0.00	0.05	0.00	0.01	0.02	0.00	0.00	0.01
GH79	0.00	0.00	0.00	0.00	0.00	0.00	0.00	0.00	0.00	0.00	0.00	0.00	0.00	0.00	0.00	0.01
GT73	0.00	0.00	0.02	0.00	0.00	0.00	0.00	0.00	0.00	0.00	0.00	0.00	0.02	0.00	0.00	0.00
GH77	0.33	0.48	0.42	0.56	0.83	0.63	0.07	0.50	0.65	0.77	0.75	0.67	0.62	0.59	0.51	0.83
PL17	0.00	0.02	0.00	0.00	0.00	0.00	0.00	0.00	0.01	0.00	0.00	0.00	0.00	0.00	0.00	0.00
PL8	0.15	0.23	0.19	0.37	0.33	0.43	0.01	0.24	0.21	0.04	0.11	0.28	0.18	0.12	0.14	0.16

CAZY_group	CN-AD-67	CN-AD-68	CN-AD-69	CN-AD-70	ES-AD-1	ES-AD-10	ES-AD-11	ES-AD-12	ES-AD-13	ES-AD-14	ES-AD-15	ES-AD-16	ES-AD-17	ES-AD-18	ES-AD-19	ES-AD-2
**CE9**	0.30	0.37	0.25	0.41	0.36	0.43	0.13	0.36	0.35	0.42	0.34	0.41	0.40	0.26	0.41	0.43
**CE15**	0.00	0.02	0.02	0.00	0.03	0.03	0.00	0.01	0.00	0.01	0.00	0.04	0.02	0.00	0.01	0.00
**GT79**	0.00	0.00	0.00	0.00	0.00	0.00	0.00	0.00	0.00	0.00	0.00	0.00	0.00	0.00	0.00	0.00
**GT10**	0.00	0.00	0.00	0.00	0.00	0.00	0.00	0.01	0.01	0.00	0.00	0.01	0.00	0.00	0.00	0.00
**GH119**	0.00	0.00	0.00	0.00	0.00	0.00	0.00	0.00	0.00	0.00	0.00	0.00	0.00	0.00	0.00	0.00
**GH16**	0.09	0.09	0.23	0.19	0.27	0.25	0.01	0.15	0.11	0.09	0.17	0.24	0.23	0.15	0.12	0.16
**GH43**	1.48	2.05	2.22	2.31	2.32	3.24	0.32	1.62	1.13	1.64	2.26	2.19	1.96	0.89	1.32	1.95
**GH51**	0.30	0.37	0.56	0.44	0.64	0.51	0.04	0.47	0.35	0.61	0.50	0.67	0.43	0.28	0.42	0.43
**GT35**	0.42	0.83	0.56	0.78	1.27	0.73	0.21	0.68	0.79	0.94	1.12	0.84	0.97	0.88	0.73	1.18
**GT25**	0.00	0.00	0.02	0.00	0.02	0.00	0.00	0.00	0.00	0.01	0.00	0.00	0.00	0.00	0.01	0.00
**GT2**	2.68	3.25	3.65	3.77	6.77	5.49	0.78	3.93	3.31	4.59	5.22	4.84	4.04	3.39	3.93	7.19
**GT51**	0.51	0.60	0.65	0.70	1.06	1.04	0.21	0.85	0.83	0.96	1.03	1.11	0.85	0.80	1.04	1.25
**GH107**	0.00	0.00	0.00	0.00	0.00	0.00	0.00	0.00	0.00	0.00	0.00	0.00	0.00	0.00	0.00	0.00
**GH7**	0.00	0.00	0.00	0.00	0.00	0.00	0.00	0.00	0.00	0.00	0.00	0.00	0.00	0.00	0.00	0.00
**GT7**	0.00	0.00	0.00	0.00	0.00	0.00	0.00	0.00	0.00	0.00	0.00	0.00	0.00	0.00	0.00	0.00
**GT36**	0.00	0.00	0.00	0.00	0.00	0.00	0.00	0.00	0.00	0.00	0.00	0.00	0.00	0.00	0.00	0.00
**PL7**	0.00	0.00	0.00	0.00	0.00	0.00	0.00	0.00	0.00	0.00	0.00	0.00	0.00	0.00	0.00	0.00
**GH110**	0.12	0.16	0.09	0.08	0.18	0.15	0.00	0.04	0.07	0.04	0.02	0.10	0.09	0.14	0.02	0.08
**GT21**	0.00	0.00	0.00	0.00	0.00	0.00	0.00	0.00	0.00	0.00	0.00	0.00	0.00	0.00	0.00	0.00
**GH62**	0.00	0.00	0.00	0.00	0.02	0.00	0.00	0.00	0.00	0.00	0.00	0.00	0.00	0.00	0.00	0.00
**GH125**	0.06	0.21	0.18	0.21	0.59	0.25	0.11	0.10	0.04	0.19	0.30	0.20	0.17	0.11	0.15	0.11
**GH117**	0.09	0.12	0.12	0.07	0.15	0.10	0.00	0.12	0.06	0.05	0.06	0.08	0.12	0.08	0.09	0.08
**GH68**	0.00	0.00	0.00	0.00	0.00	0.00	0.00	0.00	0.00	0.00	0.00	0.00	0.00	0.00	0.05	0.00
**GH85**	0.00	0.05	0.00	0.00	0.08	0.00	0.00	0.01	0.00	0.06	0.04	0.02	0.03	0.04	0.06	0.01
**GH92**	1.15	1.34	1.80	1.79	2.27	2.76	0.20	1.37	0.81	0.89	1.16	1.95	1.46	1.05	1.11	1.14
**GH40**	0.00	0.00	0.00	0.00	0.00	0.00	0.00	0.00	0.00	0.00	0.00	0.00	0.00	0.00	0.00	0.00
**GT49**	0.00	0.00	0.00	0.00	0.00	0.00	0.00	0.00	0.00	0.00	0.00	0.00	0.00	0.00	0.00	0.00
**GH112**	0.09	0.09	0.05	0.23	0.21	0.13	0.08	0.11	0.10	0.26	0.26	0.11	0.08	0.11	0.19	0.34
**GT67**	0.00	0.00	0.00	0.00	0.00	0.00	0.00	0.00	0.00	0.00	0.00	0.00	0.00	0.00	0.00	0.00
**CE8**	0.15	0.21	0.19	0.23	0.27	0.13	0.03	0.22	0.30	0.21	0.28	0.25	0.22	0.09	0.10	0.12
**GT86**	0.00	0.00	0.00	0.00	0.00	0.00	0.00	0.00	0.00	0.00	0.00	0.00	0.00	0.00	0.00	0.00
**GH101**	0.00	0.00	0.00	0.07	0.03	0.00	0.00	0.00	0.00	0.06	0.00	0.00	0.03	0.00	0.00	0.01
**GH81**	0.00	0.00	0.02	0.00	0.00	0.00	0.00	0.00	0.00	0.00	0.00	0.00	0.00	0.00	0.00	0.00
**GT93**	0.00	0.00	0.00	0.00	0.00	0.00	0.00	0.00	0.00	0.00	0.00	0.00	0.00	0.00	0.00	0.00
**GT63**	0.00	0.00	0.00	0.00	0.00	0.00	0.00	0.00	0.00	0.00	0.00	0.00	0.00	0.00	0.00	0.00
**CE11**	0.15	0.21	0.23	0.11	0.18	0.25	0.04	0.19	0.25	0.22	0.24	0.27	0.25	0.22	0.22	0.20
**GT19**	0.15	0.16	0.18	0.07	0.23	0.28	0.01	0.17	0.11	0.10	0.15	0.15	0.15	0.14	0.16	0.18
**GT33**	0.00	0.00	0.00	0.00	0.00	0.00	0.00	0.00	0.00	0.00	0.00	0.00	0.00	0.00	0.00	0.00
**GH21**	0.00	0.00	0.00	0.00	0.00	0.00	0.00	0.00	0.00	0.00	0.00	0.00	0.00	0.00	0.00	0.00
**GT68**	0.00	0.00	0.00	0.00	0.00	0.00	0.00	0.00	0.00	0.00	0.00	0.00	0.00	0.00	0.00	0.00
**GH89**	0.21	0.25	0.23	0.23	0.33	0.38	0.03	0.23	0.11	0.17	0.19	0.20	0.14	0.14	0.15	0.18
**GT82**	0.00	0.00	0.00	0.00	0.00	0.00	0.00	0.00	0.00	0.01	0.00	0.00	0.00	0.00	0.00	0.00
**GH30**	0.09	0.21	0.23	0.26	0.36	0.38	0.03	0.10	0.17	0.19	0.30	0.23	0.20	0.08	0.15	0.24
**GH20**	0.78	0.90	1.01	1.29	1.97	1.95	0.13	1.16	0.62	0.79	0.91	1.52	1.13	0.68	0.75	0.69
**GH52**	0.00	0.00	0.00	0.00	0.00	0.00	0.00	0.00	0.00	0.00	0.00	0.00	0.00	0.00	0.00	0.00
**GT84**	0.00	0.00	0.00	0.00	0.00	0.00	0.00	0.00	0.01	0.02	0.02	0.00	0.02	0.04	0.00	0.03
**GT15**	0.00	0.00	0.00	0.00	0.00	0.00	0.00	0.00	0.00	0.00	0.00	0.00	0.00	0.00	0.00	0.00
**GH93**	0.00	0.00	0.02	0.00	0.02	0.00	0.00	0.00	0.00	0.00	0.00	0.00	0.00	0.00	0.00	0.00
**PL16**	0.00	0.00	0.00	0.00	0.00	0.00	0.00	0.00	0.00	0.00	0.00	0.00	0.00	0.00	0.00	0.00
**GH26**	0.03	0.02	0.07	0.12	0.17	0.13	0.00	0.04	0.16	0.16	0.26	0.22	0.28	0.14	0.04	0.22

CAZY_group	CN-AD-67	CN-AD-68	CN-AD-69	CN-AD-70	ES-AD-1	ES-AD-10	ES-AD-11	ES-AD-12	ES-AD-13	ES-AD-14	ES-AD-15	ES-AD-16	ES-AD-17	ES-AD-18	ES-AD-19	ES-AD-2
GT75	0.00	0.00	0.00	0.00	0.00	0.00	0.00	0.00	0.00	0.00	0.00	0.00	0.00	0.00	0.00	0.00
GH53	0.00	0.05	0.09	0.10	0.05	0.20	0.00	0.16	0.16	0.26	0.34	0.10	0.08	0.12	0.09	0.11
GT88	0.00	0.00	0.00	0.00	0.00	0.00	0.00	0.00	0.00	0.00	0.00	0.00	0.00	0.00	0.00	0.00
GT24	0.00	0.00	0.00	0.00	0.00	0.00	0.00	0.00	0.00	0.00	0.00	0.00	0.00	0.00	0.00	0.00
GT81	0.00	0.00	0.00	0.00	0.00	0.00	0.00	0.00	0.00	0.00	0.00	0.00	0.00	0.00	0.00	0.00
GH4	0.15	0.05	0.19	0.16	0.23	0.18	0.07	0.13	0.13	0.17	0.15	0.13	0.25	0.16	0.28	0.15
GT78	0.00	0.00	0.00	0.00	0.00	0.00	0.00	0.00	0.00	0.00	0.00	0.00	0.00	0.00	0.00	0.00
GH8	0.00	0.00	0.07	0.00	0.03	0.03	0.00	0.06	0.03	0.05	0.13	0.04	0.06	0.08	0.04	0.04
GH70	0.00	0.00	0.00	0.00	0.00	0.00	0.00	0.00	0.00	0.00	0.00	0.00	0.00	0.01	0.23	0.01
GH122	0.00	0.00	0.00	0.00	0.00	0.00	0.00	0.00	0.00	0.00	0.00	0.00	0.00	0.00	0.00	0.00
GH74	0.00	0.02	0.02	0.00	0.02	0.00	0.00	0.02	0.04	0.01	0.06	0.04	0.03	0.05	0.00	0.01
GT41	0.00	0.00	0.00	0.01	0.02	0.00	0.00	0.01	0.01	0.04	0.02	0.00	0.00	0.00	0.02	0.00
PL1	0.24	0.18	0.26	0.31	0.29	0.33	0.03	0.29	0.14	0.20	0.30	0.24	0.31	0.14	0.14	0.39
GH15	0.03	0.02	0.07	0.07	0.09	0.15	0.00	0.04	0.03	0.09	0.04	0.04	0.08	0.00	0.04	0.01
GH32	0.30	0.23	0.35	0.64	0.65	0.56	0.10	0.45	0.37	0.48	0.58	0.62	0.37	0.23	0.59	0.61
GH44	0.00	0.00	0.00	0.00	0.00	0.00	0.00	0.00	0.00	0.00	0.02	0.00	0.03	0.00	0.00	0.00
GT6	0.00	0.00	0.00	0.00	0.00	0.00	0.00	0.00	0.00	0.00	0.00	0.01	0.02	0.00	0.01	0.00
GH13	1.45	2.23	2.65	2.89	3.19	3.27	0.70	2.79	2.90	3.86	4.66	3.56	2.57	3.01	3.04	3.87
GT80	0.00	0.00	0.02	0.01	0.05	0.00	0.01	0.01	0.00	0.00	0.00	0.04	0.00	0.00	0.00	0.00
GH71	0.00	0.00	0.00	0.00	0.00	0.00	0.00	0.00	0.00	0.00	0.00	0.00	0.00	0.00	0.00	0.00
GH63	0.06	0.18	0.11	0.14	0.17	0.18	0.00	0.08	0.08	0.09	0.13	0.06	0.18	0.12	0.10	0.08
PL21	0.12	0.09	0.04	0.00	0.03	0.00	0.00	0.03	0.03	0.00	0.00	0.00	0.03	0.00	0.02	0.00
GH65	0.06	0.09	0.12	0.05	0.17	0.13	0.07	0.13	0.14	0.11	0.24	0.18	0.12	0.05	0.23	0.14
CE4	0.27	0.32	0.49	0.47	0.50	0.48	0.14	0.52	0.68	0.78	0.78	0.66	0.60	0.65	0.44	0.49
CE14	0.00	0.02	0.05	0.00	0.02	0.03	0.03	0.00	0.00	0.00	0.02	0.02	0.03	0.03	0.00	0.01
GH115	0.12	0.09	0.12	0.33	0.23	0.46	0.07	0.27	0.14	0.15	0.37	0.39	0.17	0.09	0.17	0.20
GH104	0.00	0.00	0.00	0.00	0.00	0.00	0.00	0.00	0.00	0.00	0.00	0.00	0.02	0.00	0.02	0.00
GH111	0.00	0.00	0.00	0.00	0.00	0.00	0.00	0.00	0.00	0.00	0.00	0.00	0.00	0.00	0.00	0.00
GH95	0.48	0.60	0.58	0.68	0.70	0.73	0.07	0.72	0.41	0.31	0.54	0.61	0.51	0.36	0.31	0.73
GT70	0.00	0.00	0.00	0.00	0.00	0.00	0.00	0.00	0.00	0.00	0.00	0.00	0.00	0.00	0.00	0.00
CE10	0.15	0.12	0.12	0.21	0.20	0.25	0.10	0.15	0.06	0.16	0.15	0.23	0.26	0.09	0.16	0.24
GH10	0.03	0.07	0.14	0.11	0.08	0.13	0.01	0.13	0.08	0.12	0.11	0.14	0.06	0.05	0.05	0.05
GH130	0.24	0.32	0.18	0.44	0.44	0.58	0.08	0.35	0.23	0.30	0.45	0.47	0.29	0.31	0.17	0.22
PL20	0.00	0.00	0.00	0.00	0.00	0.00	0.00	0.00	0.00	0.00	0.00	0.00	0.00	0.00	0.00	0.00
GH69	0.00	0.00	0.00	0.00	0.00	0.00	0.00	0.00	0.00	0.00	0.00	0.00	0.00	0.00	0.00	0.00
PL2	0.00	0.00	0.00	0.00	0.00	0.00	0.00	0.00	0.00	0.00	0.00	0.00	0.00	0.00	0.01	0.00
GH49	0.00	0.00	0.00	0.00	0.00	0.00	0.00	0.00	0.00	0.00	0.00	0.00	0.00	0.00	0.00	0.00
GH25	0.27	0.28	0.19	0.25	0.45	0.48	0.07	0.33	0.35	0.48	0.65	0.47	0.39	0.41	0.64	0.46
GH59	0.00	0.02	0.00	0.00	0.00	0.00	0.00	0.01	0.01	0.00	0.04	0.01	0.03	0.03	0.00	0.03
GH35	0.12	0.25	0.26	0.41	0.56	0.51	0.13	0.22	0.23	0.19	0.39	0.48	0.31	0.19	0.16	0.30
GT39	0.00	0.00	0.00	0.00	0.02	0.00	0.00	0.00	0.00	0.00	0.00	0.00	0.00	0.00	0.01	0.01
GT57	0.00	0.00	0.00	0.00	0.00	0.00	0.00	0.00	0.00	0.00	0.00	0.00	0.00	0.00	0.00	0.00
GH129	0.00	0.00	0.00	0.07	0.05	0.00	0.03	0.01	0.01	0.04	0.00	0.02	0.02	0.01	0.01	0.01
GT22	0.00	0.00	0.00	0.00	0.00	0.00	0.00	0.00	0.00	0.00	0.00	0.00	0.00	0.00	0.00	0.00
PL12	0.09	0.12	0.16	0.11	0.12	0.00	0.01	0.07	0.01	0.07	0.02	0.09	0.06	0.00	0.07	0.03
PL18	0.00	0.00	0.00	0.00	0.00	0.00	0.00	0.00	0.00	0.00	0.00	0.00	0.00	0.00	0.00	0.00
GT92	0.00	0.00	0.00	0.00	0.00	0.00	0.00	0.00	0.00	0.00	0.00	0.00	0.00	0.00	0.00	0.00
GT44	0.00	0.00	0.00	0.00	0.00	0.00	0.00	0.01	0.00	0.00	0.00	0.00	0.00	0.00	0.00	0.00
GT29	0.00	0.00	0.00	0.00	0.00	0.00	0.00	0.00	0.00	0.00	0.00	0.00	0.00	0.00	0.00	0.00
GT13	0.00	0.00	0.00	0.00	0.00	0.00	0.00	0.00	0.00	0.00	0.00	0.00	0.00	0.00	0.00	0.00

CAZY_group	CN-AD-67	CN-AD-68	CN-AD-69	CN-AD-70	ES-AD-1	ES-AD-10	ES-AD-11	ES-AD-12	ES-AD-13	ES-AD-14	ES-AD-15	ES-AD-16	ES-AD-17	ES-AD-18	ES-AD-19	ES-AD-2
GT52	0.00	0.00	0.00	0.00	0.00	0.00	0.00	0.00	0.00	0.00	0.00	0.00	0.00	0.00	0.00	0.00
GT64	0.00	0.00	0.00	0.00	0.00	0.00	0.00	0.00	0.00	0.00	0.00	0.00	0.00	0.00	0.00	0.00
GT85	0.00	0.00	0.00	0.00	0.00	0.00	0.00	0.00	0.00	0.00	0.00	0.00	0.00	0.00	0.00	0.00
GT16	0.00	0.00	0.00	0.00	0.00	0.00	0.00	0.00	0.00	0.00	0.00	0.00	0.00	0.00	0.00	0.00
GH56	0.00	0.00	0.00	0.00	0.00	0.00	0.00	0.00	0.00	0.00	0.00	0.00	0.00	0.00	0.00	0.00
GT60	0.00	0.00	0.00	0.00	0.00	0.00	0.00	0.00	0.00	0.00	0.00	0.00	0.00	0.00	0.00	0.00
GH41	0.00	0.00	0.00	0.00	0.00	0.00	0.00	0.00	0.00	0.00	0.00	0.00	0.00	0.00	0.00	0.00
CE7	0.03	0.12	0.04	0.10	0.23	0.20	0.00	0.11	0.07	0.11	0.13	0.12	0.09	0.03	0.10	0.05
GH39	0.00	0.05	0.09	0.07	0.03	0.00	0.01	0.02	0.03	0.02	0.06	0.06	0.03	0.04	0.01	0.01
GT30	0.36	0.21	0.26	0.21	0.35	0.40	0.03	0.21	0.10	0.17	0.17	0.22	0.28	0.15	0.23	0.24
PL15	0.06	0.09	0.09	0.16	0.14	0.10	0.01	0.07	0.01	0.05	0.09	0.11	0.06	0.00	0.09	0.12
GH102	0.00	0.00	0.07	0.04	0.06	0.00	0.01	0.02	0.00	0.04	0.00	0.02	0.09	0.01	0.01	0.04

CAZY_group	ES-AD-20	ES-AD-21	ES-AD-22	ES-AD-23	ES-AD-24	ES-AD-25	ES-AD-26	ES-AD-27	ES-AD-28	ES-AD-29	ES-AD-3	ES-AD-30	ES-AD-31	ES-AD-32	ES-AD-33	ES-AD-34
GH29	0.61	0.54	0.67	0.52	0.76	0.63	0.70	0.68	0.63	0.57	0.98	0.92	0.54	0.47	0.82	0.79
PL4	0.00	0.00	0.00	0.00	0.00	0.02	0.00	0.01	0.00	0.00	0.00	0.00	0.00	0.00	0.00	0.00
GH23	1.08	1.07	0.90	0.83	1.11	0.96	1.15	0.73	0.77	0.86	1.91	1.23	1.12	1.11	1.08	1.13
CE6	0.02	0.00	0.01	0.00	0.07	0.02	0.03	0.04	0.01	0.02	0.06	0.01	0.00	0.00	0.03	0.04
GH72	0.14	0.08	0.09	0.07	0.10	0.12	0.12	0.13	0.06	0.10	0.17	0.05	0.12	0.09	0.12	0.05
GH114	0.00	0.00	0.00	0.00	0.00	0.00	0.00	0.00	0.00	0.00	0.00	0.00	0.00	0.00	0.00	0.00
GH78	1.06	0.34	0.52	0.59	0.98	0.64	0.86	0.50	0.63	0.87	1.26	0.94	0.54	0.47	0.44	0.96
PL19	0.00	0.00	0.00	0.00	0.00	0.00	0.00	0.00	0.00	0.00	0.00	0.00	0.00	0.00	0.00	0.00
GT27	0.00	0.00	0.00	0.00	0.00	0.00	0.00	0.00	0.00	0.00	0.00	0.00	0.00	0.00	0.00	0.00
GH5	0.31	0.48	0.49	0.31	0.47	0.48	0.31	0.33	0.29	0.56	0.25	0.26	0.41	0.35	0.55	0.34
GT40	0.00	0.00	0.00	0.00	0.00	0.00	0.00	0.00	0.00	0.00	0.00	0.00	0.00	0.00	0.00	0.00
CE13	0.02	0.00	0.00	0.00	0.00	0.00	0.00	0.00	0.00	0.00	0.00	0.00	0.00	0.00	0.00	0.00
GT37	0.00	0.00	0.00	0.00	0.00	0.00	0.00	0.00	0.00	0.00	0.00	0.00	0.00	0.00	0.00	0.00
GH33	0.17	0.09	0.14	0.11	0.30	0.12	0.27	0.13	0.21	0.19	0.30	0.22	0.17	0.14	0.18	0.25
GT65	0.00	0.00	0.00	0.00	0.00	0.00	0.00	0.00	0.00	0.00	0.00	0.00	0.00	0.00	0.00	0.00
GH86	0.00	0.00	0.00	0.00	0.01	0.01	0.00	0.00	0.01	0.00	0.01	0.00	0.00	0.00	0.00	0.00
GH123	0.09	0.04	0.12	0.07	0.18	0.09	0.13	0.08	0.12	0.18	0.06	0.14	0.09	0.05	0.13	0.13
GH96	0.00	0.00	0.00	0.00	0.00	0.00	0.00	0.00	0.00	0.00	0.00	0.00	0.00	0.00	0.00	0.00
GH14	0.00	0.00	0.00	0.00	0.00	0.00	0.00	0.00	0.00	0.00	0.00	0.00	0.00	0.00	0.00	0.00
CE3	0.00	0.00	0.00	0.00	0.00	0.00	0.00	0.00	0.00	0.00	0.00	0.00	0.00	0.00	0.00	0.00
PL10	0.19	0.05	0.05	0.13	0.10	0.05	0.08	0.12	0.07	0.11	0.17	0.11	0.16	0.05	0.09	0.10
GT48	0.00	0.00	0.00	0.00	0.00	0.00	0.00	0.00	0.00	0.00	0.00	0.00	0.00	0.00	0.00	0.00
PL6	0.00	0.00	0.00	0.00	0.01	0.00	0.00	0.00	0.00	0.00	0.00	0.00	0.00	0.00	0.00	0.00
GT83	0.03	0.02	0.04	0.04	0.12	0.04	0.07	0.02	0.07	0.05	0.23	0.15	0.03	0.06	0.10	0.12
GH126	0.00	0.00	0.00	0.00	0.00	0.00	0.00	0.00	0.00	0.00	0.00	0.00	0.00	0.00	0.00	0.00
GH9	0.10	0.28	0.18	0.15	0.14	0.27	0.12	0.17	0.10	0.36	0.19	0.15	0.12	0.03	0.57	0.08
GH75	0.00	0.00	0.00	0.00	0.00	0.00	0.00	0.00	0.00	0.00	0.00	0.00	0.00	0.00	0.00	0.00
GT71	0.00	0.00	0.00	0.00	0.00	0.00	0.00	0.00	0.00	0.00	0.00	0.00	0.00	0.00	0.00	0.00
GH46	0.00	0.00	0.00	0.00	0.00	0.00	0.00	0.00	0.00	0.00	0.00	0.00	0.00	0.00	0.00	0.00
GT61	0.00	0.00	0.00	0.00	0.00	0.00	0.00	0.00	0.00	0.00	0.00	0.00	0.00	0.00	0.00	0.00
GT3	0.16	0.09	0.13	0.11	0.25	0.14	0.23	0.13	0.21	0.13	0.35	0.24	0.09	0.10	0.22	0.14
PL3	0.00	0.01	0.00	0.00	0.00	0.00	0.00	0.00	0.00	0.00	0.00	0.00	0.00	0.00	0.00	0.01
GH108	0.02	0.03	0.03	0.04	0.04	0.04	0.04	0.03	0.06	0.03	0.10	0.05	0.03	0.00	0.03	0.01
GT56	0.02	0.00	0.00	0.00	0.00	0.00	0.00	0.00	0.00	0.00	0.00	0.00	0.00	0.00	0.00	0.00
PL11	0.14	0.06	0.08	0.07	0.17	0.13	0.16	0.10	0.05	0.13	0.31	0.12	0.08	0.10	0.12	0.11
GH45	0.00	0.00	0.00	0.00	0.00	0.00	0.01	0.00	0.00	0.00	0.01	0.00	0.00	0.00	0.00	0.00
GT34	0.00	0.00	0.00	0.00	0.00	0.00	0.00	0.00	0.01	0.00	0.00	0.01	0.00	0.00	0.00	0.00
PL14	0.00	0.00	0.00	0.00	0.00	0.00	0.00	0.00	0.00	0.00	0.00	0.00	0.00	0.00	0.00	0.00
GH19	0.00	0.01	0.01	0.00	0.00	0.03	0.01	0.00	0.00	0.00	0.01	0.00	0.01	0.01	0.04	0.00
GH118	0.00	0.00	0.00	0.00	0.00	0.00	0.00	0.00	0.00	0.00	0.00	0.00	0.00	0.00	0.00	0.00
GH82	0.00	0.00	0.00	0.00	0.00	0.00	0.00	0.00	0.00	0.00	0.00	0.00	0.00	0.00	0.00	0.00
GH76	0.28	0.02	0.04	0.18	0.16	0.14	0.24	0.05	0.11	0.11	0.24	0.18	0.11	0.06	0.12	0.23
GT4	1.93	1.65	1.88	1.67	1.77	1.67	1.74	1.51	1.50	1.92	2.72	1.70	1.94	1.42	1.88	1.75
GT87	0.00	0.00	0.00	0.00	0.00	0.01	0.00	0.00	0.00	0.00	0.00	0.00	0.00	0.00	0.00	0.00
GH24	0.03	0.09	0.04	0.17	0.04	0.06	0.13	0.09	0.12	0.06	0.31	0.10	0.16	0.05	0.12	0.10
GH90	0.00	0.00	0.00	0.00	0.00	0.00	0.00	0.00	0.00	0.00	0.00	0.00	0.00	0.00	0.00	0.00
GH57	0.10	0.07	0.13	0.04	0.16	0.09	0.11	0.12	0.17	0.14	0.20	0.18	0.14	0.07	0.12	0.11
GH36	0.94	0.62	0.65	0.70	0.66	0.76	0.63	0.47	0.45	0.64	1.09	0.65	0.65	0.59	0.60	0.89
GT46	0.00	0.00	0.00	0.00	0.00	0.00	0.00	0.00	0.00	0.00	0.00	0.00	0.00	0.00	0.00	0.00
GH66	0.14	0.03	0.01	0.02	0.07	0.06	0.03	0.02	0.02	0.04	0.07	0.04	0.01	0.00	0.03	0.04

CAZY_group	ES-AD-20	ES-AD-21	ES-AD-22	ES-AD-23	ES-AD-24	ES-AD-25	ES-AD-26	ES-AD-27	ES-AD-28	ES-AD-29	ES-AD-3	ES-AD-30	ES-AD-31	ES-AD-32	ES-AD-33	ES-AD-34
GH48	0.00	0.00	0.01	0.00	0.00	0.00	0.00	0.00	0.00	0.00	0.00	0.00	0.00	0.00	0.01	0.00
GT53	0.00	0.00	0.00	0.00	0.00	0.00	0.00	0.00	0.00	0.00	0.00	0.00	0.00	0.00	0.00	0.00
GH60	0.00	0.00	0.00	0.00	0.00	0.00	0.00	0.00	0.00	0.00	0.00	0.00	0.00	0.00	0.00	0.00
GT31	0.00	0.00	0.00	0.00	0.00	0.00	0.00	0.00	0.00	0.00	0.00	0.00	0.00	0.00	0.00	0.00
GH105	0.70	0.34	0.35	0.35	0.56	0.31	0.56	0.35	0.25	0.50	0.84	0.54	0.26	0.20	0.36	0.65
GT23	0.02	0.01	0.02	0.04	0.01	0.00	0.00	0.00	0.00	0.00	0.00	0.01	0.00	0.00	0.00	0.00
GH11	0.00	0.01	0.01	0.00	0.01	0.01	0.00	0.00	0.00	0.01	0.00	0.00	0.00	0.01	0.00	0.00
GT38	0.00	0.00	0.00	0.00	0.00	0.00	0.00	0.00	0.00	0.00	0.00	0.00	0.00	0.00	0.00	0.00
GT43	0.00	0.00	0.00	0.00	0.00	0.00	0.00	0.00	0.00	0.00	0.00	0.00	0.00	0.00	0.00	0.00
GH99	0.00	0.00	0.00	0.00	0.00	0.01	0.01	0.00	0.01	0.00	0.00	0.02	0.00	0.00	0.01	0.00
GH38	0.37	0.12	0.27	0.31	0.28	0.15	0.36	0.16	0.15	0.23	0.53	0.23	0.24	0.24	0.14	0.35
GH109	0.21	0.16	0.18	0.11	0.35	0.16	0.31	0.23	0.30	0.21	0.47	0.37	0.11	0.14	0.27	0.18
GH2	4.84	2.67	3.15	3.33	4.70	3.10	3.78	2.47	2.70	3.55	6.27	3.89	2.70	2.34	3.48	4.22
GT28	0.50	0.43	0.48	0.37	0.45	0.42	0.32	0.41	0.33	0.41	0.43	0.45	0.45	0.44	0.43	0.42
GH42	0.38	0.25	0.24	0.37	0.41	0.30	0.21	0.20	0.07	0.22	0.29	0.25	0.31	0.28	0.14	0.34
GT58	0.00	0.00	0.00	0.00	0.00	0.00	0.00	0.00	0.00	0.00	0.00	0.00	0.00	0.00	0.00	0.00
GH61	0.00	0.00	0.00	0.00	0.00	0.00	0.00	0.00	0.00	0.00	0.00	0.00	0.00	0.00	0.00	0.00
GH80	0.00	0.00	0.00	0.00	0.00	0.00	0.00	0.00	0.00	0.00	0.00	0.00	0.00	0.00	0.00	0.00
GH98	0.00	0.01	0.00	0.00	0.00	0.00	0.01	0.00	0.00	0.00	0.00	0.00	0.00	0.00	0.00	0.00
GH6	0.00	0.00	0.00	0.00	0.00	0.00	0.00	0.00	0.00	0.00	0.01	0.00	0.00	0.00	0.00	0.00
GT1	0.10	0.03	0.04	0.04	0.08	0.06	0.03	0.05	0.04	0.05	0.06	0.02	0.03	0.06	0.01	0.02
GH28	0.78	0.45	0.56	0.72	1.05	0.51	1.04	0.54	0.38	0.80	1.20	0.60	0.62	0.47	0.73	0.73
GT42	0.00	0.00	0.00	0.00	0.00	0.00	0.00	0.00	0.00	0.00	0.00	0.00	0.00	0.00	0.00	0.00
GH128	0.00	0.00	0.00	0.00	0.00	0.00	0.00	0.00	0.00	0.00	0.00	0.00	0.00	0.00	0.00	0.00
CE12	0.12	0.13	0.10	0.06	0.14	0.08	0.11	0.09	0.09	0.18	0.12	0.10	0.07	0.08	0.12	0.14
CE16	0.00	0.00	0.00	0.00	0.00	0.00	0.00	0.00	0.00	0.00	0.00	0.00	0.00	0.00	0.00	0.00
GT89	0.00	0.00	0.00	0.00	0.00	0.00	0.00	0.00	0.00	0.00	0.00	0.00	0.00	0.00	0.00	0.00
GT11	0.05	0.06	0.05	0.07	0.07	0.02	0.08	0.03	0.05	0.02	0.08	0.09	0.04	0.01	0.03	0.04
GT77	0.02	0.00	0.00	0.00	0.00	0.00	0.00	0.00	0.00	0.00	0.00	0.00	0.00	0.01	0.00	0.00
GT55	0.00	0.00	0.00	0.00	0.00	0.00	0.00	0.00	0.00	0.00	0.00	0.00	0.00	0.00	0.00	0.00
GT18	0.00	0.00	0.00	0.00	0.00	0.00	0.00	0.00	0.00	0.00	0.00	0.00	0.00	0.00	0.00	0.00
GH87	0.03	0.00	0.00	0.00	0.00	0.00	0.00	0.02	0.00	0.00	0.00	0.00	0.01	0.00	0.01	0.01
PL5	0.00	0.00	0.00	0.00	0.00	0.00	0.00	0.00	0.00	0.00	0.00	0.00	0.00	0.00	0.00	0.00
GH22	0.00	0.00	0.00	0.00	0.00	0.00	0.00	0.00	0.00	0.00	0.00	0.00	0.00	0.00	0.00	0.00
CE2	0.09	0.10	0.10	0.04	0.03	0.08	0.07	0.04	0.07	0.07	0.06	0.07	0.03	0.09	0.04	0.05
GH120	0.03	0.10	0.07	0.11	0.12	0.07	0.04	0.06	0.00	0.06	0.06	0.11	0.08	0.11	0.01	0.09
GH73	0.73	0.50	0.48	0.33	0.52	0.42	0.36	0.33	0.28	0.43	0.79	0.58	0.43	0.45	0.48	0.34
GT17	0.00	0.01	0.00	0.00	0.00	0.00	0.00	0.00	0.00	0.00	0.00	0.01	0.00	0.00	0.00	0.00
GH124	0.00	0.00	0.00	0.00	0.00	0.00	0.00	0.00	0.00	0.00	0.00	0.00	0.00	0.00	0.00	0.00
GH54	0.00	0.00	0.00	0.00	0.00	0.00	0.00	0.00	0.00	0.00	0.00	0.00	0.00	0.00	0.00	0.00
GH100	0.00	0.00	0.00	0.00	0.00	0.00	0.00	0.00	0.00	0.00	0.00	0.00	0.00	0.00	0.00	0.00
GH113	0.00	0.05	0.03	0.02	0.03	0.07	0.04	0.01	0.01	0.01	0.04	0.00	0.04	0.02	0.01	0.01
GT5	0.44	0.55	0.43	0.35	0.39	0.48	0.47	0.51	0.37	0.40	0.48	0.35	0.46	0.47	0.40	0.39
GH94	0.12	0.66	0.42	0.35	0.39	0.58	0.24	0.36	0.12	0.47	0.42	0.20	0.38	0.42	0.36	0.36
GT66	0.00	0.00	0.01	0.00	0.00	0.00	0.00	0.02	0.01	0.00	0.00	0.00	0.00	0.00	0.00	0.00
GT94	0.00	0.00	0.00	0.00	0.00	0.00	0.00	0.00	0.00	0.00	0.00	0.00	0.00	0.00	0.00	0.00
GH58	0.00	0.00	0.00	0.00	0.00	0.00	0.00	0.00	0.00	0.00	0.00	0.00	0.00	0.00	0.00	0.00
GH1	0.78	0.53	0.27	0.64	0.47	0.46	0.60	0.35	0.13	0.36	0.68	0.23	0.84	0.55	0.29	0.55
PL22	0.02	0.00	0.00	0.00	0.00	0.00	0.00	0.00	0.00	0.00	0.00	0.00	0.00	0.00	0.00	0.00
GT9	0.10	0.07	0.10	0.04	0.12	0.05	0.15	0.06	0.11	0.08	0.25	0.12	0.08	0.05	0.22	0.13

CAZY_group	ES-AD-20	ES-AD-21	ES-AD-22	ES-AD-23	ES-AD-24	ES-AD-25	ES-AD-26	ES-AD-27	ES-AD-28	ES-AD-29	ES-AD-3	ES-AD-30	ES-AD-31	ES-AD-32	ES-AD-33	ES-AD-34
GT76	0.00	0.00	0.00	0.00	0.00	0.00	0.00	0.00	0.00	0.00	0.00	0.00	0.00	0.00	0.00	0.00
GT32	0.05	0.08	0.08	0.09	0.04	0.10	0.04	0.11	0.08	0.13	0.12	0.03	0.08	0.06	0.05	0.09
GT47	0.02	0.03	0.06	0.04	0.09	0.05	0.03	0.09	0.01	0.05	0.04	0.05	0.06	0.02	0.04	0.07
GH84	0.07	0.06	0.13	0.17	0.16	0.07	0.13	0.06	0.12	0.12	0.31	0.14	0.08	0.13	0.12	0.10
GH83	0.00	0.00	0.00	0.00	0.00	0.00	0.00	0.00	0.00	0.00	0.00	0.00	0.00	0.00	0.00	0.00
GT59	0.00	0.00	0.00	0.00	0.00	0.00	0.00	0.00	0.00	0.00	0.00	0.00	0.00	0.00	0.00	0.00
GH34	0.00	0.00	0.00	0.00	0.00	0.00	0.00	0.00	0.00	0.00	0.00	0.00	0.00	0.00	0.00	0.00
GT14	0.03	0.02	0.03	0.07	0.03	0.03	0.04	0.01	0.03	0.03	0.04	0.05	0.02	0.00	0.03	0.04
GH47	0.00	0.00	0.00	0.00	0.00	0.00	0.00	0.00	0.00	0.00	0.00	0.00	0.00	0.00	0.00	0.00
GH97	0.82	0.38	0.52	0.57	0.81	0.44	0.94	0.32	0.56	0.69	1.37	0.96	0.47	0.36	0.96	0.82
GH50	0.00	0.00	0.01	0.00	0.08	0.01	0.03	0.00	0.02	0.02	0.05	0.01	0.00	0.00	0.00	0.01
GT26	0.10	0.20	0.22	0.11	0.31	0.12	0.19	0.21	0.06	0.20	0.23	0.20	0.17	0.20	0.13	0.15
GH18	0.77	0.41	0.44	0.66	0.58	0.58	0.75	0.55	0.50	0.53	0.64	0.56	0.47	0.38	0.44	0.50
GH37	0.03	0.00	0.00	0.00	0.01	0.00	0.00	0.01	0.02	0.00	0.00	0.01	0.00	0.01	0.00	0.00
PL13	0.05	0.02	0.01	0.00	0.04	0.02	0.05	0.02	0.02	0.01	0.01	0.10	0.01	0.01	0.01	0.07
GH27	0.21	0.18	0.18	0.18	0.21	0.21	0.21	0.14	0.23	0.23	0.25	0.32	0.19	0.17	0.26	0.24
GT54	0.00	0.00	0.00	0.00	0.00	0.00	0.00	0.00	0.00	0.00	0.00	0.00	0.00	0.00	0.00	0.00
GT91	0.00	0.00	0.00	0.00	0.00	0.00	0.00	0.00	0.00	0.00	0.00	0.00	0.00	0.00	0.00	0.00
GT72	0.00	0.00	0.00	0.00	0.00	0.00	0.00	0.00	0.00	0.00	0.00	0.00	0.00	0.00	0.00	0.00
GH67	0.10	0.03	0.03	0.06	0.13	0.02	0.08	0.04	0.02	0.06	0.07	0.07	0.03	0.04	0.07	0.04
GH12	0.00	0.00	0.00	0.00	0.00	0.00	0.00	0.00	0.00	0.00	0.00	0.00	0.00	0.00	0.00	0.00
GH91	0.05	0.00	0.01	0.00	0.03	0.04	0.01	0.00	0.02	0.00	0.04	0.03	0.01	0.01	0.03	0.00
GT69	0.00	0.00	0.00	0.00	0.00	0.00	0.00	0.00	0.00	0.00	0.00	0.00	0.00	0.00	0.00	0.00
GH106	0.30	0.09	0.14	0.17	0.34	0.13	0.32	0.12	0.18	0.19	0.43	0.30	0.17	0.08	0.25	0.25
GT62	0.00	0.00	0.00	0.00	0.00	0.00	0.00	0.00	0.00	0.00	0.00	0.00	0.00	0.00	0.00	0.00
GH17	0.00	0.00	0.00	0.00	0.00	0.00	0.00	0.00	0.00	0.00	0.00	0.00	0.00	0.00	0.00	0.00
GH88	0.45	0.13	0.18	0.18	0.43	0.23	0.52	0.15	0.23	0.37	0.50	0.41	0.18	0.11	0.22	0.28
CE5	0.00	0.00	0.00	0.00	0.00	0.00	0.00	0.00	0.00	0.00	0.00	0.00	0.00	0.00	0.00	0.00
GT8	0.19	0.14	0.08	0.11	0.10	0.02	0.12	0.08	0.08	0.07	0.11	0.19	0.16	0.09	0.08	0.11
GT50	0.00	0.00	0.00	0.00	0.00	0.00	0.00	0.00	0.00	0.00	0.00	0.00	0.00	0.00	0.00	0.00
GT20	0.07	0.02	0.04	0.09	0.08	0.06	0.15	0.05	0.09	0.09	0.18	0.12	0.04	0.03	0.07	0.05
PL9	0.05	0.10	0.05	0.09	0.05	0.10	0.08	0.04	0.04	0.14	0.08	0.08	0.08	0.05	0.03	0.11
GH55	0.00	0.07	0.01	0.04	0.01	0.01	0.01	0.02	0.02	0.01	0.06	0.04	0.06	0.01	0.05	0.01
GH64	0.00	0.00	0.00	0.00	0.00	0.00	0.00	0.00	0.00	0.00	0.00	0.00	0.00	0.00	0.00	0.00
GH127	0.40	0.27	0.37	0.26	0.31	0.27	0.32	0.18	0.15	0.26	0.40	0.31	0.33	0.26	0.26	0.33
GH103	0.02	0.00	0.00	0.00	0.00	0.00	0.00	0.00	0.00	0.00	0.00	0.01	0.00	0.01	0.00	0.00
GH3	2.30	2.42	2.39	2.52	2.78	2.00	2.61	2.08	1.45	2.67	3.86	2.63	2.45	1.80	2.03	2.69
GT45	0.00	0.00	0.00	0.00	0.00	0.00	0.00	0.00	0.00	0.00	0.00	0.00	0.00	0.00	0.00	0.00
GT74	0.00	0.00	0.00	0.00	0.00	0.00	0.00	0.00	0.00	0.00	0.00	0.00	0.00	0.00	0.00	0.00
GT90	0.00	0.00	0.00	0.00	0.00	0.01	0.00	0.00	0.00	0.00	0.01	0.00	0.00	0.00	0.00	0.00
GH116	0.02	0.07	0.05	0.06	0.07	0.07	0.04	0.06	0.06	0.04	0.14	0.04	0.04	0.00	0.08	0.08
GH31	1.01	0.78	0.96	1.12	1.04	0.79	0.91	0.76	0.60	0.97	1.19	1.10	1.02	0.90	0.85	1.19
GT12	0.00	0.00	0.00	0.00	0.00	0.00	0.00	0.00	0.00	0.00	0.00	0.00	0.00	0.00	0.00	0.00
CE1	0.44	0.32	0.43	0.35	0.45	0.36	0.35	0.31	0.35	0.30	0.46	0.33	0.45	0.27	0.33	0.36
GH121	0.00	0.00	0.00	0.09	0.04	0.00	0.00	0.00	0.00	0.01	0.02	0.00	0.04	0.02	0.00	0.09
GH79	0.00	0.02	0.00	0.02	0.00	0.00	0.00	0.01	0.00	0.00	0.00	0.00	0.00	0.01	0.00	0.00
GT73	0.00	0.00	0.00	0.00	0.00	0.00	0.00	0.00	0.00	0.00	0.00	0.00	0.00	0.00	0.00	0.00
GH77	0.56	0.67	0.55	0.64	0.56	0.63	0.63	0.59	0.46	0.62	0.92	0.48	0.65	0.62	0.55	0.74
PL17	0.00	0.02	0.00	0.00	0.00	0.00	0.00	0.00	0.00	0.00	0.00	0.02	0.02	0.00	0.00	0.00
PL8	0.14	0.07	0.12	0.15	0.31	0.19	0.27	0.05	0.25	0.17	0.29	0.41	0.10	0.05	0.31	0.23

CAZY_group	ES-AD-20	ES-AD-21	ES-AD-22	ES-AD-23	ES-AD-24	ES-AD-25	ES-AD-26	ES-AD-27	ES-AD-28	ES-AD-29	ES-AD-3	ES-AD-30	ES-AD-31	ES-AD-32	ES-AD-33	ES-AD-34
**CE9**	0.52	0.31	0.33	0.42	0.41	0.33	0.43	0.30	0.12	0.30	0.60	0.35	0.28	0.27	0.27	0.48
**CE15**	0.00	0.00	0.00	0.00	0.00	0.00	0.01	0.01	0.01	0.01	0.02	0.03	0.00	0.00	0.01	0.00
**GT79**	0.00	0.00	0.00	0.00	0.00	0.00	0.00	0.00	0.00	0.00	0.00	0.00	0.00	0.00	0.00	0.00
**GT10**	0.00	0.00	0.01	0.00	0.00	0.00	0.01	0.00	0.00	0.02	0.00	0.00	0.00	0.02	0.00	0.00
**GH119**	0.00	0.00	0.00	0.00	0.00	0.00	0.00	0.00	0.00	0.00	0.00	0.00	0.00	0.00	0.00	0.00
**GH16**	0.14	0.08	0.12	0.13	0.18	0.13	0.17	0.12	0.22	0.16	0.34	0.25	0.12	0.11	0.30	0.12
**GH43**	2.44	1.10	1.50	1.95	2.56	1.57	2.14	1.16	1.22	1.71	2.68	2.04	1.68	1.19	1.97	2.06
**GH51**	0.57	0.36	0.56	0.63	0.64	0.42	0.48	0.31	0.32	0.43	0.70	0.55	0.44	0.56	0.51	0.66
**GT35**	0.71	1.09	1.13	0.81	0.84	0.89	0.86	0.86	0.46	0.95	1.49	0.56	0.80	0.88	1.02	0.70
**GT25**	0.00	0.00	0.01	0.00	0.00	0.00	0.00	0.00	0.00	0.00	0.00	0.01	0.00	0.00	0.01	0.00
**GT2**	3.85	3.77	3.96	4.16	3.81	3.86	4.55	3.48	3.28	4.31	7.37	4.08	4.20	3.69	3.63	4.23
**GT51**	0.97	0.72	0.75	1.03	0.80	0.87	0.91	0.63	0.70	0.74	1.21	0.98	0.80	0.76	0.89	0.91
**GH107**	0.00	0.00	0.00	0.00	0.00	0.00	0.00	0.00	0.00	0.00	0.00	0.00	0.00	0.00	0.00	0.00
**GH7**	0.00	0.00	0.00	0.00	0.00	0.00	0.00	0.00	0.00	0.00	0.00	0.00	0.00	0.00	0.00	0.00
**GT7**	0.00	0.00	0.00	0.00	0.00	0.00	0.00	0.00	0.00	0.00	0.00	0.00	0.00	0.00	0.00	0.00
**GT36**	0.00	0.00	0.00	0.00	0.00	0.00	0.00	0.00	0.00	0.00	0.00	0.00	0.00	0.00	0.00	0.00
**PL7**	0.02	0.00	0.00	0.00	0.00	0.00	0.00	0.00	0.00	0.00	0.00	0.00	0.00	0.00	0.00	0.00
**GH110**	0.05	0.04	0.12	0.07	0.14	0.04	0.11	0.08	0.10	0.09	0.11	0.14	0.06	0.08	0.14	0.12
**GT21**	0.00	0.00	0.00	0.00	0.00	0.00	0.00	0.00	0.00	0.00	0.00	0.00	0.00	0.00	0.00	0.00
**GH62**	0.00	0.00	0.00	0.00	0.00	0.00	0.00	0.00	0.00	0.00	0.00	0.00	0.00	0.00	0.00	0.00
**GH125**	0.21	0.08	0.08	0.18	0.14	0.10	0.21	0.12	0.06	0.18	0.25	0.23	0.14	0.09	0.16	0.21
**GH117**	0.09	0.04	0.06	0.11	0.10	0.05	0.08	0.04	0.08	0.08	0.13	0.11	0.03	0.03	0.09	0.07
**GH68**	0.03	0.00	0.00	0.00	0.00	0.00	0.00	0.00	0.00	0.00	0.00	0.00	0.00	0.00	0.00	0.00
**GH85**	0.05	0.00	0.03	0.07	0.01	0.00	0.00	0.00	0.01	0.00	0.06	0.00	0.03	0.03	0.00	0.03
**GH92**	2.09	0.64	0.72	1.21	1.93	0.86	1.85	0.70	1.10	1.45	2.23	1.45	1.01	0.76	1.16	1.82
**GH40**	0.00	0.00	0.00	0.00	0.00	0.00	0.00	0.00	0.00	0.00	0.00	0.00	0.00	0.00	0.00	0.00
**GT49**	0.00	0.00	0.00	0.00	0.00	0.00	0.00	0.00	0.00	0.00	0.00	0.00	0.00	0.00	0.00	0.00
**GH112**	0.40	0.16	0.22	0.33	0.17	0.18	0.20	0.13	0.05	0.13	0.36	0.11	0.14	0.23	0.13	0.21
**GT67**	0.00	0.00	0.00	0.00	0.00	0.00	0.00	0.00	0.00	0.00	0.00	0.00	0.00	0.00	0.00	0.00
**CE8**	0.12	0.18	0.11	0.28	0.16	0.15	0.28	0.16	0.11	0.23	0.25	0.13	0.20	0.14	0.25	0.26
**GT86**	0.00	0.00	0.00	0.00	0.00	0.00	0.00	0.00	0.00	0.00	0.00	0.00	0.00	0.00	0.00	0.00
**GH101**	0.07	0.00	0.02	0.07	0.01	0.01	0.01	0.00	0.01	0.04	0.02	0.00	0.04	0.06	0.03	0.03
**GH81**	0.00	0.00	0.00	0.00	0.00	0.01	0.00	0.00	0.00	0.00	0.00	0.00	0.00	0.04	0.00	0.00
**GT93**	0.00	0.00	0.00	0.00	0.00	0.00	0.00	0.00	0.00	0.00	0.00	0.00	0.00	0.00	0.00	0.00
**GT63**	0.00	0.00	0.00	0.00	0.00	0.00	0.00	0.00	0.00	0.00	0.00	0.00	0.00	0.00	0.00	0.00
**CE11**	0.24	0.15	0.24	0.15	0.25	0.16	0.29	0.18	0.28	0.17	0.29	0.21	0.20	0.16	0.27	0.21
**GT19**	0.12	0.07	0.12	0.09	0.14	0.07	0.19	0.12	0.11	0.12	0.24	0.19	0.12	0.06	0.16	0.13
**GT33**	0.00	0.00	0.00	0.00	0.00	0.00	0.00	0.00	0.00	0.00	0.00	0.00	0.00	0.00	0.00	0.00
**GH21**	0.00	0.00	0.00	0.00	0.00	0.00	0.00	0.00	0.00	0.00	0.00	0.00	0.00	0.00	0.00	0.00
**GT68**	0.00	0.00	0.00	0.00	0.00	0.00	0.00	0.00	0.00	0.00	0.00	0.00	0.00	0.00	0.00	0.00
**GH89**	0.26	0.09	0.12	0.07	0.31	0.18	0.27	0.09	0.19	0.19	0.25	0.21	0.11	0.10	0.26	0.18
**GT82**	0.00	0.00	0.00	0.00	0.00	0.00	0.00	0.00	0.00	0.00	0.00	0.00	0.00	0.00	0.00	0.00
**GH30**	0.31	0.13	0.17	0.28	0.24	0.15	0.19	0.11	0.20	0.16	0.18	0.24	0.16	0.11	0.21	0.32
**GH20**	0.89	0.54	0.98	0.81	1.43	0.75	1.43	0.79	0.98	1.01	1.79	1.32	0.69	0.58	0.96	1.19
**GH52**	0.00	0.00	0.00	0.00	0.00	0.00	0.00	0.00	0.00	0.00	0.00	0.00	0.00	0.00	0.00	0.00
**GT84**	0.00	0.01	0.05	0.00	0.00	0.02	0.00	0.04	0.01	0.04	0.00	0.00	0.06	0.01	0.00	0.00
**GT15**	0.00	0.00	0.00	0.00	0.00	0.00	0.00	0.00	0.00	0.00	0.00	0.00	0.00	0.00	0.00	0.00
**GH93**	0.02	0.00	0.00	0.00	0.00	0.02	0.03	0.00	0.00	0.00	0.00	0.02	0.02	0.00	0.00	0.00
**PL16**	0.00	0.00	0.00	0.00	0.00	0.00	0.00	0.00	0.00	0.00	0.00	0.00	0.00	0.00	0.00	0.00
**GH26**	0.16	0.11	0.17	0.15	0.12	0.22	0.09	0.06	0.10	0.18	0.11	0.11	0.16	0.08	0.23	0.08

CAZY_group	ES-AD-20	ES-AD-21	ES-AD-22	ES-AD-23	ES-AD-24	ES-AD-25	ES-AD-26	ES-AD-27	ES-AD-28	ES-AD-29	ES-AD-3	ES-AD-30	ES-AD-31	ES-AD-32	ES-AD-33	ES-AD-34
GT75	0.00	0.00	0.00	0.00	0.00	0.00	0.00	0.00	0.00	0.00	0.00	0.00	0.00	0.00	0.00	0.00
GH53	0.05	0.18	0.12	0.22	0.10	0.19	0.04	0.11	0.02	0.16	0.08	0.05	0.22	0.08	0.13	0.12
GT88	0.00	0.00	0.00	0.00	0.00	0.00	0.00	0.00	0.00	0.00	0.00	0.00	0.00	0.00	0.00	0.00
GT24	0.00	0.00	0.00	0.00	0.00	0.00	0.00	0.00	0.00	0.00	0.00	0.00	0.00	0.00	0.00	0.00
GT81	0.00	0.00	0.00	0.00	0.00	0.00	0.00	0.00	0.00	0.00	0.00	0.00	0.00	0.00	0.00	0.00
GH4	0.21	0.10	0.09	0.06	0.13	0.15	0.16	0.14	0.13	0.05	0.20	0.16	0.22	0.22	0.16	0.18
GT78	0.00	0.00	0.00	0.00	0.00	0.00	0.00	0.00	0.00	0.00	0.00	0.00	0.00	0.00	0.00	0.00
GH8	0.09	0.08	0.07	0.02	0.12	0.07	0.01	0.03	0.00	0.08	0.06	0.03	0.04	0.05	0.05	0.02
GH70	0.26	0.00	0.00	0.00	0.00	0.00	0.00	0.00	0.00	0.00	0.04	0.00	0.00	0.00	0.00	0.00
GH122	0.00	0.00	0.00	0.00	0.00	0.00	0.00	0.00	0.00	0.00	0.00	0.00	0.00	0.00	0.00	0.00
GH74	0.00	0.05	0.04	0.00	0.00	0.04	0.03	0.00	0.01	0.02	0.00	0.00	0.01	0.04	0.04	0.00
GT41	0.00	0.00	0.02	0.02	0.01	0.00	0.01	0.00	0.00	0.00	0.00	0.00	0.03	0.00	0.01	0.00
PL1	0.33	0.22	0.12	0.37	0.28	0.18	0.32	0.22	0.11	0.24	0.36	0.26	0.21	0.11	0.14	0.25
GH15	0.05	0.00	0.04	0.02	0.07	0.02	0.09	0.05	0.05	0.07	0.16	0.07	0.04	0.02	0.07	0.05
GH32	0.92	0.45	0.27	0.57	0.47	0.47	0.58	0.22	0.24	0.33	0.71	0.31	0.61	0.38	0.33	0.54
GH44	0.00	0.00	0.00	0.00	0.00	0.00	0.00	0.00	0.01	0.00	0.00	0.00	0.00	0.01	0.00	0.00
GT6	0.00	0.01	0.00	0.00	0.00	0.00	0.00	0.01	0.00	0.00	0.00	0.00	0.00	0.00	0.00	0.00
GH13	2.89	3.55	3.20	3.66	3.46	3.47	2.92	2.62	2.44	2.91	3.95	2.85	3.62	3.34	2.85	3.51
GT80	0.03	0.00	0.01	0.00	0.01	0.00	0.01	0.01	0.03	0.01	0.01	0.01	0.01	0.01	0.00	0.00
GH71	0.00	0.00	0.00	0.00	0.00	0.00	0.00	0.00	0.00	0.00	0.00	0.00	0.00	0.00	0.00	0.00
GH63	0.12	0.09	0.12	0.09	0.10	0.02	0.15	0.06	0.09	0.07	0.14	0.10	0.09	0.05	0.12	0.07
PL21	0.00	0.02	0.00	0.06	0.04	0.00	0.11	0.02	0.01	0.03	0.00	0.09	0.01	0.00	0.00	0.00
GH65	0.19	0.15	0.13	0.09	0.14	0.07	0.11	0.14	0.09	0.11	0.19	0.19	0.09	0.08	0.05	0.10
CE4	0.59	0.74	0.76	0.55	0.52	0.58	0.52	0.55	0.50	0.69	0.71	0.53	0.59	0.58	0.57	0.56
CE14	0.03	0.02	0.01	0.02	0.03	0.02	0.03	0.02	0.02	0.01	0.01	0.02	0.01	0.01	0.01	0.02
GH115	0.35	0.11	0.14	0.20	0.24	0.11	0.31	0.11	0.08	0.25	0.19	0.22	0.15	0.13	0.35	0.22
GH104	0.00	0.00	0.00	0.00	0.00	0.00	0.00	0.00	0.00	0.00	0.00	0.00	0.00	0.00	0.00	0.00
GH111	0.00	0.00	0.00	0.00	0.00	0.00	0.00	0.00	0.00	0.00	0.00	0.00	0.00	0.00	0.00	0.00
GH95	0.59	0.33	0.36	0.24	0.63	0.35	0.60	0.33	0.36	0.45	0.89	0.56	0.29	0.24	0.59	0.56
GT70	0.00	0.00	0.00	0.00	0.00	0.00	0.00	0.00	0.00	0.00	0.00	0.00	0.00	0.00	0.00	0.00
CE10	0.12	0.07	0.12	0.11	0.12	0.13	0.12	0.07	0.13	0.14	0.36	0.23	0.11	0.13	0.18	0.21
GH10	0.09	0.15	0.08	0.13	0.21	0.09	0.08	0.09	0.06	0.18	0.12	0.08	0.09	0.02	0.08	0.04
GH130	0.30	0.29	0.27	0.24	0.33	0.29	0.33	0.26	0.19	0.36	0.41	0.26	0.18	0.24	0.21	0.28
PL20	0.00	0.00	0.00	0.00	0.00	0.00	0.00	0.00	0.00	0.00	0.00	0.00	0.00	0.00	0.00	0.00
GH69	0.00	0.00	0.00	0.00	0.00	0.00	0.00	0.00	0.00	0.00	0.00	0.00	0.00	0.00	0.00	0.00
PL2	0.00	0.00	0.00	0.00	0.00	0.00	0.00	0.00	0.00	0.00	0.00	0.00	0.00	0.00	0.00	0.00
GH49	0.00	0.00	0.00	0.00	0.00	0.00	0.00	0.00	0.00	0.00	0.00	0.00	0.00	0.00	0.00	0.00
GH25	0.45	0.39	0.43	0.55	0.59	0.48	0.39	0.32	0.29	0.45	0.53	0.26	0.51	0.30	0.40	0.34
GH59	0.00	0.06	0.04	0.00	0.01	0.05	0.03	0.00	0.07	0.01	0.00	0.00	0.01	0.03	0.01	0.00
GH35	0.30	0.13	0.32	0.20	0.34	0.24	0.25	0.26	0.26	0.37	0.48	0.42	0.17	0.20	0.30	0.43
GT39	0.00	0.01	0.00	0.00	0.00	0.00	0.00	0.00	0.00	0.01	0.00	0.01	0.00	0.01	0.00	0.00
GT57	0.00	0.00	0.00	0.00	0.00	0.00	0.00	0.00	0.00	0.00	0.00	0.00	0.00	0.00	0.00	0.00
GH129	0.12	0.03	0.01	0.02	0.00	0.02	0.05	0.02	0.00	0.00	0.02	0.01	0.04	0.10	0.00	0.05
GT22	0.00	0.00	0.00	0.00	0.00	0.00	0.00	0.00	0.00	0.00	0.00	0.00	0.00	0.00	0.00	0.00
PL12	0.10	0.07	0.03	0.09	0.10	0.09	0.11	0.04	0.07	0.10	0.06	0.14	0.03	0.03	0.04	0.08
PL18	0.00	0.00	0.00	0.00	0.00	0.00	0.00	0.00	0.00	0.00	0.00	0.00	0.00	0.00	0.00	0.00
GT92	0.00	0.00	0.00	0.00	0.00	0.00	0.00	0.00	0.00	0.00	0.00	0.00	0.00	0.00	0.00	0.00
GT44	0.02	0.00	0.00	0.02	0.00	0.00	0.01	0.00	0.00	0.00	0.00	0.00	0.00	0.00	0.00	0.00
GT29	0.00	0.00	0.00	0.00	0.00	0.00	0.01	0.00	0.00	0.00	0.00	0.00	0.00	0.00	0.00	0.00
GT13	0.00	0.00	0.00	0.00	0.00	0.00	0.00	0.00	0.00	0.00	0.00	0.00	0.00	0.00	0.00	0.00

CAZY_group	ES-AD-20	ES-AD-21	ES-AD-22	ES-AD-23	ES-AD-24	ES-AD-25	ES-AD-26	ES-AD-27	ES-AD-28	ES-AD-29	ES-AD-3	ES-AD-30	ES-AD-31	ES-AD-32	ES-AD-33	ES-AD-34
**GT52**	0.00	0.00	0.00	0.00	0.00	0.00	0.00	0.00	0.00	0.00	0.00	0.00	0.00	0.00	0.00	0.00
**GT64**	0.00	0.00	0.00	0.00	0.00	0.00	0.00	0.00	0.00	0.00	0.00	0.00	0.00	0.00	0.00	0.00
**GT85**	0.00	0.00	0.00	0.00	0.00	0.00	0.00	0.00	0.00	0.00	0.00	0.00	0.00	0.00	0.00	0.00
**GT16**	0.00	0.00	0.00	0.00	0.00	0.00	0.00	0.00	0.00	0.00	0.00	0.00	0.00	0.00	0.00	0.00
**GH56**	0.00	0.00	0.00	0.00	0.00	0.00	0.00	0.00	0.00	0.00	0.00	0.00	0.00	0.00	0.00	0.00
**GT60**	0.00	0.00	0.00	0.00	0.00	0.00	0.00	0.00	0.00	0.00	0.00	0.00	0.00	0.00	0.00	0.00
**GH41**	0.00	0.00	0.00	0.00	0.00	0.00	0.00	0.00	0.00	0.00	0.00	0.00	0.00	0.00	0.00	0.00
**CE7**	0.12	0.07	0.07	0.09	0.13	0.04	0.07	0.05	0.05	0.13	0.11	0.13	0.11	0.07	0.12	0.14
**GH39**	0.05	0.08	0.03	0.06	0.05	0.05	0.00	0.04	0.00	0.06	0.04	0.01	0.05	0.06	0.05	0.00
**GT30**	0.26	0.18	0.21	0.24	0.25	0.18	0.29	0.17	0.31	0.24	0.35	0.24	0.19	0.12	0.31	0.28
**PL15**	0.10	0.02	0.02	0.06	0.12	0.07	0.13	0.02	0.03	0.06	0.16	0.19	0.03	0.03	0.03	0.15
**GH102**	0.00	0.02	0.06	0.02	0.00	0.01	0.05	0.01	0.00	0.05	0.04	0.01	0.02	0.02	0.03	0.01

CAZY_group	ES-AD-35	ES-AD-36	ES-AD-37	ES-AD-38	ES-AD-39	ES-AD-4	ES-AD-5	ES-AD-6	ES-AD-7	ES-AD-8	ES-AD-9	FR-AD-1	FR-AD-2	FR-AD-3	FR-AD-4	FR-AD-5
GH29	0.71	0.82	1.03	0.81	0.55	0.80	0.57	0.51	0.63	0.85	0.49	0.87	0.90	1.39	0.87	0.64
PL4	0.00	0.00	0.00	0.00	0.00	0.00	0.00	0.00	0.00	0.00	0.00	0.00	0.00	0.00	0.00	0.00
GH23	0.94	1.16	1.23	0.96	1.06	1.81	1.06	0.75	0.86	1.40	1.08	1.61	1.86	1.67	1.55	1.62
CE6	0.00	0.06	0.00	0.01	0.02	0.03	0.00	0.03	0.01	0.03	0.00	0.00	0.02	0.05	0.00	0.01
GH72	0.07	0.09	0.07	0.11	0.11	0.11	0.16	0.07	0.07	0.16	0.12	0.24	0.20	0.18	0.18	0.13
GH114	0.00	0.00	0.00	0.00	0.00	0.00	0.00	0.00	0.00	0.00	0.00	0.00	0.00	0.00	0.00	0.00
GH78	0.47	0.69	0.89	0.67	0.49	1.40	0.33	0.38	0.47	0.88	0.15	0.55	1.04	1.29	0.85	0.98
PL19	0.00	0.00	0.00	0.00	0.00	0.00	0.00	0.00	0.00	0.00	0.00	0.00	0.00	0.00	0.00	0.00
GT27	0.00	0.00	0.00	0.00	0.00	0.00	0.00	0.00	0.00	0.00	0.00	0.00	0.00	0.00	0.00	0.00
GH5	0.35	0.74	0.24	0.43	0.34	0.42	0.18	0.56	0.61	0.31	0.86	0.58	0.55	0.65	0.58	0.46
GT40	0.00	0.00	0.00	0.00	0.00	0.00	0.00	0.00	0.00	0.00	0.00	0.00	0.00	0.00	0.00	0.00
CE13	0.00	0.00	0.00	0.00	0.00	0.00	0.00	0.00	0.00	0.00	0.00	0.00	0.00	0.00	0.00	0.00
GT37	0.00	0.00	0.00	0.00	0.00	0.00	0.00	0.00	0.00	0.00	0.00	0.00	0.00	0.00	0.00	0.00
GH33	0.15	0.18	0.27	0.19	0.20	0.21	0.10	0.12	0.17	0.21	0.03	0.26	0.30	0.39	0.26	0.14
GT65	0.00	0.00	0.00	0.00	0.00	0.00	0.00	0.00	0.00	0.00	0.00	0.00	0.00	0.00	0.00	0.00
GH86	0.00	0.00	0.00	0.00	0.01	0.00	0.00	0.00	0.00	0.00	0.00	0.01	0.00	0.02	0.00	0.00
GH123	0.10	0.08	0.25	0.09	0.05	0.17	0.02	0.05	0.09	0.16	0.06	0.13	0.15	0.22	0.11	0.07
GH96	0.00	0.00	0.00	0.00	0.00	0.00	0.00	0.00	0.00	0.00	0.00	0.00	0.00	0.00	0.00	0.00
GH14	0.00	0.00	0.00	0.00	0.00	0.00	0.00	0.00	0.00	0.00	0.00	0.00	0.00	0.00	0.00	0.00
CE3	0.00	0.00	0.00	0.00	0.00	0.00	0.00	0.00	0.00	0.00	0.00	0.00	0.00	0.00	0.00	0.00
PL10	0.04	0.12	0.20	0.09	0.03	0.12	0.14	0.10	0.09	0.21	0.18	0.07	0.17	0.37	0.11	0.20
GT48	0.00	0.00	0.00	0.00	0.00	0.00	0.00	0.00	0.00	0.00	0.00	0.00	0.00	0.00	0.00	0.00
PL6	0.00	0.00	0.00	0.01	0.00	0.00	0.00	0.00	0.00	0.00	0.00	0.00	0.02	0.00	0.00	0.00
GT83	0.05	0.05	0.25	0.05	0.04	0.03	0.10	0.05	0.02	0.13	0.00	0.06	0.13	0.08	0.11	0.01
GH126	0.00	0.00	0.00	0.00	0.00	0.00	0.00	0.00	0.00	0.00	0.00	0.00	0.00	0.00	0.00	0.00
GH9	0.11	0.36	0.07	0.13	0.26	0.25	0.00	0.34	0.09	0.54	0.52	0.27	0.42	0.55	0.27	0.09
GH75	0.00	0.00	0.00	0.00	0.00	0.00	0.00	0.00	0.00	0.00	0.00	0.00	0.00	0.00	0.00	0.00
GT71	0.00	0.00	0.00	0.00	0.00	0.00	0.00	0.00	0.00	0.00	0.00	0.00	0.00	0.00	0.00	0.00
GH46	0.00	0.00	0.00	0.00	0.00	0.00	0.00	0.00	0.00	0.00	0.00	0.00	0.00	0.00	0.00	0.00
GT61	0.00	0.00	0.00	0.00	0.00	0.00	0.00	0.00	0.00	0.00	0.00	0.00	0.00	0.00	0.00	0.00
GT3	0.13	0.16	0.14	0.08	0.13	0.34	0.06	0.11	0.12	0.23	0.15	0.20	0.23	0.40	0.16	0.20
PL3	0.00	0.00	0.00	0.00	0.00	0.00	0.02	0.00	0.00	0.00	0.00	0.00	0.00	0.00	0.00	0.00
GH108	0.04	0.05	0.04	0.03	0.03	0.01	0.04	0.03	0.04	0.08	0.00	0.01	0.03	0.07	0.01	0.03
GT56	0.00	0.01	0.00	0.00	0.00	0.00	0.00	0.00	0.00	0.00	0.00	0.00	0.00	0.00	0.01	0.00
PL11	0.09	0.12	0.09	0.05	0.03	0.16	0.02	0.07	0.11	0.13	0.00	0.16	0.10	0.25	0.06	0.16
GH45	0.00	0.00	0.02	0.00	0.00	0.01	0.02	0.00	0.00	0.00	0.00	0.00	0.00	0.02	0.00	0.00
GT34	0.00	0.00	0.00	0.00	0.00	0.00	0.00	0.00	0.00	0.00	0.00	0.01	0.00	0.00	0.00	0.01
PL14	0.00	0.00	0.00	0.00	0.00	0.00	0.00	0.00	0.00	0.00	0.00	0.00	0.00	0.00	0.00	0.00
GH19	0.01	0.06	0.00	0.00	0.01	0.00	0.00	0.01	0.00	0.03	0.00	0.00	0.00	0.02	0.00	0.00
GH118	0.00	0.00	0.00	0.00	0.00	0.00	0.00	0.00	0.00	0.00	0.00	0.00	0.00	0.00	0.00	0.00
GH82	0.00	0.00	0.00	0.00	0.00	0.00	0.00	0.00	0.00	0.00	0.00	0.00	0.00	0.00	0.00	0.00
GH76	0.08	0.18	0.18	0.10	0.04	0.28	0.04	0.08	0.01	0.16	0.00	0.06	0.13	0.28	0.03	0.10
GT4	1.65	1.82	1.86	1.59	1.53	2.87	1.61	1.81	1.66	2.07	1.63	2.12	2.86	3.01	2.97	2.47
GT87	0.01	0.00	0.02	0.00	0.00	0.00	0.00	0.00	0.00	0.00	0.00	0.01	0.02	0.00	0.00	0.00
GH24	0.13	0.09	0.11	0.05	0.06	0.12	0.14	0.08	0.16	0.18	0.12	0.23	0.18	0.12	0.18	0.14
GH90	0.00	0.00	0.00	0.00	0.00	0.00	0.00	0.00	0.00	0.00	0.00	0.00	0.00	0.00	0.00	0.00
GH57	0.14	0.10	0.13	0.06	0.18	0.19	0.08	0.11	0.09	0.23	0.12	0.11	0.17	0.23	0.11	0.10
GH36	0.69	0.66	0.61	0.64	0.50	0.94	0.73	0.67	0.64	0.72	0.74	0.94	0.95	1.16	1.18	0.95
GT46	0.00	0.00	0.00	0.00	0.00	0.00	0.00	0.00	0.00	0.00	0.00	0.00	0.00	0.00	0.00	0.00
GH66	0.00	0.06	0.02	0.02	0.01	0.05	0.00	0.03	0.00	0.08	0.00	0.01	0.10	0.02	0.03	0.06

CAZY_group	ES-AD-35	ES-AD-36	ES-AD-37	ES-AD-38	ES-AD-39	ES-AD-4	ES-AD-5	ES-AD-6	ES-AD-7	ES-AD-8	ES-AD-9	FR-AD-1	FR-AD-2	FR-AD-3	FR-AD-4	FR-AD-5
GH48	0.00	0.00	0.00	0.01	0.00	0.00	0.00	0.01	0.00	0.00	0.00	0.01	0.00	0.02	0.00	0.00
GT53	0.00	0.00	0.00	0.00	0.00	0.00	0.00	0.00	0.00	0.00	0.00	0.00	0.00	0.00	0.00	0.00
GH60	0.00	0.00	0.00	0.00	0.00	0.00	0.00	0.00	0.00	0.00	0.00	0.00	0.00	0.00	0.00	0.00
GT31	0.00	0.01	0.00	0.00	0.00	0.00	0.00	0.00	0.00	0.00	0.00	0.00	0.00	0.00	0.00	0.00
GH105	0.37	0.50	0.49	0.30	0.25	0.68	0.31	0.16	0.28	0.54	0.25	0.33	0.49	0.94	0.67	0.62
GT23	0.01	0.02	0.00	0.01	0.01	0.00	0.00	0.00	0.00	0.00	0.00	0.00	0.02	0.03	0.01	0.00
GH11	0.01	0.00	0.00	0.00	0.01	0.01	0.00	0.01	0.02	0.00	0.00	0.00	0.00	0.02	0.00	0.00
GT38	0.00	0.00	0.00	0.00	0.00	0.00	0.00	0.00	0.00	0.00	0.00	0.00	0.00	0.00	0.00	0.00
GT43	0.00	0.00	0.00	0.00	0.00	0.00	0.00	0.00	0.00	0.00	0.00	0.00	0.00	0.00	0.00	0.00
GH99	0.00	0.00	0.00	0.00	0.00	0.02	0.00	0.00	0.00	0.00	0.03	0.01	0.00	0.00	0.00	0.00
GH38	0.14	0.24	0.16	0.20	0.23	0.51	0.14	0.16	0.23	0.13	0.12	0.23	0.25	0.25	0.31	0.40
GH109	0.24	0.26	0.42	0.21	0.23	0.38	0.06	0.14	0.22	0.44	0.18	0.30	0.28	0.87	0.33	0.20
GH2	3.15	4.04	4.12	2.86	2.54	5.51	2.63	2.74	2.55	3.90	2.34	4.25	5.19	6.91	5.06	3.83
GT28	0.41	0.41	0.38	0.51	0.48	0.65	0.47	0.31	0.46	0.28	0.52	0.64	0.62	0.47	0.48	0.51
GH42	0.21	0.13	0.20	0.28	0.23	0.19	0.59	0.10	0.15	0.08	0.18	0.21	0.20	0.33	0.53	0.39
GT58	0.00	0.00	0.00	0.00	0.00	0.00	0.00	0.00	0.00	0.00	0.00	0.00	0.00	0.00	0.00	0.00
GH61	0.00	0.00	0.00	0.00	0.00	0.00	0.00	0.00	0.00	0.00	0.00	0.00	0.00	0.00	0.00	0.00
GH80	0.00	0.00	0.00	0.00	0.00	0.00	0.00	0.00	0.00	0.00	0.00	0.00	0.00	0.00	0.00	0.00
GH98	0.00	0.01	0.00	0.00	0.00	0.00	0.00	0.03	0.00	0.00	0.00	0.03	0.00	0.00	0.00	0.00
GH6	0.00	0.00	0.00	0.00	0.00	0.00	0.00	0.00	0.00	0.00	0.00	0.00	0.00	0.00	0.00	0.00
GT1	0.04	0.06	0.04	0.04	0.03	0.03	0.04	0.00	0.03	0.03	0.12	0.03	0.03	0.07	0.09	0.07
GH28	0.49	0.76	0.54	0.41	0.50	0.99	0.45	0.33	0.60	0.96	0.55	0.78	1.04	1.29	1.10	0.91
GT42	0.00	0.00	0.00	0.00	0.00	0.00	0.00	0.00	0.00	0.00	0.00	0.00	0.00	0.00	0.00	0.00
GH128	0.00	0.00	0.00	0.00	0.00	0.00	0.00	0.00	0.00	0.00	0.00	0.00	0.00	0.00	0.00	0.00
CE12	0.06	0.12	0.05	0.02	0.03	0.13	0.08	0.15	0.14	0.16	0.09	0.06	0.13	0.17	0.14	0.10
CE16	0.00	0.00	0.00	0.00	0.00	0.00	0.00	0.00	0.00	0.00	0.00	0.00	0.00	0.00	0.00	0.00
GT89	0.00	0.00	0.00	0.00	0.00	0.00	0.00	0.00	0.00	0.00	0.00	0.00	0.00	0.00	0.00	0.00
GT11	0.04	0.09	0.04	0.04	0.03	0.06	0.02	0.03	0.11	0.05	0.06	0.06	0.08	0.15	0.07	0.04
GT77	0.00	0.00	0.02	0.01	0.00	0.01	0.00	0.00	0.00	0.00	0.00	0.00	0.00	0.00	0.01	0.00
GT55	0.00	0.00	0.00	0.00	0.00	0.00	0.00	0.00	0.00	0.00	0.00	0.00	0.00	0.00	0.00	0.00
GT18	0.00	0.00	0.00	0.00	0.00	0.00	0.00	0.00	0.00	0.00	0.00	0.00	0.00	0.00	0.00	0.00
GH87	0.00	0.05	0.00	0.00	0.00	0.01	0.00	0.00	0.00	0.00	0.03	0.00	0.00	0.00	0.00	0.00
PL5	0.00	0.00	0.00	0.00	0.00	0.00	0.00	0.00	0.00	0.00	0.00	0.00	0.00	0.00	0.00	0.00
GH22	0.00	0.00	0.00	0.00	0.00	0.00	0.00	0.00	0.00	0.00	0.00	0.00	0.00	0.00	0.00	0.00
CE2	0.06	0.06	0.02	0.08	0.08	0.06	0.04	0.03	0.07	0.00	0.12	0.06	0.03	0.05	0.07	0.09
GH120	0.08	0.07	0.07	0.10	0.06	0.05	0.10	0.07	0.04	0.03	0.03	0.04	0.03	0.05	0.11	0.10
GH73	0.39	0.48	0.47	0.52	0.48	0.43	0.55	0.40	0.38	0.54	0.61	0.50	0.40	0.47	0.44	0.46
GT17	0.00	0.02	0.00	0.00	0.00	0.00	0.00	0.00	0.00	0.00	0.00	0.00	0.00	0.00	0.00	0.00
GH124	0.00	0.00	0.00	0.00	0.00	0.00	0.00	0.00	0.00	0.00	0.00	0.00	0.00	0.00	0.00	0.00
GH54	0.00	0.00	0.00	0.00	0.00	0.00	0.00	0.00	0.00	0.00	0.00	0.00	0.00	0.00	0.00	0.00
GH100	0.00	0.00	0.00	0.00	0.00	0.00	0.00	0.00	0.00	0.00	0.00	0.00	0.00	0.00	0.00	0.00
GH113	0.05	0.05	0.00	0.02	0.01	0.00	0.04	0.01	0.04	0.00	0.00	0.03	0.00	0.03	0.03	0.00
GT5	0.47	0.46	0.40	0.53	0.53	0.65	0.55	0.42	0.46	0.41	0.68	0.61	0.57	0.52	0.51	0.65
GH94	0.56	0.51	0.20	0.37	0.33	0.53	0.57	0.33	0.51	0.18	1.05	0.54	0.49	0.54	0.74	0.58
GT66	0.00	0.00	0.00	0.01	0.00	0.00	0.00	0.03	0.00	0.05	0.00	0.00	0.00	0.00	0.00	0.04
GT94	0.00	0.00	0.00	0.00	0.00	0.00	0.00	0.00	0.00	0.00	0.00	0.00	0.00	0.00	0.00	0.00
GH58	0.00	0.00	0.00	0.00	0.00	0.00	0.00	0.00	0.00	0.00	0.00	0.00	0.00	0.00	0.00	0.00
GH1	0.33	0.69	0.33	0.75	0.39	0.58	1.31	0.93	0.43	0.18	0.77	0.68	0.40	0.33	0.70	0.85
PL22	0.00	0.02	0.00	0.00	0.00	0.00	0.00	0.00	0.00	0.00	0.00	0.00	0.00	0.00	0.00	0.00
GT9	0.12	0.13	0.22	0.05	0.05	0.09	0.08	0.14	0.15	0.23	0.12	0.17	0.20	0.18	0.16	0.10

CAZY_group	ES-AD-35	ES-AD-36	ES-AD-37	ES-AD-38	ES-AD-39	ES-AD-4	ES-AD-5	ES-AD-6	ES-AD-7	ES-AD-8	ES-AD-9	FR-AD-1	FR-AD-2	FR-AD-3	FR-AD-4	FR-AD-5
GT76	0.00	0.00	0.00	0.00	0.00	0.00	0.00	0.00	0.00	0.00	0.00	0.00	0.00	0.00	0.00	0.00
GT32	0.05	0.06	0.09	0.15	0.05	0.16	0.02	0.04	0.09	0.05	0.06	0.09	0.13	0.20	0.14	0.16
GT47	0.02	0.03	0.04	0.03	0.05	0.09	0.08	0.03	0.03	0.05	0.09	0.06	0.05	0.03	0.06	0.12
GH84	0.12	0.12	0.22	0.14	0.10	0.18	0.14	0.07	0.07	0.18	0.06	0.11	0.23	0.20	0.13	0.07
GH83	0.00	0.00	0.00	0.00	0.00	0.00	0.00	0.00	0.00	0.00	0.00	0.00	0.00	0.00	0.00	0.00
GT59	0.00	0.00	0.00	0.00	0.00	0.00	0.00	0.00	0.00	0.00	0.00	0.00	0.00	0.00	0.00	0.00
GH34	0.00	0.00	0.00	0.00	0.00	0.00	0.00	0.00	0.00	0.00	0.00	0.00	0.00	0.00	0.00	0.00
GT14	0.03	0.02	0.00	0.03	0.03	0.07	0.00	0.03	0.04	0.03	0.03	0.01	0.02	0.05	0.04	0.04
GH47	0.00	0.00	0.00	0.00	0.00	0.00	0.00	0.00	0.00	0.00	0.00	0.00	0.00	0.00	0.00	0.00
GH97	0.63	0.71	0.83	0.46	0.47	1.02	0.18	0.60	0.45	0.83	0.34	0.85	1.20	1.59	0.81	0.61
GH50	0.00	0.05	0.00	0.01	0.00	0.02	0.00	0.00	0.00	0.03	0.00	0.00	0.02	0.03	0.03	0.00
GT26	0.16	0.25	0.24	0.19	0.29	0.26	0.16	0.19	0.25	0.21	0.18	0.14	0.23	0.27	0.21	0.17
GH18	0.44	0.48	0.51	0.41	0.54	0.72	0.51	0.40	0.35	0.72	0.28	0.55	0.60	0.70	0.73	0.33
GH37	0.01	0.03	0.00	0.00	0.01	0.00	0.00	0.01	0.00	0.00	0.00	0.00	0.00	0.00	0.00	0.00
PL13	0.01	0.02	0.00	0.02	0.01	0.05	0.00	0.01	0.02	0.05	0.00	0.00	0.03	0.05	0.01	0.01
GH27	0.15	0.15	0.20	0.14	0.14	0.28	0.10	0.22	0.22	0.26	0.12	0.14	0.33	0.37	0.24	0.26
GT54	0.00	0.00	0.00	0.00	0.00	0.00	0.00	0.00	0.00	0.00	0.00	0.00	0.00	0.00	0.00	0.00
GT91	0.00	0.00	0.00	0.00	0.00	0.00	0.00	0.00	0.00	0.00	0.00	0.00	0.00	0.00	0.00	0.00
GT72	0.00	0.00	0.00	0.00	0.00	0.00	0.00	0.00	0.00	0.00	0.00	0.00	0.00	0.00	0.00	0.00
GH67	0.07	0.07	0.07	0.05	0.04	0.08	0.02	0.05	0.03	0.08	0.00	0.09	0.03	0.12	0.04	0.09
GH12	0.00	0.00	0.00	0.00	0.00	0.00	0.00	0.00	0.00	0.00	0.00	0.00	0.00	0.00	0.00	0.00
GH91	0.03	0.02	0.04	0.04	0.02	0.02	0.02	0.01	0.02	0.00	0.00	0.01	0.02	0.00	0.01	0.01
GT69	0.00	0.00	0.00	0.00	0.00	0.00	0.00	0.00	0.00	0.00	0.00	0.00	0.00	0.00	0.00	0.00
GH106	0.12	0.21	0.27	0.12	0.15	0.35	0.02	0.11	0.10	0.44	0.03	0.24	0.12	0.50	0.26	0.27
GT62	0.00	0.00	0.00	0.00	0.00	0.00	0.00	0.00	0.00	0.00	0.00	0.00	0.00	0.00	0.00	0.00
GH17	0.00	0.00	0.00	0.00	0.00	0.01	0.00	0.00	0.00	0.00	0.00	0.00	0.00	0.00	0.00	0.00
GH88	0.15	0.31	0.34	0.19	0.25	0.30	0.02	0.11	0.17	0.39	0.06	0.31	0.55	0.55	0.51	0.17
CE5	0.00	0.00	0.00	0.00	0.00	0.00	0.00	0.00	0.00	0.00	0.00	0.00	0.02	0.00	0.00	0.00
GT8	0.10	0.26	0.18	0.10	0.08	0.21	0.22	0.12	0.13	0.16	0.12	0.17	0.15	0.13	0.23	0.10
GT50	0.00	0.00	0.00	0.00	0.00	0.00	0.00	0.00	0.00	0.00	0.00	0.00	0.00	0.00	0.00	0.00
GT20	0.04	0.10	0.16	0.04	0.08	0.09	0.00	0.07	0.02	0.10	0.00	0.04	0.17	0.23	0.09	0.04
PL9	0.01	0.10	0.02	0.01	0.01	0.13	0.08	0.08	0.09	0.05	0.12	0.07	0.08	0.10	0.17	0.03
GH55	0.05	0.03	0.00	0.06	0.01	0.05	0.06	0.05	0.01	0.03	0.00	0.03	0.05	0.07	0.06	0.00
GH64	0.00	0.00	0.00	0.00	0.00	0.00	0.00	0.00	0.00	0.00	0.00	0.00	0.00	0.00	0.00	0.00
GH127	0.25	0.38	0.33	0.33	0.18	0.44	0.29	0.25	0.22	0.28	0.12	0.47	0.45	0.52	0.43	0.40
GH103	0.00	0.02	0.00	0.01	0.03	0.00	0.00	0.00	0.00	0.00	0.00	0.01	0.00	0.00	0.00	0.00
GH3	1.98	2.68	2.39	2.29	1.94	2.93	2.20	1.97	2.02	1.81	1.63	3.90	3.25	4.40	3.63	2.69
GT45	0.00	0.00	0.00	0.00	0.00	0.00	0.00	0.00	0.00	0.00	0.00	0.00	0.00	0.00	0.00	0.00
GT74	0.00	0.00	0.00	0.00	0.00	0.00	0.00	0.00	0.00	0.00	0.00	0.00	0.00	0.00	0.00	0.00
GT90	0.00	0.00	0.00	0.00	0.00	0.00	0.00	0.00	0.00	0.00	0.00	0.00	0.02	0.00	0.00	0.00
GH116	0.07	0.05	0.09	0.06	0.07	0.08	0.00	0.08	0.01	0.13	0.06	0.01	0.10	0.18	0.06	0.03
GH31	1.01	1.20	1.07	0.84	0.69	1.16	0.61	0.81	0.83	0.83	0.80	1.39	1.22	1.93	1.56	1.06
GT12	0.00	0.00	0.00	0.00	0.00	0.00	0.00	0.00	0.00	0.00	0.00	0.00	0.00	0.00	0.00	0.00
CE1	0.24	0.48	0.40	0.33	0.25	0.53	0.31	0.33	0.36	0.41	0.40	0.43	0.44	0.49	0.48	0.39
GH121	0.00	0.00	0.00	0.02	0.01	0.08	0.04	0.00	0.00	0.00	0.00	0.07	0.12	0.00	0.07	0.04
GH79	0.00	0.00	0.00	0.01	0.00	0.00	0.02	0.00	0.00	0.00	0.00	0.00	0.03	0.02	0.00	0.00
GT73	0.00	0.00	0.00	0.00	0.00	0.00	0.00	0.00	0.00	0.00	0.00	0.00	0.00	0.00	0.00	0.00
GH77	0.70	0.62	0.51	0.61	0.61	1.01	0.90	0.42	0.53	0.39	0.65	1.07	0.80	0.85	1.19	1.00
PL17	0.00	0.00	0.00	0.02	0.00	0.00	0.00	0.00	0.00	0.00	0.00	0.00	0.02	0.00	0.00	0.00
PL8	0.15	0.14	0.25	0.14	0.13	0.09	0.00	0.03	0.13	0.21	0.03	0.18	0.25	0.28	0.23	0.06

Normalized_abundance_2

CAZY_group	ES-AD-35	ES-AD-36	ES-AD-37	ES-AD-38	ES-AD-39	ES-AD-4	ES-AD-5	ES-AD-6	ES-AD-7	ES-AD-8	ES-AD-9	FR-AD-1	FR-AD-2	FR-AD-3	FR-AD-4	FR-AD-5
**CE9**	0.29	0.35	0.34	0.38	0.30	0.32	0.47	0.26	0.25	0.31	0.22	0.44	0.30	0.40	0.37	0.36
**CE15**	0.00	0.00	0.02	0.02	0.03	0.02	0.00	0.00	0.00	0.03	0.00	0.00	0.02	0.07	0.03	0.01
**GT79**	0.00	0.00	0.00	0.00	0.00	0.00	0.00	0.00	0.00	0.00	0.00	0.00	0.00	0.00	0.00	0.00
**GT10**	0.00	0.00	0.00	0.02	0.00	0.02	0.00	0.00	0.00	0.00	0.00	0.01	0.00	0.02	0.03	0.00
**GH119**	0.00	0.00	0.00	0.00	0.00	0.00	0.00	0.00	0.00	0.00	0.00	0.00	0.00	0.00	0.00	0.00
**GH16**	0.12	0.13	0.40	0.12	0.13	0.18	0.08	0.07	0.11	0.16	0.09	0.16	0.20	0.42	0.17	0.19
**GH43**	1.69	1.83	1.72	1.40	1.09	2.50	1.35	1.75	1.48	1.65	1.54	2.52	2.39	3.18	2.69	2.36
**GH51**	0.53	0.50	0.47	0.47	0.37	0.68	0.29	0.42	0.42	0.36	0.31	0.78	0.64	0.57	0.80	0.58
**GT35**	0.85	0.93	0.67	0.92	0.82	1.30	1.08	0.88	0.98	0.54	0.89	1.48	1.57	1.42	1.58	1.40
**GT25**	0.01	0.02	0.00	0.00	0.00	0.00	0.00	0.00	0.01	0.00	0.00	0.00	0.00	0.00	0.00	0.00
**GT2**	3.57	4.04	4.63	3.97	3.60	5.96	4.30	4.27	3.68	3.49	4.06	6.01	6.59	7.72	7.24	5.74
**GT51**	0.79	0.86	1.19	0.79	0.66	1.06	0.94	0.83	0.72	0.88	1.08	1.11	0.95	1.00	1.29	0.95
**GH107**	0.00	0.00	0.00	0.00	0.00	0.00	0.00	0.00	0.00	0.00	0.00	0.00	0.00	0.00	0.00	0.00
**GH7**	0.00	0.00	0.00	0.00	0.00	0.00	0.00	0.00	0.00	0.00	0.00	0.00	0.00	0.00	0.00	0.00
**GT7**	0.00	0.00	0.00	0.00	0.00	0.00	0.00	0.00	0.00	0.00	0.00	0.00	0.00	0.00	0.00	0.00
**GT36**	0.00	0.00	0.00	0.00	0.00	0.00	0.00	0.00	0.00	0.00	0.00	0.00	0.00	0.00	0.00	0.00
**PL7**	0.00	0.00	0.00	0.00	0.00	0.00	0.00	0.00	0.00	0.00	0.00	0.00	0.00	0.00	0.00	0.00
**GH110**	0.12	0.10	0.25	0.10	0.06	0.16	0.04	0.11	0.10	0.13	0.00	0.13	0.15	0.30	0.16	0.06
**GT21**	0.00	0.00	0.00	0.00	0.00	0.00	0.00	0.00	0.00	0.00	0.00	0.00	0.00	0.00	0.00	0.00
**GH62**	0.00	0.00	0.00	0.00	0.00	0.00	0.00	0.00	0.01	0.00	0.00	0.00	0.00	0.00	0.00	0.00
**GH125**	0.15	0.18	0.20	0.15	0.10	0.30	0.06	0.15	0.09	0.16	0.06	0.18	0.33	0.22	0.17	0.16
**GH117**	0.08	0.08	0.09	0.05	0.06	0.08	0.02	0.04	0.08	0.10	0.00	0.09	0.12	0.22	0.09	0.01
**GH68**	0.00	0.02	0.00	0.00	0.00	0.00	0.00	0.00	0.00	0.00	0.00	0.00	0.00	0.00	0.00	0.00
**GH85**	0.00	0.05	0.00	0.01	0.00	0.03	0.02	0.00	0.03	0.13	0.03	0.06	0.02	0.00	0.01	0.00
**GH92**	1.17	1.24	1.86	1.01	0.85	2.09	0.29	0.66	0.90	1.94	0.55	1.28	1.69	2.90	1.58	1.00
**GH40**	0.00	0.00	0.00	0.00	0.00	0.00	0.00	0.00	0.00	0.00	0.00	0.00	0.00	0.00	0.00	0.00
**GT49**	0.00	0.00	0.00	0.00	0.00	0.00	0.00	0.00	0.00	0.00	0.00	0.00	0.00	0.00	0.00	0.00
**GH112**	0.17	0.18	0.13	0.13	0.11	0.29	0.24	0.04	0.13	0.10	0.15	0.31	0.42	0.18	0.16	0.26
**GT67**	0.00	0.00	0.00	0.00	0.00	0.00	0.00	0.00	0.00	0.00	0.00	0.00	0.00	0.00	0.00	0.00
**CE8**	0.11	0.26	0.11	0.10	0.10	0.24	0.22	0.15	0.15	0.10	0.28	0.18	0.17	0.40	0.18	0.26
**GT86**	0.00	0.00	0.00	0.00	0.00	0.00	0.00	0.00	0.00	0.00	0.00	0.00	0.00	0.00	0.00	0.00
**GH101**	0.00	0.00	0.00	0.04	0.01	0.07	0.06	0.00	0.00	0.00	0.00	0.09	0.03	0.07	0.04	0.03
**GH81**	0.03	0.00	0.00	0.03	0.00	0.00	0.00	0.00	0.00	0.00	0.00	0.00	0.00	0.00	0.00	0.00
**GT93**	0.00	0.00	0.00	0.00	0.00	0.00	0.00	0.00	0.00	0.00	0.00	0.00	0.00	0.00	0.00	0.00
**GT63**	0.00	0.00	0.00	0.00	0.00	0.00	0.00	0.00	0.00	0.00	0.00	0.00	0.00	0.00	0.00	0.00
**CE11**	0.19	0.21	0.29	0.24	0.16	0.20	0.14	0.29	0.20	0.23	0.34	0.17	0.22	0.37	0.21	0.20
**GT19**	0.08	0.13	0.22	0.10	0.05	0.18	0.06	0.16	0.07	0.08	0.22	0.16	0.10	0.22	0.16	0.16
**GT33**	0.00	0.00	0.00	0.00	0.00	0.00	0.00	0.00	0.00	0.00	0.00	0.00	0.00	0.00	0.00	0.00
**GH21**	0.00	0.00	0.00	0.00	0.00	0.00	0.00	0.00	0.00	0.00	0.00	0.00	0.00	0.00	0.00	0.00
**GT68**	0.00	0.00	0.00	0.00	0.00	0.00	0.00	0.00	0.00	0.00	0.00	0.00	0.00	0.00	0.00	0.00
**GH89**	0.10	0.22	0.31	0.17	0.13	0.27	0.02	0.15	0.08	0.28	0.09	0.16	0.25	0.33	0.26	0.14
**GT82**	0.00	0.00	0.00	0.00	0.01	0.00	0.00	0.00	0.00	0.00	0.00	0.00	0.00	0.02	0.00	0.00
**GH30**	0.22	0.20	0.24	0.12	0.10	0.33	0.16	0.16	0.15	0.18	0.15	0.26	0.28	0.27	0.24	0.29
**GH20**	0.93	1.13	1.79	1.01	0.77	1.49	0.35	0.67	0.54	1.27	0.49	0.78	1.32	2.16	1.19	0.65
**GH52**	0.00	0.00	0.00	0.00	0.00	0.00	0.00	0.00	0.00	0.00	0.00	0.00	0.00	0.00	0.00	0.00
**GT84**	0.03	0.00	0.00	0.02	0.04	0.08	0.06	0.00	0.01	0.08	0.06	0.06	0.03	0.02	0.01	0.04
**GT15**	0.00	0.00	0.00	0.00	0.00	0.00	0.00	0.00	0.00	0.00	0.00	0.00	0.00	0.00	0.00	0.00
**GH93**	0.00	0.00	0.00	0.00	0.00	0.02	0.00	0.00	0.00	0.00	0.00	0.00	0.02	0.00	0.00	0.00
**PL16**	0.00	0.00	0.00	0.00	0.00	0.00	0.00	0.00	0.00	0.00	0.00	0.00	0.00	0.00	0.00	0.00
**GH26**	0.10	0.17	0.04	0.12	0.09	0.15	0.00	0.16	0.11	0.05	0.12	0.16	0.17	0.18	0.16	0.03

CAZY_group	ES-AD-35	ES-AD-36	ES-AD-37	ES-AD-38	ES-AD-39	ES-AD-4	ES-AD-5	ES-AD-6	ES-AD-7	ES-AD-8	ES-AD-9	FR-AD-1	FR-AD-2	FR-AD-3	FR-AD-4	FR-AD-5
GT75	0.00	0.00	0.00	0.00	0.00	0.00	0.00	0.00	0.00	0.00	0.00	0.00	0.00	0.00	0.00	0.00
GH53	0.22	0.09	0.04	0.14	0.04	0.14	0.06	0.04	0.18	0.10	0.22	0.16	0.05	0.17	0.10	0.07
GT88	0.00	0.00	0.00	0.00	0.00	0.00	0.00	0.00	0.00	0.00	0.00	0.00	0.00	0.00	0.00	0.00
GT24	0.00	0.00	0.00	0.00	0.00	0.00	0.00	0.00	0.00	0.00	0.00	0.00	0.00	0.00	0.00	0.00
GT81	0.00	0.00	0.00	0.00	0.00	0.00	0.00	0.00	0.00	0.00	0.00	0.00	0.00	0.00	0.00	0.00
GH4	0.10	0.16	0.24	0.13	0.21	0.13	0.29	0.07	0.09	0.05	0.09	0.24	0.13	0.12	0.26	0.17
GT78	0.00	0.00	0.00	0.00	0.00	0.00	0.00	0.00	0.00	0.00	0.00	0.00	0.00	0.00	0.00	0.00
GH8	0.09	0.07	0.02	0.06	0.02	0.04	0.04	0.08	0.13	0.00	0.06	0.10	0.07	0.13	0.11	0.06
GH70	0.00	0.21	0.00	0.00	0.00	0.00	0.00	0.00	0.00	0.00	0.00	0.00	0.00	0.02	0.00	0.00
GH122	0.00	0.00	0.00	0.00	0.00	0.00	0.00	0.00	0.00	0.00	0.00	0.00	0.00	0.00	0.00	0.00
GH74	0.02	0.02	0.00	0.03	0.02	0.00	0.00	0.04	0.05	0.03	0.00	0.04	0.07	0.07	0.06	0.01
GT41	0.01	0.01	0.00	0.00	0.00	0.01	0.00	0.01	0.01	0.00	0.06	0.00	0.02	0.02	0.06	0.00
PL1	0.10	0.24	0.14	0.13	0.07	0.42	0.20	0.08	0.18	0.31	0.09	0.21	0.20	0.47	0.28	0.22
GH15	0.04	0.07	0.04	0.03	0.03	0.08	0.00	0.01	0.02	0.08	0.00	0.01	0.08	0.20	0.04	0.00
GH32	0.44	0.52	0.54	0.48	0.41	0.47	0.65	0.45	0.29	0.47	0.61	0.53	0.42	0.50	0.77	0.69
GH44	0.00	0.00	0.00	0.02	0.00	0.02	0.00	0.00	0.00	0.00	0.00	0.00	0.00	0.00	0.00	0.00
GT6	0.00	0.00	0.00	0.01	0.00	0.01	0.00	0.00	0.00	0.00	0.00	0.01	0.00	0.02	0.00	0.00
GH13	3.07	3.54	2.57	3.62	3.13	3.63	3.87	2.72	3.30	2.46	3.04	4.69	3.55	4.17	4.47	3.83
GT80	0.01	0.01	0.00	0.00	0.01	0.04	0.00	0.00	0.00	0.00	0.00	0.00	0.03	0.02	0.01	0.01
GH71	0.00	0.00	0.00	0.00	0.00	0.00	0.00	0.00	0.00	0.00	0.00	0.00	0.00	0.00	0.00	0.00
GH63	0.07	0.15	0.11	0.06	0.05	0.17	0.04	0.03	0.08	0.16	0.03	0.10	0.15	0.25	0.11	0.06
PL21	0.01	0.00	0.00	0.03	0.01	0.00	0.00	0.00	0.01	0.03	0.00	0.01	0.02	0.00	0.06	0.00
GH65	0.11	0.18	0.09	0.14	0.18	0.17	0.10	0.05	0.15	0.13	0.22	0.17	0.27	0.28	0.36	0.07
CE4	0.61	0.84	0.63	0.67	0.73	0.63	0.92	0.56	0.72	0.52	0.52	0.57	0.64	0.57	0.67	0.61
CE14	0.00	0.01	0.05	0.03	0.00	0.01	0.00	0.00	0.02	0.00	0.00	0.03	0.07	0.07	0.01	0.04
GH115	0.26	0.29	0.20	0.17	0.08	0.26	0.00	0.08	0.27	0.10	0.06	0.33	0.08	0.33	0.28	0.12
GH104	0.00	0.01	0.00	0.00	0.00	0.00	0.00	0.00	0.00	0.00	0.00	0.00	0.00	0.00	0.00	0.00
GH111	0.00	0.00	0.00	0.00	0.00	0.00	0.00	0.00	0.00	0.00	0.00	0.00	0.00	0.00	0.00	0.00
GH95	0.34	0.64	0.60	0.31	0.30	0.71	0.31	0.49	0.33	0.70	0.34	0.51	0.65	1.11	0.74	0.56
GT70	0.00	0.00	0.00	0.00	0.00	0.00	0.00	0.00	0.00	0.00	0.00	0.00	0.00	0.00	0.00	0.00
CE10	0.09	0.14	0.22	0.13	0.10	0.27	0.14	0.10	0.12	0.10	0.03	0.18	0.12	0.32	0.20	0.06
GH10	0.09	0.10	0.05	0.09	0.05	0.13	0.00	0.08	0.18	0.03	0.15	0.20	0.13	0.17	0.09	0.14
GH130	0.24	0.32	0.25	0.20	0.21	0.50	0.12	0.22	0.27	0.26	0.25	0.43	0.35	0.44	0.41	0.23
PL20	0.00	0.00	0.00	0.00	0.00	0.00	0.00	0.00	0.00	0.00	0.00	0.00	0.00	0.00	0.00	0.00
GH69	0.00	0.00	0.00	0.00	0.00	0.00	0.00	0.00	0.00	0.00	0.00	0.00	0.00	0.00	0.00	0.00
PL2	0.00	0.00	0.00	0.00	0.00	0.00	0.00	0.00	0.00	0.00	0.00	0.00	0.00	0.00	0.00	0.00
GH49	0.00	0.00	0.00	0.00	0.00	0.00	0.00	0.00	0.00	0.00	0.00	0.00	0.00	0.00	0.00	0.00
GH25	0.28	0.47	0.43	0.41	0.25	0.28	0.75	0.49	0.45	0.36	0.92	0.64	0.42	0.33	0.61	0.30
GH59	0.00	0.06	0.00	0.02	0.00	0.06	0.00	0.01	0.07	0.00	0.03	0.09	0.02	0.03	0.01	0.03
GH35	0.29	0.30	0.56	0.30	0.23	0.30	0.12	0.23	0.25	0.28	0.03	0.38	0.38	0.67	0.48	0.25
GT39	0.00	0.00	0.00	0.00	0.01	0.02	0.00	0.00	0.01	0.00	0.00	0.04	0.05	0.02	0.01	0.01
GT57	0.00	0.00	0.00	0.00	0.00	0.00	0.00	0.00	0.00	0.00	0.00	0.00	0.00	0.00	0.00	0.00
GH129	0.01	0.00	0.00	0.03	0.04	0.01	0.00	0.01	0.01	0.00	0.00	0.03	0.02	0.00	0.06	0.01
GT22	0.00	0.00	0.00	0.00	0.00	0.01	0.00	0.00	0.00	0.00	0.00	0.00	0.00	0.00	0.00	0.00
PL12	0.03	0.05	0.00	0.03	0.03	0.11	0.00	0.04	0.03	0.18	0.00	0.01	0.10	0.10	0.14	0.06
PL18	0.00	0.00	0.00	0.00	0.00	0.00	0.00	0.00	0.00	0.00	0.00	0.00	0.00	0.00	0.00	0.00
GT92	0.00	0.00	0.00	0.00	0.00	0.00	0.00	0.00	0.00	0.00	0.00	0.00	0.00	0.00	0.00	0.00
GT44	0.00	0.00	0.00	0.01	0.00	0.00	0.00	0.00	0.00	0.00	0.00	0.00	0.00	0.00	0.00	0.00
GT29	0.00	0.00	0.00	0.00	0.00	0.00	0.00	0.00	0.00	0.00	0.00	0.00	0.00	0.00	0.00	0.00
GT13	0.00	0.00	0.00	0.00	0.00	0.00	0.00	0.00	0.00	0.00	0.00	0.00	0.00	0.00	0.00	0.00

CAZY_group	ES-AD-35	ES-AD-36	ES-AD-37	ES-AD-38	ES-AD-39	ES-AD-4	ES-AD-5	ES-AD-6	ES-AD-7	ES-AD-8	ES-AD-9	FR-AD-1	FR-AD-2	FR-AD-3	FR-AD-4	FR-AD-5
**GT52**	0.00	0.00	0.00	0.00	0.00	0.00	0.00	0.00	0.00	0.00	0.00	0.00	0.00	0.00	0.00	0.00
**GT64**	0.00	0.00	0.00	0.00	0.00	0.00	0.00	0.00	0.00	0.00	0.00	0.00	0.00	0.00	0.00	0.00
**GT85**	0.00	0.00	0.00	0.00	0.00	0.00	0.00	0.00	0.00	0.00	0.00	0.00	0.00	0.00	0.00	0.00
**GT16**	0.00	0.00	0.00	0.00	0.00	0.00	0.00	0.00	0.00	0.00	0.00	0.00	0.00	0.00	0.00	0.00
**GH56**	0.00	0.00	0.00	0.00	0.00	0.00	0.00	0.00	0.00	0.00	0.00	0.00	0.00	0.00	0.00	0.00
**GT60**	0.00	0.00	0.00	0.00	0.00	0.00	0.00	0.00	0.00	0.00	0.00	0.00	0.00	0.00	0.00	0.00
**GH41**	0.00	0.00	0.00	0.00	0.00	0.00	0.00	0.00	0.00	0.00	0.00	0.00	0.00	0.00	0.00	0.00
**CE7**	0.05	0.14	0.09	0.04	0.08	0.11	0.10	0.07	0.08	0.13	0.09	0.13	0.10	0.20	0.06	0.04
**GH39**	0.05	0.06	0.00	0.02	0.02	0.02	0.02	0.01	0.07	0.05	0.09	0.06	0.08	0.07	0.09	0.04
**GT30**	0.25	0.26	0.38	0.19	0.19	0.30	0.12	0.19	0.14	0.26	0.12	0.24	0.35	0.50	0.21	0.33
**PL15**	0.02	0.02	0.00	0.04	0.05	0.03	0.00	0.05	0.02	0.05	0.00	0.04	0.13	0.02	0.16	0.01
**GH102**	0.01	0.08	0.02	0.00	0.01	0.03	0.02	0.01	0.01	0.00	0.00	0.10	0.03	0.12	0.10	0.06

CAZY_group	FR-AD-6	FR-AD-7	FR-AD-8	IN-CH-15	IN-CH-14	IN-CH-13	IT-AD-1	IT-AD-2	IT-AD-3	IT-AD-4	IT-AD-5	IT-AD-6	JP-AD-1	JP-AD-2	JP-AD-3	JP-AD-4
GH29	0.77	0.70	0.75	1.00	1.66	0.62	0.86	0.49	0.93	0.37	0.80	0.63	0.57	0.72	0.37	0.72
PL4	0.00	0.00	0.00	0.00	0.00	0.00	0.00	0.00	0.00	0.00	0.00	0.00	0.00	0.00	0.00	0.00
GH23	1.78	2.00	1.74	1.72	2.09	0.65	2.32	2.14	2.42	1.94	2.00	2.10	1.44	1.73	1.47	1.71
CE6	0.10	0.03	0.02	0.45	0.21	0.12	0.00	0.00	0.00	0.05	0.02	0.08	0.03	0.00	0.02	0.05
GH72	0.16	0.20	0.22	0.34	0.48	0.05	0.14	0.16	0.16	0.16	0.15	0.22	0.03	0.17	0.16	0.24
GH114	0.00	0.00	0.00	0.00	0.00	0.00	0.00	0.00	0.00	0.00	0.00	0.00	0.00	0.00	0.00	0.00
GH78	0.45	0.99	1.20	0.40	2.01	0.11	0.99	0.60	0.92	0.21	0.79	0.76	1.01	0.42	0.80	0.83
PL19	0.00	0.00	0.00	0.00	0.00	0.00	0.00	0.00	0.00	0.00	0.00	0.00	0.00	0.00	0.00	0.00
GT27	0.00	0.00	0.00	0.00	0.02	0.00	0.00	0.00	0.00	0.00	0.00	0.00	0.00	0.00	0.00	0.00
GH5	0.66	0.87	0.92	0.79	0.68	0.56	0.27	0.34	0.56	0.50	0.38	0.43	0.24	0.20	0.33	0.40
GT40	0.00	0.00	0.00	0.00	0.00	0.00	0.00	0.00	0.00	0.00	0.00	0.00	0.00	0.00	0.00	0.00
CE13	0.00	0.00	0.00	0.00	0.00	0.00	0.00	0.00	0.00	0.00	0.00	0.00	0.00	0.00	0.00	0.00
GT37	0.00	0.00	0.00	0.00	0.00	0.00	0.00	0.00	0.00	0.00	0.00	0.00	0.00	0.00	0.00	0.00
GH33	0.06	0.34	0.20	0.19	0.39	0.12	0.39	0.16	0.20	0.24	0.29	0.31	0.19	0.10	0.16	0.13
GT65	0.00	0.00	0.00	0.00	0.00	0.00	0.00	0.00	0.00	0.00	0.00	0.00	0.00	0.00	0.00	0.00
GH86	0.00	0.02	0.00	0.00	0.00	0.00	0.00	0.00	0.00	0.00	0.00	0.00	0.03	0.02	0.00	0.00
GH123	0.05	0.08	0.27	0.19	0.27	0.12	0.21	0.09	0.16	0.03	0.14	0.05	0.08	0.07	0.05	0.13
GH96	0.00	0.00	0.00	0.00	0.00	0.00	0.00	0.00	0.00	0.00	0.00	0.00	0.00	0.00	0.00	0.00
GH14	0.00	0.00	0.00	0.00	0.00	0.00	0.00	0.00	0.00	0.00	0.00	0.00	0.00	0.00	0.00	0.00
CE3	0.00	0.00	0.00	0.00	0.00	0.00	0.00	0.00	0.00	0.00	0.00	0.00	0.00	0.00	0.00	0.00
PL10	0.14	0.02	0.12	0.32	0.29	0.12	0.23	0.00	0.02	0.05	0.11	0.05	0.16	0.05	0.07	0.11
GT48	0.00	0.00	0.00	0.00	0.00	0.00	0.00	0.00	0.00	0.00	0.00	0.00	0.00	0.00	0.00	0.00
PL6	0.00	0.00	0.00	0.00	0.00	0.00	0.00	0.00	0.00	0.00	0.00	0.00	0.00	0.00	0.00	0.00
GT83	0.08	0.00	0.15	0.32	0.29	0.11	0.10	0.09	0.14	0.08	0.17	0.08	0.16	0.15	0.23	0.16
GH126	0.00	0.00	0.00	0.00	0.00	0.00	0.00	0.00	0.00	0.00	0.00	0.00	0.00	0.00	0.00	0.00
GH9	0.42	0.29	0.28	0.05	0.12	0.02	0.12	0.11	0.28	0.11	0.21	0.23	0.27	0.15	0.19	0.05
GH75	0.00	0.00	0.00	0.00	0.00	0.00	0.00	0.00	0.00	0.00	0.00	0.00	0.00	0.00	0.00	0.00
GT71	0.00	0.00	0.00	0.00	0.00	0.00	0.00	0.00	0.00	0.00	0.00	0.00	0.00	0.00	0.00	0.00
GH46	0.00	0.00	0.02	0.00	0.00	0.00	0.00	0.00	0.05	0.00	0.00	0.00	0.00	0.00	0.00	0.00
GT61	0.00	0.00	0.00	0.00	0.00	0.00	0.00	0.00	0.00	0.00	0.00	0.00	0.00	0.00	0.00	0.00
GT3	0.21	0.22	0.10	0.61	0.43	0.30	0.27	0.20	0.28	0.00	0.27	0.22	0.19	0.15	0.16	0.27
PL3	0.00	0.00	0.00	0.00	0.00	0.00	0.00	0.00	0.00	0.00	0.00	0.00	0.00	0.00	0.00	0.00
GH108	0.00	0.02	0.00	0.00	0.04	0.04	0.04	0.02	0.02	0.00	0.02	0.05	0.03	0.07	0.02	0.03
GT56	0.00	0.00	0.00	0.03	0.02	0.00	0.02	0.04	0.03	0.00	0.02	0.00	0.00	0.00	0.02	0.00
PL11	0.05	0.20	0.13	0.11	0.33	0.00	0.12	0.02	0.14	0.11	0.05	0.11	0.24	0.10	0.07	0.24
GH45	0.00	0.00	0.00	0.00	0.00	0.00	0.00	0.00	0.00	0.00	0.00	0.00	0.00	0.00	0.00	0.00
GT34	0.00	0.00	0.00	0.03	0.00	0.00	0.00	0.00	0.00	0.00	0.00	0.00	0.00	0.00	0.00	0.03
PL14	0.00	0.00	0.00	0.00	0.00	0.00	0.00	0.00	0.00	0.00	0.00	0.00	0.00	0.00	0.00	0.00
GH19	0.00	0.02	0.00	0.03	0.02	0.04	0.00	0.02	0.00	0.00	0.00	0.02	0.00	0.00	0.05	0.03
GH118	0.00	0.00	0.00	0.00	0.00	0.00	0.00	0.00	0.00	0.00	0.00	0.00	0.00	0.00	0.00	0.00
GH82	0.00	0.00	0.00	0.00	0.00	0.00	0.00	0.00	0.00	0.00	0.00	0.00	0.00	0.00	0.00	0.00
GH76	0.03	0.22	0.05	0.00	0.39	0.00	0.08	0.05	0.19	0.11	0.08	0.05	0.11	0.20	0.02	0.08
GT4	2.34	2.57	2.94	3.36	3.85	1.42	2.20	1.70	2.65	2.76	1.71	2.37	2.42	1.39	1.94	2.20
GT87	0.02	0.02	0.00	0.00	0.06	0.00	0.00	0.02	0.00	0.00	0.03	0.00	0.03	0.00	0.00	0.03
GH24	0.14	0.12	0.23	0.37	0.23	0.37	0.33	0.13	0.26	0.32	0.14	0.05	0.19	0.07	0.23	0.13
GH90	0.00	0.00	0.00	0.00	0.02	0.00	0.00	0.00	0.00	0.00	0.00	0.00	0.00	0.00	0.00	0.00
GH57	0.11	0.13	0.18	0.58	0.23	0.21	0.06	0.11	0.19	0.03	0.12	0.22	0.11	0.07	0.12	0.21
GH36	0.98	1.34	0.92	1.72	1.30	0.83	0.88	0.62	1.03	0.87	0.85	0.99	0.73	0.72	0.61	0.64
GT46	0.00	0.00	0.00	0.00	0.00	0.00	0.00	0.00	0.00	0.00	0.00	0.00	0.00	0.00	0.00	0.00
GH66	0.05	0.03	0.03	0.21	0.17	0.04	0.04	0.02	0.03	0.21	0.06	0.02	0.05	0.02	0.09	0.00

CAZY_group	FR-AD-6	FR-AD-7	FR-AD-8	IN-CH-15	IN-CH-14	IN-CH-13	IT-AD-1	IT-AD-2	IT-AD-3	IT-AD-4	IT-AD-5	IT-AD-6	JP-AD-1	JP-AD-2	JP-AD-3	JP-AD-4
GH48	0.00	0.00	0.00	0.00	0.00	0.00	0.00	0.00	0.00	0.00	0.00	0.00	0.00	0.00	0.00	0.00
GT53	0.00	0.00	0.00	0.00	0.00	0.00	0.00	0.00	0.00	0.00	0.00	0.00	0.00	0.00	0.00	0.00
GH60	0.00	0.00	0.00	0.00	0.00	0.00	0.00	0.00	0.00	0.00	0.00	0.00	0.00	0.00	0.00	0.00
GT31	0.00	0.00	0.00	0.03	0.02	0.00	0.00	0.00	0.00	0.00	0.00	0.02	0.00	0.00	0.00	0.00
GH105	0.42	0.62	0.65	0.63	1.20	0.12	0.58	0.24	0.79	0.32	0.36	0.42	0.79	0.32	0.49	0.62
GT23	0.00	0.00	0.02	0.00	0.00	0.00	0.00	0.00	0.02	0.03	0.00	0.03	0.08	0.00	0.00	0.00
GH11	0.00	0.02	0.00	0.00	0.00	0.00	0.00	0.00	0.00	0.00	0.00	0.00	0.00	0.00	0.00	0.03
GT38	0.00	0.00	0.00	0.00	0.00	0.00	0.00	0.00	0.00	0.00	0.00	0.00	0.00	0.00	0.00	0.00
GT43	0.00	0.00	0.00	0.00	0.00	0.00	0.00	0.00	0.00	0.00	0.00	0.00	0.00	0.00	0.00	0.00
GH99	0.00	0.00	0.00	0.03	0.00	0.00	0.00	0.00	0.00	0.00	0.00	0.00	0.00	0.00	0.00	0.00
GH38	0.32	0.55	0.37	0.26	0.41	0.00	0.25	0.40	0.45	0.16	0.47	0.35	0.24	0.45	0.26	0.37
GH109	0.16	0.17	0.52	0.34	0.50	0.35	0.49	0.29	0.37	0.16	0.23	0.32	0.52	0.07	0.09	0.48
GH2	4.34	6.05	5.33	9.39	11.10	2.27	4.39	3.33	4.83	4.06	3.67	4.41	4.84	3.12	4.45	4.87
GT28	0.42	0.57	0.57	0.82	0.54	0.39	0.68	0.87	0.62	0.64	0.58	0.68	0.49	0.49	0.49	0.54
GH42	0.32	0.32	0.37	0.03	0.14	0.07	0.33	0.38	0.39	0.27	0.38	0.23	0.33	0.20	0.42	0.46
GT58	0.00	0.00	0.00	0.00	0.00	0.00	0.00	0.00	0.00	0.00	0.00	0.00	0.00	0.00	0.00	0.00
GH61	0.00	0.00	0.00	0.00	0.00	0.00	0.00	0.00	0.00	0.00	0.00	0.00	0.00	0.00	0.00	0.00
GH80	0.00	0.00	0.00	0.00	0.00	0.00	0.00	0.00	0.00	0.00	0.00	0.00	0.00	0.00	0.00	0.00
GH98	0.02	0.00	0.00	0.00	0.00	0.00	0.00	0.00	0.00	0.00	0.00	0.00	0.00	0.00	0.00	0.00
GH6	0.00	0.00	0.00	0.00	0.00	0.00	0.00	0.00	0.00	0.00	0.00	0.00	0.00	0.00	0.00	0.00
GT1	0.13	0.12	0.12	0.00	0.12	0.02	0.06	0.02	0.08	0.27	0.08	0.03	0.00	0.05	0.07	0.08
GH28	1.09	0.87	0.78	1.16	1.74	0.49	0.70	0.43	0.86	0.45	0.58	0.71	0.79	0.54	0.82	0.99
GT42	0.00	0.00	0.00	0.00	0.00	0.00	0.00	0.00	0.00	0.00	0.00	0.00	0.00	0.00	0.00	0.00
GH128	0.00	0.00	0.00	0.03	0.00	0.00	0.00	0.00	0.00	0.00	0.00	0.00	0.00	0.00	0.00	0.00
CE12	0.16	0.08	0.10	0.24	0.23	0.12	0.06	0.02	0.08	0.05	0.06	0.08	0.08	0.10	0.05	0.08
CE16	0.00	0.00	0.00	0.00	0.00	0.00	0.00	0.00	0.00	0.00	0.00	0.00	0.00	0.00	0.00	0.00
GT89	0.00	0.00	0.00	0.00	0.00	0.00	0.00	0.00	0.00	0.00	0.00	0.00	0.00	0.00	0.00	0.00
GT11	0.08	0.03	0.10	0.05	0.14	0.05	0.04	0.04	0.09	0.00	0.02	0.03	0.08	0.05	0.00	0.03
GT77	0.02	0.02	0.00	0.11	0.12	0.07	0.08	0.51	0.00	0.00	0.08	0.00	0.00	0.00	0.02	0.00
GT55	0.00	0.00	0.00	0.00	0.00	0.00	0.00	0.00	0.00	0.00	0.00	0.00	0.00	0.00	0.00	0.00
GT18	0.00	0.00	0.00	0.00	0.00	0.00	0.00	0.00	0.00	0.00	0.00	0.00	0.00	0.00	0.00	0.00
GH87	0.03	0.00	0.02	0.11	0.00	0.00	0.00	0.00	0.03	0.05	0.00	0.00	0.00	0.02	0.00	0.05
PL5	0.00	0.00	0.00	0.00	0.00	0.00	0.00	0.00	0.00	0.00	0.00	0.00	0.00	0.00	0.02	0.00
GH22	0.00	0.00	0.00	0.00	0.00	0.00	0.00	0.00	0.00	0.00	0.00	0.00	0.00	0.00	0.00	0.00
CE2	0.05	0.15	0.05	0.11	0.04	0.05	0.06	0.07	0.08	0.05	0.05	0.06	0.00	0.02	0.09	0.05
GH120	0.06	0.12	0.10	0.00	0.02	0.00	0.12	0.07	0.14	0.13	0.05	0.08	0.00	0.07	0.07	0.00
GH73	0.69	0.70	0.72	0.79	0.60	0.40	0.57	0.29	0.56	0.64	0.47	0.25	0.57	0.37	0.49	0.43
GT17	0.00	0.00	0.00	0.03	0.00	0.00	0.00	0.00	0.00	0.00	0.00	0.00	0.00	0.00	0.00	0.00
GH124	0.00	0.00	0.00	0.00	0.00	0.00	0.00	0.00	0.00	0.00	0.00	0.00	0.00	0.00	0.00	0.00
GH54	0.00	0.00	0.00	0.00	0.00	0.00	0.00	0.00	0.00	0.00	0.00	0.00	0.00	0.00	0.00	0.00
GH100	0.00	0.00	0.00	0.00	0.00	0.00	0.00	0.00	0.00	0.00	0.00	0.00	0.00	0.00	0.00	0.00
GH113	0.00	0.07	0.02	0.00	0.00	0.02	0.00	0.02	0.00	0.03	0.00	0.05	0.00	0.00	0.00	0.05
GT5	0.86	0.69	0.73	0.58	0.43	0.42	0.33	0.54	0.61	0.37	0.68	0.59	0.54	0.57	0.35	0.37
GH94	0.61	0.69	0.78	0.63	0.21	0.25	0.29	0.45	0.76	0.61	0.41	0.59	0.30	0.37	0.40	0.37
GT66	0.00	0.03	0.00	0.00	0.12	0.00	0.04	0.00	0.02	0.00	0.06	0.00	0.00	0.00	0.00	0.05
GT94	0.00	0.00	0.00	0.00	0.00	0.00	0.00	0.00	0.00	0.00	0.00	0.00	0.00	0.00	0.00	0.00
GH58	0.00	0.00	0.00	0.00	0.00	0.00	0.00	0.00	0.00	0.00	0.00	0.00	0.00	0.00	0.00	0.00
GH1	1.22	0.96	1.05	0.77	0.35	0.26	0.47	0.71	0.73	1.72	0.55	0.71	0.68	0.89	0.94	0.56
PL22	0.00	0.00	0.00	0.00	0.00	0.00	0.00	0.02	0.02	0.00	0.00	0.00	0.00	0.00	0.05	0.00
GT9	0.26	0.02	0.15	0.71	0.64	0.40	0.10	0.07	0.22	0.13	0.12	0.11	0.19	0.32	0.26	0.11

CAZY_group	FR-AD-6	FR-AD-7	FR-AD-8	IN-CH-15	IN-CH-14	IN-CH-13	IT-AD-1	IT-AD-2	IT-AD-3	IT-AD-4	IT-AD-5	IT-AD-6	JP-AD-1	JP-AD-2	JP-AD-3	JP-AD-4
GT76	0.00	0.00	0.00	0.00	0.00	0.00	0.00	0.00	0.00	0.00	0.00	0.00	0.00	0.00	0.00	0.00
GT32	0.11	0.17	0.13	0.05	0.12	0.12	0.12	0.04	0.11	0.27	0.06	0.06	0.11	0.12	0.09	0.03
GT47	0.03	0.10	0.07	0.16	0.17	0.04	0.06	0.05	0.05	0.13	0.08	0.03	0.05	0.05	0.09	0.05
GH84	0.13	0.12	0.25	0.50	0.48	0.25	0.41	0.07	0.09	0.24	0.09	0.20	0.16	0.02	0.12	0.05
GH83	0.00	0.00	0.00	0.00	0.00	0.00	0.00	0.00	0.00	0.00	0.00	0.00	0.00	0.00	0.00	0.00
GT59	0.00	0.00	0.00	0.00	0.00	0.00	0.00	0.00	0.00	0.00	0.00	0.00	0.00	0.00	0.00	0.00
GH34	0.00	0.00	0.00	0.00	0.00	0.00	0.00	0.00	0.00	0.00	0.00	0.00	0.00	0.00	0.00	0.00
GT14	0.03	0.03	0.05	0.03	0.06	0.00	0.04	0.00	0.02	0.05	0.03	0.00	0.05	0.02	0.02	0.03
GH47	0.00	0.00	0.00	0.00	0.02	0.00	0.00	0.00	0.00	0.03	0.02	0.02	0.00	0.00	0.00	0.00
GH97	0.77	0.96	1.02	1.85	2.36	0.70	0.82	0.40	0.73	0.27	0.55	0.77	1.06	0.37	0.49	0.59
GH50	0.00	0.00	0.02	0.00	0.10	0.00	0.00	0.04	0.02	0.00	0.02	0.00	0.03	0.02	0.00	0.05
GT26	0.30	0.20	0.23	0.34	0.27	0.21	0.29	0.22	0.30	0.21	0.24	0.32	0.24	0.32	0.26	0.08
GH18	0.53	0.79	0.53	0.24	0.66	0.26	0.74	0.56	0.47	0.24	0.70	0.49	0.52	0.49	0.42	0.35
GH37	0.00	0.00	0.03	0.16	0.04	0.00	0.08	0.14	0.08	0.00	0.03	0.00	0.00	0.00	0.02	0.05
PL13	0.02	0.03	0.02	0.00	0.12	0.00	0.02	0.02	0.03	0.00	0.05	0.00	0.03	0.02	0.00	0.03
GH27	0.11	0.34	0.37	0.48	0.31	0.21	0.33	0.33	0.34	0.21	0.20	0.11	0.16	0.12	0.21	0.27
GT54	0.00	0.00	0.00	0.00	0.00	0.00	0.00	0.00	0.00	0.00	0.00	0.00	0.00	0.00	0.00	0.00
GT91	0.00	0.00	0.00	0.00	0.00	0.00	0.00	0.00	0.00	0.00	0.00	0.00	0.00	0.00	0.00	0.00
GT72	0.00	0.00	0.00	0.00	0.00	0.00	0.00	0.00	0.00	0.00	0.00	0.00	0.00	0.00	0.00	0.00
GH67	0.06	0.08	0.10	0.42	0.19	0.12	0.12	0.04	0.02	0.08	0.02	0.08	0.08	0.00	0.00	0.08
GH12	0.00	0.00	0.00	0.00	0.02	0.00	0.00	0.00	0.00	0.00	0.00	0.00	0.00	0.00	0.00	0.00
GH91	0.02	0.07	0.05	0.00	0.12	0.00	0.00	0.02	0.05	0.00	0.05	0.00	0.00	0.00	0.00	0.00
GT69	0.00	0.00	0.00	0.00	0.00	0.00	0.00	0.00	0.00	0.00	0.00	0.00	0.00	0.00	0.00	0.00
GH106	0.16	0.07	0.38	0.34	0.68	0.11	0.31	0.07	0.23	0.08	0.36	0.22	0.27	0.15	0.16	0.32
GT62	0.00	0.00	0.00	0.00	0.00	0.00	0.00	0.00	0.00	0.00	0.00	0.00	0.00	0.00	0.00	0.00
GH17	0.00	0.00	0.00	0.00	0.00	0.00	0.00	0.00	0.00	0.00	0.00	0.00	0.00	0.00	0.00	0.00
GH88	0.24	0.35	0.27	0.16	0.72	0.11	0.33	0.16	0.28	0.16	0.36	0.14	0.54	0.07	0.23	0.29
CE5	0.00	0.00	0.00	0.00	0.00	0.00	0.00	0.00	0.00	0.00	0.00	0.00	0.00	0.00	0.00	0.00
GT8	0.18	0.07	0.18	0.19	0.27	0.09	0.18	0.27	0.08	0.42	0.12	0.08	0.14	0.22	0.00	0.24
GT50	0.00	0.00	0.00	0.00	0.00	0.00	0.00	0.00	0.00	0.00	0.00	0.00	0.00	0.00	0.00	0.00
GT20	0.06	0.03	0.13	0.05	0.14	0.00	0.18	0.05	0.16	0.03	0.06	0.00	0.08	0.02	0.00	0.08
PL9	0.08	0.05	0.07	0.05	0.12	0.00	0.02	0.00	0.08	0.00	0.06	0.05	0.22	0.10	0.12	0.13
GH55	0.06	0.10	0.05	0.00	0.02	0.00	0.00	0.02	0.05	0.00	0.02	0.00	0.05	0.05	0.07	0.03
GH64	0.03	0.00	0.00	0.00	0.00	0.00	0.00	0.00	0.00	0.00	0.00	0.00	0.00	0.00	0.02	0.00
GH127	0.32	0.42	0.47	0.69	0.87	0.26	0.35	0.27	0.42	0.32	0.21	0.34	0.41	0.22	0.26	0.56
GH103	0.00	0.00	0.00	0.05	0.08	0.00	0.06	0.05	0.00	0.00	0.02	0.03	0.03	0.02	0.02	0.00
GH3	2.90	3.57	4.31	3.91	4.27	2.00	3.51	2.57	3.33	2.73	2.61	3.13	2.94	2.20	3.00	3.24
GT45	0.00	0.00	0.00	0.00	0.00	0.00	0.00	0.00	0.00	0.00	0.00	0.00	0.00	0.00	0.00	0.00
GT74	0.00	0.00	0.00	0.00	0.00	0.00	0.00	0.00	0.00	0.00	0.00	0.00	0.00	0.00	0.00	0.00
GT90	0.00	0.00	0.00	0.00	0.00	0.02	0.00	0.00	0.00	0.00	0.00	0.00	0.00	0.00	0.00	0.00
GH116	0.08	0.13	0.15	0.00	0.08	0.02	0.08	0.02	0.06	0.00	0.11	0.00	0.08	0.02	0.05	0.08
GH31	1.06	1.56	1.34	2.12	1.91	0.79	1.07	0.83	1.01	0.82	1.12	1.29	1.20	0.52	1.08	1.31
GT12	0.00	0.00	0.00	0.00	0.00	0.00	0.00	0.00	0.00	0.00	0.00	0.00	0.00	0.00	0.00	0.00
CE1	0.35	0.70	0.62	0.58	0.52	0.12	0.41	0.24	0.30	0.37	0.33	0.59	0.41	0.27	0.35	0.27
GH121	0.02	0.00	0.00	0.00	0.00	0.00	0.04	0.05	0.06	0.11	0.00	0.09	0.00	0.15	0.05	0.08
GH79	0.00	0.00	0.00	0.00	0.00	0.00	0.00	0.02	0.00	0.00	0.00	0.00	0.00	0.00	0.00	0.00
GT73	0.00	0.00	0.00	0.00	0.00	0.00	0.00	0.00	0.02	0.00	0.00	0.00	0.00	0.00	0.00	0.00
GH77	0.72	0.99	0.92	1.24	0.83	0.65	0.97	1.18	0.97	0.87	0.88	1.06	0.76	0.69	1.08	0.72
PL17	0.00	0.00	0.02	0.00	0.02	0.00	0.00	0.00	0.03	0.00	0.00	0.02	0.11	0.00	0.07	0.05
PL8	0.06	0.23	0.13	0.32	0.64	0.18	0.41	0.11	0.22	0.00	0.12	0.12	0.14	0.02	0.21	0.16

CAZY_group	FR-AD-6	FR-AD-7	FR-AD-8	IN-CH-15	IN-CH-14	IN-CH-13	IT-AD-1	IT-AD-2	IT-AD-3	IT-AD-4	IT-AD-5	IT-AD-6	JP-AD-1	JP-AD-2	JP-AD-3	JP-AD-4
**CE9**	0.30	0.34	0.52	0.66	0.85	0.18	0.64	0.33	0.44	0.50	0.42	0.39	0.44	0.32	0.42	0.32
**CE15**	0.02	0.00	0.02	0.00	0.04	0.00	0.06	0.00	0.00	0.00	0.02	0.08	0.00	0.00	0.00	0.00
**GT79**	0.00	0.00	0.00	0.00	0.00	0.00	0.00	0.00	0.00	0.00	0.00	0.00	0.00	0.00	0.00	0.00
**GT10**	0.00	0.03	0.02	0.00	0.02	0.00	0.02	0.02	0.00	0.00	0.00	0.00	0.00	0.00	0.00	0.00
**GH119**	0.00	0.00	0.00	0.00	0.00	0.00	0.00	0.00	0.00	0.00	0.00	0.00	0.00	0.00	0.00	0.00
**GH16**	0.22	0.17	0.28	0.21	0.54	0.21	0.18	0.11	0.28	0.21	0.17	0.15	0.30	0.10	0.12	0.11
**GH43**	2.37	2.73	2.27	4.39	4.06	1.53	2.38	2.14	2.70	1.70	1.67	1.80	2.83	1.31	1.54	2.86
**GH51**	0.75	0.67	0.58	1.61	1.22	0.58	0.72	0.45	0.70	0.85	0.46	0.55	0.41	0.30	0.42	0.70
**GT35**	1.73	1.63	1.34	1.24	1.08	0.79	1.17	1.03	1.54	1.91	1.03	1.37	1.03	1.11	1.12	1.15
**GT25**	0.00	0.02	0.00	0.00	0.06	0.00	0.00	0.00	0.00	0.00	0.00	0.00	0.03	0.00	0.00	0.00
**GT2**	6.54	7.06	7.85	8.17	9.59	4.98	5.79	4.91	6.62	7.24	5.93	5.83	6.61	4.58	5.85	5.27
**GT51**	1.41	1.04	1.25	4.28	4.00	1.09	1.27	1.21	1.03	1.22	1.11	1.37	0.95	0.82	1.40	0.83
**GH107**	0.00	0.00	0.00	0.00	0.00	0.00	0.00	0.00	0.00	0.00	0.00	0.00	0.00	0.00	0.00	0.00
**GH7**	0.00	0.00	0.00	0.00	0.00	0.00	0.00	0.00	0.00	0.00	0.00	0.00	0.00	0.00	0.00	0.00
**GT7**	0.00	0.00	0.00	0.00	0.00	0.00	0.00	0.00	0.00	0.00	0.00	0.00	0.00	0.00	0.00	0.00
**GT36**	0.00	0.00	0.00	0.00	0.00	0.00	0.00	0.00	0.00	0.00	0.00	0.00	0.00	0.00	0.00	0.00
**PL7**	0.00	0.00	0.00	0.00	0.00	0.00	0.00	0.00	0.00	0.03	0.00	0.00	0.00	0.02	0.00	0.00
**GH110**	0.02	0.12	0.17	0.00	0.41	0.04	0.12	0.05	0.14	0.11	0.17	0.06	0.11	0.02	0.07	0.16
**GT21**	0.00	0.00	0.00	0.00	0.00	0.00	0.00	0.00	0.00	0.00	0.00	0.00	0.00	0.00	0.00	0.00
**GH62**	0.00	0.00	0.00	0.00	0.00	0.00	0.00	0.00	0.00	0.00	0.00	0.00	0.00	0.00	0.00	0.00
**GH125**	0.18	0.29	0.18	0.42	0.37	0.09	0.29	0.07	0.19	0.13	0.15	0.28	0.30	0.15	0.26	0.21
**GH117**	0.03	0.05	0.13	0.11	0.12	0.05	0.12	0.05	0.12	0.00	0.08	0.08	0.19	0.07	0.12	0.05
**GH68**	0.00	0.00	0.00	0.03	0.00	0.00	0.00	0.00	0.00	0.11	0.00	0.00	0.00	0.00	0.00	0.00
**GH85**	0.18	0.08	0.00	0.48	0.00	0.11	0.08	0.00	0.08	0.08	0.00	0.03	0.00	0.00	0.14	0.03
**GH92**	1.06	1.56	1.60	2.75	3.91	1.14	1.74	0.63	1.71	0.58	1.33	1.37	1.58	0.57	1.10	1.15
**GH40**	0.00	0.00	0.00	0.00	0.00	0.00	0.00	0.00	0.00	0.00	0.00	0.00	0.00	0.00	0.00	0.00
**GT49**	0.00	0.00	0.00	0.00	0.00	0.00	0.00	0.00	0.00	0.00	0.00	0.00	0.00	0.00	0.00	0.00
**GH112**	0.43	0.54	0.32	0.11	0.06	0.14	0.39	0.29	0.37	0.48	0.18	0.31	0.41	0.27	0.26	0.29
**GT67**	0.00	0.00	0.00	0.00	0.00	0.00	0.00	0.00	0.00	0.00	0.00	0.00	0.00	0.00	0.00	0.00
**CE8**	0.19	0.05	0.20	0.29	0.39	0.19	0.23	0.09	0.23	0.27	0.08	0.09	0.41	0.17	0.28	0.27
**GT86**	0.00	0.00	0.00	0.00	0.00	0.00	0.00	0.00	0.00	0.00	0.00	0.00	0.00	0.00	0.00	0.00
**GH101**	0.02	0.03	0.05	0.00	0.02	0.00	0.23	0.11	0.09	0.16	0.15	0.00	0.08	0.00	0.12	0.08
**GH81**	0.02	0.02	0.03	0.00	0.00	0.00	0.00	0.00	0.00	0.00	0.00	0.00	0.00	0.02	0.00	0.00
**GT93**	0.00	0.00	0.00	0.00	0.00	0.00	0.00	0.00	0.00	0.00	0.00	0.00	0.00	0.00	0.00	0.00
**GT63**	0.00	0.00	0.00	0.00	0.00	0.00	0.00	0.00	0.00	0.00	0.00	0.00	0.00	0.00	0.00	0.00
**CE11**	0.24	0.20	0.28	0.56	0.77	0.33	0.23	0.25	0.22	0.21	0.15	0.25	0.27	0.17	0.21	0.21
**GT19**	0.10	0.15	0.15	0.42	0.39	0.30	0.16	0.13	0.20	0.11	0.15	0.31	0.11	0.15	0.26	0.08
**GT33**	0.00	0.00	0.00	0.00	0.00	0.00	0.00	0.00	0.00	0.00	0.00	0.00	0.00	0.00	0.00	0.00
**GH21**	0.00	0.00	0.00	0.00	0.00	0.00	0.00	0.00	0.00	0.00	0.00	0.00	0.00	0.00	0.00	0.00
**GT68**	0.00	0.00	0.00	0.00	0.00	0.00	0.00	0.00	0.00	0.00	0.00	0.00	0.00	0.00	0.00	0.00
**GH89**	0.24	0.13	0.23	0.56	0.50	0.25	0.12	0.16	0.23	0.21	0.18	0.18	0.19	0.10	0.12	0.27
**GT82**	0.00	0.00	0.00	0.00	0.02	0.00	0.00	0.00	0.00	0.00	0.00	0.00	0.00	0.00	0.00	0.00
**GH30**	0.27	0.25	0.22	0.24	0.31	0.09	0.31	0.14	0.20	0.16	0.23	0.31	0.33	0.05	0.12	0.21
**GH20**	0.85	0.80	1.34	2.06	2.90	0.93	1.87	0.72	1.54	0.48	1.00	1.20	0.73	0.54	0.54	0.94
**GH52**	0.00	0.00	0.00	0.00	0.00	0.00	0.00	0.00	0.00	0.00	0.00	0.00	0.00	0.00	0.00	0.00
**GT84**	0.02	0.02	0.00	0.00	0.00	0.00	0.02	0.07	0.12	0.00	0.08	0.03	0.03	0.00	0.12	0.03
**GT15**	0.00	0.00	0.00	0.00	0.00	0.00	0.00	0.00	0.00	0.00	0.00	0.00	0.00	0.00	0.00	0.00
**GH93**	0.00	0.05	0.02	0.00	0.00	0.00	0.00	0.00	0.02	0.00	0.02	0.00	0.00	0.00	0.00	0.00
**PL16**	0.00	0.00	0.00	0.00	0.00	0.00	0.00	0.00	0.00	0.00	0.00	0.00	0.00	0.00	0.00	0.00
**GH26**	0.11	0.23	0.22	0.29	0.17	0.11	0.16	0.20	0.08	0.00	0.06	0.17	0.03	0.10	0.09	0.29

CAZY_group	FR-AD-6	FR-AD-7	FR-AD-8	IN-CH-15	IN-CH-14	IN-CH-13	IT-AD-1	IT-AD-2	IT-AD-3	IT-AD-4	IT-AD-5	IT-AD-6	JP-AD-1	JP-AD-2	JP-AD-3	JP-AD-4
GT75	0.00	0.00	0.00	0.00	0.00	0.00	0.00	0.00	0.00	0.00	0.00	0.00	0.00	0.00	0.00	0.00
GH53	0.16	0.32	0.17	0.29	0.14	0.25	0.04	0.07	0.23	0.05	0.06	0.08	0.03	0.32	0.12	0.16
GT88	0.00	0.00	0.00	0.00	0.00	0.00	0.00	0.00	0.00	0.00	0.00	0.00	0.00	0.00	0.00	0.00
GT24	0.00	0.00	0.00	0.00	0.00	0.00	0.00	0.00	0.00	0.00	0.00	0.00	0.00	0.00	0.00	0.00
GT81	0.00	0.00	0.00	0.00	0.00	0.00	0.00	0.00	0.00	0.00	0.00	0.00	0.00	0.00	0.00	0.00
GH4	0.18	0.27	0.35	0.32	0.17	0.12	0.21	0.60	0.19	0.16	0.30	0.29	0.11	0.35	0.26	0.05
GT78	0.00	0.00	0.00	0.00	0.00	0.00	0.00	0.00	0.00	0.00	0.00	0.00	0.00	0.00	0.00	0.00
GH8	0.11	0.13	0.03	0.05	0.08	0.12	0.08	0.05	0.12	0.13	0.11	0.03	0.08	0.05	0.05	0.13
GH70	0.08	0.00	0.00	0.11	0.00	0.00	0.00	0.00	0.00	0.87	0.00	0.03	0.00	0.00	0.09	0.00
GH122	0.00	0.00	0.00	0.00	0.00	0.00	0.00	0.00	0.00	0.00	0.00	0.00	0.00	0.00	0.00	0.00
GH74	0.02	0.02	0.03	0.00	0.00	0.00	0.04	0.09	0.02	0.00	0.00	0.00	0.00	0.02	0.02	0.03
GT41	0.06	0.00	0.02	0.00	0.02	0.00	0.00	0.00	0.02	0.03	0.02	0.02	0.03	0.02	0.00	0.03
PL1	0.21	0.37	0.22	0.16	0.64	0.12	0.29	0.07	0.22	0.03	0.12	0.09	0.27	0.12	0.07	0.24
GH15	0.02	0.02	0.03	0.00	0.08	0.00	0.10	0.05	0.12	0.05	0.08	0.00	0.05	0.02	0.02	0.11
GH32	0.88	0.87	0.87	1.08	0.93	0.69	0.51	0.40	0.65	0.98	0.47	0.62	0.60	0.30	0.61	0.46
GH44	0.00	0.02	0.00	0.00	0.00	0.00	0.00	0.00	0.02	0.00	0.00	0.02	0.00	0.00	0.00	0.00
GT6	0.00	0.02	0.00	0.00	0.00	0.00	0.00	0.02	0.00	0.03	0.00	0.00	0.03	0.00	0.00	0.00
GH13	4.42	5.00	5.08	5.10	3.29	3.08	4.31	4.62	4.82	5.51	4.20	4.55	3.13	3.22	4.19	4.39
GT80	0.00	0.00	0.02	0.00	0.04	0.00	0.04	0.09	0.02	0.00	0.02	0.06	0.00	0.00	0.02	0.00
GH71	0.00	0.00	0.00	0.00	0.00	0.00	0.00	0.00	0.00	0.00	0.00	0.00	0.00	0.00	0.00	0.00
GH63	0.16	0.15	0.12	0.26	0.31	0.05	0.31	0.20	0.19	0.03	0.15	0.06	0.16	0.15	0.19	0.13
PL21	0.02	0.00	0.03	0.00	0.19	0.00	0.06	0.00	0.00	0.00	0.00	0.02	0.19	0.00	0.00	0.11
GH65	0.19	0.34	0.45	0.13	0.23	0.05	0.18	0.07	0.20	0.13	0.15	0.14	0.11	0.25	0.16	0.08
CE4	0.61	0.55	0.68	0.82	0.60	0.33	0.62	0.60	0.73	0.42	0.55	0.45	0.65	0.79	0.59	0.54
CE14	0.00	0.02	0.02	0.05	0.06	0.02	0.08	0.04	0.02	0.00	0.05	0.00	0.00	0.05	0.00	0.05
GH115	0.16	0.27	0.20	0.42	0.23	0.14	0.23	0.11	0.36	0.11	0.11	0.18	0.08	0.02	0.02	0.35
GH104	0.00	0.00	0.00	0.00	0.04	0.00	0.00	0.04	0.00	0.00	0.02	0.00	0.00	0.00	0.00	0.00
GH111	0.00	0.00	0.00	0.00	0.00	0.00	0.00	0.00	0.00	0.03	0.00	0.00	0.00	0.00	0.00	0.00
GH95	0.69	0.52	0.70	1.59	1.70	0.53	0.64	0.25	0.69	0.27	0.73	0.46	0.57	0.27	0.44	0.59
GT70	0.00	0.00	0.00	0.00	0.00	0.00	0.00	0.00	0.00	0.00	0.00	0.00	0.00	0.00	0.00	0.00
CE10	0.11	0.10	0.27	0.32	0.35	0.09	0.27	0.13	0.22	0.13	0.14	0.18	0.19	0.22	0.19	0.21
GH10	0.22	0.15	0.15	0.40	0.25	0.18	0.06	0.09	0.11	0.24	0.05	0.09	0.14	0.07	0.05	0.21
GH130	0.26	0.54	0.28	0.26	0.60	0.26	0.47	0.20	0.44	0.21	0.14	0.35	0.27	0.22	0.28	0.37
PL20	0.00	0.00	0.00	0.00	0.00	0.00	0.00	0.00	0.00	0.00	0.00	0.00	0.00	0.00	0.00	0.00
GH69	0.00	0.00	0.00	0.00	0.00	0.00	0.00	0.00	0.00	0.00	0.00	0.00	0.00	0.00	0.00	0.00
PL2	0.00	0.00	0.00	0.00	0.00	0.00	0.00	0.00	0.00	0.00	0.00	0.00	0.00	0.00	0.00	0.00
GH49	0.00	0.00	0.00	0.00	0.00	0.00	0.00	0.00	0.00	0.00	0.00	0.00	0.00	0.00	0.00	0.00
GH25	0.70	0.44	0.50	1.16	0.50	0.76	0.37	0.45	0.37	0.82	0.23	0.39	0.63	0.35	0.51	0.94
GH59	0.00	0.05	0.03	0.03	0.00	0.00	0.00	0.00	0.05	0.00	0.02	0.00	0.00	0.00	0.00	0.00
GH35	0.32	0.29	0.23	0.71	0.95	0.21	0.43	0.43	0.26	0.24	0.36	0.45	0.30	0.07	0.14	0.32
GT39	0.03	0.02	0.00	0.00	0.00	0.00	0.02	0.04	0.02	0.00	0.00	0.00	0.00	0.00	0.00	0.00
GT57	0.00	0.00	0.00	0.00	0.00	0.00	0.00	0.00	0.00	0.00	0.00	0.00	0.00	0.00	0.00	0.00
GH129	0.02	0.03	0.05	0.03	0.02	0.00	0.06	0.04	0.06	0.08	0.02	0.00	0.00	0.02	0.02	0.00
GT22	0.00	0.00	0.00	0.00	0.00	0.00	0.00	0.00	0.00	0.00	0.00	0.00	0.00	0.00	0.00	0.00
PL12	0.05	0.10	0.00	0.05	0.33	0.00	0.08	0.04	0.02	0.00	0.06	0.08	0.16	0.02	0.00	0.08
PL18	0.00	0.00	0.00	0.00	0.00	0.00	0.00	0.00	0.00	0.00	0.00	0.00	0.00	0.00	0.00	0.00
GT92	0.00	0.00	0.00	0.00	0.00	0.00	0.00	0.00	0.00	0.00	0.00	0.00	0.00	0.00	0.00	0.00
GT44	0.00	0.00	0.03	0.03	0.00	0.00	0.00	0.00	0.00	0.00	0.00	0.00	0.00	0.00	0.00	0.00
GT29	0.00	0.00	0.00	0.00	0.00	0.00	0.00	0.00	0.00	0.00	0.00	0.00	0.00	0.00	0.00	0.00
GT13	0.00	0.00	0.00	0.00	0.00	0.00	0.00	0.00	0.00	0.00	0.00	0.00	0.00	0.00	0.00	0.00

CAZY_group	FR-AD-6	FR-AD-7	FR-AD-8	IN-CH-15	IN-CH-14	IN-CH-13	IT-AD-1	IT-AD-2	IT-AD-3	IT-AD-4	IT-AD-5	IT-AD-6	JP-AD-1	JP-AD-2	JP-AD-3	JP-AD-4
**GT52**	0.00	0.00	0.00	0.00	0.00	0.00	0.00	0.00	0.00	0.00	0.00	0.00	0.00	0.00	0.00	0.00
**GT64**	0.00	0.00	0.00	0.00	0.00	0.00	0.00	0.00	0.00	0.00	0.00	0.00	0.00	0.00	0.00	0.00
**GT85**	0.00	0.00	0.00	0.00	0.00	0.00	0.00	0.00	0.00	0.00	0.00	0.00	0.00	0.00	0.00	0.00
**GT16**	0.00	0.00	0.00	0.00	0.00	0.00	0.00	0.00	0.00	0.00	0.00	0.00	0.00	0.00	0.00	0.00
**GH56**	0.00	0.00	0.00	0.00	0.00	0.00	0.00	0.00	0.00	0.00	0.00	0.00	0.00	0.00	0.00	0.00
**GT60**	0.00	0.00	0.00	0.00	0.00	0.00	0.00	0.00	0.00	0.00	0.00	0.00	0.00	0.00	0.00	0.00
**GH41**	0.00	0.00	0.00	0.00	0.00	0.00	0.00	0.00	0.00	0.00	0.00	0.00	0.00	0.00	0.00	0.00
**CE7**	0.10	0.07	0.08	0.34	0.19	0.18	0.06	0.07	0.16	0.11	0.11	0.06	0.14	0.07	0.12	0.08
**GH39**	0.08	0.15	0.05	0.03	0.02	0.00	0.04	0.05	0.14	0.11	0.02	0.05	0.00	0.05	0.07	0.03
**GT30**	0.22	0.25	0.30	0.69	0.66	0.28	0.33	0.36	0.25	0.16	0.27	0.34	0.30	0.20	0.37	0.21
**PL15**	0.05	0.07	0.00	0.11	0.23	0.00	0.02	0.00	0.06	0.00	0.05	0.05	0.03	0.00	0.00	0.24
**GH102**	0.02	0.07	0.05	0.05	0.10	0.00	0.06	0.09	0.11	0.00	0.05	0.02	0.03	0.05	0.16	0.11

CAZY_group	JP-AD-7	JP-AD-8	JP-AD-9	JP-CH-1	JP-CH-2	JP-IN-1	JP-IN-2	JP-IN-3	JP-IN-4	IN-CH-16	MA-AD-1	MA-AD-2	MA-AD-3	MA-AD-4	MA-IN-1	MA-IN-10
GH29	1.30	0.92	0.66	0.50	0.95	0.13	0.18	0.20	0.45	0.74	0.80	0.97	1.27	0.94	1.61	3.38
PL4	0.00	0.00	0.00	0.00	0.02	0.00	0.00	0.00	0.00	0.00	0.00	0.00	0.00	0.00	0.00	0.00
GH23	1.42	1.60	1.47	1.96	1.39	1.78	0.82	1.69	1.67	0.61	2.00	2.85	2.20	1.90	1.78	2.49
CE6	0.04	0.00	0.08	0.03	0.07	0.00	0.00	0.15	0.08	0.29	0.46	0.15	0.75	0.50	0.29	0.00
GH72	0.20	0.24	0.08	0.06	0.14	0.04	0.18	0.10	0.04	0.10	0.55	0.53	0.53	0.52	0.41	0.30
GH114	0.00	0.00	0.00	0.00	0.00	0.00	0.00	0.00	0.00	0.00	0.00	0.00	0.00	0.00	0.00	0.00
GH78	1.03	0.65	0.76	0.98	1.37	0.30	0.18	0.20	0.32	0.35	0.80	0.86	1.04	0.86	0.53	0.00
PL19	0.00	0.00	0.00	0.00	0.00	0.00	0.00	0.00	0.00	0.00	0.00	0.00	0.00	0.00	0.00	0.00
GT27	0.00	0.00	0.00	0.00	0.00	0.00	0.00	0.00	0.00	0.00	0.00	0.00	0.00	0.00	0.00	0.00
GH5	0.24	0.60	0.43	0.65	0.60	0.21	0.18	0.20	0.12	0.84	2.07	1.30	2.14	1.16	1.60	3.10
GT40	0.00	0.00	0.00	0.00	0.00	0.00	0.00	0.00	0.00	0.00	0.00	0.00	0.00	0.00	0.00	0.00
CE13	0.00	0.00	0.00	0.00	0.00	0.00	0.00	0.00	0.00	0.00	0.02	0.00	0.00	0.01	0.00	0.00
GT37	0.00	0.00	0.00	0.00	0.00	0.00	0.00	0.00	0.00	0.00	0.00	0.00	0.00	0.00	0.00	0.00
GH33	0.20	0.22	0.33	0.14	0.35	0.25	0.18	0.10	0.24	0.02	0.19	0.22	0.31	0.24	0.46	2.59
GT65	0.00	0.00	0.00	0.00	0.00	0.00	0.00	0.00	0.00	0.00	0.00	0.00	0.00	0.00	0.00	0.00
GH86	0.00	0.02	0.00	0.00	0.02	0.04	0.00	0.00	0.00	0.00	0.04	0.02	0.04	0.00	0.00	0.00
GH123	0.08	0.10	0.13	0.00	0.14	0.00	0.00	0.00	0.00	0.08	0.05	0.09	0.06	0.11	0.05	0.00
GH96	0.00	0.00	0.00	0.00	0.00	0.00	0.00	0.00	0.00	0.00	0.00	0.00	0.00	0.00	0.00	0.00
GH14	0.00	0.00	0.00	0.00	0.00	0.00	0.00	0.00	0.00	0.00	0.00	0.00	0.00	0.01	0.00	0.00
CE3	0.00	0.00	0.00	0.00	0.00	0.00	0.00	0.00	0.00	0.00	0.00	0.00	0.00	0.01	0.00	0.00
PL10	0.24	0.17	0.18	0.08	0.19	0.00	0.00	0.00	0.00	0.31	0.16	0.07	0.37	0.28	0.28	0.00
GT48	0.00	0.00	0.00	0.00	0.00	0.00	0.00	0.00	0.00	0.00	0.00	0.00	0.00	0.00	0.00	0.00
PL6	0.00	0.02	0.00	0.00	0.00	0.00	0.00	0.00	0.00	0.00	0.00	0.04	0.00	0.00	0.01	0.00
GT83	0.16	0.05	0.15	0.11	0.05	0.13	0.00	0.00	0.32	0.14	0.30	0.09	0.41	0.28	0.67	0.18
GH126	0.00	0.00	0.00	0.00	0.00	0.00	0.00	0.00	0.00	0.00	0.00	0.00	0.00	0.00	0.00	0.00
GH9	0.16	0.46	0.23	0.56	0.77	0.04	0.09	0.00	0.00	0.02	0.67	0.77	0.73	0.45	0.00	0.00
GH75	0.00	0.00	0.00	0.00	0.00	0.00	0.00	0.00	0.00	0.00	0.00	0.00	0.00	0.00	0.00	0.00
GT71	0.00	0.00	0.00	0.00	0.00	0.00	0.00	0.00	0.00	0.00	0.00	0.00	0.00	0.00	0.00	0.00
GH46	0.00	0.00	0.00	0.00	0.00	0.00	0.00	0.00	0.00	0.00	0.00	0.00	0.00	0.00	0.00	0.00
GT61	0.00	0.00	0.00	0.00	0.00	0.00	0.00	0.00	0.00	0.00	0.00	0.00	0.00	0.00	0.00	0.00
GT3	0.20	0.22	0.10	0.11	0.23	0.00	0.00	0.00	0.08	0.37	0.21	0.29	0.69	0.55	0.30	0.00
PL3	0.00	0.00	0.00	0.00	0.00	0.00	0.00	0.00	0.00	0.00	0.00	0.00	0.00	0.00	0.00	0.00
GH108	0.08	0.05	0.00	0.03	0.05	0.08	0.00	0.00	0.04	0.02	0.00	0.04	0.02	0.01	0.01	0.00
GT56	0.00	0.00	0.03	0.00	0.00	0.34	0.00	0.00	0.08	0.00	0.00	0.00	0.00	0.00	0.01	0.06
PL11	0.20	0.12	0.15	0.08	0.19	0.00	0.00	0.00	0.08	0.06	0.23	0.29	0.22	0.19	0.28	0.00
GH45	0.00	0.00	0.00	0.00	0.02	0.00	0.00	0.00	0.00	0.00	0.00	0.00	0.00	0.00	0.00	0.00
GT34	0.04	0.00	0.00	0.00	0.00	0.00	0.18	0.00	0.00	0.00	0.00	0.00	0.00	0.00	0.08	0.03
PL14	0.00	0.00	0.00	0.00	0.00	0.00	0.00	0.00	0.00	0.00	0.00	0.00	0.00	0.00	0.00	0.00
GH19	0.04	0.00	0.00	0.00	0.00	0.21	0.00	0.00	0.04	0.02	0.02	0.04	0.06	0.08	0.08	0.15
GH118	0.00	0.00	0.00	0.00	0.00	0.00	0.00	0.00	0.00	0.00	0.00	0.00	0.00	0.00	0.00	0.00
GH82	0.00	0.00	0.00	0.00	0.00	0.00	0.00	0.00	0.00	0.00	0.00	0.00	0.00	0.00	0.00	0.00
GH76	0.20	0.07	0.23	0.14	0.21	0.00	0.00	0.00	0.00	0.00	0.02	0.05	0.08	0.01	0.00	0.00
GT4	2.77	2.27	2.55	2.33	2.67	1.40	1.64	1.64	1.50	1.51	4.45	6.20	5.55	4.09	5.21	5.57
GT87	0.00	0.00	0.03	0.00	0.00	0.04	0.00	0.15	0.04	0.00	0.00	0.02	0.00	0.01	0.01	0.00
GH24	0.24	0.19	0.15	0.11	0.19	0.38	0.00	0.00	0.41	0.49	0.43	0.27	0.49	0.48	0.22	0.30
GH90	0.00	0.00	0.00	0.00	0.00	0.00	0.00	0.00	0.04	0.00	0.00	0.00	0.00	0.00	0.00	0.00
GH57	0.16	0.24	0.05	0.11	0.12	0.00	0.00	0.00	0.04	0.25	0.34	0.57	0.67	0.59	0.30	0.00
GH36	0.67	0.80	0.86	0.95	0.88	0.59	1.91	1.34	0.61	1.02	1.70	2.63	2.77	1.78	3.51	1.98
GT46	0.00	0.00	0.00	0.00	0.00	0.00	0.00	0.00	0.00	0.00	0.00	0.00	0.00	0.00	0.00	0.00
GH66	0.00	0.02	0.03	0.06	0.07	0.13	0.00	0.00	0.00	0.12	0.19	0.09	0.20	0.29	0.20	0.12

Normalized_abundance_2

CAZY_group	JP-AD-7	JP-AD-8	JP-AD-9	JP-CH-1	JP-CH-2	JP-IN-1	JP-IN-2	JP-IN-3	JP-IN-4	IN-CH-16	MA-AD-1	MA-AD-2	MA-AD-3	MA-AD-4	MA-IN-1	MA-IN-10
**GH48**	0.00	0.00	0.00	0.00	0.02	0.00	0.00	0.00	0.00	0.00	0.02	0.02	0.06	0.00	0.00	0.00
**GT53**	0.00	0.00	0.00	0.00	0.00	0.00	0.00	0.00	0.00	0.00	0.00	0.00	0.00	0.00	0.00	0.00
**GH60**	0.00	0.00	0.00	0.00	0.00	0.00	0.00	0.00	0.00	0.00	0.00	0.00	0.00	0.00	0.00	0.00
**GT31**	0.00	0.00	0.00	0.00	0.00	0.00	0.00	0.00	0.08	0.00	0.00	0.00	0.00	0.00	0.00	0.00
**GH105**	0.36	0.53	0.71	0.67	0.83	0.25	0.09	0.00	0.32	0.39	0.53	0.75	0.78	0.56	0.61	0.00
**GT23**	0.00	0.00	0.00	0.00	0.00	0.00	0.00	0.00	0.00	0.00	0.00	0.00	0.00	0.00	0.00	0.00
**GH11**	0.00	0.00	0.00	0.00	0.00	0.00	0.00	0.00	0.00	0.00	0.02	0.02	0.04	0.00	0.00	0.00
**GT38**	0.00	0.00	0.00	0.00	0.00	0.00	0.00	0.00	0.08	0.00	0.00	0.00	0.00	0.00	0.00	0.00
**GT43**	0.00	0.00	0.00	0.00	0.00	0.00	0.00	0.00	0.00	0.00	0.00	0.00	0.00	0.00	0.00	0.00
**GH99**	0.00	0.00	0.05	0.00	0.00	0.00	0.00	0.00	0.00	0.00	0.02	0.00	0.00	0.00	0.00	0.00
**GH38**	0.12	0.29	0.25	0.45	0.49	0.04	1.37	0.95	0.81	0.08	0.23	0.33	0.31	0.27	1.37	4.62
**GH109**	0.40	0.24	0.35	0.20	0.49	0.00	0.00	0.05	0.08	0.20	0.28	0.33	0.33	0.25	0.16	0.00
**GH2**	4.86	4.28	4.93	3.67	5.50	1.23	2.28	1.94	2.03	3.87	6.81	7.49	7.37	6.42	8.37	6.27
**GT28**	0.47	0.46	0.33	0.56	0.26	0.55	0.27	0.30	0.37	0.35	1.12	1.21	0.78	0.79	1.29	0.91
**GH42**	0.20	0.24	0.51	0.28	0.21	0.68	1.64	0.75	0.57	0.02	0.57	0.60	0.29	0.41	2.20	4.32
**GT58**	0.00	0.00	0.00	0.00	0.00	0.00	0.00	0.00	0.00	0.00	0.00	0.00	0.00	0.00	0.00	0.00
**GH61**	0.00	0.00	0.00	0.00	0.00	0.00	0.00	0.00	0.00	0.00	0.00	0.00	0.00	0.00	0.00	0.00
**GH80**	0.00	0.00	0.00	0.00	0.00	0.00	0.00	0.00	0.00	0.00	0.00	0.00	0.00	0.00	0.00	0.00
**GH98**	0.00	0.00	0.08	0.00	0.00	0.00	0.00	0.00	0.00	0.00	0.12	0.04	0.12	0.05	0.05	0.00
**GH6**	0.00	0.00	0.00	0.00	0.00	0.00	0.00	0.00	0.00	0.00	0.00	0.00	0.00	0.01	0.00	0.00
**GT1**	0.12	0.02	0.03	0.03	0.02	0.04	0.09	0.10	0.04	0.02	0.07	0.07	0.08	0.12	0.04	0.06
**GH28**	1.26	1.11	1.11	0.73	0.77	0.17	0.09	0.15	0.24	1.13	2.13	1.66	2.31	1.46	1.78	0.30
**GT42**	0.00	0.00	0.00	0.00	0.00	0.00	0.00	0.00	0.00	0.00	0.00	0.00	0.00	0.00	0.00	0.00
**GH128**	0.00	0.00	0.00	0.00	0.00	0.00	0.00	0.00	0.00	0.00	0.00	0.00	0.00	0.00	0.00	0.00
**CE12**	0.08	0.02	0.20	0.14	0.14	0.00	0.09	0.00	0.00	0.37	0.37	0.26	0.33	0.44	0.40	0.00
**CE16**	0.00	0.00	0.00	0.00	0.00	0.00	0.00	0.00	0.00	0.00	0.00	0.00	0.00	0.00	0.00	0.00
**GT89**	0.00	0.00	0.00	0.00	0.00	0.00	0.00	0.00	0.00	0.00	0.00	0.00	0.00	0.00	0.00	0.00
**GT11**	0.08	0.15	0.08	0.08	0.02	0.00	0.00	0.00	0.00	0.04	0.02	0.04	0.16	0.04	0.03	0.00
**GT77**	0.00	0.00	0.00	0.00	0.00	0.00	0.00	0.00	0.16	0.29	0.00	0.00	0.00	0.00	0.00	0.30
**GT55**	0.00	0.00	0.00	0.00	0.00	0.00	0.00	0.00	0.00	0.00	0.00	0.00	0.00	0.00	0.00	0.00
**GT18**	0.00	0.00	0.00	0.00	0.00	0.00	0.00	0.00	0.00	0.00	0.00	0.00	0.00	0.00	0.00	0.00
**GH87**	0.00	0.00	0.00	0.00	0.00	0.00	0.00	0.00	0.00	0.00	0.02	0.02	0.00	0.04	0.00	0.03
**PL5**	0.00	0.00	0.00	0.00	0.00	0.04	0.00	0.00	0.00	0.00	0.00	0.00	0.00	0.00	0.00	0.00
**GH22**	0.00	0.00	0.00	0.00	0.00	0.00	0.00	0.00	0.00	0.00	0.00	0.02	0.00	0.00	0.00	0.00
**CE2**	0.00	0.10	0.05	0.06	0.05	0.00	0.00	0.15	0.08	0.04	0.21	0.18	0.08	0.27	0.16	0.00
**GH120**	0.04	0.12	0.05	0.17	0.02	0.00	0.00	0.05	0.08	0.00	0.09	0.29	0.08	0.07	0.25	0.00
**GH73**	0.87	0.65	0.46	0.65	0.49	0.47	1.00	0.40	0.69	0.63	0.89	1.11	1.29	1.19	1.08	0.82
**GT17**	0.00	0.00	0.00	0.00	0.00	0.00	0.00	0.00	0.00	0.00	0.00	0.04	0.00	0.01	0.00	0.00
**GH124**	0.00	0.00	0.00	0.00	0.00	0.00	0.00	0.00	0.00	0.00	0.00	0.00	0.00	0.00	0.00	0.00
**GH54**	0.00	0.00	0.00	0.00	0.00	0.00	0.00	0.00	0.00	0.00	0.00	0.00	0.04	0.00	0.00	0.00
**GH100**	0.00	0.00	0.00	0.00	0.00	0.00	0.00	0.00	0.00	0.00	0.00	0.00	0.00	0.00	0.00	0.00
**GH113**	0.00	0.00	0.00	0.00	0.02	0.04	0.00	0.00	0.04	0.00	0.02	0.15	0.04	0.01	0.00	0.00
**GT5**	0.47	0.44	0.56	0.56	0.49	0.34	0.09	0.35	0.16	0.35	1.31	1.85	1.12	1.08	0.67	0.24
**GH94**	0.16	0.60	0.43	0.28	0.44	0.04	0.18	0.05	0.28	0.39	1.31	1.94	1.63	1.26	0.55	0.00
**GT66**	0.00	0.00	0.00	0.00	0.00	0.00	0.00	0.00	0.00	0.00	0.02	0.04	0.00	0.01	0.00	0.00
**GT94**	0.00	0.00	0.00	0.00	0.00	0.00	0.00	0.00	0.00	0.00	0.00	0.00	0.00	0.00	0.00	0.00
**GH58**	0.00	0.00	0.00	0.00	0.02	0.00	0.00	0.00	0.08	0.00	0.00	0.00	0.00	0.00	0.00	0.00
**GH1**	0.47	0.70	1.29	0.70	0.46	2.59	2.64	2.19	2.07	0.10	1.45	1.43	0.92	0.63	2.00	0.33
**PL22**	0.00	0.00	0.00	0.00	0.00	0.13	0.00	0.00	0.12	0.00	0.00	0.00	0.00	0.00	0.00	0.00
**GT9**	0.28	0.12	0.15	0.03	0.21	0.55	0.18	0.00	0.16	0.29	0.41	0.37	0.43	0.32	0.51	0.24

CAZY_group	JP-AD-7	JP-AD-8	JP-AD-9	JP-CH-1	JP-CH-2	JP-IN-1	JP-IN-2	JP-IN-3	JP-IN-4	IN-CH-16	MA-AD-1	MA-AD-2	MA-AD-3	MA-AD-4	MA-IN-1	MA-IN-10
**GT76**	0.00	0.00	0.00	0.00	0.00	0.00	0.00	0.00	0.00	0.00	0.00	0.00	0.00	0.00	0.00	0.00
**GT32**	0.20	0.12	0.10	0.08	0.09	0.00	0.00	0.00	0.04	0.12	0.12	0.24	0.16	0.09	0.30	0.55
**GT47**	0.00	0.07	0.08	0.08	0.02	0.04	0.09	0.00	0.16	0.04	0.28	0.42	0.27	0.20	0.45	0.15
**GH84**	0.12	0.02	0.13	0.03	0.12	0.00	0.00	0.00	0.00	0.29	0.25	0.22	0.12	0.13	0.37	0.00
**GH83**	0.00	0.00	0.00	0.00	0.00	0.00	0.00	0.00	0.00	0.00	0.00	0.00	0.00	0.00	0.00	0.00
**GT59**	0.00	0.00	0.00	0.00	0.00	0.00	0.00	0.00	0.00	0.00	0.00	0.00	0.00	0.00	0.00	0.00
**GH34**	0.00	0.00	0.00	0.00	0.00	0.00	0.00	0.00	0.00	0.00	0.00	0.00	0.00	0.00	0.00	0.00
**GT14**	0.00	0.10	0.08	0.06	0.05	0.00	0.00	0.00	0.00	0.04	0.05	0.07	0.10	0.13	0.11	0.49
**GH47**	0.00	0.00	0.00	0.00	0.00	0.00	0.00	0.00	0.00	0.00	0.00	0.00	0.06	0.00	0.00	0.03
**GH97**	0.91	0.82	0.83	0.87	1.21	0.00	0.00	0.00	0.08	1.41	2.48	1.21	3.10	2.07	1.73	0.00
**GH50**	0.08	0.02	0.08	0.03	0.05	0.00	0.00	0.00	0.00	0.00	0.02	0.00	0.04	0.01	0.00	0.00
**GT26**	0.04	0.15	0.23	0.25	0.26	0.38	0.09	0.15	0.24	0.23	0.43	0.46	0.39	0.39	0.49	0.18
**GH18**	0.16	0.51	0.58	0.48	0.60	0.30	0.09	0.20	0.53	0.06	0.66	1.01	0.59	0.66	0.76	2.34
**GH37**	0.00	0.00	0.00	0.00	0.00	0.64	0.00	0.00	0.12	0.02	0.00	0.04	0.00	0.00	0.00	0.24
**PL13**	0.04	0.05	0.03	0.06	0.12	0.00	0.00	0.00	0.00	0.00	0.00	0.00	0.00	0.00	0.00	0.00
**GH27**	0.24	0.22	0.28	0.34	0.35	0.08	0.18	0.15	0.04	0.27	0.50	0.55	0.51	0.36	0.28	0.00
**GT54**	0.00	0.00	0.00	0.00	0.00	0.00	0.00	0.00	0.00	0.00	0.00	0.00	0.00	0.00	0.00	0.00
**GT91**	0.00	0.00	0.00	0.00	0.00	0.00	0.00	0.00	0.00	0.00	0.00	0.00	0.00	0.00	0.00	0.00
**GT72**	0.00	0.00	0.00	0.00	0.00	0.00	0.00	0.00	0.00	0.00	0.00	0.00	0.00	0.00	0.00	0.00
**GH67**	0.12	0.05	0.03	0.08	0.07	0.13	0.09	0.00	0.04	0.25	0.28	0.33	0.20	0.19	0.36	0.00
**GH12**	0.00	0.00	0.00	0.00	0.00	0.00	0.00	0.00	0.00	0.00	0.00	0.02	0.02	0.00	0.00	0.00
**GH91**	0.00	0.05	0.00	0.00	0.00	0.04	0.00	0.00	0.00	0.00	0.00	0.02	0.00	0.00	0.00	0.00
**GT69**	0.00	0.00	0.00	0.00	0.00	0.00	0.00	0.00	0.00	0.00	0.00	0.00	0.00	0.00	0.00	0.00
**GH106**	0.28	0.24	0.33	0.20	0.35	0.00	0.00	0.00	0.00	0.31	0.48	0.22	0.69	0.43	0.42	0.00
**GT62**	0.00	0.00	0.00	0.00	0.00	0.00	0.00	0.00	0.00	0.00	0.00	0.00	0.00	0.00	0.00	0.00
**GH17**	0.00	0.00	0.00	0.00	0.00	0.00	0.00	0.00	0.00	0.00	0.00	0.00	0.00	0.00	0.00	0.00
**GH88**	0.40	0.17	0.53	0.22	0.42	0.08	0.00	0.10	0.65	0.18	0.14	0.24	0.24	0.27	0.15	0.00
**CE5**	0.00	0.00	0.00	0.00	0.00	0.00	0.00	0.00	0.00	0.00	0.00	0.00	0.00	0.00	0.00	0.00
**GT8**	0.24	0.24	0.13	0.31	0.12	0.17	0.09	0.10	0.41	0.08	0.67	0.42	0.35	0.13	0.32	0.24
**GT50**	0.00	0.00	0.00	0.00	0.00	0.00	0.00	0.00	0.00	0.00	0.00	0.00	0.00	0.00	0.00	0.00
**GT20**	0.12	0.15	0.05	0.00	0.07	0.34	0.00	0.15	0.08	0.00	0.02	0.00	0.00	0.00	0.04	0.03
**PL9**	0.08	0.02	0.23	0.06	0.12	0.30	0.18	0.20	0.00	0.02	0.43	0.24	0.33	0.17	0.11	0.00
**GH55**	0.00	0.10	0.03	0.08	0.05	0.00	0.00	0.00	0.00	0.02	0.00	0.02	0.06	0.05	0.01	0.00
**GH64**	0.00	0.00	0.00	0.00	0.00	0.00	0.00	0.00	0.00	0.00	0.00	0.00	0.00	0.00	0.00	0.00
**GH127**	0.47	0.41	0.58	0.48	0.51	0.25	0.27	0.90	0.32	0.57	0.58	0.71	1.00	0.51	1.02	0.03
**GH103**	0.04	0.00	0.00	0.00	0.05	0.34	0.00	0.00	0.04	0.00	0.00	0.05	0.00	0.00	0.03	0.12
**GH3**	2.85	3.97	3.54	2.64	3.50	1.49	2.18	2.14	2.07	3.07	6.66	5.88	7.45	4.96	7.26	5.75
**GT45**	0.00	0.00	0.00	0.00	0.00	0.00	0.00	0.00	0.00	0.00	0.00	0.00	0.00	0.00	0.00	0.00
**GT74**	0.00	0.00	0.00	0.00	0.00	0.00	0.00	0.00	0.00	0.00	0.00	0.00	0.00	0.00	0.00	0.00
**GT90**	0.00	0.00	0.00	0.00	0.00	0.00	0.00	0.00	0.00	0.00	0.00	0.00	0.00	0.04	0.01	0.00
**GH116**	0.00	0.07	0.03	0.06	0.21	0.00	0.00	0.00	0.00	0.00	0.02	0.00	0.04	0.11	0.03	0.00
**GH31**	1.23	0.99	1.79	1.40	1.65	0.89	1.27	2.04	0.89	1.21	2.68	2.69	3.18	2.52	2.65	0.24
**GT12**	0.00	0.00	0.00	0.00	0.00	0.00	0.00	0.00	0.00	0.00	0.00	0.02	0.00	0.00	0.00	0.00
**CE1**	0.43	0.41	0.48	0.53	0.63	0.25	0.09	0.00	0.12	0.18	1.17	0.66	0.80	0.62	0.53	0.18
**GH121**	0.08	0.10	0.03	0.20	0.05	0.00	0.27	0.35	0.08	0.00	0.00	0.00	0.00	0.00	0.44	0.00
**GH79**	0.00	0.00	0.03	0.00	0.00	0.00	0.00	0.00	0.00	0.00	0.04	0.02	0.00	0.03	0.00	0.00
**GT73**	0.00	0.00	0.00	0.00	0.00	0.00	0.00	0.00	0.04	0.00	0.00	0.00	0.00	0.00	0.00	0.00
**GH77**	0.55	0.89	0.68	0.95	0.58	0.47	0.91	0.85	0.49	0.59	1.77	2.17	2.39	1.89	2.98	3.92
**PL17**	0.00	0.07	0.10	0.00	0.05	0.08	0.00	0.00	0.00	0.00	0.00	0.00	0.00	0.00	0.00	0.00
**PL8**	0.28	0.27	0.23	0.25	0.30	0.00	0.00	0.00	0.04	0.00	0.05	0.22	0.02	0.04	0.08	0.00

CAZY_group	JP-AD-7	JP-AD-8	JP-AD-9	JP-CH-1	JP-CH-2	JP-IN-1	JP-IN-2	JP-IN-3	JP-IN-4	IN-CH-16	MA-AD-1	MA-AD-2	MA-AD-3	MA-AD-4	MA-IN-1	MA-IN-10
**CE9**	0.47	0.53	0.40	0.25	0.26	0.55	1.00	0.75	0.37	0.14	0.60	0.99	0.59	0.52	1.02	1.13
**CE15**	0.00	0.02	0.00	0.03	0.02	0.00	0.00	0.00	0.00	0.00	0.00	0.00	0.04	0.00	0.00	0.00
**GT79**	0.00	0.00	0.00	0.00	0.00	0.00	0.00	0.00	0.00	0.00	0.00	0.00	0.00	0.00	0.00	0.00
**GT10**	0.00	0.00	0.00	0.08	0.02	0.00	0.00	0.00	0.08	0.00	0.00	0.00	0.00	0.00	0.00	0.00
**GH119**	0.00	0.00	0.00	0.00	0.00	0.00	0.00	0.00	0.00	0.00	0.00	0.04	0.08	0.00	0.00	0.00
**GH16**	0.32	0.34	0.18	0.20	0.42	0.17	0.00	0.00	0.04	0.16	0.23	0.37	0.45	0.35	0.41	0.00
**GH43**	3.56	2.52	2.63	3.31	3.90	1.19	2.55	4.13	1.58	3.34	5.12	3.31	6.20	4.52	7.57	0.67
**GH51**	0.71	0.46	0.46	0.81	0.53	0.13	0.73	1.14	0.45	1.15	1.68	1.32	1.55	1.73	2.00	2.04
**GT35**	1.03	1.16	1.06	1.23	0.74	0.93	0.46	0.95	0.73	0.80	2.48	3.51	2.79	1.94	3.07	2.62
**GT25**	0.00	0.00	0.00	0.00	0.00	0.00	0.00	0.00	0.00	0.00	0.00	0.02	0.00	0.00	0.01	0.00
**GT2**	6.05	6.09	5.44	4.60	6.17	6.67	4.92	6.76	3.57	4.87	11.45	11.91	13.22	10.05	15.03	18.92
**GT51**	1.26	0.85	1.21	0.95	0.97	1.40	1.73	1.39	0.77	1.41	2.61	3.31	3.06	2.15	3.44	4.35
**GH107**	0.00	0.00	0.00	0.00	0.00	0.00	0.00	0.00	0.00	0.00	0.00	0.00	0.00	0.00	0.00	0.00
**GH7**	0.00	0.00	0.00	0.00	0.00	0.00	0.00	0.00	0.00	0.00	0.00	0.00	0.00	0.00	0.00	0.00
**GT7**	0.00	0.00	0.00	0.00	0.00	0.00	0.00	0.00	0.00	0.00	0.00	0.00	0.00	0.00	0.00	0.00
**GT36**	0.00	0.00	0.00	0.00	0.00	0.00	0.00	0.00	0.00	0.00	0.00	0.00	0.00	0.00	0.00	0.00
**PL7**	0.00	0.00	0.00	0.00	0.00	0.00	0.00	0.00	0.00	0.00	0.00	0.00	0.02	0.00	0.00	0.00
**GH110**	0.20	0.00	0.13	0.11	0.16	0.00	0.00	0.00	0.12	0.14	0.00	0.04	0.04	0.01	0.18	0.00
**GT21**	0.00	0.00	0.00	0.00	0.00	0.00	0.00	0.00	0.00	0.00	0.00	0.00	0.00	0.00	0.00	0.00
**GH62**	0.00	0.00	0.00	0.00	0.00	0.00	0.00	0.00	0.00	0.00	0.00	0.00	0.02	0.00	0.00	0.00
**GH125**	0.20	0.29	0.03	0.31	0.26	0.00	0.46	0.30	0.12	0.31	0.21	0.27	0.25	0.31	0.30	0.61
**GH117**	0.12	0.10	0.03	0.08	0.19	0.00	0.00	0.00	0.00	0.00	0.02	0.02	0.04	0.03	0.00	0.00
**GH68**	0.00	0.00	0.00	0.03	0.00	0.00	0.00	0.00	0.00	0.00	0.02	0.04	0.00	0.01	0.00	0.06
**GH85**	0.04	0.00	0.08	0.00	0.00	0.00	0.09	0.50	0.00	0.18	0.02	0.29	0.12	0.23	0.33	1.98
**GH92**	2.13	2.03	1.37	1.46	2.39	0.04	0.55	0.10	0.00	1.35	0.78	1.57	1.51	1.61	0.90	0.15
**GH40**	0.00	0.00	0.00	0.00	0.00	0.00	0.00	0.00	0.00	0.00	0.00	0.00	0.00	0.00	0.00	0.00
**GT49**	0.00	0.00	0.00	0.00	0.00	0.00	0.00	0.00	0.00	0.00	0.00	0.00	0.00	0.01	0.00	0.00
**GH112**	0.16	0.31	0.33	0.20	0.28	0.08	0.46	0.65	0.41	0.10	0.34	0.66	0.33	0.20	0.45	1.79
**GT67**	0.00	0.00	0.00	0.00	0.00	0.00	0.00	0.00	0.00	0.00	0.00	0.00	0.00	0.00	0.00	0.00
**CE8**	0.24	0.17	0.28	0.08	0.16	0.42	0.00	0.25	0.12	0.27	0.78	0.40	0.51	0.47	0.20	0.03
**GT86**	0.00	0.00	0.00	0.00	0.00	0.00	0.00	0.00	0.00	0.00	0.00	0.00	0.00	0.00	0.00	0.00
**GH101**	0.16	0.12	0.00	0.11	0.07	0.00	0.46	1.29	0.08	0.00	0.05	0.02	0.00	0.00	0.09	0.03
**GH81**	0.04	0.00	0.00	0.00	0.00	0.08	0.00	0.00	0.00	0.00	0.00	0.02	0.02	0.00	0.00	0.00
**GT93**	0.00	0.00	0.00	0.00	0.00	0.00	0.00	0.00	0.00	0.00	0.00	0.00	0.00	0.00	0.00	0.00
**GT63**	0.00	0.00	0.00	0.00	0.00	0.00	0.00	0.00	0.00	0.00	0.00	0.00	0.00	0.00	0.00	0.00
**CE11**	0.28	0.24	0.30	0.11	0.30	0.17	0.00	0.05	0.08	0.47	0.64	0.44	0.57	0.55	0.55	0.03
**GT19**	0.12	0.15	0.05	0.06	0.21	0.08	0.00	0.00	0.16	0.35	0.35	0.55	0.55	0.28	0.41	0.12
**GT33**	0.00	0.00	0.00	0.00	0.00	0.00	0.00	0.00	0.00	0.00	0.00	0.00	0.02	0.00	0.00	0.00
**GH21**	0.00	0.00	0.00	0.00	0.00	0.00	0.00	0.00	0.00	0.00	0.00	0.00	0.00	0.00	0.00	0.00
**GT68**	0.00	0.00	0.00	0.00	0.00	0.00	0.00	0.00	0.00	0.00	0.00	0.00	0.00	0.00	0.00	0.00
**GH89**	0.24	0.05	0.15	0.06	0.19	0.13	0.00	0.00	0.00	0.25	0.28	0.20	0.47	0.29	0.37	0.00
**GT82**	0.00	0.00	0.00	0.00	0.00	0.00	0.00	0.00	0.00	0.00	0.00	0.00	0.00	0.00	0.00	0.00
**GH30**	0.24	0.07	0.38	0.22	0.35	0.00	0.09	0.35	0.00	0.14	0.21	0.18	0.31	0.16	0.91	0.94
**GH20**	1.82	1.62	1.21	0.81	1.60	0.08	0.27	0.35	0.28	1.51	1.44	1.57	1.71	1.08	2.45	5.02
**GH52**	0.00	0.00	0.00	0.00	0.00	0.00	0.00	0.00	0.00	0.00	0.00	0.00	0.00	0.00	0.00	0.00
**GT84**	0.00	0.02	0.00	0.03	0.00	0.25	0.00	0.00	0.37	0.00	0.12	0.05	0.16	0.04	0.00	0.00
**GT15**	0.00	0.00	0.00	0.00	0.00	0.00	0.00	0.00	0.00	0.00	0.00	0.00	0.00	0.00	0.00	0.00
**GH93**	0.00	0.00	0.00	0.00	0.00	0.00	0.00	0.00	0.00	0.00	0.00	0.00	0.10	0.00	0.01	0.00
**PL16**	0.00	0.00	0.00	0.00	0.00	0.00	0.00	0.00	0.00	0.00	0.00	0.00	0.00	0.00	0.00	0.00
**GH26**	0.12	0.12	0.08	0.14	0.23	0.13	0.27	0.00	0.00	0.10	0.35	0.33	0.43	0.41	0.44	0.00

CAZY_group	JP-AD-7	JP-AD-8	JP-AD-9	JP-CH-1	JP-CH-2	JP-IN-1	JP-IN-2	JP-IN-3	JP-IN-4	IN-CH-16	MA-AD-1	MA-AD-2	MA-AD-3	MA-AD-4	MA-IN-1	MA-IN-10
GT75	0.00	0.00	0.00	0.00	0.00	0.00	0.00	0.00	0.00	0.00	0.00	0.00	0.00	0.00	0.00	0.00
GH53	0.04	0.24	0.28	0.00	0.19	0.30	0.73	0.20	0.08	0.14	0.55	0.68	0.47	0.47	0.24	0.21
GT88	0.00	0.00	0.00	0.00	0.00	0.00	0.00	0.00	0.00	0.00	0.00	0.00	0.00	0.00	0.00	0.00
GT24	0.00	0.00	0.00	0.00	0.00	0.00	0.00	0.00	0.00	0.00	0.00	0.00	0.00	0.00	0.00	0.00
GT81	0.00	0.00	0.00	0.00	0.00	0.00	0.00	0.00	0.00	0.00	0.00	0.00	0.00	0.00	0.00	0.00
GH4	0.12	0.10	0.18	0.08	0.16	1.49	0.91	0.25	0.69	0.04	0.14	0.40	0.37	0.13	0.29	0.46
GT78	0.00	0.00	0.00	0.00	0.00	0.00	0.00	0.00	0.00	0.00	0.00	0.00	0.00	0.00	0.00	0.00
GH8	0.08	0.05	0.05	0.14	0.05	0.34	0.00	0.00	0.00	0.10	0.30	0.20	0.51	0.21	0.25	0.09
GH70	0.00	0.00	0.00	0.11	0.00	0.00	0.00	0.00	0.00	0.00	0.09	0.24	0.06	0.00	0.03	0.27
GH122	0.00	0.00	0.00	0.00	0.00	0.00	0.00	0.00	0.00	0.00	0.00	0.00	0.00	0.00	0.00	0.00
GH74	0.00	0.05	0.00	0.00	0.02	0.00	0.00	0.00	0.00	0.00	0.04	0.26	0.24	0.19	0.00	0.00
GT41	0.00	0.00	0.00	0.00	0.00	0.04	0.00	0.00	0.08	0.00	0.00	0.07	0.00	0.01	0.00	0.00
PL1	0.43	0.19	0.38	0.17	0.19	0.00	0.00	0.00	0.00	0.27	0.46	0.20	0.55	0.58	0.29	0.00
GH15	0.00	0.17	0.10	0.06	0.02	0.00	0.00	0.00	0.00	0.00	0.02	0.02	0.00	0.00	0.00	0.00
GH32	0.79	0.56	0.78	0.42	0.72	0.93	0.73	0.80	0.81	0.70	0.99	1.41	0.88	0.82	1.75	1.46
GH44	0.00	0.00	0.00	0.00	0.00	0.00	0.00	0.00	0.00	0.00	0.04	0.13	0.06	0.01	0.00	0.00
GT6	0.00	0.00	0.00	0.03	0.00	0.00	0.00	0.00	0.00	0.00	0.00	0.00	0.00	0.00	0.00	0.00
GH13	3.83	3.75	3.67	3.62	3.36	3.02	6.55	4.63	3.21	3.19	9.98	12.54	11.55	8.01	12.30	15.21
GT80	0.00	0.00	0.00	0.00	0.00	0.21	0.00	0.00	0.08	0.00	0.00	0.02	0.00	0.00	0.01	0.06
GH71	0.00	0.00	0.00	0.00	0.00	0.00	0.00	0.00	0.00	0.00	0.00	0.00	0.00	0.00	0.00	0.00
GH63	0.20	0.19	0.13	0.11	0.28	0.17	0.18	0.00	0.04	0.08	0.14	0.22	0.06	0.31	0.18	0.21
PL21	0.16	0.07	0.03	0.03	0.02	0.00	0.00	0.00	0.00	0.00	0.00	0.00	0.00	0.01	0.00	0.00
GH65	0.36	0.24	0.28	0.28	0.21	0.25	0.55	0.25	0.49	0.00	0.19	0.22	0.20	0.08	0.29	0.06
CE4	0.47	0.56	0.61	0.36	0.37	0.93	0.36	0.45	0.57	0.35	0.99	1.28	0.92	0.86	0.74	0.21
CE14	0.04	0.00	0.00	0.00	0.00	0.04	0.00	0.00	0.00	0.02	0.00	0.00	0.06	0.03	0.00	0.00
GH115	0.32	0.19	0.25	0.28	0.28	0.08	0.09	0.00	0.00	0.49	0.66	0.33	0.76	0.58	0.54	0.00
GH104	0.00	0.00	0.00	0.00	0.00	0.04	0.00	0.00	0.12	0.00	0.00	0.00	0.00	0.00	0.00	0.00
GH111	0.00	0.00	0.00	0.00	0.00	0.00	0.00	0.00	0.04	0.00	0.00	0.00	0.00	0.00	0.00	0.00
GH95	0.91	0.46	0.68	0.48	0.77	0.00	0.27	0.20	0.16	0.92	1.35	1.35	2.00	1.42	2.47	2.07
GT70	0.00	0.00	0.00	0.00	0.00	0.00	0.00	0.00	0.00	0.00	0.00	0.00	0.00	0.00	0.00	0.00
CE10	0.28	0.17	0.20	0.25	0.30	0.51	0.00	0.20	0.37	0.02	0.19	0.49	0.31	0.08	0.45	0.24
GH10	0.08	0.10	0.08	0.14	0.23	0.00	0.00	0.00	0.00	0.33	0.41	0.20	0.53	0.33	0.33	0.00
GH130	0.16	0.39	0.25	0.28	0.42	0.00	0.00	0.00	0.08	0.25	0.39	0.84	0.82	0.55	0.20	0.00
PL20	0.00	0.00	0.00	0.00	0.00	0.00	0.00	0.00	0.00	0.00	0.00	0.00	0.00	0.00	0.00	0.00
GH69	0.00	0.00	0.00	0.00	0.00	0.00	0.00	0.00	0.00	0.00	0.00	0.00	0.00	0.00	0.00	0.00
PL2	0.00	0.00	0.00	0.00	0.00	0.00	0.00	0.00	0.00	0.00	0.00	0.00	0.00	0.00	0.00	0.00
GH49	0.00	0.00	0.00	0.00	0.00	0.00	0.00	0.00	0.00	0.00	0.00	0.00	0.00	0.00	0.00	0.00
GH25	0.47	0.48	0.58	0.25	0.28	0.21	0.82	0.25	0.69	1.10	1.65	1.55	1.33	1.14	2.28	1.40
GH59	0.00	0.00	0.03	0.00	0.02	0.08	0.09	0.05	0.00	0.00	0.00	0.02	0.08	0.01	0.00	0.00
GH35	0.59	0.46	0.48	0.42	0.60	0.30	0.64	0.15	0.16	0.47	0.53	0.37	0.71	0.48	0.86	0.27
GT39	0.00	0.00	0.00	0.00	0.00	0.00	0.00	0.10	0.00	0.00	0.02	0.11	0.08	0.01	0.00	0.03
GT57	0.00	0.00	0.00	0.00	0.00	0.00	0.00	0.00	0.00	0.00	0.00	0.00	0.00	0.00	0.00	0.00
GH129	0.04	0.00	0.05	0.08	0.02	0.00	0.09	0.10	0.16	0.00	0.02	0.02	0.02	0.01	0.28	1.46
GT22	0.00	0.00	0.00	0.00	0.00	0.00	0.00	0.00	0.00	0.00	0.00	0.00	0.00	0.00	0.00	0.00
PL12	0.08	0.19	0.05	0.11	0.09	0.00	0.09	0.00	0.00	0.02	0.02	0.16	0.04	0.04	0.05	0.00
PL18	0.00	0.00	0.00	0.00	0.00	0.00	0.00	0.00	0.00	0.00	0.00	0.00	0.00	0.00	0.00	0.00
GT92	0.00	0.00	0.00	0.00	0.00	0.00	0.00	0.00	0.00	0.00	0.00	0.00	0.00	0.00	0.00	0.00
GT44	0.00	0.00	0.03	0.06	0.00	0.00	0.00	0.00	0.00	0.00	0.00	0.02	0.00	0.00	0.01	0.43
GT29	0.00	0.00	0.00	0.00	0.00	0.00	0.00	0.00	0.00	0.00	0.00	0.00	0.00	0.00	0.00	0.00
GT13	0.00	0.00	0.00	0.00	0.00	0.00	0.00	0.00	0.00	0.00	0.00	0.00	0.00	0.00	0.00	0.00

Normalized_abundance_2

CAZY_group	JP-AD-7	JP-AD-8	JP-AD-9	JP-CH-1	JP-CH-2	JP-IN-1	JP-IN-2	JP-IN-3	JP-IN-4	IN-CH-16	MA-AD-1	MA-AD-2	MA-AD-3	MA-AD-4	MA-IN-1	MA-IN-10
**GT52**	0.00	0.00	0.00	0.00	0.00	0.00	0.00	0.00	0.00	0.00	0.00	0.00	0.00	0.00	0.00	0.00
**GT64**	0.00	0.00	0.00	0.00	0.00	0.00	0.00	0.00	0.00	0.00	0.00	0.00	0.00	0.00	0.00	0.00
**GT85**	0.00	0.00	0.00	0.00	0.00	0.00	0.00	0.00	0.00	0.00	0.00	0.00	0.00	0.00	0.00	0.00
**GT16**	0.00	0.00	0.00	0.00	0.00	0.00	0.00	0.00	0.00	0.00	0.00	0.00	0.00	0.00	0.00	0.00
**GH56**	0.00	0.00	0.00	0.00	0.00	0.00	0.00	0.00	0.00	0.00	0.00	0.00	0.00	0.00	0.00	0.00
**GT60**	0.00	0.00	0.00	0.00	0.00	0.00	0.00	0.00	0.00	0.00	0.00	0.00	0.00	0.00	0.00	0.00
**GH41**	0.00	0.00	0.00	0.00	0.00	0.00	0.00	0.00	0.00	0.00	0.00	0.00	0.00	0.00	0.00	0.00
**CE7**	0.20	0.12	0.10	0.17	0.19	0.04	0.09	0.10	0.08	0.31	0.14	0.05	0.20	0.24	0.28	0.00
**GH39**	0.00	0.05	0.05	0.08	0.05	0.17	0.00	0.00	0.04	0.02	0.19	0.16	0.16	0.11	0.01	0.00
**GT30**	0.43	0.39	0.10	0.14	0.37	0.42	0.09	0.00	0.28	0.43	0.51	0.35	0.33	0.35	0.61	0.46
**PL15**	0.04	0.02	0.10	0.11	0.07	0.00	0.00	0.00	0.00	0.06	0.00	0.00	0.02	0.00	0.07	0.00
**GH102**	0.00	0.02	0.05	0.00	0.05	0.64	0.00	0.10	0.08	0.02	0.05	0.13	0.02	0.07	0.01	0.09

CAZY_group	MA-IN-11	MA-IN-12	MA-IN-13	MA-IN-14	MA-IN-15	MA-IN-16	MA-IN-17	MA-IN-18	MA-IN-2	MA-IN-3	MA-IN-4	MA-IN-5	MA-IN-6	MA-IN-7	MA-IN-8	MA-IN-9
GH29	4.20	3.97	2.06	3.02	3.55	2.31	1.91	2.64	1.84	1.82	1.16	2.46	2.46	3.20	2.85	3.24
PL4	0.00	0.00	0.00	0.00	0.00	0.00	0.00	0.00	0.01	0.00	0.00	0.00	0.00	0.00	0.00	0.00
GH23	2.07	2.07	1.93	2.77	2.16	2.23	1.82	2.01	1.76	2.11	2.06	2.86	2.90	2.25	2.94	2.28
CE6	0.00	0.00	0.35	0.01	0.20	0.47	0.67	0.39	0.21	0.34	0.41	0.00	0.00	0.10	0.00	0.00
GH72	0.41	0.49	0.50	0.40	0.32	0.38	0.56	0.38	0.40	0.37	0.43	0.53	0.42	0.41	0.68	0.37
GH114	0.00	0.00	0.00	0.00	0.00	0.00	0.00	0.00	0.00	0.00	0.00	0.00	0.00	0.00	0.00	0.00
GH78	0.00	0.00	0.83	1.92	1.26	0.27	0.33	0.44	0.39	0.98	0.78	0.83	0.89	0.26	0.02	0.13
PL19	0.00	0.00	0.00	0.00	0.00	0.00	0.00	0.00	0.00	0.00	0.00	0.00	0.00	0.00	0.00	0.00
GT27	0.00	0.00	0.00	0.00	0.00	0.00	0.00	0.00	0.00	0.00	0.00	0.00	0.00	0.00	0.00	0.00
GH5	2.67	2.59	1.55	1.28	1.49	2.00	2.53	1.96	1.96	1.93	1.79	2.07	2.51	2.74	1.72	2.12
GT40	0.00	0.00	0.00	0.00	0.00	0.00	0.00	0.00	0.00	0.00	0.00	0.00	0.00	0.00	0.00	0.00
CE13	0.00	0.00	0.00	0.00	0.00	0.00	0.00	0.00	0.00	0.00	0.00	0.00	0.00	0.00	0.00	0.00
GT37	0.00	0.00	0.00	0.00	0.00	0.00	0.00	0.00	0.00	0.00	0.00	0.00	0.00	0.00	0.00	0.00
GH33	3.71	3.65	0.38	0.68	2.50	1.09	0.77	2.45	1.06	0.98	0.71	1.49	1.69	3.07	2.66	3.40
GT65	0.00	0.00	0.00	0.00	0.00	0.00	0.00	0.00	0.00	0.00	0.00	0.00	0.00	0.00	0.00	0.00
GH86	0.00	0.00	0.00	0.00	0.00	0.00	0.00	0.00	0.00	0.00	0.00	0.00	0.00	0.00	0.00	0.00
GH123	0.00	0.00	0.00	0.06	0.18	0.01	0.02	0.00	0.00	0.00	0.00	0.00	0.00	0.00	0.00	0.00
GH96	0.00	0.00	0.00	0.00	0.00	0.00	0.00	0.00	0.00	0.00	0.00	0.00	0.00	0.00	0.00	0.00
GH14	0.00	0.00	0.00	0.00	0.00	0.00	0.00	0.00	0.00	0.00	0.00	0.00	0.00	0.00	0.00	0.00
CE3	0.00	0.00	0.00	0.00	0.00	0.00	0.00	0.00	0.00	0.00	0.00	0.00	0.00	0.00	0.00	0.00
PL10	0.00	0.00	0.12	0.00	0.13	0.40	0.29	0.16	0.22	0.13	0.15	0.00	0.00	0.10	0.00	0.00
GT48	0.00	0.00	0.00	0.00	0.00	0.00	0.00	0.00	0.00	0.00	0.00	0.00	0.00	0.00	0.00	0.00
PL6	0.00	0.00	0.00	0.00	0.00	0.00	0.00	0.00	0.00	0.00	0.00	0.00	0.00	0.00	0.00	0.00
GT83	0.03	0.00	0.38	0.07	0.84	0.74	0.40	0.35	0.36	0.32	0.49	0.20	0.15	0.18	0.09	0.03
GH126	0.00	0.00	0.00	0.00	0.00	0.00	0.00	0.00	0.00	0.00	0.00	0.00	0.00	0.00	0.00	0.00
GH9	0.00	0.00	0.00	0.00	0.10	0.07	0.05	0.04	0.02	0.00	0.03	0.02	0.00	0.02	0.00	0.00
GH75	0.00	0.00	0.00	0.00	0.00	0.00	0.00	0.00	0.00	0.00	0.00	0.00	0.00	0.00	0.00	0.00
GT71	0.00	0.00	0.00	0.00	0.00	0.00	0.00	0.00	0.00	0.00	0.00	0.00	0.00	0.00	0.00	0.00
GH46	0.00	0.00	0.00	0.00	0.00	0.00	0.00	0.00	0.00	0.00	0.00	0.00	0.00	0.00	0.00	0.00
GT61	0.00	0.00	0.00	0.00	0.00	0.00	0.00	0.00	0.00	0.00	0.00	0.00	0.00	0.00	0.00	0.00
GT3	0.02	0.00	0.27	0.00	0.30	0.26	0.31	0.21	0.22	0.26	0.22	0.00	0.00	0.07	0.02	0.00
PL3	0.00	0.00	0.00	0.00	0.00	0.00	0.00	0.00	0.00	0.00	0.00	0.00	0.00	0.00	0.00	0.00
GH108	0.00	0.00	0.12	0.02	0.03	0.03	0.00	0.00	0.02	0.00	0.00	0.00	0.00	0.00	0.00	0.00
GT56	0.02	0.00	0.00	0.01	0.01	0.03	0.00	0.00	0.00	0.00	0.00	0.20	0.02	0.03	0.00	0.03
PL11	0.00	0.00	0.17	0.01	0.37	0.23	0.07	0.06	0.23	0.24	0.32	0.00	0.00	0.03	0.00	0.00
GH45	0.00	0.00	0.00	0.00	0.00	0.00	0.00	0.00	0.00	0.00	0.00	0.00	0.00	0.00	0.00	0.00
GT34	0.02	0.05	0.05	0.03	0.01	0.00	0.01	0.01	0.07	0.03	0.10	0.00	0.02	0.00	0.00	0.00
PL14	0.00	0.00	0.00	0.00	0.00	0.00	0.00	0.00	0.00	0.00	0.00	0.00	0.00	0.00	0.00	0.00
GH19	0.00	0.00	0.00	0.01	0.00	0.03	0.02	0.01	0.03	0.08	0.12	0.02	0.02	0.02	0.00	0.05
GH118	0.00	0.00	0.00	0.00	0.00	0.00	0.00	0.00	0.00	0.00	0.00	0.00	0.00	0.00	0.00	0.00
GH82	0.00	0.00	0.00	0.00	0.00	0.00	0.00	0.00	0.00	0.00	0.00	0.00	0.00	0.00	0.00	0.00
GH76	0.00	0.00	0.00	0.00	0.49	0.04	0.02	0.02	0.00	0.00	0.00	0.00	0.00	0.00	0.00	0.00
GT4	8.14	9.32	6.36	6.87	6.94	5.36	5.90	5.84	6.11	7.14	5.37	7.21	5.93	7.24	9.71	6.50
GT87	0.05	0.03	0.03	0.04	0.00	0.00	0.04	0.02	0.03	0.03	0.00	0.00	0.00	0.02	0.00	0.00
GH24	0.05	0.00	0.36	0.03	0.16	0.19	0.25	0.29	0.17	0.26	0.15	0.17	0.05	0.13	0.09	0.16
GH90	0.00	0.00	0.00	0.00	0.00	0.00	0.00	0.00	0.00	0.00	0.00	0.00	0.00	0.00	0.00	0.00
GH57	0.00	0.00	0.29	0.02	0.35	0.30	0.37	0.24	0.14	0.16	0.41	0.00	0.00	0.07	0.00	0.00
GH36	1.84	2.59	4.02	5.50	2.64	2.76	4.14	2.98	3.49	4.15	2.87	4.07	3.00	2.48	2.40	2.52
GT46	0.00	0.00	0.00	0.00	0.00	0.00	0.00	0.00	0.00	0.00	0.00	0.00	0.00	0.00	0.00	0.00
GH66	0.08	0.08	0.11	0.18	0.07	0.12	0.25	0.21	0.12	0.21	0.12	0.39	0.22	0.10	1.03	0.08

CAZY_group	MA-IN-11	MA-IN-12	MA-IN-13	MA-IN-14	MA-IN-15	MA-IN-16	MA-IN-17	MA-IN-18	MA-IN-2	MA-IN-3	MA-IN-4	MA-IN-5	MA-IN-6	MA-IN-7	MA-IN-8	MA-IN-9
GH48	0.00	0.00	0.00	0.00	0.00	0.00	0.00	0.00	0.00	0.00	0.00	0.00	0.00	0.00	0.00	0.00
GT53	0.00	0.00	0.00	0.00	0.00	0.00	0.00	0.00	0.00	0.00	0.00	0.00	0.00	0.00	0.00	0.00
GH60	0.00	0.00	0.00	0.00	0.00	0.00	0.00	0.00	0.00	0.00	0.00	0.00	0.00	0.00	0.00	0.00
GT31	0.00	0.00	0.00	0.00	0.00	0.01	0.00	0.00	0.00	0.00	0.00	0.00	0.00	0.00	0.00	0.00
GH105	0.00	0.00	0.93	0.02	0.91	0.79	0.37	0.21	0.70	0.48	0.56	0.00	0.00	0.31	0.00	0.05
GT23	0.00	0.00	0.00	0.00	0.01	0.00	0.00	0.00	0.00	0.00	0.02	0.00	0.00	0.00	0.00	0.00
GH11	0.00	0.00	0.00	0.00	0.00	0.00	0.01	0.01	0.00	0.00	0.00	0.00	0.00	0.00	0.00	0.00
GT38	0.00	0.00	0.00	0.00	0.00	0.00	0.00	0.00	0.00	0.00	0.00	0.00	0.00	0.00	0.00	0.00
GT43	0.00	0.00	0.00	0.00	0.00	0.00	0.00	0.00	0.00	0.00	0.00	0.00	0.00	0.00	0.00	0.00
GH99	0.00	0.00	0.02	0.00	0.00	0.00	0.00	0.00	0.00	0.00	0.00	0.00	0.00	0.00	0.00	0.00
GH38	7.33	7.61	1.53	2.57	2.03	1.53	1.67	2.17	2.11	1.40	0.39	3.59	4.74	3.85	3.46	6.31
GH109	0.02	0.00	0.38	0.01	0.61	0.41	0.07	0.04	0.14	0.05	0.20	0.00	0.00	0.08	0.00	0.03
GH2	6.03	5.79	8.30	6.54	10.83	8.04	8.13	7.68	7.26	9.62	8.66	3.78	4.71	6.72	4.61	5.25
GT28	1.24	1.37	1.17	1.42	1.06	1.11	1.27	1.34	1.12	1.30	1.12	1.49	1.31	1.32	1.32	1.01
GH42	5.16	4.61	2.81	4.59	2.18	1.49	2.55	2.66	2.85	2.33	1.41	2.97	4.51	4.32	5.53	3.90
GT58	0.00	0.00	0.00	0.00	0.00	0.00	0.00	0.00	0.00	0.00	0.00	0.00	0.00	0.00	0.00	0.00
GH61	0.00	0.00	0.00	0.00	0.00	0.00	0.00	0.00	0.00	0.00	0.00	0.00	0.00	0.00	0.00	0.00
GH80	0.00	0.00	0.00	0.00	0.00	0.00	0.00	0.00	0.00	0.00	0.00	0.00	0.00	0.00	0.00	0.00
GH98	0.00	0.00	0.03	0.00	0.02	0.03	0.06	0.02	0.02	0.03	0.07	0.07	0.00	0.00	0.00	0.00
GH6	0.00	0.00	0.00	0.00	0.00	0.00	0.00	0.00	0.00	0.00	0.00	0.02	0.00	0.00	0.00	0.00
GT1	0.00	0.00	0.08	0.05	0.07	0.04	0.13	0.10	0.06	0.00	0.09	0.11	0.05	0.02	0.02	0.00
GH28	0.61	0.64	1.43	0.42	1.47	1.65	2.14	1.24	1.52	1.43	2.19	0.29	0.45	0.86	0.47	0.34
GT42	0.00	0.00	0.00	0.00	0.00	0.00	0.00	0.00	0.00	0.00	0.00	0.00	0.00	0.00	0.00	0.00
GH128	0.00	0.00	0.00	0.00	0.00	0.00	0.00	0.00	0.00	0.00	0.00	0.00	0.00	0.00	0.00	0.00
CE12	0.00	0.00	0.26	0.01	0.12	0.36	0.21	0.14	0.30	0.32	0.60	0.00	0.00	0.08	0.00	0.00
CE16	0.00	0.00	0.00	0.00	0.00	0.00	0.00	0.00	0.00	0.00	0.00	0.00	0.00	0.00	0.00	0.00
GT89	0.00	0.00	0.00	0.00	0.00	0.00	0.00	0.00	0.00	0.00	0.00	0.00	0.00	0.00	0.00	0.00
GT11	0.00	0.00	0.05	0.01	0.14	0.03	0.02	0.04	0.03	0.16	0.12	0.00	0.00	0.00	0.00	0.00
GT77	0.22	0.00	0.00	0.06	0.10	0.10	0.00	0.00	0.00	0.00	0.00	0.94	0.42	0.05	0.16	0.40
GT55	0.00	0.00	0.00	0.00	0.00	0.00	0.00	0.00	0.00	0.00	0.00	0.00	0.00	0.00	0.00	0.00
GT18	0.00	0.00	0.00	0.00	0.00	0.00	0.00	0.00	0.00	0.00	0.00	0.00	0.00	0.00	0.00	0.00
GH87	0.08	0.04	0.00	0.11	0.00	0.00	0.04	0.01	0.00	0.11	0.07	0.37	0.02	0.03	0.75	0.00
PL5	0.00	0.00	0.00	0.00	0.00	0.00	0.00	0.00	0.00	0.00	0.00	0.00	0.00	0.00	0.00	0.00
GH22	0.00	0.00	0.00	0.00	0.00	0.00	0.00	0.00	0.00	0.00	0.00	0.00	0.00	0.00	0.00	0.00
CE2	0.00	0.00	0.12	0.34	0.06	0.14	0.30	0.18	0.08	0.24	0.19	0.11	0.00	0.03	0.00	0.00
GH120	0.00	0.00	0.57	0.92	0.10	0.04	0.10	0.09	0.51	0.00	0.14	0.00	0.00	0.00	0.00	0.00
GH73	0.53	0.53	1.02	1.39	0.89	1.07	1.38	0.85	0.73	0.69	0.48	0.97	0.89	0.90	1.55	0.66
GT17	0.00	0.00	0.00	0.00	0.00	0.00	0.00	0.00	0.00	0.00	0.00	0.00	0.00	0.00	0.00	0.00
GH124	0.00	0.00	0.00	0.00	0.00	0.00	0.00	0.00	0.00	0.00	0.00	0.00	0.00	0.00	0.00	0.00
GH54	0.00	0.00	0.00	0.00	0.00	0.00	0.00	0.00	0.00	0.00	0.00	0.00	0.00	0.00	0.00	0.00
GH100	0.00	0.00	0.00	0.00	0.02	0.00	0.00	0.01	0.00	0.00	0.00	0.00	0.00	0.00	0.00	0.00
GH113	0.00	0.00	0.00	0.00	0.02	0.00	0.01	0.00	0.00	0.00	0.00	0.00	0.00	0.00	0.00	0.00
GT5	0.18	0.16	0.63	0.35	0.54	0.72	0.57	0.56	0.61	0.53	0.70	0.35	0.60	0.26	0.52	0.32
GH94	0.00	0.00	0.59	0.07	0.30	0.77	0.70	0.39	0.66	0.93	0.99	0.40	0.55	0.15	0.00	0.00
GT66	0.00	0.00	0.02	0.00	0.00	0.00	0.01	0.02	0.00	0.00	0.07	0.00	0.00	0.00	0.00	0.00
GT94	0.00	0.00	0.00	0.00	0.00	0.00	0.00	0.00	0.00	0.00	0.00	0.00	0.00	0.00	0.00	0.00
GH58	0.00	0.00	0.00	0.00	0.00	0.00	0.00	0.00	0.00	0.00	0.00	0.00	0.00	0.00	0.00	0.00
GH1	1.46	1.23	1.38	3.42	1.18	0.86	2.19	1.33	1.33	2.17	1.56	4.88	2.68	0.85	1.36	0.64
PL22	0.00	0.00	0.00	0.00	0.00	0.00	0.00	0.00	0.00	0.00	0.00	0.00	0.00	0.00	0.00	0.00
GT9	0.05	0.00	0.57	0.19	0.51	0.55	0.61	0.63	0.41	0.56	0.83	0.35	0.35	0.46	0.19	0.03

CAZY_group	MA-IN-11	MA-IN-12	MA-IN-13	MA-IN-14	MA-IN-15	MA-IN-16	MA-IN-17	MA-IN-18	MA-IN-2	MA-IN-3	MA-IN-4	MA-IN-5	MA-IN-6	MA-IN-7	MA-IN-8	MA-IN-9
**GT76**	0.00	0.00	0.00	0.00	0.00	0.00	0.00	0.00	0.00	0.00	0.00	0.00	0.00	0.00	0.00	0.00
**GT32**	0.68	0.80	0.12	0.06	0.27	0.29	0.45	0.17	0.21	0.71	0.10	0.06	0.02	0.15	0.31	0.13
**GT47**	0.41	0.43	0.21	0.67	0.36	0.33	0.39	0.39	0.39	0.53	0.37	0.50	0.67	0.57	0.49	0.29
**GH84**	0.00	0.00	0.41	0.04	0.84	0.48	0.25	1.16	0.75	0.40	0.54	0.02	0.02	0.59	0.07	0.77
**GH83**	0.00	0.00	0.00	0.00	0.00	0.00	0.00	0.00	0.00	0.00	0.00	0.00	0.00	0.00	0.00	0.00
**GT59**	0.00	0.00	0.00	0.00	0.00	0.00	0.00	0.00	0.00	0.00	0.00	0.00	0.00	0.00	0.00	0.00
**GH34**	0.00	0.00	0.00	0.00	0.00	0.00	0.00	0.00	0.00	0.00	0.00	0.00	0.00	0.00	0.00	0.00
**GT14**	0.00	0.00	0.05	0.11	0.12	0.18	0.53	0.23	0.07	0.32	0.10	0.02	0.25	0.28	0.64	0.00
**GH47**	0.05	0.05	0.03	0.00	0.02	0.03	0.02	0.03	0.03	0.00	0.05	0.00	0.00	0.00	0.02	0.00
**GH97**	0.00	0.00	2.14	0.05	1.92	2.95	3.09	1.65	1.60	1.43	2.31	0.04	0.02	0.36	0.00	0.08
**GH50**	0.00	0.00	0.00	0.00	0.23	0.03	0.00	0.00	0.00	0.00	0.00	0.00	0.00	0.00	0.00	0.00
**GT26**	0.36	0.31	0.65	0.67	0.60	0.97	0.49	0.32	0.37	0.63	0.44	0.42	0.84	0.28	0.40	0.34
**GH18**	1.62	1.91	0.77	1.69	1.80	1.12	0.69	0.88	0.82	0.85	0.58	1.14	1.41	1.84	2.38	1.11
**GH37**	0.03	0.00	0.02	0.04	0.05	0.03	0.01	0.01	0.03	0.00	0.03	0.31	0.07	0.28	0.05	0.13
**PL13**	0.00	0.00	0.00	0.00	0.02	0.00	0.00	0.00	0.00	0.00	0.00	0.00	0.00	0.00	0.00	0.00
**GH27**	0.00	0.00	0.39	0.03	0.49	0.34	0.12	0.08	0.37	0.21	0.32	0.00	0.00	0.07	0.02	0.00
**GT54**	0.00	0.00	0.00	0.00	0.00	0.00	0.00	0.00	0.00	0.00	0.00	0.00	0.00	0.00	0.00	0.00
**GT91**	0.00	0.00	0.00	0.00	0.00	0.00	0.00	0.00	0.00	0.00	0.00	0.00	0.00	0.00	0.00	0.00
**GT72**	0.00	0.00	0.00	0.00	0.00	0.00	0.00	0.00	0.00	0.00	0.00	0.00	0.00	0.00	0.00	0.00
**GH67**	0.00	0.00	0.42	0.01	0.23	0.45	0.36	0.18	0.29	0.40	0.37	0.00	0.00	0.10	0.00	0.00
**GH12**	0.00	0.00	0.06	0.00	0.02	0.05	0.01	0.01	0.00	0.03	0.03	0.00	0.00	0.00	0.00	0.00
**GH91**	0.00	0.00	0.00	0.00	0.06	0.00	0.01	0.01	0.00	0.00	0.02	0.00	0.00	0.00	0.00	0.00
**GT69**	0.00	0.00	0.00	0.00	0.00	0.00	0.00	0.00	0.00	0.00	0.00	0.00	0.00	0.00	0.00	0.00
**GH106**	0.00	0.00	0.18	0.01	0.35	0.45	0.20	0.07	0.27	0.21	0.63	0.02	0.00	0.18	0.00	0.05
**GT62**	0.00	0.00	0.00	0.00	0.00	0.00	0.00	0.00	0.00	0.00	0.00	0.00	0.00	0.00	0.00	0.00
**GH17**	0.00	0.00	0.00	0.00	0.00	0.00	0.00	0.00	0.00	0.00	0.00	0.00	0.00	0.00	0.00	0.00
**GH88**	0.00	0.00	0.26	0.00	0.63	0.55	0.04	0.07	0.17	0.11	0.17	0.02	0.00	0.07	0.00	0.00
**CE5**	0.00	0.00	0.00	0.00	0.00	0.00	0.00	0.00	0.00	0.00	0.00	0.00	0.00	0.00	0.00	0.00
**GT8**	0.05	0.16	0.27	0.66	0.72	0.30	0.53	0.37	0.24	0.79	1.04	1.32	1.04	0.55	2.05	0.42
**GT50**	0.00	0.00	0.00	0.00	0.00	0.00	0.00	0.00	0.01	0.00	0.00	0.00	0.00	0.00	0.00	0.00
**GT20**	0.00	0.00	0.02	0.00	0.16	0.03	0.02	0.00	0.01	0.00	0.02	0.07	0.07	0.03	0.00	0.00
**PL9**	0.00	0.00	0.27	0.01	0.02	0.10	0.11	0.17	0.10	0.05	0.09	0.11	0.02	0.02	0.00	0.03
**GH55**	0.00	0.00	0.00	0.01	0.02	0.00	0.00	0.00	0.00	0.00	0.00	0.00	0.02	0.00	0.00	0.00
**GH64**	0.00	0.00	0.00	0.00	0.00	0.00	0.00	0.00	0.00	0.00	0.00	0.00	0.00	0.00	0.00	0.00
**GH127**	0.00	0.00	0.86	0.10	0.95	1.26	1.22	0.60	1.28	0.63	1.04	0.20	0.05	0.18	0.02	0.11
**GH103**	0.02	0.00	0.03	0.06	0.04	0.05	0.02	0.00	0.00	0.03	0.02	0.09	0.02	0.03	0.05	0.00
**GH3**	4.60	3.88	10.38	10.35	7.30	5.91	9.44	7.26	7.92	9.68	7.81	4.64	6.15	3.95	1.36	2.47
**GT45**	0.00	0.00	0.00	0.00	0.00	0.00	0.00	0.00	0.00	0.00	0.00	0.00	0.00	0.00	0.00	0.00
**GT74**	0.00	0.00	0.00	0.00	0.00	0.00	0.00	0.00	0.00	0.00	0.00	0.00	0.00	0.00	0.00	0.00
**GT90**	0.00	0.00	0.00	0.00	0.00	0.00	0.00	0.00	0.00	0.00	0.00	0.00	0.00	0.00	0.00	0.00
**GH116**	0.00	0.00	0.00	0.00	0.01	0.00	0.00	0.00	0.01	0.00	0.02	0.00	0.00	0.00	0.00	0.00
**GH31**	1.54	1.46	3.99	2.84	2.64	2.27	3.65	2.61	2.96	4.15	2.74	2.55	2.01	2.02	0.19	2.68
**GT12**	0.00	0.00	0.00	0.00	0.00	0.00	0.00	0.00	0.00	0.00	0.00	0.00	0.00	0.00	0.00	0.00
**CE1**	0.07	0.05	0.87	0.18	0.62	1.16	0.69	0.60	0.41	0.56	0.71	0.66	0.27	0.41	0.42	0.11
**GH121**	0.00	0.00	0.33	0.09	0.12	0.04	0.33	0.31	0.71	0.00	0.00	0.00	0.00	0.00	0.00	0.00
**GH79**	0.00	0.00	0.02	0.00	0.00	0.04	0.02	0.00	0.01	0.03	0.03	0.31	0.00	0.00	0.00	0.00
**GT73**	0.00	0.00	0.00	0.01	0.00	0.03	0.00	0.01	0.00	0.00	0.00	0.00	0.00	0.00	0.00	0.00
**GH77**	4.47	5.19	3.19	3.76	2.41	2.33	2.43	2.95	2.86	3.15	2.50	2.40	3.92	3.67	3.65	4.14
**PL17**	0.00	0.00	0.00	0.00	0.00	0.00	0.00	0.00	0.00	0.00	0.00	0.00	0.00	0.00	0.00	0.00
**PL8**	0.00	0.00	0.02	0.16	0.40	0.07	0.01	0.01	0.00	0.00	0.00	0.00	0.00	0.00	0.00	0.00

CAZY_group	MA-IN-11	MA-IN-12	MA-IN-13	MA-IN-14	MA-IN-15	MA-IN-16	MA-IN-17	MA-IN-18	MA-IN-2	MA-IN-3	MA-IN-4	MA-IN-5	MA-IN-6	MA-IN-7	MA-IN-8	MA-IN-9
**CE9**	0.99	1.48	0.68	1.56	1.07	0.70	0.95	1.14	1.22	0.53	0.80	1.10	1.36	0.96	1.36	0.96
**CE15**	0.00	0.00	0.00	0.00	0.06	0.01	0.00	0.01	0.00	0.00	0.00	0.00	0.00	0.00	0.00	0.00
**GT79**	0.00	0.00	0.00	0.00	0.00	0.00	0.00	0.00	0.00	0.00	0.00	0.00	0.00	0.00	0.00	0.00
**GT10**	0.00	0.00	0.00	0.00	0.07	0.00	0.00	0.00	0.00	0.00	0.00	0.00	0.00	0.00	0.00	0.00
**GH119**	0.00	0.00	0.00	0.00	0.00	0.00	0.00	0.00	0.00	0.00	0.00	0.00	0.00	0.00	0.00	0.00
**GH16**	0.00	0.00	0.39	0.03	0.91	0.78	0.29	0.22	0.33	0.24	0.39	0.02	0.00	0.11	0.05	0.03
**GH43**	1.59	1.93	10.38	10.12	5.74	6.89	9.55	6.92	9.08	8.43	9.68	2.31	2.73	3.07	0.92	0.74
**GH51**	2.28	2.07	3.37	2.26	2.21	2.89	2.45	2.55	2.92	2.25	2.43	0.95	1.71	2.14	1.39	1.75
**GT35**	3.05	2.92	2.26	3.31	2.32	1.98	2.14	2.26	2.51	2.72	2.14	2.90	3.13	2.79	3.72	2.95
**GT25**	0.00	0.00	0.00	0.01	0.00	0.00	0.01	0.00	0.01	0.03	0.00	0.04	0.00	0.00	0.00	0.03
**GT2**	17.73	18.50	16.17	17.72	16.10	15.03	14.67	16.00	14.84	15.78	13.08	14.05	16.89	18.82	18.89	18.39
**GT51**	4.59	4.45	3.02	4.93	3.26	3.49	3.43	4.02	3.54	3.09	2.93	5.45	4.29	4.31	6.28	4.09
**GH107**	0.00	0.00	0.00	0.00	0.00	0.00	0.00	0.00	0.00	0.00	0.00	0.00	0.00	0.00	0.00	0.00
**GH7**	0.00	0.00	0.00	0.00	0.00	0.00	0.00	0.00	0.00	0.00	0.00	0.00	0.00	0.00	0.00	0.00
**GT7**	0.00	0.00	0.00	0.00	0.00	0.00	0.00	0.00	0.00	0.00	0.00	0.00	0.00	0.00	0.00	0.00
**GT36**	0.00	0.00	0.00	0.00	0.00	0.00	0.00	0.00	0.00	0.00	0.00	0.00	0.00	0.00	0.00	0.00
**PL7**	0.00	0.01	0.00	0.01	0.00	0.00	0.00	0.01	0.00	0.00	0.02	0.04	0.07	0.00	0.12	0.00
**GH110**	0.00	0.00	0.30	0.04	0.56	0.31	0.06	0.36	0.38	0.13	0.37	0.00	0.00	0.21	0.00	0.13
**GT21**	0.00	0.00	0.00	0.00	0.00	0.00	0.00	0.00	0.00	0.00	0.00	0.00	0.00	0.00	0.00	0.00
**GH62**	0.00	0.00	0.00	0.00	0.00	0.00	0.00	0.00	0.00	0.00	0.00	0.00	0.00	0.00	0.00	0.00
**GH125**	1.18	1.36	0.44	0.33	0.81	0.62	0.44	0.76	0.46	0.29	0.20	0.84	0.72	0.93	0.73	0.96
**GH117**	0.02	0.00	0.00	0.00	0.22	0.01	0.00	0.00	0.00	0.00	0.00	0.00	0.00	0.00	0.00	0.00
**GH68**	0.10	0.01	0.00	0.06	0.00	0.00	0.02	0.01	0.02	0.03	0.00	0.20	0.35	0.07	0.75	0.03
**GH85**	0.00	0.00	0.26	0.44	0.60	0.04	0.33	0.56	0.41	0.16	0.07	0.72	0.12	0.82	0.40	0.19
**GH92**	0.53	0.43	1.28	0.28	4.14	1.93	0.66	0.59	0.68	1.11	0.66	0.44	0.47	0.46	0.05	0.16
**GH40**	0.00	0.00	0.00	0.00	0.00	0.00	0.00	0.00	0.00	0.00	0.00	0.00	0.00	0.00	0.00	0.00
**GT49**	0.00	0.00	0.00	0.00	0.00	0.00	0.00	0.00	0.00	0.00	0.00	0.00	0.00	0.00	0.00	0.00
**GH112**	2.55	2.26	0.41	0.56	0.89	0.60	0.34	0.97	0.80	0.85	0.51	0.79	1.09	1.53	1.48	2.26
**GT67**	0.00	0.00	0.00	0.00	0.00	0.00	0.00	0.00	0.00	0.00	0.00	0.00	0.00	0.00	0.00	0.00
**CE8**	0.03	0.00	0.51	0.02	0.38	0.63	0.61	0.39	0.21	0.21	0.46	0.15	0.00	0.13	0.05	0.05
**GT86**	0.00	0.00	0.00	0.00	0.00	0.00	0.00	0.00	0.00	0.00	0.00	0.00	0.00	0.00	0.00	0.00
**GH101**	0.00	0.00	0.03	0.11	0.30	0.04	0.14	0.49	0.89	0.24	0.73	0.02	0.02	0.20	0.09	0.27
**GH81**	0.00	0.00	0.00	0.00	0.00	0.00	0.00	0.00	0.00	0.00	0.00	0.00	0.00	0.00	0.00	0.00
**GT93**	0.00	0.00	0.00	0.00	0.00	0.00	0.00	0.00	0.00	0.00	0.00	0.00	0.00	0.00	0.00	0.00
**GT63**	0.00	0.00	0.00	0.00	0.00	0.00	0.00	0.00	0.00	0.00	0.00	0.00	0.00	0.00	0.00	0.00
**CE11**	0.02	0.01	0.47	0.24	0.49	0.64	0.45	0.28	0.46	0.37	0.66	0.40	0.22	0.20	0.19	0.08
**GT19**	0.00	0.00	0.41	0.06	0.49	0.49	0.37	0.25	0.31	0.21	0.58	0.18	0.12	0.16	0.02	0.03
**GT33**	0.00	0.00	0.00	0.00	0.00	0.00	0.00	0.00	0.00	0.00	0.00	0.00	0.00	0.00	0.00	0.00
**GH21**	0.00	0.00	0.00	0.00	0.00	0.00	0.00	0.00	0.00	0.00	0.00	0.00	0.00	0.00	0.00	0.00
**GT68**	0.00	0.00	0.00	0.00	0.00	0.00	0.00	0.00	0.00	0.00	0.00	0.00	0.00	0.00	0.00	0.00
**GH89**	0.00	0.00	0.24	0.01	0.97	0.62	0.22	0.54	0.41	0.61	0.51	0.00	0.00	0.29	0.02	0.27
**GT82**	0.00	0.00	0.00	0.00	0.01	0.00	0.00	0.00	0.00	0.00	0.00	0.00	0.00	0.00	0.00	0.00
**GH30**	0.00	0.00	1.08	0.88	0.73	0.60	0.79	0.52	1.19	0.48	0.75	0.28	0.30	0.21	0.00	0.13
**GH20**	6.19	6.40	3.02	2.06	6.15	2.98	2.21	3.57	2.87	2.41	2.07	1.74	2.43	4.47	3.55	4.14
**GH52**	0.00	0.00	0.00	0.00	0.00	0.00	0.00	0.00	0.00	0.00	0.00	0.00	0.00	0.00	0.00	0.00
**GT84**	0.00	0.00	0.00	0.00	0.00	0.00	0.00	0.03	0.00	0.00	0.00	0.00	0.00	0.00	0.00	0.00
**GT15**	0.00	0.00	0.00	0.00	0.00	0.00	0.00	0.00	0.00	0.00	0.00	0.00	0.00	0.00	0.00	0.00
**GH93**	0.00	0.00	0.00	0.00	0.00	0.01	0.01	0.01	0.00	0.00	0.00	0.02	0.00	0.00	0.00	0.00
**PL16**	0.00	0.00	0.00	0.01	0.00	0.00	0.00	0.00	0.00	0.00	0.00	0.00	0.00	0.00	0.00	0.00
**GH26**	0.00	0.00	0.26	0.09	0.33	0.44	0.26	0.13	0.61	0.24	0.43	0.13	0.20	0.16	0.00	0.00

CAZY_group	MA-IN-11	MA-IN-12	MA-IN-13	MA-IN-14	MA-IN-15	MA-IN-16	MA-IN-17	MA-IN-18	MA-IN-2	MA-IN-3	MA-IN-4	MA-IN-5	MA-IN-6	MA-IN-7	MA-IN-8	MA-IN-9
GT75	0.00	0.00	0.00	0.00	0.00	0.00	0.00	0.00	0.00	0.00	0.00	0.00	0.00	0.00	0.00	0.00
GH53	0.00	0.00	0.44	0.28	0.22	0.36	0.52	0.45	0.37	0.37	0.26	0.29	0.27	0.10	0.09	0.13
GT88	0.00	0.00	0.00	0.00	0.00	0.00	0.00	0.00	0.00	0.00	0.00	0.00	0.00	0.00	0.00	0.00
GT24	0.00	0.00	0.00	0.00	0.00	0.00	0.00	0.00	0.00	0.00	0.00	0.00	0.00	0.00	0.00	0.00
GT81	0.00	0.00	0.00	0.00	0.00	0.00	0.00	0.00	0.00	0.00	0.00	0.00	0.00	0.00	0.00	0.00
GH4	1.01	1.18	0.42	0.29	0.36	0.25	0.11	0.13	0.22	0.16	0.27	1.01	1.12	0.55	0.99	1.22
GT78	0.00	0.00	0.00	0.00	0.00	0.00	0.00	0.00	0.00	0.00	0.00	0.00	0.00	0.00	0.00	0.00
GH8	0.02	0.00	0.35	0.56	0.09	0.16	0.57	0.28	0.31	0.19	0.31	0.20	0.02	0.18	0.31	0.05
GH70	0.25	0.34	0.08	0.58	0.03	0.01	0.20	0.17	0.07	0.13	0.15	1.78	1.41	0.65	5.81	0.29
GH122	0.00	0.00	0.00	0.00	0.00	0.00	0.00	0.00	0.00	0.00	0.00	0.00	0.00	0.00	0.00	0.00
GH74	0.00	0.00	0.00	0.00	0.01	0.00	0.00	0.00	0.00	0.00	0.00	0.00	0.00	0.00	0.00	0.00
GT41	0.03	0.00	0.02	0.01	0.00	0.00	0.00	0.03	0.00	0.00	0.00	0.00	0.00	0.00	0.00	0.00
PL1	0.00	0.00	0.36	0.05	0.41	0.12	0.29	0.22	0.15	0.21	0.61	0.04	0.10	0.21	0.05	0.00
GH15	0.00	0.00	0.00	0.00	0.13	0.01	0.00	0.00	0.00	0.00	0.00	0.00	0.00	0.00	0.00	0.00
GH32	1.66	1.87	2.20	2.34	1.44	1.78	1.65	1.39	1.60	1.82	1.77	2.64	3.05	1.57	2.38	2.63
GH44	0.00	0.00	0.00	0.00	0.00	0.00	0.00	0.00	0.00	0.00	0.00	0.00	0.00	0.00	0.00	0.00
GT6	0.00	0.00	0.00	0.00	0.01	0.01	0.00	0.00	0.00	0.00	0.00	0.00	0.00	0.00	0.00	0.00
GH13	15.69	16.67	13.03	19.10	9.11	9.31	12.76	13.51	13.72	13.24	10.15	14.91	15.53	15.02	15.29	14.59
GT80	0.00	0.00	0.00	0.03	0.01	0.01	0.02	0.02	0.00	0.00	0.03	0.09	0.02	0.03	0.00	0.00
GH71	0.00	0.00	0.00	0.00	0.00	0.00	0.00	0.00	0.00	0.00	0.00	0.00	0.00	0.00	0.00	0.00
GH63	0.00	0.00	0.02	0.02	0.16	0.15	0.29	0.13	0.09	0.05	0.20	0.17	0.02	0.21	0.09	0.05
PL21	0.00	0.00	0.00	0.00	0.00	0.00	0.00	0.00	0.00	0.00	0.00	0.00	0.00	0.00	0.00	0.00
GH65	0.00	0.00	0.11	0.80	0.41	0.21	0.49	0.10	0.17	0.16	0.05	0.13	0.77	0.18	0.21	0.03
CE4	0.03	0.03	0.51	0.23	0.82	0.77	0.44	0.67	0.62	0.66	0.94	0.68	0.22	0.44	0.73	0.32
CE14	0.00	0.00	0.00	0.00	0.05	0.00	0.00	0.02	0.01	0.00	0.00	0.15	0.00	0.00	0.00	0.05
GH115	0.00	0.00	0.33	0.01	0.64	0.38	1.08	0.53	0.51	0.69	1.22	0.00	0.00	0.10	0.00	0.00
GH104	0.00	0.00	0.00	0.00	0.00	0.01	0.00	0.00	0.00	0.00	0.02	0.09	0.00	0.00	0.00	0.00
GH111	0.05	0.03	0.00	0.00	0.02	0.01	0.00	0.00	0.00	0.00	0.00	0.00	0.02	0.02	0.07	0.00
GH95	2.32	2.45	2.66	2.42	2.48	2.58	2.35	2.30	2.72	3.01	2.64	1.36	1.54	2.46	1.46	2.02
GT70	0.00	0.00	0.00	0.00	0.00	0.00	0.00	0.00	0.00	0.00	0.00	0.00	0.00	0.00	0.00	0.00
CE10	0.08	0.35	0.57	0.21	0.42	0.30	0.34	0.22	0.27	0.32	0.29	0.26	0.40	0.16	0.16	0.21
GH10	0.00	0.00	0.60	0.03	0.28	0.77	0.66	0.35	0.44	0.37	0.53	0.00	0.00	0.13	0.00	0.00
GH130	0.00	0.00	0.21	0.00	0.68	0.31	0.29	0.19	0.20	0.26	0.36	0.00	0.00	0.02	0.00	0.00
PL20	0.00	0.00	0.00	0.00	0.00	0.00	0.00	0.00	0.00	0.00	0.00	0.00	0.00	0.00	0.00	0.00
GH69	0.00	0.00	0.00	0.00	0.00	0.00	0.00	0.00	0.00	0.00	0.00	0.00	0.00	0.00	0.00	0.00
PL2	0.00	0.00	0.00	0.00	0.00	0.00	0.00	0.00	0.00	0.00	0.00	0.00	0.00	0.00	0.00	0.00
GH49	0.00	0.00	0.00	0.00	0.00	0.01	0.00	0.00	0.00	0.00	0.00	0.00	0.00	0.00	0.00	0.00
GH25	0.58	0.58	2.14	3.55	1.38	1.60	2.97	2.52	2.74	2.30	1.82	3.74	3.42	1.16	2.02	1.72
GH59	0.00	0.00	0.00	0.01	0.00	0.00	0.00	0.00	0.00	0.05	0.05	0.00	0.00	0.08	0.00	0.00
GH35	0.40	0.37	1.40	1.15	1.64	1.16	0.55	0.43	0.98	0.71	1.04	0.59	0.25	0.55	0.21	0.56
GT39	0.00	0.00	0.00	0.00	0.00	0.00	0.00	0.00	0.00	0.00	0.00	0.28	0.32	0.00	0.00	0.00
GT57	0.00	0.00	0.00	0.00	0.00	0.00	0.00	0.00	0.00	0.00	0.00	0.00	0.00	0.00	0.00	0.00
GH129	1.59	1.41	0.20	0.27	0.70	0.34	0.29	0.82	0.47	0.16	0.26	0.39	0.67	1.35	0.78	1.35
GT22	0.00	0.00	0.00	0.00	0.00	0.00	0.00	0.00	0.00	0.00	0.00	0.00	0.00	0.00	0.00	0.00
PL12	0.00	0.00	0.00	0.00	0.15	0.00	0.02	0.00	0.01	0.00	0.00	0.00	0.00	0.00	0.00	0.03
PL18	0.00	0.00	0.00	0.00	0.00	0.00	0.00	0.00	0.00	0.00	0.00	0.00	0.00	0.00	0.00	0.00
GT92	0.00	0.00	0.00	0.00	0.00	0.00	0.00	0.00	0.00	0.00	0.00	0.00	0.00	0.00	0.00	0.00
GT44	0.00	0.00	0.02	0.00	0.04	0.11	0.04	0.00	0.05	0.00	0.00	0.02	0.00	0.00	0.09	0.05
GT29	0.00	0.00	0.00	0.01	0.00	0.00	0.00	0.00	0.00	0.00	0.00	0.00	0.00	0.00	0.00	0.00
GT13	0.00	0.00	0.00	0.00	0.00	0.00	0.00	0.00	0.00	0.00	0.00	0.00	0.00	0.00	0.00	0.00

Normalized_abundance_2

CAZY_group	MA-IN-11	MA-IN-12	MA-IN-13	MA-IN-14	MA-IN-15	MA-IN-16	MA-IN-17	MA-IN-18	MA-IN-2	MA-IN-3	MA-IN-4	MA-IN-5	MA-IN-6	MA-IN-7	MA-IN-8	MA-IN-9
**GT52**	0.00	0.00	0.00	0.00	0.00	0.00	0.00	0.00	0.00	0.00	0.00	0.00	0.00	0.00	0.00	0.00
**GT64**	0.00	0.00	0.00	0.00	0.00	0.00	0.00	0.00	0.01	0.00	0.00	0.00	0.00	0.00	0.00	0.00
**GT85**	0.00	0.00	0.00	0.00	0.00	0.00	0.00	0.00	0.00	0.00	0.00	0.00	0.00	0.00	0.00	0.00
**GT16**	0.00	0.00	0.00	0.00	0.00	0.00	0.00	0.00	0.00	0.00	0.00	0.00	0.00	0.00	0.00	0.00
**GH56**	0.00	0.00	0.00	0.00	0.00	0.00	0.00	0.00	0.00	0.00	0.00	0.00	0.00	0.00	0.00	0.00
**GT60**	0.00	0.00	0.00	0.00	0.00	0.00	0.00	0.00	0.00	0.00	0.00	0.00	0.00	0.00	0.00	0.00
**GH41**	0.00	0.00	0.00	0.00	0.00	0.00	0.00	0.00	0.00	0.00	0.00	0.00	0.00	0.00	0.00	0.00
**CE7**	0.00	0.00	0.41	0.01	0.37	0.25	0.15	0.07	0.33	0.50	0.39	0.28	0.07	0.03	0.00	0.00
**GH39**	0.00	0.00	0.02	0.00	0.04	0.00	0.14	0.17	0.00	0.03	0.05	0.02	0.00	0.00	0.00	0.00
**GT30**	0.10	0.14	0.92	0.50	0.74	1.04	0.80	0.57	0.56	0.79	0.80	0.39	0.37	0.83	0.38	0.82
**PL15**	0.00	0.00	0.00	0.00	0.24	0.03	0.00	0.00	0.02	0.00	0.00	0.00	0.00	0.00	0.00	0.00
**GH102**	0.00	0.00	0.00	0.03	0.05	0.04	0.04	0.01	0.06	0.03	0.00	0.28	0.00	0.07	0.05	0.03

CAZY_group	CN-AD-71	CN-AD-72	CN-AD-73	CN-AD-74	CN-AD-75	CN-AD-76	CN-AD-77	CN-AD-78	CN-AD-79	CN-AD-80	CN-AD-81	CN-AD-82	CN-AD-83	CN-AD-84	CN-AD-85	CN-AD-86
GH29	0.89	0.67	0.29	0.93	0.58	0.47	0.93	1.07	0.58	0.35	0.37	0.92	0.50	0.52	0.50	0.77
PL4	0.00	0.00	0.00	0.00	0.00	0.00	0.00	0.00	0.00	0.00	0.00	0.00	0.00	0.00	0.00	0.00
GH23	1.22	1.01	0.65	0.96	0.83	0.91	1.10	0.97	0.78	0.71	0.72	0.99	0.91	1.09	0.98	1.13
CE6	0.00	0.09	0.00	0.00	0.00	0.03	0.03	0.00	0.04	0.00	0.02	0.05	0.05	0.00	0.03	0.00
GH72	0.07	0.04	0.10	0.12	0.13	0.02	0.12	0.08	0.06	0.03	0.09	0.02	0.03	0.05	0.08	0.15
GH114	0.00	0.00	0.00	0.00	0.00	0.00	0.00	0.00	0.00	0.00	0.00	0.00	0.00	0.00	0.00	0.00
GH78	1.24	1.08	0.40	0.32	0.43	0.87	1.24	1.07	0.35	0.24	0.48	1.27	0.93	0.34	0.35	1.02
PL19	0.00	0.00	0.00	0.00	0.00	0.00	0.00	0.00	0.00	0.00	0.00	0.00	0.00	0.00	0.00	0.00
GT27	0.00	0.00	0.00	0.00	0.00	0.00	0.00	0.00	0.00	0.00	0.00	0.00	0.00	0.00	0.00	0.00
GH5	0.24	0.38	0.73	0.35	0.77	0.03	0.45	0.10	0.89	0.62	0.44	0.30	0.21	0.36	0.20	0.07
GT40	0.00	0.00	0.00	0.00	0.00	0.00	0.00	0.00	0.00	0.00	0.00	0.00	0.00	0.00	0.00	0.00
CE13	0.00	0.00	0.00	0.00	0.00	0.00	0.00	0.00	0.00	0.00	0.00	0.00	0.00	0.00	0.00	0.00
GT37	0.00	0.00	0.00	0.00	0.00	0.00	0.00	0.00	0.00	0.00	0.00	0.00	0.00	0.00	0.00	0.00
GH33	0.26	0.22	0.10	0.18	0.09	0.14	0.34	0.42	0.16	0.10	0.16	0.37	0.22	0.13	0.20	0.40
GT65	0.00	0.00	0.00	0.00	0.00	0.00	0.00	0.00	0.00	0.00	0.00	0.00	0.00	0.00	0.00	0.00
GH86	0.00	0.00	0.00	0.00	0.00	0.00	0.00	0.00	0.02	0.03	0.00	0.02	0.00	0.00	0.00	0.00
GH123	0.11	0.13	0.05	0.09	0.06	0.14	0.14	0.21	0.06	0.04	0.10	0.27	0.12	0.05	0.05	0.15
GH96	0.00	0.00	0.00	0.00	0.00	0.00	0.00	0.00	0.00	0.00	0.00	0.00	0.00	0.00	0.00	0.00
GH14	0.00	0.00	0.00	0.00	0.00	0.00	0.00	0.00	0.00	0.00	0.00	0.00	0.00	0.00	0.00	0.00
CE3	0.00	0.00	0.00	0.00	0.00	0.00	0.00	0.00	0.00	0.00	0.00	0.00	0.00	0.00	0.00	0.00
PL10	0.22	0.13	0.10	0.32	0.10	0.09	0.19	0.13	0.14	0.03	0.05	0.15	0.15	0.10	0.08	0.07
GT48	0.00	0.00	0.00	0.00	0.00	0.00	0.00	0.00	0.00	0.00	0.00	0.00	0.00	0.00	0.00	0.00
PL6	0.00	0.00	0.00	0.00	0.00	0.00	0.02	0.00	0.00	0.00	0.00	0.00	0.00	0.00	0.00	0.00
GT83	0.13	0.09	0.02	0.12	0.07	0.11	0.09	0.16	0.02	0.01	0.05	0.20	0.09	0.10	0.05	0.26
GH126	0.00	0.00	0.00	0.00	0.00	0.00	0.00	0.00	0.00	0.00	0.00	0.00	0.00	0.00	0.00	0.00
GH9	0.17	0.07	0.18	0.50	0.26	0.08	0.12	0.13	0.41	0.27	0.16	0.20	0.09	0.36	0.18	0.11
GH75	0.00	0.00	0.00	0.00	0.00	0.00	0.00	0.00	0.00	0.00	0.00	0.00	0.00	0.00	0.00	0.00
GT71	0.00	0.00	0.00	0.00	0.00	0.00	0.00	0.00	0.00	0.00	0.00	0.00	0.00	0.00	0.00	0.00
GH46	0.00	0.00	0.00	0.00	0.00	0.00	0.00	0.00	0.00	0.00	0.00	0.00	0.00	0.00	0.00	0.00
GT61	0.00	0.00	0.00	0.00	0.00	0.00	0.00	0.00	0.00	0.00	0.00	0.00	0.00	0.00	0.00	0.00
GT3	0.22	0.07	0.03	0.09	0.08	0.11	0.16	0.08	0.02	0.03	0.06	0.12	0.09	0.03	0.03	0.18
PL3	0.00	0.00	0.00	0.00	0.00	0.00	0.00	0.00	0.00	0.00	0.00	0.00	0.00	0.00	0.00	0.00
GH108	0.04	0.09	0.03	0.00	0.03	0.05	0.03	0.05	0.02	0.03	0.02	0.02	0.03	0.08	0.05	0.00
GT56	0.00	0.00	0.00	0.00	0.00	0.03	0.00	0.00	0.00	0.00	0.00	0.00	0.03	0.05	0.03	0.04
PL11	0.26	0.31	0.16	0.12	0.14	0.16	0.20	0.18	0.06	0.11	0.09	0.17	0.10	0.10	0.13	0.04
GH45	0.00	0.00	0.00	0.00	0.00	0.00	0.00	0.00	0.02	0.00	0.00	0.00	0.00	0.00	0.03	0.00
GT34	0.00	0.00	0.00	0.00	0.01	0.00	0.00	0.00	0.00	0.00	0.00	0.00	0.00	0.00	0.00	0.00
PL14	0.00	0.00	0.00	0.00	0.00	0.00	0.00	0.00	0.00	0.00	0.00	0.00	0.00	0.00	0.00	0.00
GH19	0.00	0.00	0.00	0.00	0.01	0.00	0.00	0.03	0.02	0.00	0.00	0.00	0.00	0.00	0.00	0.00
GH118	0.00	0.00	0.00	0.00	0.00	0.00	0.00	0.00	0.00	0.00	0.00	0.00	0.00	0.00	0.00	0.00
GH82	0.00	0.00	0.00	0.00	0.00	0.00	0.00	0.00	0.00	0.00	0.00	0.00	0.00	0.00	0.00	0.00
GH76	0.35	0.38	0.02	0.03	0.10	0.16	0.28	0.13	0.06	0.07	0.15	0.37	0.26	0.03	0.03	0.22
GT4	1.92	2.15	1.53	2.34	1.59	1.19	2.24	2.17	1.61	1.07	1.39	2.71	1.38	1.43	1.75	2.75
GT87	0.00	0.00	0.00	0.00	0.01	0.00	0.00	0.00	0.00	0.00	0.00	0.00	0.00	0.00	0.00	0.04
GH24	0.20	0.18	0.16	0.18	0.14	0.39	0.14	0.16	0.16	0.23	0.15	0.20	0.14	0.18	0.40	0.33
GH90	0.00	0.00	0.00	0.00	0.00	0.00	0.00	0.00	0.00	0.00	0.00	0.00	0.00	0.00	0.00	0.00
GH57	0.15	0.11	0.05	0.18	0.04	0.09	0.12	0.13	0.12	0.07	0.10	0.10	0.09	0.05	0.10	0.15
GH36	0.76	1.01	0.63	0.38	0.52	0.37	0.87	0.97	0.54	0.48	0.64	0.60	0.62	0.47	0.70	0.51
GT46	0.00	0.00	0.00	0.00	0.00	0.00	0.00	0.00	0.00	0.00	0.00	0.00	0.00	0.00	0.00	0.00
GH66	0.04	0.07	0.02	0.00	0.03	0.06	0.03	0.00	0.00	0.01	0.01	0.00	0.03	0.03	0.03	0.00

CAZY_group	CN-AD-71	CN-AD-72	CN-AD-73	CN-AD-74	CN-AD-75	CN-AD-76	CN-AD-77	CN-AD-78	CN-AD-79	CN-AD-80	CN-AD-81	CN-AD-82	CN-AD-83	CN-AD-84	CN-AD-85	CN-AD-86
GH48	0.00	0.00	0.00	0.00	0.00	0.00	0.00	0.00	0.00	0.00	0.00	0.00	0.00	0.00	0.00	0.00
GT53	0.00	0.00	0.00	0.00	0.00	0.00	0.00	0.00	0.00	0.00	0.00	0.00	0.00	0.00	0.00	0.00
GH60	0.00	0.00	0.00	0.00	0.00	0.00	0.00	0.00	0.00	0.00	0.00	0.00	0.00	0.00	0.00	0.00
GT31	0.00	0.00	0.00	0.00	0.00	0.02	0.00	0.00	0.00	0.00	0.00	0.00	0.00	0.03	0.03	0.04
GH105	1.07	0.63	0.39	0.64	0.43	0.19	0.98	1.00	0.35	0.23	0.53	0.67	0.55	0.52	0.55	0.26
GT23	0.02	0.02	0.00	0.06	0.00	0.00	0.00	0.00	0.00	0.00	0.01	0.00	0.00	0.00	0.03	0.00
GH11	0.00	0.00	0.02	0.00	0.01	0.00	0.00	0.00	0.00	0.00	0.00	0.00	0.00	0.00	0.00	0.00
GT38	0.00	0.00	0.00	0.00	0.00	0.00	0.00	0.00	0.00	0.00	0.00	0.00	0.00	0.00	0.00	0.00
GT43	0.00	0.00	0.00	0.00	0.00	0.00	0.00	0.00	0.00	0.00	0.00	0.00	0.00	0.00	0.00	0.00
GH99	0.02	0.04	0.00	0.00	0.00	0.00	0.02	0.00	0.00	0.00	0.04	0.00	0.03	0.00	0.00	0.00
GH38	0.35	0.20	0.06	0.06	0.16	0.11	0.19	0.10	0.14	0.16	0.24	0.22	0.21	0.31	0.25	0.44
GH109	0.33	0.34	0.29	0.35	0.16	0.20	0.37	0.55	0.23	0.10	0.09	0.30	0.10	0.23	0.18	0.26
GH2	5.29	4.73	2.97	3.94	2.99	2.67	4.84	4.79	2.35	2.38	3.16	4.40	4.15	2.34	2.68	3.81
GT28	0.35	0.25	0.31	0.38	0.31	0.23	0.25	0.21	0.35	0.27	0.34	0.25	0.29	0.23	0.48	0.22
GH42	0.15	0.13	0.16	0.20	0.14	0.09	0.11	0.08	0.14	0.06	0.15	0.15	0.12	0.03	0.10	0.00
GT58	0.00	0.00	0.00	0.00	0.00	0.00	0.00	0.00	0.00	0.00	0.00	0.00	0.00	0.00	0.00	0.00
GH61	0.00	0.00	0.00	0.00	0.00	0.00	0.00	0.00	0.00	0.00	0.00	0.00	0.00	0.00	0.00	0.00
GH80	0.00	0.00	0.00	0.00	0.00	0.00	0.00	0.00	0.00	0.00	0.00	0.00	0.00	0.00	0.00	0.00
GH98	0.00	0.02	0.00	0.00	0.00	0.00	0.00	0.00	0.00	0.01	0.00	0.00	0.00	0.03	0.00	0.00
GH6	0.00	0.00	0.00	0.00	0.00	0.00	0.00	0.00	0.00	0.00	0.00	0.00	0.00	0.00	0.00	0.00
GT1	0.02	0.04	0.03	0.03	0.03	0.05	0.03	0.00	0.02	0.03	0.01	0.05	0.02	0.00	0.00	0.07
GH28	1.35	1.50	0.86	1.05	0.73	0.45	1.38	1.07	0.66	0.44	0.65	0.87	0.93	0.62	0.53	0.37
GT42	0.00	0.00	0.00	0.00	0.00	0.00	0.00	0.00	0.00	0.00	0.00	0.00	0.00	0.00	0.00	0.00
GH128	0.00	0.00	0.00	0.00	0.00	0.00	0.00	0.00	0.00	0.00	0.00	0.00	0.00	0.00	0.00	0.00
CE12	0.26	0.22	0.29	0.12	0.10	0.09	0.22	0.13	0.04	0.04	0.21	0.07	0.10	0.13	0.05	0.04
CE16	0.00	0.00	0.00	0.00	0.00	0.00	0.00	0.00	0.00	0.00	0.00	0.00	0.00	0.00	0.00	0.00
GT89	0.00	0.00	0.00	0.00	0.00	0.00	0.00	0.00	0.00	0.00	0.00	0.00	0.00	0.00	0.00	0.00
GT11	0.09	0.07	0.08	0.12	0.04	0.05	0.06	0.05	0.06	0.01	0.04	0.10	0.05	0.05	0.10	0.04
GT77	0.00	0.02	0.00	0.00	0.00	0.00	0.02	0.00	0.00	0.01	0.00	0.02	0.00	0.00	0.05	0.04
GT55	0.00	0.00	0.00	0.00	0.00	0.00	0.00	0.00	0.00	0.00	0.00	0.00	0.00	0.00	0.00	0.00
GT18	0.00	0.00	0.00	0.00	0.00	0.00	0.00	0.00	0.00	0.00	0.00	0.00	0.00	0.00	0.00	0.00
GH87	0.00	0.00	0.00	0.00	0.03	0.00	0.00	0.00	0.00	0.00	0.01	0.00	0.00	0.00	0.00	0.00
PL5	0.00	0.00	0.00	0.00	0.00	0.00	0.00	0.00	0.00	0.00	0.00	0.00	0.00	0.00	0.00	0.00
GH22	0.00	0.00	0.00	0.00	0.00	0.00	0.00	0.00	0.00	0.00	0.00	0.00	0.00	0.00	0.00	0.00
CE2	0.04	0.07	0.03	0.00	0.08	0.00	0.12	0.03	0.04	0.06	0.02	0.02	0.03	0.00	0.00	0.04
GH120	0.07	0.07	0.06	0.00	0.07	0.03	0.03	0.00	0.00	0.01	0.02	0.02	0.07	0.00	0.05	0.04
GH73	0.33	0.34	0.34	0.26	0.39	0.22	0.26	0.45	0.29	0.35	0.32	0.30	0.31	0.29	0.45	0.33
GT17	0.00	0.00	0.00	0.00	0.01	0.00	0.00	0.00	0.00	0.00	0.00	0.00	0.00	0.00	0.00	0.00
GH124	0.00	0.00	0.00	0.00	0.00	0.00	0.00	0.00	0.00	0.00	0.00	0.00	0.00	0.00	0.00	0.00
GH54	0.00	0.00	0.00	0.00	0.00	0.00	0.00	0.00	0.00	0.00	0.00	0.00	0.00	0.00	0.00	0.00
GH100	0.00	0.00	0.00	0.00	0.00	0.00	0.00	0.00	0.00	0.00	0.00	0.00	0.00	0.00	0.00	0.00
GH113	0.02	0.00	0.03	0.00	0.00	0.00	0.03	0.00	0.02	0.03	0.04	0.00	0.02	0.03	0.00	0.00
GT5	0.37	0.27	0.35	0.41	0.43	0.16	0.23	0.26	0.33	0.42	0.35	0.17	0.22	0.36	0.25	0.15
GH94	0.37	0.22	0.61	0.47	0.77	0.06	0.16	0.10	0.39	0.32	0.53	0.02	0.22	0.39	0.08	0.00
GT66	0.00	0.00	0.00	0.00	0.00	0.00	0.00	0.00	0.00	0.00	0.00	0.00	0.00	0.00	0.00	0.00
GT94	0.00	0.00	0.00	0.00	0.00	0.00	0.00	0.00	0.00	0.00	0.00	0.00	0.00	0.00	0.00	0.00
GH58	0.00	0.00	0.00	0.00	0.00	0.00	0.00	0.00	0.00	0.00	0.00	0.00	0.00	0.00	0.00	0.04
GH1	0.52	0.22	0.29	0.20	0.49	0.09	0.33	0.13	0.19	0.27	0.34	0.12	0.12	0.26	0.30	0.18
PL22	0.00	0.00	0.00	0.00	0.00	0.00	0.00	0.00	0.00	0.00	0.00	0.00	0.00	0.00	0.00	0.00
GT9	0.17	0.09	0.05	0.23	0.07	0.12	0.08	0.13	0.08	0.04	0.15	0.22	0.17	0.13	0.23	0.07

CAZY_group	CN-AD-71	CN-AD-72	CN-AD-73	CN-AD-74	CN-AD-75	CN-AD-76	CN-AD-77	CN-AD-78	CN-AD-79	CN-AD-80	CN-AD-81	CN-AD-82	CN-AD-83	CN-AD-84	CN-AD-85	CN-AD-86
GT76	0.00	0.00	0.00	0.00	0.00	0.00	0.00	0.00	0.00	0.00	0.00	0.00	0.00	0.00	0.00	0.00
GT32	0.15	0.07	0.03	0.09	0.08	0.08	0.09	0.05	0.12	0.04	0.06	0.17	0.05	0.08	0.00	0.15
GT47	0.00	0.02	0.05	0.06	0.02	0.02	0.05	0.00	0.00	0.00	0.04	0.02	0.00	0.00	0.05	0.04
GH84	0.11	0.09	0.10	0.20	0.08	0.09	0.11	0.13	0.10	0.06	0.06	0.12	0.09	0.08	0.25	0.15
GH83	0.00	0.00	0.00	0.00	0.00	0.00	0.00	0.00	0.00	0.00	0.00	0.00	0.00	0.00	0.00	0.00
GT59	0.00	0.00	0.00	0.00	0.00	0.00	0.00	0.00	0.00	0.00	0.00	0.00	0.00	0.00	0.00	0.00
GH34	0.00	0.00	0.00	0.00	0.00	0.00	0.00	0.00	0.00	0.00	0.00	0.00	0.00	0.00	0.00	0.00
GT14	0.07	0.09	0.02	0.00	0.00	0.00	0.02	0.05	0.02	0.03	0.05	0.05	0.02	0.05	0.03	0.04
GH47	0.00	0.00	0.00	0.00	0.00	0.00	0.00	0.00	0.00	0.00	0.00	0.00	0.00	0.00	0.00	0.00
GH97	1.09	1.05	0.42	1.20	0.48	0.53	1.09	0.97	0.49	0.31	0.48	1.04	0.77	0.68	0.80	0.95
GH50	0.02	0.11	0.00	0.00	0.02	0.02	0.06	0.00	0.04	0.00	0.10	0.05	0.05	0.00	0.00	0.07
GT26	0.13	0.20	0.19	0.12	0.17	0.14	0.19	0.24	0.10	0.10	0.18	0.25	0.21	0.18	0.15	0.11
GH18	0.52	0.63	0.32	0.35	0.28	0.41	0.68	0.37	0.45	0.34	0.61	0.62	0.64	0.39	0.35	0.44
GH37	0.00	0.00	0.00	0.00	0.00	0.06	0.00	0.00	0.00	0.00	0.02	0.02	0.00	0.05	0.05	0.11
PL13	0.13	0.04	0.00	0.06	0.01	0.00	0.08	0.05	0.04	0.00	0.02	0.07	0.07	0.03	0.03	0.04
GH27	0.26	0.22	0.23	0.15	0.18	0.11	0.20	0.18	0.19	0.07	0.28	0.42	0.21	0.10	0.05	0.15
GT54	0.00	0.00	0.00	0.00	0.00	0.00	0.00	0.00	0.00	0.00	0.00	0.00	0.00	0.00	0.00	0.00
GT91	0.00	0.00	0.00	0.00	0.00	0.00	0.00	0.00	0.00	0.00	0.00	0.00	0.00	0.00	0.00	0.00
GT72	0.00	0.00	0.00	0.00	0.00	0.00	0.00	0.00	0.00	0.00	0.00	0.00	0.00	0.00	0.00	0.00
GH67	0.15	0.09	0.05	0.18	0.08	0.03	0.06	0.13	0.06	0.01	0.07	0.15	0.05	0.05	0.08	0.00
GH12	0.00	0.00	0.00	0.00	0.00	0.00	0.00	0.00	0.00	0.00	0.00	0.00	0.00	0.00	0.00	0.00
GH91	0.00	0.00	0.00	0.00	0.00	0.02	0.03	0.05	0.02	0.01	0.00	0.02	0.03	0.00	0.03	0.00
GT69	0.00	0.00	0.00	0.00	0.00	0.00	0.00	0.00	0.00	0.00	0.00	0.00	0.00	0.00	0.00	0.00
GH106	0.57	0.27	0.19	0.26	0.13	0.17	0.42	0.37	0.10	0.07	0.11	0.30	0.29	0.16	0.15	0.22
GT62	0.00	0.00	0.00	0.00	0.00	0.00	0.00	0.00	0.00	0.00	0.00	0.00	0.00	0.00	0.00	0.00
GH17	0.00	0.00	0.00	0.00	0.00	0.00	0.00	0.00	0.02	0.00	0.00	0.00	0.00	0.00	0.00	0.00
GH88	0.37	0.63	0.16	0.44	0.30	0.19	0.62	0.42	0.19	0.16	0.22	0.47	0.36	0.10	0.20	0.18
CE5	0.00	0.00	0.00	0.00	0.00	0.00	0.00	0.00	0.00	0.00	0.00	0.00	0.00	0.00	0.00	0.00
GT8	0.04	0.13	0.05	0.20	0.09	0.06	0.17	0.13	0.16	0.01	0.09	0.15	0.09	0.10	0.15	0.11
GT50	0.00	0.00	0.00	0.00	0.00	0.00	0.00	0.00	0.00	0.00	0.00	0.00	0.00	0.00	0.00	0.00
GT20	0.11	0.09	0.03	0.09	0.03	0.16	0.06	0.16	0.04	0.04	0.04	0.12	0.12	0.10	0.10	0.26
PL9	0.20	0.22	0.18	0.09	0.03	0.02	0.22	0.03	0.04	0.14	0.13	0.05	0.07	0.26	0.08	0.00
GH55	0.02	0.04	0.03	0.03	0.04	0.00	0.05	0.08	0.08	0.03	0.02	0.02	0.02	0.03	0.03	0.04
GH64	0.00	0.00	0.00	0.03	0.00	0.00	0.00	0.00	0.00	0.00	0.00	0.00	0.00	0.00	0.00	0.00
GH127	0.39	0.27	0.19	0.20	0.31	0.27	0.29	0.50	0.16	0.08	0.21	0.30	0.36	0.16	0.25	0.48
GH103	0.00	0.00	0.00	0.00	0.00	0.03	0.00	0.00	0.00	0.00	0.00	0.00	0.00	0.05	0.05	0.04
GH3	3.51	2.35	2.00	1.96	2.65	1.56	2.89	2.91	1.81	1.82	1.98	2.79	2.32	1.71	1.68	2.27
GT45	0.00	0.00	0.00	0.00	0.00	0.00	0.00	0.00	0.00	0.00	0.00	0.00	0.00	0.00	0.03	0.00
GT74	0.00	0.00	0.00	0.00	0.00	0.00	0.00	0.00	0.00	0.00	0.00	0.00	0.00	0.00	0.00	0.00
GT90	0.00	0.00	0.00	0.00	0.00	0.00	0.00	0.00	0.00	0.00	0.00	0.00	0.00	0.00	0.00	0.00
GH116	0.02	0.09	0.08	0.09	0.04	0.05	0.05	0.08	0.12	0.08	0.05	0.10	0.00	0.08	0.05	0.07
GH31	1.20	1.32	1.00	0.88	0.88	0.72	1.15	1.20	0.66	0.52	0.87	1.24	0.95	0.65	0.73	0.77
GT12	0.00	0.00	0.00	0.00	0.00	0.00	0.00	0.00	0.00	0.00	0.00	0.00	0.00	0.00	0.00	0.00
CE1	0.46	0.29	0.34	0.56	0.31	0.41	0.39	0.42	0.37	0.31	0.27	0.32	0.31	0.29	0.35	0.48
GH121	0.00	0.00	0.00	0.00	0.00	0.00	0.00	0.00	0.00	0.00	0.00	0.10	0.00	0.00	0.00	0.00
GH79	0.00	0.00	0.00	0.00	0.01	0.00	0.00	0.00	0.00	0.00	0.01	0.00	0.00	0.00	0.00	0.00
GT73	0.00	0.00	0.00	0.00	0.00	0.00	0.00	0.00	0.00	0.00	0.00	0.00	0.00	0.00	0.00	0.00
GH77	0.54	0.47	0.60	0.61	0.51	0.27	0.57	0.47	0.41	0.44	0.43	0.32	0.38	0.39	0.43	0.40
PL17	0.00	0.00	0.05	0.03	0.02	0.02	0.05	0.00	0.00	0.01	0.02	0.07	0.00	0.00	0.00	0.00
PL8	0.33	0.38	0.08	0.53	0.18	0.23	0.37	0.45	0.19	0.16	0.21	0.35	0.34	0.26	0.23	0.26

CAZY_group	CN-AD-71	CN-AD-72	CN-AD-73	CN-AD-74	CN-AD-75	CN-AD-76	CN-AD-77	CN-AD-78	CN-AD-79	CN-AD-80	CN-AD-81	CN-AD-82	CN-AD-83	CN-AD-84	CN-AD-85	CN-AD-86
**CE9**	0.46	0.38	0.32	0.29	0.32	0.28	0.43	0.37	0.16	0.32	0.29	0.30	0.29	0.26	0.35	0.40
**CE15**	0.00	0.02	0.02	0.00	0.00	0.02	0.05	0.03	0.00	0.00	0.00	0.00	0.02	0.00	0.00	0.00
**GT79**	0.00	0.00	0.00	0.00	0.00	0.00	0.00	0.00	0.00	0.00	0.00	0.00	0.00	0.00	0.00	0.00
**GT10**	0.00	0.00	0.00	0.00	0.00	0.00	0.02	0.00	0.00	0.01	0.00	0.00	0.00	0.00	0.00	0.07
**GH119**	0.00	0.00	0.00	0.00	0.00	0.00	0.00	0.00	0.00	0.00	0.00	0.00	0.00	0.00	0.00	0.00
**GH16**	0.30	0.29	0.13	0.23	0.16	0.16	0.31	0.50	0.16	0.14	0.12	0.45	0.21	0.16	0.08	0.22
**GH43**	2.81	2.69	1.66	2.92	1.73	1.34	2.93	2.85	1.90	1.13	2.06	2.69	2.43	1.14	1.55	1.43
**GH51**	0.57	0.56	0.40	0.58	0.40	0.47	0.65	0.52	0.49	0.31	0.39	0.62	0.50	0.23	0.18	0.55
**GT35**	0.74	0.83	1.02	0.73	0.94	0.33	0.76	0.47	0.82	0.73	0.77	0.55	0.59	0.78	0.63	0.29
**GT25**	0.00	0.00	0.00	0.00	0.00	0.00	0.00	0.00	0.00	0.00	0.00	0.05	0.00	0.00	0.00	0.00
**GT2**	4.48	3.52	3.90	4.65	3.71	2.58	4.73	3.88	3.15	2.72	3.07	4.70	2.84	2.96	4.01	5.53
**GT51**	0.81	0.58	0.74	0.67	0.73	0.62	0.96	0.84	0.49	0.54	0.71	0.92	0.88	0.60	1.15	1.02
**GH107**	0.00	0.00	0.00	0.00	0.00	0.00	0.00	0.00	0.00	0.00	0.00	0.00	0.00	0.00	0.00	0.00
**GH7**	0.00	0.00	0.00	0.00	0.00	0.00	0.00	0.00	0.00	0.00	0.00	0.00	0.00	0.00	0.00	0.00
**GT7**	0.00	0.00	0.00	0.00	0.00	0.00	0.00	0.00	0.00	0.00	0.00	0.00	0.00	0.00	0.00	0.00
**GT36**	0.00	0.00	0.00	0.00	0.00	0.00	0.00	0.00	0.00	0.00	0.00	0.00	0.00	0.00	0.00	0.00
**PL7**	0.00	0.00	0.00	0.00	0.00	0.00	0.00	0.00	0.00	0.00	0.00	0.00	0.00	0.00	0.00	0.00
**GH110**	0.24	0.09	0.10	0.12	0.07	0.05	0.12	0.26	0.06	0.03	0.06	0.27	0.15	0.08	0.18	0.18
**GT21**	0.00	0.00	0.00	0.00	0.00	0.00	0.00	0.00	0.00	0.00	0.00	0.00	0.00	0.00	0.00	0.00
**GH62**	0.00	0.00	0.00	0.00	0.00	0.00	0.00	0.00	0.00	0.00	0.00	0.00	0.00	0.00	0.00	0.00
**GH125**	0.15	0.20	0.06	0.23	0.09	0.14	0.11	0.08	0.08	0.08	0.15	0.25	0.12	0.05	0.23	0.11
**GH117**	0.09	0.07	0.06	0.03	0.08	0.11	0.17	0.18	0.10	0.06	0.06	0.10	0.05	0.05	0.05	0.11
**GH68**	0.00	0.00	0.00	0.00	0.00	0.00	0.00	0.00	0.00	0.00	0.00	0.00	0.00	0.00	0.00	0.00
**GH85**	0.04	0.00	0.02	0.06	0.00	0.00	0.00	0.00	0.00	0.00	0.02	0.00	0.00	0.00	0.05	0.00
**GH92**	1.92	1.61	0.65	1.99	0.79	1.27	2.10	1.86	0.82	0.71	1.06	2.26	1.67	0.91	0.93	2.05
**GH40**	0.00	0.00	0.00	0.00	0.00	0.00	0.00	0.00	0.00	0.00	0.00	0.00	0.00	0.00	0.00	0.00
**GT49**	0.00	0.00	0.00	0.00	0.00	0.00	0.00	0.00	0.00	0.00	0.00	0.00	0.00	0.00	0.00	0.00
**GH112**	0.22	0.11	0.15	0.03	0.17	0.06	0.20	0.13	0.10	0.21	0.16	0.05	0.12	0.10	0.05	0.00
**GT67**	0.00	0.00	0.00	0.00	0.00	0.00	0.00	0.00	0.00	0.00	0.00	0.00	0.00	0.00	0.00	0.00
**CE8**	0.35	0.29	0.29	0.26	0.14	0.16	0.25	0.34	0.16	0.17	0.21	0.17	0.19	0.10	0.15	0.07
**GT86**	0.00	0.00	0.00	0.00	0.00	0.00	0.00	0.00	0.00	0.00	0.00	0.00	0.00	0.00	0.00	0.00
**GH101**	0.00	0.00	0.00	0.00	0.00	0.00	0.00	0.00	0.00	0.00	0.00	0.00	0.00	0.00	0.18	0.04
**GH81**	0.00	0.02	0.00	0.00	0.00	0.00	0.00	0.00	0.04	0.00	0.00	0.00	0.00	0.00	0.00	0.00
**GT93**	0.00	0.00	0.00	0.00	0.00	0.00	0.00	0.00	0.00	0.00	0.00	0.00	0.00	0.00	0.00	0.00
**GT63**	0.00	0.00	0.00	0.00	0.00	0.00	0.00	0.00	0.00	0.00	0.00	0.00	0.00	0.00	0.00	0.00
**CE11**	0.24	0.22	0.23	0.29	0.16	0.19	0.28	0.21	0.16	0.11	0.23	0.17	0.24	0.34	0.23	0.40
**GT19**	0.17	0.11	0.10	0.15	0.09	0.17	0.16	0.21	0.04	0.07	0.07	0.22	0.14	0.16	0.18	0.22
**GT33**	0.00	0.00	0.00	0.00	0.00	0.00	0.00	0.00	0.00	0.00	0.00	0.00	0.00	0.00	0.00	0.00
**GH21**	0.00	0.00	0.00	0.00	0.00	0.00	0.00	0.00	0.00	0.00	0.00	0.00	0.00	0.00	0.00	0.00
**GT68**	0.00	0.00	0.00	0.00	0.00	0.00	0.00	0.00	0.00	0.00	0.00	0.00	0.00	0.00	0.00	0.00
**GH89**	0.44	0.38	0.10	0.15	0.08	0.25	0.33	0.39	0.14	0.13	0.16	0.37	0.19	0.18	0.05	0.26
**GT82**	0.00	0.00	0.00	0.00	0.01	0.00	0.00	0.00	0.00	0.00	0.00	0.00	0.00	0.00	0.00	0.00
**GH30**	0.26	0.29	0.11	0.12	0.22	0.14	0.26	0.26	0.14	0.10	0.22	0.42	0.21	0.18	0.20	0.22
**GH20**	1.35	1.21	0.63	1.11	0.67	0.72	1.63	1.62	0.51	0.49	0.71	1.57	0.88	0.52	0.80	1.35
**GH52**	0.00	0.00	0.00	0.00	0.00	0.00	0.00	0.00	0.00	0.00	0.00	0.00	0.00	0.00	0.00	0.00
**GT84**	0.00	0.00	0.06	0.00	0.00	0.00	0.00	0.00	0.04	0.00	0.04	0.00	0.00	0.03	0.00	0.00
**GT15**	0.00	0.00	0.00	0.00	0.00	0.00	0.00	0.00	0.00	0.00	0.00	0.00	0.00	0.00	0.00	0.00
**GH93**	0.02	0.07	0.00	0.00	0.00	0.00	0.00	0.00	0.00	0.00	0.02	0.02	0.00	0.00	0.00	0.00
**PL16**	0.00	0.00	0.00	0.00	0.00	0.00	0.00	0.00	0.00	0.00	0.00	0.00	0.00	0.00	0.00	0.00
**GH26**	0.04	0.02	0.11	0.20	0.19	0.03	0.12	0.10	0.21	0.16	0.11	0.12	0.07	0.13	0.00	0.07

CAZY_group	CN-AD-71	CN-AD-72	CN-AD-73	CN-AD-74	CN-AD-75	CN-AD-76	CN-AD-77	CN-AD-78	CN-AD-79	CN-AD-80	CN-AD-81	CN-AD-82	CN-AD-83	CN-AD-84	CN-AD-85	CN-AD-86
GT75	0.00	0.00	0.00	0.00	0.00	0.00	0.00	0.00	0.00	0.00	0.00	0.00	0.00	0.00	0.00	0.00
GH53	0.07	0.04	0.26	0.20	0.13	0.00	0.19	0.05	0.12	0.08	0.20	0.00	0.07	0.18	0.10	0.04
GT88	0.00	0.00	0.00	0.00	0.00	0.00	0.00	0.00	0.00	0.00	0.00	0.00	0.00	0.00	0.00	0.00
GT24	0.00	0.00	0.00	0.00	0.00	0.00	0.00	0.00	0.00	0.00	0.00	0.00	0.00	0.00	0.00	0.00
GT81	0.00	0.00	0.00	0.00	0.00	0.00	0.00	0.00	0.00	0.00	0.00	0.00	0.00	0.00	0.00	0.00
GH4	0.11	0.04	0.05	0.00	0.07	0.08	0.03	0.00	0.04	0.11	0.13	0.07	0.14	0.18	0.35	0.22
GT78	0.00	0.00	0.00	0.00	0.00	0.00	0.00	0.00	0.00	0.00	0.00	0.00	0.00	0.00	0.00	0.00
GH8	0.09	0.00	0.13	0.06	0.08	0.02	0.05	0.00	0.04	0.06	0.10	0.02	0.05	0.03	0.10	0.04
GH70	0.00	0.00	0.00	0.00	0.00	0.00	0.00	0.00	0.00	0.00	0.00	0.00	0.00	0.00	0.00	0.00
GH122	0.00	0.00	0.00	0.00	0.00	0.00	0.00	0.00	0.00	0.00	0.00	0.00	0.00	0.00	0.00	0.00
GH74	0.04	0.00	0.11	0.00	0.06	0.00	0.09	0.00	0.04	0.04	0.02	0.00	0.03	0.03	0.00	0.00
GT41	0.00	0.00	0.02	0.00	0.00	0.00	0.00	0.00	0.00	0.00	0.00	0.00	0.02	0.00	0.00	0.04
PL1	0.37	0.52	0.27	0.26	0.23	0.12	0.53	0.50	0.16	0.14	0.26	0.37	0.36	0.16	0.18	0.26
GH15	0.04	0.02	0.06	0.06	0.02	0.02	0.05	0.08	0.00	0.03	0.01	0.07	0.03	0.00	0.03	0.11
GH32	0.61	0.56	0.29	0.58	0.32	0.28	0.43	0.42	0.29	0.31	0.33	0.32	0.46	0.29	0.65	0.40
GH44	0.00	0.00	0.02	0.00	0.03	0.00	0.00	0.00	0.00	0.03	0.00	0.00	0.00	0.00	0.00	0.00
GT6	0.00	0.00	0.00	0.00	0.00	0.00	0.00	0.00	0.00	0.00	0.00	0.00	0.00	0.00	0.00	0.00
GH13	2.44	2.31	3.10	3.21	2.95	1.47	2.72	2.04	2.16	2.06	2.65	1.74	2.36	2.05	2.26	1.76
GT80	0.00	0.00	0.00	0.00	0.00	0.00	0.03	0.00	0.00	0.00	0.00	0.00	0.00	0.03	0.03	0.07
GH71	0.00	0.00	0.00	0.00	0.00	0.00	0.00	0.00	0.00	0.00	0.00	0.00	0.00	0.00	0.00	0.00
GH63	0.15	0.11	0.11	0.26	0.08	0.03	0.16	0.21	0.06	0.06	0.02	0.07	0.00	0.10	0.15	0.29
PL21	0.04	0.00	0.03	0.12	0.03	0.06	0.05	0.05	0.04	0.06	0.02	0.07	0.00	0.05	0.08	0.00
GH65	0.13	0.13	0.06	0.12	0.23	0.12	0.12	0.10	0.14	0.17	0.15	0.17	0.15	0.13	0.18	0.18
CE4	0.52	0.49	0.68	0.47	0.47	0.34	0.51	0.45	0.49	0.42	0.54	0.32	0.38	0.42	0.45	0.29
CE14	0.02	0.02	0.03	0.00	0.03	0.00	0.03	0.03	0.00	0.00	0.00	0.05	0.00	0.03	0.05	0.04
GH115	0.44	0.31	0.21	0.35	0.18	0.14	0.31	0.39	0.08	0.07	0.23	0.42	0.40	0.13	0.13	0.18
GH104	0.00	0.00	0.00	0.00	0.00	0.00	0.00	0.00	0.00	0.00	0.00	0.00	0.02	0.00	0.03	0.04
GH111	0.00	0.00	0.00	0.00	0.00	0.00	0.00	0.00	0.00	0.00	0.00	0.00	0.00	0.00	0.00	0.00
GH95	0.76	0.69	0.32	0.67	0.33	0.44	0.82	1.10	0.35	0.27	0.40	0.62	0.57	0.36	0.25	0.73
GT70	0.00	0.00	0.00	0.00	0.00	0.00	0.00	0.00	0.00	0.00	0.00	0.00	0.00	0.00	0.00	0.00
CE10	0.17	0.25	0.06	0.15	0.10	0.16	0.23	0.13	0.10	0.10	0.09	0.27	0.21	0.10	0.25	0.40
GH10	0.09	0.09	0.10	0.32	0.19	0.05	0.11	0.05	0.04	0.08	0.15	0.12	0.17	0.10	0.10	0.00
GH130	0.30	0.36	0.29	0.32	0.24	0.17	0.51	0.34	0.21	0.21	0.26	0.52	0.31	0.16	0.10	0.37
PL20	0.00	0.00	0.00	0.00	0.00	0.00	0.00	0.00	0.00	0.00	0.00	0.00	0.00	0.00	0.00	0.00
GH69	0.00	0.00	0.00	0.00	0.00	0.00	0.00	0.00	0.00	0.00	0.00	0.00	0.00	0.00	0.00	0.00
PL2	0.00	0.00	0.00	0.00	0.00	0.00	0.00	0.00	0.00	0.00	0.00	0.00	0.02	0.00	0.00	0.00
GH49	0.00	0.00	0.00	0.00	0.00	0.00	0.00	0.00	0.00	0.00	0.00	0.00	0.00	0.00	0.00	0.00
GH25	0.22	0.27	0.39	0.38	0.42	0.16	0.34	0.31	0.29	0.42	0.32	0.15	0.29	0.36	0.40	0.15
GH59	0.00	0.00	0.11	0.00	0.03	0.00	0.00	0.00	0.06	0.03	0.01	0.00	0.00	0.03	0.00	0.00
GH35	0.74	0.38	0.18	0.23	0.24	0.27	0.50	0.55	0.19	0.14	0.23	0.67	0.46	0.31	0.25	0.62
GT39	0.00	0.00	0.00	0.00	0.00	0.00	0.00	0.00	0.00	0.00	0.00	0.00	0.00	0.00	0.00	0.00
GT57	0.00	0.00	0.00	0.00	0.00	0.00	0.00	0.00	0.00	0.00	0.00	0.00	0.00	0.00	0.00	0.00
GH129	0.00	0.00	0.00	0.00	0.00	0.00	0.00	0.00	0.00	0.00	0.00	0.00	0.00	0.00	0.03	0.00
GT22	0.00	0.00	0.00	0.00	0.00	0.00	0.00	0.00	0.00	0.00	0.00	0.00	0.00	0.00	0.00	0.00
PL12	0.22	0.16	0.03	0.18	0.04	0.09	0.23	0.13	0.04	0.11	0.09	0.22	0.12	0.03	0.10	0.07
PL18	0.00	0.00	0.00	0.00	0.00	0.00	0.00	0.00	0.00	0.00	0.00	0.00	0.00	0.00	0.00	0.00
GT92	0.00	0.00	0.00	0.00	0.00	0.00	0.00	0.00	0.00	0.00	0.00	0.00	0.00	0.00	0.00	0.00
GT44	0.00	0.00	0.00	0.00	0.00	0.00	0.00	0.00	0.00	0.00	0.00	0.00	0.00	0.00	0.00	0.00
GT29	0.00	0.00	0.00	0.00	0.00	0.00	0.00	0.00	0.00	0.00	0.00	0.00	0.00	0.00	0.00	0.00
GT13	0.00	0.00	0.00	0.00	0.00	0.00	0.00	0.00	0.00	0.00	0.00	0.00	0.00	0.00	0.00	0.00

CAZY_group	CN-AD-71	CN-AD-72	CN-AD-73	CN-AD-74	CN-AD-75	CN-AD-76	CN-AD-77	CN-AD-78	CN-AD-79	CN-AD-80	CN-AD-81	CN-AD-82	CN-AD-83	CN-AD-84	CN-AD-85	CN-AD-86
**GT52**	0.00	0.00	0.00	0.00	0.00	0.00	0.00	0.00	0.00	0.00	0.00	0.00	0.00	0.00	0.00	0.00
**GT64**	0.00	0.00	0.00	0.00	0.00	0.00	0.00	0.00	0.00	0.00	0.00	0.00	0.00	0.00	0.00	0.00
**GT85**	0.00	0.00	0.00	0.00	0.00	0.00	0.00	0.00	0.00	0.00	0.00	0.00	0.00	0.00	0.00	0.00
**GT16**	0.00	0.00	0.00	0.00	0.00	0.00	0.00	0.00	0.00	0.00	0.00	0.00	0.00	0.00	0.00	0.00
**GH56**	0.00	0.00	0.00	0.00	0.00	0.00	0.00	0.00	0.00	0.00	0.00	0.00	0.00	0.00	0.00	0.00
**GT60**	0.00	0.00	0.00	0.00	0.00	0.00	0.00	0.00	0.00	0.00	0.00	0.00	0.00	0.00	0.00	0.00
**GH41**	0.00	0.00	0.00	0.00	0.00	0.00	0.00	0.00	0.00	0.00	0.00	0.00	0.00	0.00	0.00	0.00
**CE7**	0.13	0.13	0.05	0.18	0.04	0.05	0.19	0.16	0.08	0.08	0.06	0.20	0.17	0.08	0.10	0.00
**GH39**	0.02	0.02	0.03	0.12	0.03	0.00	0.05	0.00	0.06	0.04	0.13	0.00	0.05	0.00	0.03	0.04
**GT30**	0.33	0.25	0.13	0.23	0.14	0.31	0.31	0.39	0.21	0.14	0.18	0.45	0.33	0.29	0.20	0.33
**PL15**	0.17	0.16	0.02	0.09	0.01	0.17	0.16	0.16	0.08	0.07	0.05	0.25	0.09	0.03	0.03	0.11
**GH102**	0.02	0.02	0.00	0.03	0.00	0.05	0.00	0.00	0.00	0.00	0.04	0.02	0.03	0.03	0.08	0.11

CAZY_group	CN-AD-87	CN-AD-88	CN-AD-89	CN-AD-90	CN-AD-91	CN-AD-92	CN-AD-93	CN-AD-94	CN-AD-95	CN-AD-96	CN-AD-97	CN-AD-98	CN-AD-99	CN-AD-100	CN-AD-101	CN-AD-102
GH29	0.17	0.80	0.82	0.31	0.64	0.64	0.84	0.46	0.49	0.68	0.58	0.54	0.52	0.69	0.51	0.62
PL4	0.00	0.00	0.00	0.00	0.00	0.00	0.00	0.00	0.00	0.00	0.00	0.00	0.00	0.00	0.00	0.00
GH23	0.86	1.48	1.11	1.18	0.86	0.85	0.93	1.09	0.54	0.99	0.84	1.03	1.01	0.78	0.60	0.96
CE6	0.03	0.05	0.08	0.05	0.07	0.04	0.01	0.08	0.10	0.06	0.02	0.03	0.06	0.07	0.11	0.03
GH72	0.06	0.12	0.08	0.03	0.09	0.11	0.08	0.08	0.05	0.07	0.08	0.08	0.06	0.02	0.17	0.03
GH114	0.00	0.00	0.00	0.00	0.00	0.00	0.00	0.00	0.00	0.00	0.00	0.00	0.00	0.00	0.00	0.00
GH78	0.17	0.99	0.87	0.65	0.75	0.57	0.69	0.32	0.10	0.91	0.62	0.49	1.00	0.84	0.17	0.51
PL19	0.00	0.00	0.00	0.00	0.00	0.00	0.00	0.00	0.00	0.00	0.00	0.00	0.00	0.00	0.00	0.00
GT27	0.00	0.00	0.00	0.00	0.00	0.00	0.00	0.00	0.00	0.00	0.00	0.00	0.00	0.00	0.00	0.00
GH5	0.59	0.29	0.25	0.19	0.33	0.25	0.44	0.48	0.49	0.21	0.48	0.44	0.22	0.29	0.29	0.33
GT40	0.00	0.00	0.00	0.00	0.00	0.00	0.00	0.00	0.00	0.00	0.00	0.00	0.00	0.00	0.00	0.00
CE13	0.00	0.00	0.00	0.00	0.00	0.00	0.00	0.00	0.00	0.00	0.00	0.00	0.00	0.00	0.00	0.00
GT37	0.00	0.00	0.00	0.00	0.00	0.00	0.00	0.00	0.00	0.00	0.00	0.00	0.00	0.00	0.00	0.00
GH33	0.02	0.24	0.25	0.27	0.18	0.14	0.24	0.21	0.05	0.27	0.17	0.17	0.15	0.20	0.03	0.09
GT65	0.00	0.00	0.00	0.00	0.00	0.00	0.00	0.00	0.00	0.00	0.00	0.00	0.00	0.00	0.00	0.00
GH86	0.00	0.00	0.00	0.00	0.00	0.00	0.00	0.00	0.00	0.00	0.01	0.00	0.00	0.00	0.00	0.00
GH123	0.00	0.12	0.12	0.10	0.04	0.14	0.15	0.11	0.05	0.15	0.11	0.05	0.14	0.22	0.09	0.08
GH96	0.00	0.00	0.00	0.00	0.00	0.00	0.00	0.00	0.00	0.00	0.00	0.00	0.00	0.00	0.00	0.00
GH14	0.00	0.00	0.00	0.00	0.00	0.00	0.00	0.00	0.00	0.00	0.00	0.00	0.00	0.00	0.00	0.00
CE3	0.00	0.00	0.00	0.00	0.00	0.00	0.00	0.00	0.00	0.00	0.00	0.00	0.00	0.00	0.00	0.00
PL10	0.08	0.24	0.17	0.07	0.11	0.18	0.13	0.06	0.20	0.09	0.09	0.14	0.11	0.20	0.06	0.11
GT48	0.00	0.00	0.00	0.00	0.00	0.00	0.00	0.00	0.00	0.00	0.00	0.00	0.00	0.00	0.00	0.00
PL6	0.00	0.00	0.00	0.00	0.00	0.00	0.01	0.00	0.00	0.01	0.00	0.00	0.00	0.00	0.00	0.01
GT83	0.00	0.07	0.10	0.12	0.07	0.18	0.11	0.06	0.10	0.13	0.05	0.05	0.15	0.18	0.06	0.06
GH126	0.00	0.00	0.00	0.00	0.00	0.00	0.00	0.00	0.00	0.00	0.00	0.00	0.00	0.00	0.00	0.00
GH9	0.23	0.10	0.13	0.15	0.00	0.07	0.31	0.42	0.10	0.22	0.06	0.15	0.22	0.09	0.43	0.21
GH75	0.00	0.00	0.00	0.00	0.00	0.00	0.00	0.00	0.00	0.00	0.00	0.00	0.00	0.00	0.00	0.00
GT71	0.00	0.00	0.00	0.00	0.00	0.00	0.00	0.00	0.00	0.00	0.00	0.00	0.00	0.00	0.00	0.00
GH46	0.00	0.00	0.00	0.00	0.00	0.00	0.00	0.00	0.00	0.00	0.00	0.00	0.00	0.00	0.00	0.00
GT61	0.00	0.00	0.00	0.00	0.00	0.00	0.00	0.00	0.00	0.00	0.00	0.00	0.00	0.00	0.00	0.00
GT3	0.06	0.10	0.17	0.07	0.09	0.07	0.15	0.15	0.29	0.07	0.06	0.13	0.15	0.09	0.26	0.09
PL3	0.00	0.00	0.00	0.00	0.00	0.00	0.00	0.00	0.00	0.00	0.00	0.00	0.00	0.02	0.00	0.01
GH108	0.02	0.00	0.04	0.03	0.00	0.00	0.02	0.02	0.05	0.04	0.01	0.03	0.01	0.00	0.03	0.03
GT56	0.00	0.00	0.04	0.00	0.00	0.00	0.00	0.02	0.05	0.01	0.00	0.01	0.01	0.02	0.00	0.00
PL11	0.00	0.19	0.14	0.09	0.07	0.14	0.11	0.08	0.00	0.15	0.17	0.18	0.17	0.09	0.06	0.11
GH45	0.00	0.00	0.00	0.00	0.00	0.00	0.00	0.00	0.00	0.00	0.00	0.00	0.00	0.00	0.00	0.00
GT34	0.00	0.00	0.00	0.00	0.00	0.00	0.00	0.00	0.00	0.00	0.00	0.00	0.00	0.00	0.00	0.00
PL14	0.00	0.00	0.00	0.00	0.00	0.00	0.00	0.00	0.00	0.00	0.00	0.00	0.00	0.00	0.00	0.00
GH19	0.00	0.00	0.02	0.00	0.02	0.00	0.00	0.02	0.00	0.00	0.01	0.01	0.00	0.00	0.00	0.00
GH118	0.00	0.00	0.00	0.00	0.00	0.00	0.00	0.00	0.00	0.00	0.00	0.00	0.00	0.00	0.00	0.00
GH82	0.00	0.00	0.00	0.00	0.00	0.00	0.00	0.00	0.00	0.00	0.00	0.00	0.00	0.00	0.00	0.00
GH76	0.00	0.41	0.24	0.17	0.33	0.00	0.20	0.11	0.00	0.21	0.20	0.17	0.23	0.07	0.03	0.09
GT4	1.17	2.16	1.75	1.21	1.37	1.70	1.43	2.06	0.93	1.48	1.43	1.24	1.50	1.58	1.34	1.33
GT87	0.00	0.00	0.00	0.00	0.00	0.00	0.00	0.00	0.00	0.00	0.00	0.00	0.00	0.00	0.00	0.00
GH24	0.09	0.15	0.26	0.17	0.22	0.28	0.12	0.38	0.49	0.18	0.15	0.23	0.09	0.20	0.29	0.15
GH90	0.00	0.00	0.00	0.00	0.00	0.00	0.00	0.00	0.00	0.00	0.00	0.00	0.00	0.00	0.00	0.00
GH57	0.05	0.19	0.14	0.09	0.13	0.07	0.10	0.15	0.15	0.09	0.04	0.06	0.10	0.16	0.14	0.10
GH36	0.86	0.75	0.68	0.38	0.68	0.74	0.63	0.44	0.69	0.55	0.68	0.54	0.62	0.58	0.60	0.66
GT46	0.00	0.00	0.00	0.00	0.00	0.00	0.00	0.00	0.00	0.00	0.00	0.00	0.00	0.00	0.00	0.00
GH66	0.06	0.07	0.02	0.05	0.02	0.00	0.06	0.02	0.05	0.04	0.04	0.03	0.06	0.00	0.03	0.03

CAZY_group	CN-AD-87	CN-AD-88	CN-AD-89	CN-AD-90	CN-AD-91	CN-AD-92	CN-AD-93	CN-AD-94	CN-AD-95	CN-AD-96	CN-AD-97	CN-AD-98	CN-AD-99	CN-AD-100	CN-AD-101	CN-AD-102
GH48	0.00	0.00	0.00	0.00	0.00	0.00	0.01	0.02	0.00	0.00	0.00	0.00	0.00	0.00	0.00	0.01
GT53	0.00	0.00	0.00	0.00	0.00	0.00	0.00	0.00	0.00	0.00	0.00	0.00	0.00	0.00	0.00	0.00
GH60	0.00	0.00	0.00	0.00	0.00	0.00	0.00	0.00	0.00	0.00	0.00	0.00	0.00	0.00	0.00	0.00
GT31	0.00	0.00	0.01	0.00	0.00	0.00	0.00	0.00	0.00	0.00	0.00	0.01	0.01	0.02	0.00	0.00
GH105	0.16	0.85	0.48	0.39	0.44	0.64	0.52	0.21	0.10	0.58	0.63	0.60	0.60	0.29	0.23	0.42
GT23	0.02	0.02	0.01	0.00	0.00	0.04	0.01	0.00	0.00	0.00	0.02	0.00	0.00	0.00	0.00	0.05
GH11	0.00	0.00	0.00	0.00	0.00	0.00	0.00	0.00	0.00	0.00	0.01	0.00	0.00	0.00	0.00	0.00
GT38	0.00	0.00	0.00	0.00	0.00	0.00	0.00	0.00	0.00	0.00	0.00	0.00	0.00	0.00	0.00	0.00
GT43	0.00	0.00	0.00	0.00	0.00	0.00	0.00	0.00	0.00	0.00	0.00	0.00	0.00	0.00	0.00	0.00
GH99	0.00	0.02	0.04	0.00	0.02	0.00	0.01	0.02	0.00	0.01	0.01	0.01	0.02	0.00	0.00	0.00
GH38	0.17	0.12	0.15	0.12	0.11	0.18	0.18	0.25	0.20	0.18	0.14	0.17	0.21	0.07	0.06	0.20
GH109	0.05	0.29	0.26	0.17	0.15	0.32	0.30	0.23	0.15	0.25	0.26	0.26	0.17	0.27	0.23	0.15
GH2	2.42	4.46	3.92	2.85	3.27	3.33	3.84	3.05	1.72	4.28	3.77	3.32	4.10	2.77	1.80	2.96
GT28	0.53	0.36	0.33	0.29	0.31	0.57	0.29	0.34	0.29	0.32	0.33	0.37	0.29	0.38	0.31	0.33
GH42	0.41	0.24	0.15	0.14	0.22	0.21	0.15	0.08	0.00	0.18	0.14	0.19	0.16	0.02	0.09	0.14
GT58	0.00	0.00	0.00	0.00	0.00	0.00	0.00	0.00	0.00	0.00	0.00	0.00	0.00	0.00	0.00	0.00
GH61	0.00	0.00	0.00	0.00	0.00	0.00	0.00	0.00	0.00	0.00	0.00	0.00	0.00	0.00	0.00	0.00
GH80	0.00	0.00	0.00	0.00	0.00	0.00	0.00	0.00	0.00	0.00	0.00	0.00	0.00	0.00	0.00	0.00
GH98	0.00	0.00	0.00	0.00	0.00	0.00	0.01	0.04	0.00	0.00	0.00	0.00	0.00	0.00	0.00	0.00
GH6	0.00	0.00	0.00	0.00	0.00	0.00	0.00	0.00	0.00	0.00	0.01	0.00	0.00	0.00	0.00	0.00
GT1	0.06	0.00	0.02	0.00	0.02	0.04	0.03	0.04	0.05	0.01	0.04	0.03	0.02	0.02	0.00	0.03
GH28	0.47	1.36	0.88	0.58	0.62	0.78	0.63	0.71	0.74	0.75	0.86	0.85	0.94	0.58	0.51	0.62
GT42	0.00	0.00	0.00	0.00	0.00	0.00	0.00	0.00	0.00	0.00	0.00	0.00	0.00	0.00	0.00	0.00
GH128	0.00	0.00	0.00	0.00	0.00	0.00	0.00	0.00	0.00	0.00	0.00	0.00	0.00	0.00	0.00	0.00
CE12	0.12	0.19	0.10	0.10	0.09	0.14	0.08	0.04	0.10	0.16	0.14	0.09	0.19	0.04	0.14	0.06
CE16	0.00	0.00	0.00	0.00	0.00	0.00	0.00	0.00	0.00	0.00	0.00	0.00	0.00	0.00	0.00	0.00
GT89	0.00	0.00	0.00	0.00	0.00	0.00	0.00	0.00	0.00	0.00	0.00	0.00	0.00	0.00	0.00	0.00
GT11	0.05	0.05	0.08	0.05	0.15	0.14	0.08	0.11	0.05	0.03	0.02	0.04	0.02	0.04	0.00	0.05
GT77	0.00	0.00	0.01	0.00	0.00	0.04	0.01	0.04	0.00	0.00	0.01	0.01	0.03	0.04	0.06	0.00
GT55	0.00	0.00	0.00	0.00	0.00	0.00	0.00	0.00	0.00	0.00	0.00	0.00	0.00	0.00	0.00	0.00
GT18	0.00	0.00	0.00	0.00	0.00	0.00	0.00	0.00	0.00	0.00	0.00	0.00	0.00	0.00	0.00	0.00
GH87	0.02	0.00	0.00	0.00	0.00	0.00	0.00	0.00	0.00	0.00	0.00	0.01	0.00	0.00	0.00	0.00
PL5	0.00	0.00	0.00	0.00	0.00	0.00	0.00	0.00	0.00	0.00	0.00	0.00	0.00	0.00	0.00	0.00
GH22	0.00	0.00	0.00	0.02	0.00	0.00	0.00	0.00	0.00	0.00	0.00	0.00	0.00	0.00	0.00	0.00
CE2	0.03	0.12	0.06	0.00	0.00	0.14	0.03	0.06	0.00	0.06	0.07	0.08	0.04	0.02	0.06	0.07
GH120	0.11	0.05	0.01	0.02	0.00	0.07	0.02	0.06	0.00	0.03	0.04	0.01	0.05	0.00	0.00	0.06
GH73	0.52	0.39	0.40	0.31	0.40	0.35	0.30	0.34	0.44	0.37	0.28	0.32	0.33	0.47	0.40	0.36
GT17	0.00	0.00	0.00	0.00	0.00	0.00	0.00	0.00	0.00	0.00	0.00	0.00	0.00	0.00	0.00	0.00
GH124	0.00	0.00	0.00	0.00	0.00	0.00	0.00	0.00	0.00	0.00	0.00	0.00	0.00	0.00	0.00	0.00
GH54	0.00	0.00	0.00	0.00	0.00	0.00	0.00	0.00	0.00	0.00	0.00	0.00	0.00	0.00	0.00	0.00
GH100	0.00	0.00	0.00	0.00	0.00	0.00	0.00	0.00	0.00	0.00	0.00	0.00	0.00	0.00	0.00	0.00
GH113	0.03	0.05	0.00	0.00	0.00	0.14	0.01	0.02	0.00	0.01	0.03	0.06	0.01	0.00	0.03	0.05
GT5	0.61	0.27	0.27	0.32	0.42	0.35	0.29	0.36	0.29	0.24	0.38	0.38	0.20	0.18	0.37	0.36
GH94	0.94	0.29	0.16	0.10	0.33	0.46	0.33	0.34	0.15	0.12	0.47	0.53	0.15	0.07	0.49	0.44
GT66	0.00	0.00	0.00	0.00	0.00	0.00	0.00	0.00	0.00	0.00	0.00	0.00	0.00	0.00	0.00	0.01
GT94	0.00	0.00	0.00	0.00	0.00	0.00	0.00	0.00	0.00	0.00	0.00	0.00	0.00	0.00	0.00	0.00
GH58	0.00	0.00	0.00	0.00	0.00	0.00	0.00	0.00	0.00	0.00	0.00	0.00	0.00	0.00	0.00	0.00
GH1	1.30	0.56	0.36	0.15	0.24	0.43	0.20	0.21	0.20	0.28	0.35	0.54	0.39	0.16	0.40	0.39
PL22	0.00	0.00	0.01	0.00	0.00	0.00	0.00	0.00	0.00	0.00	0.00	0.00	0.00	0.00	0.00	0.00
GT9	0.19	0.17	0.27	0.20	0.11	0.11	0.06	0.21	0.29	0.19	0.06	0.13	0.24	0.35	0.14	0.07

CAZY_group	CN-AD-87	CN-AD-88	CN-AD-89	CN-AD-90	CN-AD-91	CN-AD-92	CN-AD-93	CN-AD-94	CN-AD-95	CN-AD-96	CN-AD-97	CN-AD-98	CN-AD-99	CN-AD-100	CN-AD-101	CN-AD-102
GT76	0.00	0.00	0.00	0.00	0.00	0.00	0.00	0.00	0.00	0.00	0.00	0.00	0.00	0.00	0.00	0.00
GT32	0.11	0.10	0.10	0.05	0.07	0.04	0.08	0.06	0.05	0.01	0.05	0.06	0.06	0.13	0.06	0.07
GT47	0.06	0.05	0.02	0.03	0.00	0.07	0.00	0.06	0.05	0.00	0.00	0.01	0.02	0.00	0.03	0.01
GH84	0.12	0.10	0.14	0.10	0.07	0.14	0.12	0.15	0.05	0.15	0.13	0.10	0.14	0.20	0.14	0.07
GH83	0.00	0.00	0.00	0.00	0.00	0.00	0.00	0.00	0.00	0.00	0.00	0.00	0.00	0.00	0.00	0.00
GT59	0.00	0.00	0.00	0.00	0.00	0.00	0.00	0.00	0.00	0.00	0.00	0.00	0.00	0.00	0.00	0.00
GH34	0.00	0.00	0.00	0.00	0.00	0.00	0.00	0.00	0.00	0.00	0.00	0.00	0.00	0.00	0.00	0.00
GT14	0.00	0.10	0.01	0.00	0.02	0.00	0.02	0.08	0.00	0.01	0.02	0.03	0.03	0.02	0.00	0.02
GH47	0.00	0.00	0.00	0.00	0.00	0.00	0.00	0.02	0.00	0.00	0.00	0.00	0.00	0.02	0.00	0.00
GH97	0.23	1.38	1.11	0.68	0.53	0.57	0.93	0.82	0.74	0.90	0.64	0.49	0.93	0.67	0.94	0.51
GH50	0.00	0.12	0.05	0.09	0.07	0.00	0.02	0.00	0.00	0.06	0.03	0.06	0.06	0.00	0.00	0.06
GT26	0.22	0.17	0.18	0.17	0.27	0.25	0.11	0.27	0.29	0.09	0.13	0.08	0.16	0.22	0.17	0.18
GH18	0.36	0.87	0.54	0.38	0.55	0.21	0.35	0.46	0.10	0.46	0.60	0.33	0.55	0.24	0.23	0.65
GH37	0.00	0.00	0.04	0.00	0.00	0.00	0.00	0.04	0.05	0.03	0.00	0.03	0.04	0.07	0.00	0.00
PL13	0.00	0.12	0.07	0.02	0.02	0.00	0.02	0.00	0.00	0.07	0.04	0.01	0.07	0.00	0.00	0.06
GH27	0.20	0.27	0.16	0.07	0.09	0.11	0.19	0.25	0.10	0.16	0.24	0.17	0.15	0.09	0.14	0.19
GT54	0.00	0.00	0.00	0.00	0.00	0.00	0.00	0.00	0.00	0.00	0.00	0.00	0.00	0.00	0.00	0.00
GT91	0.00	0.00	0.00	0.00	0.00	0.00	0.00	0.00	0.00	0.00	0.00	0.00	0.00	0.00	0.00	0.00
GT72	0.00	0.00	0.00	0.00	0.00	0.00	0.00	0.00	0.00	0.00	0.00	0.00	0.00	0.00	0.00	0.00
GH67	0.05	0.15	0.11	0.03	0.07	0.04	0.03	0.11	0.10	0.06	0.06	0.09	0.11	0.09	0.09	0.08
GH12	0.00	0.00	0.00	0.00	0.00	0.00	0.00	0.00	0.00	0.00	0.00	0.00	0.00	0.00	0.00	0.00
GH91	0.00	0.07	0.02	0.05	0.00	0.00	0.03	0.00	0.00	0.04	0.00	0.00	0.01	0.02	0.00	0.03
GT69	0.00	0.00	0.00	0.00	0.00	0.00	0.00	0.00	0.00	0.00	0.00	0.00	0.00	0.00	0.00	0.00
GH106	0.08	0.39	0.25	0.09	0.15	0.14	0.21	0.08	0.25	0.30	0.28	0.26	0.31	0.24	0.09	0.18
GT62	0.00	0.00	0.00	0.00	0.00	0.00	0.00	0.00	0.00	0.00	0.00	0.00	0.00	0.00	0.00	0.00
GH17	0.00	0.00	0.00	0.00	0.00	0.00	0.00	0.00	0.00	0.00	0.00	0.00	0.00	0.00	0.00	0.00
GH88	0.05	0.48	0.45	0.31	0.20	0.25	0.33	0.23	0.05	0.43	0.38	0.24	0.51	0.18	0.09	0.29
CE5	0.00	0.00	0.00	0.00	0.00	0.00	0.00	0.00	0.00	0.00	0.00	0.00	0.00	0.00	0.00	0.00
GT8	0.20	0.17	0.06	0.14	0.18	0.11	0.09	0.11	0.10	0.07	0.07	0.04	0.03	0.09	0.00	0.06
GT50	0.00	0.00	0.00	0.00	0.00	0.00	0.00	0.00	0.00	0.00	0.00	0.00	0.00	0.00	0.00	0.00
GT20	0.00	0.10	0.14	0.10	0.13	0.04	0.08	0.06	0.00	0.07	0.03	0.06	0.11	0.09	0.00	0.02
PL9	0.11	0.22	0.08	0.12	0.04	0.04	0.04	0.06	0.00	0.09	0.13	0.13	0.05	0.09	0.14	0.10
GH55	0.06	0.00	0.03	0.05	0.00	0.07	0.02	0.08	0.00	0.01	0.01	0.05	0.03	0.00	0.00	0.01
GH64	0.00	0.00	0.01	0.00	0.00	0.00	0.00	0.00	0.00	0.00	0.00	0.00	0.00	0.00	0.00	0.00
GH127	0.25	0.24	0.26	0.27	0.11	0.25	0.20	0.23	0.34	0.31	0.31	0.22	0.42	0.38	0.26	0.20
GH103	0.03	0.00	0.03	0.00	0.00	0.00	0.00	0.04	0.00	0.00	0.00	0.03	0.03	0.04	0.00	0.00
GH3	1.94	2.28	2.14	2.03	2.19	2.06	2.47	2.18	1.03	2.66	2.01	2.23	2.36	1.97	1.29	2.08
GT45	0.00	0.00	0.00	0.00	0.00	0.00	0.00	0.00	0.00	0.00	0.00	0.00	0.00	0.00	0.00	0.00
GT74	0.00	0.00	0.00	0.00	0.00	0.00	0.00	0.00	0.00	0.00	0.00	0.00	0.00	0.00	0.00	0.00
GT90	0.00	0.00	0.00	0.00	0.00	0.00	0.00	0.00	0.00	0.00	0.00	0.00	0.00	0.00	0.00	0.00
GH116	0.00	0.15	0.08	0.09	0.09	0.00	0.11	0.04	0.00	0.07	0.03	0.05	0.05	0.02	0.00	0.09
GH31	0.66	1.09	1.00	0.72	0.86	0.92	1.13	0.71	0.59	0.97	0.90	0.95	0.94	0.78	0.54	0.64
GT12	0.00	0.00	0.00	0.00	0.00	0.00	0.00	0.00	0.00	0.00	0.00	0.00	0.00	0.00	0.00	0.00
CE1	0.28	0.44	0.39	0.29	0.27	0.32	0.26	0.36	0.15	0.37	0.26	0.31	0.36	0.38	0.09	0.26
GH121	0.00	0.00	0.00	0.00	0.00	0.00	0.00	0.00	0.00	0.00	0.00	0.00	0.00	0.00	0.00	0.00
GH79	0.00	0.00	0.00	0.00	0.00	0.00	0.00	0.00	0.00	0.00	0.01	0.00	0.01	0.00	0.00	0.02
GT73	0.00	0.00	0.01	0.00	0.00	0.00	0.00	0.02	0.00	0.00	0.00	0.00	0.00	0.00	0.00	0.00
GH77	0.69	0.53	0.45	0.43	0.38	0.50	0.48	0.38	0.20	0.35	0.50	0.54	0.44	0.33	0.49	0.44
PL17	0.00	0.02	0.02	0.02	0.02	0.00	0.00	0.02	0.05	0.00	0.02	0.00	0.01	0.02	0.03	0.00
PL8	0.00	0.48	0.41	0.20	0.24	0.00	0.31	0.17	0.00	0.44	0.24	0.13	0.42	0.22	0.20	0.20

CAZY_group	CN-AD-87	CN-AD-88	CN-AD-89	CN-AD-90	CN-AD-91	CN-AD-92	CN-AD-93	CN-AD-94	CN-AD-95	CN-AD-96	CN-AD-97	CN-AD-98	CN-AD-99	CN-AD-100	CN-AD-101	CN-AD-102
**CE9**	0.28	0.44	0.35	0.20	0.38	0.39	0.27	0.23	0.20	0.25	0.25	0.37	0.29	0.38	0.14	0.26
**CE15**	0.00	0.00	0.00	0.02	0.00	0.00	0.01	0.00	0.00	0.00	0.01	0.00	0.00	0.00	0.00	0.00
**GT79**	0.00	0.00	0.00	0.00	0.00	0.00	0.00	0.00	0.00	0.00	0.00	0.00	0.00	0.00	0.00	0.00
**GT10**	0.00	0.00	0.00	0.02	0.02	0.00	0.02	0.00	0.00	0.00	0.01	0.00	0.00	0.02	0.00	0.00
**GH119**	0.00	0.00	0.00	0.00	0.00	0.00	0.00	0.00	0.00	0.00	0.00	0.00	0.00	0.00	0.00	0.00
**GH16**	0.09	0.22	0.21	0.10	0.20	0.11	0.25	0.13	0.15	0.19	0.17	0.15	0.16	0.13	0.14	0.14
**GH43**	1.17	2.62	2.62	1.35	1.75	1.28	1.97	1.81	1.37	2.10	1.84	1.69	2.05	1.64	1.23	1.27
**GH51**	0.64	0.44	0.54	0.31	0.38	0.53	0.53	0.50	0.74	0.52	0.61	0.45	0.51	0.44	0.31	0.41
**GT35**	1.16	0.73	0.62	0.56	0.75	0.78	0.52	0.69	0.59	0.44	0.76	0.86	0.49	0.53	0.83	0.75
**GT25**	0.08	0.02	0.00	0.03	0.00	0.00	0.00	0.00	0.00	0.01	0.00	0.01	0.00	0.00	0.00	0.00
**GT2**	3.72	5.07	3.75	2.99	3.36	4.43	3.49	3.82	2.79	3.33	3.33	3.52	3.51	2.75	3.46	3.49
**GT51**	0.86	0.95	1.02	0.80	0.71	0.89	0.67	0.86	0.69	0.78	0.69	0.86	0.75	0.98	0.91	0.55
**GH107**	0.00	0.00	0.00	0.00	0.00	0.00	0.00	0.00	0.00	0.00	0.00	0.00	0.00	0.00	0.00	0.00
**GH7**	0.00	0.00	0.00	0.00	0.00	0.00	0.00	0.00	0.00	0.00	0.00	0.00	0.00	0.00	0.00	0.00
**GT7**	0.00	0.00	0.00	0.00	0.00	0.00	0.00	0.00	0.00	0.00	0.00	0.00	0.00	0.00	0.00	0.00
**GT36**	0.00	0.00	0.00	0.00	0.00	0.00	0.00	0.00	0.00	0.00	0.00	0.00	0.00	0.00	0.00	0.00
**PL7**	0.00	0.00	0.00	0.00	0.00	0.00	0.00	0.00	0.00	0.00	0.00	0.00	0.00	0.00	0.00	0.00
**GH110**	0.03	0.10	0.16	0.09	0.13	0.04	0.19	0.15	0.05	0.18	0.12	0.05	0.10	0.13	0.11	0.06
**GT21**	0.00	0.00	0.00	0.00	0.00	0.00	0.00	0.00	0.00	0.00	0.00	0.00	0.00	0.00	0.00	0.00
**GH62**	0.00	0.00	0.00	0.00	0.00	0.00	0.00	0.00	0.00	0.00	0.00	0.00	0.00	0.00	0.00	0.00
**GH125**	0.08	0.24	0.17	0.26	0.07	0.07	0.16	0.15	0.05	0.12	0.11	0.12	0.14	0.11	0.09	0.14
**GH117**	0.00	0.15	0.11	0.09	0.09	0.04	0.12	0.13	0.00	0.16	0.09	0.04	0.11	0.11	0.03	0.07
**GH68**	0.03	0.00	0.00	0.00	0.00	0.00	0.00	0.00	0.00	0.00	0.00	0.00	0.00	0.00	0.00	0.00
**GH85**	0.05	0.00	0.00	0.00	0.00	0.14	0.00	0.00	0.10	0.00	0.02	0.00	0.01	0.00	0.03	0.00
**GH92**	0.11	2.42	1.73	1.35	1.10	0.60	1.61	0.80	0.44	1.45	1.39	1.08	1.58	1.69	0.91	1.33
**GH40**	0.00	0.00	0.00	0.00	0.00	0.00	0.00	0.00	0.00	0.00	0.00	0.00	0.00	0.00	0.00	0.00
**GT49**	0.00	0.00	0.00	0.00	0.00	0.00	0.00	0.00	0.00	0.00	0.00	0.00	0.00	0.00	0.00	0.00
**GH112**	0.23	0.15	0.09	0.07	0.18	0.21	0.13	0.15	0.15	0.12	0.11	0.18	0.18	0.04	0.11	0.15
**GT67**	0.00	0.00	0.00	0.00	0.00	0.00	0.00	0.00	0.00	0.00	0.00	0.00	0.00	0.00	0.00	0.00
**CE8**	0.16	0.41	0.22	0.15	0.20	0.25	0.20	0.21	0.29	0.22	0.27	0.26	0.16	0.16	0.17	0.16
**GT86**	0.00	0.00	0.00	0.00	0.00	0.00	0.00	0.00	0.00	0.00	0.00	0.00	0.00	0.00	0.00	0.00
**GH101**	0.02	0.00	0.00	0.00	0.02	0.00	0.00	0.02	0.00	0.04	0.00	0.00	0.02	0.00	0.00	0.01
**GH81**	0.00	0.00	0.00	0.00	0.00	0.00	0.00	0.00	0.00	0.00	0.00	0.00	0.00	0.00	0.00	0.00
**GT93**	0.00	0.00	0.00	0.00	0.00	0.00	0.00	0.00	0.00	0.00	0.00	0.00	0.00	0.00	0.00	0.00
**GT63**	0.00	0.00	0.00	0.00	0.00	0.00	0.00	0.00	0.00	0.00	0.00	0.00	0.00	0.00	0.00	0.00
**CE11**	0.17	0.34	0.26	0.26	0.13	0.28	0.25	0.15	0.20	0.24	0.18	0.18	0.25	0.29	0.20	0.16
**GT19**	0.11	0.17	0.23	0.15	0.07	0.14	0.10	0.17	0.10	0.12	0.05	0.10	0.17	0.18	0.23	0.07
**GT33**	0.00	0.00	0.00	0.00	0.00	0.00	0.00	0.00	0.00	0.00	0.00	0.00	0.00	0.00	0.00	0.00
**GH21**	0.00	0.00	0.00	0.00	0.00	0.00	0.00	0.00	0.00	0.00	0.00	0.00	0.00	0.00	0.00	0.00
**GT68**	0.00	0.00	0.00	0.00	0.00	0.00	0.00	0.00	0.00	0.00	0.00	0.00	0.00	0.00	0.00	0.00
**GH89**	0.02	0.36	0.24	0.12	0.24	0.07	0.22	0.11	0.10	0.27	0.24	0.19	0.23	0.13	0.11	0.17
**GT82**	0.00	0.00	0.00	0.00	0.00	0.00	0.00	0.00	0.00	0.00	0.00	0.00	0.00	0.00	0.00	0.00
**GH30**	0.08	0.32	0.23	0.17	0.15	0.18	0.25	0.17	0.15	0.28	0.20	0.21	0.22	0.16	0.09	0.15
**GH20**	0.19	1.53	1.10	0.94	0.80	0.99	1.32	0.69	0.69	1.21	0.88	0.65	1.03	0.91	0.40	0.87
**GH52**	0.00	0.00	0.00	0.00	0.00	0.00	0.00	0.00	0.00	0.00	0.00	0.00	0.00	0.00	0.00	0.00
**GT84**	0.03	0.00	0.00	0.00	0.02	0.00	0.00	0.00	0.00	0.00	0.00	0.00	0.00	0.00	0.00	0.08
**GT15**	0.00	0.00	0.00	0.00	0.00	0.00	0.00	0.00	0.00	0.00	0.00	0.00	0.00	0.00	0.00	0.00
**GH93**	0.00	0.00	0.02	0.00	0.00	0.00	0.01	0.02	0.00	0.01	0.00	0.00	0.01	0.00	0.00	0.00
**PL16**	0.00	0.00	0.00	0.00	0.00	0.00	0.00	0.00	0.00	0.00	0.00	0.00	0.00	0.00	0.00	0.00
**GH26**	0.11	0.05	0.02	0.05	0.02	0.04	0.12	0.21	0.15	0.04	0.11	0.13	0.08	0.04	0.00	0.14

CAZY_group	CN-AD-87	CN-AD-88	CN-AD-89	CN-AD-90	CN-AD-91	CN-AD-92	CN-AD-93	CN-AD-94	CN-AD-95	CN-AD-96	CN-AD-97	CN-AD-98	CN-AD-99	CN-AD-100	CN-AD-101	CN-AD-102
GT75	0.00	0.00	0.00	0.00	0.00	0.00	0.00	0.00	0.00	0.00	0.00	0.00	0.00	0.00	0.00	0.00
GH53	0.20	0.12	0.09	0.02	0.04	0.18	0.06	0.08	0.15	0.04	0.19	0.12	0.09	0.02	0.03	0.24
GT88	0.00	0.00	0.00	0.00	0.00	0.00	0.00	0.00	0.00	0.00	0.00	0.00	0.00	0.00	0.00	0.00
GT24	0.00	0.00	0.00	0.00	0.00	0.00	0.00	0.00	0.00	0.00	0.00	0.00	0.00	0.00	0.00	0.00
GT81	0.00	0.00	0.00	0.00	0.00	0.00	0.00	0.00	0.00	0.00	0.00	0.00	0.00	0.00	0.00	0.00
GH4	0.14	0.10	0.25	0.09	0.13	0.04	0.01	0.08	0.10	0.06	0.09	0.14	0.10	0.13	0.03	0.09
GT78	0.00	0.00	0.00	0.00	0.00	0.00	0.00	0.00	0.00	0.00	0.00	0.00	0.00	0.00	0.00	0.00
GH8	0.03	0.02	0.09	0.02	0.00	0.04	0.04	0.08	0.05	0.09	0.05	0.04	0.05	0.11	0.09	0.03
GH70	0.28	0.00	0.00	0.00	0.00	0.00	0.00	0.00	0.00	0.01	0.00	0.00	0.00	0.00	0.00	0.00
GH122	0.00	0.00	0.00	0.00	0.00	0.00	0.00	0.00	0.00	0.00	0.00	0.00	0.00	0.00	0.00	0.00
GH74	0.02	0.05	0.00	0.00	0.00	0.04	0.06	0.08	0.00	0.00	0.03	0.04	0.00	0.00	0.00	0.01
GT41	0.03	0.00	0.03	0.02	0.00	0.07	0.00	0.00	0.00	0.00	0.00	0.00	0.00	0.00	0.03	0.00
PL1	0.14	0.48	0.22	0.17	0.33	0.21	0.18	0.08	0.10	0.35	0.28	0.24	0.38	0.07	0.11	0.16
GH15	0.00	0.05	0.06	0.02	0.13	0.07	0.03	0.04	0.00	0.01	0.02	0.04	0.03	0.04	0.00	0.05
GH32	0.80	0.53	0.33	0.41	0.49	0.28	0.31	0.27	0.34	0.40	0.37	0.29	0.46	0.38	0.40	0.38
GH44	0.00	0.00	0.00	0.00	0.00	0.00	0.02	0.00	0.00	0.00	0.00	0.01	0.00	0.00	0.00	0.00
GT6	0.00	0.00	0.00	0.00	0.00	0.00	0.00	0.00	0.00	0.00	0.00	0.00	0.01	0.00	0.00	0.00
GH13	3.70	2.59	2.61	1.62	2.30	3.22	2.29	2.23	1.91	2.05	2.55	2.87	1.91	1.62	2.23	2.77
GT80	0.00	0.02	0.01	0.00	0.00	0.00	0.01	0.02	0.05	0.01	0.02	0.01	0.01	0.02	0.00	0.02
GH71	0.00	0.00	0.00	0.00	0.00	0.00	0.00	0.00	0.00	0.00	0.00	0.00	0.00	0.00	0.00	0.00
GH63	0.03	0.07	0.08	0.07	0.07	0.14	0.12	0.17	0.05	0.06	0.09	0.15	0.14	0.13	0.03	0.07
PL21	0.00	0.12	0.09	0.03	0.00	0.00	0.02	0.00	0.00	0.03	0.04	0.04	0.06	0.00	0.00	0.05
GH65	0.20	0.15	0.13	0.14	0.13	0.14	0.16	0.13	0.05	0.12	0.11	0.15	0.14	0.04	0.06	0.17
CE4	0.64	0.48	0.44	0.29	0.51	0.53	0.35	0.46	0.39	0.31	0.60	0.59	0.40	0.33	0.31	0.55
CE14	0.00	0.05	0.05	0.02	0.02	0.00	0.01	0.00	0.00	0.01	0.02	0.00	0.04	0.02	0.00	0.01
GH115	0.17	0.51	0.24	0.05	0.27	0.11	0.20	0.08	0.15	0.27	0.28	0.22	0.31	0.16	0.17	0.11
GH104	0.00	0.00	0.00	0.00	0.00	0.00	0.00	0.02	0.00	0.00	0.00	0.01	0.00	0.02	0.00	0.00
GH111	0.00	0.00	0.00	0.00	0.00	0.00	0.00	0.00	0.00	0.00	0.00	0.00	0.00	0.00	0.00	0.00
GH95	0.30	0.75	0.65	0.49	0.66	0.21	0.50	0.32	0.59	0.68	0.56	0.45	0.56	0.40	0.37	0.44
GT70	0.00	0.00	0.00	0.00	0.00	0.00	0.00	0.00	0.00	0.00	0.00	0.00	0.00	0.00	0.00	0.00
CE10	0.02	0.15	0.24	0.10	0.22	0.07	0.13	0.21	0.05	0.22	0.08	0.09	0.24	0.18	0.11	0.11
GH10	0.11	0.15	0.14	0.07	0.13	0.07	0.02	0.19	0.05	0.09	0.16	0.13	0.09	0.02	0.09	0.07
GH130	0.20	0.32	0.27	0.27	0.18	0.39	0.21	0.21	0.10	0.21	0.37	0.44	0.34	0.22	0.23	0.28
PL20	0.00	0.00	0.00	0.00	0.00	0.00	0.00	0.00	0.00	0.00	0.00	0.00	0.00	0.00	0.00	0.00
GH69	0.00	0.00	0.00	0.00	0.00	0.00	0.00	0.00	0.00	0.00	0.00	0.00	0.00	0.00	0.00	0.00
PL2	0.00	0.00	0.00	0.00	0.00	0.00	0.00	0.00	0.00	0.00	0.00	0.00	0.00	0.02	0.00	0.00
GH49	0.00	0.00	0.00	0.00	0.00	0.00	0.00	0.00	0.00	0.00	0.00	0.00	0.00	0.00	0.00	0.00
GH25	0.47	0.34	0.29	0.24	0.33	0.46	0.17	0.38	0.64	0.27	0.24	0.33	0.18	0.31	0.40	0.27
GH59	0.00	0.00	0.00	0.00	0.00	0.00	0.04	0.00	0.00	0.00	0.04	0.01	0.00	0.00	0.00	0.01
GH35	0.06	0.41	0.37	0.31	0.15	0.28	0.40	0.27	0.25	0.46	0.21	0.18	0.40	0.42	0.29	0.23
GT39	0.02	0.00	0.00	0.00	0.00	0.00	0.00	0.02	0.00	0.00	0.00	0.00	0.00	0.00	0.00	0.01
GT57	0.00	0.00	0.00	0.00	0.00	0.00	0.00	0.00	0.00	0.00	0.00	0.00	0.00	0.00	0.00	0.00
GH129	0.00	0.02	0.00	0.00	0.00	0.00	0.00	0.00	0.00	0.00	0.00	0.00	0.00	0.00	0.00	0.00
GT22	0.00	0.00	0.00	0.00	0.00	0.00	0.00	0.00	0.00	0.00	0.00	0.00	0.00	0.00	0.00	0.00
PL12	0.00	0.22	0.13	0.09	0.09	0.00	0.08	0.04	0.00	0.07	0.14	0.05	0.13	0.00	0.00	0.15
PL18	0.00	0.00	0.00	0.00	0.00	0.00	0.00	0.00	0.00	0.00	0.00	0.00	0.00	0.00	0.00	0.00
GT92	0.00	0.00	0.00	0.00	0.00	0.00	0.00	0.00	0.00	0.00	0.00	0.00	0.00	0.00	0.00	0.00
GT44	0.00	0.00	0.00	0.00	0.00	0.00	0.00	0.00	0.00	0.00	0.00	0.00	0.00	0.00	0.00	0.00
GT29	0.00	0.00	0.00	0.00	0.00	0.00	0.00	0.00	0.00	0.00	0.00	0.00	0.00	0.00	0.00	0.00
GT13	0.00	0.00	0.00	0.00	0.00	0.00	0.00	0.00	0.00	0.00	0.00	0.00	0.00	0.00	0.00	0.00

CAZY_group	CN-AD-87	CN-AD-88	CN-AD-89	CN-AD-90	CN-AD-91	CN-AD-92	CN-AD-93	CN-AD-94	CN-AD-95	CN-AD-96	CN-AD-97	CN-AD-98	CN-AD-99	CN-AD-100	CN-AD-101	CN-AD-102
**GT52**	0.00	0.00	0.00	0.00	0.00	0.00	0.00	0.00	0.00	0.00	0.00	0.00	0.00	0.00	0.00	0.00
**GT64**	0.00	0.00	0.00	0.00	0.00	0.00	0.00	0.00	0.00	0.00	0.00	0.00	0.00	0.00	0.00	0.00
**GT85**	0.00	0.00	0.00	0.00	0.00	0.00	0.00	0.00	0.00	0.00	0.00	0.00	0.00	0.00	0.00	0.00
**GT16**	0.00	0.00	0.00	0.00	0.00	0.00	0.00	0.00	0.00	0.00	0.00	0.00	0.00	0.00	0.00	0.00
**GH56**	0.00	0.00	0.00	0.00	0.00	0.00	0.00	0.00	0.00	0.00	0.00	0.00	0.00	0.00	0.00	0.00
**GT60**	0.00	0.00	0.00	0.00	0.00	0.00	0.00	0.00	0.00	0.00	0.00	0.00	0.00	0.00	0.00	0.00
**GH41**	0.00	0.00	0.00	0.00	0.00	0.00	0.00	0.00	0.00	0.00	0.00	0.00	0.00	0.00	0.00	0.00
**CE7**	0.08	0.19	0.12	0.07	0.15	0.07	0.09	0.06	0.15	0.12	0.09	0.08	0.18	0.11	0.06	0.07
**GH39**	0.11	0.12	0.02	0.02	0.02	0.21	0.03	0.04	0.00	0.00	0.04	0.03	0.02	0.00	0.00	0.05
**GT30**	0.19	0.46	0.41	0.27	0.18	0.21	0.24	0.25	0.25	0.32	0.20	0.21	0.31	0.35	0.17	0.17
**PL15**	0.00	0.22	0.15	0.05	0.09	0.00	0.10	0.00	0.10	0.06	0.12	0.17	0.18	0.00	0.00	0.14
**GH102**	0.00	0.07	0.07	0.02	0.00	0.04	0.02	0.06	0.05	0.00	0.01	0.05	0.00	0.07	0.06	0.01

CAZY_group	CN-AD-103	CN-AD-104	CN-AD-105	CN-AD-106	CN-AD-107	CN-AD-108	CN-AD-109	CN-AD-110	CN-AD-111	CN-AD-112	CN-AD-113	CN-AD-114	CN-AD-115	CN-AD-116	CN-AD-117	CN-AD-118
GH29	0.64	0.78	0.55	0.62	0.58	0.86	0.21	0.67	0.69	0.80	0.54	0.57	0.78	0.65	0.58	0.72
PL4	0.00	0.00	0.00	0.01	0.01	0.00	0.00	0.00	0.00	0.00	0.00	0.00	0.00	0.01	0.00	0.00
GH23	0.93	1.05	0.92	1.00	1.07	0.92	0.93	1.01	1.04	0.89	0.76	0.93	0.78	0.98	0.89	1.06
CE6	0.00	0.08	0.01	0.07	0.01	0.04	0.00	0.05	0.02	0.05	0.02	0.04	0.06	0.07	0.13	0.06
GH72	0.07	0.05	0.11	0.06	0.11	0.06	0.08	0.03	0.03	0.07	0.05	0.07	0.04	0.06	0.13	0.09
GH114	0.00	0.00	0.00	0.00	0.00	0.00	0.00	0.00	0.00	0.00	0.00	0.00	0.00	0.00	0.00	0.00
GH78	0.28	0.83	0.50	0.64	0.44	0.65	0.23	0.91	0.77	1.32	0.72	0.58	1.10	1.12	1.04	0.92
PL19	0.00	0.00	0.00	0.00	0.00	0.00	0.00	0.00	0.00	0.00	0.00	0.00	0.00	0.00	0.00	0.00
GT27	0.00	0.00	0.00	0.00	0.00	0.00	0.00	0.00	0.00	0.02	0.00	0.00	0.00	0.00	0.00	0.00
GH5	0.21	0.37	0.42	0.34	0.27	0.39	0.48	0.79	0.12	0.27	0.15	0.46	0.21	0.24	0.40	0.37
GT40	0.00	0.00	0.00	0.00	0.00	0.00	0.00	0.00	0.00	0.00	0.00	0.00	0.00	0.00	0.00	0.00
CE13	0.01	0.00	0.00	0.00	0.00	0.00	0.00	0.00	0.00	0.00	0.00	0.00	0.00	0.00	0.00	0.00
GT37	0.00	0.00	0.00	0.00	0.00	0.00	0.00	0.00	0.00	0.00	0.00	0.00	0.00	0.00	0.00	0.00
GH33	0.12	0.19	0.23	0.25	0.17	0.14	0.08	0.15	0.26	0.27	0.22	0.16	0.38	0.29	0.21	0.14
GT65	0.00	0.00	0.00	0.00	0.00	0.00	0.00	0.00	0.00	0.00	0.00	0.00	0.00	0.00	0.00	0.00
GH86	0.00	0.00	0.00	0.00	0.00	0.00	0.00	0.02	0.00	0.00	0.00	0.01	0.00	0.00	0.00	0.00
GH123	0.00	0.16	0.14	0.11	0.05	0.04	0.02	0.14	0.13	0.15	0.22	0.09	0.13	0.16	0.13	0.11
GH96	0.00	0.00	0.00	0.00	0.00	0.00	0.00	0.00	0.00	0.00	0.00	0.00	0.00	0.00	0.00	0.00
GH14	0.00	0.00	0.00	0.00	0.00	0.00	0.00	0.00	0.00	0.00	0.00	0.00	0.00	0.00	0.00	0.00
CE3	0.00	0.00	0.00	0.00	0.00	0.00	0.00	0.00	0.00	0.00	0.00	0.00	0.00	0.00	0.00	0.00
PL10	0.00	0.18	0.11	0.20	0.08	0.10	0.02	0.22	0.06	0.20	0.07	0.10	0.13	0.14	0.09	0.11
GT48	0.00	0.00	0.00	0.00	0.00	0.00	0.00	0.00	0.00	0.00	0.00	0.00	0.00	0.00	0.00	0.00
PL6	0.00	0.02	0.00	0.00	0.00	0.00	0.00	0.00	0.00	0.00	0.00	0.00	0.00	0.00	0.00	0.00
GT83	0.14	0.06	0.02	0.12	0.12	0.10	0.08	0.07	0.10	0.13	0.12	0.07	0.13	0.09	0.06	0.08
GH126	0.00	0.00	0.00	0.00	0.00	0.00	0.00	0.00	0.00	0.00	0.00	0.00	0.00	0.00	0.00	0.00
GH9	0.01	0.18	0.27	0.35	0.06	0.23	0.06	0.38	0.06	0.23	0.08	0.30	0.13	0.13	0.23	0.23
GH75	0.00	0.00	0.00	0.00	0.00	0.00	0.00	0.00	0.00	0.00	0.00	0.00	0.00	0.00	0.00	0.00
GT71	0.00	0.00	0.00	0.00	0.00	0.00	0.00	0.00	0.00	0.00	0.00	0.00	0.00	0.00	0.00	0.00
GH46	0.00	0.00	0.00	0.00	0.00	0.00	0.00	0.00	0.00	0.00	0.00	0.00	0.00	0.00	0.00	0.00
GT61	0.00	0.00	0.00	0.00	0.00	0.00	0.00	0.00	0.00	0.00	0.00	0.00	0.00	0.00	0.00	0.00
GT3	0.00	0.08	0.09	0.09	0.13	0.06	0.00	0.22	0.10	0.07	0.08	0.16	0.08	0.16	0.11	0.12
PL3	0.00	0.00	0.01	0.00	0.00	0.00	0.00	0.00	0.00	0.00	0.00	0.00	0.00	0.00	0.00	0.00
GH108	0.00	0.03	0.01	0.09	0.04	0.00	0.00	0.03	0.01	0.08	0.02	0.02	0.08	0.05	0.06	0.08
GT56	0.01	0.00	0.00	0.00	0.00	0.00	0.02	0.00	0.00	0.02	0.00	0.00	0.00	0.02	0.00	0.00
PL11	0.01	0.16	0.17	0.31	0.13	0.14	0.04	0.15	0.06	0.25	0.02	0.17	0.32	0.15	0.21	0.06
GH45	0.00	0.00	0.00	0.00	0.00	0.00	0.00	0.00	0.00	0.00	0.00	0.00	0.00	0.01	0.00	0.00
GT34	0.00	0.00	0.00	0.00	0.00	0.00	0.00	0.00	0.00	0.00	0.00	0.00	0.00	0.00	0.00	0.00
PL14	0.00	0.00	0.00	0.00	0.00	0.00	0.00	0.00	0.00	0.00	0.00	0.00	0.00	0.00	0.00	0.00
GH19	0.04	0.00	0.00	0.00	0.00	0.00	0.00	0.02	0.00	0.00	0.00	0.00	0.00	0.00	0.02	0.02
GH118	0.00	0.00	0.00	0.00	0.00	0.00	0.00	0.00	0.00	0.00	0.00	0.00	0.00	0.00	0.00	0.00
GH82	0.00	0.00	0.00	0.00	0.00	0.00	0.00	0.00	0.00	0.00	0.00	0.00	0.00	0.00	0.00	0.00
GH76	0.00	0.22	0.16	0.25	0.00	0.29	0.04	0.12	0.18	0.38	0.12	0.18	0.32	0.24	0.34	0.37
GT4	1.43	1.27	1.45	1.78	1.54	2.21	1.16	1.71	1.71	1.71	1.68	1.51	1.58	1.72	1.64	1.68
GT87	0.00	0.00	0.00	0.00	0.00	0.00	0.00	0.00	0.00	0.00	0.00	0.00	0.00	0.00	0.00	0.00
GH24	0.01	0.13	0.12	0.21	0.15	0.31	0.10	0.24	0.18	0.20	0.20	0.16	0.27	0.15	0.17	0.14
GH90	0.00	0.00	0.00	0.00	0.00	0.00	0.00	0.00	0.00	0.00	0.00	0.00	0.00	0.00	0.00	0.00
GH57	0.00	0.05	0.07	0.09	0.08	0.08	0.02	0.12	0.11	0.08	0.07	0.10	0.10	0.13	0.21	0.15
GH36	0.62	0.75	0.58	0.66	0.66	0.59	0.41	0.70	0.57	0.74	0.51	0.64	0.67	0.57	0.43	0.65
GT46	0.00	0.00	0.00	0.00	0.00	0.00	0.00	0.00	0.00	0.00	0.00	0.00	0.00	0.00	0.00	0.00
GH66	0.07	0.00	0.05	0.06	0.01	0.04	0.00	0.03	0.03	0.08	0.00	0.03	0.00	0.06	0.04	0.03

CAZY_group	CN-AD-103	CN-AD-104	CN-AD-105	CN-AD-106	CN-AD-107	CN-AD-108	CN-AD-109	CN-AD-110	CN-AD-111	CN-AD-112	CN-AD-113	CN-AD-114	CN-AD-115	CN-AD-116	CN-AD-117	CN-AD-118
GH48	0.00	0.00	0.00	0.00	0.00	0.00	0.00	0.00	0.00	0.00	0.00	0.01	0.00	0.00	0.00	0.00
GT53	0.00	0.00	0.00	0.00	0.00	0.00	0.00	0.00	0.00	0.00	0.00	0.00	0.00	0.00	0.00	0.00
GH60	0.00	0.00	0.00	0.00	0.00	0.00	0.00	0.00	0.00	0.00	0.00	0.00	0.00	0.00	0.00	0.00
GT31	0.00	0.00	0.00	0.00	0.00	0.00	0.02	0.00	0.00	0.00	0.00	0.00	0.00	0.00	0.00	0.00
GH105	0.06	0.73	0.58	0.77	0.37	0.51	0.14	0.67	0.32	0.59	0.40	0.59	0.57	0.55	0.66	0.72
GT23	0.00	0.02	0.01	0.00	0.01	0.00	0.00	0.00	0.02	0.00	0.00	0.00	0.02	0.00	0.00	0.00
GH11	0.00	0.00	0.00	0.00	0.00	0.00	0.00	0.00	0.00	0.00	0.00	0.02	0.00	0.00	0.00	0.00
GT38	0.00	0.00	0.00	0.00	0.00	0.00	0.00	0.00	0.00	0.00	0.00	0.00	0.00	0.00	0.00	0.00
GT43	0.00	0.00	0.00	0.00	0.00	0.00	0.00	0.00	0.00	0.00	0.00	0.00	0.00	0.00	0.00	0.00
GH99	0.00	0.00	0.01	0.02	0.00	0.04	0.00	0.00	0.01	0.02	0.02	0.03	0.02	0.02	0.00	0.02
GH38	0.22	0.14	0.15	0.26	0.10	0.14	0.23	0.27	0.18	0.25	0.19	0.19	0.15	0.23	0.30	0.34
GH109	0.00	0.29	0.16	0.26	0.13	0.29	0.02	0.31	0.15	0.27	0.29	0.27	0.17	0.28	0.21	0.28
GH2	1.48	3.55	3.39	3.82	2.92	3.97	2.02	4.22	3.40	5.05	3.37	3.49	4.46	3.98	4.84	4.68
GT28	0.53	0.43	0.34	0.31	0.47	0.25	0.27	0.27	0.38	0.23	0.40	0.33	0.15	0.27	0.23	0.32
GH42	0.40	0.27	0.28	0.20	0.23	0.06	0.21	0.17	0.17	0.15	0.10	0.12	0.13	0.06	0.11	0.17
GT58	0.00	0.00	0.00	0.00	0.00	0.00	0.00	0.00	0.00	0.00	0.00	0.00	0.00	0.00	0.00	0.00
GH61	0.00	0.00	0.00	0.00	0.00	0.00	0.00	0.00	0.00	0.00	0.00	0.00	0.00	0.00	0.00	0.00
GH80	0.00	0.00	0.00	0.00	0.00	0.00	0.00	0.00	0.00	0.00	0.00	0.00	0.00	0.00	0.00	0.00
GH98	0.00	0.00	0.00	0.00	0.01	0.00	0.00	0.02	0.00	0.02	0.02	0.02	0.02	0.01	0.02	0.02
GH6	0.00	0.00	0.00	0.00	0.00	0.00	0.00	0.00	0.00	0.00	0.00	0.00	0.00	0.00	0.00	0.00
GT1	0.17	0.03	0.05	0.02	0.03	0.02	0.04	0.02	0.02	0.02	0.03	0.02	0.02	0.02	0.06	0.03
GH28	0.17	0.78	0.79	0.87	0.52	0.68	0.27	1.01	0.55	0.85	0.47	0.62	1.14	0.76	0.98	0.92
GT42	0.00	0.00	0.00	0.00	0.00	0.00	0.00	0.00	0.00	0.00	0.00	0.00	0.00	0.00	0.00	0.00
GH128	0.00	0.00	0.00	0.00	0.00	0.00	0.00	0.00	0.00	0.00	0.00	0.00	0.00	0.00	0.00	0.00
CE12	0.00	0.19	0.16	0.17	0.10	0.18	0.04	0.15	0.10	0.18	0.03	0.12	0.25	0.20	0.11	0.17
CE16	0.00	0.00	0.00	0.00	0.00	0.00	0.00	0.00	0.00	0.00	0.00	0.00	0.00	0.00	0.00	0.00
GT89	0.00	0.00	0.00	0.00	0.00	0.00	0.00	0.00	0.00	0.00	0.00	0.00	0.00	0.00	0.00	0.00
GT11	0.00	0.08	0.05	0.07	0.06	0.10	0.00	0.02	0.07	0.03	0.07	0.05	0.06	0.06	0.02	0.05
GT77	0.00	0.03	0.00	0.01	0.00	0.02	0.00	0.02	0.01	0.00	0.02	0.00	0.02	0.00	0.02	0.00
GT55	0.00	0.00	0.00	0.00	0.00	0.00	0.00	0.00	0.00	0.00	0.00	0.00	0.00	0.00	0.00	0.00
GT18	0.00	0.00	0.00	0.00	0.00	0.00	0.00	0.00	0.00	0.00	0.00	0.00	0.00	0.00	0.00	0.00
GH87	0.10	0.00	0.00	0.00	0.03	0.00	0.00	0.02	0.01	0.00	0.02	0.00	0.00	0.00	0.00	0.02
PL5	0.00	0.00	0.00	0.00	0.00	0.00	0.00	0.00	0.00	0.00	0.00	0.00	0.00	0.00	0.00	0.00
GH22	0.00	0.00	0.00	0.00	0.00	0.00	0.00	0.00	0.00	0.00	0.00	0.00	0.00	0.00	0.00	0.00
CE2	0.03	0.00	0.05	0.02	0.03	0.08	0.02	0.12	0.06	0.05	0.00	0.05	0.02	0.07	0.08	0.08
GH120	0.12	0.02	0.10	0.04	0.04	0.00	0.10	0.03	0.06	0.00	0.03	0.00	0.00	0.01	0.00	0.11
GH73	0.68	0.43	0.32	0.39	0.36	0.35	0.21	0.41	0.40	0.32	0.24	0.37	0.30	0.41	0.34	0.32
GT17	0.00	0.00	0.00	0.00	0.00	0.00	0.00	0.00	0.00	0.00	0.00	0.00	0.00	0.00	0.00	0.00
GH124	0.00	0.00	0.00	0.00	0.00	0.00	0.00	0.00	0.00	0.00	0.00	0.00	0.00	0.00	0.00	0.00
GH54	0.00	0.00	0.00	0.00	0.00	0.00	0.00	0.00	0.00	0.00	0.00	0.00	0.00	0.00	0.00	0.00
GH100	0.00	0.00	0.00	0.00	0.00	0.00	0.00	0.00	0.00	0.00	0.00	0.00	0.00	0.00	0.00	0.00
GH113	0.01	0.02	0.03	0.01	0.03	0.04	0.04	0.05	0.03	0.00	0.03	0.03	0.00	0.00	0.00	0.00
GT5	0.46	0.45	0.42	0.42	0.40	0.31	0.25	0.31	0.49	0.13	0.34	0.43	0.19	0.24	0.21	0.31
GH94	0.28	0.19	0.40	0.35	0.44	0.23	0.37	0.27	0.19	0.13	0.29	0.31	0.08	0.13	0.13	0.22
GT66	0.00	0.00	0.00	0.00	0.00	0.00	0.00	0.00	0.00	0.00	0.00	0.00	0.00	0.00	0.00	0.00
GT94	0.00	0.00	0.00	0.00	0.00	0.00	0.00	0.00	0.00	0.00	0.00	0.00	0.00	0.00	0.00	0.00
GH58	0.00	0.00	0.00	0.00	0.00	0.00	0.00	0.00	0.00	0.00	0.00	0.00	0.00	0.00	0.00	0.00
GH1	1.48	0.81	0.54	0.52	0.93	0.14	0.54	0.17	0.74	0.20	0.30	0.26	0.11	0.39	0.38	0.57
PL22	0.01	0.00	0.00	0.00	0.00	0.00	0.00	0.00	0.00	0.00	0.00	0.01	0.00	0.00	0.00	0.00
GT9	0.30	0.16	0.09	0.12	0.24	0.18	0.23	0.15	0.07	0.20	0.08	0.06	0.23	0.21	0.06	0.11

CAZY_group	CN-AD-103	CN-AD-104	CN-AD-105	CN-AD-106	CN-AD-107	CN-AD-108	CN-AD-109	CN-AD-110	CN-AD-111	CN-AD-112	CN-AD-113	CN-AD-114	CN-AD-115	CN-AD-116	CN-AD-117	CN-AD-118
GT76	0.00	0.00	0.00	0.00	0.00	0.00	0.00	0.00	0.00	0.00	0.00	0.00	0.00	0.00	0.00	0.00
GT32	0.01	0.08	0.03	0.07	0.08	0.10	0.04	0.05	0.07	0.05	0.13	0.11	0.08	0.07	0.02	0.14
GT47	0.10	0.08	0.02	0.05	0.03	0.04	0.02	0.00	0.02	0.02	0.02	0.01	0.00	0.02	0.09	0.03
GH84	0.17	0.21	0.08	0.10	0.10	0.14	0.06	0.05	0.17	0.12	0.19	0.15	0.11	0.08	0.08	0.14
GH83	0.00	0.00	0.00	0.00	0.00	0.00	0.00	0.00	0.00	0.00	0.00	0.00	0.00	0.00	0.00	0.00
GT59	0.00	0.00	0.00	0.00	0.00	0.00	0.00	0.00	0.00	0.00	0.00	0.00	0.00	0.00	0.00	0.00
GH34	0.00	0.00	0.00	0.00	0.00	0.00	0.00	0.00	0.00	0.00	0.00	0.00	0.00	0.00	0.00	0.00
GT14	0.00	0.00	0.00	0.06	0.03	0.10	0.00	0.02	0.03	0.03	0.03	0.01	0.04	0.03	0.06	0.02
GH47	0.00	0.00	0.00	0.00	0.00	0.00	0.00	0.00	0.00	0.00	0.00	0.00	0.00	0.01	0.00	0.00
GH97	0.00	0.88	0.59	0.90	0.26	1.00	0.19	1.01	0.48	1.25	0.44	0.75	0.97	1.31	0.96	1.25
GH50	0.00	0.03	0.05	0.02	0.03	0.00	0.00	0.07	0.02	0.05	0.00	0.03	0.11	0.02	0.06	0.05
GT26	0.24	0.18	0.09	0.16	0.17	0.16	0.14	0.10	0.12	0.15	0.17	0.17	0.02	0.16	0.15	0.14
GH18	0.43	0.53	0.42	0.52	0.39	0.61	0.39	0.60	0.64	0.52	0.30	0.62	0.61	0.55	0.66	0.75
GH37	0.03	0.00	0.00	0.00	0.00	0.00	0.06	0.00	0.00	0.08	0.00	0.00	0.00	0.01	0.00	0.00
PL13	0.00	0.00	0.03	0.05	0.01	0.06	0.00	0.03	0.01	0.12	0.00	0.03	0.06	0.03	0.06	0.02
GH27	0.03	0.21	0.17	0.25	0.14	0.23	0.06	0.27	0.22	0.22	0.19	0.26	0.23	0.30	0.36	0.20
GT54	0.00	0.00	0.00	0.00	0.00	0.00	0.00	0.00	0.00	0.00	0.00	0.00	0.00	0.00	0.00	0.00
GT91	0.00	0.00	0.00	0.00	0.00	0.00	0.00	0.00	0.00	0.00	0.00	0.00	0.00	0.00	0.00	0.00
GT72	0.00	0.00	0.00	0.00	0.00	0.00	0.00	0.00	0.00	0.00	0.00	0.00	0.00	0.00	0.00	0.00
GH67	0.00	0.11	0.07	0.05	0.02	0.04	0.04	0.12	0.02	0.10	0.00	0.07	0.10	0.06	0.09	0.08
GH12	0.00	0.00	0.00	0.00	0.00	0.00	0.00	0.00	0.00	0.00	0.00	0.00	0.00	0.00	0.00	0.00
GH91	0.03	0.08	0.00	0.00	0.01	0.06	0.00	0.02	0.03	0.03	0.05	0.04	0.02	0.01	0.02	0.00
GT69	0.00	0.00	0.00	0.00	0.00	0.00	0.00	0.00	0.00	0.00	0.00	0.00	0.00	0.00	0.00	0.00
GH106	0.00	0.18	0.18	0.20	0.18	0.20	0.02	0.29	0.20	0.40	0.15	0.16	0.46	0.27	0.25	0.18
GT62	0.00	0.00	0.00	0.00	0.00	0.00	0.00	0.00	0.00	0.00	0.00	0.00	0.00	0.00	0.00	0.00
GH17	0.00	0.00	0.00	0.00	0.00	0.00	0.00	0.00	0.00	0.00	0.00	0.00	0.00	0.00	0.00	0.00
GH88	0.15	0.29	0.24	0.32	0.11	0.25	0.00	0.63	0.26	0.60	0.29	0.41	0.32	0.31	0.64	0.51
CE5	0.00	0.00	0.00	0.00	0.00	0.00	0.00	0.00	0.00	0.00	0.00	0.00	0.00	0.00	0.00	0.00
GT8	0.29	0.05	0.06	0.05	0.19	0.08	0.14	0.03	0.10	0.07	0.05	0.04	0.10	0.07	0.08	0.05
GT50	0.00	0.00	0.00	0.00	0.00	0.00	0.00	0.00	0.00	0.00	0.00	0.00	0.00	0.00	0.00	0.00
GT20	0.01	0.08	0.03	0.05	0.02	0.12	0.04	0.07	0.04	0.17	0.13	0.06	0.11	0.13	0.06	0.08
PL9	0.00	0.13	0.05	0.16	0.10	0.16	0.04	0.05	0.07	0.05	0.10	0.08	0.08	0.12	0.08	0.20
GH55	0.00	0.03	0.01	0.01	0.03	0.04	0.04	0.05	0.00	0.03	0.05	0.01	0.02	0.01	0.02	0.02
GH64	0.00	0.00	0.00	0.00	0.00	0.00	0.00	0.00	0.00	0.00	0.02	0.00	0.00	0.00	0.00	0.00
GH127	0.12	0.21	0.25	0.17	0.18	0.23	0.12	0.21	0.28	0.28	0.20	0.15	0.17	0.36	0.28	0.29
GH103	0.06	0.00	0.00	0.00	0.01	0.00	0.04	0.00	0.00	0.02	0.00	0.00	0.00	0.02	0.00	0.00
GH3	1.66	2.69	2.09	2.58	1.72	2.09	1.67	3.23	1.92	3.06	2.19	1.87	2.23	2.59	2.41	2.77
GT45	0.00	0.00	0.00	0.00	0.00	0.00	0.00	0.00	0.00	0.00	0.00	0.00	0.00	0.00	0.00	0.00
GT74	0.00	0.00	0.00	0.00	0.00	0.00	0.00	0.00	0.00	0.00	0.00	0.00	0.00	0.00	0.00	0.00
GT90	0.00	0.00	0.00	0.00	0.00	0.00	0.00	0.00	0.00	0.00	0.02	0.00	0.00	0.00	0.00	0.00
GH116	0.00	0.03	0.03	0.09	0.03	0.12	0.00	0.03	0.03	0.05	0.10	0.11	0.04	0.07	0.11	0.12
GH31	0.48	1.23	1.01	1.12	0.64	0.92	0.91	0.91	0.84	1.05	0.79	0.84	0.99	1.02	1.13	1.09
GT12	0.00	0.00	0.00	0.00	0.00	0.00	0.00	0.00	0.00	0.00	0.00	0.00	0.00	0.00	0.00	0.00
CE1	0.22	0.32	0.26	0.37	0.24	0.41	0.21	0.38	0.25	0.48	0.24	0.28	0.34	0.37	0.38	0.45
GH121	0.03	0.02	0.00	0.00	0.02	0.00	0.00	0.00	0.00	0.00	0.00	0.00	0.00	0.00	0.00	0.00
GH79	0.00	0.00	0.00	0.01	0.01	0.00	0.04	0.00	0.00	0.00	0.02	0.00	0.00	0.00	0.00	0.00
GT73	0.00	0.00	0.00	0.00	0.00	0.00	0.00	0.00	0.00	0.00	0.00	0.00	0.00	0.00	0.00	0.00
GH77	0.55	0.62	0.55	0.52	0.71	0.23	0.50	0.32	0.57	0.33	0.64	0.55	0.30	0.43	0.43	0.31
PL17	0.00	0.02	0.01	0.01	0.01	0.00	0.00	0.02	0.00	0.00	0.00	0.00	0.04	0.01	0.00	0.00
PL8	0.06	0.40	0.15	0.46	0.14	0.41	0.08	0.48	0.23	0.37	0.34	0.38	0.30	0.37	0.34	0.63

CAZY_group	CN-AD-103	CN-AD-104	CN-AD-105	CN-AD-106	CN-AD-107	CN-AD-108	CN-AD-109	CN-AD-110	CN-AD-111	CN-AD-112	CN-AD-113	CN-AD-114	CN-AD-115	CN-AD-116	CN-AD-117	CN-AD-118
CE9	0.50	0.40	0.31	0.32	0.39	0.23	0.29	0.38	0.39	0.35	0.44	0.28	0.36	0.26	0.21	0.25
CE15	0.00	0.00	0.01	0.02	0.00	0.02	0.00	0.00	0.01	0.00	0.02	0.02	0.00	0.02	0.02	0.03
GT79	0.00	0.00	0.00	0.00	0.00	0.00	0.00	0.00	0.00	0.00	0.00	0.00	0.00	0.00	0.00	0.00
GT10	0.00	0.00	0.00	0.00	0.00	0.00	0.00	0.02	0.01	0.02	0.02	0.00	0.00	0.01	0.02	0.02
GH119	0.00	0.00	0.00	0.00	0.00	0.00	0.00	0.00	0.00	0.00	0.00	0.00	0.00	0.00	0.00	0.00
GH16	0.04	0.29	0.14	0.22	0.16	0.31	0.06	0.27	0.22	0.37	0.32	0.10	0.27	0.24	0.17	0.34
GH43	0.55	1.91	1.64	2.27	0.83	2.35	0.64	2.34	1.43	2.79	1.36	1.97	2.55	2.22	2.83	3.12
GH51	0.22	0.57	0.38	0.55	0.30	0.47	0.35	0.63	0.53	0.59	0.61	0.36	0.61	0.59	0.38	0.48
GT35	0.83	0.89	0.77	0.75	1.08	0.43	0.87	0.63	0.77	0.48	0.88	0.84	0.32	0.49	0.57	0.85
GT25	0.06	0.00	0.00	0.00	0.01	0.02	0.00	0.00	0.00	0.00	0.00	0.01	0.00	0.00	0.00	0.00
GT2	2.99	3.73	3.07	3.67	3.64	3.97	2.91	3.16	3.73	3.78	3.74	3.32	3.37	3.39	3.81	4.12
GT51	1.09	0.81	0.63	0.56	0.78	0.72	0.76	0.67	0.83	0.89	0.64	0.67	0.72	0.86	0.58	0.68
GH107	0.00	0.00	0.00	0.00	0.00	0.00	0.00	0.00	0.00	0.00	0.00	0.00	0.00	0.00	0.00	0.00
GH7	0.00	0.00	0.00	0.00	0.00	0.00	0.00	0.00	0.00	0.00	0.00	0.00	0.00	0.00	0.00	0.00
GT7	0.00	0.00	0.00	0.00	0.00	0.00	0.00	0.00	0.00	0.00	0.00	0.00	0.00	0.00	0.00	0.00
GT36	0.00	0.00	0.00	0.00	0.00	0.00	0.00	0.00	0.00	0.00	0.00	0.00	0.00	0.00	0.00	0.00
PL7	0.00	0.00	0.00	0.00	0.00	0.00	0.00	0.00	0.00	0.00	0.00	0.00	0.00	0.00	0.00	0.00
GH110	0.00	0.11	0.08	0.07	0.06	0.18	0.02	0.07	0.16	0.13	0.15	0.08	0.17	0.14	0.11	0.15
GT21	0.00	0.00	0.00	0.00	0.00	0.00	0.00	0.00	0.00	0.00	0.00	0.00	0.00	0.00	0.00	0.00
GH62	0.00	0.00	0.00	0.00	0.00	0.00	0.00	0.00	0.00	0.00	0.00	0.00	0.00	0.00	0.00	0.00
GH125	0.11	0.18	0.06	0.20	0.02	0.23	0.02	0.21	0.13	0.17	0.12	0.14	0.15	0.19	0.13	0.23
GH117	0.00	0.08	0.07	0.14	0.10	0.10	0.02	0.10	0.09	0.13	0.15	0.06	0.08	0.10	0.06	0.11
GH68	0.03	0.00	0.00	0.00	0.02	0.00	0.00	0.00	0.00	0.00	0.00	0.00	0.00	0.00	0.00	0.00
GH85	0.11	0.00	0.02	0.04	0.00	0.00	0.00	0.07	0.02	0.00	0.00	0.01	0.00	0.01	0.00	0.02
GH92	0.07	1.34	0.92	1.78	0.67	1.90	0.31	1.73	1.27	2.14	1.52	1.21	2.38	2.14	2.13	2.14
GH40	0.00	0.00	0.00	0.00	0.00	0.00	0.00	0.00	0.00	0.00	0.00	0.00	0.00	0.00	0.00	0.00
GT49	0.00	0.00	0.00	0.00	0.00	0.00	0.00	0.00	0.00	0.00	0.00	0.00	0.00	0.00	0.00	0.00
GH112	0.25	0.21	0.21	0.21	0.27	0.06	0.12	0.07	0.20	0.03	0.17	0.12	0.06	0.07	0.13	0.15
GT67	0.00	0.00	0.00	0.00	0.00	0.00	0.00	0.00	0.00	0.00	0.00	0.00	0.00	0.00	0.00	0.00
CE8	0.03	0.29	0.19	0.19	0.15	0.20	0.08	0.22	0.17	0.23	0.13	0.14	0.29	0.21	0.21	0.22
GT86	0.00	0.00	0.00	0.00	0.00	0.00	0.00	0.00	0.00	0.00	0.00	0.00	0.00	0.00	0.00	0.00
GH101	0.10	0.00	0.00	0.01	0.01	0.00	0.02	0.00	0.06	0.00	0.02	0.03	0.00	0.00	0.00	0.02
GH81	0.00	0.00	0.00	0.00	0.01	0.00	0.00	0.00	0.00	0.00	0.05	0.00	0.00	0.00	0.00	0.00
GT93	0.00	0.00	0.00	0.00	0.00	0.00	0.00	0.00	0.00	0.00	0.00	0.00	0.00	0.00	0.00	0.00
GT63	0.00	0.00	0.00	0.00	0.00	0.00	0.00	0.00	0.00	0.00	0.00	0.00	0.00	0.00	0.00	0.00
CE11	0.18	0.16	0.14	0.17	0.24	0.27	0.14	0.14	0.19	0.22	0.20	0.14	0.27	0.31	0.25	0.25
GT19	0.10	0.11	0.10	0.11	0.14	0.18	0.06	0.10	0.12	0.13	0.10	0.09	0.15	0.17	0.15	0.14
GT33	0.00	0.00	0.00	0.00	0.00	0.00	0.00	0.00	0.00	0.00	0.00	0.00	0.00	0.00	0.00	0.00
GH21	0.00	0.00	0.00	0.00	0.00	0.00	0.00	0.00	0.00	0.00	0.00	0.00	0.00	0.00	0.00	0.00
GT68	0.00	0.00	0.00	0.00	0.00	0.00	0.00	0.00	0.00	0.00	0.00	0.00	0.00	0.00	0.00	0.00
GH89	0.00	0.24	0.20	0.25	0.07	0.18	0.02	0.27	0.19	0.23	0.24	0.18	0.44	0.21	0.34	0.28
GT82	0.00	0.00	0.00	0.00	0.01	0.00	0.00	0.00	0.00	0.00	0.00	0.00	0.00	0.00	0.00	0.00
GH30	0.06	0.18	0.12	0.30	0.07	0.27	0.08	0.36	0.10	0.37	0.15	0.22	0.27	0.33	0.32	0.37
GH20	0.39	0.96	0.66	1.09	0.71	1.21	0.31	1.08	1.04	1.19	1.15	0.92	1.31	1.18	0.96	1.25
GH52	0.00	0.00	0.00	0.00	0.00	0.00	0.00	0.00	0.00	0.00	0.00	0.00	0.00	0.00	0.00	0.00
GT84	0.00	0.00	0.01	0.00	0.02	0.00	0.00	0.00	0.00	0.00	0.03	0.01	0.00	0.00	0.00	0.00
GT15	0.00	0.00	0.00	0.00	0.00	0.00	0.00	0.00	0.00	0.00	0.00	0.00	0.00	0.00	0.00	0.00
GH93	0.00	0.00	0.00	0.00	0.00	0.02	0.00	0.00	0.01	0.02	0.00	0.00	0.02	0.00	0.02	0.00
PL16	0.00	0.00	0.00	0.00	0.00	0.00	0.00	0.00	0.00	0.00	0.00	0.00	0.00	0.01	0.00	0.00
GH26	0.01	0.02	0.16	0.06	0.05	0.08	0.06	0.24	0.06	0.15	0.07	0.11	0.02	0.10	0.06	0.06

CAZY_group	CN-AD-103	CN-AD-104	CN-AD-105	CN-AD-106	CN-AD-107	CN-AD-108	CN-AD-109	CN-AD-110	CN-AD-111	CN-AD-112	CN-AD-113	CN-AD-114	CN-AD-115	CN-AD-116	CN-AD-117	CN-AD-118
GT75	0.00	0.00	0.00	0.00	0.00	0.00	0.00	0.00	0.00	0.00	0.00	0.00	0.00	0.00	0.00	0.00
GH53	0.07	0.05	0.16	0.09	0.16	0.16	0.06	0.10	0.03	0.08	0.10	0.03	0.10	0.07	0.06	0.06
GT88	0.00	0.00	0.00	0.00	0.00	0.00	0.00	0.00	0.00	0.00	0.00	0.00	0.00	0.00	0.00	0.00
GT24	0.00	0.00	0.00	0.00	0.00	0.00	0.00	0.00	0.00	0.00	0.00	0.00	0.00	0.00	0.00	0.00
GT81	0.00	0.00	0.00	0.00	0.00	0.00	0.00	0.00	0.00	0.00	0.00	0.00	0.00	0.00	0.00	0.00
GH4	0.26	0.18	0.08	0.12	0.15	0.10	0.29	0.00	0.13	0.15	0.12	0.09	0.08	0.12	0.08	0.11
GT78	0.00	0.00	0.00	0.00	0.00	0.00	0.00	0.00	0.00	0.00	0.00	0.00	0.00	0.00	0.00	0.00
GH8	0.06	0.00	0.05	0.09	0.05	0.02	0.04	0.05	0.01	0.05	0.02	0.07	0.04	0.09	0.06	0.09
GH70	0.37	0.00	0.00	0.00	0.23	0.02	0.00	0.00	0.00	0.00	0.00	0.00	0.00	0.00	0.00	0.00
GH122	0.00	0.00	0.00	0.00	0.00	0.00	0.00	0.00	0.00	0.00	0.00	0.00	0.00	0.00	0.00	0.00
GH74	0.00	0.00	0.09	0.02	0.00	0.00	0.02	0.05	0.00	0.00	0.02	0.02	0.00	0.00	0.00	0.00
GT41	0.01	0.00	0.00	0.01	0.02	0.00	0.00	0.00	0.00	0.02	0.00	0.01	0.00	0.00	0.00	0.03
PL1	0.01	0.33	0.26	0.30	0.14	0.25	0.04	0.22	0.23	0.42	0.19	0.27	0.30	0.28	0.34	0.32
GH15	0.00	0.03	0.02	0.04	0.01	0.02	0.00	0.02	0.02	0.07	0.02	0.05	0.04	0.06	0.00	0.02
GH32	0.84	0.68	0.36	0.32	0.52	0.41	0.29	0.26	0.61	0.40	0.49	0.29	0.46	0.37	0.30	0.45
GH44	0.00	0.00	0.01	0.00	0.00	0.00	0.04	0.02	0.00	0.00	0.00	0.04	0.00	0.00	0.00	0.00
GT6	0.00	0.00	0.00	0.00	0.01	0.00	0.00	0.00	0.00	0.00	0.00	0.00	0.00	0.00	0.00	0.00
GH13	3.36	2.69	2.47	2.21	3.55	2.07	2.19	2.07	2.86	1.72	2.39	2.17	1.31	2.00	1.49	1.75
GT80	0.00	0.02	0.00	0.00	0.00	0.00	0.02	0.02	0.00	0.02	0.00	0.03	0.00	0.03	0.02	0.03
GH71	0.00	0.00	0.00	0.00	0.00	0.00	0.00	0.00	0.00	0.00	0.00	0.00	0.00	0.00	0.00	0.00
GH63	0.03	0.21	0.05	0.09	0.06	0.12	0.04	0.09	0.03	0.12	0.05	0.07	0.11	0.14	0.06	0.11
PL21	0.00	0.03	0.00	0.07	0.05	0.00	0.00	0.03	0.02	0.05	0.07	0.08	0.04	0.06	0.04	0.06
GH65	0.01	0.11	0.09	0.21	0.11	0.08	0.12	0.17	0.13	0.23	0.17	0.09	0.06	0.17	0.09	0.15
CE4	0.65	0.59	0.55	0.45	0.68	0.37	0.35	0.43	0.58	0.47	0.47	0.47	0.34	0.39	0.49	0.31
CE14	0.00	0.02	0.01	0.00	0.04	0.02	0.02	0.00	0.01	0.03	0.00	0.00	0.00	0.01	0.00	0.00
GH115	0.03	0.18	0.25	0.25	0.08	0.10	0.10	0.34	0.13	0.40	0.17	0.26	0.29	0.33	0.34	0.37
GH104	0.01	0.00	0.00	0.00	0.00	0.00	0.00	0.00	0.00	0.00	0.00	0.00	0.00	0.00	0.00	0.00
GH111	0.00	0.00	0.00	0.00	0.00	0.00	0.00	0.00	0.00	0.00	0.00	0.00	0.00	0.00	0.00	0.00
GH95	0.10	0.83	0.44	0.62	0.35	0.47	0.17	0.73	0.49	0.95	0.45	0.45	0.61	0.66	0.66	0.71
GT70	0.00	0.00	0.00	0.00	0.00	0.00	0.00	0.00	0.00	0.00	0.00	0.00	0.00	0.00	0.00	0.00
CE10	0.08	0.10	0.09	0.15	0.06	0.18	0.10	0.15	0.20	0.27	0.10	0.06	0.27	0.26	0.19	0.09
GH10	0.00	0.10	0.17	0.15	0.07	0.02	0.02	0.31	0.10	0.13	0.05	0.14	0.10	0.13	0.13	0.11
GH130	0.08	0.21	0.25	0.32	0.17	0.35	0.27	0.31	0.16	0.38	0.25	0.29	0.34	0.29	0.40	0.45
PL20	0.00	0.00	0.00	0.00	0.00	0.00	0.00	0.00	0.00	0.00	0.00	0.00	0.00	0.00	0.00	0.00
GH69	0.00	0.00	0.00	0.00	0.00	0.00	0.00	0.00	0.00	0.00	0.00	0.00	0.00	0.00	0.00	0.00
PL2	0.00	0.00	0.00	0.00	0.00	0.00	0.00	0.00	0.00	0.00	0.00	0.00	0.00	0.00	0.00	0.00
GH49	0.00	0.00	0.00	0.00	0.00	0.00	0.00	0.00	0.00	0.00	0.00	0.00	0.00	0.00	0.00	0.00
GH25	0.61	0.37	0.36	0.34	0.42	0.45	0.45	0.34	0.39	0.20	0.29	0.33	0.11	0.16	0.23	0.28
GH59	0.00	0.00	0.01	0.00	0.00	0.00	0.04	0.05	0.00	0.00	0.00	0.02	0.00	0.00	0.00	0.02
GH35	0.19	0.30	0.29	0.37	0.12	0.37	0.12	0.60	0.42	0.59	0.51	0.41	0.44	0.37	0.43	0.49
GT39	0.00	0.00	0.00	0.01	0.00	0.00	0.00	0.00	0.00	0.00	0.00	0.00	0.00	0.00	0.00	0.00
GT57	0.00	0.00	0.00	0.00	0.00	0.00	0.00	0.00	0.00	0.00	0.00	0.00	0.00	0.00	0.00	0.00
GH129	0.04	0.00	0.00	0.00	0.02	0.00	0.00	0.00	0.00	0.00	0.00	0.00	0.00	0.00	0.00	0.00
GT22	0.00	0.00	0.00	0.00	0.00	0.00	0.00	0.00	0.00	0.00	0.00	0.00	0.00	0.00	0.00	0.00
PL12	0.03	0.08	0.07	0.21	0.06	0.06	0.00	0.17	0.10	0.13	0.08	0.11	0.19	0.15	0.30	0.15
PL18	0.00	0.00	0.00	0.00	0.00	0.00	0.00	0.00	0.00	0.00	0.00	0.00	0.00	0.00	0.00	0.00
GT92	0.00	0.00	0.00	0.00	0.00	0.00	0.00	0.00	0.00	0.00	0.00	0.00	0.00	0.00	0.00	0.00
GT44	0.00	0.00	0.00	0.01	0.00	0.00	0.00	0.00	0.00	0.00	0.00	0.00	0.00	0.00	0.00	0.00
GT29	0.00	0.00	0.00	0.00	0.00	0.00	0.00	0.00	0.00	0.00	0.00	0.00	0.00	0.00	0.00	0.00
GT13	0.00	0.00	0.00	0.00	0.00	0.00	0.00	0.00	0.00	0.00	0.00	0.00	0.00	0.00	0.00	0.00

Normalized_abundance_2

CAZY_group	CN-AD-103	CN-AD-104	CN-AD-105	CN-AD-106	CN-AD-107	CN-AD-108	CN-AD-109	CN-AD-110	CN-AD-111	CN-AD-112	CN-AD-113	CN-AD-114	CN-AD-115	CN-AD-116	CN-AD-117	CN-AD-118
GT52	0.00	0.00	0.00	0.00	0.00	0.00	0.00	0.00	0.00	0.00	0.00	0.00	0.00	0.00	0.00	0.00
GT64	0.00	0.00	0.00	0.00	0.00	0.00	0.00	0.00	0.00	0.00	0.00	0.00	0.00	0.00	0.00	0.00
GT85	0.00	0.00	0.00	0.00	0.00	0.00	0.00	0.00	0.00	0.00	0.00	0.00	0.00	0.00	0.00	0.00
GT16	0.00	0.00	0.00	0.00	0.00	0.00	0.00	0.00	0.00	0.00	0.00	0.00	0.00	0.00	0.00	0.00
GH56	0.00	0.00	0.00	0.00	0.00	0.00	0.00	0.00	0.00	0.00	0.00	0.00	0.00	0.00	0.00	0.00
GT60	0.00	0.00	0.00	0.00	0.00	0.00	0.00	0.00	0.00	0.00	0.00	0.00	0.00	0.00	0.00	0.00
GH41	0.00	0.00	0.00	0.00	0.00	0.00	0.00	0.00	0.00	0.00	0.00	0.00	0.00	0.00	0.00	0.00
CE7	0.07	0.14	0.12	0.16	0.02	0.14	0.04	0.09	0.07	0.18	0.08	0.05	0.13	0.08	0.19	0.14
GH39	0.08	0.02	0.05	0.04	0.02	0.06	0.04	0.05	0.01	0.03	0.02	0.03	0.00	0.02	0.00	0.02
GT30	0.19	0.18	0.16	0.21	0.27	0.31	0.10	0.22	0.22	0.38	0.25	0.19	0.29	0.33	0.30	0.32
PL15	0.00	0.10	0.11	0.11	0.03	0.04	0.02	0.17	0.08	0.20	0.03	0.09	0.15	0.14	0.21	0.08
GH102	0.07	0.02	0.03	0.01	0.04	0.00	0.10	0.00	0.01	0.03	0.05	0.02	0.02	0.01	0.00	0.00

CAZY_group	CN-AD-119	CN-AD-120	CN-AD-121	CN-AD-122	CN-AD-123	CN-AD-124	CN-AD-125	CN-AD-126	CN-AD-127	CN-AD-128	CN-AD-129	CN-AD-130	CN-AD-131	CN-AD-132	CN-AD-133	CN-AD-134
GH29	0.86	0.55	0.69	0.20	0.33	0.37	0.58	1.23	0.34	0.82	0.90	0.65	0.75	0.87	0.93	0.72
PL4	0.00	0.00	0.00	0.01	0.00	0.00	0.00	0.00	0.00	0.00	0.00	0.00	0.00	0.00	0.01	0.02
GH23	0.87	1.00	1.00	0.92	0.88	1.01	0.97	1.04	0.88	0.87	1.22	0.98	0.86	1.09	1.02	1.15
CE6	0.05	0.04	0.03	0.01	0.04	0.03	0.00	0.02	0.00	0.01	0.08	0.04	0.00	0.02	0.04	0.07
GH72	0.05	0.10	0.12	0.05	0.09	0.10	0.11	0.04	0.06	0.07	0.10	0.09	0.09	0.00	0.06	0.05
GH114	0.00	0.00	0.00	0.00	0.00	0.00	0.00	0.00	0.00	0.00	0.00	0.00	0.00	0.00	0.00	0.00
GH78	1.21	0.66	1.00	0.46	0.59	0.54	0.49	0.83	0.34	0.84	0.76	0.30	0.86	1.08	0.99	0.52
PL19	0.00	0.00	0.00	0.00	0.00	0.00	0.00	0.00	0.00	0.00	0.00	0.00	0.00	0.00	0.00	0.00
GT27	0.00	0.00	0.00	0.00	0.00	0.00	0.00	0.00	0.00	0.00	0.00	0.00	0.00	0.00	0.00	0.00
GH5	0.43	0.63	0.63	0.18	0.13	0.25	0.52	0.19	0.20	0.28	0.44	0.45	0.14	0.11	0.62	0.41
GT40	0.00	0.00	0.00	0.00	0.00	0.00	0.00	0.00	0.00	0.00	0.00	0.00	0.00	0.00	0.00	0.00
CE13	0.00	0.00	0.00	0.00	0.00	0.00	0.00	0.00	0.00	0.00	0.00	0.00	0.00	0.00	0.00	0.00
GT37	0.00	0.00	0.00	0.00	0.00	0.00	0.00	0.00	0.00	0.00	0.00	0.00	0.00	0.00	0.00	0.00
GH33	0.40	0.11	0.24	0.05	0.13	0.10	0.07	0.31	0.14	0.26	0.18	0.13	0.21	0.30	0.31	0.11
GT65	0.00	0.00	0.00	0.00	0.00	0.00	0.00	0.00	0.00	0.00	0.00	0.00	0.00	0.00	0.00	0.00
GH86	0.00	0.00	0.00	0.00	0.00	0.00	0.00	0.00	0.00	0.00	0.00	0.00	0.00	0.00	0.00	0.00
GH123	0.14	0.08	0.03	0.03	0.09	0.03	0.07	0.21	0.02	0.14	0.13	0.06	0.16	0.25	0.13	0.09
GH96	0.00	0.00	0.00	0.00	0.00	0.00	0.00	0.00	0.00	0.00	0.00	0.00	0.00	0.00	0.00	0.00
GH14	0.00	0.00	0.00	0.00	0.00	0.00	0.00	0.00	0.00	0.00	0.00	0.00	0.00	0.00	0.00	0.00
CE3	0.00	0.00	0.00	0.00	0.00	0.00	0.00	0.00	0.00	0.00	0.00	0.00	0.00	0.00	0.00	0.00
PL10	0.16	0.19	0.12	0.03	0.04	0.01	0.13	0.19	0.02	0.07	0.18	0.00	0.12	0.11	0.21	0.14
GT48	0.00	0.00	0.00	0.00	0.00	0.00	0.00	0.00	0.00	0.00	0.00	0.00	0.00	0.00	0.00	0.00
PL6	0.00	0.01	0.00	0.00	0.00	0.00	0.00	0.00	0.00	0.01	0.00	0.00	0.00	0.00	0.00	0.00
GT83	0.12	0.07	0.03	0.05	0.15	0.12	0.02	0.15	0.04	0.13	0.05	0.04	0.07	0.14	0.07	0.09
GH126	0.00	0.00	0.00	0.00	0.00	0.00	0.00	0.00	0.00	0.00	0.00	0.00	0.00	0.00	0.00	0.00
GH9	0.22	0.36	0.21	0.04	0.06	0.01	0.19	0.10	0.01	0.20	0.30	0.30	0.21	0.07	0.08	0.11
GH75	0.00	0.00	0.00	0.00	0.00	0.00	0.00	0.00	0.00	0.00	0.00	0.00	0.00	0.00	0.00	0.00
GT71	0.00	0.00	0.00	0.00	0.00	0.00	0.00	0.00	0.00	0.00	0.00	0.00	0.00	0.00	0.00	0.00
GH46	0.00	0.00	0.00	0.00	0.00	0.00	0.00	0.00	0.00	0.00	0.00	0.00	0.00	0.00	0.00	0.00
GT61	0.00	0.00	0.00	0.00	0.00	0.00	0.00	0.00	0.00	0.00	0.00	0.00	0.00	0.00	0.00	0.00
GT3	0.15	0.11	0.09	0.01	0.06	0.04	0.09	0.13	0.01	0.11	0.28	0.20	0.07	0.09	0.16	0.14
PL3	0.00	0.00	0.00	0.00	0.00	0.00	0.02	0.00	0.00	0.00	0.00	0.00	0.00	0.00	0.00	0.00
GH108	0.05	0.03	0.00	0.01	0.02	0.01	0.02	0.02	0.02	0.04	0.03	0.02	0.09	0.04	0.03	0.02
GT56	0.00	0.00	0.00	0.00	0.02	0.03	0.00	0.02	0.00	0.00	0.00	0.00	0.00	0.00	0.00	0.00
PL11	0.24	0.10	0.27	0.07	0.04	0.03	0.15	0.19	0.06	0.13	0.18	0.04	0.17	0.05	0.21	0.29
GH45	0.00	0.00	0.00	0.00	0.00	0.00	0.00	0.00	0.00	0.00	0.00	0.00	0.00	0.00	0.00	0.00
GT34	0.00	0.00	0.00	0.00	0.00	0.00	0.00	0.00	0.00	0.00	0.00	0.00	0.00	0.00	0.00	0.00
PL14	0.00	0.00	0.00	0.00	0.00	0.00	0.00	0.00	0.00	0.00	0.00	0.00	0.00	0.00	0.00	0.00
GH19	0.01	0.01	0.00	0.01	0.00	0.03	0.00	0.00	0.00	0.01	0.00	0.02	0.00	0.00	0.01	0.02
GH118	0.00	0.00	0.00	0.00	0.00	0.00	0.00	0.00	0.00	0.00	0.00	0.00	0.00	0.00	0.00	0.00
GH82	0.00	0.00	0.00	0.00	0.00	0.00	0.00	0.00	0.00	0.00	0.00	0.00	0.00	0.00	0.00	0.00
GH76	0.33	0.27	0.00	0.05	0.06	0.10	0.04	0.33	0.00	0.24	0.25	0.07	0.14	0.21	0.35	0.00
GT4	1.87	1.74	1.66	1.08	1.03	1.59	1.72	2.23	1.49	1.66	2.02	1.19	1.78	1.82	2.05	1.47
GT87	0.00	0.00	0.00	0.00	0.00	0.00	0.00	0.00	0.01	0.00	0.00	0.00	0.00	0.02	0.00	0.00
GH24	0.12	0.10	0.09	0.12	0.17	0.21	0.17	0.25	0.02	0.10	0.21	0.15	0.31	0.25	0.13	0.16
GH90	0.00	0.00	0.00	0.00	0.00	0.06	0.00	0.00	0.00	0.00	0.00	0.00	0.00	0.00	0.00	0.00
GH57	0.14	0.12	0.06	0.04	0.06	0.07	0.13	0.06	0.01	0.11	0.16	0.13	0.05	0.09	0.11	0.09
GH36	0.61	0.62	0.81	0.62	0.37	0.57	0.67	0.65	0.64	0.58	0.71	0.74	0.40	0.44	1.03	0.88
GT46	0.00	0.00	0.00	0.00	0.00	0.00	0.00	0.00	0.00	0.00	0.00	0.00	0.00	0.00	0.00	0.00
GH66	0.06	0.01	0.12	0.01	0.00	0.01	0.02	0.08	0.02	0.04	0.05	0.02	0.00	0.02	0.06	0.00

CAZY_group	CN-AD-119	CN-AD-120	CN-AD-121	CN-AD-122	CN-AD-123	CN-AD-124	CN-AD-125	CN-AD-126	CN-AD-127	CN-AD-128	CN-AD-129	CN-AD-130	CN-AD-131	CN-AD-132	CN-AD-133	CN-AD-134
GH48	0.00	0.00	0.03	0.00	0.00	0.00	0.00	0.02	0.00	0.00	0.00	0.00	0.00	0.00	0.00	0.00
GT53	0.00	0.00	0.00	0.00	0.00	0.00	0.00	0.00	0.00	0.00	0.00	0.00	0.00	0.00	0.00	0.00
GH60	0.00	0.00	0.00	0.00	0.00	0.00	0.00	0.00	0.00	0.00	0.00	0.00	0.00	0.00	0.00	0.00
GT31	0.00	0.00	0.00	0.00	0.02	0.01	0.00	0.00	0.00	0.00	0.00	0.00	0.00	0.00	0.00	0.00
GH105	0.82	0.38	0.39	0.17	0.18	0.32	0.43	0.73	0.26	0.54	0.84	0.33	0.82	0.55	1.05	0.47
GT23	0.00	0.00	0.03	0.00	0.00	0.00	0.02	0.00	0.01	0.00	0.02	0.00	0.03	0.00	0.01	0.02
GH11	0.00	0.00	0.00	0.00	0.00	0.00	0.00	0.00	0.00	0.00	0.00	0.00	0.00	0.00	0.00	0.00
GT38	0.00	0.00	0.00	0.00	0.00	0.00	0.00	0.00	0.00	0.00	0.00	0.00	0.00	0.00	0.00	0.00
GT43	0.00	0.00	0.00	0.00	0.00	0.00	0.00	0.00	0.00	0.00	0.00	0.00	0.00	0.00	0.00	0.00
GH99	0.00	0.00	0.00	0.00	0.00	0.01	0.00	0.00	0.00	0.03	0.02	0.00	0.00	0.02	0.01	0.00
GH38	0.30	0.26	0.21	0.13	0.15	0.21	0.09	0.27	0.06	0.16	0.23	0.11	0.17	0.21	0.24	0.14
GH109	0.22	0.18	0.21	0.07	0.09	0.07	0.32	0.37	0.09	0.30	0.26	0.22	0.24	0.25	0.35	0.18
GH2	5.47	3.38	3.59	2.09	2.64	2.70	2.83	4.87	1.80	4.25	4.29	2.69	3.56	4.55	4.80	3.66
GT28	0.32	0.30	0.39	0.34	0.26	0.44	0.34	0.31	0.43	0.27	0.35	0.35	0.37	0.26	0.35	0.52
GH42	0.16	0.12	0.12	0.28	0.09	0.54	0.22	0.00	0.26	0.09	0.16	0.09	0.05	0.07	0.14	0.32
GT58	0.00	0.00	0.00	0.00	0.00	0.00	0.00	0.00	0.00	0.00	0.00	0.00	0.00	0.00	0.00	0.00
GH61	0.00	0.00	0.00	0.00	0.00	0.00	0.00	0.00	0.00	0.00	0.00	0.00	0.00	0.00	0.00	0.00
GH80	0.00	0.00	0.00	0.00	0.00	0.00	0.00	0.00	0.00	0.00	0.00	0.00	0.00	0.00	0.00	0.00
GH98	0.01	0.00	0.00	0.00	0.00	0.00	0.00	0.00	0.00	0.00	0.03	0.02	0.03	0.00	0.00	0.00
GH6	0.00	0.00	0.00	0.00	0.00	0.00	0.00	0.00	0.00	0.00	0.00	0.00	0.00	0.00	0.00	0.00
GT1	0.00	0.04	0.03	0.01	0.02	0.07	0.02	0.02	0.07	0.00	0.00	0.00	0.03	0.02	0.01	0.02
GH28	1.09	0.66	0.72	0.24	0.42	0.57	0.51	0.71	0.50	0.75	1.23	0.45	0.87	0.69	0.98	0.72
GT42	0.00	0.00	0.00	0.00	0.00	0.00	0.00	0.00	0.00	0.00	0.00	0.00	0.00	0.00	0.00	0.00
GH128	0.00	0.00	0.00	0.00	0.00	0.00	0.00	0.00	0.00	0.00	0.00	0.00	0.00	0.00	0.00	0.00
CE12	0.19	0.12	0.15	0.05	0.06	0.12	0.13	0.10	0.14	0.11	0.21	0.13	0.10	0.07	0.13	0.18
CE16	0.00	0.00	0.00	0.00	0.00	0.00	0.00	0.00	0.00	0.00	0.00	0.00	0.00	0.00	0.00	0.00
GT89	0.00	0.00	0.00	0.00	0.00	0.00	0.00	0.00	0.00	0.00	0.00	0.00	0.00	0.00	0.00	0.00
GT11	0.02	0.04	0.06	0.03	0.02	0.06	0.11	0.06	0.03	0.06	0.10	0.02	0.05	0.05	0.10	0.11
GT77	0.01	0.00	0.00	0.00	0.02	0.01	0.00	0.00	0.00	0.01	0.00	0.00	0.02	0.02	0.00	0.02
GT55	0.00	0.00	0.00	0.00	0.00	0.00	0.00	0.00	0.00	0.00	0.00	0.00	0.00	0.00	0.00	0.00
GT18	0.00	0.00	0.00	0.00	0.00	0.00	0.00	0.00	0.00	0.00	0.00	0.00	0.00	0.00	0.00	0.00
GH87	0.02	0.00	0.00	0.00	0.00	0.01	0.00	0.00	0.04	0.00	0.00	0.00	0.00	0.00	0.01	0.00
PL5	0.00	0.00	0.00	0.00	0.00	0.00	0.00	0.00	0.00	0.00	0.00	0.00	0.00	0.00	0.00	0.00
GH22	0.00	0.00	0.00	0.00	0.00	0.00	0.00	0.00	0.00	0.00	0.00	0.00	0.00	0.00	0.00	0.00
CE2	0.07	0.08	0.00	0.04	0.06	0.01	0.02	0.02	0.03	0.03	0.10	0.09	0.02	0.02	0.07	0.05
GH120	0.00	0.05	0.00	0.08	0.02	0.07	0.02	0.00	0.08	0.03	0.08	0.06	0.07	0.00	0.06	0.18
GH73	0.29	0.48	0.57	0.42	0.31	0.40	0.37	0.29	0.53	0.24	0.39	0.48	0.47	0.28	0.44	0.38
GT17	0.00	0.00	0.00	0.00	0.00	0.00	0.02	0.00	0.00	0.00	0.00	0.00	0.00	0.00	0.00	0.00
GH124	0.00	0.00	0.00	0.00	0.00	0.00	0.00	0.00	0.00	0.00	0.00	0.00	0.00	0.00	0.00	0.00
GH54	0.00	0.00	0.00	0.00	0.00	0.00	0.00	0.00	0.00	0.00	0.00	0.00	0.00	0.00	0.00	0.00
GH100	0.00	0.00	0.00	0.00	0.00	0.00	0.00	0.00	0.00	0.00	0.00	0.00	0.00	0.00	0.00	0.00
GH113	0.00	0.07	0.00	0.01	0.02	0.00	0.02	0.00	0.02	0.00	0.03	0.06	0.00	0.00	0.04	0.07
GT5	0.24	0.34	0.48	0.45	0.17	0.44	0.41	0.21	0.57	0.21	0.30	0.28	0.31	0.35	0.35	0.56
GH94	0.26	0.54	0.45	0.40	0.11	0.40	0.60	0.21	0.41	0.30	0.48	0.43	0.33	0.11	0.57	0.68
GT66	0.00	0.00	0.00	0.00	0.00	0.00	0.00	0.00	0.00	0.00	0.00	0.00	0.00	0.00	0.00	0.00
GT94	0.00	0.00	0.00	0.00	0.00	0.00	0.00	0.00	0.00	0.00	0.00	0.00	0.00	0.00	0.00	0.00
GH58	0.00	0.00	0.00	0.00	0.00	0.03	0.00	0.00	0.00	0.00	0.00	0.00	0.00	0.00	0.00	0.00
GH1	0.16	0.27	0.87	0.75	0.42	1.16	0.34	0.52	1.20	0.23	0.38	0.37	0.23	0.32	0.24	0.43
PL22	0.01	0.00	0.00	0.00	0.00	0.03	0.00	0.00	0.00	0.00	0.00	0.00	0.00	0.00	0.00	0.00
GT9	0.15	0.10	0.12	0.04	0.20	0.15	0.07	0.19	0.03	0.13	0.13	0.07	0.23	0.19	0.13	0.14

CAZY_group	CN-AD-119	CN-AD-120	CN-AD-121	CN-AD-122	CN-AD-123	CN-AD-124	CN-AD-125	CN-AD-126	CN-AD-127	CN-AD-128	CN-AD-129	CN-AD-130	CN-AD-131	CN-AD-132	CN-AD-133	CN-AD-134
GT76	0.00	0.00	0.00	0.00	0.00	0.00	0.00	0.00	0.00	0.00	0.00	0.00	0.00	0.00	0.00	0.00
GT32	0.04	0.11	0.00	0.03	0.09	0.07	0.06	0.10	0.04	0.14	0.15	0.09	0.10	0.07	0.07	0.05
GT47	0.00	0.03	0.06	0.05	0.00	0.06	0.02	0.00	0.04	0.01	0.00	0.00	0.00	0.04	0.04	0.02
GH84	0.11	0.05	0.09	0.08	0.09	0.03	0.09	0.19	0.04	0.09	0.15	0.15	0.16	0.18	0.17	0.09
GH83	0.00	0.00	0.00	0.00	0.00	0.00	0.00	0.00	0.00	0.00	0.00	0.00	0.00	0.00	0.00	0.00
GT59	0.00	0.00	0.00	0.00	0.00	0.00	0.00	0.00	0.00	0.00	0.00	0.00	0.00	0.00	0.00	0.00
GH34	0.00	0.00	0.00	0.00	0.00	0.00	0.00	0.00	0.00	0.00	0.00	0.00	0.00	0.00	0.00	0.00
GT14	0.05	0.03	0.00	0.01	0.00	0.00	0.04	0.02	0.01	0.06	0.07	0.02	0.03	0.09	0.04	0.07
GH47	0.00	0.00	0.00	0.00	0.00	0.00	0.00	0.00	0.00	0.00	0.00	0.00	0.00	0.00	0.00	0.00
GH97	1.45	0.96	0.60	0.20	0.63	0.25	0.45	0.87	0.10	0.88	1.22	0.37	0.61	0.90	0.95	0.47
GH50	0.07	0.04	0.00	0.01	0.00	0.03	0.00	0.00	0.00	0.01	0.08	0.00	0.00	0.02	0.06	0.00
GT26	0.17	0.18	0.30	0.11	0.06	0.16	0.22	0.25	0.11	0.17	0.16	0.17	0.14	0.19	0.14	0.23
GH18	0.51	0.44	0.39	0.42	0.52	0.54	0.34	0.58	0.37	0.60	0.44	0.48	0.40	0.56	0.85	0.25
GH37	0.00	0.01	0.00	0.00	0.07	0.06	0.00	0.00	0.00	0.00	0.00	0.00	0.00	0.00	0.00	0.00
PL13	0.11	0.05	0.00	0.01	0.00	0.00	0.00	0.08	0.00	0.06	0.05	0.00	0.02	0.02	0.06	0.00
GH27	0.26	0.21	0.27	0.11	0.17	0.09	0.15	0.29	0.13	0.26	0.18	0.13	0.14	0.28	0.24	0.20
GT54	0.00	0.00	0.00	0.00	0.00	0.00	0.00	0.00	0.00	0.00	0.00	0.00	0.00	0.00	0.00	0.00
GT91	0.00	0.00	0.00	0.00	0.00	0.00	0.00	0.00	0.00	0.00	0.00	0.00	0.00	0.00	0.00	0.00
GT72	0.00	0.00	0.00	0.00	0.00	0.00	0.00	0.00	0.00	0.00	0.00	0.00	0.00	0.00	0.00	0.00
GH67	0.09	0.08	0.12	0.01	0.04	0.03	0.00	0.12	0.02	0.04	0.10	0.06	0.03	0.04	0.13	0.07
GH12	0.00	0.00	0.00	0.00	0.00	0.00	0.00	0.00	0.00	0.00	0.00	0.00	0.00	0.00	0.00	0.00
GH91	0.01	0.04	0.00	0.03	0.02	0.01	0.00	0.00	0.01	0.01	0.00	0.00	0.00	0.05	0.01	0.00
GT69	0.00	0.00	0.00	0.00	0.00	0.00	0.00	0.00	0.00	0.00	0.00	0.00	0.00	0.00	0.00	0.00
GH106	0.36	0.12	0.18	0.07	0.11	0.07	0.11	0.31	0.07	0.16	0.35	0.07	0.24	0.34	0.41	0.18
GT62	0.00	0.00	0.00	0.00	0.00	0.00	0.00	0.00	0.00	0.00	0.00	0.00	0.00	0.00	0.00	0.00
GH17	0.00	0.00	0.00	0.00	0.00	0.00	0.00	0.00	0.00	0.00	0.00	0.00	0.00	0.00	0.00	0.00
GH88	0.63	0.38	0.21	0.22	0.26	0.10	0.21	0.60	0.08	0.45	0.43	0.09	0.37	0.30	0.44	0.23
CE5	0.00	0.00	0.00	0.00	0.00	0.00	0.00	0.00	0.00	0.00	0.00	0.00	0.00	0.00	0.00	0.00
GT8	0.09	0.14	0.12	0.03	0.04	0.15	0.09	0.04	0.14	0.09	0.07	0.09	0.02	0.09	0.07	0.09
GT50	0.00	0.00	0.00	0.00	0.00	0.00	0.00	0.00	0.00	0.00	0.00	0.00	0.00	0.00	0.00	0.00
GT20	0.07	0.05	0.06	0.04	0.07	0.07	0.06	0.08	0.01	0.09	0.08	0.02	0.09	0.07	0.10	0.02
PL9	0.06	0.04	0.06	0.07	0.06	0.23	0.07	0.08	0.10	0.16	0.13	0.06	0.03	0.11	0.13	0.09
GH55	0.05	0.04	0.00	0.04	0.00	0.01	0.06	0.02	0.01	0.01	0.03	0.06	0.02	0.00	0.00	0.07
GH64	0.00	0.00	0.00	0.00	0.00	0.00	0.00	0.00	0.00	0.00	0.00	0.00	0.00	0.00	0.00	0.00
GH127	0.40	0.27	0.21	0.13	0.18	0.13	0.21	0.31	0.14	0.26	0.33	0.20	0.30	0.30	0.35	0.38
GH103	0.00	0.00	0.00	0.00	0.04	0.03	0.00	0.02	0.00	0.01	0.00	0.00	0.00	0.00	0.00	0.00
GH3	3.20	2.58	2.35	1.66	1.62	2.13	2.26	2.87	1.98	2.61	2.47	1.67	1.94	2.35	3.07	2.37
GT45	0.00	0.00	0.00	0.00	0.00	0.00	0.00	0.00	0.00	0.00	0.00	0.00	0.00	0.00	0.00	0.00
GT74	0.00	0.00	0.00	0.00	0.00	0.00	0.00	0.00	0.00	0.00	0.00	0.00	0.00	0.00	0.00	0.00
GT90	0.00	0.00	0.00	0.00	0.00	0.00	0.00	0.00	0.00	0.00	0.00	0.00	0.00	0.00	0.00	0.00
GH116	0.06	0.07	0.00	0.05	0.02	0.03	0.13	0.10	0.01	0.06	0.18	0.04	0.10	0.11	0.03	0.00
GH31	0.99	1.00	0.94	0.62	0.74	0.81	0.95	1.02	0.69	0.91	0.92	0.63	1.03	1.01	1.15	0.93
GT12	0.00	0.00	0.00	0.00	0.00	0.00	0.00	0.00	0.00	0.00	0.00	0.00	0.00	0.00	0.00	0.00
CE1	0.42	0.37	0.21	0.15	0.26	0.19	0.32	0.38	0.17	0.16	0.38	0.19	0.37	0.37	0.27	0.32
GH121	0.00	0.00	0.06	0.01	0.00	0.00	0.00	0.00	0.00	0.00	0.00	0.00	0.00	0.00	0.00	0.02
GH79	0.02	0.00	0.00	0.00	0.00	0.00	0.00	0.00	0.00	0.03	0.02	0.00	0.00	0.00	0.01	0.00
GT73	0.00	0.00	0.00	0.00	0.00	0.03	0.00	0.00	0.00	0.00	0.00	0.00	0.00	0.00	0.00	0.00
GH77	0.47	0.48	0.72	0.48	0.33	0.57	0.49	0.48	0.70	0.48	0.54	0.54	0.40	0.55	0.54	0.52
PL17	0.00	0.01	0.00	0.00	0.02	0.01	0.04	0.02	0.02	0.03	0.02	0.00	0.00	0.00	0.00	0.00
PL8	0.53	0.38	0.15	0.07	0.18	0.13	0.09	0.50	0.02	0.60	0.30	0.11	0.28	0.30	0.41	0.20

CAZY_group	CN-AD-119	CN-AD-120	CN-AD-121	CN-AD-122	CN-AD-123	CN-AD-124	CN-AD-125	CN-AD-126	CN-AD-127	CN-AD-128	CN-AD-129	CN-AD-130	CN-AD-131	CN-AD-132	CN-AD-133	CN-AD-134
CE9	0.27	0.29	0.48	0.25	0.24	0.34	0.22	0.46	0.45	0.33	0.26	0.28	0.35	0.49	0.40	0.32
CE15	0.02	0.00	0.03	0.03	0.04	0.03	0.02	0.00	0.01	0.00	0.00	0.00	0.00	0.00	0.01	0.02
GT79	0.00	0.00	0.00	0.00	0.00	0.00	0.00	0.00	0.00	0.00	0.00	0.00	0.00	0.00	0.00	0.00
GT10	0.01	0.01	0.03	0.00	0.00	0.01	0.04	0.00	0.00	0.00	0.00	0.00	0.00	0.00	0.01	0.00
GH119	0.00	0.00	0.00	0.00	0.00	0.00	0.00	0.00	0.00	0.00	0.00	0.00	0.00	0.00	0.00	0.00
GH16	0.30	0.19	0.12	0.03	0.09	0.12	0.09	0.31	0.06	0.28	0.28	0.15	0.21	0.32	0.35	0.23
GH43	2.58	2.07	2.20	0.91	0.98	1.12	1.48	1.81	0.85	1.96	2.86	1.02	1.69	2.28	2.53	1.92
GH51	0.46	0.49	0.51	0.22	0.35	0.34	0.34	0.46	0.30	0.45	0.56	0.37	0.45	0.49	0.69	0.56
GT35	0.48	0.74	1.15	0.81	0.53	1.15	0.95	0.67	1.09	0.68	0.92	0.87	0.54	0.72	0.84	0.86
GT25	0.00	0.00	0.00	0.00	0.00	0.00	0.00	0.02	0.00	0.00	0.00	0.00	0.00	0.00	0.00	0.00
GT2	3.99	4.15	3.62	2.52	2.78	3.72	3.46	4.41	3.49	3.68	4.18	3.14	3.35	4.06	4.02	3.50
GT51	0.88	0.67	0.66	0.63	0.83	0.78	0.58	0.62	0.78	0.62	0.67	0.56	0.75	0.87	0.86	0.70
GH107	0.00	0.00	0.00	0.00	0.00	0.00	0.00	0.00	0.00	0.00	0.00	0.00	0.00	0.00	0.00	0.00
GH7	0.00	0.00	0.00	0.00	0.00	0.00	0.00	0.00	0.00	0.00	0.00	0.00	0.00	0.00	0.00	0.00
GT7	0.00	0.00	0.00	0.00	0.00	0.00	0.00	0.00	0.00	0.00	0.00	0.00	0.00	0.00	0.00	0.00
GT36	0.00	0.00	0.00	0.00	0.00	0.00	0.00	0.00	0.00	0.00	0.00	0.00	0.00	0.00	0.00	0.00
PL7	0.00	0.00	0.00	0.00	0.00	0.00	0.00	0.00	0.00	0.00	0.00	0.00	0.00	0.00	0.00	0.00
GH110	0.16	0.05	0.06	0.05	0.07	0.03	0.13	0.31	0.04	0.14	0.20	0.07	0.12	0.37	0.18	0.05
GT21	0.00	0.00	0.00	0.00	0.00	0.00	0.00	0.00	0.00	0.00	0.00	0.00	0.00	0.00	0.00	0.00
GH62	0.00	0.00	0.00	0.00	0.00	0.00	0.00	0.00	0.00	0.00	0.00	0.00	0.00	0.00	0.00	0.00
GH125	0.21	0.23	0.03	0.13	0.04	0.09	0.15	0.17	0.01	0.11	0.12	0.04	0.14	0.18	0.23	0.05
GH117	0.11	0.11	0.03	0.04	0.04	0.03	0.09	0.12	0.01	0.09	0.12	0.04	0.09	0.07	0.06	0.07
GH68	0.00	0.00	0.03	0.00	0.00	0.00	0.00	0.00	0.03	0.00	0.00	0.00	0.00	0.00	0.00	0.00
GH85	0.00	0.00	0.00	0.00	0.00	0.00	0.02	0.00	0.00	0.00	0.02	0.00	0.00	0.00	0.04	0.07
GH92	1.76	1.41	0.78	0.45	0.92	0.40	0.82	1.94	0.14	1.58	1.45	0.72	1.34	2.12	2.24	0.63
GH40	0.00	0.00	0.00	0.00	0.00	0.00	0.00	0.00	0.00	0.00	0.00	0.00	0.00	0.00	0.00	0.00
GT49	0.00	0.00	0.00	0.00	0.00	0.00	0.00	0.00	0.00	0.00	0.00	0.00	0.00	0.00	0.00	0.00
GH112	0.07	0.15	0.39	0.15	0.06	0.19	0.26	0.10	0.28	0.11	0.20	0.11	0.07	0.12	0.23	0.25
GT67	0.00	0.00	0.00	0.00	0.00	0.00	0.00	0.00	0.00	0.00	0.00	0.00	0.00	0.00	0.00	0.00
CE8	0.29	0.18	0.33	0.09	0.13	0.32	0.19	0.25	0.15	0.17	0.41	0.15	0.14	0.23	0.34	0.23
GT86	0.00	0.00	0.00	0.00	0.00	0.00	0.00	0.00	0.00	0.00	0.00	0.00	0.00	0.00	0.00	0.00
GH101	0.00	0.00	0.09	0.01	0.00	0.00	0.00	0.00	0.00	0.00	0.00	0.00	0.00	0.00	0.00	0.00
GH81	0.02	0.00	0.00	0.00	0.00	0.00	0.00	0.00	0.00	0.00	0.00	0.00	0.00	0.00	0.00	0.05
GT93	0.00	0.00	0.00	0.00	0.00	0.00	0.00	0.00	0.00	0.00	0.00	0.00	0.00	0.00	0.00	0.00
GT63	0.00	0.00	0.00	0.00	0.00	0.00	0.00	0.00	0.00	0.00	0.00	0.00	0.00	0.00	0.00	0.00
CE11	0.21	0.16	0.15	0.08	0.20	0.10	0.22	0.23	0.10	0.17	0.26	0.19	0.24	0.21	0.27	0.16
GT19	0.21	0.08	0.18	0.04	0.17	0.06	0.09	0.15	0.02	0.17	0.13	0.15	0.17	0.16	0.14	0.11
GT33	0.00	0.00	0.00	0.00	0.00	0.00	0.00	0.00	0.00	0.00	0.00	0.00	0.00	0.00	0.00	0.00
GH21	0.00	0.00	0.00	0.00	0.00	0.00	0.00	0.00	0.00	0.00	0.00	0.00	0.00	0.00	0.00	0.00
GT68	0.00	0.00	0.00	0.00	0.00	0.00	0.00	0.00	0.00	0.00	0.00	0.00	0.00	0.00	0.00	0.00
GH89	0.36	0.12	0.15	0.09	0.09	0.12	0.06	0.44	0.04	0.26	0.33	0.19	0.21	0.32	0.38	0.20
GT82	0.00	0.00	0.00	0.00	0.00	0.00	0.00	0.00	0.00	0.00	0.00	0.00	0.00	0.00	0.00	0.00
GH30	0.36	0.33	0.27	0.09	0.26	0.06	0.15	0.35	0.08	0.33	0.20	0.17	0.17	0.21	0.35	0.09
GH20	1.52	0.69	0.51	0.46	0.63	0.44	0.64	1.67	0.32	1.09	1.17	1.04	1.20	1.55	1.51	0.70
GH52	0.00	0.00	0.00	0.00	0.00	0.00	0.00	0.00	0.00	0.00	0.00	0.00	0.00	0.00	0.00	0.00
GT84	0.00	0.00	0.00	0.00	0.00	0.00	0.06	0.00	0.00	0.00	0.02	0.02	0.00	0.00	0.00	0.00
GT15	0.00	0.00	0.00	0.00	0.00	0.00	0.00	0.00	0.00	0.00	0.00	0.00	0.00	0.00	0.00	0.00
GH93	0.01	0.00	0.00	0.00	0.02	0.00	0.00	0.02	0.00	0.01	0.02	0.00	0.00	0.02	0.00	0.00
PL16	0.00	0.00	0.00	0.00	0.00	0.00	0.00	0.00	0.01	0.00	0.00	0.00	0.00	0.00	0.00	0.00
GH26	0.04	0.18	0.18	0.00	0.06	0.00	0.07	0.06	0.01	0.04	0.16	0.11	0.03	0.09	0.16	0.05

CAZY_group	CN-AD-119	CN-AD-120	CN-AD-121	CN-AD-122	CN-AD-123	CN-AD-124	CN-AD-125	CN-AD-126	CN-AD-127	CN-AD-128	CN-AD-129	CN-AD-130	CN-AD-131	CN-AD-132	CN-AD-133	CN-AD-134
GT75	0.00	0.00	0.00	0.00	0.00	0.00	0.00	0.00	0.00	0.00	0.00	0.00	0.00	0.00	0.00	0.00
GH53	0.07	0.27	0.06	0.07	0.00	0.07	0.13	0.00	0.03	0.01	0.20	0.09	0.05	0.07	0.20	0.25
GT88	0.00	0.00	0.00	0.00	0.00	0.00	0.00	0.00	0.00	0.00	0.00	0.00	0.00	0.00	0.00	0.00
GT24	0.00	0.00	0.00	0.00	0.00	0.00	0.00	0.00	0.00	0.00	0.00	0.00	0.00	0.00	0.00	0.00
GT81	0.00	0.00	0.00	0.00	0.00	0.00	0.00	0.00	0.00	0.00	0.00	0.00	0.00	0.00	0.00	0.00
GH4	0.02	0.05	0.24	0.24	0.24	0.29	0.02	0.10	0.21	0.03	0.07	0.06	0.19	0.04	0.03	0.09
GT78	0.00	0.00	0.00	0.00	0.00	0.00	0.00	0.00	0.00	0.00	0.00	0.00	0.00	0.00	0.00	0.00
GH8	0.02	0.03	0.03	0.01	0.06	0.07	0.07	0.00	0.03	0.00	0.03	0.02	0.00	0.05	0.01	0.07
GH70	0.00	0.00	0.09	0.00	0.00	0.00	0.00	0.00	0.18	0.00	0.00	0.00	0.00	0.00	0.00	0.02
GH122	0.00	0.00	0.00	0.00	0.00	0.00	0.00	0.00	0.00	0.00	0.00	0.00	0.00	0.00	0.00	0.00
GH74	0.00	0.07	0.09	0.01	0.00	0.00	0.06	0.00	0.01	0.00	0.00	0.00	0.00	0.00	0.04	0.02
GT41	0.00	0.00	0.00	0.00	0.02	0.00	0.00	0.00	0.00	0.00	0.02	0.00	0.00	0.02	0.01	0.00
PL1	0.61	0.23	0.27	0.15	0.15	0.25	0.19	0.35	0.10	0.24	0.35	0.20	0.26	0.26	0.54	0.25
GH15	0.02	0.07	0.03	0.01	0.00	0.04	0.06	0.02	0.00	0.01	0.07	0.02	0.03	0.05	0.04	0.02
GH32	0.45	0.36	0.87	0.44	0.20	0.37	0.24	0.37	0.55	0.34	0.48	0.45	0.30	0.44	0.37	0.47
GH44	0.00	0.00	0.00	0.00	0.00	0.00	0.00	0.00	0.00	0.00	0.00	0.00	0.00	0.00	0.01	0.00
GT6	0.00	0.00	0.00	0.00	0.00	0.01	0.04	0.00	0.00	0.00	0.00	0.00	0.00	0.00	0.00	0.00
GH13	1.95	2.73	3.47	2.86	1.62	3.70	2.73	1.89	3.74	2.14	2.60	2.43	2.29	1.99	2.75	3.05
GT80	0.00	0.00	0.00	0.00	0.02	0.03	0.00	0.02	0.00	0.00	0.00	0.02	0.00	0.00	0.00	0.02
GH71	0.00	0.00	0.00	0.00	0.00	0.00	0.00	0.00	0.00	0.00	0.00	0.00	0.00	0.00	0.00	0.00
GH63	0.14	0.15	0.09	0.01	0.09	0.04	0.22	0.08	0.06	0.03	0.08	0.04	0.14	0.05	0.14	0.07
PL21	0.09	0.10	0.00	0.00	0.00	0.00	0.04	0.04	0.00	0.10	0.08	0.00	0.03	0.05	0.03	0.00
GH65	0.26	0.18	0.00	0.12	0.09	0.12	0.07	0.08	0.07	0.10	0.05	0.09	0.09	0.09	0.20	0.11
CE4	0.46	0.66	0.63	0.48	0.31	0.56	0.52	0.37	0.69	0.38	0.53	0.37	0.44	0.28	0.64	0.47
CE14	0.00	0.01	0.00	0.00	0.02	0.01	0.00	0.02	0.00	0.00	0.03	0.00	0.02	0.02	0.00	0.00
GH115	0.38	0.30	0.12	0.05	0.18	0.21	0.07	0.44	0.10	0.21	0.25	0.17	0.12	0.16	0.28	0.25
GH104	0.00	0.00	0.00	0.00	0.06	0.01	0.00	0.00	0.00	0.00	0.00	0.00	0.00	0.00	0.00	0.00
GH111	0.00	0.00	0.00	0.00	0.00	0.00	0.00	0.00	0.00	0.00	0.00	0.00	0.00	0.00	0.00	0.00
GH95	0.73	0.51	0.48	0.16	0.31	0.31	0.30	0.85	0.21	0.62	0.77	0.39	0.56	0.60	0.85	0.36
GT70	0.00	0.00	0.00	0.00	0.00	0.00	0.00	0.00	0.00	0.00	0.00	0.00	0.00	0.00	0.00	0.00
CE10	0.24	0.11	0.12	0.09	0.18	0.15	0.06	0.15	0.06	0.16	0.20	0.07	0.12	0.26	0.20	0.07
GH10	0.09	0.11	0.12	0.08	0.06	0.04	0.02	0.04	0.06	0.06	0.13	0.04	0.03	0.05	0.16	0.14
GH130	0.48	0.33	0.15	0.16	0.17	0.16	0.19	0.38	0.13	0.28	0.43	0.28	0.21	0.25	0.54	0.20
PL20	0.00	0.00	0.00	0.00	0.00	0.00	0.00	0.00	0.00	0.00	0.00	0.00	0.00	0.00	0.00	0.00
GH69	0.00	0.00	0.00	0.00	0.00	0.00	0.00	0.00	0.00	0.00	0.00	0.00	0.00	0.00	0.00	0.00
PL2	0.00	0.00	0.00	0.00	0.00	0.00	0.00	0.00	0.00	0.00	0.00	0.00	0.00	0.00	0.00	0.00
GH49	0.00	0.00	0.00	0.00	0.00	0.00	0.00	0.00	0.00	0.00	0.00	0.00	0.00	0.00	0.00	0.00
GH25	0.24	0.38	0.48	0.30	0.17	0.35	0.26	0.23	0.44	0.23	0.44	0.35	0.24	0.28	0.37	0.59
GH59	0.01	0.04	0.03	0.00	0.02	0.00	0.06	0.02	0.00	0.03	0.00	0.00	0.00	0.00	0.04	0.00
GH35	0.55	0.27	0.24	0.13	0.35	0.13	0.15	0.67	0.06	0.57	0.31	0.39	0.35	0.64	0.50	0.27
GT39	0.00	0.00	0.00	0.00	0.00	0.00	0.00	0.00	0.00	0.00	0.00	0.00	0.00	0.00	0.01	0.00
GT57	0.00	0.00	0.00	0.00	0.00	0.00	0.00	0.00	0.00	0.00	0.00	0.00	0.00	0.00	0.00	0.00
GH129	0.00	0.00	0.03	0.00	0.00	0.03	0.00	0.00	0.06	0.00	0.00	0.00	0.00	0.00	0.00	0.00
GT22	0.00	0.00	0.00	0.00	0.00	0.00	0.00	0.00	0.00	0.00	0.00	0.00	0.00	0.00	0.00	0.00
PL12	0.31	0.15	0.00	0.05	0.07	0.03	0.04	0.17	0.00	0.21	0.13	0.00	0.12	0.12	0.16	0.00
PL18	0.00	0.00	0.00	0.00	0.00	0.00	0.00	0.00	0.00	0.00	0.00	0.00	0.00	0.00	0.00	0.00
GT92	0.00	0.00	0.00	0.00	0.00	0.00	0.00	0.00	0.00	0.00	0.00	0.00	0.00	0.00	0.00	0.00
GT44	0.00	0.00	0.00	0.00	0.00	0.00	0.00	0.00	0.00	0.00	0.00	0.00	0.00	0.00	0.00	0.00
GT29	0.00	0.00	0.00	0.00	0.00	0.00	0.00	0.00	0.00	0.00	0.00	0.00	0.00	0.00	0.00	0.00
GT13	0.00	0.00	0.00	0.00	0.00	0.00	0.00	0.00	0.00	0.00	0.00	0.00	0.00	0.00	0.00	0.00

Normalized_abundance_2

CAZY_group	CN-AD-119	CN-AD-120	CN-AD-121	CN-AD-122	CN-AD-123	CN-AD-124	CN-AD-125	CN-AD-126	CN-AD-127	CN-AD-128	CN-AD-129	CN-AD-130	CN-AD-131	CN-AD-132	CN-AD-133	CN-AD-134
**GT52**	0.00	0.00	0.00	0.00	0.00	0.00	0.00	0.00	0.00	0.00	0.00	0.00	0.00	0.00	0.00	0.00
**GT64**	0.00	0.00	0.00	0.00	0.00	0.00	0.00	0.00	0.00	0.00	0.00	0.00	0.00	0.00	0.00	0.00
**GT85**	0.00	0.00	0.00	0.00	0.00	0.00	0.00	0.00	0.00	0.00	0.00	0.00	0.00	0.00	0.00	0.00
**GT16**	0.00	0.00	0.00	0.00	0.00	0.00	0.00	0.00	0.00	0.00	0.00	0.00	0.00	0.00	0.00	0.00
**GH56**	0.00	0.00	0.00	0.00	0.00	0.00	0.00	0.00	0.00	0.00	0.00	0.00	0.00	0.00	0.00	0.00
**GT60**	0.00	0.00	0.00	0.00	0.00	0.00	0.00	0.00	0.00	0.00	0.00	0.00	0.00	0.00	0.00	0.00
**GH41**	0.00	0.00	0.00	0.00	0.00	0.00	0.00	0.00	0.00	0.00	0.00	0.00	0.00	0.00	0.00	0.00
**CE7**	0.16	0.11	0.12	0.07	0.06	0.07	0.09	0.12	0.03	0.13	0.15	0.04	0.12	0.07	0.16	0.09
**GH39**	0.00	0.03	0.03	0.05	0.06	0.04	0.09	0.00	0.02	0.01	0.10	0.02	0.00	0.00	0.06	0.11
**GT30**	0.31	0.18	0.21	0.13	0.18	0.19	0.11	0.33	0.07	0.20	0.31	0.19	0.23	0.35	0.30	0.11
**PL15**	0.26	0.05	0.00	0.04	0.06	0.06	0.02	0.15	0.00	0.24	0.07	0.00	0.05	0.12	0.14	0.14
**GH102**	0.01	0.01	0.03	0.03	0.02	0.09	0.00	0.00	0.01	0.00	0.02	0.00	0.02	0.02	0.00	0.02

CAZY_group	CN-AD-135	CN-AD-136	CN-AD-137	CN-AD-138	CN-AD-139	CN-AD-140	CN-AD-141	CN-AD-142	CN-AD-143	CN-AD-144	IN-CH-17	IN-CH-18	IN-CH-19	IN-CH-20	IN-IN-2	VE-AD-1
GH29	0.42	0.54	0.48	0.80	0.58	0.45	0.44	0.23	0.68	0.53	0.58	0.49	0.54	0.67	0.54	1.04
PL4	0.00	0.00	0.00	0.00	0.00	0.02	0.00	0.00	0.01	0.00	0.00	0.00	0.00	0.00	0.00	0.00
GH23	0.89	1.14	0.99	0.76	0.83	1.04	0.96	1.01	0.95	0.92	0.58	0.57	0.61	0.89	0.68	2.08
CE6	0.04	0.03	0.02	0.02	0.04	0.04	0.07	0.02	0.05	0.05	0.19	0.33	0.31	0.24	0.10	0.31
GH72	0.06	0.08	0.05	0.06	0.10	0.02	0.09	0.02	0.05	0.11	0.10	0.13	0.14	0.10	0.10	0.73
GH114	0.00	0.00	0.00	0.00	0.00	0.00	0.00	0.00	0.00	0.00	0.00	0.00	0.00	0.00	0.00	0.00
GH78	0.47	0.55	0.66	0.78	0.87	0.74	0.25	0.25	1.18	1.03	0.13	0.20	0.14	0.22	0.50	0.10
PL19	0.00	0.00	0.00	0.00	0.00	0.00	0.00	0.00	0.00	0.00	0.00	0.00	0.00	0.00	0.00	0.00
GT27	0.00	0.00	0.00	0.00	0.00	0.00	0.00	0.00	0.00	0.00	0.00	0.00	0.00	0.00	0.00	0.10
GH5	0.33	0.28	0.23	0.19	0.19	0.32	0.22	0.18	0.08	0.29	0.26	0.84	0.68	0.75	0.41	0.73
GT40	0.00	0.00	0.00	0.00	0.00	0.00	0.00	0.00	0.00	0.00	0.00	0.00	0.00	0.00	0.00	0.00
CE13	0.00	0.02	0.00	0.00	0.00	0.00	0.00	0.00	0.00	0.00	0.00	0.00	0.00	0.00	0.00	0.00
GT37	0.00	0.00	0.00	0.00	0.00	0.00	0.00	0.00	0.00	0.00	0.00	0.00	0.00	0.00	0.00	0.00
GH33	0.14	0.25	0.25	0.31	0.23	0.20	0.15	0.05	0.15	0.21	0.00	0.02	0.02	0.02	0.15	0.10
GT65	0.00	0.00	0.00	0.00	0.00	0.00	0.00	0.00	0.00	0.00	0.00	0.00	0.00	0.00	0.00	0.00
GH86	0.00	0.00	0.00	0.00	0.00	0.00	0.00	0.00	0.00	0.00	0.00	0.00	0.00	0.00	0.00	0.00
GH123	0.04	0.06	0.12	0.06	0.19	0.11	0.01	0.02	0.10	0.15	0.03	0.07	0.09	0.10	0.06	0.21
GH96	0.00	0.00	0.00	0.00	0.00	0.00	0.00	0.00	0.00	0.00	0.00	0.00	0.00	0.00	0.00	0.00
GH14	0.00	0.00	0.00	0.00	0.00	0.00	0.00	0.00	0.00	0.00	0.00	0.00	0.00	0.00	0.00	0.00
CE3	0.00	0.00	0.00	0.00	0.00	0.00	0.00	0.00	0.00	0.00	0.00	0.00	0.00	0.00	0.00	0.00
PL10	0.10	0.05	0.07	0.10	0.10	0.05	0.19	0.00	0.15	0.06	0.16	0.18	0.22	0.24	0.23	0.10
GT48	0.00	0.00	0.00	0.00	0.00	0.00	0.00	0.00	0.00	0.00	0.00	0.00	0.00	0.00	0.00	0.00
PL6	0.00	0.00	0.00	0.00	0.00	0.00	0.00	0.00	0.00	0.00	0.00	0.00	0.00	0.00	0.00	0.00
GT83	0.05	0.03	0.15	0.08	0.14	0.13	0.04	0.11	0.08	0.08	0.06	0.13	0.13	0.26	0.06	0.10
GH126	0.00	0.00	0.00	0.00	0.00	0.00	0.00	0.00	0.00	0.00	0.00	0.00	0.00	0.00	0.00	0.00
GH9	0.15	0.35	0.12	0.19	0.17	0.27	0.01	0.02	0.07	0.16	0.03	0.02	0.05	0.08	0.17	0.00
GH75	0.00	0.00	0.00	0.00	0.00	0.00	0.00	0.00	0.00	0.00	0.00	0.00	0.00	0.00	0.00	0.00
GT71	0.00	0.00	0.00	0.00	0.00	0.00	0.00	0.00	0.00	0.00	0.00	0.00	0.00	0.00	0.00	0.00
GH46	0.00	0.00	0.00	0.00	0.00	0.00	0.00	0.00	0.00	0.00	0.00	0.00	0.00	0.00	0.00	0.00
GT61	0.00	0.00	0.00	0.00	0.00	0.00	0.00	0.00	0.00	0.00	0.00	0.00	0.00	0.00	0.00	0.00
GT3	0.05	0.09	0.12	0.15	0.02	0.04	0.04	0.00	0.08	0.06	0.16	0.20	0.25	0.22	0.12	0.10
PL3	0.00	0.00	0.00	0.00	0.00	0.00	0.00	0.00	0.00	0.00	0.00	0.00	0.00	0.00	0.00	0.00
GH108	0.01	0.02	0.03	0.04	0.04	0.09	0.01	0.02	0.05	0.05	0.03	0.00	0.02	0.00	0.00	0.00
GT56	0.00	0.00	0.05	0.00	0.00	0.02	0.01	0.00	0.01	0.02	0.00	0.02	0.00	0.00	0.00	0.21
PL11	0.11	0.03	0.07	0.10	0.10	0.05	0.06	0.09	0.20	0.08	0.00	0.09	0.02	0.10	0.02	0.00
GH45	0.00	0.00	0.00	0.00	0.00	0.00	0.00	0.00	0.00	0.00	0.00	0.00	0.00	0.00	0.00	0.00
GT34	0.00	0.00	0.00	0.00	0.00	0.00	0.00	0.00	0.00	0.00	0.00	0.00	0.00	0.00	0.00	0.00
PL14	0.00	0.00	0.00	0.00	0.00	0.00	0.00	0.00	0.00	0.00	0.00	0.00	0.00	0.00	0.00	0.00
GH19	0.04	0.02	0.02	0.00	0.02	0.00	0.09	0.00	0.01	0.00	0.00	0.04	0.02	0.02	0.00	0.10
GH118	0.00	0.00	0.00	0.00	0.00	0.00	0.00	0.00	0.00	0.00	0.00	0.00	0.00	0.00	0.00	0.00
GH82	0.00	0.00	0.00	0.00	0.00	0.00	0.00	0.00	0.00	0.00	0.00	0.00	0.00	0.00	0.00	0.00
GH76	0.14	0.06	0.15	0.13	0.12	0.20	0.00	0.00	0.41	0.34	0.00	0.02	0.00	0.02	0.02	0.00
GT4	1.60	1.49	1.50	1.91	1.71	1.55	1.54	0.89	1.81	1.76	1.09	1.64	1.58	1.63	1.26	2.91
GT87	0.00	0.02	0.00	0.00	0.00	0.00	0.00	0.00	0.00	0.00	0.00	0.00	0.00	0.00	0.00	0.10
GH24	0.13	0.24	0.30	0.13	0.16	0.22	0.12	0.25	0.18	0.26	0.35	0.42	0.38	0.57	0.35	0.10
GH90	0.00	0.00	0.02	0.00	0.00	0.00	0.00	0.00	0.00	0.00	0.00	0.00	0.00	0.00	0.00	0.00
GH57	0.06	0.13	0.03	0.15	0.08	0.07	0.07	0.02	0.05	0.11	0.29	0.09	0.23	0.35	0.08	0.21
GH36	0.66	0.47	0.41	0.46	0.50	0.54	0.73	0.50	0.79	0.76	0.71	0.88	0.86	1.04	0.91	1.35
GT46	0.00	0.00	0.00	0.00	0.00	0.00	0.00	0.00	0.00	0.00	0.00	0.00	0.00	0.00	0.00	0.00
GH66	0.01	0.02	0.03	0.00	0.02	0.05	0.04	0.05	0.04	0.06	0.06	0.09	0.09	0.10	0.04	0.31

CAZY_group	CN-AD-135	CN-AD-136	CN-AD-137	CN-AD-138	CN-AD-139	CN-AD-140	CN-AD-141	CN-AD-142	CN-AD-143	CN-AD-144	IN-CH-17	IN-CH-18	IN-CH-19	IN-CH-20	IN-IN-2	VE-AD-1
GH48	0.00	0.00	0.00	0.00	0.00	0.00	0.00	0.00	0.00	0.00	0.00	0.00	0.00	0.00	0.00	0.00
GT53	0.00	0.00	0.00	0.00	0.00	0.00	0.00	0.00	0.00	0.00	0.00	0.00	0.00	0.00	0.00	0.00
GH60	0.00	0.00	0.00	0.00	0.00	0.00	0.00	0.00	0.00	0.00	0.00	0.00	0.00	0.00	0.00	0.00
GT31	0.00	0.00	0.02	0.00	0.00	0.00	0.00	0.00	0.00	0.02	0.00	0.02	0.00	0.00	0.00	0.00
GH105	0.51	0.32	0.41	0.50	0.60	0.29	0.39	0.21	0.61	0.52	0.13	0.24	0.18	0.31	0.15	0.62
GT23	0.01	0.00	0.00	0.00	0.00	0.00	0.00	0.02	0.00	0.00	0.00	0.00	0.00	0.00	0.00	0.00
GH11	0.00	0.00	0.00	0.00	0.00	0.00	0.00	0.00	0.00	0.00	0.00	0.00	0.00	0.00	0.00	0.00
GT38	0.00	0.00	0.00	0.00	0.00	0.00	0.00	0.00	0.00	0.00	0.00	0.00	0.00	0.00	0.00	0.00
GT43	0.00	0.00	0.00	0.00	0.00	0.00	0.00	0.00	0.00	0.00	0.00	0.00	0.00	0.00	0.00	0.00
GH99	0.00	0.00	0.00	0.00	0.00	0.04	0.00	0.00	0.03	0.00	0.00	0.00	0.00	0.00	0.00	0.00
GH38	0.24	0.19	0.13	0.19	0.10	0.20	0.09	0.21	0.24	0.29	0.00	0.04	0.02	0.02	0.04	0.73
GH109	0.19	0.25	0.25	0.32	0.19	0.20	0.16	0.09	0.23	0.18	0.29	0.20	0.22	0.26	0.21	0.31
GH2	3.08	3.35	3.35	3.46	3.39	3.51	2.24	2.01	3.37	3.64	2.41	3.25	3.10	2.93	2.99	8.21
GT28	0.46	0.33	0.28	0.32	0.33	0.20	0.51	0.50	0.39	0.27	0.19	0.27	0.38	0.45	0.35	0.94
GH42	0.18	0.09	0.07	0.17	0.16	0.11	0.26	0.23	0.23	0.23	0.00	0.00	0.00	0.04	0.06	0.94
GT58	0.00	0.00	0.00	0.00	0.00	0.00	0.00	0.00	0.00	0.00	0.00	0.00	0.00	0.00	0.00	0.00
GH61	0.00	0.00	0.00	0.00	0.00	0.00	0.00	0.00	0.00	0.00	0.00	0.00	0.00	0.00	0.00	0.00
GH80	0.00	0.00	0.00	0.00	0.00	0.00	0.00	0.00	0.00	0.00	0.00	0.00	0.00	0.00	0.00	0.00
GH98	0.00	0.00	0.03	0.00	0.00	0.00	0.00	0.00	0.00	0.00	0.00	0.00	0.00	0.00	0.00	0.00
GH6	0.00	0.00	0.00	0.00	0.00	0.00	0.00	0.00	0.00	0.00	0.00	0.00	0.00	0.00	0.00	0.00
GT1	0.04	0.05	0.00	0.04	0.06	0.00	0.06	0.05	0.03	0.05	0.00	0.02	0.04	0.00	0.04	0.00
GH28	0.57	0.40	0.66	0.63	0.58	0.50	0.52	0.48	0.97	0.66	0.64	0.88	1.04	0.77	0.85	1.66
GT42	0.00	0.00	0.00	0.00	0.00	0.00	0.00	0.00	0.00	0.00	0.00	0.00	0.00	0.00	0.00	0.00
GH128	0.00	0.00	0.00	0.00	0.00	0.00	0.00	0.00	0.00	0.00	0.00	0.02	0.02	0.00	0.00	0.00
CE12	0.13	0.02	0.15	0.02	0.08	0.09	0.15	0.05	0.05	0.06	0.10	0.35	0.32	0.22	0.15	0.83
CE16	0.00	0.00	0.00	0.00	0.00	0.00	0.00	0.00	0.00	0.00	0.00	0.00	0.00	0.00	0.00	0.00
GT89	0.00	0.00	0.00	0.00	0.00	0.00	0.00	0.00	0.00	0.00	0.00	0.00	0.00	0.00	0.00	0.00
GT11	0.08	0.05	0.05	0.11	0.04	0.02	0.04	0.11	0.04	0.03	0.00	0.00	0.02	0.02	0.02	0.00
GT77	0.01	0.02	0.02	0.02	0.00	0.02	0.01	0.02	0.01	0.00	0.03	0.11	0.13	0.02	0.04	0.83
GT55	0.00	0.00	0.00	0.00	0.00	0.00	0.00	0.00	0.00	0.00	0.00	0.00	0.00	0.00	0.00	0.00
GT18	0.00	0.00	0.00	0.00	0.00	0.00	0.00	0.00	0.00	0.00	0.00	0.00	0.00	0.00	0.00	0.00
GH87	0.00	0.00	0.00	0.00	0.02	0.00	0.06	0.05	0.00	0.02	0.00	0.00	0.02	0.00	0.00	0.00
PL5	0.00	0.00	0.00	0.00	0.00	0.00	0.00	0.00	0.00	0.00	0.00	0.00	0.00	0.00	0.00	0.00
GH22	0.00	0.00	0.00	0.00	0.00	0.00	0.00	0.00	0.00	0.00	0.00	0.00	0.00	0.00	0.00	0.00
CE2	0.04	0.06	0.00	0.02	0.02	0.04	0.07	0.00	0.03	0.00	0.03	0.04	0.02	0.04	0.04	0.00
GH120	0.01	0.00	0.00	0.02	0.00	0.02	0.10	0.18	0.04	0.05	0.00	0.00	0.00	0.00	0.00	0.21
GH73	0.32	0.35	0.30	0.38	0.37	0.27	0.51	0.60	0.49	0.44	0.29	0.71	0.59	0.71	0.44	1.35
GT17	0.00	0.00	0.00	0.00	0.00	0.00	0.00	0.00	0.00	0.00	0.00	0.00	0.00	0.00	0.00	0.00
GH124	0.00	0.00	0.00	0.00	0.00	0.00	0.00	0.00	0.00	0.00	0.00	0.00	0.00	0.00	0.00	0.00
GH54	0.00	0.00	0.00	0.00	0.00	0.00	0.00	0.00	0.00	0.00	0.00	0.00	0.00	0.00	0.00	0.00
GH100	0.00	0.00	0.00	0.00	0.00	0.00	0.00	0.00	0.00	0.00	0.00	0.00	0.00	0.00	0.00	0.00
GH113	0.04	0.03	0.00	0.00	0.00	0.00	0.01	0.02	0.00	0.02	0.00	0.02	0.00	0.02	0.00	0.00
GT5	0.40	0.25	0.23	0.27	0.14	0.25	0.45	0.48	0.27	0.39	0.23	0.33	0.31	0.31	0.29	0.42
GH94	0.46	0.33	0.25	0.11	0.08	0.29	0.35	0.32	0.12	0.26	0.23	0.42	0.43	0.41	0.31	0.83
GT66	0.00	0.00	0.00	0.00	0.00	0.00	0.00	0.00	0.00	0.00	0.00	0.00	0.00	0.00	0.02	0.00
GT94	0.00	0.00	0.00	0.00	0.00	0.00	0.00	0.00	0.00	0.00	0.00	0.00	0.00	0.00	0.00	0.00
GH58	0.00	0.00	0.00	0.00	0.00	0.00	0.00	0.00	0.00	0.00	0.00	0.00	0.00	0.00	0.00	0.00
GH1	0.43	0.30	0.53	0.23	0.23	0.41	1.00	0.60	0.73	0.47	0.03	0.24	0.09	0.10	0.19	0.94
PL22	0.00	0.00	0.02	0.00	0.00	0.00	0.00	0.00	0.00	0.00	0.00	0.00	0.02	0.00	0.00	0.00
GT9	0.13	0.13	0.18	0.27	0.17	0.05	0.25	0.07	0.11	0.06	0.32	0.40	0.27	0.41	0.29	0.62

CAZY_group	CN-AD-135	CN-AD-136	CN-AD-137	CN-AD-138	CN-AD-139	CN-AD-140	CN-AD-141	CN-AD-142	CN-AD-143	CN-AD-144	IN-CH-17	IN-CH-18	IN-CH-19	IN-CH-20	IN-IN-2	VE-AD-1
GT76	0.00	0.00	0.00	0.00	0.00	0.00	0.00	0.00	0.00	0.00	0.00	0.00	0.00	0.00	0.00	0.00
GT32	0.03	0.09	0.08	0.06	0.04	0.02	0.07	0.02	0.14	0.13	0.03	0.09	0.04	0.12	0.06	0.10
GT47	0.03	0.03	0.02	0.00	0.04	0.02	0.06	0.05	0.03	0.02	0.03	0.00	0.02	0.10	0.02	0.42
GH84	0.09	0.13	0.10	0.17	0.19	0.11	0.23	0.05	0.19	0.08	0.19	0.18	0.25	0.18	0.06	0.31
GH83	0.00	0.00	0.00	0.00	0.00	0.00	0.00	0.00	0.00	0.00	0.00	0.00	0.00	0.00	0.00	0.00
GT59	0.00	0.00	0.00	0.00	0.00	0.00	0.00	0.00	0.00	0.00	0.00	0.00	0.00	0.00	0.00	0.00
GH34	0.00	0.00	0.00	0.00	0.00	0.00	0.00	0.00	0.00	0.00	0.00	0.00	0.00	0.00	0.00	0.00
GT14	0.04	0.03	0.03	0.04	0.06	0.05	0.01	0.00	0.01	0.03	0.00	0.04	0.05	0.04	0.00	0.00
GH47	0.00	0.00	0.00	0.00	0.00	0.00	0.00	0.00	0.00	0.00	0.00	0.00	0.00	0.00	0.00	0.00
GH97	0.45	0.70	0.56	0.71	0.79	0.59	0.25	0.09	0.67	0.87	1.06	1.30	1.40	1.18	0.91	1.25
GH50	0.05	0.00	0.03	0.04	0.00	0.09	0.00	0.00	0.05	0.05	0.00	0.00	0.00	0.00	0.00	0.00
GT26	0.18	0.16	0.17	0.19	0.10	0.25	0.15	0.23	0.14	0.18	0.26	0.24	0.23	0.28	0.17	0.52
GH18	0.45	0.27	0.30	0.31	0.52	0.50	0.42	0.39	0.72	0.61	0.16	0.11	0.11	0.26	0.19	0.73
GH37	0.00	0.00	0.12	0.00	0.00	0.02	0.04	0.00	0.03	0.03	0.00	0.04	0.00	0.00	0.00	0.10
PL13	0.01	0.00	0.03	0.00	0.04	0.05	0.03	0.00	0.01	0.05	0.00	0.00	0.00	0.00	0.00	0.00
GH27	0.13	0.06	0.07	0.15	0.14	0.13	0.09	0.02	0.23	0.19	0.16	0.22	0.23	0.22	0.15	0.31
GT54	0.00	0.00	0.00	0.00	0.00	0.00	0.00	0.00	0.00	0.00	0.00	0.00	0.00	0.00	0.00	0.00
GT91	0.00	0.00	0.00	0.00	0.00	0.00	0.00	0.00	0.00	0.00	0.00	0.00	0.00	0.00	0.00	0.00
GT72	0.00	0.00	0.00	0.00	0.00	0.00	0.00	0.00	0.00	0.00	0.00	0.00	0.00	0.00	0.00	0.00
GH67	0.04	0.03	0.10	0.02	0.04	0.07	0.00	0.02	0.10	0.06	0.16	0.29	0.25	0.22	0.12	0.21
GH12	0.00	0.00	0.00	0.00	0.00	0.00	0.00	0.00	0.00	0.00	0.00	0.00	0.00	0.00	0.00	0.00
GH91	0.03	0.02	0.03	0.02	0.00	0.00	0.00	0.00	0.04	0.03	0.00	0.00	0.00	0.00	0.00	0.00
GT69	0.00	0.00	0.00	0.00	0.00	0.00	0.00	0.00	0.00	0.00	0.00	0.00	0.00	0.00	0.00	0.00
GH106	0.11	0.21	0.28	0.15	0.25	0.22	0.13	0.11	0.19	0.13	0.10	0.20	0.16	0.28	0.21	0.21
GT62	0.00	0.00	0.00	0.00	0.00	0.00	0.00	0.00	0.00	0.00	0.00	0.00	0.00	0.00	0.00	0.00
GH17	0.00	0.00	0.00	0.00	0.00	0.00	0.00	0.00	0.00	0.00	0.00	0.00	0.00	0.00	0.00	0.00
GH88	0.23	0.25	0.30	0.27	0.47	0.27	0.22	0.16	0.31	0.37	0.10	0.11	0.14	0.16	0.17	0.00
CE5	0.00	0.00	0.00	0.00	0.00	0.00	0.00	0.00	0.00	0.00	0.00	0.00	0.00	0.00	0.00	0.00
GT8	0.18	0.09	0.07	0.06	0.04	0.05	0.13	0.16	0.04	0.00	0.03	0.04	0.02	0.10	0.04	0.10
GT50	0.00	0.00	0.00	0.00	0.00	0.00	0.00	0.00	0.00	0.00	0.00	0.00	0.00	0.00	0.00	0.00
GT20	0.03	0.06	0.15	0.11	0.08	0.07	0.03	0.05	0.08	0.13	0.00	0.02	0.00	0.00	0.00	0.10
PL9	0.09	0.09	0.26	0.06	0.06	0.05	0.19	0.07	0.08	0.06	0.00	0.00	0.07	0.06	0.00	0.00
GH55	0.05	0.02	0.02	0.04	0.02	0.04	0.01	0.00	0.00	0.02	0.00	0.00	0.02	0.02	0.00	0.10
GH64	0.00	0.00	0.00	0.00	0.00	0.00	0.00	0.00	0.00	0.00	0.00	0.00	0.00	0.00	0.00	0.00
GH127	0.22	0.11	0.17	0.13	0.21	0.11	0.19	0.23	0.29	0.24	0.32	0.38	0.45	0.39	0.29	1.14
GH103	0.03	0.00	0.03	0.00	0.00	0.00	0.06	0.00	0.01	0.02	0.00	0.04	0.04	0.00	0.00	0.21
GH3	2.40	1.85	1.90	1.95	2.13	2.25	1.54	1.37	2.22	3.05	1.93	2.63	2.74	2.64	2.34	5.51
GT45	0.00	0.00	0.00	0.00	0.00	0.00	0.00	0.00	0.00	0.00	0.00	0.00	0.00	0.00	0.00	0.00
GT74	0.00	0.00	0.00	0.00	0.00	0.00	0.00	0.00	0.00	0.00	0.00	0.00	0.00	0.00	0.00	0.00
GT90	0.00	0.00	0.00	0.00	0.00	0.00	0.00	0.00	0.00	0.00	0.00	0.00	0.00	0.00	0.00	0.00
GH116	0.10	0.13	0.08	0.15	0.06	0.13	0.04	0.00	0.01	0.06	0.00	0.00	0.00	0.02	0.00	0.00
GH31	0.89	0.71	0.97	0.92	0.83	0.84	0.71	0.76	0.92	0.98	1.00	0.84	1.01	0.77	0.89	2.49
GT12	0.00	0.00	0.00	0.00	0.00	0.00	0.00	0.00	0.00	0.00	0.00	0.00	0.00	0.00	0.00	0.00
CE1	0.28	0.25	0.36	0.38	0.27	0.29	0.35	0.18	0.33	0.37	0.19	0.35	0.23	0.26	0.25	0.83
GH121	0.00	0.00	0.00	0.00	0.00	0.00	0.00	0.02	0.01	0.00	0.00	0.00	0.00	0.00	0.00	0.00
GH79	0.00	0.02	0.00	0.00	0.00	0.00	0.00	0.00	0.00	0.00	0.00	0.00	0.00	0.00	0.00	0.21
GT73	0.00	0.00	0.00	0.00	0.00	0.00	0.00	0.00	0.00	0.00	0.00	0.00	0.00	0.00	0.00	0.00
GH77	0.63	0.63	0.50	0.36	0.37	0.50	0.64	0.53	0.37	0.48	0.39	0.44	0.45	0.55	0.41	2.18
PL17	0.00	0.00	0.03	0.00	0.04	0.04	0.00	0.05	0.00	0.00	0.00	0.00	0.00	0.00	0.00	0.00
PL8	0.20	0.13	0.25	0.32	0.25	0.25	0.10	0.00	0.29	0.16	0.00	0.13	0.02	0.12	0.08	0.21

CAZY_group	CN-AD-135	CN-AD-136	CN-AD-137	CN-AD-138	CN-AD-139	CN-AD-140	CN-AD-141	CN-AD-142	CN-AD-143	CN-AD-144	IN-CH-17	IN-CH-18	IN-CH-19	IN-CH-20	IN-IN-2	VE-AD-1
CE9	0.31	0.27	0.30	0.36	0.21	0.32	0.45	0.37	0.44	0.35	0.10	0.18	0.09	0.22	0.17	1.25
CE15	0.00	0.03	0.02	0.02	0.04	0.00	0.00	0.00	0.01	0.02	0.00	0.00	0.00	0.02	0.00	0.10
GT79	0.00	0.00	0.00	0.00	0.00	0.00	0.00	0.00	0.00	0.00	0.00	0.00	0.00	0.00	0.00	0.00
GT10	0.00	0.05	0.03	0.04	0.00	0.00	0.00	0.00	0.00	0.00	0.00	0.00	0.00	0.00	0.02	0.00
GH119	0.00	0.00	0.00	0.00	0.00	0.00	0.00	0.00	0.00	0.00	0.00	0.00	0.00	0.00	0.00	0.00
GH16	0.09	0.17	0.13	0.27	0.21	0.23	0.06	0.02	0.19	0.18	0.13	0.20	0.16	0.26	0.21	0.10
GH43	1.41	1.15	1.44	1.99	1.43	1.62	1.03	0.85	1.71	1.84	1.86	2.54	2.90	2.70	1.97	4.05
GH51	0.38	0.40	0.30	0.34	0.45	0.31	0.26	0.43	0.34	0.45	0.64	0.84	0.88	0.94	0.83	1.35
GT35	0.83	0.76	0.79	0.55	0.52	0.49	1.09	0.78	0.61	0.77	0.48	0.64	0.56	0.53	0.64	2.18
GT25	0.05	0.00	0.02	0.00	0.00	0.00	0.03	0.00	0.00	0.00	0.00	0.00	0.00	0.00	0.00	0.00
GT2	3.62	3.00	3.27	3.23	2.64	2.84	3.75	3.16	3.59	3.53	4.89	4.35	5.13	5.12	4.13	11.85
GT51	0.72	0.60	0.79	0.55	0.72	0.68	0.95	0.69	0.72	0.84	0.96	1.17	1.13	1.18	0.93	3.33
GH107	0.00	0.00	0.00	0.00	0.00	0.00	0.00	0.00	0.00	0.00	0.00	0.00	0.00	0.00	0.00	0.00
GH7	0.00	0.00	0.00	0.00	0.00	0.00	0.00	0.00	0.00	0.00	0.00	0.00	0.00	0.00	0.00	0.00
GT7	0.00	0.00	0.00	0.00	0.00	0.00	0.00	0.00	0.00	0.00	0.00	0.00	0.00	0.00	0.00	0.00
GT36	0.00	0.00	0.00	0.00	0.00	0.00	0.00	0.00	0.00	0.00	0.00	0.00	0.00	0.00	0.00	0.00
PL7	0.00	0.00	0.00	0.00	0.00	0.00	0.00	0.00	0.00	0.00	0.00	0.00	0.00	0.00	0.00	0.00
GH110	0.04	0.14	0.10	0.17	0.16	0.02	0.07	0.02	0.10	0.10	0.06	0.04	0.09	0.04	0.10	0.00
GT21	0.00	0.00	0.00	0.00	0.00	0.00	0.00	0.00	0.00	0.00	0.00	0.00	0.00	0.00	0.00	0.00
GH62	0.00	0.00	0.00	0.00	0.00	0.00	0.00	0.00	0.00	0.00	0.00	0.00	0.00	0.00	0.00	0.00
GH125	0.15	0.16	0.17	0.15	0.08	0.22	0.06	0.02	0.15	0.23	0.19	0.07	0.18	0.22	0.08	0.31
GH117	0.08	0.06	0.07	0.10	0.10	0.05	0.06	0.00	0.10	0.05	0.00	0.00	0.00	0.02	0.02	0.00
GH68	0.00	0.00	0.00	0.00	0.00	0.00	0.01	0.05	0.00	0.00	0.00	0.00	0.00	0.00	0.00	0.00
GH85	0.01	0.02	0.00	0.02	0.00	0.00	0.01	0.05	0.00	0.00	0.00	0.09	0.04	0.18	0.00	0.21
GH92	1.16	1.17	1.42	1.74	1.43	1.20	0.42	0.09	1.88	1.56	1.25	0.88	1.10	1.75	0.91	1.66
GH40	0.00	0.00	0.00	0.00	0.00	0.00	0.00	0.00	0.00	0.00	0.00	0.00	0.00	0.00	0.00	0.00
GT49	0.00	0.00	0.00	0.00	0.00	0.00	0.00	0.00	0.00	0.00	0.00	0.00	0.00	0.00	0.00	0.00
GH112	0.22	0.16	0.12	0.10	0.08	0.18	0.17	0.18	0.15	0.16	0.03	0.07	0.05	0.02	0.08	0.31
GT67	0.00	0.00	0.00	0.00	0.00	0.00	0.00	0.00	0.00	0.00	0.00	0.00	0.00	0.00	0.00	0.00
CE8	0.24	0.05	0.30	0.21	0.14	0.16	0.32	0.07	0.18	0.08	0.10	0.38	0.23	0.14	0.17	0.21
GT86	0.00	0.00	0.00	0.00	0.00	0.00	0.00	0.00	0.00	0.00	0.00	0.00	0.00	0.00	0.00	0.00
GH101	0.01	0.00	0.00	0.04	0.00	0.00	0.06	0.00	0.01	0.02	0.00	0.00	0.00	0.00	0.00	0.00
GH81	0.00	0.00	0.00	0.00	0.00	0.00	0.00	0.00	0.00	0.00	0.00	0.00	0.00	0.00	0.00	0.00
GT93	0.00	0.00	0.00	0.00	0.00	0.00	0.00	0.00	0.00	0.00	0.00	0.00	0.00	0.00	0.00	0.00
GT63	0.00	0.00	0.00	0.00	0.00	0.00	0.00	0.00	0.00	0.00	0.00	0.00	0.00	0.00	0.00	0.00
CE11	0.17	0.14	0.20	0.17	0.21	0.07	0.19	0.07	0.18	0.13	0.39	0.35	0.45	0.41	0.35	0.42
GT19	0.10	0.08	0.08	0.19	0.19	0.11	0.15	0.02	0.16	0.10	0.29	0.24	0.32	0.35	0.17	0.42
GT33	0.00	0.00	0.00	0.00	0.00	0.00	0.00	0.00	0.00	0.00	0.00	0.00	0.00	0.00	0.00	0.00
GH21	0.00	0.00	0.00	0.00	0.00	0.00	0.00	0.00	0.00	0.00	0.00	0.00	0.00	0.00	0.00	0.00
GT68	0.00	0.00	0.00	0.00	0.00	0.00	0.00	0.00	0.00	0.00	0.00	0.00	0.00	0.00	0.00	0.00
GH89	0.11	0.22	0.13	0.15	0.25	0.32	0.12	0.05	0.20	0.15	0.23	0.13	0.27	0.26	0.12	0.31
GT82	0.00	0.00	0.00	0.02	0.00	0.00	0.00	0.00	0.00	0.00	0.00	0.00	0.00	0.00	0.00	0.00
GH30	0.19	0.06	0.28	0.27	0.27	0.18	0.03	0.02	0.22	0.23	0.03	0.15	0.13	0.10	0.06	0.31
GH20	0.64	0.95	0.74	1.13	1.01	0.93	0.48	0.30	1.10	0.97	0.90	1.13	1.13	0.94	1.02	0.94
GH52	0.00	0.00	0.00	0.00	0.00	0.00	0.00	0.00	0.00	0.00	0.00	0.00	0.00	0.00	0.00	0.00
GT84	0.00	0.00	0.00	0.00	0.00	0.00	0.00	0.00	0.00	0.00	0.00	0.00	0.00	0.00	0.00	0.00
GT15	0.00	0.00	0.00	0.00	0.00	0.00	0.00	0.00	0.00	0.00	0.00	0.00	0.00	0.00	0.00	0.00
GH93	0.00	0.00	0.00	0.00	0.00	0.02	0.00	0.00	0.00	0.03	0.00	0.00	0.00	0.00	0.00	0.00
PL16	0.00	0.00	0.00	0.00	0.00	0.00	0.00	0.00	0.00	0.00	0.00	0.00	0.00	0.00	0.00	0.00
GH26	0.06	0.06	0.08	0.11	0.04	0.04	0.01	0.07	0.03	0.10	0.00	0.07	0.04	0.04	0.02	0.31

CAZY_group	CN-AD-135	CN-AD-136	CN-AD-137	CN-AD-138	CN-AD-139	CN-AD-140	CN-AD-141	CN-AD-142	CN-AD-143	CN-AD-144	IN-CH-17	IN-CH-18	IN-CH-19	IN-CH-20	IN-IN-2	VE-AD-1
GT75	0.00	0.00	0.00	0.00	0.00	0.00	0.00	0.00	0.00	0.00	0.00	0.00	0.00	0.00	0.00	0.00
GH53	0.22	0.11	0.03	0.06	0.06	0.04	0.09	0.07	0.05	0.03	0.19	0.09	0.09	0.14	0.15	0.42
GT88	0.00	0.00	0.00	0.00	0.00	0.00	0.00	0.00	0.00	0.00	0.00	0.00	0.00	0.00	0.00	0.00
GT24	0.00	0.00	0.00	0.00	0.00	0.00	0.00	0.00	0.00	0.00	0.00	0.00	0.00	0.00	0.00	0.00
GT81	0.00	0.00	0.00	0.00	0.00	0.00	0.00	0.00	0.00	0.00	0.00	0.00	0.00	0.00	0.00	0.00
GH4	0.11	0.11	0.21	0.08	0.06	0.16	0.38	0.23	0.20	0.19	0.00	0.13	0.00	0.00	0.06	0.62
GT78	0.00	0.00	0.00	0.00	0.00	0.00	0.00	0.00	0.00	0.00	0.00	0.00	0.00	0.00	0.00	0.00
GH8	0.06	0.02	0.07	0.00	0.02	0.00	0.07	0.05	0.01	0.00	0.00	0.09	0.11	0.08	0.04	0.00
GH70	0.00	0.00	0.00	0.00	0.00	0.00	0.23	0.21	0.00	0.00	0.00	0.00	0.02	0.00	0.00	0.00
GH122	0.00	0.00	0.00	0.00	0.00	0.00	0.00	0.00	0.00	0.00	0.00	0.00	0.00	0.00	0.00	0.00
GH74	0.05	0.00	0.00	0.04	0.00	0.00	0.00	0.07	0.00	0.02	0.00	0.00	0.00	0.00	0.00	0.00
GT41	0.03	0.00	0.00	0.00	0.00	0.00	0.03	0.02	0.00	0.00	0.00	0.00	0.00	0.00	0.02	0.10
PL1	0.15	0.14	0.28	0.36	0.25	0.31	0.31	0.11	0.29	0.19	0.03	0.24	0.18	0.30	0.10	0.31
GH15	0.03	0.03	0.08	0.02	0.00	0.02	0.00	0.02	0.04	0.06	0.00	0.00	0.00	0.00	0.02	0.00
GH32	0.43	0.24	0.25	0.32	0.31	0.22	0.86	0.60	0.64	0.63	0.55	0.62	0.81	0.75	0.60	0.73
GH44	0.00	0.00	0.00	0.00	0.00	0.00	0.00	0.00	0.00	0.00	0.00	0.00	0.00	0.00	0.00	0.00
GT6	0.00	0.00	0.00	0.00	0.00	0.00	0.00	0.00	0.00	0.02	0.00	0.00	0.00	0.00	0.00	0.00
GH13	2.99	2.17	2.10	2.12	1.80	1.96	3.10	3.34	2.15	2.90	1.96	2.90	2.48	2.56	2.45	8.73
GT80	0.00	0.00	0.05	0.00	0.00	0.00	0.00	0.00	0.00	0.02	0.00	0.02	0.04	0.00	0.00	0.00
GH71	0.00	0.00	0.00	0.00	0.00	0.00	0.00	0.00	0.00	0.00	0.00	0.00	0.00	0.00	0.00	0.00
GH63	0.03	0.13	0.13	0.08	0.10	0.07	0.12	0.02	0.12	0.08	0.03	0.11	0.04	0.08	0.02	0.10
PL21	0.04	0.05	0.03	0.08	0.00	0.00	0.01	0.00	0.03	0.00	0.00	0.00	0.00	0.00	0.00	0.00
GH65	0.17	0.05	0.08	0.11	0.06	0.07	0.09	0.09	0.12	0.19	0.03	0.07	0.02	0.04	0.04	0.42
CE4	0.60	0.32	0.33	0.31	0.31	0.32	0.87	0.50	0.39	0.40	0.26	0.42	0.29	0.35	0.33	1.14
CE14	0.00	0.00	0.03	0.00	0.00	0.00	0.01	0.00	0.03	0.02	0.00	0.00	0.00	0.00	0.04	0.10
GH115	0.17	0.05	0.13	0.17	0.19	0.32	0.04	0.07	0.30	0.19	0.23	0.22	0.25	0.22	0.25	0.73
GH104	0.00	0.00	0.00	0.00	0.00	0.02	0.00	0.00	0.00	0.00	0.00	0.04	0.02	0.00	0.00	0.00
GH111	0.00	0.00	0.00	0.00	0.00	0.00	0.00	0.00	0.00	0.00	0.00	0.00	0.00	0.00	0.00	0.00
GH95	0.40	0.35	0.36	0.53	0.45	0.50	0.25	0.16	0.57	0.58	0.84	0.75	0.83	0.83	0.58	1.14
GT70	0.00	0.00	0.00	0.00	0.00	0.00	0.00	0.00	0.00	0.00	0.00	0.00	0.00	0.00	0.00	0.00
CE10	0.13	0.09	0.18	0.13	0.16	0.18	0.12	0.16	0.14	0.10	0.06	0.15	0.05	0.10	0.02	0.31
GH10	0.18	0.05	0.05	0.06	0.06	0.11	0.03	0.11	0.04	0.08	0.10	0.27	0.16	0.16	0.10	0.31
GH130	0.23	0.16	0.17	0.21	0.21	0.25	0.16	0.16	0.19	0.26	0.26	0.24	0.29	0.28	0.21	0.52
PL20	0.00	0.00	0.00	0.00	0.00	0.00	0.00	0.00	0.00	0.00	0.00	0.00	0.00	0.00	0.00	0.00
GH69	0.00	0.00	0.00	0.00	0.00	0.00	0.00	0.00	0.00	0.00	0.00	0.00	0.00	0.00	0.00	0.00
PL2	0.00	0.00	0.00	0.02	0.04	0.00	0.01	0.00	0.00	0.00	0.00	0.00	0.00	0.00	0.00	0.00
GH49	0.00	0.00	0.00	0.00	0.00	0.00	0.00	0.00	0.00	0.00	0.00	0.00	0.00	0.00	0.00	0.00
GH25	0.40	0.17	0.26	0.32	0.16	0.31	0.57	0.64	0.34	0.26	0.90	0.66	0.97	0.73	0.79	2.49
GH59	0.00	0.00	0.00	0.00	0.00	0.02	0.00	0.00	0.00	0.00	0.00	0.00	0.00	0.00	0.00	0.00
GH35	0.22	0.24	0.35	0.27	0.39	0.27	0.12	0.07	0.42	0.31	0.19	0.40	0.27	0.49	0.25	0.21
GT39	0.00	0.00	0.00	0.00	0.00	0.00	0.00	0.00	0.00	0.00	0.00	0.00	0.00	0.00	0.00	0.00
GT57	0.00	0.00	0.00	0.00	0.00	0.00	0.00	0.00	0.00	0.00	0.00	0.00	0.00	0.00	0.00	0.00
GH129	0.00	0.00	0.00	0.00	0.00	0.00	0.00	0.00	0.04	0.02	0.00	0.00	0.00	0.00	0.02	0.00
GT22	0.00	0.00	0.00	0.00	0.00	0.00	0.00	0.00	0.00	0.00	0.00	0.00	0.00	0.00	0.00	0.00
PL12	0.04	0.05	0.07	0.13	0.10	0.13	0.06	0.00	0.08	0.16	0.00	0.02	0.04	0.02	0.04	0.00
PL18	0.00	0.00	0.00	0.00	0.00	0.00	0.00	0.00	0.00	0.00	0.00	0.00	0.00	0.00	0.00	0.00
GT92	0.00	0.00	0.00	0.00	0.00	0.00	0.00	0.00	0.00	0.00	0.00	0.00	0.00	0.00	0.00	0.00
GT44	0.00	0.00	0.00	0.00	0.00	0.00	0.00	0.00	0.00	0.00	0.00	0.00	0.00	0.00	0.00	0.00
GT29	0.00	0.00	0.00	0.00	0.00	0.00	0.00	0.00	0.00	0.00	0.00	0.00	0.00	0.00	0.00	0.00
GT13	0.00	0.00	0.00	0.00	0.00	0.00	0.00	0.00	0.00	0.00	0.00	0.00	0.00	0.00	0.00	0.00

CAZY_group	CN-AD-135	CN-AD-136	CN-AD-137	CN-AD-138	CN-AD-139	CN-AD-140	CN-AD-141	CN-AD-142	CN-AD-143	CN-AD-144	IN-CH-17	IN-CH-18	IN-CH-19	IN-CH-20	IN-IN-2	VE-AD-1
GT52	0.00	0.00	0.00	0.00	0.00	0.00	0.00	0.00	0.00	0.00	0.00	0.00	0.00	0.00	0.00	0.00
GT64	0.00	0.00	0.00	0.00	0.00	0.00	0.00	0.00	0.00	0.00	0.00	0.00	0.00	0.00	0.00	0.00
GT85	0.00	0.00	0.00	0.00	0.00	0.00	0.00	0.00	0.00	0.00	0.00	0.00	0.00	0.00	0.00	0.00
GT16	0.00	0.00	0.00	0.00	0.00	0.00	0.00	0.00	0.00	0.00	0.00	0.00	0.00	0.00	0.00	0.00
GH56	0.00	0.00	0.00	0.00	0.00	0.00	0.00	0.00	0.00	0.00	0.00	0.00	0.00	0.00	0.00	0.00
GT60	0.00	0.00	0.00	0.00	0.00	0.00	0.00	0.00	0.00	0.00	0.00	0.00	0.00	0.00	0.00	0.00
GH41	0.00	0.00	0.00	0.00	0.00	0.00	0.00	0.00	0.00	0.00	0.00	0.00	0.00	0.00	0.00	0.00
CE7	0.09	0.08	0.12	0.13	0.10	0.13	0.03	0.02	0.15	0.15	0.23	0.18	0.29	0.18	0.17	0.00
GH39	0.05	0.03	0.03	0.02	0.00	0.09	0.09	0.05	0.01	0.05	0.00	0.02	0.04	0.04	0.00	0.10
GT30	0.24	0.25	0.33	0.32	0.27	0.18	0.17	0.09	0.27	0.21	0.29	0.44	0.36	0.47	0.46	0.52
PL15	0.09	0.00	0.12	0.08	0.14	0.09	0.00	0.00	0.12	0.15	0.03	0.04	0.05	0.08	0.06	0.00
GH102	0.06	0.02	0.08	0.02	0.00	0.05	0.09	0.00	0.03	0.03	0.00	0.04	0.04	0.02	0.00	0.21

CAZY_group	VE-AD-2	VE-AD-3	VE-AD-4	VE-AD-5	VE-AD-6	VE-CH-1	VE-CH-2	VE-CH-3	VE-CH-4	VE-CH-5	VE-CH-6	VE-IN-1	VE-IN-2	VE-IN-3	VE-IN-4	VE-IN-5
GH29	1.11	1.25	1.29	0.81	1.00	1.24	1.25	1.02	0.59	1.62	3.38	3.32	0.98	5.16	3.27	2.22
PL4	0.00	0.00	0.01	0.00	0.00	0.00	0.00	0.00	0.00	0.00	0.00	0.00	0.00	0.00	0.00	0.00
GH23	2.09	3.02	2.17	2.07	2.90	2.38	2.15	3.13	1.63	3.74	2.76	2.43	4.23	3.66	3.51	2.91
CE6	0.22	0.40	0.03	0.49	0.39	0.12	0.51	0.25	0.42	0.01	0.00	0.00	0.00	0.00	0.08	0.23
GH72	0.44	0.61	0.69	0.67	0.45	0.60	0.43	0.43	0.50	0.59	0.52	0.32	0.16	0.48	0.45	0.62
GH114	0.00	0.00	0.00	0.00	0.00	0.00	0.00	0.00	0.00	0.00	0.00	0.00	0.00	0.00	0.00	0.00
GH78	0.55	0.91	2.38	0.64	1.06	1.65	1.02	0.54	0.67	0.31	0.05	0.09	0.00	0.02	1.60	1.77
PL19	0.00	0.00	0.00	0.00	0.00	0.00	0.00	0.00	0.00	0.00	0.00	0.00	0.00	0.00	0.00	0.00
GT27	0.00	0.00	0.00	0.00	0.00	0.00	0.00	0.00	0.00	0.00	0.00	0.00	0.00	0.00	0.00	0.00
GH5	0.89	1.46	0.74	1.51	1.04	0.83	1.49	0.98	1.21	0.60	1.24	2.18	1.14	2.86	0.81	1.00
GT40	0.00	0.00	0.00	0.00	0.00	0.00	0.00	0.00	0.00	0.00	0.00	0.00	0.00	0.00	0.00	0.00
CE13	0.00	0.00	0.00	0.00	0.00	0.00	0.00	0.00	0.00	0.00	0.01	0.00	0.00	0.00	0.00	0.00
GT37	0.00	0.00	0.00	0.00	0.00	0.00	0.00	0.00	0.00	0.00	0.00	0.00	0.00	0.00	0.00	0.00
GH33	0.16	0.04	0.59	0.09	0.13	0.43	0.08	0.30	0.17	3.50	5.18	3.38	1.14	4.85	1.75	2.39
GT65	0.00	0.00	0.00	0.00	0.00	0.00	0.00	0.00	0.00	0.00	0.01	0.00	0.00	0.00	0.00	0.00
GH86	0.00	0.00	0.07	0.06	0.00	0.02	0.00	0.00	0.00	0.00	0.00	0.00	0.00	0.00	0.00	0.00
GH123	0.22	0.25	0.25	0.12	0.17	0.13	0.08	0.11	0.13	0.00	0.00	0.00	0.00	0.00	0.81	0.14
GH96	0.00	0.00	0.00	0.00	0.00	0.00	0.00	0.00	0.00	0.00	0.00	0.00	0.00	0.00	0.00	0.00
GH14	0.00	0.00	0.00	0.00	0.00	0.00	0.00	0.00	0.00	0.00	0.00	0.00	0.00	0.00	0.00	0.00
CE3	0.00	0.00	0.00	0.03	0.00	0.01	0.00	0.00	0.00	0.00	0.00	0.00	0.00	0.00	0.00	0.00
PL10	0.40	0.27	0.05	0.35	0.15	0.15	0.24	0.30	0.17	0.00	0.01	0.00	0.00	0.00	0.08	0.05
GT48	0.00	0.00	0.00	0.00	0.00	0.00	0.00	0.00	0.00	0.00	0.00	0.00	0.00	0.00	0.00	0.00
PL6	0.00	0.00	0.00	0.00	0.00	0.00	0.00	0.00	0.00	0.00	0.00	0.00	0.00	0.00	0.00	0.00
GT83	0.29	0.34	0.25	0.32	0.39	0.20	0.31	0.41	0.21	0.08	0.05	0.00	0.33	0.10	0.65	0.83
GH126	0.00	0.00	0.00	0.00	0.00	0.00	0.00	0.00	0.00	0.08	0.00	0.00	0.16	0.00	0.00	0.00
GH9	0.13	0.46	0.47	1.22	0.52	0.72	0.71	0.25	0.50	0.00	0.01	0.00	0.16	0.00	0.03	0.09
GH75	0.00	0.00	0.00	0.00	0.00	0.00	0.00	0.00	0.00	0.00	0.00	0.00	0.00	0.00	0.00	0.00
GT71	0.00	0.00	0.00	0.00	0.00	0.00	0.00	0.00	0.00	0.00	0.00	0.00	0.00	0.00	0.00	0.00
GH46	0.00	0.00	0.01	0.00	0.00	0.01	0.00	0.02	0.00	0.00	0.00	0.00	0.00	0.00	0.00	0.00
GT61	0.00	0.00	0.00	0.00	0.00	0.00	0.00	0.00	0.00	0.00	0.00	0.00	0.00	0.00	0.00	0.00
GT3	0.51	0.49	0.90	0.32	0.35	0.93	0.74	0.38	0.33	0.00	0.02	0.00	0.00	0.00	0.39	0.42
PL3	0.00	0.00	0.00	0.00	0.00	0.00	0.00	0.00	0.00	0.00	0.00	0.00	0.00	0.00	0.00	0.00
GH108	0.04	0.11	0.02	0.12	0.00	0.03	0.00	0.23	0.00	0.00	0.01	0.00	0.16	0.00	0.00	0.00
GT56	0.09	0.00	0.00	0.00	0.02	0.00	0.00	0.29	0.04	0.05	0.00	0.00	0.00	0.10	0.03	0.03
PL11	0.55	0.15	0.05	0.26	0.24	0.09	0.43	0.16	0.29	0.00	0.01	0.00	0.00	0.02	0.13	0.45
GH45	0.00	0.00	0.01	0.00	0.00	0.00	0.00	0.00	0.00	0.00	0.00	0.00	0.00	0.00	0.00	0.00
GT34	0.00	0.00	0.00	0.00	0.00	0.00	0.00	0.02	0.04	0.02	0.01	0.00	0.00	0.04	0.00	0.02
PL14	0.00	0.00	0.00	0.00	0.00	0.00	0.00	0.00	0.00	0.00	0.00	0.00	0.00	0.00	0.00	0.00
GH19	0.00	0.06	0.04	0.09	0.04	0.03	0.00	0.07	0.00	0.02	0.00	0.00	0.16	0.02	0.03	0.02
GH118	0.00	0.00	0.00	0.00	0.00	0.00	0.00	0.00	0.00	0.00	0.00	0.00	0.00	0.00	0.00	0.00
GH82	0.00	0.00	0.00	0.00	0.00	0.00	0.00	0.00	0.00	0.00	0.00	0.00	0.00	0.00	0.00	0.00
GH76	0.00	0.02	0.16	0.00	0.06	0.10	0.00	0.02	0.04	0.00	0.00	0.00	0.00	0.00	1.00	0.78
GT4	3.48	4.67	3.78	5.06	4.81	3.44	3.60	3.44	3.26	7.02	7.11	5.09	3.58	8.26	8.46	6.04
GT87	0.00	0.00	0.01	0.00	0.00	0.00	0.04	0.02	0.00	0.02	0.01	0.03	0.00	0.02	0.03	0.00
GH24	0.80	0.42	0.18	0.15	0.37	0.26	0.27	0.47	0.25	0.05	0.04	0.03	0.65	0.08	0.13	0.33
GH90	0.00	0.00	0.00	0.00	0.00	0.00	0.00	0.00	0.00	0.00	0.00	0.00	0.00	0.00	0.00	0.00
GH57	0.44	0.46	0.33	0.47	0.43	0.39	0.39	0.30	0.38	0.00	0.01	0.00	0.16	0.00	0.39	0.16
GH36	2.17	1.58	1.43	2.44	2.43	1.52	2.23	1.25	1.59	3.73	2.53	1.87	0.49	2.82	3.17	2.29
GT46	0.00	0.00	0.00	0.00	0.00	0.00	0.00	0.00	0.00	0.00	0.00	0.00	0.00	0.00	0.00	0.00
GH66	0.11	0.44	0.17	0.29	0.17	0.11	0.24	0.23	0.13	0.27	0.12	0.00	0.00	0.00	0.10	0.17

CAZY_group	VE-AD-2	VE-AD-3	VE-AD-4	VE-AD-5	VE-AD-6	VE-CH-1	VE-CH-2	VE-CH-3	VE-CH-4	VE-CH-5	VE-CH-6	VE-IN-1	VE-IN-2	VE-IN-3	VE-IN-4	VE-IN-5
GH48	0.00	0.02	0.02	0.03	0.02	0.00	0.00	0.02	0.04	0.00	0.00	0.00	0.00	0.00	0.00	0.00
GT53	0.00	0.00	0.00	0.00	0.00	0.00	0.00	0.00	0.00	0.00	0.00	0.00	0.00	0.00	0.00	0.00
GH60	0.00	0.00	0.00	0.00	0.00	0.00	0.00	0.00	0.00	0.00	0.00	0.00	0.00	0.00	0.00	0.00
GT31	0.00	0.00	0.00	0.00	0.00	0.00	0.04	0.09	0.00	0.00	0.01	0.00	0.00	0.00	0.00	0.00
GH105	1.53	1.20	0.31	0.93	1.30	0.36	0.90	0.54	0.79	0.03	0.06	0.06	0.00	0.00	0.92	0.99
GT23	0.00	0.00	0.00	0.00	0.00	0.00	0.00	0.00	0.00	0.00	0.00	0.00	0.00	0.00	0.00	0.00
GH11	0.00	0.02	0.01	0.03	0.00	0.01	0.00	0.00	0.00	0.00	0.00	0.00	0.00	0.00	0.00	0.00
GT38	0.00	0.00	0.00	0.00	0.00	0.00	0.00	0.00	0.00	0.00	0.00	0.00	0.00	0.00	0.00	0.00
GT43	0.00	0.00	0.00	0.00	0.00	0.00	0.00	0.00	0.00	0.00	0.00	0.00	0.00	0.00	0.00	0.00
GH99	0.04	0.00	0.00	0.03	0.00	0.02	0.00	0.04	0.00	0.00	0.00	0.00	0.00	0.00	0.00	0.02
GH38	0.02	0.15	0.59	0.35	0.39	0.46	0.16	0.30	0.33	0.64	3.22	2.72	1.79	6.21	1.73	0.71
GH109	0.84	0.34	0.31	0.35	0.35	0.43	0.74	0.21	0.17	0.00	0.01	0.00	0.00	0.00	0.73	0.33
GH2	9.79	9.97	6.44	10.07	10.22	6.24	11.12	8.14	7.11	7.86	7.54	4.05	3.74	7.13	17.39	10.75
GT28	0.84	0.99	0.84	0.96	1.21	1.02	1.10	1.07	0.50	1.35	1.54	1.04	0.49	1.88	1.07	1.30
GH42	0.24	0.40	0.18	0.32	0.32	0.27	0.43	0.13	0.46	3.38	4.22	3.86	1.79	5.94	0.65	1.82
GT58	0.00	0.00	0.00	0.00	0.00	0.00	0.00	0.00	0.00	0.00	0.00	0.00	0.00	0.00	0.00	0.00
GH61	0.00	0.00	0.00	0.00	0.00	0.00	0.00	0.00	0.00	0.00	0.00	0.00	0.00	0.00	0.00	0.00
GH80	0.00	0.00	0.00	0.00	0.00	0.00	0.00	0.00	0.00	0.00	0.00	0.00	0.00	0.00	0.00	0.00
GH98	0.02	0.00	0.00	0.00	0.06	0.01	0.04	0.00	0.04	0.00	0.00	0.00	0.00	0.00	0.08	0.05
GH6	0.00	0.00	0.02	0.00	0.00	0.00	0.00	0.00	0.00	0.00	0.00	0.00	0.00	0.00	0.00	0.00
GT1	0.11	0.08	0.07	0.00	0.09	0.08	0.08	0.11	0.17	0.18	0.05	0.00	0.49	0.06	0.16	0.05
GH28	2.11	2.05	1.07	2.74	2.23	1.09	2.23	1.52	1.30	0.53	0.66	0.28	0.65	0.58	1.39	2.23
GT42	0.00	0.00	0.00	0.00	0.00	0.00	0.00	0.00	0.00	0.00	0.00	0.00	0.00	0.00	0.00	0.00
GH128	0.00	0.04	0.00	0.03	0.00	0.00	0.00	0.00	0.00	0.00	0.00	0.00	0.00	0.00	0.00	0.00
CE12	0.36	0.38	0.08	0.35	0.22	0.16	0.39	0.39	0.17	0.02	0.01	0.00	0.00	0.02	0.10	0.19
CE16	0.00	0.00	0.00	0.00	0.00	0.00	0.00	0.00	0.00	0.00	0.00	0.00	0.00	0.00	0.00	0.00
GT89	0.00	0.00	0.00	0.00	0.00	0.00	0.00	0.00	0.00	0.00	0.00	0.00	0.00	0.00	0.00	0.00
GT11	0.11	0.02	0.04	0.06	0.13	0.03	0.04	0.05	0.00	0.00	0.04	0.00	0.00	0.00	0.16	0.02
GT77	0.16	0.06	0.10	0.06	0.02	0.00	0.00	0.32	0.17	0.54	0.12	0.03	6.01	0.27	0.08	0.02
GT55	0.00	0.00	0.00	0.00	0.00	0.00	0.00	0.00	0.00	0.00	0.00	0.00	0.00	0.00	0.00	0.00
GT18	0.00	0.00	0.00	0.00	0.00	0.00	0.00	0.00	0.00	0.00	0.00	0.00	0.00	0.00	0.00	0.00
GH87	0.02	0.00	0.04	0.06	0.00	0.02	0.00	0.00	0.00	0.16	0.02	0.00	0.00	0.00	0.00	0.03
PL5	0.00	0.00	0.00	0.00	0.00	0.00	0.00	0.00	0.00	0.00	0.00	0.00	0.00	0.00	0.00	0.00
GH22	0.00	0.00	0.00	0.00	0.00	0.00	0.00	0.02	0.00	0.00	0.00	0.00	0.00	0.00	0.00	0.00
CE2	0.09	0.25	0.09	0.32	0.13	0.10	0.31	0.05	0.21	0.25	0.24	0.00	0.16	0.00	0.05	0.29
GH120	0.02	0.15	0.01	0.03	0.09	0.09	0.20	0.07	0.21	0.44	0.12	0.22	0.00	0.00	0.05	0.03
GH73	1.58	1.31	0.67	1.05	1.60	0.84	1.88	1.32	1.21	2.19	1.02	0.73	0.33	0.71	0.52	0.99
GT17	0.02	0.00	0.01	0.00	0.00	0.01	0.00	0.00	0.00	0.00	0.00	0.00	0.00	0.00	0.00	0.00
GH124	0.00	0.00	0.00	0.00	0.00	0.00	0.00	0.00	0.00	0.00	0.00	0.00	0.00	0.00	0.00	0.00
GH54	0.00	0.00	0.00	0.00	0.00	0.00	0.00	0.00	0.00	0.00	0.00	0.00	0.00	0.00	0.00	0.00
GH100	0.00	0.00	0.00	0.00	0.00	0.00	0.00	0.00	0.00	0.00	0.00	0.00	0.00	0.00	0.00	0.00
GH113	0.07	0.06	0.03	0.03	0.00	0.02	0.00	0.04	0.04	0.00	0.00	0.00	0.00	0.00	0.08	0.00
GT5	0.62	0.84	0.94	0.90	1.04	0.86	0.71	0.75	1.51	0.50	0.36	0.51	0.33	0.13	1.10	0.74
GH94	0.67	1.16	0.51	1.75	1.34	0.61	1.06	0.86	1.26	0.06	0.07	0.03	0.00	0.00	0.10	0.31
GT66	0.02	0.00	0.05	0.00	0.00	0.04	0.00	0.00	0.00	0.00	0.00	0.00	0.00	0.00	0.00	0.02
GT94	0.00	0.00	0.00	0.00	0.00	0.00	0.00	0.00	0.00	0.00	0.00	0.00	0.00	0.00	0.00	0.00
GH58	0.00	0.00	0.00	0.00	0.00	0.01	0.00	0.04	0.00	0.00	0.00	0.00	0.00	0.02	0.00	0.00
GH1	0.69	1.01	0.85	1.46	1.36	0.70	1.10	1.72	1.26	4.89	2.38	0.60	0.98	1.78	1.13	2.56
PL22	0.07	0.00	0.00	0.00	0.00	0.00	0.00	0.02	0.00	0.01	0.00	0.00	0.00	0.00	0.00	0.00
GT9	0.49	0.44	0.08	0.29	0.41	0.19	0.55	0.77	0.29	0.18	0.06	0.06	0.33	0.10	0.16	0.64

CAZY_group	VE-AD-2	VE-AD-3	VE-AD-4	VE-AD-5	VE-AD-6	VE-CH-1	VE-CH-2	VE-CH-3	VE-CH-4	VE-CH-5	VE-CH-6	VE-IN-1	VE-IN-2	VE-IN-3	VE-IN-4	VE-IN-5
GT76	0.00	0.00	0.01	0.00	0.00	0.00	0.00	0.00	0.00	0.00	0.00	0.03	0.00	0.00	0.00	0.00
GT32	0.13	0.11	0.16	0.26	0.11	0.11	0.16	0.13	0.21	0.31	0.63	0.13	0.00	0.00	0.18	0.12
GT47	0.13	0.11	0.20	0.26	0.35	0.17	0.27	0.30	0.13	0.44	0.35	0.51	0.00	0.56	0.39	0.43
GH84	0.44	0.34	0.08	0.09	0.56	0.15	0.35	0.32	0.04	2.57	2.86	0.79	0.00	0.31	0.45	1.23
GH83	0.00	0.00	0.00	0.00	0.00	0.00	0.00	0.00	0.00	0.00	0.00	0.00	0.00	0.00	0.00	0.00
GT59	0.00	0.00	0.00	0.00	0.00	0.00	0.00	0.00	0.00	0.00	0.00	0.00	0.00	0.00	0.00	0.00
GH34	0.00	0.00	0.00	0.00	0.00	0.00	0.00	0.00	0.00	0.00	0.00	0.00	0.00	0.00	0.00	0.00
GT14	0.11	0.02	0.02	0.00	0.17	0.00	0.00	0.04	0.04	0.31	0.06	0.00	0.00	0.00	0.16	0.03
GH47	0.00	0.00	0.00	0.00	0.00	0.00	0.00	0.00	0.00	0.01	0.02	0.00	0.00	0.06	0.00	0.02
GH97	2.71	2.20	1.80	2.74	2.69	1.80	2.43	1.72	1.84	0.00	0.01	0.03	0.00	0.00	2.44	2.30
GH50	0.00	0.00	0.05	0.00	0.00	0.03	0.00	0.00	0.04	0.00	0.00	0.00	0.16	0.00	0.03	0.55
GT26	0.38	0.63	0.20	0.38	0.41	0.23	0.51	0.45	0.46	0.51	0.52	0.19	0.00	0.35	0.55	0.47
GH18	0.36	0.57	0.61	0.29	0.61	0.59	0.71	0.70	0.96	0.73	1.34	0.89	0.49	0.31	0.92	1.00
GH37	0.09	0.02	0.03	0.12	0.00	0.04	0.00	0.48	0.00	0.14	0.02	0.00	0.98	0.12	0.03	0.02
PL13	0.00	0.02	0.00	0.00	0.00	0.00	0.00	0.00	0.00	0.00	0.00	0.00	0.00	0.00	0.03	0.10
GH27	0.75	0.61	0.39	0.58	0.48	0.36	0.55	0.23	0.46	0.43	0.01	0.00	0.00	0.00	0.94	0.38
GT54	0.00	0.00	0.00	0.00	0.00	0.00	0.00	0.00	0.00	0.00	0.00	0.00	0.00	0.00	0.00	0.00
GT91	0.00	0.00	0.00	0.00	0.00	0.00	0.00	0.00	0.00	0.00	0.00	0.00	0.00	0.00	0.00	0.00
GT72	0.18	0.00	0.00	0.00	0.00	0.00	0.00	0.00	0.00	0.00	0.00	0.00	0.00	0.00	0.00	0.00
GH67	0.33	0.21	0.04	0.41	0.32	0.08	0.16	0.14	0.29	0.00	0.01	0.00	0.00	0.02	0.18	0.40
GH12	0.00	0.00	0.00	0.00	0.04	0.00	0.00	0.02	0.00	0.00	0.00	0.00	0.00	0.02	0.03	0.00
GH91	0.00	0.11	0.01	0.00	0.00	0.02	0.00	0.04	0.00	0.00	0.00	0.00	0.00	0.00	0.00	0.00
GT69	0.00	0.00	0.00	0.00	0.00	0.00	0.00	0.00	0.00	0.00	0.00	0.00	0.00	0.00	0.00	0.00
GH106	0.36	0.63	0.96	0.47	0.56	0.82	0.63	0.30	0.29	0.00	0.00	0.00	0.16	0.00	0.21	0.55
GT62	0.00	0.00	0.00	0.00	0.00	0.00	0.00	0.00	0.00	0.00	0.00	0.00	0.00	0.00	0.00	0.00
GH17	0.00	0.00	0.00	0.00	0.00	0.00	0.00	0.00	0.00	0.00	0.00	0.00	0.16	0.00	0.00	0.00
GH88	0.27	0.21	0.28	0.32	0.19	0.16	0.27	0.18	0.21	0.06	0.05	0.16	0.00	0.00	0.89	0.90
CE5	0.00	0.00	0.00	0.00	0.00	0.00	0.00	0.00	0.00	0.00	0.00	0.00	0.00	0.00	0.00	0.00
GT8	0.16	0.13	0.17	0.29	0.24	0.16	0.39	0.29	0.33	1.30	0.95	0.66	0.98	0.15	1.36	0.90
GT50	0.00	0.00	0.00	0.00	0.00	0.00	0.00	0.00	0.00	0.00	0.00	0.00	0.00	0.00	0.00	0.00
GT20	0.02	0.00	0.00	0.00	0.00	0.00	0.00	0.14	0.00	0.06	0.00	0.06	0.16	0.00	0.03	0.19
PL9	0.55	0.21	0.04	0.12	0.30	0.10	0.04	0.59	0.25	0.11	0.00	0.00	0.00	0.02	1.31	0.03
GH55	0.02	0.00	0.02	0.03	0.00	0.01	0.04	0.00	0.00	0.00	0.00	0.00	0.00	0.00	0.03	0.02
GH64	0.02	0.00	0.00	0.00	0.02	0.00	0.00	0.00	0.00	0.00	0.00	0.00	0.00	0.00	0.00	0.00
GH127	1.00	0.95	0.54	0.61	1.00	0.62	0.98	0.73	0.63	0.65	0.05	0.22	0.00	0.08	1.91	0.48
GH103	0.00	0.00	0.01	0.03	0.00	0.00	0.00	0.27	0.00	0.06	0.01	0.06	0.16	0.08	0.00	0.07
GH3	5.61	6.44	4.36	6.43	5.70	4.82	6.50	4.89	4.56	4.46	3.81	3.64	1.14	3.05	11.68	7.19
GT45	0.00	0.00	0.00	0.00	0.00	0.00	0.00	0.00	0.00	0.00	0.00	0.00	0.00	0.00	0.00	0.00
GT74	0.00	0.00	0.00	0.00	0.00	0.00	0.00	0.00	0.00	0.00	0.00	0.00	0.00	0.00	0.00	0.00
GT90	0.00	0.02	0.02	0.00	0.00	0.00	0.00	0.00	0.00	0.00	0.00	0.00	0.00	0.00	0.00	0.00
GH116	0.00	0.02	0.01	0.12	0.02	0.04	0.04	0.00	0.00	0.00	0.00	0.00	0.00	0.00	0.00	0.00
GH31	2.80	3.00	1.44	3.17	2.97	1.40	2.51	2.99	2.51	2.41	1.56	1.83	0.16	0.33	4.30	3.12
GT12	0.00	0.00	0.00	0.00	0.00	0.00	0.00	0.00	0.00	0.00	0.00	0.00	0.16	0.00	0.00	0.00
CE1	0.58	0.65	0.54	0.90	0.67	0.59	1.06	0.77	0.21	0.51	0.21	0.19	0.16	0.33	0.58	0.69
GH121	0.00	0.00	0.00	0.03	0.00	0.04	0.04	0.00	0.29	0.94	0.02	0.54	0.00	0.06	0.05	0.47
GH79	0.07	0.00	0.00	0.00	0.02	0.00	0.04	0.02	0.04	0.01	0.00	0.06	0.00	0.00	0.03	0.03
GT73	0.02	0.00	0.00	0.00	0.00	0.00	0.00	0.02	0.00	0.00	0.02	0.00	0.00	0.00	0.00	0.00
GH77	1.95	2.18	1.57	1.46	1.80	1.28	2.04	1.61	2.55	2.64	2.71	3.92	1.46	5.62	2.41	2.06
PL17	0.00	0.06	0.00	0.00	0.02	0.00	0.00	0.00	0.00	0.00	0.00	0.00	0.00	0.00	0.00	0.00
PL8	0.29	0.23	0.16	0.06	0.11	0.19	0.16	0.11	0.17	0.01	0.00	0.00	0.00	0.00	0.58	0.73

CAZY_group	VE-AD-2	VE-AD-3	VE-AD-4	VE-AD-5	VE-AD-6	VE-CH-1	VE-CH-2	VE-CH-3	VE-CH-4	VE-CH-5	VE-CH-6	VE-IN-1	VE-IN-2	VE-IN-3	VE-IN-4	VE-IN-5
CE9	0.49	0.65	0.46	0.61	0.80	0.48	0.51	1.04	0.71	1.58	1.43	1.55	0.65	1.50	1.20	1.59
CE15	0.00	0.02	0.06	0.00	0.02	0.05	0.00	0.00	0.00	0.00	0.00	0.00	0.00	0.00	0.03	0.03
GT79	0.00	0.00	0.00	0.00	0.00	0.00	0.00	0.00	0.00	0.00	0.00	0.00	0.00	0.00	0.00	0.00
GT10	0.00	0.00	0.05	0.00	0.00	0.07	0.00	0.00	0.00	0.00	0.00	0.00	0.00	0.00	0.16	0.00
GH119	0.00	0.00	0.01	0.00	0.00	0.00	0.00	0.00	0.00	0.00	0.00	0.00	0.00	0.00	0.00	0.00
GH16	0.29	0.23	0.36	0.49	0.35	0.31	0.51	0.23	0.17	0.09	0.15	0.03	0.16	0.04	0.94	0.42
GH43	4.90	6.04	2.06	5.30	5.35	2.59	5.29	3.94	2.64	7.70	1.47	2.88	0.16	1.15	6.34	5.71
GH51	1.60	2.18	0.52	2.24	1.58	0.70	1.80	0.89	1.13	2.98	2.35	1.83	0.33	2.40	2.44	2.15
GT35	2.02	2.66	1.80	2.27	2.43	1.55	2.08	2.27	2.55	3.03	2.25	2.62	1.14	3.66	2.02	2.30
GT25	0.00	0.00	0.00	0.00	0.02	0.00	0.04	0.00	0.00	0.00	0.02	0.00	0.00	0.00	0.00	0.00
GT2	11.87	12.12	8.19	10.97	11.76	7.83	9.79	11.01	11.42	15.29	18.84	16.63	9.10	21.09	15.09	15.06
GT51	3.04	2.81	1.80	2.68	2.88	1.86	2.98	3.17	2.34	5.24	4.58	3.51	2.93	6.08	3.14	3.84
GH107	0.00	0.00	0.00	0.00	0.00	0.00	0.00	0.00	0.00	0.00	0.00	0.00	0.00	0.00	0.00	0.00
GH7	0.00	0.00	0.00	0.00	0.00	0.00	0.00	0.00	0.00	0.00	0.00	0.00	0.00	0.00	0.00	0.00
GT7	0.00	0.00	0.00	0.00	0.00	0.00	0.00	0.00	0.00	0.00	0.00	0.00	0.00	0.00	0.00	0.00
GT36	0.00	0.00	0.00	0.00	0.00	0.00	0.00	0.00	0.00	0.00	0.00	0.00	0.00	0.00	0.00	0.00
PL7	0.00	0.00	0.02	0.00	0.00	0.01	0.00	0.00	0.00	0.00	0.00	0.00	0.00	0.00	0.00	0.00
GH110	0.02	0.25	0.21	0.00	0.22	0.08	0.04	0.16	0.00	0.74	0.98	0.16	0.00	0.15	1.07	0.43
GT21	0.00	0.00	0.00	0.00	0.00	0.00	0.00	0.00	0.00	0.00	0.00	0.00	0.00	0.00	0.00	0.00
GH62	0.00	0.00	0.00	0.00	0.00	0.00	0.00	0.00	0.04	0.00	0.00	0.00	0.00	0.00	0.00	0.00
GH125	0.27	0.38	0.27	0.20	0.43	0.25	0.43	0.23	0.17	0.26	0.58	0.89	0.00	1.36	0.86	0.57
GH117	0.07	0.02	0.04	0.00	0.04	0.04	0.12	0.02	0.08	0.00	0.00	0.00	0.00	0.00	0.45	0.17
GH68	0.00	0.00	0.00	0.00	0.00	0.00	0.00	0.00	0.00	0.14	0.25	0.00	0.00	0.04	0.00	0.14
GH85	0.67	0.36	0.03	0.06	0.24	0.12	0.63	0.29	0.25	0.13	0.09	0.89	0.65	0.25	0.10	0.28
GH92	2.20	2.01	3.10	1.80	1.82	2.57	2.70	1.95	1.59	0.08	0.34	0.32	0.49	0.65	5.42	3.79
GH40	0.00	0.00	0.00	0.00	0.00	0.00	0.00	0.00	0.00	0.00	0.00	0.00	0.00	0.00	0.00	0.00
GT49	0.00	0.00	0.01	0.00	0.00	0.00	0.00	0.00	0.00	0.00	0.00	0.00	0.00	0.00	0.00	0.00
GH112	0.24	0.42	0.07	0.55	0.52	0.21	0.39	0.38	0.71	1.65	2.23	2.06	0.49	2.68	0.63	1.06
GT67	0.00	0.00	0.00	0.00	0.00	0.00	0.00	0.00	0.00	0.00	0.00	0.00	0.00	0.00	0.00	0.00
CE8	0.67	0.59	0.07	0.81	0.61	0.21	0.78	0.77	0.50	0.17	0.14	0.06	0.33	0.06	0.31	0.35
GT86	0.00	0.00	0.00	0.00	0.00	0.00	0.00	0.00	0.00	0.00	0.00	0.00	0.00	0.00	0.00	0.00
GH101	0.02	0.00	0.00	0.06	0.00	0.04	0.04	0.13	0.00	1.67	1.84	0.60	0.00	0.29	0.08	0.68
GH81	0.00	0.02	0.01	0.00	0.00	0.00	0.00	0.00	0.00	0.00	0.00	0.00	0.00	0.00	0.03	0.03
GT93	0.00	0.00	0.00	0.00	0.00	0.00	0.00	0.00	0.00	0.00	0.00	0.00	0.00	0.00	0.00	0.00
GT63	0.00	0.00	0.00	0.00	0.00	0.00	0.00	0.00	0.00	0.00	0.00	0.00	0.00	0.00	0.00	0.00
CE11	1.04	0.76	0.30	0.55	0.50	0.38	0.59	0.79	0.46	0.27	0.15	0.00	0.16	0.04	0.39	0.40
GT19	0.42	0.44	0.33	0.44	0.28	0.21	0.35	0.45	0.13	0.02	0.02	0.00	0.33	0.10	0.52	0.45
GT33	0.00	0.00	0.00	0.00	0.00	0.00	0.00	0.00	0.00	0.00	0.00	0.00	0.00	0.00	0.00	0.00
GH21	0.00	0.00	0.00	0.00	0.00	0.00	0.00	0.00	0.00	0.00	0.00	0.00	0.00	0.00	0.00	0.00
GT68	0.00	0.00	0.00	0.00	0.00	0.00	0.00	0.00	0.00	0.00	0.00	0.00	0.00	0.00	0.00	0.00
GH89	0.40	0.32	0.59	0.23	0.35	0.38	0.59	0.25	0.17	1.33	1.42	0.41	0.00	0.21	0.94	1.45
GT82	0.00	0.00	0.00	0.00	0.00	0.00	0.00	0.00	0.00	0.00	0.00	0.00	0.00	0.00	0.00	0.00
GH30	0.31	0.21	0.24	0.26	0.06	0.26	0.24	0.13	0.13	0.85	0.42	0.73	0.00	0.10	0.47	0.90
GH20	2.42	2.20	1.93	1.43	2.58	2.00	1.88	1.83	1.38	4.41	7.60	4.58	1.95	8.01	5.24	5.09
GH52	0.00	0.00	0.00	0.00	0.00	0.00	0.00	0.00	0.00	0.00	0.00	0.00	0.00	0.00	0.00	0.00
GT84	0.02	0.04	0.09	0.00	0.02	0.03	0.00	0.00	0.04	0.00	0.00	0.00	0.00	0.00	0.00	0.00
GT15	0.00	0.00	0.00	0.00	0.00	0.00	0.00	0.00	0.00	0.00	0.00	0.00	0.00	0.00	0.00	0.00
GH93	0.00	0.00	0.01	0.00	0.00	0.01	0.00	0.02	0.00	0.01	0.00	0.00	0.00	0.00	0.00	0.00
PL16	0.00	0.00	0.00	0.00	0.00	0.00	0.00	0.00	0.00	0.00	0.00	0.00	0.00	0.00	0.00	0.00
GH26	0.38	0.46	0.14	0.64	0.45	0.27	0.55	0.32	0.63	0.01	0.02	0.00	0.65	0.00	0.60	0.12

CAZY_group	VE-AD-2	VE-AD-3	VE-AD-4	VE-AD-5	VE-AD-6	VE-CH-1	VE-CH-2	VE-CH-3	VE-CH-4	VE-CH-5	VE-CH-6	VE-IN-1	VE-IN-2	VE-IN-3	VE-IN-4	VE-IN-5
GT75	0.00	0.00	0.00	0.00	0.00	0.00	0.00	0.00	0.00	0.00	0.00	0.00	0.00	0.00	0.00	0.00
GH53	0.44	0.36	0.20	0.55	0.65	0.13	0.51	0.43	0.42	0.56	0.52	1.30	0.33	0.92	0.26	0.55
GT88	0.00	0.00	0.00	0.00	0.00	0.00	0.00	0.00	0.00	0.00	0.00	0.00	0.00	0.00	0.00	0.00
GT24	0.00	0.00	0.00	0.00	0.00	0.00	0.00	0.00	0.00	0.00	0.00	0.00	0.00	0.00	0.00	0.00
GT81	0.00	0.00	0.00	0.00	0.00	0.00	0.00	0.00	0.00	0.00	0.00	0.00	0.00	0.00	0.00	0.00
GH4	0.31	0.21	0.24	0.32	0.24	0.20	0.20	0.79	0.29	0.56	0.75	0.22	1.14	0.17	0.18	0.48
GT78	0.00	0.00	0.00	0.00	0.00	0.00	0.00	0.00	0.00	0.00	0.00	0.00	0.00	0.00	0.00	0.00
GH8	0.16	0.21	0.01	0.23	0.06	0.07	0.04	0.29	0.04	0.32	0.17	0.03	0.49	0.12	0.05	0.19
GH70	0.00	0.17	0.00	0.06	0.00	0.00	0.00	0.04	0.00	1.05	0.11	0.00	0.16	0.36	0.00	0.55
GH122	0.00	0.00	0.00	0.00	0.00	0.00	0.00	0.00	0.00	0.00	0.00	0.00	0.00	0.00	0.00	0.00
GH74	0.00	0.04	0.04	0.06	0.06	0.04	0.08	0.05	0.04	0.00	0.00	0.00	0.00	0.00	0.00	0.00
GT41	0.09	0.00	0.03	0.06	0.02	0.00	0.00	0.02	0.04	0.00	0.04	0.00	0.16	0.00	0.00	0.03
PL1	0.64	0.63	0.24	0.61	0.52	0.46	0.35	0.38	0.54	0.00	0.00	0.00	0.00	0.00	0.18	0.71
GH15	0.00	0.00	0.00	0.00	0.00	0.00	0.00	0.00	0.00	0.00	0.00	0.00	0.00	0.00	0.08	0.12
GH32	1.49	1.92	0.56	2.12	1.56	0.84	1.65	1.27	1.34	2.34	1.57	1.83	1.79	3.09	2.04	1.39
GH44	0.00	0.02	0.03	0.06	0.02	0.00	0.04	0.00	0.04	0.00	0.00	0.00	0.00	0.00	0.00	0.00
GT6	0.02	0.00	0.00	0.00	0.04	0.00	0.00	0.00	0.00	0.00	0.00	0.00	0.00	0.00	0.00	0.00
GH13	6.79	9.17	6.00	9.26	9.68	6.08	8.15	7.95	8.33	17.24	14.52	12.14	6.01	19.67	8.98	9.18
GT80	0.07	0.00	0.03	0.03	0.04	0.02	0.00	0.16	0.00	0.05	0.00	0.00	0.33	0.02	0.00	0.02
GH71	0.00	0.00	0.00	0.00	0.00	0.00	0.00	0.00	0.00	0.00	0.00	0.00	0.00	0.00	0.00	0.00
GH63	0.42	0.27	0.11	0.15	0.22	0.17	0.12	0.47	0.13	0.13	0.00	0.00	0.33	0.12	0.45	0.12
PL21	0.00	0.00	0.06	0.00	0.00	0.04	0.00	0.00	0.00	0.00	0.00	0.00	0.00	0.00	0.03	0.00
GH65	0.04	0.11	0.31	0.20	0.15	0.31	0.47	0.27	0.17	1.26	0.71	0.06	0.33	0.12	0.55	0.47
CE4	0.73	0.87	0.99	0.99	0.91	1.03	1.18	0.68	1.34	0.83	0.60	0.51	0.49	0.12	0.73	0.88
CE14	0.00	0.02	0.02	0.03	0.00	0.03	0.00	0.09	0.00	0.01	0.04	0.00	0.00	0.00	0.05	0.03
GH115	0.69	0.97	0.16	0.84	0.65	0.24	1.14	0.79	0.46	0.00	0.02	0.00	0.00	0.00	0.89	1.39
GH104	0.00	0.00	0.01	0.00	0.04	0.00	0.00	0.11	0.04	0.00	0.01	0.00	0.33	0.00	0.00	0.00
GH111	0.00	0.00	0.01	0.00	0.00	0.00	0.00	0.00	0.00	0.00	0.00	0.03	0.00	0.00	0.00	0.00
GH95	2.09	2.22	0.82	1.34	2.23	1.17	1.96	1.34	1.21	2.36	2.76	1.52	0.81	3.07	3.09	2.44
GT70	0.00	0.00	0.00	0.00	0.00	0.00	0.00	0.00	0.00	0.00	0.00	0.00	0.00	0.00	0.00	0.00
CE10	0.49	0.32	0.32	0.20	0.39	0.24	0.39	0.41	0.25	0.40	0.40	0.35	0.65	0.61	0.71	0.42
GH10	0.36	0.46	0.07	0.64	0.76	0.10	0.39	0.29	0.29	0.00	0.00	0.03	0.00	0.00	0.21	0.35
GH130	0.40	0.49	0.32	0.64	0.41	0.34	0.78	0.25	0.50	0.00	0.00	0.00	0.00	0.00	0.45	0.80
PL20	0.00	0.00	0.00	0.00	0.00	0.00	0.00	0.00	0.00	0.00	0.00	0.00	0.00	0.00	0.00	0.00
GH69	0.00	0.00	0.00	0.00	0.00	0.00	0.00	0.00	0.00	0.00	0.00	0.00	0.00	0.00	0.00	0.00
PL2	0.00	0.00	0.00	0.00	0.00	0.00	0.00	0.02	0.00	0.00	0.00	0.00	0.00	0.00	0.00	0.00
GH49	0.00	0.00	0.00	0.00	0.00	0.00	0.00	0.00	0.00	0.00	0.00	0.00	0.00	0.00	0.00	0.00
GH25	0.98	1.54	0.61	1.40	2.43	0.91	1.18	1.13	0.88	4.32	3.41	4.62	0.33	0.92	1.18	1.94
GH59	0.02	0.00	0.01	0.03	0.00	0.01	0.00	0.02	0.00	0.00	0.00	0.00	0.00	0.00	0.00	0.00
GH35	0.71	0.72	0.62	0.99	0.45	0.60	0.67	0.68	0.59	0.31	0.47	0.38	0.33	0.40	2.41	0.88
GT39	0.00	0.06	0.04	0.09	0.00	0.02	0.00	0.02	0.00	0.00	0.01	0.00	0.00	0.02	0.00	0.00
GT57	0.00	0.00	0.00	0.00	0.00	0.00	0.00	0.00	0.00	0.00	0.00	0.00	0.00	0.00	0.00	0.00
GH129	0.00	0.00	0.00	0.03	0.04	0.01	0.00	0.00	0.00	0.85	1.59	1.04	0.33	1.53	0.24	0.64
GT22	0.00	0.00	0.00	0.00	0.00	0.00	0.00	0.00	0.00	0.00	0.00	0.00	0.00	0.00	0.00	0.00
PL12	0.02	0.04	0.05	0.00	0.00	0.03	0.00	0.04	0.04	0.00	0.00	0.00	0.00	0.00	0.13	0.31
PL18	0.00	0.00	0.00	0.00	0.00	0.00	0.00	0.00	0.00	0.00	0.00	0.00	0.00	0.00	0.00	0.00
GT92	0.00	0.00	0.00	0.00	0.00	0.00	0.00	0.00	0.00	0.00	0.00	0.00	0.00	0.00	0.00	0.00
GT44	0.00	0.02	0.00	0.00	0.00	0.00	0.00	0.00	0.00	0.00	0.00	0.00	0.00	0.00	0.00	0.00
GT29	0.00	0.00	0.00	0.00	0.00	0.00	0.00	0.00	0.00	0.00	0.00	0.00	0.00	0.00	0.00	0.00
GT13	0.00	0.00	0.00	0.00	0.00	0.00	0.00	0.00	0.00	0.00	0.00	0.00	0.00	0.00	0.00	0.00

Normalized_abundance_2

CAZY_group	VE-AD-2	VE-AD-3	VE-AD-4	VE-AD-5	VE-AD-6	VE-CH-1	VE-CH-2	VE-CH-3	VE-CH-4	VE-CH-5	VE-CH-6	VE-IN-1	VE-IN-2	VE-IN-3	VE-IN-4	VE-IN-5
**GT52**	0.00	0.00	0.00	0.00	0.00	0.00	0.00	0.00	0.00	0.00	0.00	0.00	0.00	0.00	0.00	0.00
**GT64**	0.00	0.00	0.00	0.00	0.00	0.00	0.00	0.00	0.00	0.00	0.00	0.00	0.00	0.00	0.00	0.00
**GT85**	0.00	0.00	0.00	0.00	0.00	0.00	0.00	0.00	0.04	0.00	0.00	0.00	0.00	0.00	0.00	0.00
**GT16**	0.00	0.00	0.00	0.00	0.00	0.00	0.00	0.00	0.00	0.00	0.00	0.00	0.00	0.00	0.00	0.00
**GH56**	0.00	0.00	0.00	0.00	0.00	0.00	0.00	0.00	0.00	0.00	0.00	0.00	0.16	0.00	0.00	0.00
**GT60**	0.00	0.00	0.00	0.00	0.00	0.00	0.00	0.00	0.00	0.00	0.00	0.00	0.00	0.00	0.00	0.00
**GH41**	0.00	0.00	0.00	0.00	0.00	0.00	0.00	0.00	0.00	0.00	0.00	0.00	0.00	0.00	0.00	0.00
**CE7**	0.33	0.25	0.09	0.49	0.22	0.11	0.35	0.13	0.13	0.03	0.00	0.00	0.00	0.02	0.21	0.42
**GH39**	0.04	0.06	0.05	0.06	0.04	0.02	0.00	0.00	0.00	0.17	0.00	0.00	0.16	0.00	0.13	0.02
**GT30**	0.69	0.46	0.33	0.70	0.78	0.28	0.51	0.64	0.25	0.65	0.81	0.44	0.16	1.00	1.00	1.14
**PL15**	0.07	0.06	0.00	0.00	0.04	0.00	0.00	0.04	0.00	0.00	0.00	0.00	0.00	0.00	0.10	0.42
**GH102**	0.13	0.08	0.02	0.17	0.02	0.04	0.08	0.25	0.04	0.16	0.09	0.00	0.16	0.04	0.10	0.12

CAZY_group	VE-IN-6	VE-IN-7	VE-IN-8
GH29	4.07	3.06	3.19
PL4	0.00	0.00	0.00
GH23	3.39	2.77	3.19
CE6	0.08	0.00	0.00
GH72	0.37	0.44	0.70
GH114	0.00	0.00	0.00
GH78	2.12	0.89	0.10
PL19	0.00	0.00	0.00
GT27	0.00	0.00	0.00
GH5	1.06	1.73	1.74
GT40	0.00	0.00	0.00
CE13	0.00	0.00	0.00
GT37	0.00	0.00	0.00
GH33	4.78	5.44	3.97
GT65	0.00	0.00	0.00
GH86	0.02	0.00	0.00
GH123	0.31	0.00	0.00
GH96	0.00	0.00	0.00
GH14	0.00	0.00	0.00
CE3	0.00	0.00	0.00
PL10	0.12	0.00	0.00
GT48	0.00	0.00	0.00
PL6	0.00	0.00	0.00
GT83	0.94	0.07	0.49
GH126	0.00	0.00	0.02
GH9	0.02	0.02	0.00
GH75	0.00	0.00	0.00
GT71	0.00	0.00	0.00
GH46	0.00	0.00	0.00
GT61	0.00	0.00	0.00
GT3	0.27	0.00	0.00
PL3	0.00	0.00	0.00
GH108	0.02	0.00	0.00
GT56	0.00	0.07	0.14
PL11	0.25	0.00	0.00
GH45	0.00	0.00	0.00
GT34	0.00	0.03	0.02
PL14	0.00	0.00	0.00
GH19	0.00	0.00	0.08
GH118	0.00	0.00	0.00
GH82	0.00	0.00	0.00
GH76	0.67	0.00	0.02
GT4	6.58	7.47	8.35
GT87	0.00	0.02	0.00
GH24	0.25	0.07	0.27
GH90	0.00	0.00	0.00
GH57	0.16	0.00	0.00
GH36	2.96	4.11	2.82
GT46	0.00	0.00	0.00
GH66	0.12	0.02	0.31

CAZY_group	VE-IN-6	VE-IN-7	VE-IN-8
GH48	0.00	0.00	0.00
GT53	0.00	0.00	0.00
GH60	0.00	0.00	0.00
GT31	0.00	0.00	0.00
GH105	0.86	0.08	0.08
GT23	0.00	0.00	0.00
GH11	0.00	0.00	0.00
GT38	0.00	0.00	0.00
GT43	0.00	0.00	0.00
GH99	0.02	0.00	0.00
GH38	3.25	2.84	4.03
GH109	0.45	0.02	0.04
GH2	13.53	10.63	5.42
GT28	1.41	1.80	1.74
GH42	2.23	5.94	4.25
GT58	0.00	0.00	0.00
GH61	0.00	0.00	0.00
GH80	0.00	0.00	0.00
GH98	0.00	0.00	0.00
GH6	0.00	0.00	0.00
GT1	0.14	0.13	0.02
GH28	1.47	0.92	0.65
GT42	0.00	0.00	0.00
GH128	0.00	0.00	0.00
CE12	0.25	0.02	0.00
CE16	0.00	0.00	0.00
GT89	0.00	0.00	0.00
GT11	0.04	0.00	0.00
GT77	0.10	0.10	0.88
GT55	0.00	0.00	0.00
GT18	0.00	0.00	0.00
GH87	0.00	0.03	0.18
PL5	0.00	0.00	0.00
GH22	0.00	0.00	0.00
CE2	0.10	0.30	0.06
GH120	0.10	0.05	0.16
GH73	1.16	0.91	1.17
GT17	0.00	0.00	0.00
GH124	0.00	0.00	0.00
GH54	0.00	0.00	0.00
GH100	0.00	0.00	0.00
GH113	0.00	0.00	0.00
GT5	0.65	0.25	0.31
GH94	0.10	0.45	0.14
GT66	0.00	0.00	0.00
GT94	0.00	0.00	0.00
GH58	0.00	0.00	0.00
GH1	0.96	3.78	2.23
PL22	0.00	0.00	0.06
GT9	0.45	0.27	1.02

CAZY_group	VE-IN-6	VE-IN-7	VE-IN-8
GT76	0.00	0.00	0.00
GT32	0.20	0.39	0.80
GT47	0.49	0.49	0.55
GH84	2.10	1.63	1.08
GH83	0.00	0.00	0.00
GT59	0.00	0.00	0.00
GH34	0.00	0.00	0.00
GT14	0.06	0.32	0.57
GH47	0.02	0.05	0.02
GH97	2.02	0.00	0.00
GH50	0.04	0.00	0.00
GT26	0.29	0.54	0.68
GH18	0.96	0.77	0.49
GH37	0.04	0.02	0.33
PL13	0.02	0.00	0.00
GH27	0.31	0.02	0.00
GT54	0.00	0.00	0.00
GT91	0.00	0.00	0.00
GT72	0.00	0.00	0.00
GH67	0.08	0.00	0.00
GH12	0.02	0.00	0.00
GH91	0.00	0.02	0.02
GT69	0.00	0.00	0.00
GH106	0.78	0.00	0.00
GT62	0.00	0.00	0.00
GH17	0.00	0.00	0.00
GH88	0.33	0.02	0.02
CE5	0.00	0.00	0.00
GT8	0.37	0.30	3.76
GT50	0.00	0.00	0.00
GT20	0.14	0.02	0.00
PL9	0.12	0.07	0.14
GH55	0.02	0.05	0.00
GH64	0.00	0.00	0.00
GH127	1.41	0.44	0.10
GH103	0.06	0.07	0.12
GH3	6.70	7.66	3.74
GT45	0.00	0.00	0.00
GT74	0.00	0.00	0.00
GT90	0.00	0.00	0.00
GH116	0.00	0.00	0.00
GH31	2.98	1.13	1.84
GT12	0.00	0.00	0.00
CE1	0.59	0.22	0.76
GH121	0.00	0.20	0.14
GH79	0.00	0.00	0.00
GT73	0.00	0.00	0.06
GH77	3.21	4.35	3.23
PL17	0.00	0.00	0.00
PL8	0.74	0.07	0.04

CAZY_group	VE-IN-6	VE-IN-7	VE-IN-8
CE9	1.53	1.95	0.98
CE15	0.00	0.00	0.00
GT79	0.00	0.00	0.00
GT10	0.02	0.00	0.00
GH119	0.00	0.00	0.00
GH16	0.41	0.05	0.10
GH43	5.09	2.22	1.92
GH51	2.33	2.03	1.90
GT35	2.31	4.00	2.88
GT25	0.00	0.00	0.02
GT2	17.47	21.75	19.29
GT51	4.45	5.29	5.11
GH107	0.00	0.00	0.00
GH7	0.00	0.00	0.00
GT7	0.00	0.00	0.00
GT36	0.00	0.00	0.00
PL7	0.00	0.02	0.00
GH110	0.96	0.67	0.39
GT21	0.00	0.00	0.00
GH62	0.00	0.00	0.00
GH125	0.55	0.79	0.78
GH117	0.22	0.00	0.00
GH68	0.00	0.00	0.14
GH85	0.59	0.12	0.14
GH92	5.27	0.45	0.41
GH40	0.00	0.00	0.00
GT49	0.00	0.00	0.00
GH112	1.57	2.50	1.96
GT67	0.00	0.00	0.00
CE8	0.16	0.05	0.16
GT86	0.00	0.00	0.00
GH101	1.14	1.09	0.86
GH81	0.00	0.00	0.00
GT93	0.00	0.00	0.00
GT63	0.00	0.00	0.00
CE11	0.27	0.15	0.18
GT19	0.31	0.13	0.25
GT33	0.00	0.00	0.00
GH21	0.00	0.00	0.00
GT68	0.00	0.00	0.00
GH89	1.53	0.76	0.76
GT82	0.00	0.00	0.00
GH30	0.61	0.62	0.18
GH20	8.17	6.90	6.01
GH52	0.00	0.00	0.00
GT84	0.00	0.00	0.00
GT15	0.00	0.00	0.00
GH93	0.00	0.00	0.00
PL16	0.00	0.00	0.00
GH26	0.20	0.00	0.00

CAZY_group	VE-IN-6	VE-IN-7	VE-IN-8
GT75	0.00	0.00	0.00
GH53	0.33	0.67	0.78
GT88	0.00	0.00	0.00
GT24	0.00	0.00	0.00
GT81	0.00	0.00	0.00
GH4	0.22	0.57	0.80
GT78	0.00	0.00	0.00
GH8	0.06	0.08	0.20
GH70	0.02	0.24	1.15
GH122	0.00	0.00	0.00
GH74	0.00	0.00	0.00
GT41	0.00	0.00	0.02
PL1	0.57	0.00	0.00
GH15	0.16	0.00	0.00
GH32	1.57	2.65	2.99
GH44	0.00	0.00	0.00
GT6	0.02	0.00	0.00
GH13	11.32	23.02	14.36
GT80	0.00	0.03	0.08
GH71	0.00	0.00	0.00
GH63	0.14	0.08	0.10
PL21	0.00	0.00	0.00
GH65	0.20	1.18	0.41
CE4	0.86	0.42	0.86
CE14	0.02	0.03	0.10
GH115	0.94	0.00	0.02
GH104	0.00	0.03	0.00
GH111	0.00	0.00	0.00
GH95	3.60	2.77	2.39
GT70	0.00	0.00	0.00
CE10	0.57	0.76	0.41
GH10	0.10	0.00	0.02
GH130	0.59	0.00	0.00
PL20	0.00	0.00	0.00
GH69	0.00	0.00	0.00
PL2	0.00	0.00	0.00
GH49	0.00	0.00	0.00
GH25	2.23	2.40	2.39
GH59	0.00	0.00	0.00
GH35	0.98	0.39	0.45
GT39	0.04	0.02	0.00
GT57	0.00	0.00	0.00
GH129	0.92	1.70	1.19
GT22	0.00	0.00	0.00
PL12	0.16	0.02	0.00
PL18	0.00	0.00	0.00
GT92	0.00	0.00	0.00
GT44	0.00	0.00	0.00
GT29	0.00	0.00	0.00
GT13	0.00	0.00	0.00

CAZY_group	VE-IN-6	VE-IN-7	VE-IN-8
GT52	0.00	0.00	0.00
GT64	0.00	0.00	0.00
GT85	0.00	0.00	0.00
GT16	0.00	0.00	0.00
GH56	0.00	0.00	0.00
GT60	0.00	0.00	0.00
GH41	0.00	0.00	0.00
CE7	0.20	0.03	0.00
GH39	0.00	0.05	0.06
GT30	0.94	0.81	0.61
PL15	0.18	0.00	0.00
GH102	0.06	0.08	0.12

CAZY_group	CN-AD-1	CN-AD-2	CN-AD-3	CN-AD-4	CN-AD-5	CN-AD-6	CN-AD-7	CN-AD-8	CN-AD-9	CN-AD-10	CN-AD-11	CN-AD-12	CN-AD-13	CN-AD-14	CN-AD-15	CN-AD-16	CN-AD-17	CN-AD-18	CN-AD-19	CN-AD-20	CN-AD-21
**GH29**	0.46	0.59	0.51	0.95	0.70	0.49	0.59	0.70	0.23	0.44	0.20	0.44	0.27	0.53	0.58	1.06	0.66	0.53	0.80	1.12	0.26
**PL4**	0.00	0.00	0.00	0.00	0.00	0.00	0.00	0.00	0.00	0.00	0.00	0.00	0.00	0.00	0.00	0.00	0.00	0.00	0.00	0.06	0.03
**GH23**	1.07	1.24	1.07	0.87	1.13	1.46	0.78	0.85	1.14	0.98	0.81	1.00	0.79	0.84	1.02	1.00	0.79	1.14	0.90	1.18	1.01
**CE6**	0.05	0.02	0.09	0.00	0.03	0.02	0.02	0.04	0.00	0.05	0.02	0.06	0.02	0.02	0.06	0.02	0.04	0.02	0.04	0.06	0.00
**GH72**	0.03	0.09	0.14	0.05	0.10	0.09	0.02	0.02	0.05	0.05	0.11	0.08	0.06	0.03	0.11	0.06	0.02	0.11	0.07	0.04	0.10
**GH114**	0.00	0.00	0.00	0.00	0.00	0.00	0.00	0.00	0.00	0.00	0.00	0.00	0.00	0.01	0.00	0.00	0.00	0.00	0.00	0.00	0.00
**GH78**	1.17	0.90	0.49	0.64	0.30	0.79	1.11	0.92	0.66	0.54	0.09	0.70	0.17	0.61	0.76	0.87	1.01	0.70	1.03	1.25	0.33
**PL19**	0.00	0.00	0.00	0.00	0.00	0.00	0.00	0.00	0.00	0.00	0.00	0.00	0.00	0.00	0.00	0.00	0.00	0.00	0.00	0.00	0.00
**GT27**	0.00	0.00	0.00	0.02	0.00	0.00	0.00	0.00	0.00	0.00	0.00	0.00	0.00	0.00	0.00	0.00	0.00	0.00	0.00	0.00	0.00
**GH5**	0.08	0.38	0.44	0.34	0.78	0.31	0.26	0.26	0.16	0.62	0.09	0.31	0.25	0.12	0.37	0.28	0.28	0.46	0.64	0.39	0.77
**GT40**	0.00	0.00	0.00	0.00	0.00	0.00	0.00	0.00	0.00	0.00	0.00	0.00	0.00	0.00	0.00	0.00	0.00	0.00	0.00	0.00	0.00
**CE13**	0.00	0.00	0.05	0.00	0.03	0.00	0.00	0.00	0.00	0.00	0.00	0.00	0.00	0.00	0.00	0.00	0.00	0.00	0.00	0.00	0.00
**GT37**	0.00	0.00	0.00	0.00	0.00	0.00	0.00	0.00	0.00	0.00	0.00	0.00	0.00	0.00	0.00	0.00	0.00	0.00	0.00	0.00	0.00
**GH33**	0.23	0.25	0.05	0.30	0.20	0.29	0.17	0.21	0.23	0.13	0.02	0.30	0.10	0.18	0.19	0.26	0.31	0.14	0.30	0.24	0.09
**GT65**	0.00	0.00	0.00	0.00	0.00	0.00	0.00	0.00	0.00	0.00	0.00	0.00	0.00	0.00	0.00	0.00	0.00	0.00	0.00	0.00	0.00
**GH86**	0.00	0.00	0.00	0.00	0.00	0.02	0.00	0.00	0.00	0.02	0.00	0.00	0.00	0.01	0.01	0.00	0.00	0.02	0.00	0.00	0.00
**GH123**	0.13	0.14	0.07	0.25	0.06	0.11	0.13	0.13	0.18	0.03	0.00	0.09	0.00	0.10	0.09	0.19	0.09	0.07	0.23	0.17	0.05
**GH96**	0.00	0.00	0.00	0.00	0.00	0.00	0.00	0.00	0.00	0.00	0.00	0.00	0.00	0.00	0.00	0.00	0.00	0.00	0.00	0.00	0.00
**GH14**	0.00	0.00	0.00	0.00	0.00	0.00	0.00	0.00	0.00	0.00	0.00	0.00	0.00	0.00	0.00	0.00	0.00	0.00	0.00	0.00	0.00
**CE3**	0.00	0.00	0.00	0.00	0.00	0.00	0.00	0.00	0.00	0.00	0.00	0.00	0.00	0.00	0.00	0.00	0.00	0.00	0.00	0.00	0.02
**PL10**	0.05	0.02	0.16	0.04	0.11	0.09	0.13	0.09	0.00	0.13	0.00	0.08	0.04	0.03	0.25	0.17	0.04	0.16	0.21	0.18	0.12
**GT48**	0.00	0.00	0.00	0.00	0.00	0.00	0.00	0.00	0.00	0.00	0.00	0.00	0.00	0.00	0.00	0.00	0.00	0.00	0.00	0.00	0.00
**PL6**	0.00	0.00	0.00	0.00	0.00	0.00	0.00	0.00	0.00	0.03	0.00	0.02	0.00	0.00	0.00	0.02	0.00	0.00	0.00	0.00	0.00
**GT83**	0.10	0.18	0.05	0.07	0.06	0.16	0.15	0.15	0.09	0.05	0.04	0.06	0.15	0.12	0.06	0.13	0.13	0.25	0.07	0.09	0.02
**GH126**	0.00	0.00	0.00	0.00	0.00	0.00	0.00	0.00	0.00	0.00	0.00	0.00	0.00	0.00	0.00	0.00	0.00	0.00	0.00	0.00	0.00
**GH9**	0.13	0.07	0.40	0.16	0.48	0.22	0.00	0.04	0.05	0.28	0.00	0.14	0.04	0.04	0.25	0.15	0.09	0.18	0.09	0.20	0.30
**GH75**	0.00	0.00	0.00	0.00	0.00	0.00	0.00	0.00	0.00	0.00	0.00	0.00	0.00	0.00	0.00	0.00	0.00	0.00	0.00	0.00	0.00
**GT71**	0.00	0.00	0.00	0.00	0.00	0.00	0.00	0.00	0.00	0.00	0.00	0.00	0.00	0.00	0.00	0.00	0.00	0.00	0.00	0.00	0.00
**GH46**	0.00	0.00	0.00	0.00	0.00	0.00	0.00	0.00	0.00	0.00	0.00	0.00	0.00	0.00	0.00	0.00	0.00	0.00	0.00	0.00	0.00
**GT61**	0.00	0.00	0.00	0.00	0.00	0.00	0.00	0.00	0.00	0.00	0.00	0.00	0.00	0.00	0.00	0.00	0.00	0.00	0.00	0.00	0.00
**GT3**	0.05	0.14	0.09	0.14	0.06	0.11	0.07	0.08	0.14	0.11	0.00	0.13	0.02	0.18	0.17	0.26	0.09	0.19	0.14	0.24	0.14
**PL3**	0.00	0.00	0.00	0.00	0.00	0.00	0.00	0.00	0.00	0.00	0.00	0.00	0.00	0.00	0.00	0.00	0.00	0.00	0.00	0.00	0.00
**GH108**	0.05	0.05	0.02	0.00	0.00	0.04	0.00	0.02	0.00	0.02	0.02	0.05	0.00	0.03	0.05	0.02	0.09	0.00	0.04	0.04	0.05
**GT56**	0.00	0.02	0.00	0.00	0.00	0.04	0.04	0.02	0.02	0.02	0.04	0.00	0.00	0.02	0.00	0.00	0.00	0.02	0.00	0.00	0.00
**PL11**	0.10	0.20	0.14	0.12	0.13	0.07	0.07	0.19	0.00	0.07	0.02	0.19	0.04	0.10	0.18	0.15	0.15	0.09	0.32	0.26	0.07
**GH45**	0.00	0.00	0.00	0.00	0.00	0.00	0.00	0.00	0.00	0.00	0.00	0.00	0.00	0.00	0.00	0.02	0.00	0.00	0.00	0.00	0.00
**GT34**	0.00	0.00	0.00	0.00	0.00	0.00	0.00	0.02	0.00	0.00	0.00	0.00	0.00	0.00	0.00	0.00	0.00	0.00	0.00	0.00	0.00
**PL14**	0.00	0.00	0.00	0.00	0.00	0.00	0.00	0.00	0.00	0.00	0.00	0.00	0.00	0.00	0.00	0.00	0.00	0.00	0.00	0.00	0.00
**GH19**	0.00	0.00	0.00	0.00	0.00	0.02	0.00	0.00	0.05	0.00	0.00	0.00	0.00	0.00	0.01	0.02	0.00	0.00	0.00	0.00	0.00
**GH118**	0.00	0.00	0.00	0.00	0.00	0.00	0.00	0.00	0.00	0.00	0.00	0.00	0.00	0.00	0.00	0.00	0.00	0.00	0.00	0.00	0.00
**GH82**	0.00	0.00	0.00	0.00	0.00	0.00	0.00	0.00	0.00	0.00	0.00	0.00	0.00	0.00	0.00	0.00	0.00	0.00	0.00	0.00	0.00
**GH76**	0.18	0.29	0.00	0.18	0.01	0.07	0.39	0.36	0.02	0.02	0.00	0.22	0.00	0.10	0.28	0.11	0.31	0.14	0.30	0.39	0.05
**GT4**	1.48	2.15	1.42	1.66	1.64	1.68	1.35	1.30	1.45	1.17	0.98	1.78	0.90	1.31	1.69	2.28	1.60	1.74	1.95	2.16	1.68
**GT87**	0.00	0.00	0.00	0.00	0.00	0.02	0.00	0.00	0.00	0.00	0.00	0.00	0.00	0.00	0.00	0.00	0.00	0.00	0.00	0.00	0.02
**GH24**	0.18	0.18	0.30	0.21	0.20	0.26	0.20	0.21	0.30	0.08	0.11	0.17	0.10	0.14	0.11	0.20	0.35	0.26	0.19	0.26	0.24
**GH90**	0.00	0.00	0.00	0.00	0.00	0.00	0.00	0.00	0.00	0.00	0.00	0.00	0.00	0.00	0.00	0.00	0.00	0.00	0.00	0.00	0.00
**GH57**	0.05	0.11	0.16	0.14	0.13	0.11	0.13	0.11	0.07	0.08	0.02	0.16	0.02	0.11	0.17	0.19	0.09	0.12	0.14	0.17	0.07
**GH36**	0.59	0.50	0.37	0.88	0.61	0.53	0.54	0.66	0.48	0.53	0.27	0.64	0.58	0.61	0.69	0.43	0.50	0.63	0.81	0.70	0.40
**GT46**	0.00	0.00	0.00	0.00	0.00	0.00	0.00	0.00	0.00	0.00	0.00	0.00	0.00	0.00	0.00	0.00	0.00	0.00	0.00	0.00	0.00
**GH66**	0.05	0.05	0.00	0.00	0.01	0.00	0.04	0.04	0.00	0.02	0.02	0.06	0.08	0.01	0.05	0.04	0.00	0.04	0.04	0.07	0.00
**GH48**	0.00	0.00	0.00	0.00	0.00	0.00	0.00	0.00	0.00	0.00	0.00	0.00	0.00	0.01	0.00	0.00	0.00	0.00	0.00	0.00	0.02
**GT53**	0.00	0.00	0.00	0.00	0.00	0.00	0.00	0.00	0.00	0.00	0.00	0.00	0.00	0.00	0.00	0.00	0.00	0.00	0.00	0.00	0.00
**GH60**	0.00	0.00	0.00	0.00	0.00	0.00	0.00	0.00	0.00	0.00	0.00	0.00	0.00	0.00	0.00	0.00	0.00	0.00	0.00	0.00	0.00

CAZY_group	CN-AD-1	CN-AD-2	CN-AD-3	CN-AD-4	CN-AD-5	CN-AD-6	CN-AD-7	CN-AD-8	CN-AD-9	CN-AD-10	CN-AD-11	CN-AD-12	CN-AD-13	CN-AD-14	CN-AD-15	CN-AD-16	CN-AD-17	CN-AD-18	CN-AD-19	CN-AD-20	CN-AD-21
**GT31**	0.00	0.00	0.00	0.00	0.00	0.02	0.00	0.02	0.02	0.00	0.02	0.00	0.00	0.00	0.00	0.00	0.00	0.00	0.00	0.00	0.00
**GH105**	0.33	0.36	0.51	0.71	0.56	0.35	0.46	0.55	0.34	0.31	0.13	0.28	0.13	0.27	0.75	0.70	0.42	0.44	0.94	0.94	0.24
**GT23**	0.00	0.00	0.00	0.00	0.00	0.00	0.00	0.00	0.00	0.03	0.00	0.02	0.00	0.00	0.00	0.02	0.00	0.00	0.00	0.00	0.02
**GH11**	0.00	0.00	0.00	0.00	0.00	0.00	0.00	0.00	0.00	0.00	0.00	0.00	0.00	0.00	0.00	0.00	0.00	0.00	0.00	0.00	0.00
**GT38**	0.00	0.00	0.00	0.00	0.00	0.00	0.00	0.00	0.00	0.00	0.00	0.00	0.00	0.00	0.00	0.00	0.00	0.00	0.00	0.00	0.00
**GT43**	0.00	0.00	0.00	0.00	0.00	0.00	0.00	0.00	0.00	0.00	0.00	0.00	0.00	0.00	0.00	0.00	0.00	0.00	0.00	0.00	0.00
**GH99**	0.05	0.02	0.00	0.02	0.00	0.00	0.02	0.02	0.00	0.00	0.00	0.02	0.00	0.01	0.00	0.00	0.00	0.00	0.00	0.02	0.00
**GH38**	0.18	0.11	0.12	0.14	0.16	0.22	0.13	0.13	0.36	0.18	0.22	0.20	0.10	0.13	0.33	0.17	0.15	0.26	0.25	0.39	0.09
**GH109**	0.28	0.18	0.19	0.44	0.20	0.27	0.11	0.15	0.07	0.16	0.09	0.23	0.08	0.24	0.25	0.30	0.26	0.25	0.25	0.37	0.19
**GH2**	3.24	3.32	2.54	3.99	2.81	3.18	2.81	3.67	1.79	2.61	1.48	2.97	1.46	2.82	3.69	4.17	3.13	3.38	5.35	5.20	2.33
**GT28**	0.26	0.29	0.37	0.28	0.38	0.26	0.20	0.24	0.32	0.25	0.34	0.30	0.50	0.30	0.33	0.31	0.24	0.26	0.27	0.30	0.30
**GH42**	0.10	0.05	0.19	0.09	0.16	0.11	0.09	0.09	0.20	0.15	0.20	0.25	0.19	0.08	0.15	0.13	0.04	0.12	0.25	0.35	0.16
**GT58**	0.00	0.00	0.00	0.00	0.00	0.00	0.00	0.00	0.00	0.00	0.00	0.00	0.00	0.00	0.00	0.00	0.00	0.00	0.00	0.00	0.00
**GH61**	0.00	0.00	0.00	0.00	0.00	0.00	0.00	0.00	0.00	0.00	0.00	0.00	0.00	0.00	0.00	0.00	0.00	0.00	0.00	0.00	0.00
**GH80**	0.00	0.00	0.00	0.00	0.00	0.00	0.00	0.00	0.00	0.00	0.00	0.00	0.00	0.00	0.00	0.00	0.00	0.00	0.00	0.00	0.00
**GH98**	0.00	0.00	0.00	0.04	0.00	0.00	0.00	0.00	0.00	0.00	0.00	0.00	0.00	0.00	0.01	0.00	0.00	0.00	0.00	0.02	0.00
**GH6**	0.00	0.00	0.00	0.00	0.00	0.00	0.00	0.00	0.00	0.00	0.00	0.00	0.00	0.00	0.00	0.00	0.00	0.00	0.00	0.00	0.00
**GT1**	0.03	0.02	0.02	0.02	0.11	0.07	0.02	0.02	0.05	0.08	0.00	0.02	0.00	0.03	0.03	0.04	0.02	0.00	0.02	0.06	0.02
**GH28**	0.66	0.72	0.70	0.65	0.60	0.68	0.39	0.64	0.18	0.69	0.20	0.45	0.29	0.51	1.00	1.02	0.50	0.65	1.26	1.11	0.72
**GT42**	0.00	0.00	0.00	0.00	0.00	0.00	0.00	0.00	0.00	0.00	0.00	0.00	0.00	0.00	0.00	0.00	0.00	0.00	0.00	0.00	0.00
**GH128**	0.00	0.00	0.00	0.00	0.00	0.00	0.00	0.00	0.00	0.00	0.00	0.00	0.00	0.00	0.00	0.00	0.00	0.00	0.00	0.00	0.00
**CE12**	0.03	0.18	0.12	0.14	0.16	0.11	0.09	0.09	0.02	0.15	0.04	0.05	0.00	0.04	0.18	0.20	0.11	0.07	0.27	0.18	0.12
**CE16**	0.00	0.00	0.00	0.00	0.00	0.00	0.00	0.00	0.00	0.00	0.00	0.00	0.00	0.00	0.00	0.00	0.00	0.00	0.00	0.00	0.00
**GT89**	0.00	0.00	0.00	0.00	0.00	0.00	0.00	0.00	0.00	0.00	0.00	0.00	0.00	0.00	0.00	0.00	0.00	0.00	0.00	0.00	0.00
**GT11**	0.03	0.05	0.07	0.11	0.09	0.05	0.02	0.06	0.00	0.03	0.04	0.03	0.04	0.06	0.05	0.15	0.09	0.07	0.09	0.07	0.07
**GT77**	0.03	0.05	0.02	0.02	0.00	0.00	0.02	0.00	0.00	0.00	0.02	0.00	0.00	0.01	0.01	0.00	0.02	0.00	0.00	0.00	0.02
**GT55**	0.00	0.00	0.00	0.00	0.00	0.00	0.00	0.00	0.00	0.00	0.00	0.00	0.00	0.00	0.00	0.00	0.00	0.00	0.00	0.00	0.00
**GT18**	0.00	0.00	0.00	0.00	0.00	0.00	0.00	0.00	0.00	0.00	0.00	0.00	0.00	0.00	0.00	0.00	0.00	0.00	0.00	0.00	0.00
**GH87**	0.00	0.00	0.00	0.00	0.00	0.00	0.00	0.00	0.00	0.00	0.00	0.00	0.02	0.00	0.01	0.00	0.02	0.00	0.00	0.00	0.03
**PL5**	0.00	0.00	0.00	0.00	0.00	0.00	0.00	0.00	0.00	0.00	0.00	0.00	0.00	0.00	0.00	0.00	0.00	0.00	0.00	0.00	0.00
**GH22**	0.00	0.00	0.00	0.00	0.00	0.00	0.00	0.00	0.00	0.00	0.00	0.00	0.00	0.00	0.00	0.00	0.00	0.00	0.00	0.00	0.00
**CE2**	0.00	0.00	0.02	0.04	0.10	0.04	0.00	0.04	0.02	0.07	0.00	0.06	0.02	0.02	0.10	0.04	0.00	0.09	0.05	0.09	0.07
**GH120**	0.00	0.00	0.00	0.00	0.09	0.00	0.00	0.00	0.05	0.15	0.02	0.05	0.13	0.01	0.00	0.04	0.00	0.00	0.09	0.09	0.02
**GH73**	0.36	0.25	0.37	0.42	0.44	0.53	0.26	0.41	0.39	0.23	0.49	0.30	0.42	0.35	0.41	0.46	0.44	0.40	0.35	0.37	0.31
**GT17**	0.00	0.00	0.00	0.00	0.00	0.00	0.00	0.00	0.00	0.02	0.00	0.00	0.00	0.00	0.00	0.00	0.00	0.00	0.00	0.00	0.00
**GH124**	0.00	0.00	0.00	0.00	0.00	0.00	0.00	0.00	0.00	0.00	0.00	0.00	0.00	0.00	0.00	0.00	0.00	0.00	0.00	0.00	0.00
**GH54**	0.00	0.00	0.00	0.00	0.00	0.00	0.00	0.00	0.00	0.00	0.00	0.00	0.00	0.00	0.00	0.00	0.00	0.00	0.00	0.00	0.00
**GH100**	0.00	0.00	0.00	0.00	0.00	0.00	0.00	0.00	0.00	0.00	0.00	0.00	0.00	0.00	0.00	0.00	0.00	0.00	0.00	0.00	0.00
**GH113**	0.03	0.00	0.00	0.02	0.04	0.07	0.00	0.02	0.00	0.05	0.02	0.02	0.02	0.00	0.04	0.00	0.00	0.04	0.04	0.02	0.02
**GT5**	0.13	0.27	0.42	0.25	0.43	0.46	0.13	0.08	0.20	0.23	0.25	0.31	0.27	0.24	0.29	0.28	0.11	0.33	0.35	0.30	0.42
**GH94**	0.10	0.16	0.65	0.30	0.43	0.38	0.11	0.00	0.00	0.48	0.07	0.08	0.25	0.23	0.29	0.07	0.02	0.12	0.18	0.30	0.58
**GT66**	0.00	0.00	0.00	0.00	0.00	0.00	0.00	0.00	0.00	0.00	0.00	0.00	0.00	0.00	0.00	0.00	0.00	0.00	0.00	0.00	0.00
**GT94**	0.00	0.00	0.00	0.00	0.00	0.00	0.00	0.00	0.00	0.00	0.00	0.00	0.00	0.00	0.00	0.00	0.00	0.00	0.00	0.00	0.00
**GH58**	0.03	0.00	0.00	0.00	0.00	0.00	0.00	0.00	0.00	0.00	0.00	0.00	0.00	0.00	0.00	0.00	0.00	0.00	0.00	0.00	0.00
**GH1**	0.20	0.27	0.30	0.12	0.77	0.27	0.13	0.88	0.57	0.41	1.54	0.88	0.46	0.18	0.22	0.37	0.09	0.25	0.12	0.11	0.65
**PL22**	0.00	0.00	0.00	0.00	0.00	0.00	0.00	0.00	0.00	0.03	0.00	0.00	0.00	0.00	0.00	0.00	0.02	0.00	0.00	0.00	0.00
**GT9**	0.15	0.18	0.21	0.23	0.06	0.20	0.13	0.11	0.05	0.16	0.07	0.09	0.12	0.13	0.09	0.26	0.07	0.19	0.14	0.09	0.02
**GT76**	0.00	0.00	0.00	0.00	0.00	0.00	0.00	0.00	0.00	0.00	0.00	0.00	0.00	0.00	0.00	0.00	0.00	0.00	0.00	0.00	0.00
**GT32**	0.03	0.09	0.00	0.16	0.06	0.18	0.07	0.00	0.18	0.13	0.02	0.05	0.02	0.07	0.04	0.19	0.07	0.09	0.05	0.09	0.05
**GT47**	0.00	0.00	0.05	0.04	0.09	0.04	0.02	0.02	0.00	0.05	0.07	0.06	0.04	0.02	0.04	0.07	0.00	0.07	0.02	0.00	0.02
**GH84**	0.15	0.07	0.26	0.23	0.19	0.11	0.09	0.11	0.11	0.05	0.02	0.13	0.06	0.11	0.14	0.22	0.13	0.11	0.07	0.15	0.05
**GH83**	0.00	0.00	0.00	0.00	0.00	0.00	0.00	0.00	0.00	0.00	0.00	0.00	0.00	0.00	0.00	0.00	0.00	0.00	0.00	0.00	0.00
**GT59**	0.00	0.00	0.00	0.00	0.00	0.00	0.00	0.00	0.00	0.00	0.00	0.00	0.00	0.00	0.00	0.00	0.00	0.00	0.00	0.00	0.00

CAZY_group	CN-AD-1	CN-AD-2	CN-AD-3	CN-AD-4	CN-AD-5	CN-AD-6	CN-AD-7	CN-AD-8	CN-AD-9	CN-AD-10	CN-AD-11	CN-AD-12	CN-AD-13	CN-AD-14	CN-AD-15	CN-AD-16	CN-AD-17	CN-AD-18	CN-AD-19	CN-AD-20	CN-AD-21
GH34	0.00	0.00	0.00	0.00	0.00	0.00	0.00	0.00	0.00	0.00	0.00	0.00	0.00	0.00	0.00	0.00	0.00	0.00	0.00	0.00	0.00
GT14	0.03	0.02	0.00	0.00	0.01	0.02	0.02	0.02	0.00	0.02	0.07	0.00	0.00	0.00	0.05	0.04	0.02	0.05	0.07	0.06	0.02
GH47	0.00	0.00	0.00	0.00	0.00	0.00	0.00	0.00	0.00	0.00	0.02	0.00	0.00	0.01	0.01	0.00	0.00	0.00	0.00	0.00	0.00
GH97	0.69	0.72	0.81	0.90	0.58	0.64	0.67	0.58	0.52	0.43	0.02	0.70	0.12	0.70	0.99	1.24	0.68	0.92	1.66	1.48	0.44
GH50	0.00	0.09	0.00	0.05	0.00	0.00	0.07	0.15	0.00	0.00	0.00	0.05	0.00	0.02	0.04	0.00	0.09	0.04	0.07	0.06	0.05
GT26	0.23	0.18	0.26	0.21	0.19	0.16	0.11	0.15	0.07	0.18	0.16	0.14	0.10	0.08	0.19	0.17	0.11	0.18	0.21	0.17	0.17
GH18	0.43	0.54	0.37	0.48	0.48	0.53	0.57	0.64	0.39	0.28	0.25	0.56	0.31	0.28	0.56	0.33	0.42	0.53	0.53	0.66	0.42
GH37	0.00	0.02	0.00	0.00	0.00	0.07	0.02	0.04	0.05	0.03	0.09	0.00	0.00	0.02	0.00	0.00	0.00	0.04	0.00	0.00	0.00
PL13	0.03	0.09	0.00	0.02	0.00	0.02	0.02	0.06	0.00	0.00	0.00	0.00	0.00	0.02	0.08	0.02	0.02	0.02	0.05	0.06	0.03
GH27	0.15	0.18	0.14	0.19	0.13	0.13	0.09	0.21	0.11	0.16	0.00	0.17	0.10	0.16	0.22	0.22	0.11	0.19	0.25	0.33	0.12
GT54	0.00	0.00	0.00	0.00	0.00	0.00	0.00	0.00	0.00	0.00	0.00	0.00	0.00	0.00	0.00	0.00	0.00	0.00	0.00	0.00	0.00
GT91	0.00	0.00	0.00	0.00	0.00	0.00	0.00	0.00	0.00	0.00	0.00	0.00	0.00	0.00	0.00	0.00	0.00	0.00	0.00	0.00	0.00
GT72	0.00	0.00	0.00	0.00	0.00	0.00	0.00	0.00	0.00	0.00	0.00	0.00	0.00	0.00	0.00	0.00	0.00	0.00	0.00	0.00	0.00
GH67	0.03	0.02	0.09	0.09	0.09	0.04	0.04	0.06	0.05	0.08	0.00	0.09	0.02	0.05	0.08	0.20	0.02	0.00	0.19	0.04	0.09
GH12	0.00	0.00	0.00	0.00	0.00	0.00	0.00	0.00	0.00	0.00	0.00	0.00	0.00	0.00	0.00	0.00	0.00	0.00	0.00	0.00	0.00
GH91	0.03	0.05	0.00	0.07	0.03	0.00	0.07	0.06	0.00	0.02	0.04	0.03	0.02	0.01	0.03	0.00	0.09	0.05	0.00	0.06	0.02
GT69	0.00	0.00	0.00	0.00	0.00	0.00	0.00	0.00	0.00	0.00	0.00	0.00	0.00	0.00	0.00	0.00	0.00	0.00	0.00	0.00	0.00
GH106	0.15	0.20	0.14	0.14	0.11	0.22	0.13	0.26	0.11	0.28	0.02	0.17	0.04	0.36	0.20	0.31	0.22	0.30	0.34	0.30	0.12
GT62	0.00	0.00	0.00	0.00	0.00	0.00	0.00	0.00	0.00	0.00	0.00	0.00	0.00	0.00	0.00	0.00	0.00	0.00	0.00	0.00	0.00
GH17	0.00	0.00	0.00	0.00	0.00	0.00	0.00	0.00	0.00	0.00	0.00	0.00	0.00	0.00	0.00	0.00	0.00	0.00	0.00	0.00	0.00
GH88	0.23	0.34	0.16	0.48	0.19	0.15	0.24	0.30	0.16	0.23	0.07	0.28	0.13	0.20	0.55	0.41	0.42	0.26	0.53	0.57	0.24
CE5	0.00	0.00	0.00	0.00	0.00	0.00	0.00	0.00	0.00	0.00	0.00	0.00	0.00	0.00	0.00	0.00	0.00	0.00	0.00	0.00	0.00
GT8	0.08	0.05	0.09	0.09	0.13	0.09	0.11	0.06	0.05	0.03	0.11	0.00	0.25	0.07	0.04	0.09	0.07	0.05	0.04	0.09	0.09
GT50	0.00	0.00	0.00	0.00	0.00	0.00	0.00	0.00	0.00	0.00	0.00	0.00	0.00	0.00	0.00	0.00	0.00	0.00	0.00	0.00	0.00
GT20	0.10	0.07	0.05	0.12	0.09	0.16	0.04	0.09	0.05	0.08	0.04	0.09	0.02	0.07	0.05	0.09	0.11	0.09	0.12	0.06	0.05
PL9	0.03	0.16	0.00	0.07	0.11	0.02	0.07	0.08	0.02	0.00	0.20	0.08	0.02	0.02	0.08	0.02	0.04	0.00	0.07	0.09	0.12
GH55	0.03	0.00	0.02	0.05	0.06	0.02	0.00	0.00	0.00	0.02	0.00	0.03	0.00	0.02	0.06	0.04	0.02	0.04	0.04	0.06	0.02
GH64	0.00	0.00	0.00	0.00	0.00	0.00	0.00	0.00	0.00	0.00	0.00	0.00	0.00	0.00	0.00	0.00	0.00	0.00	0.02	0.00	0.00
GH127	0.20	0.18	0.40	0.18	0.27	0.33	0.17	0.21	0.23	0.31	0.11	0.22	0.12	0.25	0.19	0.30	0.22	0.25	0.43	0.30	0.21
GH103	0.00	0.02	0.00	0.00	0.00	0.04	0.02	0.02	0.00	0.00	0.04	0.00	0.00	0.01	0.00	0.00	0.00	0.02	0.00	0.00	0.00
GH3	2.37	2.28	1.98	2.53	1.50	2.16	1.39	2.09	1.23	1.61	1.03	2.08	1.10	1.69	2.84	2.33	2.30	2.55	3.38	3.13	1.89
GT45	0.00	0.00	0.00	0.00	0.00	0.02	0.00	0.00	0.00	0.00	0.00	0.00	0.00	0.00	0.00	0.00	0.00	0.00	0.00	0.00	0.00
GT74	0.00	0.00	0.00	0.00	0.00	0.00	0.00	0.00	0.00	0.00	0.00	0.00	0.00	0.00	0.00	0.00	0.00	0.00	0.00	0.00	0.00
GT90	0.00	0.00	0.00	0.00	0.00	0.00	0.00	0.00	0.00	0.00	0.00	0.00	0.00	0.00	0.00	0.00	0.00	0.00	0.00	0.00	0.00
GH116	0.08	0.05	0.05	0.05	0.06	0.15	0.02	0.02	0.00	0.05	0.00	0.02	0.00	0.04	0.08	0.09	0.09	0.18	0.04	0.13	0.03
GH31	0.94	0.68	0.81	1.08	0.88	0.73	0.63	0.96	0.66	0.67	0.45	0.83	0.54	0.58	1.03	0.89	0.77	0.79	1.43	1.35	0.70
GT12	0.00	0.00	0.00	0.00	0.00	0.00	0.00	0.00	0.00	0.00	0.00	0.00	0.00	0.00	0.00	0.00	0.00	0.00	0.00	0.00	0.00
CE1	0.31	0.41	0.23	0.37	0.33	0.40	0.37	0.21	0.20	0.18	0.13	0.20	0.27	0.15	0.42	0.39	0.35	0.35	0.51	0.42	0.33
GH121	0.00	0.00	0.00	0.00	0.00	0.00	0.00	0.00	0.02	0.00	0.00	0.11	0.00	0.01	0.00	0.00	0.00	0.04	0.00	0.00	0.00
GH79	0.00	0.00	0.00	0.00	0.00	0.00	0.00	0.00	0.00	0.00	0.00	0.00	0.04	0.02	0.00	0.00	0.00	0.00	0.00	0.00	0.03
GT73	0.00	0.00	0.00	0.00	0.00	0.00	0.00	0.00	0.00	0.02	0.00	0.00	0.00	0.00	0.00	0.00	0.00	0.00	0.00	0.00	0.00
GH77	0.20	0.54	0.56	0.48	0.66	0.44	0.24	0.24	0.36	0.39	0.27	0.59	0.40	0.34	0.60	0.39	0.26	0.42	0.50	0.42	0.63
PL17	0.00	0.02	0.02	0.00	0.00	0.00	0.00	0.00	0.00	0.03	0.00	0.00	0.00	0.01	0.01	0.02	0.00	0.00	0.02	0.00	0.03
PL8	0.20	0.14	0.16	0.60	0.16	0.07	0.15	0.32	0.07	0.03	0.04	0.36	0.00	0.17	0.39	0.41	0.42	0.37	0.25	0.55	0.19
CE9	0.26	0.32	0.23	0.48	0.24	0.22	0.35	0.36	0.39	0.16	0.29	0.34	0.29	0.25	0.36	0.35	0.33	0.37	0.25	0.26	0.37
CE15	0.00	0.02	0.02	0.00	0.00	0.05	0.00	0.02	0.00	0.02	0.00	0.00	0.00	0.02	0.01	0.00	0.00	0.04	0.04	0.00	0.02
GT79	0.00	0.00	0.00	0.00	0.00	0.00	0.00	0.00	0.00	0.00	0.00	0.00	0.00	0.00	0.00	0.00	0.00	0.00	0.00	0.00	0.00
GT10	0.03	0.00	0.00	0.00	0.00	0.00	0.00	0.02	0.00	0.00	0.00	0.00	0.00	0.01	0.00	0.02	0.02	0.00	0.00	0.00	0.00
GH119	0.00	0.00	0.00	0.00	0.00	0.00	0.00	0.00	0.00	0.00	0.00	0.00	0.00	0.00	0.00	0.00	0.00	0.00	0.00	0.00	0.00
GH16	0.18	0.18	0.16	0.42	0.19	0.16	0.13	0.26	0.16	0.15	0.00	0.19	0.06	0.17	0.23	0.33	0.24	0.12	0.23	0.35	0.05
GH43	1.40	1.20	1.79	1.98	1.45	1.64	1.44	1.60	1.27	1.54	0.31	2.09	0.62	1.12	2.16	2.69	1.80	2.04	3.45	2.86	1.66
GH51	0.43	0.16	0.40	0.42	0.47	0.46	0.39	0.40	0.39	0.44	0.09	0.50	0.29	0.42	0.41	0.63	0.42	0.51	0.69	0.77	0.40
GT35	0.59	0.68	0.74	0.62	0.94	0.75	0.54	0.38	0.48	0.77	0.67	0.81	0.65	0.51	0.62	0.43	0.39	0.55	0.67	0.70	1.01

CAZY_group	CN-AD-1	CN-AD-2	CN-AD-3	CN-AD-4	CN-AD-5	CN-AD-6	CN-AD-7	CN-AD-8	CN-AD-9	CN-AD-10	CN-AD-11	CN-AD-12	CN-AD-13	CN-AD-14	CN-AD-15	CN-AD-16	CN-AD-17	CN-AD-18	CN-AD-19	CN-AD-20	CN-AD-21
**GT25**	0.00	0.00	0.00	0.00	0.00	0.02	0.00	0.00	0.00	0.00	0.04	0.00	0.00	0.00	0.00	0.00	0.02	0.00	0.00	0.00	0.00
**GT2**	2.89	3.89	2.93	3.74	3.48	3.82	2.94	2.99	3.18	3.04	2.46	3.39	2.25	2.85	3.70	4.39	2.96	3.87	4.18	3.87	3.64
**GT51**	0.64	0.79	0.79	0.85	0.80	0.93	0.89	0.85	0.93	0.51	0.92	0.77	0.58	0.68	0.79	0.91	0.88	0.86	0.81	0.85	0.66
**GH107**	0.00	0.00	0.00	0.00	0.00	0.00	0.00	0.00	0.00	0.00	0.00	0.00	0.00	0.00	0.00	0.00	0.00	0.00	0.00	0.00	0.00
**GH7**	0.00	0.00	0.00	0.00	0.00	0.00	0.00	0.00	0.00	0.00	0.00	0.00	0.00	0.00	0.00	0.00	0.00	0.00	0.00	0.00	0.00
**GT7**	0.00	0.00	0.00	0.00	0.00	0.00	0.00	0.00	0.00	0.00	0.00	0.00	0.00	0.00	0.00	0.00	0.00	0.00	0.00	0.00	0.00
**GT36**	0.00	0.00	0.00	0.00	0.00	0.00	0.00	0.00	0.00	0.00	0.00	0.00	0.00	0.00	0.00	0.00	0.00	0.00	0.00	0.00	0.00
**PL7**	0.00	0.00	0.00	0.00	0.00	0.00	0.00	0.00	0.00	0.00	0.00	0.00	0.00	0.00	0.00	0.00	0.00	0.00	0.00	0.00	0.00
**GH110**	0.10	0.07	0.07	0.23	0.13	0.09	0.11	0.17	0.07	0.05	0.02	0.13	0.08	0.10	0.09	0.28	0.15	0.07	0.12	0.18	0.07
**GT21**	0.00	0.00	0.00	0.00	0.00	0.00	0.00	0.00	0.00	0.00	0.00	0.00	0.00	0.00	0.00	0.00	0.00	0.00	0.00	0.00	0.00
**GH62**	0.00	0.00	0.00	0.00	0.00	0.00	0.00	0.00	0.00	0.00	0.00	0.00	0.00	0.00	0.00	0.00	0.00	0.00	0.00	0.00	0.00
**GH125**	0.20	0.20	0.12	0.18	0.07	0.18	0.15	0.17	0.05	0.07	0.07	0.22	0.06	0.07	0.18	0.24	0.18	0.18	0.34	0.24	0.09
**GH117**	0.13	0.11	0.05	0.11	0.04	0.05	0.09	0.06	0.05	0.05	0.00	0.06	0.02	0.05	0.10	0.15	0.15	0.12	0.04	0.11	0.03
**GH68**	0.00	0.00	0.00	0.00	0.00	0.00	0.00	0.00	0.00	0.00	0.00	0.00	0.04	0.00	0.00	0.00	0.00	0.00	0.00	0.00	0.00
**GH85**	0.00	0.00	0.05	0.00	0.00	0.00	0.02	0.02	0.00	0.02	0.02	0.03	0.00	0.01	0.01	0.02	0.00	0.00	0.04	0.02	0.02
**GH92**	1.56	1.60	1.23	1.68	0.71	1.28	1.39	1.60	1.02	0.71	0.07	1.38	0.17	1.13	1.67	1.93	1.69	1.44	1.84	2.29	0.59
**GH40**	0.00	0.00	0.00	0.00	0.00	0.00	0.00	0.00	0.00	0.00	0.00	0.00	0.00	0.00	0.00	0.00	0.00	0.00	0.00	0.00	0.00
**GT49**	0.00	0.00	0.00	0.00	0.00	0.00	0.00	0.00	0.00	0.00	0.00	0.00	0.00	0.00	0.00	0.00	0.00	0.00	0.00	0.00	0.00
**GH112**	0.08	0.14	0.14	0.12	0.16	0.13	0.04	0.09	0.20	0.11	0.29	0.09	0.10	0.12	0.15	0.04	0.00	0.14	0.11	0.17	0.28
**GT67**	0.00	0.00	0.00	0.00	0.00	0.00	0.00	0.00	0.00	0.00	0.00	0.00	0.00	0.00	0.00	0.00	0.00	0.00	0.00	0.00	0.00
**CE8**	0.05	0.14	0.14	0.25	0.19	0.15	0.33	0.17	0.07	0.13	0.20	0.17	0.10	0.12	0.24	0.20	0.13	0.23	0.35	0.18	0.14
**GT86**	0.00	0.00	0.00	0.00	0.00	0.00	0.00	0.00	0.00	0.00	0.00	0.00	0.00	0.00	0.00	0.00	0.00	0.00	0.00	0.00	0.00
**GH101**	0.00	0.00	0.00	0.00	0.07	0.02	0.00	0.04	0.05	0.00	0.02	0.05	0.00	0.01	0.00	0.02	0.00	0.05	0.00	0.00	0.00
**GH81**	0.00	0.02	0.00	0.00	0.00	0.00	0.00	0.00	0.00	0.00	0.00	0.00	0.00	0.01	0.00	0.00	0.00	0.00	0.02	0.00	0.05
**GT93**	0.00	0.00	0.00	0.00	0.00	0.00	0.00	0.00	0.00	0.00	0.00	0.00	0.00	0.00	0.00	0.00	0.00	0.00	0.00	0.00	0.00
**GT63**	0.00	0.00	0.00	0.00	0.00	0.00	0.00	0.00	0.00	0.00	0.00	0.00	0.00	0.00	0.00	0.00	0.00	0.00	0.00	0.00	0.00
**CE11**	0.23	0.25	0.19	0.26	0.13	0.27	0.17	0.15	0.14	0.15	0.07	0.19	0.12	0.15	0.20	0.33	0.15	0.26	0.32	0.22	0.10
**GT19**	0.15	0.09	0.16	0.19	0.09	0.18	0.07	0.15	0.07	0.16	0.09	0.11	0.02	0.12	0.13	0.33	0.11	0.28	0.21	0.18	0.07
**GT33**	0.00	0.00	0.00	0.00	0.00	0.00	0.00	0.00	0.00	0.00	0.00	0.00	0.00	0.00	0.00	0.00	0.00	0.00	0.00	0.00	0.00
**GH21**	0.00	0.00	0.00	0.00	0.00	0.00	0.00	0.00	0.00	0.00	0.00	0.00	0.00	0.00	0.00	0.00	0.00	0.00	0.00	0.00	0.00
**GT68**	0.00	0.00	0.00	0.00	0.00	0.00	0.00	0.00	0.00	0.00	0.00	0.00	0.00	0.00	0.00	0.00	0.00	0.00	0.00	0.00	0.00
**GH89**	0.26	0.25	0.14	0.39	0.10	0.11	0.26	0.38	0.05	0.11	0.04	0.27	0.08	0.25	0.18	0.30	0.31	0.16	0.34	0.42	0.14
**GT82**	0.00	0.00	0.00	0.00	0.00	0.00	0.00	0.00	0.00	0.00	0.00	0.00	0.00	0.00	0.00	0.00	0.00	0.00	0.00	0.00	0.00
**GH30**	0.28	0.11	0.14	0.26	0.07	0.18	0.15	0.23	0.18	0.05	0.04	0.28	0.06	0.15	0.31	0.19	0.26	0.21	0.37	0.30	0.09
**GH20**	1.00	1.24	0.74	1.78	0.74	1.17	1.22	1.32	0.68	0.46	0.22	1.00	0.23	0.78	1.03	1.52	1.05	0.83	1.05	1.38	0.38
**GH52**	0.00	0.00	0.00	0.00	0.00	0.00	0.00	0.00	0.00	0.00	0.00	0.00	0.00	0.00	0.00	0.00	0.00	0.00	0.00	0.00	0.00
**GT84**	0.00	0.00	0.00	0.00	0.06	0.00	0.00	0.00	0.05	0.00	0.00	0.00	0.00	0.00	0.00	0.00	0.00	0.00	0.00	0.00	0.00
**GT15**	0.00	0.00	0.00	0.00	0.00	0.00	0.00	0.00	0.00	0.00	0.00	0.00	0.00	0.00	0.00	0.00	0.00	0.00	0.00	0.00	0.00
**GH93**	0.03	0.00	0.00	0.00	0.00	0.00	0.02	0.00	0.00	0.00	0.00	0.00	0.00	0.01	0.00	0.00	0.02	0.00	0.00	0.00	0.00
**PL16**	0.00	0.00	0.00	0.00	0.00	0.00	0.00	0.00	0.00	0.00	0.00	0.00	0.00	0.00	0.00	0.00	0.00	0.00	0.00	0.00	0.00
**GH26**	0.03	0.02	0.09	0.12	0.23	0.13	0.02	0.06	0.02	0.11	0.00	0.03	0.02	0.08	0.13	0.19	0.09	0.12	0.28	0.18	0.17
**GT75**	0.00	0.00	0.00	0.00	0.00	0.00	0.00	0.00	0.00	0.00	0.00	0.00	0.00	0.00	0.00	0.00	0.00	0.00	0.00	0.00	0.00
**GH53**	0.00	0.02	0.14	0.14	0.10	0.26	0.02	0.02	0.00	0.13	0.02	0.08	0.08	0.04	0.15	0.07	0.04	0.14	0.23	0.13	0.09
**GT88**	0.00	0.00	0.00	0.00	0.00	0.00	0.00	0.00	0.00	0.00	0.00	0.00	0.00	0.00	0.00	0.00	0.00	0.00	0.00	0.00	0.00
**GT24**	0.00	0.00	0.00	0.00	0.00	0.00	0.00	0.00	0.00	0.00	0.00	0.00	0.00	0.00	0.00	0.00	0.00	0.00	0.00	0.00	0.00
**GT81**	0.00	0.00	0.00	0.00	0.00	0.00	0.00	0.00	0.00	0.00	0.00	0.00	0.00	0.00	0.00	0.00	0.00	0.00	0.00	0.00	0.00
**GH4**	0.15	0.02	0.02	0.02	0.23	0.07	0.17	0.28	0.27	0.20	0.36	0.13	0.19	0.08	0.04	0.09	0.20	0.18	0.04	0.06	0.07
**GT78**	0.00	0.00	0.00	0.00	0.00	0.00	0.00	0.00	0.00	0.00	0.00	0.00	0.00	0.00	0.00	0.00	0.00	0.00	0.00	0.00	0.00
**GH8**	0.00	0.00	0.00	0.00	0.06	0.07	0.00	0.02	0.00	0.08	0.04	0.00	0.02	0.02	0.08	0.06	0.00	0.04	0.04	0.04	0.09
**GH70**	0.00	0.00	0.00	0.00	0.00	0.00	0.00	0.00	0.00	0.00	0.00	0.00	0.52	0.00	0.00	0.04	0.00	0.00	0.00	0.02	0.00
**GH122**	0.00	0.00	0.00	0.00	0.00	0.00	0.00	0.00	0.00	0.00	0.00	0.00	0.00	0.00	0.00	0.00	0.00	0.00	0.00	0.00	0.00
**GH74**	0.00	0.00	0.02	0.00	0.07	0.00	0.00	0.00	0.00	0.07	0.00	0.00	0.00	0.02	0.00	0.02	0.00	0.02	0.04	0.02	0.07
**GT41**	0.03	0.00	0.02	0.00	0.00	0.02	0.00	0.00	0.00	0.02	0.00	0.00	0.00	0.00	0.00	0.00	0.00	0.00	0.00	0.00	0.00

CAZY_group	CN-AD-1	CN-AD-2	CN-AD-3	CN-AD-4	CN-AD-5	CN-AD-6	CN-AD-7	CN-AD-8	CN-AD-9	CN-AD-10	CN-AD-11	CN-AD-12	CN-AD-13	CN-AD-14	CN-AD-15	CN-AD-16	CN-AD-17	CN-AD-18	CN-AD-19	CN-AD-20	CN-AD-21
**PL1**	0.13	0.25	0.16	0.28	0.23	0.13	0.33	0.36	0.00	0.15	0.09	0.20	0.02	0.09	0.33	0.15	0.26	0.25	0.43	0.30	0.17
**GH15**	0.03	0.02	0.02	0.09	0.01	0.07	0.04	0.00	0.02	0.02	0.00	0.05	0.00	0.03	0.00	0.04	0.02	0.02	0.09	0.09	0.02
**GH32**	0.41	0.29	0.49	0.39	0.43	0.22	0.30	0.43	0.27	0.34	0.31	0.45	0.63	0.13	0.34	0.33	0.35	0.30	0.50	0.41	0.31
**GH44**	0.00	0.00	0.00	0.00	0.01	0.00	0.00	0.00	0.00	0.03	0.00	0.00	0.00	0.00	0.00	0.00	0.00	0.00	0.00	0.00	0.00
**GT6**	0.00	0.00	0.00	0.00	0.00	0.00	0.00	0.00	0.00	0.00	0.02	0.00	0.00	0.00	0.00	0.00	0.00	0.00	0.00	0.00	0.00
**GH13**	1.51	2.58	2.72	1.94	2.88	2.32	1.26	1.24	2.13	1.89	2.80	2.89	2.36	1.35	2.70	2.07	1.75	2.15	2.02	2.84	2.92
**GT80**	0.00	0.00	0.00	0.00	0.00	0.00	0.00	0.02	0.02	0.02	0.02	0.05	0.00	0.03	0.00	0.00	0.04	0.02	0.00	0.00	0.02
**GH71**	0.00	0.00	0.00	0.00	0.00	0.00	0.00	0.00	0.00	0.00	0.00	0.00	0.00	0.00	0.00	0.00	0.00	0.00	0.00	0.00	0.00
**GH63**	0.08	0.07	0.02	0.09	0.03	0.22	0.02	0.06	0.05	0.11	0.16	0.09	0.00	0.05	0.08	0.22	0.02	0.16	0.21	0.04	0.07
**PL21**	0.00	0.00	0.00	0.04	0.00	0.00	0.00	0.00	0.00	0.00	0.00	0.03	0.00	0.04	0.03	0.07	0.04	0.02	0.02	0.07	0.10
**GH65**	0.08	0.07	0.09	0.19	0.16	0.13	0.00	0.09	0.23	0.02	0.18	0.11	0.04	0.09	0.22	0.20	0.11	0.23	0.14	0.13	0.16
**CE4**	0.26	0.29	0.44	0.42	0.47	0.53	0.30	0.32	0.48	0.46	0.40	0.39	0.58	0.34	0.51	0.41	0.22	0.46	0.62	0.46	0.61
**CE14**	0.00	0.02	0.00	0.02	0.00	0.07	0.04	0.02	0.00	0.03	0.02	0.02	0.00	0.00	0.04	0.00	0.02	0.02	0.02	0.02	0.00
**GH115**	0.15	0.25	0.19	0.16	0.11	0.11	0.22	0.41	0.14	0.07	0.00	0.16	0.06	0.14	0.24	0.28	0.15	0.16	0.53	0.42	0.24
**GH104**	0.03	0.00	0.00	0.00	0.00	0.00	0.00	0.02	0.00	0.00	0.02	0.00	0.00	0.00	0.00	0.00	0.00	0.02	0.00	0.00	0.00
**GH111**	0.00	0.00	0.00	0.00	0.00	0.00	0.00	0.00	0.00	0.00	0.00	0.00	0.00	0.00	0.00	0.00	0.00	0.00	0.00	0.00	0.00
**GH95**	0.28	0.54	0.33	0.69	0.51	0.33	0.48	0.75	0.20	0.44	0.11	0.38	0.15	0.50	0.64	0.74	0.66	0.46	0.94	0.74	0.49
**GT70**	0.00	0.00	0.00	0.00	0.00	0.00	0.00	0.00	0.00	0.00	0.00	0.00	0.00	0.00	0.00	0.00	0.00	0.00	0.00	0.00	0.00
**CE10**	0.28	0.16	0.02	0.23	0.06	0.22	0.20	0.26	0.14	0.11	0.09	0.20	0.08	0.10	0.14	0.22	0.22	0.18	0.12	0.26	0.07
**GH10**	0.05	0.05	0.05	0.04	0.09	0.07	0.13	0.04	0.00	0.03	0.00	0.05	0.02	0.06	0.15	0.19	0.04	0.11	0.18	0.15	0.19
**GH130**	0.26	0.32	0.23	0.41	0.29	0.31	0.37	0.30	0.11	0.18	0.02	0.34	0.04	0.19	0.32	0.19	0.33	0.30	0.41	0.39	0.23
**PL20**	0.00	0.00	0.00	0.00	0.00	0.00	0.00	0.00	0.00	0.00	0.00	0.00	0.00	0.00	0.00	0.00	0.00	0.00	0.00	0.00	0.00
**GH69**	0.00	0.00	0.00	0.00	0.00	0.00	0.00	0.00	0.00	0.00	0.00	0.00	0.00	0.00	0.00	0.00	0.00	0.00	0.00	0.00	0.00
**PL2**	0.00	0.00	0.00	0.00	0.00	0.00	0.00	0.00	0.00	0.00	0.00	0.00	0.00	0.00	0.00	0.00	0.00	0.00	0.00	0.00	0.00
**GH49**	0.00	0.00	0.00	0.00	0.00	0.00	0.00	0.00	0.00	0.00	0.00	0.00	0.00	0.00	0.00	0.00	0.00	0.00	0.00	0.00	0.00
**GH25**	0.13	0.29	0.47	0.32	0.51	0.29	0.28	0.28	0.39	0.30	0.20	0.28	0.29	0.20	0.36	0.30	0.28	0.33	0.44	0.15	0.35
**GH59**	0.00	0.00	0.00	0.02	0.04	0.00	0.00	0.00	0.00	0.03	0.00	0.00	0.00	0.00	0.00	0.00	0.00	0.00	0.02	0.00	0.02
**GH35**	0.38	0.34	0.09	0.58	0.37	0.26	0.39	0.51	0.30	0.21	0.07	0.20	0.04	0.38	0.33	0.44	0.46	0.33	0.55	0.48	0.12
**GT39**	0.00	0.00	0.00	0.00	0.00	0.00	0.00	0.00	0.00	0.00	0.00	0.00	0.00	0.00	0.00	0.00	0.00	0.00	0.00	0.00	0.00
**GT57**	0.00	0.00	0.00	0.00	0.00	0.00	0.00	0.00	0.00	0.00	0.00	0.00	0.00	0.00	0.00	0.00	0.00	0.00	0.00	0.00	0.00
**GH129**	0.00	0.00	0.00	0.00	0.03	0.00	0.00	0.00	0.07	0.00	0.16	0.00	0.02	0.00	0.00	0.00	0.00	0.02	0.00	0.02	0.02
**GT22**	0.00	0.00	0.00	0.00	0.00	0.00	0.00	0.00	0.00	0.00	0.00	0.00	0.00	0.00	0.00	0.00	0.00	0.00	0.00	0.00	0.00
**PL12**	0.08	0.11	0.00	0.07	0.00	0.04	0.11	0.13	0.00	0.03	0.00	0.11	0.02	0.09	0.18	0.19	0.15	0.07	0.11	0.28	0.19
**PL18**	0.00	0.00	0.00	0.00	0.00	0.00	0.00	0.00	0.00	0.00	0.00	0.00	0.00	0.00	0.00	0.00	0.00	0.00	0.00	0.00	0.00
**GT92**	0.00	0.00	0.00	0.00	0.00	0.00	0.00	0.00	0.00	0.00	0.00	0.00	0.00	0.00	0.00	0.00	0.00	0.00	0.00	0.00	0.00
**GT44**	0.00	0.00	0.00	0.00	0.00	0.00	0.00	0.00	0.00	0.00	0.00	0.00	0.00	0.00	0.00	0.00	0.00	0.00	0.00	0.00	0.00
**GT29**	0.00	0.00	0.00	0.00	0.00	0.00	0.00	0.00	0.00	0.00	0.00	0.00	0.00	0.00	0.00	0.00	0.00	0.00	0.00	0.00	0.00
**GT13**	0.00	0.00	0.00	0.00	0.00	0.00	0.00	0.00	0.00	0.00	0.00	0.00	0.00	0.00	0.00	0.00	0.00	0.00	0.00	0.00	0.00
**GT52**	0.00	0.00	0.00	0.00	0.00	0.00	0.00	0.00	0.00	0.00	0.00	0.00	0.00	0.00	0.00	0.00	0.00	0.00	0.00	0.00	0.00
**GT64**	0.00	0.00	0.00	0.00	0.00	0.00	0.00	0.00	0.00	0.00	0.00	0.00	0.00	0.00	0.00	0.00	0.00	0.00	0.00	0.00	0.00
**GT85**	0.00	0.00	0.00	0.00	0.00	0.00	0.00	0.00	0.00	0.00	0.00	0.00	0.00	0.00	0.00	0.00	0.00	0.00	0.00	0.00	0.00
**GT16**	0.00	0.00	0.00	0.00	0.00	0.00	0.00	0.00	0.00	0.00	0.00	0.00	0.00	0.00	0.00	0.00	0.00	0.00	0.00	0.00	0.00
**GH56**	0.00	0.00	0.00	0.00	0.00	0.00	0.00	0.00	0.00	0.00	0.00	0.00	0.00	0.00	0.00	0.00	0.00	0.00	0.00	0.00	0.00
**GT60**	0.00	0.00	0.00	0.00	0.00	0.00	0.00	0.00	0.00	0.00	0.00	0.00	0.00	0.00	0.00	0.00	0.00	0.00	0.00	0.00	0.00
**GH41**	0.00	0.00	0.00	0.00	0.00	0.00	0.00	0.00	0.00	0.00	0.00	0.00	0.00	0.00	0.00	0.00	0.00	0.00	0.00	0.00	0.00
**CE7**	0.08	0.18	0.19	0.09	0.04	0.11	0.09	0.13	0.05	0.11	0.07	0.13	0.04	0.08	0.14	0.19	0.11	0.14	0.23	0.13	0.07
**GH39**	0.00	0.02	0.00	0.04	0.04	0.09	0.04	0.02	0.00	0.05	0.02	0.06	0.04	0.01	0.04	0.00	0.02	0.00	0.05	0.06	0.02
**GT30**	0.38	0.27	0.26	0.34	0.14	0.31	0.33	0.30	0.20	0.25	0.27	0.25	0.17	0.23	0.25	0.33	0.39	0.40	0.18	0.30	0.16
**PL15**	0.10	0.29	0.00	0.12	0.00	0.02	0.17	0.17	0.00	0.00	0.00	0.09	0.00	0.07	0.10	0.17	0.18	0.04	0.23	0.17	0.10
**GH102**	0.03	0.05	0.02	0.00	0.00	0.04	0.00	0.04	0.07	0.07	0.04	0.02	0.00	0.01	0.00	0.00	0.00	0.05	0.00	0.02	0.00

CAZY_group	CN-AD-22	CN-AD-23	CN-AD-24	CN-AD-25	CN-AD-26	CN-AD-27	CN-AD-28	CN-AD-29	CN-AD-30	CN-AD-31	CN-AD-32	CN-AD-33	CN-AD-34	CN-AD-35	CN-AD-36	CN-AD-37	CN-AD-38	CN-AD-39	CN-AD-40	CN-AD-41	CN-AD-42
**GH29**	0.34	0.79	0.67	0.53	0.73	0.52	0.75	0.98	0.44	0.54	0.39	0.56	0.56	0.69	0.09	0.36	0.38	0.59	0.74	0.34	0.37
**PL4**	0.00	0.00	0.00	0.00	0.00	0.00	0.00	0.00	0.00	0.00	0.00	0.00	0.00	0.03	0.00	0.00	0.01	0.00	0.00	0.00	0.00
**GH23**	0.99	1.11	1.13	1.22	0.92	1.02	1.15	0.89	0.98	0.98	0.78	1.03	0.88	1.04	1.13	0.95	0.90	1.11	0.87	0.93	0.90
**CE6**	0.00	0.05	0.06	0.06	0.03	0.02	0.03	0.05	0.02	0.02	0.01	0.03	0.00	0.07	0.06	0.02	0.01	0.00	0.07	0.01	0.04
**GH72**	0.10	0.02	0.06	0.11	0.04	0.06	0.08	0.05	0.10	0.04	0.05	0.07	0.08	0.07	0.03	0.06	0.03	0.04	0.05	0.05	0.07
**GH114**	0.00	0.00	0.00	0.00	0.00	0.00	0.00	0.00	0.00	0.00	0.00	0.00	0.00	0.00	0.00	0.00	0.00	0.00	0.00	0.00	0.00
**GH78**	0.22	0.62	0.99	0.59	0.92	0.94	0.92	0.43	0.41	0.43	0.96	0.76	0.43	1.17	0.22	0.32	0.28	0.63	0.85	0.31	0.74
**PL19**	0.00	0.00	0.00	0.00	0.00	0.00	0.00	0.00	0.00	0.00	0.00	0.00	0.00	0.00	0.00	0.00	0.00	0.00	0.00	0.00	0.00
**GT27**	0.00	0.00	0.00	0.00	0.00	0.00	0.00	0.00	0.00	0.00	0.00	0.00	0.00	0.00	0.00	0.00	0.00	0.00	0.00	0.00	0.00
**GH5**	0.22	0.35	0.22	0.08	0.26	0.23	0.07	0.28	0.34	0.27	0.12	0.20	0.27	0.51	0.03	0.13	0.49	0.24	0.08	0.26	0.19
**GT40**	0.00	0.00	0.00	0.00	0.00	0.00	0.00	0.00	0.00	0.00	0.00	0.00	0.00	0.00	0.00	0.00	0.00	0.00	0.00	0.00	0.00
**CE13**	0.00	0.00	0.00	0.00	0.00	0.00	0.00	0.00	0.00	0.00	0.00	0.00	0.00	0.00	0.00	0.00	0.00	0.00	0.00	0.00	0.00
**GT37**	0.00	0.00	0.00	0.00	0.00	0.00	0.00	0.00	0.00	0.00	0.00	0.00	0.00	0.00	0.00	0.00	0.00	0.00	0.00	0.00	0.00
**GH33**	0.17	0.34	0.18	0.25	0.22	0.27	0.27	0.20	0.14	0.17	0.20	0.27	0.16	0.26	0.09	0.13	0.09	0.26	0.27	0.11	0.07
**GT65**	0.00	0.00	0.00	0.00	0.00	0.00	0.00	0.00	0.00	0.00	0.00	0.00	0.00	0.00	0.00	0.00	0.00	0.00	0.00	0.00	0.00
**GH86**	0.00	0.00	0.00	0.00	0.00	0.00	0.00	0.00	0.00	0.00	0.00	0.02	0.02	0.01	0.00	0.00	0.00	0.00	0.01	0.00	0.00
**GH123**	0.00	0.17	0.18	0.08	0.10	0.14	0.14	0.07	0.03	0.09	0.12	0.08	0.08	0.09	0.00	0.02	0.05	0.11	0.14	0.06	0.09
**GH96**	0.00	0.00	0.00	0.00	0.00	0.00	0.00	0.00	0.00	0.00	0.00	0.00	0.00	0.00	0.00	0.00	0.00	0.00	0.00	0.00	0.00
**GH14**	0.00	0.00	0.00	0.00	0.00	0.00	0.00	0.00	0.00	0.00	0.00	0.00	0.00	0.00	0.00	0.00	0.00	0.00	0.00	0.00	0.00
**CE3**	0.00	0.00	0.00	0.00	0.00	0.00	0.00	0.00	0.00	0.00	0.00	0.00	0.00	0.00	0.00	0.00	0.00	0.00	0.00	0.00	0.00
**PL10**	0.02	0.05	0.18	0.00	0.13	0.12	0.13	0.08	0.03	0.04	0.09	0.07	0.03	0.15	0.00	0.06	0.03	0.04	0.10	0.03	0.04
**GT48**	0.00	0.00	0.00	0.00	0.00	0.00	0.00	0.00	0.00	0.00	0.00	0.00	0.00	0.00	0.00	0.00	0.00	0.00	0.00	0.00	0.00
**PL6**	0.00	0.02	0.00	0.00	0.00	0.02	0.00	0.00	0.00	0.00	0.00	0.00	0.00	0.01	0.00	0.00	0.00	0.00	0.00	0.00	0.00
**GT83**	0.17	0.19	0.16	0.17	0.12	0.12	0.10	0.08	0.19	0.02	0.12	0.10	0.08	0.05	0.06	0.13	0.03	0.07	0.13	0.06	0.07
**GH126**	0.00	0.00	0.00	0.00	0.00	0.00	0.00	0.00	0.00	0.00	0.00	0.00	0.00	0.00	0.00	0.00	0.00	0.00	0.00	0.00	0.00
**GH9**	0.19	0.22	0.12	0.06	0.11	0.05	0.03	0.15	0.17	0.13	0.03	0.05	0.22	0.04	0.00	0.00	0.29	0.15	0.11	0.16	0.04
**GH75**	0.00	0.00	0.00	0.00	0.00	0.00	0.00	0.00	0.00	0.00	0.00	0.00	0.00	0.00	0.00	0.00	0.00	0.00	0.00	0.00	0.00
**GT71**	0.00	0.00	0.00	0.00	0.00	0.00	0.00	0.00	0.00	0.00	0.00	0.00	0.00	0.00	0.00	0.00	0.00	0.00	0.00	0.00	0.00
**GH46**	0.00	0.00	0.00	0.00	0.00	0.00	0.00	0.00	0.00	0.00	0.00	0.00	0.00	0.00	0.00	0.00	0.00	0.00	0.00	0.00	0.00
**GT61**	0.00	0.00	0.00	0.00	0.00	0.00	0.00	0.00	0.00	0.00	0.00	0.00	0.00	0.00	0.00	0.00	0.00	0.00	0.00	0.00	0.00
**GT3**	0.02	0.08	0.20	0.08	0.11	0.08	0.10	0.17	0.10	0.13	0.05	0.14	0.10	0.11	0.00	0.02	0.01	0.09	0.10	0.06	0.09
**PL3**	0.00	0.00	0.00	0.00	0.00	0.00	0.00	0.00	0.00	0.00	0.00	0.00	0.00	0.00	0.00	0.00	0.00	0.00	0.00	0.00	0.00
**GH108**	0.05	0.03	0.08	0.02	0.04	0.03	0.01	0.07	0.02	0.04	0.05	0.00	0.02	0.08	0.00	0.00	0.03	0.02	0.02	0.03	0.02
**GT56**	0.02	0.02	0.04	0.02	0.00	0.02	0.01	0.00	0.00	0.00	0.01	0.00	0.01	0.01	0.06	0.00	0.03	0.00	0.00	0.01	0.04
**PL11**	0.02	0.12	0.24	0.04	0.26	0.09	0.24	0.13	0.08	0.13	0.24	0.10	0.05	0.23	0.03	0.06	0.03	0.09	0.09	0.05	0.11
**GH45**	0.00	0.00	0.00	0.00	0.00	0.00	0.00	0.00	0.00	0.00	0.00	0.00	0.00	0.00	0.00	0.00	0.00	0.02	0.00	0.00	0.00
**GT34**	0.00	0.00	0.00	0.02	0.00	0.00	0.00	0.00	0.00	0.00	0.00	0.00	0.00	0.00	0.03	0.02	0.00	0.00	0.00	0.00	0.00
**PL14**	0.00	0.00	0.00	0.00	0.00	0.00	0.00	0.00	0.00	0.00	0.00	0.00	0.00	0.00	0.00	0.00	0.00	0.00	0.00	0.00	0.00
**GH19**	0.02	0.00	0.02	0.02	0.00	0.00	0.00	0.00	0.00	0.01	0.03	0.00	0.01	0.03	0.03	0.00	0.00	0.00	0.00	0.03	0.00
**GH118**	0.00	0.00	0.00	0.00	0.00	0.00	0.00	0.00	0.00	0.00	0.00	0.00	0.00	0.00	0.00	0.00	0.00	0.00	0.00	0.00	0.00
**GH82**	0.00	0.00	0.00	0.00	0.00	0.00	0.00	0.00	0.00	0.00	0.00	0.00	0.00	0.00	0.00	0.00	0.00	0.00	0.00	0.00	0.00
**GH76**	0.02	0.28	0.22	0.15	0.16	0.23	0.24	0.03	0.03	0.02	0.32	0.46	0.08	0.31	0.00	0.04	0.03	0.17	0.25	0.04	0.16
**GT4**	0.82	1.73	1.80	1.72	1.94	1.58	1.41	1.34	1.29	1.32	1.31	1.76	1.23	1.51	0.69	1.48	1.37	1.60	1.72	1.30	1.39
**GT87**	0.00	0.00	0.00	0.00	0.00	0.00	0.00	0.00	0.00	0.00	0.00	0.00	0.01	0.00	0.00	0.00	0.00	0.00	0.00	0.01	0.00
**GH24**	0.22	0.19	0.26	0.15	0.21	0.27	0.18	0.23	0.14	0.10	0.20	0.20	0.19	0.18	0.13	0.08	0.12	0.17	0.21	0.18	0.16
**GH90**	0.00	0.00	0.00	0.00	0.00	0.00	0.00	0.00	0.00	0.00	0.00	0.00	0.00	0.00	0.00	0.00	0.00	0.00	0.00	0.00	0.00
**GH57**	0.02	0.17	0.20	0.08	0.09	0.12	0.11	0.12	0.07	0.09	0.18	0.08	0.15	0.08	0.00	0.02	0.09	0.13	0.12	0.05	0.07
**GH36**	0.41	0.66	0.67	0.80	0.66	0.74	0.71	0.56	0.56	0.34	0.51	0.46	0.51	0.65	0.32	0.76	0.55	0.54	0.76	0.45	0.51
**GT46**	0.00	0.00	0.00	0.00	0.00	0.00	0.00	0.00	0.00	0.00	0.00	0.00	0.00	0.00	0.00	0.00	0.00	0.00	0.00	0.00	0.00
**GH66**	0.02	0.03	0.08	0.04	0.03	0.02	0.04	0.03	0.02	0.00	0.05	0.05	0.02	0.04	0.00	0.00	0.03	0.04	0.05	0.00	0.09
**GH48**	0.00	0.00	0.00	0.00	0.00	0.00	0.00	0.00	0.00	0.00	0.00	0.00	0.00	0.00	0.00	0.00	0.00	0.00	0.00	0.03	0.00
**GT53**	0.00	0.00	0.00	0.00	0.00	0.00	0.00	0.00	0.00	0.00	0.00	0.00	0.00	0.00	0.00	0.00	0.00	0.00	0.00	0.00	0.00
**GH60**	0.00	0.00	0.00	0.00	0.00	0.00	0.00	0.00	0.00	0.00	0.00	0.00	0.00	0.00	0.00	0.00	0.00	0.00	0.00	0.00	0.00

CAZY_group	CN-AD-22	CN-AD-23	CN-AD-24	CN-AD-25	CN-AD-26	CN-AD-27	CN-AD-28	CN-AD-29	CN-AD-30	CN-AD-31	CN-AD-32	CN-AD-33	CN-AD-34	CN-AD-35	CN-AD-36	CN-AD-37	CN-AD-38	CN-AD-39	CN-AD-40	CN-AD-41	CN-AD-42
**GT31**	0.00	0.00	0.00	0.02	0.00	0.00	0.00	0.00	0.00	0.00	0.00	0.00	0.00	0.00	0.03	0.00	0.00	0.00	0.00	0.01	0.02
**GH105**	0.12	0.57	0.75	0.17	0.68	0.67	0.62	0.55	0.32	0.40	0.43	0.39	0.22	0.59	0.25	0.25	0.28	0.69	0.53	0.21	0.21
**GT23**	0.00	0.00	0.00	0.00	0.01	0.02	0.01	0.00	0.00	0.01	0.00	0.00	0.00	0.00	0.00	0.02	0.01	0.00	0.00	0.00	0.00
**GH11**	0.00	0.00	0.00	0.00	0.00	0.00	0.00	0.00	0.00	0.00	0.00	0.00	0.00	0.00	0.00	0.00	0.00	0.00	0.00	0.00	0.00
**GT38**	0.00	0.00	0.00	0.00	0.00	0.00	0.00	0.00	0.00	0.00	0.00	0.00	0.00	0.00	0.00	0.00	0.00	0.00	0.00	0.00	0.00
**GT43**	0.00	0.00	0.00	0.00	0.00	0.00	0.00	0.00	0.00	0.00	0.00	0.00	0.00	0.00	0.00	0.00	0.00	0.00	0.00	0.00	0.00
**GH99**	0.00	0.00	0.04	0.02	0.00	0.02	0.00	0.00	0.00	0.00	0.00	0.02	0.01	0.04	0.00	0.00	0.00	0.04	0.02	0.00	0.00
**GH38**	0.27	0.20	0.18	0.19	0.24	0.17	0.22	0.08	0.29	0.13	0.13	0.34	0.19	0.22	0.25	0.15	0.13	0.35	0.15	0.16	0.12
**GH109**	0.17	0.23	0.32	0.15	0.25	0.24	0.29	0.31	0.10	0.16	0.12	0.29	0.27	0.16	0.00	0.11	0.09	0.22	0.17	0.14	0.05
**GH2**	2.04	3.87	4.64	2.86	3.63	3.49	3.62	3.41	2.40	2.43	3.64	3.80	2.23	3.71	0.95	2.19	2.89	3.47	3.95	2.13	2.57
**GT28**	0.41	0.39	0.30	0.46	0.25	0.32	0.29	0.30	0.42	0.28	0.16	0.39	0.23	0.35	0.22	0.30	0.32	0.20	0.32	0.28	0.26
**GH42**	0.10	0.17	0.04	0.27	0.10	0.12	0.18	0.10	0.34	0.10	0.05	0.08	0.16	0.22	0.09	0.34	0.29	0.17	0.19	0.18	0.16
**GT58**	0.00	0.00	0.00	0.00	0.00	0.00	0.00	0.00	0.00	0.00	0.00	0.00	0.00	0.00	0.00	0.00	0.00	0.00	0.00	0.00	0.00
**GH61**	0.00	0.00	0.00	0.00	0.00	0.00	0.00	0.00	0.00	0.00	0.00	0.00	0.00	0.00	0.00	0.00	0.00	0.00	0.00	0.00	0.00
**GH80**	0.00	0.00	0.00	0.00	0.00	0.00	0.00	0.00	0.00	0.00	0.00	0.00	0.00	0.00	0.00	0.00	0.00	0.00	0.00	0.00	0.00
**GH98**	0.00	0.00	0.00	0.00	0.00	0.02	0.00	0.00	0.00	0.04	0.01	0.00	0.00	0.01	0.00	0.00	0.00	0.00	0.00	0.00	0.00
**GH6**	0.00	0.00	0.00	0.00	0.00	0.00	0.00	0.00	0.00	0.00	0.00	0.00	0.00	0.00	0.00	0.00	0.00	0.00	0.00	0.00	0.00
**GT1**	0.00	0.02	0.04	0.08	0.05	0.02	0.03	0.02	0.00	0.00	0.03	0.05	0.05	0.03	0.00	0.02	0.05	0.07	0.01	0.04	0.04
**GH28**	0.29	0.72	1.05	0.27	0.99	0.89	0.98	0.71	0.39	0.51	0.72	0.76	0.19	0.97	0.22	0.55	0.30	0.46	0.63	0.34	0.48
**GT42**	0.00	0.00	0.00	0.00	0.00	0.00	0.00	0.00	0.00	0.00	0.00	0.00	0.00	0.00	0.00	0.00	0.00	0.00	0.00	0.00	0.00
**GH128**	0.00	0.00	0.00	0.00	0.00	0.00	0.00	0.00	0.00	0.01	0.00	0.00	0.00	0.00	0.00	0.00	0.00	0.00	0.00	0.00	0.00
**CE12**	0.05	0.19	0.14	0.02	0.22	0.06	0.24	0.21	0.15	0.10	0.16	0.07	0.02	0.11	0.00	0.06	0.04	0.04	0.14	0.05	0.02
**CE16**	0.00	0.00	0.00	0.00	0.00	0.00	0.00	0.00	0.00	0.00	0.00	0.00	0.00	0.00	0.00	0.00	0.00	0.00	0.00	0.00	0.00
**GT89**	0.00	0.00	0.00	0.00	0.00	0.00	0.00	0.00	0.00	0.00	0.00	0.00	0.00	0.00	0.00	0.00	0.00	0.00	0.00	0.00	0.00
**GT11**	0.02	0.09	0.04	0.02	0.09	0.06	0.04	0.07	0.03	0.06	0.03	0.12	0.06	0.03	0.00	0.04	0.03	0.04	0.05	0.01	0.02
**GT77**	0.02	0.00	0.00	0.02	0.00	0.03	0.00	0.02	0.00	0.00	0.00	0.02	0.00	0.01	0.03	0.00	0.00	0.00	0.01	0.00	0.02
**GT55**	0.00	0.00	0.00	0.00	0.00	0.00	0.00	0.00	0.00	0.00	0.00	0.00	0.00	0.00	0.00	0.00	0.00	0.00	0.00	0.00	0.00
**GT18**	0.00	0.00	0.00	0.00	0.00	0.00	0.00	0.00	0.00	0.00	0.00	0.00	0.00	0.00	0.00	0.00	0.00	0.00	0.00	0.00	0.00
**GH87**	0.00	0.00	0.02	0.00	0.00	0.00	0.00	0.00	0.00	0.02	0.00	0.00	0.01	0.00	0.00	0.00	0.00	0.04	0.00	0.00	0.00
**PL5**	0.00	0.00	0.00	0.00	0.00	0.00	0.00	0.00	0.00	0.00	0.00	0.00	0.00	0.00	0.00	0.00	0.00	0.00	0.00	0.00	0.00
**GH22**	0.00	0.00	0.00	0.00	0.00	0.00	0.00	0.00	0.00	0.00	0.00	0.00	0.00	0.00	0.00	0.00	0.00	0.00	0.00	0.00	0.00
**CE2**	0.00	0.03	0.02	0.02	0.04	0.05	0.04	0.03	0.00	0.02	0.00	0.03	0.05	0.04	0.00	0.00	0.09	0.09	0.02	0.01	0.00
**GH120**	0.02	0.05	0.00	0.11	0.00	0.00	0.06	0.00	0.10	0.02	0.00	0.00	0.05	0.11	0.06	0.19	0.12	0.07	0.03	0.10	0.05
**GH73**	0.39	0.45	0.30	0.63	0.36	0.26	0.35	0.41	0.39	0.34	0.28	0.37	0.40	0.47	0.44	0.42	0.29	0.33	0.32	0.30	0.40
**GT17**	0.00	0.00	0.00	0.00	0.00	0.00	0.00	0.00	0.00	0.00	0.00	0.00	0.00	0.00	0.00	0.00	0.03	0.00	0.00	0.00	0.00
**GH124**	0.00	0.00	0.00	0.00	0.00	0.00	0.00	0.00	0.00	0.00	0.00	0.00	0.00	0.00	0.00	0.00	0.00	0.00	0.00	0.00	0.00
**GH54**	0.00	0.00	0.00	0.00	0.00	0.00	0.00	0.00	0.00	0.00	0.00	0.00	0.00	0.00	0.00	0.00	0.00	0.00	0.00	0.00	0.00
**GH100**	0.00	0.00	0.00	0.00	0.00	0.00	0.00	0.00	0.00	0.00	0.00	0.00	0.00	0.00	0.00	0.00	0.00	0.00	0.00	0.00	0.00
**GH113**	0.02	0.05	0.00	0.02	0.01	0.02	0.04	0.02	0.00	0.01	0.01	0.00	0.00	0.01	0.00	0.00	0.01	0.00	0.00	0.00	0.02
**GT5**	0.24	0.29	0.22	0.32	0.22	0.26	0.25	0.36	0.44	0.31	0.12	0.30	0.22	0.31	0.13	0.25	0.32	0.33	0.24	0.30	0.14
**GH94**	0.10	0.17	0.08	0.06	0.16	0.18	0.15	0.26	0.25	0.29	0.08	0.24	0.18	0.26	0.06	0.25	0.51	0.30	0.10	0.33	0.04
**GT66**	0.00	0.00	0.00	0.00	0.00	0.00	0.01	0.00	0.00	0.00	0.00	0.00	0.02	0.00	0.00	0.00	0.00	0.00	0.00	0.00	0.02
**GT94**	0.00	0.00	0.00	0.00	0.00	0.00	0.00	0.00	0.00	0.00	0.00	0.00	0.00	0.00	0.00	0.00	0.00	0.00	0.00	0.00	0.00
**GH58**	0.02	0.00	0.00	0.04	0.00	0.00	0.00	0.00	0.00	0.00	0.00	0.00	0.00	0.00	0.00	0.00	0.00	0.00	0.00	0.00	0.02
**GH1**	0.32	1.16	0.61	1.28	0.15	0.35	0.52	0.41	0.56	0.36	0.08	0.30	0.20	0.26	0.91	0.40	0.49	0.13	0.10	0.38	0.42
**PL22**	0.00	0.00	0.00	0.02	0.00	0.00	0.01	0.00	0.00	0.00	0.00	0.00	0.00	0.00	0.00	0.00	0.01	0.00	0.00	0.00	0.02
**GT9**	0.41	0.20	0.22	0.08	0.16	0.18	0.13	0.12	0.12	0.11	0.12	0.17	0.11	0.15	0.09	0.04	0.04	0.15	0.11	0.09	0.26
**GT76**	0.00	0.00	0.00	0.00	0.00	0.00	0.00	0.00	0.00	0.00	0.00	0.00	0.00	0.00	0.00	0.00	0.00	0.00	0.00	0.00	0.00
**GT32**	0.05	0.08	0.12	0.15	0.10	0.03	0.04	0.08	0.03	0.07	0.05	0.07	0.07	0.05	0.00	0.13	0.05	0.13	0.12	0.08	0.02
**GT47**	0.02	0.02	0.02	0.06	0.01	0.00	0.06	0.05	0.02	0.05	0.00	0.00	0.05	0.04	0.00	0.00	0.00	0.11	0.02	0.01	0.02
**GH84**	0.07	0.28	0.20	0.13	0.14	0.09	0.11	0.17	0.07	0.09	0.08	0.10	0.12	0.11	0.00	0.11	0.07	0.15	0.12	0.08	0.09
**GH83**	0.00	0.00	0.00	0.00	0.00	0.00	0.00	0.00	0.00	0.00	0.00	0.00	0.00	0.00	0.00	0.00	0.00	0.00	0.00	0.00	0.00
**GT59**	0.00	0.00	0.00	0.00	0.00	0.00	0.00	0.00	0.00	0.00	0.00	0.00	0.00	0.00	0.00	0.00	0.00	0.00	0.00	0.00	0.00

CAZY_group	CN-AD-22	CN-AD-23	CN-AD-24	CN-AD-25	CN-AD-26	CN-AD-27	CN-AD-28	CN-AD-29	CN-AD-30	CN-AD-31	CN-AD-32	CN-AD-33	CN-AD-34	CN-AD-35	CN-AD-36	CN-AD-37	CN-AD-38	CN-AD-39	CN-AD-40	CN-AD-41	CN-AD-42
GH34	0.00	0.00	0.00	0.00	0.00	0.00	0.00	0.00	0.00	0.00	0.00	0.00	0.00	0.00	0.00	0.00	0.00	0.00	0.00	0.00	0.00
GT14	0.07	0.05	0.04	0.06	0.01	0.00	0.01	0.02	0.00	0.01	0.00	0.05	0.02	0.04	0.00	0.00	0.03	0.07	0.02	0.01	0.00
GH47	0.00	0.00	0.00	0.00	0.00	0.00	0.01	0.00	0.00	0.00	0.00	0.00	0.00	0.00	0.00	0.00	0.00	0.00	0.00	0.00	0.00
GH97	0.34	0.79	0.97	0.42	1.09	0.89	0.91	0.78	0.42	0.47	0.70	1.01	0.38	0.81	0.00	0.21	0.48	0.76	0.89	0.23	0.44
GH50	0.00	0.12	0.06	0.00	0.07	0.11	0.06	0.00	0.00	0.02	0.07	0.05	0.02	0.11	0.00	0.02	0.00	0.02	0.07	0.00	0.04
GT26	0.17	0.23	0.16	0.13	0.16	0.14	0.17	0.15	0.15	0.21	0.12	0.15	0.09	0.16	0.09	0.15	0.13	0.15	0.10	0.11	0.12
GH18	0.27	0.65	0.75	0.55	0.37	0.41	0.73	0.31	0.37	0.59	0.55	0.71	0.48	0.65	0.44	0.25	0.33	0.28	0.53	0.23	0.51
GH37	0.05	0.05	0.04	0.06	0.00	0.02	0.00	0.00	0.00	0.00	0.03	0.00	0.01	0.01	0.13	0.00	0.07	0.00	0.00	0.03	0.04
PL13	0.00	0.06	0.02	0.00	0.03	0.05	0.06	0.05	0.00	0.05	0.07	0.03	0.01	0.05	0.00	0.00	0.01	0.02	0.04	0.01	0.02
GH27	0.05	0.15	0.18	0.08	0.16	0.15	0.17	0.15	0.08	0.15	0.18	0.24	0.15	0.16	0.00	0.13	0.11	0.28	0.21	0.09	0.05
GT54	0.00	0.00	0.00	0.00	0.00	0.00	0.00	0.00	0.00	0.00	0.00	0.00	0.00	0.00	0.00	0.00	0.00	0.00	0.00	0.00	0.00
GT91	0.00	0.00	0.00	0.00	0.00	0.00	0.00	0.00	0.00	0.00	0.00	0.00	0.00	0.00	0.00	0.00	0.00	0.00	0.00	0.00	0.00
GT72	0.00	0.00	0.00	0.00	0.00	0.00	0.00	0.00	0.00	0.00	0.00	0.00	0.00	0.00	0.00	0.00	0.00	0.00	0.00	0.00	0.00
GH67	0.00	0.05	0.06	0.00	0.09	0.09	0.13	0.08	0.08	0.07	0.05	0.05	0.02	0.04	0.03	0.02	0.04	0.09	0.09	0.04	0.04
GH12	0.00	0.00	0.00	0.00	0.00	0.00	0.00	0.00	0.00	0.00	0.00	0.00	0.00	0.00	0.00	0.00	0.00	0.00	0.00	0.00	0.00
GH91	0.00	0.08	0.04	0.00	0.04	0.06	0.01	0.00	0.02	0.00	0.04	0.03	0.00	0.01	0.00	0.00	0.01	0.00	0.02	0.04	0.04
GT69	0.00	0.00	0.00	0.00	0.00	0.00	0.00	0.00	0.00	0.00	0.00	0.00	0.00	0.00	0.00	0.00	0.00	0.00	0.00	0.00	0.00
GH106	0.05	0.14	0.22	0.04	0.31	0.26	0.27	0.17	0.14	0.13	0.32	0.20	0.12	0.26	0.00	0.11	0.12	0.13	0.24	0.04	0.18
GT62	0.00	0.00	0.00	0.00	0.00	0.00	0.00	0.00	0.00	0.00	0.00	0.00	0.00	0.00	0.00	0.00	0.00	0.00	0.00	0.00	0.00
GH17	0.00	0.00	0.00	0.00	0.00	0.00	0.00	0.00	0.00	0.00	0.00	0.00	0.00	0.00	0.00	0.00	0.00	0.00	0.00	0.00	0.00
GH88	0.19	0.48	0.52	0.21	0.47	0.24	0.32	0.26	0.14	0.28	0.34	0.24	0.16	0.40	0.44	0.11	0.08	0.35	0.41	0.11	0.30
CE5	0.00	0.00	0.00	0.00	0.00	0.00	0.00	0.00	0.00	0.00	0.00	0.00	0.00	0.00	0.00	0.00	0.00	0.00	0.00	0.00	0.00
GT8	0.17	0.12	0.10	0.19	0.05	0.12	0.07	0.18	0.20	0.07	0.08	0.05	0.02	0.01	0.03	0.08	0.05	0.20	0.10	0.09	0.09
GT50	0.00	0.00	0.00	0.00	0.00	0.00	0.00	0.00	0.00	0.00	0.00	0.00	0.00	0.00	0.00	0.00	0.00	0.00	0.00	0.00	0.00
GT20	0.10	0.09	0.10	0.11	0.07	0.11	0.11	0.08	0.00	0.04	0.11	0.07	0.04	0.05	0.06	0.02	0.05	0.07	0.07	0.08	0.09
PL9	0.02	0.08	0.10	0.13	0.14	0.08	0.17	0.23	0.12	0.10	0.09	0.05	0.02	0.08	0.03	0.02	0.07	0.04	0.07	0.20	0.02
GH55	0.02	0.05	0.02	0.00	0.01	0.03	0.00	0.05	0.02	0.05	0.00	0.08	0.02	0.01	0.00	0.00	0.08	0.04	0.01	0.01	0.00
GH64	0.00	0.00	0.00	0.00	0.00	0.02	0.00	0.00	0.00	0.00	0.00	0.00	0.00	0.00	0.00	0.00	0.00	0.00	0.00	0.00	0.00
GH127	0.19	0.19	0.24	0.29	0.23	0.35	0.18	0.17	0.27	0.10	0.22	0.22	0.12	0.32	0.09	0.42	0.18	0.28	0.31	0.20	0.11
GH103	0.02	0.02	0.02	0.04	0.00	0.02	0.00	0.00	0.00	0.00	0.00	0.00	0.00	0.00	0.06	0.00	0.03	0.00	0.00	0.01	0.04
GH3	1.62	2.65	2.52	2.25	2.24	1.97	2.33	2.02	2.15	1.91	1.84	2.04	1.32	2.17	0.85	1.75	2.10	2.02	2.44	1.45	1.95
GT45	0.00	0.00	0.02	0.00	0.00	0.00	0.00	0.00	0.00	0.00	0.00	0.00	0.00	0.00	0.00	0.00	0.00	0.00	0.00	0.00	0.00
GT74	0.00	0.00	0.00	0.00	0.00	0.00	0.00	0.00	0.00	0.00	0.00	0.00	0.00	0.00	0.00	0.00	0.00	0.00	0.00	0.00	0.00
GT90	0.00	0.00	0.00	0.00	0.00	0.00	0.00	0.00	0.00	0.00	0.00	0.00	0.00	0.01	0.00	0.00	0.00	0.00	0.00	0.00	0.00
GH116	0.02	0.06	0.06	0.06	0.07	0.02	0.04	0.12	0.00	0.02	0.07	0.12	0.10	0.03	0.00	0.00	0.03	0.07	0.07	0.04	0.02
GH31	0.46	1.20	0.95	0.84	0.81	0.92	0.98	0.88	0.86	0.65	0.86	1.00	0.55	0.90	0.63	0.78	1.00	0.85	1.03	0.74	1.00
GT12	0.00	0.00	0.00	0.00	0.00	0.00	0.00	0.00	0.00	0.00	0.00	0.00	0.00	0.00	0.00	0.00	0.00	0.00	0.00	0.00	0.00
CE1	0.29	0.34	0.34	0.29	0.43	0.33	0.29	0.28	0.17	0.21	0.32	0.32	0.17	0.26	0.09	0.15	0.20	0.35	0.32	0.21	0.21
GH121	0.00	0.00	0.00	0.02	0.00	0.00	0.00	0.00	0.02	0.02	0.01	0.00	0.00	0.00	0.00	0.11	0.00	0.00	0.00	0.00	0.00
GH79	0.00	0.00	0.00	0.00	0.00	0.00	0.00	0.00	0.00	0.00	0.00	0.00	0.00	0.01	0.00	0.00	0.01	0.00	0.00	0.00	0.00
GT73	0.00	0.00	0.00	0.00	0.00	0.00	0.00	0.00	0.00	0.00	0.00	0.00	0.00	0.00	0.00	0.00	0.00	0.00	0.00	0.00	0.00
GH77	0.32	0.35	0.50	0.32	0.46	0.52	0.35	0.51	0.54	0.55	0.30	0.59	0.28	0.58	0.22	0.61	0.42	0.39	0.35	0.56	0.30
PL17	0.00	0.00	0.02	0.00	0.01	0.02	0.01	0.00	0.02	0.01	0.00	0.00	0.00	0.00	0.00	0.06	0.01	0.02	0.00	0.00	0.00
PL8	0.07	0.48	0.50	0.04	0.29	0.15	0.24	0.21	0.07	0.17	0.36	0.42	0.12	0.23	0.06	0.00	0.11	0.13	0.41	0.11	0.19
CE9	0.22	0.45	0.50	0.50	0.26	0.39	0.39	0.38	0.42	0.28	0.27	0.34	0.16	0.36	0.22	0.25	0.28	0.22	0.27	0.25	0.30
CE15	0.00	0.03	0.00	0.00	0.01	0.00	0.00	0.00	0.00	0.02	0.00	0.00	0.01	0.00	0.00	0.00	0.01	0.02	0.04	0.00	0.00
GT79	0.00	0.00	0.00	0.00	0.00	0.00	0.00	0.00	0.00	0.00	0.00	0.00	0.00	0.00	0.00	0.00	0.00	0.00	0.00	0.00	0.00
GT10	0.00	0.02	0.00	0.00	0.00	0.00	0.00	0.00	0.00	0.01	0.01	0.00	0.02	0.00	0.00	0.00	0.00	0.00	0.01	0.01	0.00
GH119	0.00	0.00	0.00	0.00	0.00	0.00	0.00	0.00	0.00	0.00	0.00	0.00	0.00	0.00	0.00	0.00	0.00	0.00	0.00	0.00	0.00
GH16	0.19	0.32	0.18	0.25	0.25	0.21	0.10	0.13	0.05	0.16	0.18	0.34	0.16	0.11	0.00	0.06	0.09	0.22	0.29	0.08	0.14
GH43	0.85	2.01	2.42	1.01	2.65	1.82	1.83	1.52	1.15	1.30	1.96	1.79	0.77	2.00	0.47	1.60	1.47	1.67	2.14	0.78	1.14
GH51	0.15	0.39	0.48	0.46	0.49	0.38	0.63	0.40	0.46	0.25	0.42	0.46	0.30	0.59	0.16	0.42	0.46	0.35	0.49	0.30	0.39
GT35	0.48	0.85	0.61	0.95	0.54	0.61	0.73	0.66	0.79	0.65	0.42	0.54	0.55	0.73	0.54	0.72	0.99	0.54	0.58	0.74	0.67

CAZY_group	CN-AD-22	CN-AD-23	CN-AD-24	CN-AD-25	CN-AD-26	CN-AD-27	CN-AD-28	CN-AD-29	CN-AD-30	CN-AD-31	CN-AD-32	CN-AD-33	CN-AD-34	CN-AD-35	CN-AD-36	CN-AD-37	CN-AD-38	CN-AD-39	CN-AD-40	CN-AD-41	CN-AD-42
GT25	0.00	0.00	0.02	0.00	0.00	0.00	0.00	0.00	0.00	0.00	0.01	0.00	0.00	0.00	0.00	0.00	0.00	0.00	0.00	0.00	0.00
GT2	2.52	3.93	4.13	3.85	3.83	3.56	3.75	3.54	2.86	3.03	2.73	3.66	2.57	3.49	1.51	3.04	2.79	3.47	3.65	2.98	2.69
GT51	0.75	0.96	0.95	0.90	0.85	0.74	0.73	0.86	0.79	0.56	0.77	0.79	0.45	0.69	0.63	0.59	0.74	0.67	0.87	0.59	0.77
GH107	0.00	0.00	0.00	0.00	0.00	0.00	0.00	0.00	0.00	0.00	0.00	0.00	0.00	0.00	0.00	0.00	0.00	0.00	0.00	0.00	0.00
GH7	0.00	0.00	0.00	0.00	0.00	0.00	0.00	0.00	0.00	0.00	0.00	0.00	0.00	0.00	0.00	0.00	0.00	0.00	0.00	0.00	0.00
GT7	0.00	0.00	0.00	0.00	0.00	0.00	0.00	0.00	0.00	0.00	0.00	0.00	0.00	0.00	0.00	0.00	0.00	0.00	0.00	0.00	0.00
GT36	0.00	0.00	0.00	0.00	0.00	0.00	0.00	0.00	0.00	0.00	0.00	0.00	0.00	0.00	0.00	0.00	0.00	0.00	0.00	0.00	0.00
PL7	0.00	0.00	0.00	0.00	0.00	0.00	0.00	0.00	0.00	0.00	0.00	0.00	0.00	0.00	0.00	0.00	0.00	0.00	0.00	0.00	0.00
GH110	0.05	0.12	0.18	0.15	0.13	0.11	0.08	0.15	0.07	0.09	0.13	0.15	0.12	0.11	0.00	0.08	0.07	0.24	0.13	0.06	0.05
GT21	0.00	0.00	0.00	0.00	0.00	0.00	0.00	0.00	0.00	0.00	0.00	0.00	0.00	0.00	0.00	0.00	0.00	0.00	0.00	0.00	0.00
GH62	0.00	0.00	0.00	0.00	0.00	0.00	0.00	0.00	0.00	0.00	0.00	0.00	0.00	0.00	0.00	0.00	0.00	0.00	0.00	0.00	0.00
GH125	0.22	0.23	0.20	0.11	0.13	0.09	0.21	0.12	0.03	0.12	0.13	0.14	0.11	0.15	0.03	0.08	0.07	0.09	0.14	0.05	0.14
GH117	0.00	0.09	0.18	0.02	0.09	0.09	0.11	0.13	0.00	0.01	0.07	0.14	0.07	0.09	0.00	0.04	0.03	0.09	0.14	0.04	0.04
GH68	0.00	0.00	0.00	0.02	0.00	0.00	0.00	0.00	0.00	0.00	0.00	0.00	0.00	0.00	0.00	0.00	0.00	0.00	0.00	0.00	0.00
GH85	0.00	0.08	0.00	0.04	0.01	0.00	0.00	0.02	0.02	0.00	0.00	0.00	0.02	0.00	0.00	0.06	0.00	0.00	0.01	0.01	0.00
GH92	0.53	1.59	2.42	1.26	1.75	1.44	1.68	1.06	0.51	0.80	1.89	1.82	0.86	1.73	0.03	0.61	0.57	1.15	2.11	0.53	1.11
GH40	0.00	0.00	0.00	0.00	0.00	0.00	0.00	0.00	0.00	0.00	0.00	0.00	0.00	0.00	0.00	0.00	0.00	0.00	0.00	0.00	0.00
GT49	0.00	0.00	0.00	0.00	0.00	0.00	0.00	0.00	0.00	0.00	0.00	0.00	0.00	0.00	0.00	0.00	0.00	0.00	0.00	0.00	0.00
GH112	0.07	0.12	0.06	0.19	0.10	0.14	0.20	0.13	0.24	0.09	0.03	0.08	0.10	0.19	0.25	0.17	0.17	0.13	0.03	0.10	0.12
GT67	0.00	0.00	0.00	0.00	0.00	0.00	0.00	0.00	0.00	0.00	0.00	0.00	0.00	0.00	0.00	0.00	0.00	0.00	0.00	0.00	0.00
CE8	0.05	0.20	0.26	0.11	0.22	0.24	0.22	0.15	0.08	0.15	0.26	0.32	0.03	0.23	0.06	0.13	0.11	0.09	0.22	0.15	0.09
GT86	0.00	0.00	0.00	0.00	0.00	0.00	0.00	0.00	0.00	0.00	0.00	0.00	0.00	0.00	0.00	0.00	0.00	0.00	0.00	0.00	0.00
GH101	0.02	0.05	0.00	0.06	0.00	0.02	0.01	0.00	0.05	0.00	0.00	0.03	0.00	0.00	0.00	0.06	0.00	0.00	0.00	0.04	0.05
GH81	0.00	0.00	0.00	0.00	0.01	0.00	0.00	0.00	0.00	0.01	0.00	0.00	0.00	0.00	0.00	0.00	0.00	0.02	0.00	0.00	0.00
GT93	0.00	0.00	0.00	0.00	0.00	0.00	0.00	0.00	0.00	0.00	0.00	0.00	0.00	0.00	0.00	0.00	0.00	0.00	0.00	0.00	0.00
GT63	0.00	0.00	0.00	0.00	0.00	0.00	0.00	0.00	0.00	0.00	0.00	0.00	0.00	0.00	0.00	0.00	0.00	0.00	0.00	0.00	0.00
CE11	0.24	0.22	0.24	0.19	0.29	0.21	0.21	0.23	0.25	0.12	0.15	0.22	0.14	0.15	0.09	0.15	0.11	0.20	0.22	0.10	0.14
GT19	0.22	0.19	0.18	0.11	0.18	0.12	0.07	0.15	0.15	0.06	0.13	0.07	0.09	0.11	0.03	0.02	0.09	0.11	0.10	0.09	0.11
GT33	0.00	0.00	0.00	0.00	0.00	0.00	0.00	0.00	0.00	0.00	0.00	0.00	0.00	0.00	0.00	0.00	0.00	0.00	0.00	0.00	0.00
GH21	0.00	0.00	0.00	0.00	0.00	0.00	0.00	0.00	0.00	0.00	0.00	0.00	0.00	0.00	0.00	0.00	0.00	0.00	0.00	0.00	0.00
GT68	0.00	0.00	0.00	0.00	0.00	0.00	0.00	0.00	0.00	0.00	0.00	0.00	0.00	0.00	0.00	0.00	0.00	0.00	0.00	0.00	0.00
GH89	0.05	0.34	0.30	0.15	0.18	0.27	0.25	0.28	0.07	0.16	0.31	0.34	0.11	0.30	0.00	0.15	0.08	0.15	0.28	0.11	0.18
GT82	0.00	0.00	0.00	0.00	0.00	0.00	0.00	0.00	0.00	0.00	0.00	0.00	0.00	0.00	0.00	0.00	0.00	0.00	0.00	0.00	0.00
GH30	0.12	0.12	0.32	0.15	0.31	0.20	0.15	0.12	0.14	0.21	0.22	0.19	0.15	0.20	0.03	0.17	0.16	0.28	0.22	0.09	0.18
GH20	0.34	1.25	1.21	0.95	1.21	1.05	1.13	1.14	0.30	0.76	1.05	0.96	0.88	1.20	0.00	0.40	0.33	1.24	1.55	0.61	0.58
GH52	0.00	0.00	0.00	0.00	0.00	0.00	0.00	0.00	0.00	0.00	0.00	0.00	0.00	0.00	0.00	0.00	0.00	0.00	0.00	0.00	0.00
GT84	0.00	0.00	0.00	0.00	0.00	0.00	0.00	0.02	0.02	0.01	0.00	0.00	0.02	0.03	0.00	0.00	0.00	0.07	0.00	0.00	0.00
GT15	0.00	0.00	0.00	0.00	0.00	0.00	0.00	0.00	0.00	0.00	0.00	0.00	0.00	0.00	0.00	0.00	0.00	0.00	0.00	0.00	0.00
GH93	0.00	0.00	0.04	0.00	0.00	0.00	0.01	0.00	0.00	0.00	0.01	0.00	0.01	0.00	0.00	0.00	0.00	0.00	0.01	0.00	0.00
PL16	0.00	0.00	0.00	0.00	0.00	0.00	0.00	0.00	0.00	0.00	0.00	0.00	0.00	0.00	0.00	0.00	0.00	0.00	0.00	0.00	0.00
GH26	0.02	0.22	0.02	0.11	0.08	0.06	0.03	0.03	0.00	0.05	0.05	0.00	0.12	0.07	0.00	0.00	0.22	0.09	0.07	0.10	0.04
GT75	0.00	0.00	0.00	0.00	0.00	0.00	0.00	0.00	0.00	0.00	0.00	0.00	0.00	0.00	0.00	0.00	0.00	0.00	0.00	0.00	0.00
GH53	0.02	0.09	0.02	0.06	0.12	0.06	0.04	0.15	0.10	0.02	0.03	0.02	0.03	0.08	0.00	0.06	0.17	0.11	0.07	0.01	0.02
GT88	0.00	0.00	0.00	0.00	0.00	0.00	0.00	0.00	0.00	0.00	0.00	0.00	0.00	0.00	0.00	0.00	0.00	0.00	0.00	0.00	0.00
GT24	0.00	0.00	0.00	0.00	0.00	0.00	0.00	0.00	0.00	0.00	0.00	0.00	0.00	0.00	0.00	0.00	0.00	0.00	0.00	0.00	0.00
GT81	0.00	0.00	0.00	0.00	0.00	0.00	0.00	0.00	0.00	0.00	0.00	0.00	0.00	0.00	0.00	0.00	0.00	0.00	0.00	0.00	0.00
GH4	0.12	0.26	0.14	0.38	0.10	0.17	0.07	0.13	0.08	0.06	0.11	0.12	0.19	0.19	0.50	0.02	0.16	0.07	0.07	0.16	0.30
GT78	0.00	0.00	0.00	0.00	0.00	0.00	0.00	0.00	0.00	0.00	0.00	0.00	0.00	0.00	0.00	0.00	0.00	0.00	0.00	0.00	0.00
GH8	0.02	0.02	0.04	0.06	0.05	0.03	0.01	0.03	0.05	0.02	0.03	0.02	0.02	0.05	0.03	0.02	0.12	0.02	0.01	0.01	0.02
GH70	0.00	0.00	0.00	0.00	0.00	0.00	0.00	0.00	0.00	0.00	0.00	0.00	0.00	0.00	0.00	0.00	0.00	0.00	0.00	0.00	0.00
GH122	0.00	0.00	0.00	0.00	0.00	0.00	0.00	0.00	0.00	0.00	0.00	0.00	0.00	0.00	0.00	0.00	0.00	0.00	0.00	0.00	0.00
GH74	0.00	0.00	0.00	0.00	0.00	0.00	0.00	0.00	0.00	0.00	0.00	0.00	0.02	0.01	0.00	0.00	0.03	0.00	0.00	0.03	0.00
GT41	0.02	0.00	0.00	0.00	0.00	0.02	0.01	0.02	0.00	0.01	0.00	0.00	0.00	0.00	0.00	0.00	0.01	0.00	0.00	0.00	0.00

CAZY_group	CN-AD-22	CN-AD-23	CN-AD-24	CN-AD-25	CN-AD-26	CN-AD-27	CN-AD-28	CN-AD-29	CN-AD-30	CN-AD-31	CN-AD-32	CN-AD-33	CN-AD-34	CN-AD-35	CN-AD-36	CN-AD-37	CN-AD-38	CN-AD-39	CN-AD-40	CN-AD-41	CN-AD-42
**PL1**	0.10	0.28	0.40	0.13	0.34	0.21	0.36	0.20	0.10	0.17	0.27	0.27	0.03	0.30	0.03	0.08	0.07	0.09	0.25	0.06	0.14
**GH15**	0.02	0.05	0.04	0.04	0.03	0.03	0.03	0.05	0.02	0.01	0.01	0.00	0.05	0.04	0.00	0.02	0.03	0.00	0.03	0.04	0.02
**GH32**	0.24	0.62	0.46	0.59	0.31	0.24	0.41	0.33	0.54	0.16	0.27	0.32	0.17	0.44	0.19	0.49	0.37	0.26	0.45	0.36	0.37
**GH44**	0.00	0.00	0.00	0.00	0.00	0.00	0.00	0.00	0.00	0.00	0.00	0.00	0.00	0.00	0.00	0.00	0.00	0.00	0.00	0.00	0.00
**GT6**	0.00	0.00	0.00	0.00	0.00	0.00	0.01	0.00	0.02	0.00	0.00	0.00	0.00	0.00	0.00	0.00	0.00	0.00	0.00	0.00	0.00
**GH13**	2.13	2.25	2.10	3.43	2.15	2.15	2.63	2.58	2.98	2.27	1.12	2.36	1.56	2.22	1.77	2.83	2.48	1.86	1.88	1.95	1.74
**GT80**	0.05	0.00	0.02	0.02	0.01	0.00	0.00	0.00	0.00	0.01	0.00	0.02	0.02	0.01	0.00	0.00	0.01	0.00	0.00	0.01	0.02
**GH71**	0.00	0.00	0.00	0.00	0.00	0.00	0.00	0.00	0.00	0.00	0.00	0.00	0.00	0.00	0.00	0.00	0.00	0.00	0.00	0.00	0.00
**GH63**	0.12	0.08	0.04	0.15	0.10	0.11	0.10	0.10	0.05	0.07	0.03	0.08	0.06	0.07	0.16	0.11	0.09	0.11	0.01	0.04	0.04
**PL21**	0.00	0.05	0.06	0.00	0.05	0.03	0.06	0.05	0.00	0.02	0.07	0.03	0.00	0.03	0.00	0.00	0.00	0.00	0.00	0.06	0.00
**GH65**	0.12	0.19	0.14	0.21	0.16	0.08	0.08	0.07	0.05	0.15	0.05	0.12	0.05	0.07	0.41	0.11	0.20	0.22	0.12	0.10	0.07
**CE4**	0.29	0.40	0.38	0.40	0.38	0.52	0.42	0.43	0.59	0.47	0.22	0.35	0.37	0.51	0.38	0.38	0.54	0.39	0.39	0.41	0.40
**CE14**	0.02	0.03	0.02	0.02	0.02	0.02	0.03	0.00	0.02	0.05	0.00	0.00	0.01	0.00	0.06	0.00	0.01	0.02	0.01	0.00	0.02
**GH115**	0.05	0.37	0.16	0.06	0.27	0.29	0.22	0.17	0.14	0.09	0.24	0.25	0.07	0.19	0.03	0.13	0.15	0.13	0.20	0.00	0.19
**GH104**	0.02	0.00	0.00	0.04	0.00	0.00	0.00	0.00	0.00	0.00	0.00	0.00	0.00	0.00	0.00	0.00	0.00	0.00	0.00	0.00	0.04
**GH111**	0.00	0.00	0.00	0.00	0.00	0.00	0.00	0.00	0.00	0.00	0.00	0.00	0.00	0.00	0.00	0.00	0.00	0.00	0.00	0.00	0.00
**GH95**	0.15	0.69	0.75	0.53	0.72	0.58	0.69	0.56	0.30	0.36	0.49	0.57	0.37	0.65	0.03	0.36	0.29	0.59	0.67	0.28	0.28
**GT70**	0.00	0.00	0.00	0.00	0.00	0.00	0.00	0.00	0.00	0.00	0.00	0.00	0.00	0.00	0.00	0.00	0.00	0.00	0.00	0.00	0.00
**CE10**	0.12	0.25	0.30	0.21	0.20	0.26	0.18	0.20	0.02	0.10	0.22	0.20	0.10	0.08	0.06	0.04	0.13	0.13	0.17	0.18	0.14
**GH10**	0.00	0.09	0.08	0.02	0.14	0.06	0.11	0.12	0.03	0.05	0.07	0.05	0.05	0.05	0.00	0.02	0.05	0.15	0.12	0.04	0.09
**GH130**	0.07	0.40	0.30	0.11	0.25	0.35	0.29	0.17	0.15	0.23	0.28	0.15	0.25	0.32	0.00	0.11	0.17	0.26	0.36	0.09	0.11
**PL20**	0.00	0.00	0.00	0.00	0.00	0.00	0.00	0.00	0.00	0.00	0.00	0.00	0.00	0.00	0.00	0.00	0.00	0.00	0.00	0.00	0.00
**GH69**	0.00	0.00	0.00	0.00	0.00	0.00	0.00	0.00	0.00	0.00	0.00	0.00	0.00	0.00	0.00	0.00	0.00	0.00	0.00	0.00	0.00
**PL2**	0.00	0.02	0.00	0.00	0.00	0.00	0.00	0.00	0.00	0.00	0.00	0.00	0.00	0.00	0.00	0.00	0.00	0.00	0.00	0.00	0.00
**GH49**	0.00	0.00	0.00	0.00	0.00	0.00	0.00	0.00	0.00	0.00	0.00	0.00	0.00	0.00	0.00	0.00	0.00	0.00	0.00	0.00	0.00
**GH25**	0.22	0.26	0.24	0.29	0.27	0.35	0.38	0.35	0.30	0.23	0.22	0.34	0.19	0.23	0.57	0.65	0.26	0.22	0.27	0.29	0.25
**GH59**	0.00	0.00	0.00	0.00	0.00	0.00	0.00	0.00	0.00	0.01	0.00	0.00	0.01	0.03	0.00	0.00	0.01	0.00	0.00	0.04	0.00
**GH35**	0.10	0.40	0.48	0.42	0.35	0.32	0.31	0.30	0.14	0.20	0.54	0.46	0.33	0.43	0.13	0.13	0.21	0.33	0.43	0.18	0.23
**GT39**	0.00	0.00	0.00	0.00	0.00	0.00	0.00	0.02	0.00	0.00	0.00	0.00	0.00	0.01	0.00	0.00	0.00	0.02	0.00	0.00	0.00
**GT57**	0.00	0.00	0.00	0.00	0.00	0.00	0.00	0.00	0.00	0.00	0.00	0.00	0.00	0.00	0.00	0.00	0.00	0.00	0.00	0.00	0.00
**GH129**	0.00	0.00	0.00	0.04	0.00	0.00	0.01	0.00	0.03	0.00	0.00	0.00	0.02	0.00	0.00	0.04	0.00	0.00	0.00	0.00	0.04
**GT22**	0.00	0.00	0.00	0.00	0.00	0.00	0.00	0.00	0.00	0.00	0.00	0.00	0.00	0.00	0.00	0.00	0.00	0.00	0.00	0.00	0.00
**PL12**	0.05	0.11	0.26	0.02	0.16	0.11	0.13	0.12	0.02	0.09	0.12	0.10	0.02	0.11	0.00	0.00	0.04	0.17	0.10	0.06	0.04
**PL18**	0.00	0.00	0.00	0.00	0.00	0.00	0.00	0.00	0.00	0.00	0.00	0.00	0.00	0.00	0.00	0.00	0.00	0.00	0.00	0.00	0.00
**GT92**	0.00	0.00	0.00	0.00	0.00	0.00	0.00	0.00	0.00	0.00	0.00	0.00	0.00	0.00	0.00	0.00	0.00	0.00	0.00	0.00	0.00
**GT44**	0.00	0.00	0.00	0.00	0.00	0.00	0.00	0.00	0.00	0.00	0.00	0.00	0.00	0.00	0.00	0.00	0.00	0.00	0.00	0.01	0.00
**GT29**	0.00	0.00	0.00	0.00	0.00	0.00	0.00	0.00	0.00	0.00	0.00	0.00	0.00	0.00	0.00	0.00	0.00	0.00	0.00	0.00	0.00
**GT13**	0.00	0.00	0.00	0.00	0.00	0.00	0.00	0.00	0.00	0.00	0.00	0.00	0.00	0.00	0.00	0.00	0.00	0.00	0.00	0.00	0.00
**GT52**	0.00	0.00	0.00	0.00	0.00	0.00	0.00	0.00	0.00	0.00	0.00	0.00	0.00	0.00	0.00	0.00	0.00	0.00	0.00	0.00	0.00
**GT64**	0.00	0.00	0.00	0.00	0.00	0.00	0.00	0.00	0.00	0.00	0.00	0.00	0.00	0.00	0.00	0.00	0.00	0.00	0.00	0.00	0.00
**GT85**	0.00	0.00	0.00	0.00	0.00	0.00	0.00	0.00	0.00	0.00	0.00	0.00	0.00	0.00	0.00	0.00	0.00	0.00	0.00	0.00	0.00
**GT16**	0.00	0.00	0.00	0.00	0.00	0.00	0.00	0.00	0.00	0.00	0.00	0.00	0.00	0.00	0.00	0.00	0.00	0.00	0.00	0.00	0.00
**GH56**	0.00	0.00	0.00	0.00	0.00	0.00	0.00	0.00	0.00	0.00	0.00	0.00	0.00	0.00	0.00	0.00	0.00	0.00	0.00	0.00	0.00
**GT60**	0.00	0.00	0.00	0.00	0.00	0.00	0.00	0.00	0.00	0.00	0.00	0.00	0.00	0.00	0.00	0.00	0.00	0.00	0.00	0.00	0.00
**GH41**	0.00	0.00	0.00	0.00	0.00	0.00	0.00	0.00	0.00	0.00	0.00	0.00	0.00	0.00	0.00	0.00	0.00	0.00	0.00	0.00	0.00
**CE7**	0.07	0.14	0.14	0.08	0.11	0.09	0.08	0.05	0.03	0.04	0.20	0.08	0.05	0.13	0.00	0.04	0.07	0.07	0.13	0.03	0.16
**GH39**	0.00	0.08	0.00	0.06	0.00	0.02	0.00	0.03	0.02	0.00	0.01	0.00	0.00	0.05	0.03	0.04	0.04	0.00	0.00	0.03	0.04
**GT30**	0.24	0.29	0.38	0.34	0.28	0.35	0.24	0.30	0.24	0.15	0.24	0.29	0.19	0.28	0.03	0.15	0.16	0.33	0.25	0.15	0.25
**PL15**	0.00	0.12	0.26	0.00	0.18	0.17	0.18	0.05	0.00	0.16	0.19	0.07	0.03	0.11	0.00	0.00	0.01	0.04	0.16	0.01	0.09
**GH102**	0.02	0.08	0.08	0.04	0.00	0.02	0.04	0.02	0.02	0.02	0.03	0.03	0.02	0.01	0.06	0.06	0.07	0.00	0.00	0.08	0.07

CAZY_group	CN-AD-43	CN-AD-44	CN-AD-45	CN-AD-46	CN-AD-47	CN-AD-48	CN-AD-49	CN-AD-50	CN-AD-51	CN-AD-52	CN-AD-53	CN-AD-54	CN-AD-55	CN-AD-56	CN-AD-57	CN-AD-58	CN-AD-59	CN-AD-60	CN-AD-61	CN-AD-62	CN-AD-63
**GH29**	0.59	0.45	0.83	0.55	0.59	0.42	0.79	0.50	0.66	0.26	0.42	0.47	0.69	0.71	0.70	0.78	0.45	0.54	1.16	0.39	0.56
**PL4**	0.00	0.00	0.00	0.00	0.00	0.00	0.00	0.00	0.00	0.00	0.00	0.00	0.00	0.00	0.00	0.00	0.00	0.00	0.00	0.00	0.01
**GH23**	0.94	1.06	1.02	0.78	0.71	0.92	1.14	0.91	1.11	0.90	0.62	0.73	0.66	1.01	1.03	1.32	0.71	0.88	0.82	0.68	0.93
**CE6**	0.00	0.02	0.02	0.09	0.00	0.02	0.04	0.04	0.06	0.06	0.04	0.10	0.02	0.04	0.02	0.05	0.06	0.04	0.07	0.00	0.06
**GH72**	0.09	0.14	0.10	0.07	0.00	0.04	0.09	0.10	0.05	0.20	0.02	0.03	0.00	0.05	0.08	0.05	0.04	0.08	0.09	0.13	0.01
**GH114**	0.00	0.00	0.00	0.00	0.00	0.00	0.00	0.00	0.00	0.00	0.00	0.00	0.00	0.00	0.00	0.00	0.00	0.00	0.00	0.00	0.00
**GH78**	1.11	0.38	0.56	1.05	0.39	0.50	1.18	0.70	1.03	0.38	0.85	0.64	0.75	0.60	0.67	0.90	0.51	0.61	1.13	0.29	0.77
**PL19**	0.00	0.00	0.00	0.00	0.00	0.00	0.00	0.00	0.00	0.00	0.00	0.00	0.00	0.00	0.00	0.00	0.00	0.00	0.00	0.00	0.00
**GT27**	0.00	0.00	0.00	0.00	0.00	0.00	0.00	0.00	0.00	0.00	0.00	0.00	0.00	0.00	0.00	0.00	0.00	0.00	0.00	0.00	0.00
**GH5**	0.09	0.27	0.33	0.28	0.20	0.17	0.26	0.60	0.51	0.17	0.25	0.14	0.14	0.23	0.43	0.24	0.16	0.15	0.34	0.47	0.54
**GT40**	0.00	0.00	0.00	0.00	0.00	0.00	0.00	0.00	0.00	0.00	0.00	0.00	0.00	0.00	0.00	0.00	0.00	0.00	0.00	0.00	0.00
**CE13**	0.00	0.02	0.00	0.02	0.00	0.00	0.00	0.00	0.00	0.00	0.00	0.00	0.00	0.02	0.00	0.00	0.00	0.00	0.00	0.00	0.00
**GT37**	0.00	0.00	0.00	0.00	0.00	0.00	0.00	0.00	0.00	0.00	0.00	0.00	0.00	0.00	0.00	0.00	0.00	0.00	0.00	0.00	0.00
**GH33**	0.22	0.06	0.15	0.30	0.24	0.17	0.28	0.12	0.17	0.03	0.11	0.21	0.22	0.12	0.21	0.28	0.06	0.18	0.24	0.06	0.20
**GT65**	0.00	0.00	0.00	0.00	0.00	0.00	0.00	0.00	0.00	0.00	0.00	0.00	0.00	0.00	0.00	0.00	0.00	0.00	0.00	0.00	0.00
**GH86**	0.00	0.00	0.00	0.00	0.00	0.00	0.00	0.00	0.00	0.00	0.00	0.00	0.00	0.02	0.00	0.00	0.00	0.00	0.00	0.02	0.00
**GH123**	0.17	0.02	0.10	0.05	0.08	0.10	0.24	0.04	0.15	0.00	0.15	0.12	0.10	0.05	0.11	0.24	0.04	0.11	0.14	0.02	0.11
**GH96**	0.00	0.00	0.00	0.00	0.00	0.00	0.00	0.00	0.00	0.00	0.00	0.00	0.00	0.00	0.00	0.00	0.00	0.00	0.00	0.00	0.00
**GH14**	0.00	0.00	0.00	0.00	0.00	0.00	0.00	0.00	0.00	0.00	0.00	0.00	0.00	0.00	0.00	0.00	0.00	0.00	0.00	0.00	0.00
**CE3**	0.00	0.00	0.00	0.00	0.00	0.00	0.00	0.00	0.00	0.00	0.00	0.00	0.00	0.00	0.00	0.00	0.00	0.00	0.00	0.00	0.00
**PL10**	0.04	0.06	0.15	0.21	0.08	0.02	0.15	0.08	0.14	0.06	0.18	0.09	0.08	0.16	0.07	0.17	0.06	0.11	0.20	0.03	0.14
**GT48**	0.00	0.00	0.00	0.00	0.00	0.00	0.00	0.00	0.00	0.00	0.00	0.00	0.00	0.00	0.00	0.00	0.00	0.00	0.00	0.00	0.00
**PL6**	0.00	0.00	0.00	0.00	0.00	0.00	0.02	0.00	0.00	0.00	0.00	0.00	0.00	0.00	0.00	0.00	0.00	0.00	0.02	0.00	0.00
**GT83**	0.13	0.00	0.20	0.14	0.04	0.08	0.15	0.06	0.19	0.09	0.05	0.26	0.09	0.14	0.15	0.12	0.02	0.08	0.10	0.03	0.07
**GH126**	0.00	0.00	0.00	0.00	0.00	0.02	0.00	0.00	0.00	0.00	0.00	0.00	0.00	0.00	0.00	0.00	0.00	0.00	0.00	0.00	0.00
**GH9**	0.00	0.03	0.10	0.25	0.12	0.23	0.11	0.38	0.23	0.03	0.07	0.10	0.09	0.11	0.11	0.07	0.06	0.13	0.32	0.06	0.32
**GH75**	0.00	0.00	0.00	0.00	0.00	0.00	0.00	0.00	0.00	0.00	0.00	0.00	0.00	0.00	0.00	0.00	0.00	0.00	0.00	0.00	0.00
**GT71**	0.00	0.00	0.00	0.00	0.00	0.00	0.00	0.00	0.00	0.00	0.00	0.00	0.00	0.00	0.00	0.00	0.00	0.00	0.00	0.00	0.00
**GH46**	0.00	0.00	0.00	0.00	0.00	0.00	0.00	0.00	0.00	0.00	0.00	0.00	0.00	0.00	0.00	0.00	0.00	0.00	0.00	0.00	0.00
**GT61**	0.00	0.00	0.00	0.00	0.00	0.00	0.00	0.00	0.00	0.00	0.00	0.00	0.00	0.00	0.00	0.00	0.00	0.00	0.00	0.00	0.00
**GT3**	0.04	0.03	0.17	0.07	0.04	0.06	0.17	0.12	0.14	0.03	0.09	0.05	0.13	0.14	0.14	0.21	0.00	0.05	0.12	0.06	0.09
**PL3**	0.00	0.00	0.00	0.00	0.00	0.00	0.00	0.00	0.00	0.00	0.00	0.00	0.00	0.00	0.00	0.00	0.00	0.00	0.00	0.00	0.00
**GH108**	0.00	0.00	0.03	0.05	0.04	0.06	0.06	0.02	0.00	0.00	0.05	0.05	0.01	0.04	0.04	0.00	0.04	0.04	0.03	0.05	0.02
**GT56**	0.02	0.02	0.00	0.02	0.00	0.02	0.00	0.00	0.01	0.03	0.02	0.02	0.00	0.05	0.00	0.00	0.00	0.01	0.00	0.00	0.00
**PL11**	0.28	0.00	0.13	0.09	0.20	0.00	0.17	0.20	0.18	0.03	0.22	0.10	0.13	0.05	0.18	0.19	0.10	0.09	0.26	0.02	0.17
**GH45**	0.00	0.00	0.00	0.00	0.00	0.00	0.00	0.00	0.00	0.00	0.00	0.00	0.00	0.00	0.00	0.00	0.00	0.00	0.00	0.00	0.00
**GT34**	0.00	0.00	0.00	0.00	0.00	0.00	0.00	0.00	0.00	0.00	0.00	0.00	0.00	0.00	0.00	0.00	0.00	0.00	0.00	0.00	0.00
**PL14**	0.00	0.00	0.00	0.00	0.00	0.00	0.00	0.00	0.00	0.00	0.00	0.00	0.00	0.00	0.00	0.00	0.00	0.00	0.00	0.00	0.00
**GH19**	0.00	0.05	0.00	0.00	0.00	0.00	0.00	0.00	0.00	0.00	0.00	0.03	0.00	0.04	0.00	0.00	0.00	0.01	0.00	0.02	0.01
**GH118**	0.00	0.00	0.00	0.00	0.00	0.00	0.00	0.00	0.00	0.00	0.00	0.00	0.00	0.00	0.00	0.00	0.00	0.00	0.00	0.00	0.00
**GH82**	0.00	0.00	0.00	0.00	0.00	0.00	0.00	0.00	0.00	0.00	0.00	0.00	0.00	0.00	0.00	0.00	0.00	0.00	0.00	0.00	0.00
**GH76**	0.33	0.05	0.10	0.28	0.00	0.02	0.30	0.26	0.23	0.00	0.18	0.31	0.16	0.00	0.23	0.07	0.10	0.10	0.22	0.00	0.19
**GT4**	1.68	1.14	1.60	1.65	1.78	1.61	2.03	1.63	1.76	1.21	1.40	1.24	1.28	1.13	1.72	2.26	1.26	1.49	2.30	1.05	1.68
**GT87**	0.00	0.00	0.00	0.00	0.00	0.00	0.00	0.00	0.00	0.00	0.00	0.02	0.00	0.00	0.00	0.00	0.00	0.00	0.00	0.00	0.00
**GH24**	0.11	0.08	0.12	0.23	0.28	0.38	0.21	0.14	0.14	0.17	0.16	0.29	0.13	0.23	0.17	0.17	0.20	0.24	0.22	0.13	0.12
**GH90**	0.00	0.00	0.00	0.00	0.00	0.00	0.00	0.00	0.00	0.00	0.00	0.02	0.00	0.00	0.00	0.00	0.00	0.00	0.00	0.00	0.00
**GH57**	0.09	0.03	0.18	0.05	0.08	0.04	0.09	0.10	0.10	0.00	0.05	0.12	0.07	0.11	0.13	0.17	0.02	0.06	0.09	0.05	0.05
**GH36**	0.59	0.74	0.69	0.87	0.55	0.59	0.94	0.68	0.71	0.75	0.38	0.59	0.60	0.50	0.48	0.47	0.57	0.69	0.68	0.50	0.59
**GT46**	0.00	0.00	0.00	0.00	0.00	0.00	0.00	0.00	0.00	0.00	0.00	0.00	0.00	0.00	0.00	0.00	0.00	0.00	0.00	0.00	0.00
**GH66**	0.04	0.02	0.05	0.05	0.00	0.10	0.02	0.06	0.04	0.00	0.04	0.03	0.01	0.04	0.04	0.05	0.00	0.03	0.03	0.00	0.04
**GH48**	0.00	0.00	0.00	0.00	0.00	0.00	0.00	0.02	0.01	0.00	0.00	0.00	0.00	0.00	0.00	0.00	0.00	0.00	0.00	0.00	0.01
**GT53**	0.00	0.00	0.00	0.00	0.00	0.00	0.00	0.00	0.00	0.00	0.00	0.00	0.00	0.00	0.00	0.00	0.00	0.00	0.00	0.00	0.00
**GH60**	0.00	0.00	0.00	0.00	0.00	0.00	0.00	0.00	0.00	0.00	0.00	0.00	0.00	0.00	0.00	0.00	0.00	0.00	0.00	0.00	0.00

CAZY_group	CN-AD-43	CN-AD-44	CN-AD-45	CN-AD-46	CN-AD-47	CN-AD-48	CN-AD-49	CN-AD-50	CN-AD-51	CN-AD-52	CN-AD-53	CN-AD-54	CN-AD-55	CN-AD-56	CN-AD-57	CN-AD-58	CN-AD-59	CN-AD-60	CN-AD-61	CN-AD-62	CN-AD-63
GT31	0.00	0.00	0.00	0.00	0.00	0.02	0.00	0.00	0.00	0.00	0.00	0.02	0.00	0.00	0.00	0.00	0.00	0.01	0.00	0.00	0.00
GH105	0.76	0.30	0.61	0.71	0.16	0.15	0.56	0.36	0.64	0.06	0.54	0.31	0.47	0.30	0.55	0.90	0.32	0.50	0.87	0.24	0.65
GT23	0.00	0.00	0.00	0.00	0.00	0.00	0.02	0.00	0.00	0.00	0.00	0.00	0.00	0.00	0.00	0.00	0.00	0.01	0.00	0.00	0.00
GH11	0.00	0.00	0.00	0.00	0.00	0.00	0.00	0.00	0.00	0.00	0.00	0.00	0.00	0.00	0.00	0.00	0.00	0.00	0.00	0.00	0.00
GT38	0.00	0.00	0.00	0.05	0.00	0.00	0.00	0.00	0.00	0.00	0.00	0.02	0.00	0.02	0.00	0.00	0.00	0.00	0.00	0.00	0.00
GT43	0.00	0.00	0.00	0.00	0.00	0.00	0.00	0.00	0.00	0.00	0.00	0.00	0.00	0.00	0.00	0.00	0.00	0.00	0.00	0.00	0.00
GH99	0.00	0.00	0.00	0.00	0.00	0.00	0.00	0.02	0.00	0.00	0.02	0.02	0.00	0.00	0.00	0.00	0.00	0.00	0.00	0.00	0.01
GH38	0.07	0.17	0.18	0.30	0.04	0.13	0.24	0.30	0.18	0.12	0.20	0.31	0.20	0.09	0.14	0.28	0.14	0.21	0.27	0.16	0.17
GH109	0.24	0.08	0.26	0.30	0.32	0.19	0.30	0.14	0.33	0.09	0.18	0.10	0.27	0.16	0.18	0.38	0.16	0.31	0.29	0.13	0.20
GH2	3.97	1.94	3.62	3.74	2.88	2.64	4.26	3.04	4.20	1.76	4.12	3.23	3.26	1.98	3.45	3.89	2.82	3.03	5.83	2.40	4.20
GT28	0.28	0.45	0.33	0.32	0.24	0.44	0.36	0.38	0.29	0.26	0.18	0.22	0.24	0.30	0.31	0.24	0.24	0.38	0.32	0.34	0.25
GH42	0.07	0.41	0.25	0.11	0.12	0.25	0.17	0.20	0.08	0.40	0.07	0.21	0.10	0.11	0.13	0.07	0.14	0.14	0.20	0.23	0.15
GT58	0.00	0.00	0.00	0.00	0.00	0.00	0.00	0.00	0.00	0.00	0.00	0.00	0.00	0.00	0.00	0.00	0.00	0.00	0.00	0.00	0.00
GH61	0.00	0.00	0.00	0.00	0.00	0.00	0.00	0.00	0.00	0.00	0.00	0.00	0.00	0.00	0.00	0.00	0.00	0.00	0.00	0.00	0.00
GH80	0.00	0.00	0.00	0.00	0.00	0.00	0.00	0.00	0.00	0.00	0.00	0.00	0.00	0.00	0.00	0.00	0.00	0.00	0.00	0.00	0.00
GH98	0.02	0.00	0.00	0.02	0.08	0.00	0.00	0.00	0.00	0.00	0.00	0.00	0.00	0.00	0.01	0.00	0.00	0.00	0.02	0.03	0.00
GH6	0.00	0.00	0.00	0.00	0.00	0.00	0.00	0.00	0.00	0.00	0.00	0.00	0.00	0.00	0.00	0.00	0.00	0.00	0.00	0.00	0.00
GT1	0.04	0.05	0.00	0.02	0.04	0.04	0.04	0.02	0.04	0.09	0.02	0.05	0.02	0.02	0.07	0.07	0.02	0.01	0.03	0.02	0.05
GH28	0.83	0.36	0.74	0.76	0.43	0.23	1.03	0.60	0.94	0.23	0.93	0.93	0.50	0.48	0.59	1.04	0.75	0.68	1.28	0.35	1.00
GT42	0.00	0.00	0.00	0.00	0.00	0.00	0.00	0.00	0.00	0.00	0.00	0.00	0.00	0.00	0.00	0.00	0.00	0.00	0.00	0.00	0.00
GH128	0.00	0.00	0.00	0.00	0.00	0.00	0.00	0.00	0.00	0.00	0.00	0.00	0.00	0.00	0.00	0.00	0.00	0.00	0.00	0.00	0.00
CE12	0.09	0.05	0.13	0.07	0.08	0.00	0.34	0.12	0.13	0.03	0.18	0.10	0.07	0.07	0.15	0.14	0.04	0.11	0.20	0.10	0.25
CE16	0.00	0.00	0.00	0.00	0.00	0.00	0.00	0.00	0.00	0.00	0.00	0.00	0.00	0.00	0.00	0.00	0.00	0.00	0.00	0.00	0.00
GT89	0.00	0.00	0.00	0.00	0.00	0.00	0.00	0.00	0.00	0.00	0.00	0.00	0.00	0.00	0.00	0.00	0.00	0.00	0.00	0.00	0.00
GT11	0.07	0.03	0.03	0.11	0.08	0.04	0.09	0.04	0.09	0.00	0.00	0.03	0.06	0.11	0.06	0.09	0.04	0.10	0.09	0.05	0.04
GT77	0.02	0.02	0.02	0.00	0.00	0.00	0.00	0.00	0.01	0.00	0.02	0.02	0.01	0.00	0.00	0.00	0.00	0.01	0.00	0.00	0.00
GT55	0.00	0.00	0.00	0.00	0.00	0.00	0.00	0.00	0.00	0.00	0.00	0.00	0.00	0.00	0.00	0.00	0.00	0.00	0.00	0.00	0.00
GT18	0.00	0.00	0.00	0.00	0.00	0.00	0.00	0.00	0.00	0.00	0.00	0.00	0.00	0.00	0.00	0.00	0.00	0.00	0.00	0.00	0.00
GH87	0.00	0.00	0.02	0.00	0.00	0.00	0.00	0.00	0.01	0.00	0.00	0.00	0.00	0.02	0.00	0.00	0.02	0.00	0.00	0.00	0.00
PL5	0.00	0.00	0.00	0.00	0.00	0.00	0.00	0.00	0.00	0.00	0.00	0.00	0.00	0.00	0.00	0.00	0.00	0.00	0.00	0.00	0.00
GH22	0.00	0.00	0.00	0.00	0.00	0.00	0.00	0.00	0.00	0.00	0.00	0.00	0.00	0.00	0.00	0.00	0.00	0.00	0.00	0.00	0.00
CE2	0.04	0.05	0.02	0.05	0.00	0.00	0.02	0.04	0.06	0.03	0.02	0.03	0.02	0.04	0.06	0.02	0.06	0.05	0.03	0.03	0.06
GH120	0.04	0.11	0.02	0.05	0.00	0.08	0.00	0.06	0.01	0.03	0.00	0.05	0.00	0.04	0.06	0.00	0.04	0.04	0.00	0.08	0.05
GH73	0.22	0.42	0.36	0.34	0.39	0.63	0.34	0.30	0.28	0.38	0.31	0.29	0.23	0.39	0.38	0.40	0.26	0.51	0.31	0.21	0.30
GT17	0.00	0.02	0.00	0.00	0.00	0.00	0.00	0.00	0.00	0.00	0.02	0.00	0.00	0.00	0.00	0.00	0.00	0.00	0.00	0.00	0.01
GH124	0.00	0.00	0.00	0.00	0.00	0.00	0.00	0.00	0.00	0.00	0.00	0.00	0.00	0.00	0.00	0.00	0.00	0.00	0.00	0.00	0.00
GH54	0.00	0.00	0.00	0.00	0.00	0.00	0.00	0.00	0.00	0.00	0.00	0.00	0.00	0.00	0.00	0.00	0.00	0.00	0.00	0.00	0.00
GH100	0.00	0.00	0.00	0.00	0.00	0.00	0.00	0.00	0.00	0.00	0.00	0.00	0.00	0.00	0.00	0.00	0.00	0.00	0.00	0.00	0.00
GH113	0.00	0.02	0.00	0.00	0.00	0.02	0.00	0.02	0.01	0.00	0.00	0.00	0.00	0.02	0.01	0.00	0.02	0.05	0.02	0.02	0.02
GT5	0.15	0.34	0.35	0.21	0.32	0.19	0.24	0.26	0.36	0.35	0.18	0.14	0.25	0.34	0.27	0.31	0.32	0.25	0.38	0.39	0.30
GH94	0.02	0.39	0.17	0.09	0.28	0.17	0.09	0.28	0.17	0.20	0.04	0.00	0.09	0.27	0.15	0.05	0.34	0.20	0.36	0.58	0.19
GT66	0.00	0.00	0.00	0.00	0.00	0.00	0.00	0.00	0.00	0.00	0.00	0.00	0.00	0.00	0.00	0.00	0.00	0.00	0.00	0.02	0.00
GT94	0.00	0.00	0.00	0.00	0.00	0.00	0.00	0.00	0.00	0.00	0.00	0.00	0.00	0.00	0.00	0.00	0.00	0.00	0.00	0.00	0.00
GH58	0.00	0.00	0.00	0.00	0.00	0.00	0.00	0.00	0.00	0.00	0.00	0.02	0.00	0.04	0.00	0.00	0.00	0.00	0.00	0.02	0.00
GH1	0.28	0.61	0.25	0.48	0.20	1.07	0.34	0.20	0.22	0.64	0.27	0.29	0.23	0.18	0.29	0.00	0.34	0.20	0.17	0.29	0.15
PL22	0.02	0.02	0.00	0.00	0.00	0.00	0.00	0.00	0.00	0.00	0.02	0.00	0.00	0.00	0.00	0.00	0.00	0.00	0.00	0.00	0.00
GT9	0.11	0.03	0.15	0.14	0.20	0.17	0.17	0.06	0.18	0.14	0.11	0.19	0.14	0.25	0.17	0.07	0.04	0.18	0.22	0.06	0.08
GT76	0.00	0.00	0.00	0.00	0.00	0.00	0.00	0.00	0.00	0.00	0.00	0.00	0.00	0.00	0.00	0.00	0.00	0.00	0.00	0.00	0.00
GT32	0.09	0.03	0.02	0.09	0.04	0.17	0.17	0.00	0.08	0.00	0.09	0.07	0.07	0.05	0.06	0.12	0.08	0.00	0.12	0.02	0.06
GT47	0.00	0.00	0.03	0.02	0.00	0.02	0.04	0.02	0.04	0.09	0.02	0.03	0.03	0.05	0.05	0.05	0.06	0.04	0.02	0.03	0.05
GH84	0.11	0.05	0.15	0.07	0.12	0.10	0.11	0.08	0.08	0.03	0.13	0.10	0.13	0.05	0.05	0.07	0.08	0.11	0.07	0.05	0.12
GH83	0.00	0.00	0.00	0.00	0.00	0.00	0.00	0.00	0.00	0.00	0.00	0.00	0.00	0.00	0.00	0.00	0.00	0.00	0.00	0.00	0.00
GT59	0.00	0.00	0.00	0.00	0.00	0.00	0.00	0.00	0.00	0.00	0.00	0.00	0.00	0.00	0.00	0.00	0.00	0.00	0.00	0.00	0.00

CAZY_group	CN-AD-43	CN-AD-44	CN-AD-45	CN-AD-46	CN-AD-47	CN-AD-48	CN-AD-49	CN-AD-50	CN-AD-51	CN-AD-52	CN-AD-53	CN-AD-54	CN-AD-55	CN-AD-56	CN-AD-57	CN-AD-58	CN-AD-59	CN-AD-60	CN-AD-61	CN-AD-62	CN-AD-63
**GH34**	0.00	0.00	0.00	0.00	0.00	0.00	0.00	0.00	0.00	0.00	0.00	0.00	0.00	0.00	0.00	0.00	0.00	0.00	0.00	0.00	0.00
**GT14**	0.00	0.00	0.02	0.05	0.00	0.04	0.04	0.00	0.04	0.00	0.02	0.02	0.02	0.00	0.01	0.02	0.00	0.03	0.05	0.00	0.04
**GH47**	0.00	0.00	0.00	0.00	0.00	0.00	0.00	0.00	0.00	0.00	0.00	0.00	0.00	0.00	0.00	0.00	0.00	0.00	0.00	0.00	0.00
**GH97**	0.94	0.11	0.88	1.17	0.39	0.50	1.01	0.64	1.04	0.26	1.07	0.76	0.76	0.62	0.67	1.30	0.34	0.59	1.16	0.16	0.97
**GH50**	0.09	0.00	0.08	0.11	0.00	0.00	0.02	0.08	0.10	0.00	0.04	0.07	0.07	0.00	0.07	0.02	0.02	0.00	0.09	0.00	0.02
**GT26**	0.09	0.13	0.17	0.16	0.20	0.15	0.21	0.16	0.11	0.17	0.09	0.12	0.14	0.11	0.20	0.19	0.16	0.20	0.17	0.10	0.14
**GH18**	0.52	0.39	0.51	0.50	0.12	0.42	0.58	0.42	0.47	0.26	0.54	0.67	0.53	0.35	0.51	0.31	0.32	0.43	0.61	0.24	0.41
**GH37**	0.07	0.05	0.00	0.05	0.00	0.04	0.00	0.02	0.04	0.03	0.04	0.03	0.00	0.04	0.00	0.00	0.00	0.03	0.00	0.00	0.00
**PL13**	0.07	0.00	0.03	0.05	0.00	0.00	0.09	0.06	0.04	0.00	0.04	0.07	0.02	0.00	0.07	0.02	0.02	0.00	0.03	0.02	0.05
**GH27**	0.17	0.11	0.23	0.25	0.08	0.04	0.11	0.10	0.27	0.09	0.16	0.21	0.22	0.05	0.19	0.40	0.08	0.19	0.43	0.08	0.34
**GT54**	0.00	0.00	0.00	0.00	0.00	0.00	0.00	0.00	0.00	0.00	0.00	0.00	0.00	0.00	0.00	0.00	0.00	0.00	0.00	0.00	0.00
**GT91**	0.00	0.00	0.00	0.00	0.00	0.00	0.00	0.00	0.00	0.00	0.00	0.00	0.00	0.00	0.00	0.00	0.00	0.00	0.00	0.00	0.00
**GT72**	0.00	0.00	0.00	0.00	0.00	0.00	0.00	0.00	0.00	0.00	0.00	0.00	0.00	0.00	0.00	0.00	0.00	0.00	0.00	0.00	0.00
**GH67**	0.04	0.02	0.03	0.09	0.08	0.02	0.15	0.04	0.06	0.09	0.02	0.03	0.05	0.05	0.06	0.09	0.04	0.03	0.20	0.00	0.11
**GH12**	0.00	0.00	0.00	0.00	0.00	0.00	0.00	0.00	0.00	0.00	0.00	0.00	0.00	0.00	0.00	0.00	0.00	0.00	0.00	0.00	0.00
**GH91**	0.00	0.02	0.07	0.02	0.00	0.00	0.02	0.00	0.04	0.00	0.04	0.03	0.01	0.04	0.01	0.05	0.02	0.01	0.02	0.03	0.00
**GT69**	0.00	0.00	0.00	0.00	0.00	0.00	0.00	0.00	0.00	0.00	0.00	0.00	0.00	0.00	0.00	0.00	0.00	0.00	0.00	0.00	0.00
**GH106**	0.33	0.16	0.18	0.34	0.20	0.08	0.34	0.34	0.56	0.03	0.24	0.24	0.19	0.34	0.27	0.28	0.10	0.24	0.32	0.02	0.27
**GT62**	0.00	0.00	0.00	0.00	0.00	0.00	0.00	0.00	0.00	0.00	0.00	0.00	0.00	0.00	0.00	0.00	0.00	0.00	0.00	0.00	0.00
**GH17**	0.00	0.00	0.00	0.00	0.00	0.00	0.00	0.00	0.00	0.00	0.00	0.00	0.00	0.00	0.00	0.00	0.00	0.00	0.00	0.00	0.00
**GH88**	0.50	0.02	0.36	0.39	0.04	0.27	0.36	0.22	0.47	0.06	0.47	0.35	0.35	0.14	0.39	0.59	0.14	0.25	0.58	0.11	0.34
**CE5**	0.00	0.00	0.00	0.00	0.00	0.00	0.00	0.00	0.00	0.00	0.00	0.00	0.00	0.00	0.00	0.00	0.00	0.00	0.00	0.00	0.00
**GT8**	0.13	0.08	0.08	0.16	0.04	0.23	0.04	0.06	0.06	0.09	0.15	0.10	0.03	0.23	0.10	0.09	0.06	0.05	0.14	0.06	0.09
**GT50**	0.00	0.00	0.00	0.00	0.00	0.00	0.00	0.00	0.00	0.00	0.00	0.00	0.00	0.00	0.00	0.00	0.00	0.00	0.00	0.00	0.00
**GT20**	0.13	0.03	0.12	0.16	0.04	0.15	0.11	0.06	0.11	0.00	0.11	0.10	0.08	0.05	0.05	0.17	0.06	0.10	0.09	0.05	0.05
**PL9**	0.07	0.08	0.08	0.07	0.16	0.00	0.17	0.08	0.06	0.09	0.15	0.07	0.07	0.02	0.06	0.05	0.02	0.04	0.07	0.15	0.13
**GH55**	0.00	0.00	0.02	0.02	0.08	0.02	0.02	0.02	0.03	0.00	0.00	0.03	0.03	0.02	0.04	0.02	0.00	0.03	0.07	0.05	0.01
**GH64**	0.00	0.00	0.00	0.00	0.00	0.00	0.00	0.00	0.00	0.00	0.00	0.00	0.00	0.00	0.00	0.00	0.00	0.00	0.00	0.00	0.00
**GH127**	0.22	0.27	0.15	0.30	0.16	0.17	0.26	0.18	0.39	0.32	0.22	0.29	0.23	0.21	0.21	0.31	0.18	0.24	0.43	0.15	0.30
**GH103**	0.04	0.02	0.00	0.02	0.00	0.02	0.00	0.00	0.01	0.00	0.02	0.02	0.00	0.05	0.01	0.00	0.00	0.03	0.00	0.00	0.00
**GH3**	2.27	1.77	2.59	2.54	1.58	2.09	2.18	2.57	2.14	1.91	2.74	1.73	2.07	1.35	2.22	2.24	1.70	1.95	3.34	1.89	2.41
**GT45**	0.00	0.00	0.00	0.00	0.00	0.00	0.00	0.00	0.00	0.00	0.00	0.00	0.00	0.00	0.00	0.00	0.00	0.00	0.00	0.00	0.00
**GT74**	0.00	0.00	0.00	0.00	0.00	0.00	0.00	0.00	0.00	0.00	0.00	0.00	0.00	0.00	0.00	0.00	0.00	0.00	0.00	0.00	0.00
**GT90**	0.00	0.00	0.02	0.00	0.00	0.00	0.00	0.02	0.00	0.00	0.00	0.00	0.00	0.00	0.00	0.00	0.00	0.00	0.00	0.00	0.00
**GH116**	0.02	0.00	0.07	0.07	0.12	0.10	0.13	0.02	0.05	0.00	0.11	0.07	0.07	0.05	0.10	0.12	0.02	0.09	0.09	0.05	0.06
**GH31**	1.05	0.97	0.91	0.89	0.67	0.90	0.69	0.72	1.02	0.52	0.89	0.76	0.72	0.43	0.80	0.90	0.71	0.96	1.23	0.68	1.03
**GT12**	0.00	0.00	0.00	0.00	0.00	0.00	0.00	0.00	0.00	0.00	0.00	0.00	0.00	0.00	0.00	0.00	0.00	0.00	0.00	0.00	0.00
**CE1**	0.35	0.17	0.36	0.30	0.24	0.27	0.45	0.32	0.33	0.09	0.36	0.40	0.24	0.23	0.29	0.52	0.18	0.24	0.48	0.16	0.30
**GH121**	0.00	0.03	0.02	0.00	0.00	0.00	0.00	0.10	0.00	0.06	0.00	0.00	0.00	0.00	0.00	0.00	0.00	0.00	0.00	0.02	0.00
**GH79**	0.00	0.00	0.00	0.14	0.00	0.00	0.00	0.00	0.00	0.00	0.00	0.00	0.00	0.00	0.00	0.00	0.00	0.00	0.00	0.00	0.01
**GT73**	0.00	0.00	0.00	0.00	0.00	0.00	0.00	0.00	0.00	0.00	0.00	0.00	0.00	0.04	0.00	0.00	0.00	0.00	0.00	0.00	0.00
**GH77**	0.26	0.45	0.51	0.34	0.51	0.29	0.32	0.34	0.39	0.66	0.31	0.22	0.49	0.34	0.45	0.40	0.39	0.34	0.44	0.63	0.44
**PL17**	0.02	0.02	0.00	0.00	0.04	0.00	0.00	0.00	0.00	0.00	0.00	0.00	0.02	0.00	0.00	0.00	0.02	0.00	0.00	0.03	0.04
**PL8**	0.37	0.00	0.40	0.28	0.12	0.04	0.24	0.20	0.43	0.00	0.33	0.31	0.34	0.14	0.33	0.42	0.16	0.21	0.46	0.15	0.40
**CE9**	0.35	0.39	0.31	0.30	0.36	0.31	0.41	0.40	0.24	0.32	0.25	0.43	0.35	0.21	0.37	0.28	0.36	0.34	0.36	0.26	0.22
**CE15**	0.00	0.00	0.03	0.05	0.04	0.04	0.00	0.00	0.01	0.00	0.00	0.02	0.00	0.00	0.05	0.05	0.00	0.01	0.00	0.00	0.01
**GT79**	0.00	0.00	0.00	0.00	0.00	0.00	0.00	0.00	0.00	0.00	0.00	0.00	0.00	0.00	0.00	0.00	0.00	0.00	0.00	0.00	0.00
**GT10**	0.02	0.00	0.00	0.00	0.00	0.02	0.00	0.06	0.00	0.00	0.00	0.00	0.00	0.00	0.01	0.00	0.00	0.00	0.00	0.00	0.02
**GH119**	0.00	0.00	0.00	0.00	0.00	0.00	0.00	0.00	0.00	0.00	0.00	0.00	0.00	0.00	0.00	0.00	0.00	0.00	0.00	0.00	0.00
**GH16**	0.20	0.02	0.21	0.16	0.04	0.15	0.17	0.16	0.18	0.00	0.31	0.21	0.30	0.16	0.24	0.24	0.06	0.19	0.36	0.10	0.17
**GH43**	1.77	0.89	2.00	2.36	0.71	0.98	2.66	1.71	2.45	1.44	2.00	1.57	1.45	1.08	1.64	3.35	1.16	1.44	3.14	0.79	2.56
**GH51**	0.39	0.34	0.51	0.44	0.16	0.50	0.51	0.34	0.59	0.64	0.36	0.40	0.42	0.46	0.34	0.47	0.28	0.43	0.66	0.32	0.54
**GT35**	0.41	0.94	0.71	0.50	0.59	0.50	0.58	0.72	0.45	0.87	0.33	0.47	0.57	0.53	0.77	0.42	0.63	0.54	0.66	0.89	0.65

CAZY_group	CN-AD-43	CN-AD-44	CN-AD-45	CN-AD-46	CN-AD-47	CN-AD-48	CN-AD-49	CN-AD-50	CN-AD-51	CN-AD-52	CN-AD-53	CN-AD-54	CN-AD-55	CN-AD-56	CN-AD-57	CN-AD-58	CN-AD-59	CN-AD-60	CN-AD-61	CN-AD-62	CN-AD-63
**GT25**	0.04	0.00	0.00	0.00	0.00	0.00	0.00	0.00	0.00	0.00	0.00	0.00	0.01	0.00	0.00	0.00	0.00	0.00	0.02	0.00	0.00
**GT2**	2.99	3.08	3.60	3.90	2.80	2.84	3.79	3.48	3.64	2.89	2.69	2.68	2.83	2.99	3.22	3.80	2.51	3.33	4.43	2.56	3.44
**GT51**	0.79	0.74	0.94	0.85	0.55	0.75	0.84	0.56	0.83	0.75	0.74	0.67	0.75	0.69	0.53	0.73	0.43	0.74	0.70	0.68	0.74
**GH107**	0.00	0.00	0.00	0.00	0.00	0.00	0.00	0.00	0.00	0.00	0.00	0.00	0.00	0.00	0.00	0.00	0.00	0.00	0.00	0.00	0.00
**GH7**	0.00	0.00	0.00	0.00	0.00	0.00	0.00	0.00	0.00	0.00	0.00	0.00	0.00	0.00	0.00	0.00	0.00	0.00	0.00	0.00	0.00
**GT7**	0.00	0.00	0.00	0.00	0.00	0.00	0.00	0.00	0.00	0.00	0.00	0.00	0.00	0.00	0.00	0.00	0.00	0.00	0.00	0.00	0.00
**GT36**	0.00	0.00	0.00	0.00	0.00	0.00	0.00	0.00	0.00	0.00	0.00	0.00	0.00	0.00	0.00	0.00	0.00	0.00	0.00	0.00	0.00
**PL7**	0.00	0.00	0.00	0.00	0.00	0.00	0.00	0.00	0.00	0.00	0.00	0.00	0.00	0.00	0.00	0.00	0.00	0.00	0.00	0.00	0.00
**GH110**	0.20	0.03	0.13	0.02	0.12	0.17	0.24	0.02	0.10	0.03	0.07	0.09	0.14	0.12	0.11	0.24	0.04	0.11	0.22	0.08	0.12
**GT21**	0.00	0.00	0.00	0.00	0.00	0.00	0.00	0.00	0.00	0.00	0.00	0.00	0.00	0.00	0.00	0.00	0.00	0.00	0.00	0.00	0.00
**GH62**	0.00	0.00	0.00	0.00	0.00	0.00	0.00	0.00	0.00	0.00	0.00	0.00	0.00	0.00	0.00	0.00	0.00	0.00	0.00	0.00	0.00
**GH125**	0.17	0.03	0.15	0.21	0.00	0.17	0.19	0.12	0.15	0.00	0.15	0.16	0.19	0.04	0.17	0.07	0.12	0.18	0.15	0.05	0.15
**GH117**	0.07	0.00	0.13	0.11	0.08	0.13	0.11	0.10	0.10	0.00	0.13	0.09	0.08	0.02	0.07	0.07	0.06	0.06	0.10	0.03	0.08
**GH68**	0.00	0.02	0.00	0.00	0.00	0.04	0.00	0.00	0.00	0.00	0.00	0.00	0.00	0.00	0.00	0.00	0.00	0.00	0.00	0.00	0.00
**GH85**	0.00	0.00	0.05	0.00	0.00	0.00	0.00	0.02	0.00	0.00	0.00	0.00	0.01	0.00	0.00	0.00	0.08	0.04	0.00	0.00	0.00
**GH92**	1.83	0.30	1.47	2.27	0.71	0.90	2.08	1.19	1.97	0.26	1.67	1.48	1.73	0.41	1.49	1.79	0.73	1.04	2.44	0.37	1.62
**GH40**	0.00	0.00	0.00	0.00	0.00	0.00	0.00	0.00	0.00	0.00	0.00	0.00	0.00	0.00	0.00	0.00	0.00	0.00	0.00	0.00	0.00
**GT49**	0.00	0.00	0.00	0.00	0.00	0.00	0.00	0.00	0.00	0.00	0.00	0.00	0.00	0.00	0.00	0.00	0.00	0.00	0.00	0.00	0.00
**GH112**	0.07	0.23	0.20	0.02	0.08	0.08	0.04	0.12	0.04	0.35	0.04	0.10	0.08	0.11	0.06	0.02	0.32	0.09	0.17	0.15	0.08
**GT67**	0.00	0.00	0.00	0.00	0.00	0.00	0.00	0.00	0.00	0.00	0.00	0.00	0.00	0.00	0.00	0.00	0.00	0.00	0.00	0.00	0.00
**CE8**	0.13	0.16	0.15	0.21	0.32	0.04	0.32	0.14	0.20	0.09	0.31	0.16	0.14	0.18	0.20	0.19	0.16	0.23	0.26	0.15	0.19
**GT86**	0.00	0.00	0.00	0.00	0.00	0.00	0.00	0.00	0.00	0.00	0.00	0.00	0.00	0.00	0.00	0.00	0.00	0.00	0.00	0.00	0.00
**GH101**	0.04	0.03	0.05	0.00	0.00	0.00	0.02	0.02	0.00	0.14	0.00	0.00	0.00	0.00	0.00	0.00	0.04	0.01	0.00	0.00	0.00
**GH81**	0.00	0.00	0.00	0.00	0.00	0.00	0.04	0.00	0.00	0.00	0.00	0.00	0.00	0.02	0.02	0.00	0.00	0.00	0.00	0.00	0.00
**GT93**	0.00	0.00	0.00	0.00	0.00	0.00	0.00	0.00	0.00	0.00	0.00	0.00	0.00	0.00	0.00	0.00	0.00	0.00	0.00	0.00	0.00
**GT63**	0.00	0.00	0.00	0.00	0.00	0.00	0.00	0.00	0.00	0.00	0.00	0.00	0.00	0.00	0.00	0.00	0.00	0.00	0.00	0.00	0.00
**CE11**	0.13	0.09	0.21	0.11	0.08	0.13	0.24	0.14	0.29	0.12	0.15	0.16	0.17	0.32	0.23	0.14	0.08	0.23	0.19	0.13	0.18
**GT19**	0.15	0.08	0.15	0.18	0.12	0.10	0.15	0.06	0.14	0.14	0.09	0.12	0.15	0.16	0.07	0.12	0.04	0.13	0.19	0.03	0.09
**GT33**	0.00	0.00	0.00	0.00	0.00	0.00	0.00	0.00	0.00	0.00	0.00	0.00	0.00	0.00	0.00	0.00	0.00	0.00	0.00	0.00	0.00
**GH21**	0.00	0.00	0.00	0.00	0.00	0.00	0.00	0.00	0.00	0.00	0.00	0.00	0.00	0.00	0.00	0.00	0.00	0.00	0.00	0.00	0.00
**GT68**	0.00	0.00	0.00	0.00	0.00	0.00	0.00	0.00	0.00	0.00	0.00	0.00	0.00	0.00	0.00	0.00	0.00	0.00	0.00	0.00	0.00
**GH89**	0.41	0.05	0.25	0.30	0.20	0.06	0.21	0.22	0.25	0.17	0.34	0.24	0.24	0.09	0.24	0.40	0.16	0.15	0.39	0.10	0.21
**GT82**	0.00	0.00	0.00	0.00	0.00	0.00	0.00	0.00	0.00	0.00	0.00	0.00	0.00	0.00	0.00	0.00	0.00	0.00	0.02	0.00	0.00
**GH30**	0.33	0.05	0.13	0.39	0.12	0.06	0.24	0.16	0.25	0.03	0.16	0.26	0.14	0.14	0.23	0.26	0.16	0.18	0.44	0.06	0.33
**GH20**	1.29	0.41	1.21	1.10	1.14	0.65	1.31	0.70	1.30	0.29	0.98	1.12	1.18	0.51	1.16	1.25	0.45	0.84	1.40	0.31	0.87
**GH52**	0.00	0.00	0.00	0.00	0.00	0.00	0.00	0.00	0.00	0.00	0.00	0.00	0.00	0.00	0.00	0.00	0.00	0.00	0.00	0.00	0.00
**GT84**	0.00	0.00	0.00	0.00	0.00	0.02	0.00	0.00	0.00	0.00	0.00	0.00	0.00	0.00	0.01	0.05	0.00	0.00	0.00	0.00	0.00
**GT15**	0.00	0.00	0.00	0.00	0.00	0.00	0.00	0.00	0.00	0.00	0.00	0.00	0.00	0.00	0.00	0.00	0.00	0.00	0.00	0.00	0.00
**GH93**	0.02	0.00	0.00	0.00	0.00	0.00	0.02	0.00	0.00	0.00	0.02	0.02	0.01	0.00	0.00	0.00	0.00	0.01	0.03	0.00	0.01
**PL16**	0.00	0.00	0.00	0.00	0.00	0.00	0.00	0.00	0.00	0.00	0.00	0.00	0.00	0.00	0.00	0.00	0.00	0.00	0.00	0.00	0.00
**GH26**	0.02	0.03	0.07	0.14	0.00	0.04	0.02	0.20	0.18	0.03	0.07	0.00	0.02	0.07	0.11	0.07	0.04	0.05	0.14	0.02	0.20
**GT75**	0.00	0.00	0.00	0.00	0.00	0.00	0.00	0.00	0.00	0.00	0.00	0.00	0.00	0.00	0.00	0.00	0.00	0.00	0.00	0.00	0.00
**GH53**	0.00	0.06	0.08	0.05	0.08	0.02	0.06	0.02	0.06	0.06	0.05	0.00	0.01	0.11	0.01	0.07	0.12	0.16	0.22	0.08	0.11
**GT88**	0.00	0.00	0.00	0.00	0.00	0.00	0.00	0.00	0.00	0.00	0.00	0.00	0.00	0.00	0.00	0.00	0.00	0.00	0.00	0.00	0.00
**GT24**	0.00	0.00	0.00	0.00	0.00	0.00	0.00	0.00	0.00	0.00	0.00	0.00	0.00	0.00	0.00	0.00	0.00	0.00	0.00	0.00	0.00
**GT81**	0.00	0.00	0.00	0.00	0.00	0.00	0.00	0.00	0.00	0.00	0.00	0.00	0.00	0.00	0.00	0.00	0.00	0.00	0.00	0.00	0.00
**GH4**	0.24	0.27	0.02	0.18	0.00	0.29	0.09	0.04	0.09	0.38	0.20	0.16	0.17	0.07	0.11	0.05	0.08	0.19	0.10	0.19	0.07
**GT78**	0.00	0.00	0.00	0.00	0.00	0.00	0.00	0.00	0.00	0.00	0.00	0.00	0.00	0.00	0.00	0.00	0.00	0.00	0.00	0.00	0.00
**GH8**	0.02	0.02	0.02	0.09	0.00	0.00	0.00	0.04	0.08	0.03	0.05	0.00	0.00	0.02	0.04	0.09	0.06	0.06	0.10	0.02	0.07
**GH70**	0.00	0.00	0.00	0.00	0.00	0.10	0.00	0.00	0.01	0.00	0.00	0.00	0.00	0.00	0.00	0.00	0.00	0.00	0.00	0.00	0.00
**GH122**	0.00	0.00	0.00	0.00	0.00	0.00	0.00	0.00	0.00	0.00	0.00	0.00	0.00	0.00	0.00	0.00	0.00	0.00	0.00	0.00	0.00
**GH74**	0.00	0.00	0.02	0.00	0.00	0.04	0.00	0.04	0.03	0.03	0.00	0.00	0.00	0.00	0.02	0.00	0.02	0.03	0.02	0.02	0.07
**GT41**	0.00	0.03	0.00	0.00	0.04	0.00	0.00	0.00	0.00	0.00	0.00	0.00	0.00	0.00	0.00	0.00	0.00	0.01	0.00	0.00	0.00

CAZY_group	CN-AD-43	CN-AD-44	CN-AD-45	CN-AD-46	CN-AD-47	CN-AD-48	CN-AD-49	CN-AD-50	CN-AD-51	CN-AD-52	CN-AD-53	CN-AD-54	CN-AD-55	CN-AD-56	CN-AD-57	CN-AD-58	CN-AD-59	CN-AD-60	CN-AD-61	CN-AD-62	CN-AD-63
PL1	0.41	0.14	0.25	0.28	0.20	0.00	0.41	0.32	0.34	0.00	0.42	0.22	0.30	0.16	0.21	0.33	0.16	0.16	0.48	0.13	0.35
GH15	0.02	0.02	0.03	0.05	0.00	0.06	0.06	0.00	0.04	0.00	0.05	0.02	0.07	0.00	0.08	0.09	0.02	0.01	0.03	0.03	0.04
GH32	0.46	0.58	0.48	0.41	0.36	0.65	0.49	0.38	0.32	0.72	0.27	0.43	0.24	0.39	0.29	0.28	0.30	0.31	0.55	0.32	0.30
GH44	0.00	0.00	0.00	0.00	0.00	0.00	0.00	0.04	0.00	0.00	0.00	0.00	0.00	0.00	0.01	0.00	0.00	0.00	0.00	0.03	0.01
GT6	0.02	0.00	0.00	0.02	0.00	0.00	0.00	0.00	0.00	0.00	0.00	0.00	0.00	0.00	0.00	0.00	0.04	0.00	0.00	0.00	0.00
GH13	1.24	3.49	2.43	1.74	2.45	1.84	1.61	3.06	1.69	3.61	1.47	1.48	1.61	2.23	2.26	2.17	1.97	2.06	2.32	2.72	2.06
GT80	0.02	0.02	0.03	0.02	0.00	0.00	0.00	0.00	0.03	0.00	0.02	0.02	0.01	0.04	0.02	0.05	0.00	0.01	0.00	0.00	0.00
GH71	0.00	0.00	0.00	0.00	0.00	0.00	0.00	0.00	0.00	0.00	0.00	0.00	0.00	0.00	0.00	0.00	0.00	0.00	0.00	0.00	0.00
GH63	0.11	0.02	0.02	0.16	0.08	0.10	0.11	0.00	0.09	0.09	0.09	0.02	0.07	0.09	0.04	0.17	0.02	0.11	0.14	0.00	0.07
PL21	0.00	0.00	0.05	0.00	0.08	0.00	0.00	0.06	0.00	0.00	0.04	0.03	0.02	0.00	0.02	0.00	0.06	0.03	0.00	0.03	0.02
GH65	0.07	0.11	0.12	0.16	0.04	0.15	0.06	0.16	0.10	0.12	0.13	0.09	0.08	0.04	0.10	0.14	0.16	0.13	0.20	0.03	0.09
CE4	0.20	0.70	0.38	0.46	0.20	0.48	0.45	0.30	0.42	0.64	0.33	0.41	0.34	0.53	0.43	0.50	0.39	0.50	0.46	0.34	0.50
CE14	0.02	0.00	0.00	0.00	0.00	0.00	0.04	0.00	0.04	0.00	0.00	0.00	0.03	0.02	0.00	0.00	0.02	0.03	0.03	0.00	0.00
GH115	0.20	0.05	0.18	0.37	0.12	0.06	0.34	0.08	0.39	0.14	0.20	0.19	0.19	0.12	0.26	0.35	0.16	0.09	0.55	0.08	0.35
GH104	0.00	0.00	0.00	0.00	0.00	0.00	0.00	0.00	0.00	0.00	0.00	0.05	0.00	0.00	0.00	0.00	0.00	0.00	0.00	0.00	0.00
GH111	0.00	0.00	0.00	0.00	0.00	0.00	0.00	0.00	0.00	0.00	0.00	0.00	0.00	0.00	0.00	0.00	0.00	0.00	0.00	0.00	0.00
GH95	0.63	0.28	0.76	0.50	0.36	0.33	0.66	0.42	0.69	0.26	0.53	0.55	0.49	0.48	0.53	0.61	0.32	0.36	1.02	0.32	0.78
GT70	0.00	0.00	0.00	0.00	0.00	0.00	0.00	0.00	0.00	0.00	0.00	0.00	0.00	0.00	0.00	0.00	0.00	0.00	0.00	0.00	0.00
CE10	0.35	0.17	0.23	0.28	0.12	0.08	0.26	0.12	0.15	0.03	0.18	0.16	0.18	0.14	0.08	0.21	0.10	0.13	0.19	0.06	0.09
GH10	0.04	0.02	0.12	0.14	0.00	0.02	0.09	0.06	0.19	0.12	0.15	0.07	0.02	0.05	0.10	0.17	0.06	0.05	0.10	0.05	0.09
GH130	0.39	0.16	0.43	0.32	0.08	0.10	0.19	0.16	0.37	0.06	0.15	0.21	0.24	0.16	0.32	0.19	0.26	0.25	0.39	0.06	0.27
PL20	0.00	0.00	0.00	0.00	0.00	0.00	0.00	0.00	0.00	0.00	0.00	0.00	0.00	0.00	0.00	0.00	0.00	0.00	0.00	0.00	0.00
GH69	0.00	0.00	0.00	0.00	0.00	0.00	0.00	0.00	0.00	0.00	0.00	0.00	0.00	0.00	0.00	0.00	0.00	0.00	0.00	0.00	0.00
PL2	0.00	0.00	0.00	0.00	0.00	0.00	0.00	0.00	0.00	0.00	0.00	0.00	0.00	0.00	0.00	0.00	0.00	0.00	0.02	0.00	0.00
GH49	0.00	0.00	0.00	0.00	0.00	0.00	0.00	0.00	0.00	0.00	0.00	0.00	0.00	0.00	0.00	0.00	0.00	0.00	0.00	0.00	0.00
GH25	0.22	0.44	0.30	0.30	0.36	0.42	0.32	0.34	0.18	0.66	0.29	0.14	0.22	0.25	0.30	0.12	0.24	0.35	0.20	0.32	0.21
GH59	0.00	0.00	0.00	0.00	0.00	0.00	0.00	0.02	0.01	0.00	0.00	0.00	0.00	0.00	0.02	0.00	0.00	0.00	0.00	0.03	0.01
GH35	0.50	0.13	0.38	0.32	0.32	0.27	0.45	0.24	0.45	0.14	0.45	0.31	0.34	0.14	0.24	0.31	0.24	0.30	0.44	0.16	0.50
GT39	0.00	0.00	0.00	0.00	0.00	0.00	0.00	0.00	0.00	0.00	0.00	0.00	0.00	0.00	0.00	0.00	0.00	0.00	0.00	0.00	0.00
GT57	0.00	0.00	0.00	0.00	0.00	0.00	0.00	0.00	0.00	0.00	0.00	0.00	0.00	0.00	0.00	0.00	0.00	0.00	0.00	0.00	0.00
GH129	0.00	0.06	0.00	0.00	0.00	0.04	0.00	0.04	0.00	0.00	0.00	0.00	0.00	0.00	0.01	0.00	0.00	0.00	0.02	0.02	0.00
GT22	0.00	0.00	0.00	0.00	0.00	0.00	0.00	0.00	0.00	0.00	0.00	0.00	0.00	0.00	0.00	0.00	0.00	0.00	0.00	0.00	0.00
PL12	0.04	0.00	0.10	0.07	0.08	0.02	0.09	0.16	0.13	0.00	0.04	0.19	0.07	0.00	0.14	0.12	0.06	0.04	0.17	0.03	0.08
PL18	0.00	0.00	0.00	0.00	0.00	0.00	0.00	0.00	0.00	0.00	0.00	0.00	0.00	0.00	0.00	0.00	0.00	0.00	0.00	0.00	0.00
GT92	0.00	0.00	0.00	0.00	0.00	0.00	0.00	0.00	0.00	0.00	0.00	0.00	0.00	0.00	0.00	0.00	0.00	0.00	0.00	0.00	0.00
GT44	0.00	0.00	0.00	0.00	0.00	0.00	0.00	0.00	0.00	0.00	0.00	0.00	0.00	0.00	0.00	0.00	0.00	0.00	0.00	0.00	0.00
GT29	0.00	0.00	0.00	0.00	0.00	0.00	0.00	0.00	0.00	0.00	0.00	0.00	0.00	0.00	0.00	0.00	0.00	0.00	0.00	0.00	0.00
GT13	0.00	0.00	0.00	0.00	0.00	0.00	0.00	0.00	0.00	0.00	0.00	0.00	0.00	0.00	0.00	0.00	0.00	0.00	0.00	0.00	0.00
GT52	0.00	0.00	0.00	0.00	0.00	0.00	0.00	0.00	0.00	0.00	0.00	0.00	0.00	0.00	0.00	0.00	0.00	0.00	0.00	0.00	0.00
GT64	0.00	0.00	0.00	0.00	0.00	0.00	0.00	0.00	0.00	0.00	0.00	0.00	0.00	0.00	0.00	0.00	0.00	0.00	0.00	0.00	0.00
GT85	0.00	0.00	0.00	0.00	0.00	0.00	0.00	0.00	0.00	0.00	0.00	0.00	0.00	0.00	0.00	0.00	0.00	0.00	0.00	0.00	0.00
GT16	0.00	0.00	0.00	0.00	0.00	0.00	0.00	0.00	0.00	0.00	0.00	0.00	0.00	0.00	0.00	0.00	0.00	0.00	0.00	0.00	0.00
GH56	0.00	0.00	0.00	0.00	0.00	0.00	0.00	0.00	0.00	0.00	0.00	0.00	0.00	0.00	0.00	0.00	0.00	0.00	0.00	0.00	0.00
GT60	0.00	0.00	0.00	0.00	0.00	0.00	0.00	0.00	0.00	0.00	0.00	0.00	0.00	0.00	0.00	0.00	0.00	0.00	0.00	0.00	0.00
GH41	0.00	0.00	0.00	0.00	0.00	0.00	0.00	0.00	0.00	0.00	0.00	0.00	0.00	0.00	0.00	0.00	0.00	0.00	0.00	0.00	0.00
CE7	0.13	0.03	0.10	0.23	0.12	0.08	0.11	0.10	0.11	0.03	0.13	0.19	0.08	0.05	0.07	0.05	0.10	0.05	0.17	0.02	0.11
GH39	0.02	0.06	0.03	0.00	0.00	0.00	0.02	0.00	0.08	0.00	0.02	0.02	0.00	0.09	0.02	0.02	0.06	0.03	0.05	0.02	0.05
GT30	0.24	0.08	0.28	0.25	0.28	0.17	0.41	0.18	0.33	0.23	0.27	0.43	0.28	0.30	0.25	0.38	0.22	0.24	0.34	0.13	0.22
PL15	0.09	0.00	0.12	0.16	0.08	0.00	0.13	0.18	0.05	0.00	0.11	0.24	0.10	0.00	0.06	0.00	0.06	0.04	0.15	0.03	0.11
GH102	0.02	0.06	0.02	0.05	0.04	0.04	0.00	0.02	0.03	0.09	0.02	0.03	0.01	0.05	0.04	0.00	0.04	0.04	0.02	0.08	0.01

CAZY_group	CN-AD-64	CN-AD-65	CN-AD-66
GH29	0.87	0.67	0.41
PL4	0.00	0.00	0.00
GH23	0.90	1.13	0.71
CE6	0.00	0.04	0.01
GH72	0.03	0.08	0.06
GH114	0.00	0.00	0.00
GH78	0.79	0.83	0.72
PL19	0.00	0.00	0.00
GT27	0.00	0.00	0.00
GH5	0.21	0.16	0.13
GT40	0.00	0.00	0.00
CE13	0.00	0.00	0.00
GT37	0.00	0.00	0.00
GH33	0.24	0.18	0.18
GT65	0.00	0.00	0.00
GH86	0.00	0.00	0.00
GH123	0.08	0.08	0.09
GH96	0.00	0.00	0.00
GH14	0.00	0.00	0.00
CE3	0.00	0.00	0.00
PL10	0.11	0.14	0.07
GT48	0.00	0.00	0.00
PL6	0.00	0.00	0.00
GT83	0.05	0.22	0.08
GH126	0.00	0.00	0.00
GH9	0.05	0.18	0.08
GH75	0.00	0.00	0.00
GT71	0.00	0.00	0.00
GH46	0.00	0.00	0.00
GT61	0.00	0.00	0.00
GT3	0.08	0.24	0.04
PL3	0.00	0.00	0.00
GH108	0.03	0.08	0.04
GT56	0.00	0.00	0.00
PL11	0.08	0.16	0.09
GH45	0.00	0.00	0.00
GT34	0.00	0.00	0.00
PL14	0.00	0.00	0.00
GH19	0.00	0.00	0.00
GH118	0.00	0.00	0.00
GH82	0.00	0.00	0.00
GH76	0.13	0.38	0.04
GT4	1.48	2.00	1.31
GT87	0.00	0.00	0.00
GH24	0.19	0.16	0.12
GH90	0.00	0.00	0.00
GH57	0.11	0.16	0.05
GH36	0.82	0.89	0.47
GT46	0.00	0.00	0.00
GH66	0.05	0.08	0.04
GH48	0.00	0.00	0.00
GT53	0.00	0.00	0.00
GH60	0.00	0.00	0.00

CAZY_group	CN-AD-64	CN-AD-65	CN-AD-66
GT31	0.00	0.00	0.00
GH105	0.63	0.61	0.28
GT23	0.03	0.00	0.00
GH11	0.00	0.00	0.00
GT38	0.00	0.00	0.00
GT43	0.00	0.00	0.00
GH99	0.00	0.02	0.00
GH38	0.21	0.24	0.13
GH109	0.26	0.40	0.14
GH2	3.49	4.28	2.30
GT28	0.40	0.26	0.28
GH42	0.11	0.14	0.09
GT58	0.00	0.00	0.00
GH61	0.00	0.00	0.00
GH80	0.00	0.00	0.00
GH98	0.00	0.02	0.00
GH6	0.00	0.00	0.00
GT1	0.03	0.02	0.08
GH28	0.69	0.93	0.42
GT42	0.00	0.00	0.00
GH128	0.00	0.00	0.00
CE12	0.13	0.06	0.08
CE16	0.00	0.00	0.00
GT89	0.00	0.00	0.00
GT11	0.08	0.04	0.02
GT77	0.00	0.00	0.01
GT55	0.00	0.00	0.00
GT18	0.00	0.00	0.00
GH87	0.00	0.00	0.00
PL5	0.00	0.00	0.00
GH22	0.00	0.00	0.00
CE2	0.00	0.10	0.01
GH120	0.00	0.00	0.01
GH73	0.40	0.48	0.36
GT17	0.00	0.00	0.00
GH124	0.00	0.00	0.00
GH54	0.00	0.00	0.00
GH100	0.00	0.00	0.00
GH113	0.03	0.00	0.01
GT5	0.32	0.36	0.25
GH94	0.32	0.12	0.11
GT66	0.00	0.00	0.00
GT94	0.00	0.00	0.00
GH58	0.00	0.00	0.00
GH1	0.29	0.02	0.33
PL22	0.00	0.00	0.00
GT9	0.19	0.24	0.13
GT76	0.00	0.00	0.00
GT32	0.13	0.00	0.07
GT47	0.03	0.00	0.00
GH84	0.11	0.12	0.09
GH83	0.00	0.00	0.00
GT59	0.00	0.00	0.00

CAZY_group	CN-AD-64	CN-AD-65	CN-AD-66
GH34	0.00	0.00	0.00
GT14	0.03	0.06	0.01
GH47	0.00	0.00	0.00
GH97	0.66	1.37	0.43
GH50	0.00	0.10	0.00
GT26	0.16	0.10	0.15
GH18	0.61	0.75	0.30
GH37	0.00	0.00	0.00
PL13	0.03	0.10	0.01
GH27	0.16	0.30	0.13
GT54	0.00	0.00	0.00
GT91	0.00	0.00	0.00
GT72	0.00	0.00	0.00
GH67	0.03	0.04	0.04
GH12	0.00	0.00	0.00
GH91	0.00	0.04	0.01
GT69	0.00	0.00	0.00
GH106	0.26	0.32	0.11
GT62	0.00	0.00	0.00
GH17	0.00	0.00	0.00
GH88	0.08	0.48	0.18
CE5	0.00	0.00	0.00
GT8	0.19	0.04	0.07
GT50	0.00	0.00	0.00
GT20	0.08	0.08	0.07
PL9	0.13	0.04	0.05
GH55	0.03	0.04	0.00
GH64	0.00	0.00	0.00
GH127	0.19	0.36	0.15
GH103	0.00	0.00	0.00
GH3	1.93	2.52	1.46
GT45	0.00	0.00	0.00
GT74	0.00	0.00	0.00
GT90	0.00	0.00	0.00
GH116	0.08	0.12	0.02
GH31	1.14	0.99	0.70
GT12	0.00	0.00	0.00
CE1	0.29	0.32	0.25
GH121	0.00	0.00	0.00
GH79	0.00	0.00	0.01
GT73	0.00	0.00	0.00
GH77	0.56	0.36	0.37
PL17	0.05	0.02	0.01
PL8	0.26	0.54	0.12
CE9	0.16	0.34	0.32
CE15	0.00	0.02	0.00
GT79	0.00	0.00	0.00
GT10	0.00	0.02	0.01
GH119	0.00	0.00	0.00
GH16	0.11	0.26	0.11
GH43	1.75	2.40	1.02
GH51	0.32	0.59	0.22
GT35	0.71	0.52	0.64

CAZY_group	CN-AD-64	CN-AD-65	CN-AD-66
GT25	0.00	0.04	0.00
GT2	3.31	3.69	2.84
GT51	0.56	0.87	0.75
GH107	0.00	0.00	0.00
GH7	0.00	0.00	0.00
GT7	0.00	0.00	0.00
GT36	0.00	0.00	0.00
PL7	0.00	0.00	0.01
GH110	0.16	0.16	0.09
GT21	0.00	0.00	0.00
GH62	0.00	0.00	0.00
GH125	0.11	0.22	0.07
GH117	0.16	0.18	0.08
GH68	0.00	0.00	0.02
GH85	0.00	0.02	0.02
GH92	1.32	2.46	0.81
GH40	0.00	0.00	0.00
GT49	0.00	0.00	0.00
GH112	0.11	0.04	0.14
GT67	0.00	0.00	0.00
CE8	0.16	0.18	0.12
GT86	0.00	0.00	0.00
GH101	0.00	0.00	0.02
GH81	0.00	0.00	0.00
GT93	0.00	0.00	0.00
GT63	0.00	0.00	0.00
CE11	0.16	0.22	0.14
GT19	0.08	0.10	0.09
GT33	0.00	0.00	0.00
GH21	0.00	0.00	0.00
GT68	0.00	0.00	0.00
GH89	0.26	0.40	0.08
GT82	0.00	0.00	0.00
GH30	0.21	0.20	0.21
GH20	1.06	2.16	0.69
GH52	0.00	0.00	0.00
GT84	0.00	0.00	0.00
GT15	0.00	0.00	0.00
GH93	0.00	0.02	0.00
PL16	0.00	0.00	0.00
GH26	0.08	0.02	0.02
GT75	0.00	0.00	0.00
GH53	0.19	0.12	0.02
GT88	0.00	0.00	0.00
GT24	0.00	0.00	0.00
GT81	0.00	0.00	0.00
GH4	0.11	0.04	0.07
GT78	0.00	0.00	0.00
GH8	0.05	0.08	0.01
GH70	0.00	0.00	0.15
GH122	0.00	0.00	0.00
GH74	0.05	0.00	0.00
GT41	0.00	0.00	0.00

CAZY_group	CN-AD-64	CN-AD-65	CN-AD-66
**PL1**	0.26	0.36	0.07
**GH15**	0.00	0.04	0.04
**GH32**	0.42	0.38	0.32
**GH44**	0.00	0.00	0.00
**GT6**	0.00	0.00	0.00
**GH13**	2.80	2.02	1.59
**GT80**	0.00	0.00	0.00
**GH71**	0.00	0.00	0.00
**GH63**	0.05	0.08	0.05
**PL21**	0.00	0.04	0.02
**GH65**	0.13	0.12	0.11
**CE4**	0.45	0.32	0.34
**CE14**	0.00	0.00	0.00
**GH115**	0.24	0.14	0.12
**GH104**	0.00	0.00	0.00
**GH111**	0.00	0.00	0.00
**GH95**	0.48	0.71	0.29
**GT70**	0.00	0.00	0.00
**CE10**	0.13	0.18	0.14
**GH10**	0.24	0.12	0.07
**GH130**	0.26	0.30	0.11
**PL20**	0.00	0.00	0.00
**GH69**	0.00	0.00	0.00
**PL2**	0.00	0.00	0.00
**GH49**	0.00	0.00	0.00
**GH25**	0.40	0.20	0.35
**GH59**	0.00	0.00	0.00
**GH35**	0.29	0.38	0.27
**GT39**	0.00	0.00	0.00
**GT57**	0.00	0.00	0.00
**GH129**	0.00	0.00	0.00
**GT22**	0.00	0.00	0.00
**PL12**	0.05	0.26	0.05
**PL18**	0.00	0.00	0.00
**GT92**	0.00	0.00	0.00
**GT44**	0.00	0.00	0.00
**GT29**	0.00	0.00	0.00
**GT13**	0.00	0.00	0.00
**GT52**	0.00	0.00	0.00
**GT64**	0.00	0.00	0.00
**GT85**	0.00	0.00	0.00
**GT16**	0.00	0.00	0.00
**GH56**	0.00	0.00	0.00
**GT60**	0.00	0.00	0.00
**GH41**	0.00	0.00	0.00
**CE7**	0.16	0.12	0.02
**GH39**	0.08	0.02	0.02
**GT30**	0.24	0.44	0.20
**PL15**	0.03	0.22	0.06
**GH102**	0.03	0.00	0.02
