# Supplementary material for: Global Profiling of Carbohydrate Active Enzymes in Human Gut Microbiome
Source: PLoS One. 2015 Nov 6;10(11):e0142038. doi: 10.1371/journal.pone.0142038 (PMC4636310; doi:10.1371/journal.pone.0142038)

CALCULATION OF GINI COEFFICIENT FROM THE CAZYME PROFILE OF A METAGENOME

The following document provides an example of how the Gini coefficient of a hypothetical microbiome (M1) is computed from its CAZyme hit profile.

Step 1: This step consists of obtaining the normalized abundance of each detected CAZyme family in M1. This is achieved by dividing the number of hits obtained for each CAZyme family by the metagenome size. For example, consider the normalized abundance of the various CAZyme families detected in M1 as below:

CAZyme Family	Normalized Abundance
GH1	0.01
GH13	0.05
GH4	0.10
GH2	0.02

Step 2: The CAZyme families are then sorted in the increasing order (of their normalized abundances

CAZyme Family	Normalized Abundance
GH1	0.01
GH2	0.02
GH13	0.05
GH4	0.10

Step 3: The cumulative abundance is then obtained corresponding to each CAZyme family (in the serial order of their ranked abundances). For each CAZyme family, the Cumulative CAZyme abundance is calculated by adding the abundance of the corresponding family to that of all the other families having abundance values below it in the given metagenome M1. The following table clarifies this aspect.

Sl. No	CAZyme Family	Normalized Abundance	Cumulative Abundance
1	GH1	0.01	0.01
2	GH2	0.02	0.03
3	GH13	0.05	0.08
4	GH4	0.10	0.18

Subsequently, the rank of each family is plotted against the cumulative abundance. The obtained blue curve is shown in the figure below:

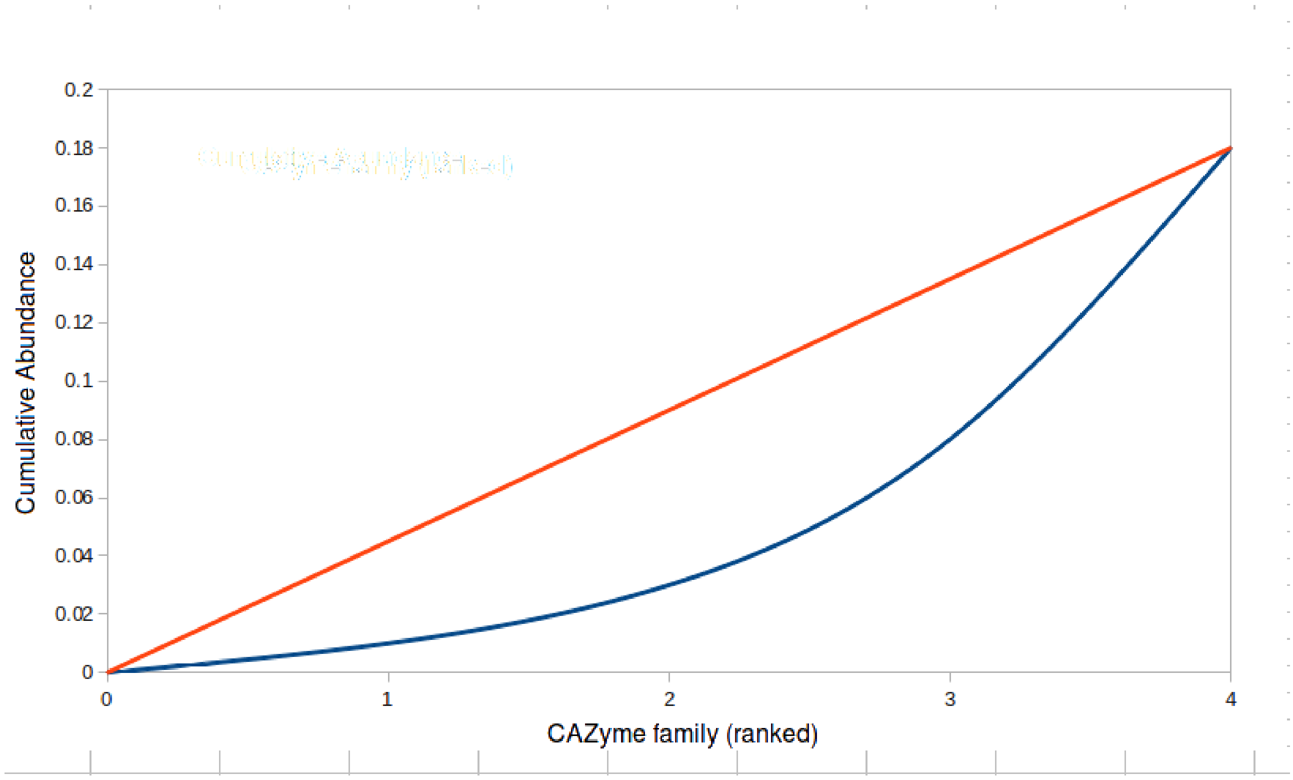

In the above figure, the diagonal line (shown in red) is indicative of a ideal scenario of complete equality wherein all the CAZyme families contribute to an equal extent (in other words, are of similar abundances). However, given the inequality in the representation of the various CAZyme families in the current scenario, the obtained curve (of cumulative abundance v/s rank of the CAZyme family), shown in blue, deviates from this diagonal line. With an increase in the inequality of representation in the metagenome, the deviation of this curve from the diagonal line will increase. This result in increasing the area bound by the diagonal line and the curve of the cumulatie abundances (shaded in red in the figure below).

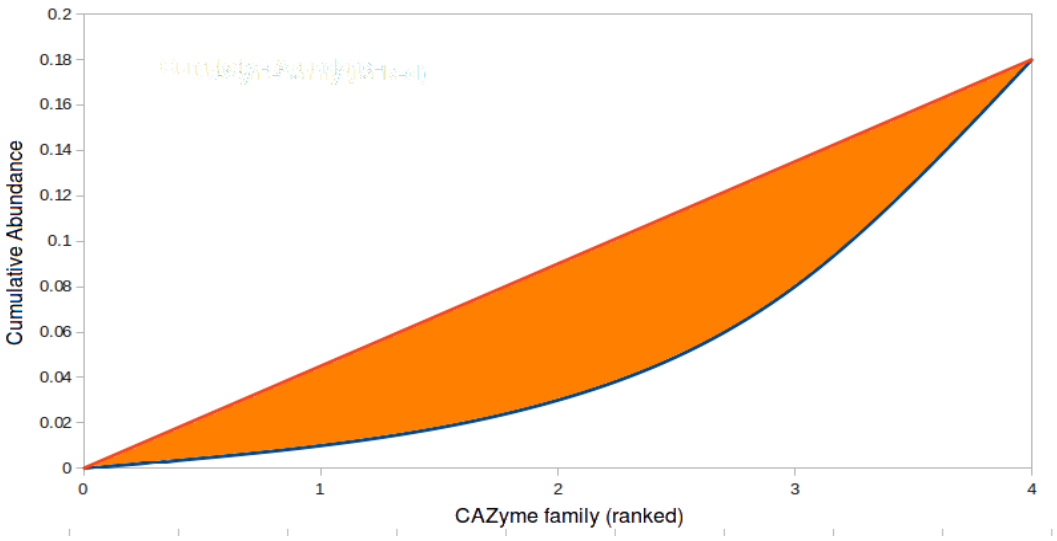

Obtaining the quantitative measure of this area is thus indicative of the inequality of representation in M1. This is used to calculate the Gini coefficient as described below:

Step 4: The last step involves the calculation of Gini coefficient considering two areas A and B, where in:

A denotes the area bound by the diagonal line and the curve of the cumulative abundances (shaded in red in the figure below) and,

B denotes the area bound by the curve and the two axes (shaded in cyan color).

The Gini coefficient of M1 is computed as A / (A+B).

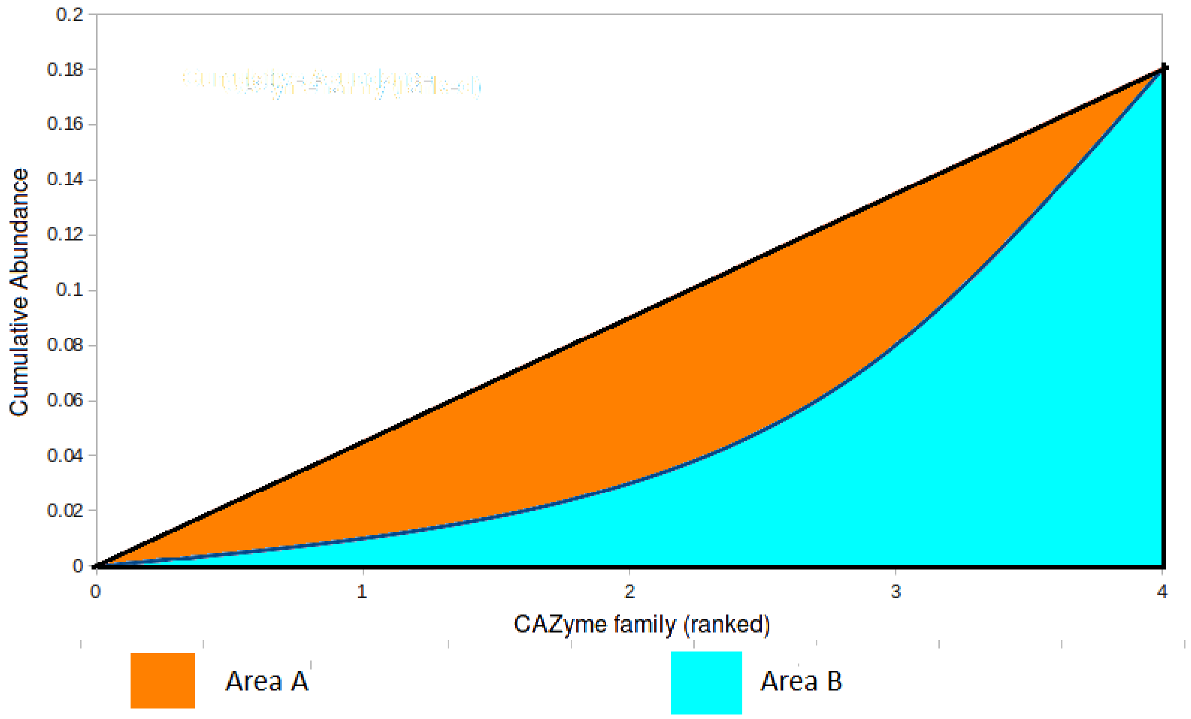

Supplement: S1 Text — (PDF) [file pone.0142038.s011.pdf]
