# Supplementary material for: Global Profiling of Carbohydrate Active Enzymes in Human Gut Microbiome
Source: PLoS One. 2015 Nov 6;10(11):e0142038. doi: 10.1371/journal.pone.0142038 (PMC4636310; doi:10.1371/journal.pone.0142038)
Supplement: S2 Text — (PDF) [file pone.0142038.s012.pdf]

Supplementary Text S2

Investigation of the CAZotype predictive ability of the CAZyme and taxonomic profiles using Partial Least Square (PLS) Regression

We performed two different PLS regression analyses for this purpose.

Analysis 1

In this analysis, we evaluated the ability of gut CAZyme abundance profiles (predictors) of the ndividuals for the prediction of the CAZotypes (response) obtained earlier (using the BCA approach) for the corresponding individuals.

Objective: The objective was to obtain a cross validation of how good the CAZyme profiles are for the prediction of CAZotypes, as well as to identify the key CAZymes that have the best predictive power for CAZotype classification.

Methods: The PLS regression analyses were performed using plsdepot package of the R programming interface with the CAZyme abundance profiles as the predictor variables and the CAZotype as the response. The predictive ability of the predictor variables for the response was evaluated using two parameters, namely the correlation of the three PLS components with the response and the fraction variance of the response explained by each PLS component.

In order to identify CAZyme families with the most predictive power, the following approach was adopted. For each PLS component, the coefficient weights for the various CAZyme families were first obtained. CAZymes with the most positive weights (top 15) and the most negative weights (top 15 with the most negative weight) were then selected as the ones with the highest predictive power. This was done for all the PLS components. Subsequently, a unique of CAZyme was prepared containing CAZymes with the most predictive ability in either of the three PLS Components.

Results: The PLS regression indicated that the two PLS components t1 and t2, obtained were observed to have good correlation values (0.845 and 0.792, respectively) with the response (i.e. CAZotypes). The percentage of variance of response explained by the three PLS components was also observed to be high, ranging from 0.707 (for component t1) to 0.758 (for component t3).

Table 1 provides the list of predictive CAZymes obtained in the PLS regression analysis.

Table 1: PLS component weights for the list of predictive CAZymes

CAZyme	Weight (t1)	Weight (t2)	Weight (t3)	CAZotype Specificity
GH115	0.1073	0.0179	-0.0294	CT1-Specific
GH2	0.1574	0.0257	-0.0550	CT1-Specific
GH28	0.1291	0.0778	0.0829	CT1-Specific
GH43	0.1344	-0.0046	0.0029	CT1-Specific
GH76	0.0486	0.0789	-0.1889	CT1-Specific
GH78	0.0793	0.1517	-0.0073	CT1-Specific
GH88	0.0624	0.2096	0.0806	CT1-Specific
GH92	0.0830	0.0934	-0.1113	CT1-Specific
GH97	0.1044	-0.0167	-0.0986	CT1-Specific
PL1	0.0780	0.1114	0.0147	CT1-Specific
PL8	0.0480	0.1750	0.0036	CT1-Specific
GH23	0.1363	0.0135	0.0662	CT2-Specific
GH24	0.0135	-0.1991	-0.2249	CT2-Specific
GH31	0.1411	-0.0308	-0.0212	CT2-Specific
GH4	0.0598	0.0157	0.1527	CT2-Specific
GH73	0.1159	-0.0481	0.0223	CT2-Specific
GH101	0.0664	-0.0932	-0.1305	CT3-Specific
GH129	0.0977	0.0785	0.1421	CT3-Specific
GH29	0.1411	0.1094	0.1190	CT3-Specific
GH32	0.1304	-0.0162	0.0769	CT3-Specific
GH33	0.1076	0.0563	0.0553	CT3-Specific
GH36	0.1421	-0.0164	0.0481	CT3-Specific
GH38	0.1038	0.1534	0.2593	CT3-Specific
GH42	0.1114	0.0962	0.2061	CT3-Specific
GH5	0.1268	0.0598	0.1911	CT3-Specific
GH51	0.1464	-0.0291	0.0058	CT3-Specific
GH77	0.1332	0.0487	0.1488	CT3-Specific
GH95	0.1549	-0.0061	-0.0251	CT3-Specific
GH20	0.1395	0.0618	-0.0063	CT3/CT1-Specific
GH13	0.1331	0.0213	0.1339	CT3/CT2-Specific
GH25	0.1145	-0.0277	0.1053	CT3/CT2-Specific
GH102	0.0191	-0.1643	-0.1333	
GH105	0.0858	0.1412	0.0331	
GH110	0.0874	-0.0662	-0.2562	
GH112	0.1070	0.0920	0.1840	
GH116	0.0074	0.1198	-0.0127	
GH117	0.0388	0.0851	-0.1466	
GH125	0.1369	0.1608	0.1942	
GH127	0.1050	-0.1180	-0.1762	
GH16	0.0877	-0.0119	-0.1583	
GH18	0.1236	0.2287	0.2397	
GH27	0.0804	-0.0312	-0.1653	
GH3	0.1517	0.0066	0.0003	
GH30	0.1220	0.1005	0.0812	
GH35	0.1225	-0.0108	-0.1303	
GH37	0.0192	-0.1234	-0.0704	
GH50	0.0435	0.0359	-0.1780	
GH55	0.0188	0.1327	0.1090	
GH63	0.0693	-0.0560	-0.1310	
GH72	0.1329	-0.0301	0.0527	
GH84	0.0864	-0.1056	-0.1772	
GH89	0.1058	-0.0505	-0.2213	
GH91	0.0133	0.0398	-0.1177	
GH99	0.0230	0.0178	-0.1060	
PL12	0.0466	0.2231	0.0886	
PL13	0.0430	0.2061	0.0521	
PL15	0.0469	0.1921	0.0464	
PL21	0.0110	0.1431	0.0804	
PL9	0.0438	-0.1150	-0.2035	

A total of 59 CAZyme families were identified to have good predictive abilities for the CAZotypes. Interestingly, this list of CAZyme families was also observed to contain 12 (out of the 14), seven (out of the nine) and 15 (out of the 17) CAZyme families identified earlier (using Welch's t-test) to be significantly over-abundant in CAZotypes 1, 2 and 3 respectively (S5 Fig). This result further validates that these sets of over-abundant CAZymes act as drivers for the different CAZotypes.

Analysis 2

Objective: The objective was to investigate whether the taxonomic composition of the gut microbiomes can have enough predictive ability for the CAZotype of an individual.

Methods: Three different gut taxonomic profiles of the individuals were obtained at the levels of phylum, order and genus, respectively. The three profiles were then individually used as predictors for the CAZotypes of the corresponding individuals. The PLS regression analyses were performed using plsdepot package of the R programming interface. The predictive ability of the predictor variables for the response was evaluated using two parameters, namely the correlation of the three PLS components with the response and the fraction variance of the response explained by each PLS component.

Results: Table 2 shows the CAZotype predictive ability of the taxonomic profiles at the taxonomic levels of phylum, order, and genus.

Table 2 (a) Correlation of the PLS components with the response variables for the three different scenarios (b) Fraction of variance explained by each PLS component for the response variables for the different scenarios.

(A)

Predictors	Correlation of the PLS Components		
	t1	t2	t3
Taxonomic Profile at Phylum Level	0.257	0.038	0.025
Taxonomic Profile at Order Level	0.284	0.077	0.031
Taxonomic Profile at Genus Level	0.684	0.41	0.31

(B)

Predictors	Variance within Response explained		
	t1	t2	t3
Taxonomic Profile at Phylum Level	0.067	0.07	0.064
Taxonomic Profile at Order Level	0.115	0.113	0.112
Taxonomic Profile at Genus Level	0.681	0.51	0.36

As observed in Table 2, although the predictive power of the taxonomic profiles is low at the non-specific levels of phylum and order, it increases noticeably at the specific level of genus. The above results suggest that specific microbial groups tend to harbor specific groups of CAZymes, which in turn influence the CAZotype of an individual. The lower predictive ability at non-specific levels may indicate a high degree of functional heterogeneity within microbial genera/species within a specific broader group of phylum or order, leading to lower predictive power of profiles at these taxonomic levels.
